# Global, regional, and national burden of stroke and its risk factors, 1990–2019: a systematic analysis for the Global Burden of Disease Study 2019

**DOI:** 10.1016/S1474-4422(21)00252-0

**Published:** 2021-10

**Authors:** GBD 2019 Stroke Collaborators, Valery L Feigin, Benjamin A Stark, Catherine Owens Johnson, Gregory A Roth, Catherine Bisignano, Gdiom Gebreheat Abady, Mitra Abbasifard, Mohsen Abbasi-Kangevari, Foad Abd-Allah, Vida Abedi, Ahmed Abualhasan, Niveen ME Abu-Rmeileh, Abdelrahman I Abushouk, Oladimeji M Adebayo, Gina Agarwal, Pradyumna Agasthi, Bright Opoku Ahinkorah, Sohail Ahmad, Sepideh Ahmadi, Yusra Ahmed Salih, Budi Aji, Samaneh Akbarpour, Rufus Olusola Akinyemi, Hanadi Al Hamad, Fares Alahdab, Sheikh Mohammad Alif, Vahid Alipour, Syed Mohamed Aljunid, Sami Almustanyir, Rajaa M Al-Raddadi, Rustam Al-Shahi Salman, Nelson Alvis-Guzman, Robert Ancuceanu, Deanna Anderlini, Jason A Anderson, Adnan Ansar, Ippazio Cosimo Antonazzo, Jalal Arabloo, Johan Ärnlöv, Kurnia Dwi Artanti, Zahra Aryan, Samaneh Asgari, Tahira Ashraf, Mohammad Athar, Alok Atreya, Marcel Ausloos, Atif Amin Baig, Ovidiu Constantin Baltatu, Maciej Banach, Miguel A Barboza, Suzanne Lyn Barker-Collo, Till Winfried Bärnighausen, Mark Thomaz Ugliara Barone, Sanjay Basu, Gholamreza Bazmandegan, Ettore Beghi, Mahya Beheshti, Yannick Béjot, Arielle Wilder Bell, Derrick A Bennett, Isabela M Bensenor, Woldesellassie Mequanint Bezabhe, Yihienew Mequanint Bezabih, Akshaya Srikanth Bhagavathula, Pankaj Bhardwaj, Krittika Bhattacharyya, Ali Bijani, Boris Bikbov, Mulugeta M Birhanu, Archith Boloor, Aime Bonny, Michael Brauer, Hermann Brenner, Dana Bryazka, Zahid A Butt, Florentino Luciano Caetano dos Santos, Ismael R Campos-Nonato, Carlos Cantu-Brito, Juan J Carrero, Carlos A Castañeda-Orjuela, Alberico L Catapano, Promit Ananyo Chakraborty, Jaykaran Charan, Sonali Gajanan Choudhari, Enayet Karim Chowdhury, Dinh-Toi Chu, Sheng-Chia Chung, David Colozza, Vera Marisa Costa, Simona Costanzo, Michael H Criqui, Omid Dadras, Baye Dagnew, Xiaochen Dai, Koustuv Dalal, Albertino Antonio Moura Damasceno, Emanuele D'Amico, Lalit Dandona, Rakhi Dandona, Jiregna Darega Gela, Kairat Davletov, Vanessa De la Cruz-Góngora, Rupak Desai, Deepak Dhamnetiya, Samath Dhamminda Dharmaratne, Mandira Lamichhane Dhimal, Meghnath Dhimal, Daniel Diaz, Martin Dichgans, Klara Dokova, Rajkumar Doshi, Abdel Douiri, Bruce B Duncan, Sahar Eftekharzadeh, Michael Ekholuenetale, Nevine El Nahas, Islam Y Elgendy, Muhammed Elhadi, Shaimaa I El-Jaafary, Matthias Endres, Aman Yesuf Endries, Daniel Asfaw Erku, Emerito Jose A Faraon, Umar Farooque, Farshad Farzadfar, Abdullah Hamid Feroze, Irina Filip, Florian Fischer, David Flood, Mohamed M Gad, Shilpa Gaidhane, Reza Ghanei Gheshlagh, Ahmad Ghashghaee, Nermin Ghith, Ghozali Ghozali, Sherief Ghozy, Alessandro Gialluisi, Simona Giampaoli, Syed Amir Gilani, Paramjit Singh Gill, Elena V Gnedovskaya, Mahaveer Golechha, Alessandra C Goulart, Yuming Guo, Rajeev Gupta, Veer Bala Gupta, Vivek Kumar Gupta, Pradip Gyanwali, Nima Hafezi-Nejad, Samer Hamidi, Asif Hanif, Graeme J Hankey, Arief Hargono, Abdiwahab Hashi, Treska S Hassan, Hamid Yimam Hassen, Rasmus J Havmoeller, Simon I Hay, Khezar Hayat, Mohamed I Hegazy, Claudiu Herteliu, Ramesh Holla, Sorin Hostiuc, Mowafa Househ, Junjie Huang, Ayesha Humayun, Bing-Fang Hwang, Licia Iacoviello, Ivo Iavicoli, Segun Emmanuel Ibitoye, Olayinka Stephen Ilesanmi, Irena M Ilic, Milena D Ilic, Usman Iqbal, Seyed Sina Naghibi Irvani, Sheikh Mohammed Shariful Islam, Nahlah Elkudssiah Ismail, Hiroyasu Iso, Gaetano Isola, Masao Iwagami, Louis Jacob, Vardhmaan Jain, Sung-In Jang, Sathish Kumar Jayapal, Shubha Jayaram, Ranil Jayawardena, Panniyammakal Jeemon, Ravi Prakash Jha, Walter D Johnson, Jost B Jonas, Nitin Joseph, Jacek Jerzy Jozwiak, Mikk Jürisson, Rizwan Kalani, Rohollah Kalhor, Yogeshwar Kalkonde, Ashwin Kamath, Zahra Kamiab, Tanuj Kanchan, Himal Kandel, André Karch, Patrick DMC Katoto, Gbenga A Kayode, Pedram Keshavarz, Yousef Saleh Khader, Ejaz Ahmad Khan, Imteyaz A Khan, Maseer Khan, Moien AB Khan, Mahalaqua Nazli Khatib, Jagdish Khubchandani, Gyu Ri Kim, Min Seo Kim, Yun Jin Kim, Adnan Kisa, Sezer Kisa, Mika Kivimäki, Dhaval Kolte, Ali Koolivand, Sindhura Lakshmi Koulmane Laxminarayana, Ai Koyanagi, Kewal Krishan, Vijay Krishnamoorthy, Rita V Krishnamurthi, G Anil Kumar, Dian Kusuma, Carlo La Vecchia, Ben Lacey, Hassan Mehmood Lak, Tea Lallukka, Savita Lasrado, Pablo M Lavados, Matilde Leonardi, Bingyu Li, Shanshan Li, Hualiang Lin, Ro-Ting Lin, Xuefeng Liu, Warren David Lo, Stefan Lorkowski, Giancarlo Lucchetti, Ricardo Lutzky Saute, Hassan Magdy Abd El Razek, Francesca Giulia Magnani, Preetam Bhalchandra Mahajan, Azeem Majeed, Alaa Makki, Reza Malekzadeh, Ahmad Azam Malik, Navid Manafi, Mohammad Ali Mansournia, Lorenzo Giovanni Mantovani, Santi Martini, Giampiero Mazzaglia, Man Mohan Mehndiratta, Ritesh G Menezes, Atte Meretoja, Amanual Getnet Mersha, Junmei Miao Jonasson, Bartosz Miazgowski, Tomasz Miazgowski, Irmina Maria Michalek, Erkin M Mirrakhimov, Yousef Mohammad, Abdollah Mohammadian-Hafshejani, Shafiu Mohammed, Ali H Mokdad, Yaser Mokhayeri, Mariam Molokhia, Mohammad Ali Moni, Ahmed Al Montasir, Rahmatollah Moradzadeh, Lidia Morawska, Jakub Morze, Walter Muruet, Kamarul Imran Musa, Ahamarshan Jayaraman Nagarajan, Mohsen Naghavi, Sreenivas Narasimha Swamy, Bruno Ramos Nascimento, Ruxandra Irina Negoi, Sandhya Neupane Kandel, Trang Huyen Nguyen, Bo Norrving, Jean Jacques Noubiap, Vincent Ebuka Nwatah, Bogdan Oancea, Oluwakemi Ololade Odukoya, Andrew T Olagunju, Hans Orru, Mayowa O Owolabi, Jagadish Rao Padubidri, Adrian Pana, Tarang Parekh, Eun-Cheol Park, Fatemeh Pashazadeh Kan, Mona Pathak, Mario F P Peres, Arokiasamy Perianayagam, Truong-Minh Pham, Michael A Piradov, Vivek Podder, Suzanne Polinder, Maarten J Postma, Akram Pourshams, Amir Radfar, Alireza Rafiei, Alberto Raggi, Fakher Rahim, Vafa Rahimi-Movaghar, Mosiur Rahman, Muhammad Aziz Rahman, Amir Masoud Rahmani, Nazanin Rajai, Priyanga Ranasinghe, Chythra R Rao, Sowmya J Rao, Priya Rathi, David Laith Rawaf, Salman Rawaf, Marissa B Reitsma, Vishnu Renjith, Andre M N Renzaho, Aziz Rezapour, Jefferson Antonio Buendia Rodriguez, Leonardo Roever, Michele Romoli, Andrzej Rynkiewicz, Simona Sacco, Masoumeh Sadeghi, Sahar Saeedi Moghaddam, Amirhossein Sahebkar, KM Saif-Ur-Rahman, Rehab Salah, Mehrnoosh Samaei, Abdallah M Samy, Itamar S Santos, Milena M Santric-Milicevic, Nizal Sarrafzadegan, Brijesh Sathian, Davide Sattin, Silvia Schiavolin, Markus P Schlaich, Maria Inês Schmidt, Aletta Elisabeth Schutte, Sadaf G Sepanlou, Allen Seylani, Feng Sha, Saeed Shahabi, Masood Ali Shaikh, Mohammed Shannawaz, Md Shajedur Rahman Shawon, Aziz Sheikh, Sara Sheikhbahaei, Kenji Shibuya, Soraya Siabani, Diego Augusto Santos Silva, Jasvinder A Singh, Jitendra Kumar Singh, Valentin Yurievich Skryabin, Anna Aleksandrovna Skryabina, Badr Hasan Sobaih, Stefan Stortecky, Saverio Stranges, Eyayou Girma Tadesse, Ingan Ukur Tarigan, Mohamad-Hani Temsah, Yvonne Teuschl, Amanda G Thrift, Marcello Tonelli, Marcos Roberto Tovani-Palone, Bach Xuan Tran, Manjari Tripathi, Gebiyaw Wudie Tsegaye, Anayat Ullah, Brigid Unim, Bhaskaran Unnikrishnan, Alireza Vakilian, Sahel Valadan Tahbaz, Tommi Juhani Vasankari, Narayanaswamy Venketasubramanian, Dominique Vervoort, Bay Vo, Victor Volovici, Kia Vosoughi, Giang Thu Vu, Linh Gia Vu, Hatem A Wafa, Yasir Waheed, Yanzhong Wang, Tissa Wijeratne, Andrea Sylvia Winkler, Charles D A Wolfe, Mark Woodward, Jason H Wu, Sarah Wulf Hanson, Xiaoyue Xu, Lalit Yadav, Ali Yadollahpour, Seyed Hossein Yahyazadeh Jabbari, Kazumasa Yamagishi, Hiroshi Yatsuya, Naohiro Yonemoto, Chuanhua Yu, Ismaeel Yunusa, Muhammed Shahriar Zaman, Sojib Bin Zaman, Maryam Zamanian, Ramin Zand, Alireza Zandifar, Mikhail Sergeevich Zastrozhin, Anasthasia Zastrozhina, Yunquan Zhang, Zhi-Jiang Zhang, Chenwen Zhong, Yves Miel H Zuniga, Christopher J L Murray

## Abstract

**Background:**

Regularly updated data on stroke and its pathological types, including data on their incidence, prevalence, mortality, disability, risk factors, and epidemiological trends, are important for evidence-based stroke care planning and resource allocation. The Global Burden of Diseases, Injuries, and Risk Factors Study (GBD) aims to provide a standardised and comprehensive measurement of these metrics at global, regional, and national levels.

**Methods:**

We applied GBD 2019 analytical tools to calculate stroke incidence, prevalence, mortality, disability-adjusted life-years (DALYs), and the population attributable fraction (PAF) of DALYs (with corresponding 95% uncertainty intervals [UIs]) associated with 19 risk factors, for 204 countries and territories from 1990 to 2019. These estimates were provided for ischaemic stroke, intracerebral haemorrhage, subarachnoid haemorrhage, and all strokes combined, and stratified by sex, age group, and World Bank country income level.

**Findings:**

In 2019, there were 12·2 million (95% UI 11·0–13·6) incident cases of stroke, 101 million (93·2–111) prevalent cases of stroke, 143 million (133–153) DALYs due to stroke, and 6·55 million (6·00–7·02) deaths from stroke. Globally, stroke remained the second-leading cause of death (11·6% [10·8–12·2] of total deaths) and the third-leading cause of death and disability combined (5·7% [5·1–6·2] of total DALYs) in 2019. From 1990 to 2019, the absolute number of incident strokes increased by 70·0% (67·0–73·0), prevalent strokes increased by 85·0% (83·0–88·0), deaths from stroke increased by 43·0% (31·0–55·0), and DALYs due to stroke increased by 32·0% (22·0–42·0). During the same period, age-standardised rates of stroke incidence decreased by 17·0% (15·0–18·0), mortality decreased by 36·0% (31·0–42·0), prevalence decreased by 6·0% (5·0–7·0), and DALYs decreased by 36·0% (31·0–42·0). However, among people younger than 70 years, prevalence rates increased by 22·0% (21·0–24·0) and incidence rates increased by 15·0% (12·0–18·0). In 2019, the age-standardised stroke-related mortality rate was 3·6 (3·5–3·8) times higher in the World Bank low-income group than in the World Bank high-income group, and the age-standardised stroke-related DALY rate was 3·7 (3·5–3·9) times higher in the low-income group than the high-income group. Ischaemic stroke constituted 62·4% of all incident strokes in 2019 (7·63 million [6·57–8·96]), while intracerebral haemorrhage constituted 27·9% (3·41 million [2·97–3·91]) and subarachnoid haemorrhage constituted 9·7% (1·18 million [1·01–1·39]). In 2019, the five leading risk factors for stroke were high systolic blood pressure (contributing to 79·6 million [67·7–90·8] DALYs or 55·5% [48·2–62·0] of total stroke DALYs), high body-mass index (34·9 million [22·3–48·6] DALYs or 24·3% [15·7–33·2]), high fasting plasma glucose (28·9 million [19·8–41·5] DALYs or 20·2% [13·8–29·1]), ambient particulate matter pollution (28·7 million [23·4–33·4] DALYs or 20·1% [16·6–23·0]), and smoking (25·3 million [22·6–28·2] DALYs or 17·6% [16·4–19·0]).

**Interpretation:**

The annual number of strokes and deaths due to stroke increased substantially from 1990 to 2019, despite substantial reductions in age-standardised rates, particularly among people older than 70 years. The highest age-standardised stroke-related mortality and DALY rates were in the World Bank low-income group. The fastest-growing risk factor for stroke between 1990 and 2019 was high body-mass index. Without urgent implementation of effective primary prevention strategies, the stroke burden will probably continue to grow across the world, particularly in low-income countries.

**Funding:**

Bill & Melinda Gates Foundation.

## Introduction

Disease and population distribution patterns, life expectancy, mortality, causes of death, and socio-demographic factors continue to change across the world, including ageing of populations and changes in the prevalence of risk factors for non-communicable disorders. Timely estimates of the burden of stroke and its pathological types, the burden attributable to risk factors, and trends in the burden over time are necessary at the global, regional, and national levels to guide evidence-based health-care policy, planning, and resource allocation for stroke.

**Research in context**

**Evidence before this study**

The Global Burden of Diseases, Injuries, and Risk Factors Study (GBD) produces the most comprehensive estimates of the global, regional, and country-specific burden of stroke. Population-level estimates for stroke incidence or mortality have been published by WHO and independent research groups, but those of GBD include more extensive estimates by age, sex, location, and year. To evaluate the availability of evidence, we did a structured review of the published scientific literature in Medline, Scopus, Google Scholar, and PubMed for relevant reports published in any language up to June 30, 2021, using search terms that included “stroke”, “cerebral infarction”, “isch(a)emic stroke”, “intracerebral h(a)emorrage”, “h(a)emorrhagic stroke”, or “subarachnoid h(a)emorrage”, AND “incidence”, “prevalence”, “mortality”, or “epidemiology” or “population attributable fraction (PAF)”, “risk factor(s)”, or “disability-adjusted life-year(s) (DALYs)”. GBD 2017 included stroke in its analysis, but the most recent paper by the GBD Collaborator Network on the topic of stroke was from GBD 2016. The report concluded that because the decrease in global age-standardised incidence rates from 1990 to 2016 was minimal, the burden of stroke was likely to remain high well into the future.

**Added value of this study**

As part of GBD 2019, this study provides updated estimates of the burden of overall stroke, ischaemic stroke, intracerebral haemorrhage, and subarachnoid haemorrhage for 204 countries and territories in 21 GBD regions from 1990 to 2019, by age, sex, and country income level (by the World Bank classification). Stroke burden was measured by incidence, prevalence, mortality, and DALYs as well as the PAF of stroke-related DALYs associated with potentially modifiable behavioural, environmental and occupational, and metabolic risk factors or risk factor clusters. Until GBD 2017, intracerebral haemorrhage and subarachnoid haemorrhage were not estimated separately, so this is the first report by the GBD Collaborator Network to present the global, regional, and national burden of haemorrhagic strokes by intracerebral haemorrhage and subarachnoid haemorrhage separately. This study is also the first systematic analysis to determine the effect of non-optimal temperature on stroke burden.

**Implications of all the available evidence**

The findings from this study can help guide evidence-based health-care planning, prevention, and resource allocation for stroke and its pathological types, including country-specific prioritisation of these measures. By evaluating the risk-attributable burden of different stroke types in different geographical locations, this study can be used to develop location-specific strategies for reducing the burden of stroke. Based on the available evidence, public health and research priorities should include: expanding evidence-based prevention strategies that reduce exposure to stroke risk factors; reducing the gaps in acute and chronic stroke prevention, screening, and treatment services between high-income and low-income to middle-income countries; and further epidemiological research on stroke risk and outcomes across different countries and populations.

The Global Burden of Diseases, Injuries, and Risk Factors Study (GBD) 2017 showed that stroke was the third-leading cause of death and disability combined (as measured by disability-adjusted life-years [DALYs]) and the second-leading cause of death in the world in 2017.^1^, ^2^ A GBD 2017 stroke analysis found that, although age-standardised mortality rates for stroke decreased sharply from 1990 to 2017,^2^ the decrease in age-standardised incidence was much less steep, suggesting that prevention efforts have been less successful than treatment efforts. The results from GBD 2016^3^ showed that 87·9% of ischaemic stroke DALYs and 89·5% of haemorrhagic stroke DALYs were due to potentially modifiable risk factors measured in GBD, demonstrating the enormous potential to reduce the burden of stroke through reductions in risk factor exposure. According to WHO, effective stroke prevention strategies include reducing the risk associated with hypertension (high systolic blood pressure), elevated lipids, diabetes (high fasting plasma glucose), smoking, low physical activity, unhealthy diet, and abdominal obesity (high body-mass index [BMI]),^4^ which is similar to the findings from GBD 2016^3^ and GBD 2017.^5^

In this study, we estimated the global, regional, and national burden of overall stroke, ischaemic stroke, intracerebral haemorrhage, and subarachnoid haemorrhage in terms of their incidence, prevalence, mortality, and DALYs, as well as stroke-related DALYs associated with 19 potentially modifiable behavioural, environmental and occupational, and metabolic risk factors or groups of risk factors. We present data for 204 countries and territories, 21 GBD regions, and four World Bank income level groups from 1990 to 2019, by age group and sex. This manuscript was produced as part of the GBD Collaborator Network and in accordance with the GBD Protocol.

## Methods

### Overview and case definition

Details of the GBD 2019 eligibility criteria, the literature search strategy, and data extraction are described in detail elsewhere^6^, ^7^ (appendix sections 1.1–4.3). In brief, stroke was defined by WHO clinical criteria^8^ as rapidly developing clinical signs of (usually focal) disturbance of cerebral function lasting more than 24 h or leading to death. Ischaemic stroke was defined as an episode of neurological dysfunction due to focal cerebral, spinal, or retinal infarction. Intracerebral haemorrhage was defined as stroke with a focal collection of blood in the brain not due to trauma. Subarachnoid haemorrhage was defined as non-traumatic stroke due to bleeding into the subarachnoid space of the brain. The GBD methods for assigning cause of death to stroke and stroke subtypes in regions where neuroimaging was not available have been previously described.^9^ GBD classifies causes into four levels, from the broadest (Level 1; eg, non-communicable diseases), to the most specific (Level 4; eg, intracerebral haemorrhage). Stroke is a Level 3 cause, within the Level 2 category of cardiovascular diseases, while its subtypes are Level 4 causes.

### Fatal disease modelling

We used vital registration and verbal autopsy data as inputs into the Cause of Death Ensemble modelling (CODEm) framework to estimate deaths due to overall stroke and stroke subtypes. CODEm is a flexible modelling tool that utilises geospatial relationships and information from covariates to produce estimates of death for all locations across the time series (1990–2019). Deaths from vital registration systems coded to impossible or intermediate causes of death or unspecified stroke were reassigned by use of statistical methods (appendix sections 1.4, 1.7, 1.8).^10^

### Non-fatal disease modelling

Estimates of the incidence and prevalence of stroke were generated with the DisMod-MR 2.1 (disease-model-Bayesian meta-regression) modelling tool.^3^ DisMod-MR is a Bayesian geospatial disease modelling software that uses data on various disease parameters, the epidemiological relationships between these parameters, and geospatial relationships to produce estimates of prevalence and incidence (appendix section 3). All available high-quality data on incidence, prevalence, and mortality were used to estimate non-fatal stroke burden. We modelled first-ever ischaemic stroke, intracerebral haemorrhage, and subarachnoid haemorrhage from the day of incidence through 28 days and separately modelled survival beyond 28 days.

### Risk factor estimation

To analyse the attributable burden of stroke due to 19 risk factors currently available for such analysis in GBD 2019, we calculated population attributable fractions (PAFs) of DALYs (appendix section 2).^7^ This work was done within the comparative risk assessments framework of GBD by use of four datasets: the burden estimates for stroke and its three pathological types; the exposure level for each risk factor; the relative risk of stroke as an outcome of exposure to the risk factor; and the theoretical minimum risk exposure level (TMREL), which is the level of exposure that minimises risk for each individual in the population.^11^ The relative risks included in this analysis were generated from meta-analyses of epidemiological studies reporting associations between the risk factors of interest and stroke; these analyses are not stroke-type specific. The PAF (estimated independently for each risk factor) is the proportion of the cause that would be decreased if the exposure to the risk factor in the past had been reduced to the counterfactual level of the TMREL.

Risks included in the analysis were ambient particulate matter pollution; household air pollution from solid fuels; non-optimal temperature—ie, low temperature (daily temperatures below the TMREL) and high temperature (daily temperatures above the TMREL); lead exposure; diet high in sodium; diet high in red meat; diet low in fruits; diet low in vegetables; diet low in whole grains; alcohol consumption (any dosage); low physical activity (only for ischaemic stroke burden); smoking; secondhand smoke; high BMI; high fasting plasma glucose; high systolic blood pressure; high LDL cholesterol (only for ischaemic stroke burden); and kidney dysfunction, as measured by low glomerular filtration rate (GFR; not assessed for subarachnoid haemorrhage burden). As with causes, GBD organises risk factors into four levels, from the broadest (Level 1) to the most specific (Level 4). In addition to the specific risk factors above, we assessed the Level 1 groups of risks: behavioural, environmental and occupational, and metabolic. The PAFs of risk factor groups took into account interactions between risk factors included in the group, as explained elsewhere.^12^ Percentages and number of DALYs are not mutually exclusive. The crude sum of the PAF of the risk factors might exceed 100% because the effects of many of these risk factors are mediated partly or wholly through another risk factor or risk factors. Definitions of risk factors and risk groups and further details of risk factors are provided in the appendix (section 2.1).

### Data sources and presentation

For GBD 2019, we used data from 3686 vital registration sources, 147 verbal autopsy sources, 368 incidence sources, 117 prevalence sources, 229 excess mortality sources, 7753 risk factor exposure sources, and 2733 risk factor relative risk sources. Further details of the data sources used in this analysis are available on the Global Health Data Exchange website.

Estimates in this Article are presented in absolute numbers and as age-standardised rates per 100 000 population (with 95% uncertainty intervals [UIs]) and are stratified by age, sex, 21 GBD regions, seven GBD super-regions (appendix figure 6.1), and four income levels (as determined by the World Bank).^13^ Count data are presented in tables to two decimal places (and rounded to one decimal place in the text), and percentage data (including percentage change) are presented to one decimal place.

### Role of the funding source

The funder had no role in study design, data collection, data analysis, interpretation of the study results, writing of the report, or the decision to submit the manuscript for publication.

## Results

### Overall stroke burden

**Figure 1:**
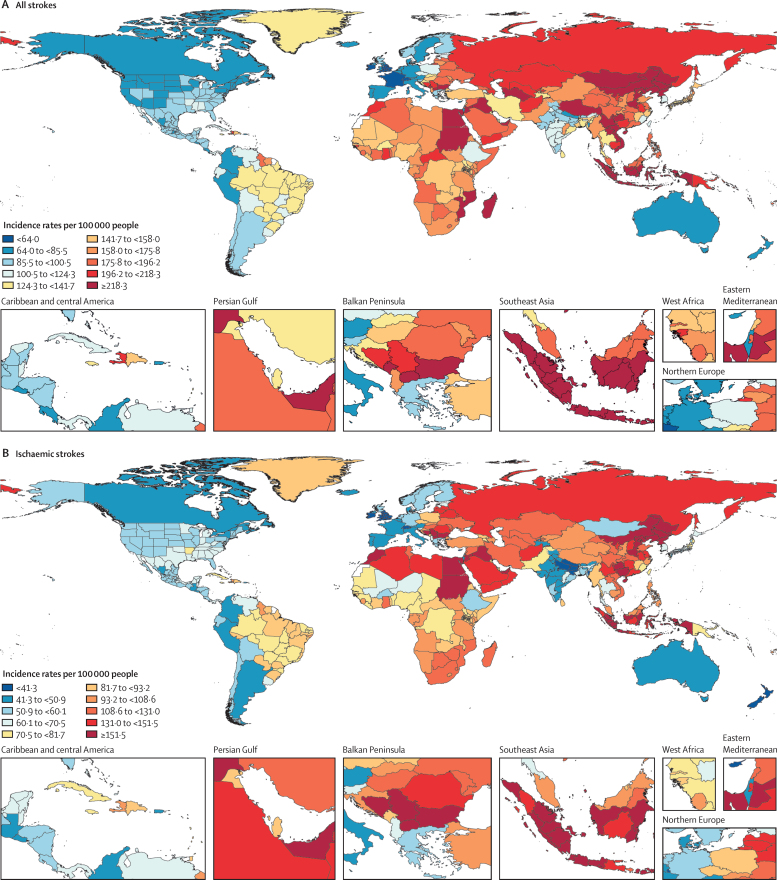

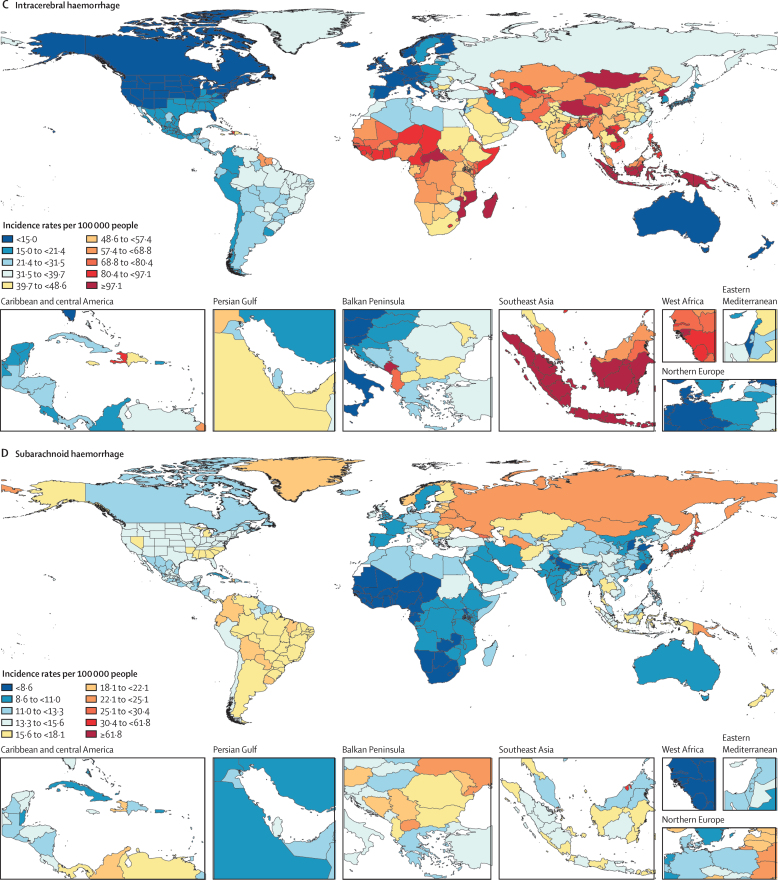
Age-standardised stroke incidence rates per 100 000 people by stroke type and country, for both sexes, 2019 (A) All strokes. (B) Ischaemic stroke. (C) Intracerebral haemorrhage. (D) Subarachnoid haemorrhage.

**Figure 2:**
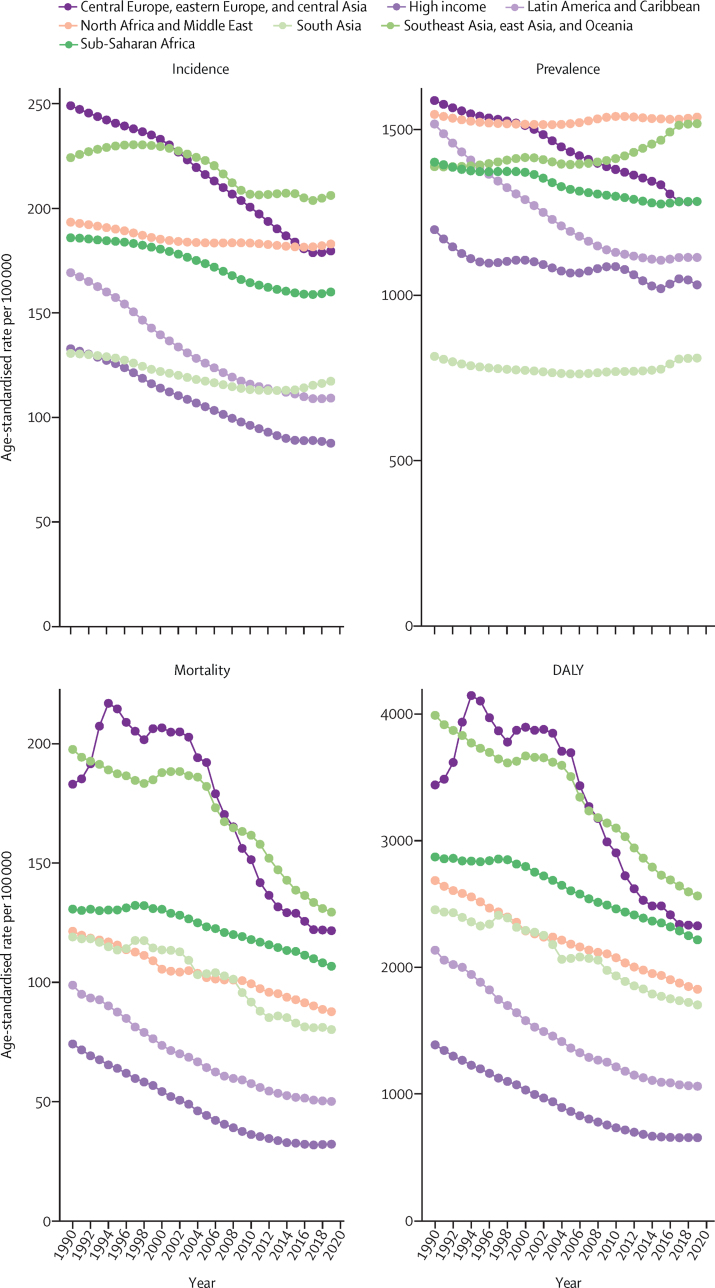
Age-standardised incidence, prevalence, mortality, and DALY rates (per 100 000 people per year) in seven GBD super regions, 1990–2019, for both sexes and all ages DALY=disability-adjusted life-year. GBD=Global Burden of Diseases, Injuries, and Risk Factors Study.

In 2019, there were 12·2 million (95% UI 11·0–13·6) incident strokes and 101 million (93·2–111) prevalent strokes, 143 million (133–153) DALYs due to stroke, and 6·55 million (6·00–7·02) deaths from stroke (table 1). Globally, stroke was the second-leading Level 3 cause of death (11·6% [10·8–12·2] of total deaths) after ischaemic heart disease (16·2% [15·0–16·9]). Stroke was also the third-leading Level 3 cause of death and disability combined in 2019 (5·7% [5·1–6·2] of total DALYs), after neonatal disorders (7·3% [64·4–8·4]) and ischaemic heart disease (7·2% [6·5–8·0]; appendix section 4.1 and figure S2). In 2019, the World Bank low-income group of countries had an age-standardised stroke-related mortality rate 3·6 (3·5–3·8) times higher and an age-standardised stroke-related DALY rate 3·7 (3·5–3·9) times higher than those of high-income countries (see appendix tables S1, S3, and S5 and figures S2–7 for more detailed results by country and World Bank income group). In 2019, 86·0% (85·9–86·9) of all stroke-related deaths and 89·0% (88·9–89·3) of stroke-related DALYs occurred in lower-income, lower-middle-income, and upper-middle-income countries (appendix table S1). There were substantial between-country variations (figure 1A) in age-standardised stroke incidence rates and regional variations (figure 2) in age-standardised incidence, prevalence, mortality, and DALY rates. The absolute number of incident strokes globally increased by 70·0% (67·0–73·0) from 1990 to 2019, whereas prevalent strokes increased by 85·0% (83·0–88·0), deaths from stroke increased by 43·0% (31·0–55·0), and DALYs due to stroke increased by 32·0% (22·0–42·0; table 1, appendix figure S3). Although absolute numbers increased over the study period, age-standardised rates all decreased between 1990 and 2019: by 17·0% (15·0–18·0) for incidence; by 6·0% (5·0–7·0) for prevalence; by 36·0% (31·0–42·0) for mortality; and by 36·0% (31·0–42·0) for DALYs (table 1). However, among those younger than 70 years, age-specific stroke prevalence and incidence rates increased substantially over the study period (22·0% [21·0–24·0] increase in prevalence and 15·0% [12·0–18·0] increase in incidence; incidence data are shown in appendix figure 7, prevalence data are available on the Global Health Data Exchange).

**Table 1:** Absolute number and age-standardised rates per year of incident and prevalent strokes, deaths from stroke and DALYs due to stroke in 2019, and percentage change globally for 1990–2019, by pathological types of stroke

	**Incidence (95% UI)**		**Deaths (95% UI)**		**Prevalence (95% UI)**		**DALYs (95% UI)**	
	2019	Percentage change, 1990–2019	2019	Percentage change, 1990–2019	2019	Percentage change, 1990–2019	2019	Percentage change, 1990–2019
**Ischaemic stroke**								
Absolute number, millions	7·63 (6·57 to 8·96)	88·0% (83·0 to 92·0)	3·29 (2·97 to 3·54)	61·0% (46·0 to 75·0)	77·19 (68·86 to 86·46)	95·0% (92·0 to 99·0)	63·48 (57·83 to 68·99)	57·0% (43·0 to 68·0)
Age-standardised rate, per 100 000 people	94·51 (81·9 to 110·76)	−10·0% (−12·0 to −8·0)	43·50 (39·08 to 46·77)	−34·0% (−39·0 to −28·0)	951·0 (849·2 to 1064·1)	−2·0% (−3·0 to 0·0)	798·8 (727·5 to 866·9)	−29·0% (−35·0 to −23·0)
**Intracerebral haemorrhage**								
Absolute number, millions	3·41 (2·97 to 3·91)	43·0% (41·0 to 45·0)	2·89 (2·64 to 3·10)	37·0% (22·0 to 51·0)	20·66 (18·02 to 23·42)	58·0% (56·0 to 60·0)	68·57 (63·27 to 73·68)	25·0% (12·0 to 36·0)
Age-standardised rate, per 100 000 people	41·81 (36·53 to 47·88)	−29·0% (−30·0 to −28·0)	36·04 (32·98 to 38·67)	−36·0% (−43·0 to −29·0)	248·8 (217·1 to 281·4)	−17·0% (−18·0 to −15·0)	823·8 (769·2 to 894·7)	−37·0% (−43·0 to −31·0)
**Subarachnoid haemorrhage**								
Absolute number, millions	1·18 (1·01 to 1·39)	61·0% (56·0 to 65·0)	0·37 (0·33 to 0·42)	−12·0% (−25·0 to 26·0)	8·40 (7·19 to 9·83)	65·0% (60·0 to 68·0)	11·18 (9·89 to 12·67)	−14% (−26·0 to 17·0)
Age-standardised rate, per 100 000 people	14·46 (12·33 to 16·94)	−17·0% (−19·0 to −15·0)	4·66 (4·13 to 5·17)	−57·0% (−64·0 to −39·0)	101·6 (87·1 to 118·5)	−37·0% (−43·0 to −31·0)	136·5 (120·8 to 154·7)	−54·0% (−61·0 to −37·0)
**Total stroke**								
Absolute number, millions	12·22 (11·04 to 13·59)	70·0% (67·0 to 73·0)	6·55 (6·00 to 7·02)	43·0% (31·0 to 55·0)	101·47 (93·21 to 110·53)	85·0% (83·0 to 88·0)	143·23 (133·10 to 153·24)	32·0% (22·0 to 42·0)
Age-standardised rate, per 100 000 people	150·8 (136·5 to 167·5)	−17·0% (−18·0 to −15·0)	84·2 (76·8 to 90·2)	−36·0% (−42·0 to −31·0)	1240·3 (1139·7 to 1353·0)	−6·0% (−7·0 to −5·0)	1768·1 (1640·7 to 1889·4)	−36·0% (−42·0 to −31·0)

Absolute numbers in millions and age-standardised rates per 100 000 people are presented to two decimal places and percentage change is shown to one decimal place. UI=uncertainty interval. DALY=disability-adjusted life-year.

Although the absolute number of DALYs due to stroke in males (76·9 million [95% UI 70·2–83·5]) exceeded that in females (66·4 million [60·5–72·3]) at the global level in 2019, the point estimates of incident and prevalent strokes were higher in females (6·44 million [5·81–7·17] incident strokes and 56·4 million [52·0–61·5] prevalent strokes) than in males (5·79 million [5·24–6·45] incident strokes and 45·0 million [41·1–49·3] prevalent strokes), and there were no noticeable sex differences in the number of stroke-related deaths (appendix table S2). Although age-standardised incidence rates did not differ significantly between males and females, age-standardised death rates were greater in males than in females (96·4 [87·6–104·2] per 100 000 *vs* 73·5 [65·2–80·7] per 100 000) as were DALY rates (2024·3 [1852·4–2195·6] per 100 000 *vs* 1531·3 [1397·1–1667·6] per 100 000; see appendix section 4.1 for details of age-specific trends by country).

### Burden of pathological types of stroke

Ischaemic stroke constituted 62·4% of all new strokes in 2019 (7·63 million [95% UI 6·57–8·96] strokes), intracerebral haemorrhage constituted 27·9% (3·41 million [2·97–3·91]), and subarachnoid haemorrhage constituted 9·7% (1·18 million [1·01–1·39]; table 1). Intracerebral haemorrhage and subarachnoid haemorrhage showed larger reductions in age-standardised rates from 1990 to 2019 than ischaemic stroke (table 1; appendix section 4·2, tables S3–5, and figure S8). There were substantial between-country variations in the age-standardised incidence (figures 1B–D), prevalence, mortality, and DALY rates (appendix figures S8–11) of these three pathological types of stroke by GBD regions, country income level, and sex (appendix section 4.2), with an almost two-fold greater proportion of intracerebral haemorrhage in World Bank low-income to upper-middle-income countries compared with high-income countries (29·5% [28·4–30·3] *vs* 15·8% [15·5–16·2]), but a lower proportion of subarachnoid haemorrhage in low-income to upper-middle-income countries compared with high-income countries (7·9% [7·5–8·3] *vs* 19·7% [18·4–21·0]).

### Stroke-related DALYs attributable to risk factors

GBD stroke estimates for 1990–2019 are available to download from the GBD Results Tool. In 2019, 87·0% (95% UI 84·2–89·8) of total stroke DALYs were attributable to the 19 risk factors modelled in GBD 2019. The PAF of DALYs attributable to all risk factors combined was similar for ischaemic stroke (85·7% [81·2–90·3]), intracerebral haemorrhage (88·7% [85·2–91·0]), and subarachnoid haemorrhage (84·6% [81·3–87·6]; appendix section 4.3 and tables S6–8). From 1990 to 2019, the total number of stroke-related DALYs due to risk factors increased from 91·5 million (85·8–98·3) to 125 million (115–134), with a decrease in the high-income group (from 16·4 million [15·4–17·4] in 1990 to 13·1 million [11·8–14·4] in 2019) and an increase in the low-income to upper-middle-income groups (from 75·1 million [68·5–82·3] DALYs in all three income groups combined in 1990 to 111 million [100·3–122·5] DALYs in 2019). From 1990 to 2019, the largest increase in the age-standardised stroke PAF globally was for high BMI, increasing from 15·4% (8·2–24·2) to 24·3% (15·7–33·2), a 57·8% increase. In other words, if high BMI exposure were reduced to its TMREL, there would be a 24·3% reduction in stroke in 2019, compared to just a 15·4% reduction in 1990. Other risk factors with an increasing age-standardised stroke PAF from 1990 to 2019 included high systolic blood pressure (from 52·0% [44·6–58·6] to 55·5% [48·2–62·0], a 6·7% increase) and high fasting plasma glucose (from 14·4% [9·9–20·8] to 20·2% [13·8–29·1], a 40·3% increase). By contrast, from 1990 to 2019, the stroke PAF of ambient particulate matter with a diameter of <2.5 μm (known as PM_2·5_) pollution decreased from 32·5% (29·6–35·6) to 20·1% (16·6–23·0; a 38·2% decrease), and that of dietary risks decreased from 32·6% (24·7–41·5) to 30·6% (22·6–39·8; a 6·1% decrease).

**Figure 3:**
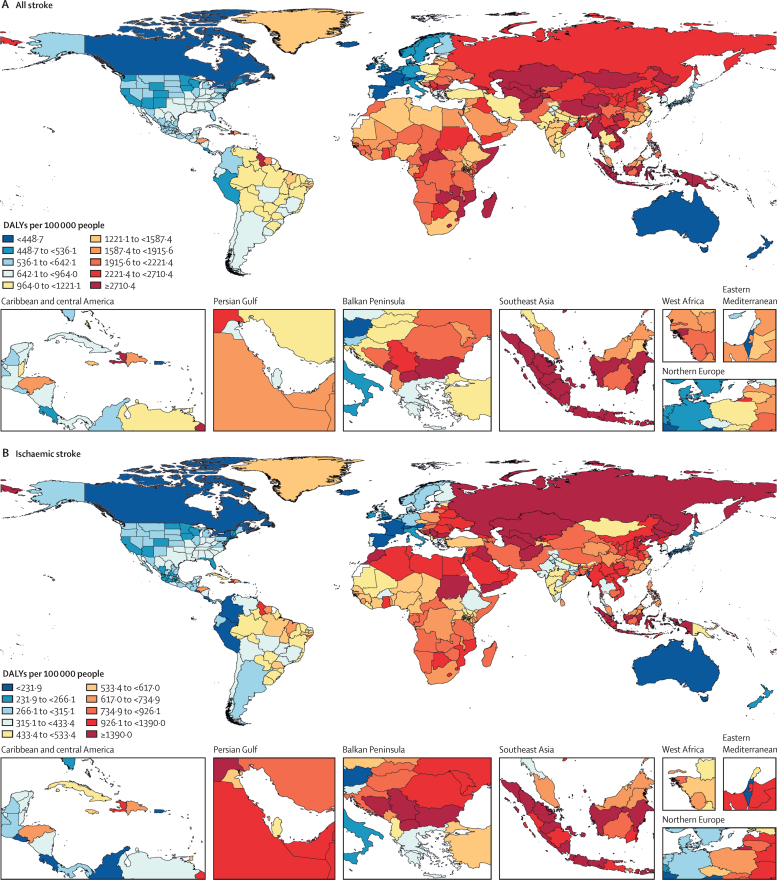

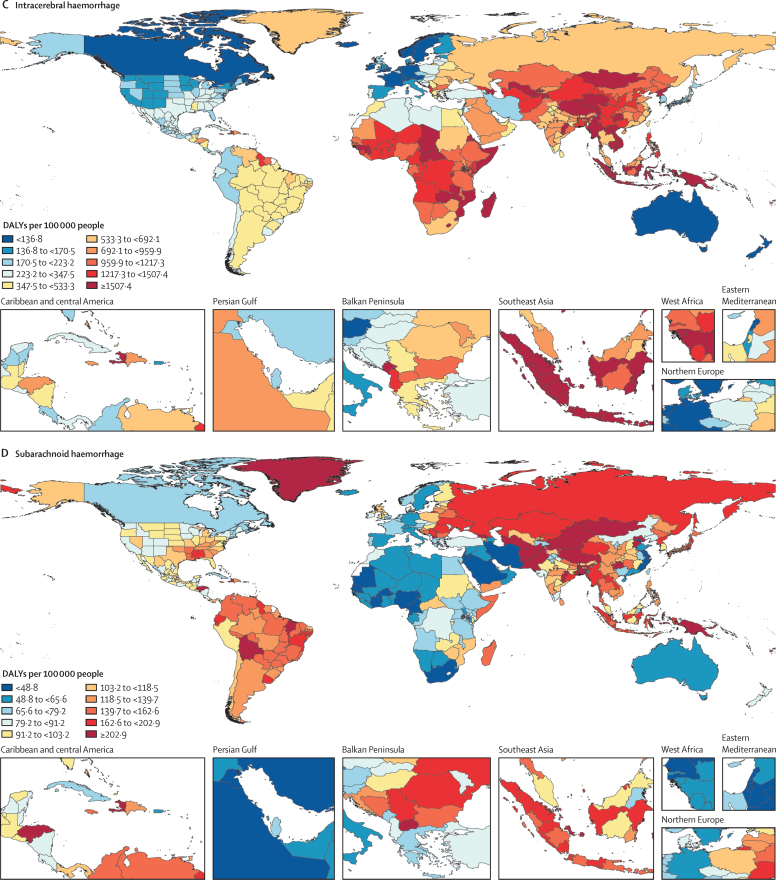
Age-standardised stroke-related DALYs attributable to all risk factors combined, for both sexes, 2019 (A) All strokes. (B) Ischaemic stroke. (C) Intracerebral haemorrhage. (D) Subarachnoid haemorrhage. DALY=disability-adjusted life-year.

**Figure 4:**
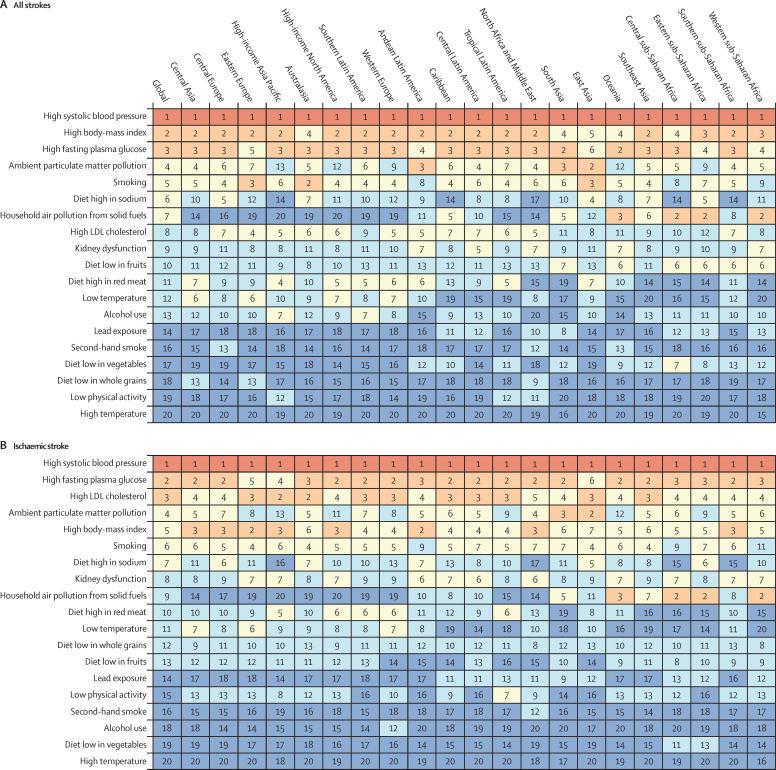

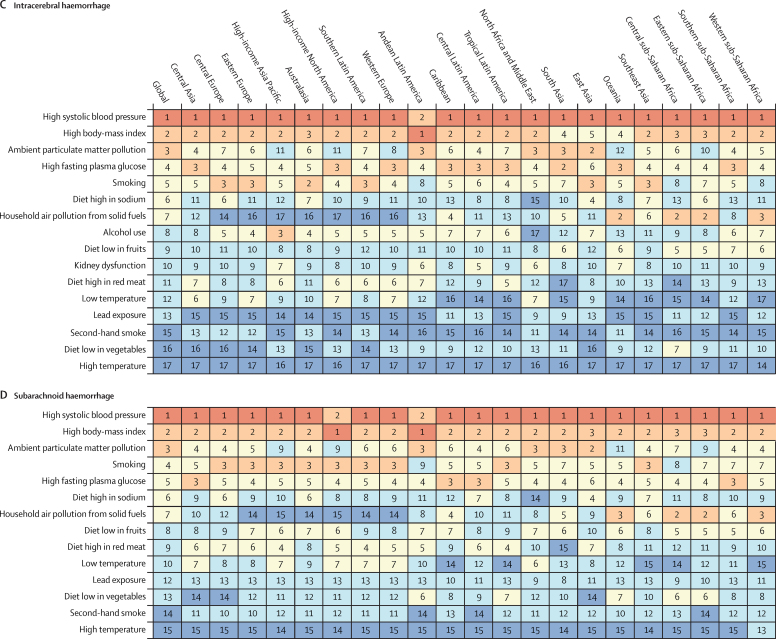
Age-standardised stroke-related DALYs attributable to risk factors by 21 GBD regions, for both sexes, 2019 (A) All strokes. (B) Ischaemic stroke. (C) Intracerebral haemorrhage. (D) Subarachnoid haemorrhage. Numbers show the ranking level (1=highest, 15=lowest) by the number of DALYs attributable to the corresponding risk factors. Red shows 1st ranking; light brown, 2nd and 3rd ranking; very light yellow, 4–7 ranking; very light blue, 8–13 ranking; and dark blue, 14–15 ranking. Diet low in whole grains, low physical activity, and high LDL cholesterol were not assessed for intracerebral haemorrhage. Diet low in whole grains, alcohol use, low physical activity, high LDL cholesterol, and kidney dysfunction were not assessed for subarachnoid haemorrhage. DALY=disability-adjusted life-year. GBD=Global Burden of Diseases, Injuries, and Risk Factors Study.

**Figure 5:**
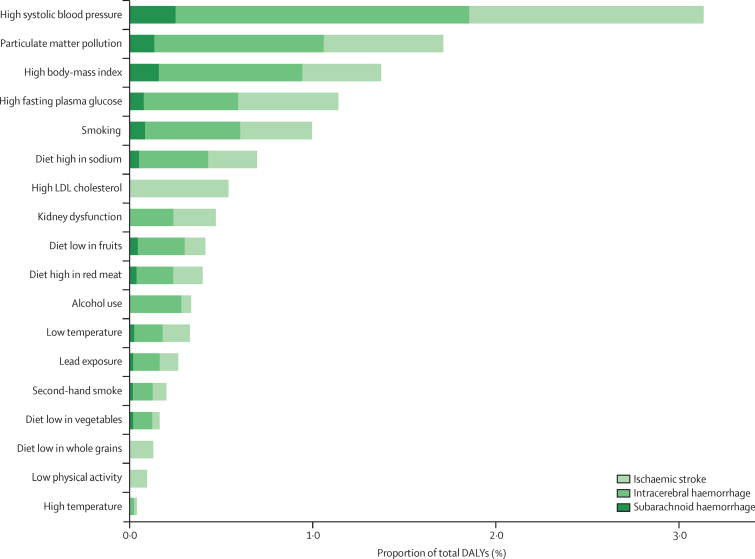
Proportion of DALYs attributable to risk factors by pathological type of stroke for both sexes combined, 2019 Proportion of DALYs attributable to household air pollution from solid fuels are not shown in this figure. DALY=disability-adjusted life-year.

In 2019, there were moderate between-country (1·3 times), regional (as measured by 21 GBD regions), and country economic development level (as measured by the World Bank income groups) variations in the proportion of stroke-related DALYs and its DALYs related to stroke pathological types that were attributable to risk factors. Between-country variations were more pronounced for subarachnoid haemorrhage (figure 3; appendix tables S6–12 and figures S12–14), and the highest proportion of stroke-related DALYs was observed in the World Bank low-income to upper-middle-income groups (ranging from 85·9% [95% UI 83·2–88·6] in the World bank low-income group to 87·3% [84·4–89·9] in the World Bank upper-middle-income group). From 1990 to 2019, there was an increase in the total number of stroke-related DALYs due to high BMI, high fasting plasma glucose, high LDL cholesterol, kidney dysfunction, a diet high in red meat, alcohol consumption, and second-hand smoking, but a decrease in DALYs due to smoking and a diet low in fruits and vegetables (appendix figure S15). There were also moderate variations in the ranking of risk factors by pathological types of stroke (figure 4; appendix figures S16–18). In 2019, the five leading specific risk factors contributing to stroke death and disability combined (DALYs) were high systolic blood pressure (79·6 million [67·7–90·8] attributable DALYs; 55·5% [48·2–62·0] of all stroke DALYs]), high BMI (34·9 million [22·3–48·6]; [24·3% [15·7–33·2]), high fasting plasma glucose (28·9 million [19·8–41·5]; 20·2% [13·8–29·1]), ambient particulate matter pollution (28·7 million [23·4–33·4]; 20·1% [16·6–23·0]), and smoking (25·3 million [22·6–28·2]; 17·6% [16·4–19·0]; table 2, figure 5). For risk factors by pathological type of stroke and changes in risk factor rankings from 1990 to 2019 by GBD regions, see the appendix (section 4.3 and figures S19–26).

**Table 2:** Stroke-related DALYs (absolute numbers and percentages) associated with risk factors and their clusters in 2019, for all ages and both sexes

	**Globally**		**World Bank high-income countries**		**World Bank upper-middle-income countries**		**World Bank lower-middle-income countries**		**World Bank low-income countries**	
	Absolute number (millions)	Percentage	Absolute number (millions)	Percentage	Absolute number (millions)	Percentage	Absolute number (millions)	Percentage	Absolute number (millions)	Percentage
**Environmental risks**										
Ambient PM_2·5_pollution	28·70 (23·40–33·40)	20·1% (16·6–23·0)	1·57 (1·20–1·95)	9·9% (7·8–12·3)	16·10 (13·20–18·90)	23·9% (20·5–26·7)	10·30 (8·04–12·60)	20·0% (15·7–24·1)	0·75 (0·42–1·16)	8·7% (5·0–13·4)
Household air pollution from solid fuels	14·70 (10·10–20·10)	10·3% (7·1–14·0)	0·03 (0·01–0·07)	0·2% (0·1–0·5)	3·64 (1·86–6·14)	5·4% (2·8–9·0)	8·09 (5·59–10·90)	15·7% (11·0–21·0)	2·96 (2·29–3·69)	34·4% (28·5–40·2)
Low ambient temperature	8·36 (6·19–10·80)	5·8% (4·4–7·5)	1·28 (0·96–1·64)	8·2% (6·1–10·5)	5·47 (4·07–7·29)	8·1% (6·2–10·7)	1·26 (0·45–2·03)	2·4% (0·9–3·9)	0·35 (0·22–0·50)	4·0% (2·6–5·6)
High ambient temperature	1·09 (0·11–2·38)	0·8% (0·1–1·6)	0·03 (0·01–0·06)	0·2% (0·1–0·4)	0·14 (0·00–0·39)	0·2% (0·0–0·6)	0·82 (0·06–1·72)	1·6% (0·1–3·4)	0·10 (0·02–0·28)	1·2% (0·3–3·1)
Lead exposure	6·74 (3·91–9·82)	4·7% (2·8–6·8)	0·25 (0·06–0·50)	1·6% (0·4–3·2)	3·13 (1·80–4·60)	4·7% (2·8–6·7)	2·94 (1·78–4·17)	5·7% (3·5–8·0)	0·42 (0·23–0·64)	4·9% (2·7–7·2)
**Dietary risks**										
Diet high in sodium	17·70 (5·75–34·90)	12·3% (4·1–24·3)	1·03 (0·15–2·59)	6·5% (0·9–16·4)	11·60 (4·77–20·20)	17·3% (7·3–29·6)	4·33 (0·56–10·50)	8·4% (1·1–20·3)	0·69 (0·09–1·88)	8·0% (1·0–21·6)
Diet high in red meat	10·10 (6·37–13·50)	7·1% (4·5–9·3)	1·45 (0·98–1·85)	9·2% (6·2–11·5)	6·73 (4·46–8·84)	10·0% (6·7–12·8)	1·63 (0·75–2·44)	3·2% (1·5–4·7)	0·29 (0·10–0·47)	3·4% (1·1–5·3)
Diet low in fruits	10·50 (6·24–16·00)	7·3% (4·4–11·2)	0·81 (0·41–1·30)	5·1% (2·6–8·2)	3·70 (1·92–5·97)	5·5% (2·9–8·8)	5·17 (3·23–7·74)	10·0% (6·2–14·9)	0·81 (0·47–1·27)	9·4% (5·6–14·4)
Diet low in vegetables	4·15 (1·54–6·84)	2·9% (1·1–4·8)	0·30 (0·10–0·54)	1·9% (0·6–3·4)	0·73 (0·31–1·19)	1·1% (0·5–1·8)	2·52 (0·80–4·30)	4·9% (1·5–8·2)	0·60 (0·24–0·95)	7·0% (2·8–11·1)
Diet low in whole grains	3·26 (0·98–4·76)	2·3% (0·7–3·3)	0·42 (0·12–0·62)	2·7% (0·8–3·9)	1·73 (0·48–2·57)	2·6% (0·7–3·7)	0·96 (0·31–1·43)	1·9% (0·6–2·8)	0·13 (0·04–0·20)	1·6% (0·5–2·3)
Alcohol consumption	8·54 (6·02–11·10)	6·0% (4·3–7·6)	1·00 (0·67–1·34)	6·3% (4·2–8·4)	4·99 (3·48–6·64)	7·4% (5·3–9·5)	2·13 (1·46–2·82)	4·1% (2·8–5·5)	0·42 (0·25–0·60)	4·9% (3·1–6·7)
**Physical activity**										
Low physical activity	2·41 (0·43–6·38)	1·7% (0·3–4·5)	0·46 (0·07–1·26)	2·9% (0·5–8·0)	1·23 (0·23–3·29)	1·8% (0·4–4·9)	0·65 (0·12–1·78)	1·3% (0·2–3·4)	0·07 (0·01–0·20)	0·8% (0·1–2·3)
**Tobacco smoking**										
Smoking	25·30 (22·60–28·20)	17·6% (16·4–19·0)	2·68 (2·44–2·94)	17·0% (15·8–18·3)	13·90 (11·90–16·10)	20·7% (19·0–22·4)	7·81 (6·94–8·74)	15·1% (13·9–16·4)	0·88 (0·72–1·06)	10·2% (9·1–11·3)
Second-hand smoking	5·09 (3·79–6·56)	3·5% (2·7–4·5)	0·31 (0·24–0·39)	2·0% (1·5–2·5)	2·62 (1·93–3·37)	3·9% (2·9–4·9)	1·93 (1·41–2·56)	3·7% (2·8–4·8)	0·22 (0·15–0·30)	2·6% (1·9–3·4)
**Physiological factors**										
High body-mass index	34·90 (22·30–48·60)	24·3% (15·7–33·2)	3·99 (2·73–5·36)	25·4% (17·2–34·2)	15·70 (9·39–22·80)	23·4% (14·1–33·0)	13·30 (8·65–18·30)	25·8% (17·0–34·7)	1·87 (1·04–2·84)	21·8% (12·9–31·6)
High fasting plasma glucose	28·90 (19·80–41·50)	20·2% (13·8–29·1)	3·88 (2·45–6·35)	24·7% (15·7–40·4)	12·30 (8·28–18·30)	18·3% (12·4–26·5)	11·30 (7·73–15·90)	21·9% (15·2–30·6)	1·37 (0·92–1·96)	15·9% (11·1–22·7)
High systolic blood pressure	79·60 (67·70–90·80)	55·5% (48·2–62·0)	7·71 (6·44–9·07)	48·9% (41·3–56·5)	37·20 (31·10–43·40)	55·4% (47·2–62·6)	30·00 (25·50–34·00)	58·1% (50·4–64·4)	4·57 (3·60–5·56)	53·2% (45·6–59·6)
High LDL cholesterol	13·70 (7·72–23·40)	9·6% (5·5–16·4)	2·02 (0·87–3·91)	12·8% (5·5–24·3)	7·34 (4·07–12·50)	10·9% (6·2–18·9)	3·84 (2·37–6·23)	7·4% (4·6–12·1)	0·50 (0·31–0·77)	5·8% (3·7–9·0)
Kidney dysfunction	11·90 (9·75–14·10)	8·3% (7·0–9·7)	1·07 (0·73–1·38)	6·8% (4·7–8·7)	5·62 (4·48–6·65)	8·4% (7·0–9·7)	4·70 (3·88–5·55)	9·1% (7·6–10·6)	0·56 (0·44–0·69)	6·5% (5·5–7·7)
**Cluster of risk factors**										
Air pollution*	43·50 (38·40–48·70)	30·4% (27·7–33·1)	1·60 (1·23–2·00)	10·2% (8·0–12·6)	19·70 (16·70–22·80)	29·3% (26·5–32·2)	18·40 (16·20–20·70)	35·7% (32·7–38·8)	3·71 (3·08–4·38)	43·1% (40·1–46·3)
Tobacco smoke†	29·50 (26·30–32·70)	20·6% (19·2–22·0)	2·92 (2·65–3·20)	18·5% (17·3–19·8)	16·00 (13·80–18·50)	23·8% (22·1–25·6)	9·49 (8·44–10·60)	18·4% (16·8–19·8)	1·07 (0·89–1·30)	12·5% (11·2–13·8)
Dietary risks‡	43·80 (32·10–58·10)	30·6% (22·6–39·8)	4·01 (2·98–5·39)	25·5% (18·9–33·6)	22·40 (15·90–29·70)	33·3% (24·4–42·9)	15·00 (10·60–20·00)	29·0% (20·7–38·5)	2·42 (1·61–3·46)	28·2% (19·6–38·9)
Behavioural risks§	67·90 (58·20–79·30)	47·4% (41·3–54·4)	6·88 (5·90–7·99)	43·7% (38·0–49·8)	34·90 (29·10–41·20)	51·9% (45·3–58·6)	22·70 (19·00–27·00)	44·0% (37·7–51·5)	3·42 (2·59–4·43)	39·8% (32·8–48·8)
Environmental or occupational risks¶	54·20 (48·20–60·00)	37·8% (35·0–41·0)	2·98 (2·48–3·53)	18·9% (16·0–22·4)	25·60 (22·00–29·10)	38·1% (34·9–41·4)	21·40 (18·90–24·10)	41·5% (38·4–44·9)	4·17 (3·48–4·87)	48·6% (45·3–51·8)
Metabolic risks‖	102·00 (89·80–112·00)	71·0% (64·6–77·1)	10·90 (9·36–12·50)	69·1% (61·1–77·0)	47·50 (40·70–53·70)	70·7% (64·0–77·0)	37·60 (33·00–41·50)	72·8% (66·6–78·1)	5·63 (4·57–6·70)	65·5% (58·6–71·2)
**Combined risk factors**										
All factors	125·00 (115·00–134·00)	87·0% (84·2–89·8)	13·10 (11·80–14·40)	83·2% (78·6–88·2)	59·10 (53·00–65·10)	87·9% (84·9–90·7)	45·20 (41·30–49·00)	87·6% (85·2–89·9)	7·18 (6·08–8·41)	83·7% (81·0–86·1)

Data in parentheses are 95% uncertainty intervals. Count data in millions are presented to two decimal places and percentage data are presented to one decimal place. Percentages and number of DALYs are not mutually exclusive: the sum of percentages and number of DALYs in the columns exceeds the totals for all risk factors combined because of overlap between various risk factors. The crude sum of population attributable fraction (PAF) of the risk factors might exceed 100% because the effects of many of these risk factors are mediated partly or wholly through another risk factor or risk factors. DALY=disability-adjusted life-year. PM_2·5_=particulate matter with a diameter of <25 μm.

* Air pollution cluster includes ambient PM_2·5_ pollution and household air pollution from solid fuels.

† Tobacco smoke cluster includes smoking and second-hand smoking.

‡ Dietary risks cluster includes diet high in sodium, diet low in fruits, diet low in vegetables, diet high in red meat, and diet low in whole grains, and alcohol consumption.

§ Behavioural risks cluster includes smoking (including second-hand smoking), dietary risks (diet high in sodium, diet low in fruits, diet low in vegetables, diet high in red meat, diet low in whole grains, and alcohol consumption), and low physical activity.

¶ Environmental risks cluster includes air pollution cluster, low ambient temperature, high ambient temperature, and lead exposure.

‖ Metabolic risks cluster includes high body-mass index, high fasting plasma glucose, high LDL cholesterol, high systolic blood pressure, and kidney dysfunction.

## Discussion

In 2019, stroke remained the second-leading Level 3 cause of death and the third-leading Level 3 cause of death and disability combined in the world, and its burden (in terms of the absolute number of cases) increased substantially from 1990 to 2019. Our findings indicate that the bulk of the global stroke burden (86·0% [95% UI 85·9–86·9] of deaths and 89·0% [88·9–89·3] of DALYs) is in lower-income and lower-middle-income countries. Globally, over the past three decades, the total number of stroke-related DALYs due to risk factors increased substantially (by 33·5 million, from 91·5 million in 1990 to 125 million in 2019), with diverging trends in World Bank high-income countries and low-income to upper-middle-income countries: a relatively small decrease in the high-income group and large increases in the low-income to upper-middle income groups. The large increase in the global burden of stroke was probably not only due to population growth and ageing but also because of the substantial increase in exposure to several important risk factors such as high BMI, ambient particulate matter pollution, high fasting plasma glucose, high systolic blood pressure, alcohol consumption, low physical activity, kidney dysfunction, and high temperature (appendix figure S26).^7^, ^14^ This study is also the first systematic analysis to determine the effect of non-optimal temperature on stroke burden. The greater age-standardised burden of stroke in World Bank low-income to upper-middle-income countries than in the high-income countries might also relate to poorer acute health care for stroke,^15^ poorer stroke awareness,^16^ and greater prevalence or effect of some risk factors (eg, tobacco use, poor diet, diabetes, hypertension, cardiovascular disease, rheumatic heart disease, dyslipidaemia, and obesity) in low-income countries than in upper-middle-income countries,^17^, ^18^ which highlights the inadequacy of primary prevention efforts in these settings.

For the first time, we have presented the global, regional, and national burden of stroke and its risk factors by its major pathological types. Although ischaemic stroke continues to constitute the largest proportion of all new strokes (comprising 62·4% of all incident strokes in 2019), followed by intracerebral haemorrhage (27·9%), and subarachnoid haemorrhage (9·7%), the relative proportions of each pathological type varied substantially by income group. For example, a new stroke case was nearly twice as likely to be intracerebral haemorrhage in the World Bank low-income to upper-middle-income groups combined than in the high-income group (29·5% of all incident strokes in 2019 *vs* 15·8%), whereas a new stroke case was more than twice as likely to be subarachnoid haemorrhage in the World Bank high-income group than in the low-income to upper-middle-income groups combined (19·7% *vs* 7·9%). The increased risk of intracerebral haemorrhage in low-income and upper-middle-income countries might be related to the high relative clinical significance and population-attributable risk of hypertension in these countries.^18^ Our finding that a greater proportion of incident strokes in low-income to upper-middle-income countries are intracerebral haemorrhages in males than in females (appendix figure F6.9) are in line with previous observations,^19^, ^20^ and might be explained by lower levels of awareness and control of hypertension in low-income to upper-middle-income countries than in high-income countries,^18^, ^21^ as well as increased exposure to risk factors predisposing a higher proportion of males to intracerebral haemorrhage compared with females.^20^, ^22^ Our study also adds to the body of research on the incidence of subarachnoid haemorrhage; a previous systematic review of population-based studies of subarachnoid haemorrhage incidence by Etminan and colleagues^23^ had similar findings to ours, as the authors used many of the same sources, but they only used crude incidence rates, which is perhaps why we found smaller between-country variations in the age-standardised incidence of subarachnoid haemorrhage (approximately a tenfold variation in our findings [appendix table s5] compared with a >20-fold variation in the systematic review).^23^ The size of between-country variations we observed in age-standardised incidence, prevalence, and DALY rates of other pathological types of stroke and stroke overall were in line with previous observations.^2^, ^3^, ^19^, ^20^, ^24^

Despite the overall declines in age-standardised stroke incidence, prevalence, death, and DALY rates, three concerning trends have emerged. First, the greatest share of the global burden of stroke continues to be borne by low-income to upper-middle-income countries. The proportion of DALYs attributable to GBD-modelled risk factors was also particularly high in low-income to upper-middle-income countries. Second, the pace of the global decline in age-standardised stroke incidence, death, and DALY rates was noticeably slower over the past decade (2010–19) than in the previous decade (2000–09), and global age-standardised prevalence significantly increased from 2010 to 2019 (appendix figure 6.8). There was a significant increase in stroke prevalence and incidence rates in people younger than 70 years between 1990 and 2019 with even faster increases from 2010 to 2019 (appendix figure 6.7). A trend towards plateauing or increasing stroke incidence or mortality rates, or both, in middle-aged people was recently observed in the USA, European countries, Brazil, and China.^25^, ^26^, ^27^, ^28^, ^29^, ^30^ This trend might be a reflection of the increased exposure to some risk factors for stroke, such as elevated blood pressure, high BMI, and high fasting plasma glucose, across most countries.^31^, ^32^, ^33^ In the USA, a worrisome trend observed in recent years (2017–18) is that awareness of hypertension in the population whose blood pressure is controlled is declining.^34^ Third, most countries have not achieved sufficient declines in stroke incidence rates to offset the demographic force of population growth and ageing, resulting in overall increases in the number of incident, prevalent, fatal, and disabling strokes over time. A linear interpolation shows that if current trends continue, by 2050 there will be more than 200 million stroke survivors and almost 300 million DALYs, 25 million new strokes, and 13 million deaths from stroke annually.

This study was, to our knowledge, the first systematic analysis to provide estimates of the burden of stroke and its subtypes associated with non-optimal temperature (daily temperatures below or above the TMREL). Although previous studies have made ecological observations of the effects of ambient temperature on the risk of stroke, this study was the first to show the sizeable global effect of non-optimal temperature (primarily low temperature, at 8·36 million [95% UI 6·19–10·80] DALYs or a PAF of 5·8% [4·4–7·5]) on the burden of stroke and its pathological types (appendix tables T10b, T11b, and T12b). These findings were in line with a recent systematic review on ambient temperature and stroke occurrence.^35^ Our estimates of geographical variations in the burden of stroke and its pathological types associated with non-optimal temperature and other risk factors suggest that country-specific and stroke type-specific priorities and strategies should be developed and implemented for reducing the burden of stroke in different geographical locations.

Our findings of the high proportion (87·0%) of age-standardised stroke-related DALYs associated with GBD risk factors are in line with previous observations^17^, ^36^ and highlight the potential to greatly reduce the stroke burden by addressing risk factor exposure. The increased contribution of certain metabolic risk factors in 2019 compared with 1990 (eg, an increase in the proportional contribution to stroke-related DALYs of 57·8% by high BMI and 40·3% by high fasting plasma glucose) and a decreasing contribution of certain environmental and occupational and behavioural risk factors to the stroke-related DALY burden over the same period (eg, a 38·2% decrease for household air pollution from solid fuels and a 6·1% decrease for a diet low in vegetables) might be related to a growing proportion of the global population reaching the final stages of the epidemiological transition, in which the risk burden has shifted towards metabolic risk factors and an increased proportion of the disease burden comes from stroke and other non-communicable diseases.^37^ This observation also means that guidance on reducing the risk of stroke by targeting certain risk factors will need to change to reflect changes in the risk-attributable profile.

Our estimates of the global, regional, and national burden of stroke and its pathological types and risk factors are important for evidence-based health-care planning, priority setting, and resource allocation for stroke care, primary prevention, and research. The high and increasing stroke burden alongside stagnant or even increasing mortality rates from cardiovascular disease in some countries,^14^ and increasing rates of exposure to many important stroke risk factors from 1990 to 2019,^7^, ^14^ suggest that current primary stroke prevention strategies and measures are not sufficient, and that efforts to implement population-wide primary prevention strategies more widely must be reinforced worldwide.^38^ For every US$1 spent on prevention of stroke and cardiovascular disease, there is an estimated $10·9 return on investment.^39^ Population-wide interventions for primary prevention of stroke and cardiovascular disease should include measures to reduce exposure to metabolic risk factors (eg, screening for and proper management of systolic blood pressure and weight), behavioural risk factors (eg, smoking cessation programmes and programmes to increase the accessibility and affordability of nutrient-rich foods), and environmental and occupational risk factors (eg, measures to reduce air pollution and lead exposure). The development and implementation of such population-level interventions, alongside efforts to reduce poverty and racial and socioeconomic inequities, through legislation, taxation, and other measures at the government level, must be the mainstream approach for reducing the risk of stroke, cardiovascular disease, and other non-communicable diseases, but the importance of primary prevention measures at the individual level should not be overlooked. In this respect, the emphasis should be on strategies that are appropriate for most people at risk of stroke and cardiovascular disease regardless of their level of risk exposure,^38^ such as digital health technologies for affordable identification of people at increased risk of stroke and cardiovascular disease, universal health coverage, cheap and effective multidrug regimens (eg, polypills) for people at increased risk of cardiovascular disease, and involvement of health-care volunteers in primary prevention activities. For example, the World Stroke Organization recommends that all adults know their individual risk of having a stroke, their personal risk factors for stroke, and how to control these risk factors using the validated, internationally endorsed, and free Stroke Riskometer app, which is currently available in 19 languages for more than 70% of the global population.^40^ A recent Cochrane systematic review showed the feasibility and potential effectiveness of several health promotion interventions targeting risk factors to achieve behavioural changes for primary prevention of cardiovascular disease in low-income to upper-middle-income countries.^41^ Although knowledge of personal risk and management of behavioural risk factor activities is primarily the prerogative of individuals, health professionals have a responsibility to identify risk factors that require pharmacological and non-pharmacological treatment to reduce the chance of stroke occurrence (eg, elevated blood pressure, atrial fibrillation, diabetes, dyslipidaemia, or symptomatic carotid artery stenosis). Simple, inexpensive screening for cardiovascular disease risks (eg, elevated blood pressure, smoking, and overweight) by health professionals in low-income and middle-income settings or more accurate screening for high cardiovascular disease risks (including blood lipid tests) by health professionals in higher-income locations can help to identify people who might require prophylactic drug therapy, in conjunction with behavioural interventions.^40^ However, health professionals often do not have enough time to conduct detailed assessments of behavioural risk factors or to develop individually tailored recommendations for primary prevention of stroke and cardiovascular disease. To ameliorate this problem, data on stroke risk and risk factors from individuals should be integrated with the electronic patient management systems of health service providers. A study in Finland suggests that the quality of stroke prevention by primary health-care professionals could be improved by developing digital clinical decision-making tools and by implementing inter-professional teamwork^42^ (eg, the PreventS web app currently being developed in New Zealand). All of these measures should be facilitated by ongoing, culturally appropriate health education campaigns (including coordinated activities of non-governmental organisations) and inclusion of such health education information into standardised educational curricula at all levels.

In addition to primary stroke prevention efforts, appropriate secondary prevention efforts and adequate acute treatment and rehabilitation are essential to improve stroke outcomes. Our findings of large geographical variations in stroke prevalence, mortality, and disability are a reflection not only of geographical differences in stroke incidence but also of major inequities in acute stroke care and rehabilitation across countries.^43^ Even in European countries, only 7·3% of all patients with acute ischaemic stroke receive intravenous thrombolysis and only 1·9% receive endovascular treatment, with the highest country-level rates being 20·6% for intravenous thrombolysis (in the Netherlands) and 5·6% for endovascular treatment (in Malta),^44^ and one in three patients discontinues using one or more secondary stroke prevention drugs about 1 year after stroke.^45^ Treatment rates are even lower in many low-income and middle-income countries.^21^, ^43^ To reduce inequalities in stroke care, a roadmap for delivering quality stroke care and various action plans^46^, ^47^ have been suggested, with emphasis on the importance of applying culturally appropriate and context-appropriate strategies. There is a pressing need to implement evidence-based guidelines for stroke management and to reduce the gap in stroke care between high-income countries and low-income and middle-income countries. Recent evidence suggests that delivering an adequate level of stroke care^48^, ^49^ and preventive interventions^49^ in low-income and middle-income countries are feasible. Attention should be paid to developing the workforce for stroke care and setting up affordable and accessible rehabilitation facilities. Promising results^50^ suggest that self-management could be used as an adjunct strategy for ongoing rehabilitation at home or in other settings. The importance of country-based ongoing stroke registries and stroke risk factors surveys, which are profoundly lacking in low-income and middle-income countries, should also be emphasised.

Although this study was, to our knowledge, the first and most comprehensive review of the global, regional, and national burden of stroke and its 19 specific risk factors by all three pathological types, it was not free from limitations common to all previous GBD estimates of stroke risk and risk factors,^2^, ^3^, ^11^, ^36^ particularly the absence of original, good-quality stroke epidemiological studies for most countries. We therefore were not able to include some important potential risk factors (eg, atrial fibrillation and substance abuse), or include different patterns in risk factor exposure (eg, different doses and types of alcohol consumption, pack-years of smoking) and doses of exposure, analyse stroke burden by ischaemic stroke subtypes, or do a decomposition analysis to attribute changes in stroke burden to changes in the population growth, ageing, and risk factors separately. Additionally, evidence for the selection of TMRELs for some risk factors was uncertain and based on non-experimental studies, although all TMRELs were discussed and approved by a team of risk epidemiologists and stroke experts. Despite these limitations, our results are broadly consistent with previous estimates from population-based and analytical epidemiological studies, thus supporting the validity of our results.

In summary, although strokes are largely preventable, as indicated by declining incidence rates globally, stroke remained the second-leading cause of death and third-leading cause of death and disability combined worldwide in 2019. Without wider implementation of population-wide primary stroke and cardiovascular disease prevention strategies, the burden of stroke is likely to continue growing, disproportionally affecting low-income and middle-income countries. As the 19 analysed risk factors for stroke are common for other major non-communicable diseases, appropriate control of these risk factors will also reduce the burden of coronary heart disease, vascular dementia, type 2 diabetes, and even some types of cancer. Further research on the frequency, outcomes, and determinants of stroke and its pathological types in different locations and over time is warranted. Such research could include identifying populations at highest risk as well as further investigating differences in stroke pathological types and their geographical patterns, all of which would be useful for more targeted prevention and treatment efforts. Closing the gaps between high-income countries and low-income and middle-income countries in the adaptation and implementation of internationally recognised guidelines and recommendations for reducing stroke morbidity and mortality, with an emphasis on primary prevention strategies, is crucial to addressing the global stroke burden.

## Data sharing

All data presented in the manuscript can be found on the Institute for Health Metrics and Evaluation GBD Compare and Viz Hub website at https://vizhub.healthdata.org/gbd-compare/#.

## Declaration of interests

V Feigin reports support for the present manuscript from PreventS web app and free Stroke Riskometer app, which are owned and copyrighted by Auckland University of Technology, New Zealand. V Feigin reports grants received from the Brain Research New Zealand Centre of Research Excellence (16/STH/36), National Health & Medical Research Council (NHMRC, Australia APP1182071) and World Stroke Organization to their institution; leadership or fiduciary role in board, society, committee or advocacy group, paid or unpaid with World Stroke Organization as Executive Committee member, New Zealand Stroke Education (charitable) Trust as CEO, Stroke Central New Zealand as Honorary Medical Director, all of which are honorary unpaid roles; all outside the submitted work. O Adebayo reports grants or contracts from Merck Foundation; support for attending meetings and/or travel from Novartis; all outside the submitted work. R Akinyemi reports grants or contracts from NIH (U01HG010273), and GCRF (GCRFNGR6\1498), all outside the submitted work. R Ancuceanu consulting fees from AbbVie and AstraZeneca; payment or honoraria for lectures, presentations, speakers bureaus, manuscript writing or educational events from AbbVie, Sandoz, and B. Braun; support for attending meetings and/or travel from AbbVie and AstraZeneca; all outside the submitted work. J Ärnlöv reports payment or honoraria for lectures, presentations, speakers bureaus, manuscript writing or educational events from AstraZeneca and Novartis; participation on a Data Safety Monitoring Board or Advisory Board with AstraZeneca and Boehringer Ingelheim; all outside the submitted work. Z Aryan reports support for the present manuscript from American Heart Association as funding to their institution, and from Brigham and Women's Hospital as an employee. M Ausloos reports grants or contracts from [Romanian National Authority for Scientific Research and Innovation, CNDS-UEFISCDI project number PN-III-P4-ID-PCCF-2016-0084, research grant (Oct 2018–Sept 2022), grant title “Understanding and modelling time-space patterns of psychology-related inequalities and polarization” outside the submitted work. T Bärnighausen reports grants or contracts from Research grants from the European Union (Horizon 2020 and EIT Health), German Research Foundation (DFG), US National Institutes of Health, German Ministry of Education Research, Alexander von Humboldt Foundation, Else-Kröner-Fresenius-Foundation, Wellcome Trust, Bill & Melinda Gates Foundation, KfW, UNAIDS, and WHO; consulting fees from KfW on the OSCAR initiative in Vietnam; participation on a Data Safety Monitoring Board or Advisory Board with the NIH-funded study “Healthy Options” (PIs: Smith Fawzi, Kaaya), Chair of the Data Safety and Monitoring Board (DSMB), German National Committee on the “Future of Public Health Research and Education”, Chair of the scientific advisory board to the EDCTP Evaluation, Member of the UNAIDS Evaluation Expert Advisory Committee, National Institutes of Health Study Section Member on Population and Public Health Approaches to HIV/AIDS (PPAH), US National Academies of Sciences, Engineering, and Medicine's Committee for the “Evaluation of Human Resources for Health in the Republic of Rwanda under the President's Emergency Plan for AIDS Relief (PEPFAR)”, and University of Pennsylvania (UPenn) Population Aging Research Center (PARC) External Advisory Board Member; leadership or fiduciary role in board, society, committee or advocacy group, paid or unpaid as the co-chair of the Global Health Hub Germany (which was initiated by the German Ministry of Health); all outside the submitted work. E Beghi reports grants or contracts from Italian Health Ministry, American ALS Association, and SOBI Pharmaceutical Company made to their institution; payment or honoraria for lectures, presentations, speakers bureaus, manuscript writing or educational events from University of Rochester; support for attending meetings and/or travel from ILAE; participation on a Data Safety Monitoring Board or Advisory Board with Arvelle Therapeutics; all outside the submitted work. Y Béjot reports payment or honoraria for lectures, presentations, speakers bureaus, manuscript writing or educational events from Medtronic, Boehringer-Ingelheim, Pfizer, BMS, Servier, and Amgen; support for attending meetings and/or travel from Servier; all outside the submitted work. A Catapano reports grants or contracts from Sanofi, Eli Lilly, Mylan, Sanofi Regeneron, Menarini, and Amgen; payment or honoraria for lectures, presentations, speakers bureaus, manuscript writing or educational events from Akcea, Amgen, AstraZeneca, Aegerion, Amryt, Daiichi, Sankyo, Esperion, Kowa, Ionis Pharmaceuticals, Mylan, Merck, Menarini, Novartis, Recordati, Regeneron, Sandoz, and Sanofi; all outside the submitted work. S Costanzo reports grants or contracts from ERAB (the European Foundation for Alcohol Research; id. EA1767; 2018–2020 and Italian Ministry of Health (grant RF-2018-12367074, CoPI), both paid to their institution; payment or honoraria for lectures, presentations, speakers bureaus, manuscript writing or educational events from as a member of the Organizing Committee and speaker for the 9th European Beer and Health Symposium (Bruxelles 2019) and for given lecture at the 13th European Nutrition Conference FENS 2019 (Dublin), sponsored by the Beer and Health Initiative (The Dutch Beer Institute foundation—The Brewers of Europe); all outside the submitted work. M Endres reports grants or contracts from Bayer as an unrestricted grant to Charité for MonDAFIS study and Berlin AFib registry; consulting fees from Bayer paid to their institution; payment or honoraria for lectures, presentations, speakers bureaus, manuscript writing or educational events from Bayer, Boehringer Ingelheim, Pfizer, Amgen, GSK, Sanofi, and Novartis, all paid to their institution; participation on a Data Safety Monitoring Board or Advisory Board with BMS as a Country PI for Axiomatic-SSP, Bayer as Country PO for NAVIGATE-ESUS, AstraZeneca, Boehringer Ingelheim, Daiichi Sankyo, Amgen, Covidien, with all fees paid to their institution; leadership or fiduciary role in board, society, committee or advocacy group, paid or unpaid with EAN as part of the Board of Directors, DGN, ISCBFM, AHA/ASA, ESO, WSO, DZHK (German Centre of Cardiovascular Research) as a PI, all of which are unpaid positions, and with DZNE (German Center of Neurodegenerative Diseases) as a paid PI; receipt of PCSK9 inhibitors for mouse studies from Amgen; all outside the submitted work. I Filip reports payment or honoraria for lectures, presentations, speakers bureaus, manuscript writing or educational events from Avicenna Medical and Clinical Research Institute, outside the submitted work. A Gialluisi reports grants or contracts from Italian Ministry of Economic Development (PLATONE project, bando “Agenda Digitale” PON I&C 2014–2020; Prog. n. F/080032/01-03/X35) paid to their institution, outside the submitted work. C Herteliu reports grants or contracts from Romanian National Authority for Scientific Research and Innovation, CNDS-UEFISCDI project number PN-III-P4-ID-PCCF-2016-0084 research grant (Oct 2018–Sept 2022) “Understanding and modelling time-space patterns of psychology-related inequalities and polarization,” and project number PN-III-P2-2.1-SOL-2020-2-0351 research grant (June–Oct, 2021) “Approaches within public health management in the context of COVID-19 pandemic,” and from the Ministry of Labour and Social Justice, Romania, project number 30/PSCD/2018 research grant (Sept 2018–June 2019) “Agenda for skills Romania 2020–2025;” all outside the submitted work. S Islam reports grants or contracts from National Heart Foundation Vanguard grant, Postdoctoral Fellowship and NHMRC Emerging Leadership Fellowship, outside the submitted work. Y Kalkonde reports grants or contracts from DBT/Wellcome Trust India Alliance as a DBT/Wellcome Trust India Alliance fellow in Public Health (grant number IA/CPHI/14/1/501514). M Kivimäki reports support for the present manuscript from The Wellcome Trust (221854/Z/20/Z) and Medical Research Council (MR/S011676/1), as research grants paid to their institute. K Krishnan reports non-financial support from UGC Centre of Advanced Study, Phase II, Department of Anthropology, Panjab University, Chandigarh, India, outside the submitted work. P Lavados reports grants or contracts from Boehringer Ingelheim as grant support to their institute for RECCA stroke registry, and from ANID as personal grant support for ADDSPISE trial; payment or honoraria for lectures, presentations, speakers bureaus, manuscript writing or educational events from Boehringer Ingelheim as personal honoraria for lectures; support for attending meetings and/or travel from Boehringer Ingelheim as support for attending Global Stroke Netowrk meeting in 2020; all outside the submitted work. W Lo reports grants or contracts from 1U01NS106655-01A1 (MPI: S Ramey, Lo), 5U24NS1072050-02 (PI: Kolb), and 1P2CHD101912-01 (PD: S. Ramey), all outside the submitted work. S Lorkowski reports grants or contracts from Akcea Therapeutics Germany as payments made to their institution; consulting fees from Danone, Swedish Orphan Biovitrum (SOBI), and Upfield; payment or honoraria for lectures, presentations, speakers bureaus, manuscript writing or educational events from Akcea Therapeutics Germany, AMARIN Germany, Amedes Holding, AMGEN, Berlin-Chemie, Boehringer Ingelheim Pharma, Daiichi Sankyo Deutschland, Danone, Hubert Burda Media Holding, Lilly Deutschland, Novo Nordisk Pharma, Roche Pharma, Sanofi-Aventis, and SYNLAB Holding Deutschland & SYNLAB Akademie as personal payments; support for attending meetings and/or travel from Amgen as personal payments; participation on a Data Safety Monitoring Board or Advisory Board with Akcea Therapeutics Germany, Amgen, Daiichi Sankyo Deutschland, and Sanofi-Aventis as personal payments; all outside the submitted work. N Manafi reports support for the present manuscript from BMGF as funding to IHME for this project. B Norrving reports consulting fees from AstraZeneca and Bayer as personal payments outside the submitted work. O Odukoya reports grants or contracts from the Fogarty International Center of the National Institutes of Health as protected time towards the research reported in this publication was supported under the Award Number K43TW010704. The content is solely the responsibility of the authors and does not necessarily represent the official views of the National Institutes of Health. A Pana reports grants or contracts from Romanian National Authority for Scientific Research and Innovation, CNDS-UEFISCDI project number PN-III-P4-ID-PCCF-2016-0084 research grant (Oct 2018–Sept 2022) “Understanding and modelling time-space patterns of psychology-related inequalities and polarization,” and project number PN-III-P2-2·1-SOL-2020-2-0351 research grant (June–Oct 2021) “Approaches within public health management in the context of COVID-19 pandemic,” all outside the submitted work. M Postma reports leadership or fiduciary role in other board, society, committee or advocacy group, paid or unpaid with the UK's JCVI as an unpaid member, outside the current manuscript. A Radfar reports payment or honoraria for lectures, presentations, speakers' bureaus, manuscript writing or educational events from Avicenna Medical and Clinical Research Institute. S Sacco reports grants or contracts from Novartis and Allergan-AbbVie; consulting fees from Allergan-AbbVie, Novartis, Eli Lilly, AstraZeneca, and Novo Nordisk; payment or honoraria for lectures, presentations, speakers bureaus, manuscript writing or educational events from Allergan-AbbVie, Novartis, Eli Lilly, TEVA, Abbott, Medscape, and Olgology; support for attending meetings and/or travel from Allergan, Eli Lilly, Abbott, Novartis, and Teva; leadership or fiduciary role in other board, society, committee or advocacy group, paid or unpaid with Guideline Board European Stroke Organization as Co-chair, and with the European Headache Federation as a board member; all outside the submitted work. A Schutte reports payment or honoraria for lectures, presentations, speaker's bureaus, manuscript writing or educational events from Sanofi, Takeda, Abbott, Servier, and Omron Healthcare as honoraria for lectures during educational events; support for attending meetings and/or travel from Takeda and Omron; all outside the submitted work. J Singh reports consulting fees from Crealta/Horizon, Medisys, Fidia, Two labs, Adept Field Solutions, Clinical Care options, Clearview healthcare partners, Putnam associates, Focus forward, Navigant consulting, Spherix, MedIQ, UBM LLC, Trio Health, Medscape, WebMD, and Practice Point communications; and the National Institutes of Health and the American College of Rheumatology; payment or honoraria for lectures, presentations, speakers bureaus, manuscript writing or educational events from Simply Speaking; support for attending meetings and/or travel from OMERACT, an international organization that develops measures for clinical trials and receives arm's length funding from 12 pharmaceutical companies, when traveling to OMERACT meetings; participation on a Data Safety Monitoring Board or Advisory Board as a member of the FDA Arthritis Advisory Committee; leadership or fiduciary role in other board, society, committee or advocacy group, paid or unpaid, with OMERACT as a member of the steering committee, with the Veterans Affairs Rheumatology Field Advisory Committee as a member, and with the UAB Cochrane Musculoskeletal Group Satellite Center on Network Meta-analysis as a director and editor; stock or stock options in TPT Global Tech, Vaxart pharmaceuticals, Charlotte's Web Holdings and previously owned stock options in Amarin, Viking, and Moderna pharmaceuticals; all outside the submitted work. S Stortecky reports grants or contracts from Edwards Lifesciences, Medtronic, Abbott, and Boston Scientific as grants made to their institution; consulting fees from Boston Scientific/BTG Teleflex; payment or honoraria for lectures, presentations, speakers bureaus, manuscript writing or educational events from Boston Scientific; all outside the submitted work. M Woodward reports consulting fees from Amgen as personal payment. All other authors declare no competing interests.
