# Supplementary material for: Global, regional, and national burden of stroke and its risk factors, 1990–2019: a systematic analysis for the Global Burden of Disease Study 2019
Source: Lancet Neurol. 2021 Oct;20(10):795–820. doi: 10.1016/S1474-4422(21)00252-0 (PMC8443449; doi:10.1016/S1474-4422(21)00252-0)
Supplement: Supplementary appendix [file mmc1.pdf]

THE LANCET
Neurology

Supplementary appendix

This appendix formed part of the original submission and has been peer reviewed. We post it as supplied by the authors.

Supplement to: GBD 2019 Stroke Collaborators. Global, regional, and national burden of stroke and its risk factors, 1990–2019: a systematic analysis for the Global Burden of Disease Study 2019. *Lancet Neurol* 2021; published online Sept 3. https://doi.org/10.1016/S1474-4422(21)00252-0.

SUPPLEMENTARY MATERIALS

These supplementary materials formed part of the original submission and have been peer reviewed. We post it as supplied by the authors.

Supplement to: Global, regional and national burden of stroke and its risk factors, 1990-2019: A systematic analysis for the Global Burden of Disease Study 2019. The Lancet Neurology 2021:

Portions of this Methods section have been reproduced or adapted from Appendix of methodological detail for "Global burden of 369 diseases and injuries in 204 countries and territories, 1990–2019: a systematic analysis for the Global Burden of Disease Study 2019"[1] and "Global burden of 87 risk factors in 204 countries and territories, 1990-2019: a systematic analysis for the Global Burden of Disease Study 2019"[2] as well as from Johnson et al.,[3] Roth et al.,[4] James et al.,[5] Kyu et al.,[6] and Stanaway et al.[7] References are provided for reproduced sections.

Table of Contents

Authors' Contributions 7

Section 1. Summary of GBD methods for stroke burden estimates 11

1.1 Input data 11

1.2 Search terms for systematic review 12

1.3 Severity split inputs 14

1.3.1 Acute stroke severity splits 15

1.3.2 Chronic stroke severity splits 15

1.4 Modelling strategy 16

1.5 Data sources and types 18

1.6 Stroke definition 20

1.7 Modelling strategy 20

1.7.1 Ischaemic stroke 22

1.7.1.1 Input data 22

1.7.1.2 Modelling strategy 22

1.7.2 Intracerebral haemorrhage 23

1.7.2.1 Input data 23

1.7.2.2 Modelling strategy 23

1.7.3 Subarachnoid haemorrhage 24

1.7.3.1 Input data 24

1.7.3.2 Modelling strategy 24

1.8 Methods tables 25

1.8.1 Table 1. ICD Codes used in fatal and non-fatal analysis 25

1.8.2 Table 2. Selected covariates for CODEm models, overall stroke and subtypes 25

1.8.3 Table 3. Counts of data used by measure and stroke model for GBD 2019 26

1.8.4 Table 4. DisMod covariates 28

1.8.5 Table 5. Sequelae and disability weights for ischaemic stroke, intracerebral haemorrhage and subarachnoid haemorrhage 30

Section 2. Summary of GBD methods for estimates of burden of risk factors 31

2.1 Definition of risk factors and their estimation 31

2.2 Effect size estimation 33

2.2.1 Criteria for inclusion of risk-outcome pairs 33

2.2.2 The World Cancer Research Fund grading system 33

2.2.2.1 Convincing evidence 33

2.2.2.2 Probable evidence 33

2.2.2.3 Possible evidence 33

2.2.2.4 Insufficient evidence 33
2.3 Determine relative risks 34
2.4 Search terms and data preparation 35
2.5 Data analysis 35
2.6 Estimates exposure 37
2.6.1 Theoretical minimum-risk exposure level (TMREL) 38
2.6.2 Estimate population-attributable fractions 38
2.6.3 Estimate summary exposure values 38
2.6.4 Mediation 39
2.6.5 Calculating the burden of multiple risk factors....40
2.6.6 Adjusting for mediation 40
2.6.7 Calculating mediation factor....40
2.6.8 Uncertainty of aggregated and mediated PAFs 41
2.6.9 Important assumptions in aggregating risk factors and including mediation....41
2.6.10 Estimate attributable burden 41
2.6.11 Decomposition analysis of deaths and DALYs....41
Section 3. Changes in the modelling of stroke for GBD 2019 42
Section 4. Details of the Results....45
4.1 Overall stroke burden 45
4.2 Burden of pathological types of stroke.... 46
4.3 Stroke related DALYs attributable to risk factors.... 47
Section 5. Supplementary tables 48
Supplement Table 1. Incident case, death, prevalent cases, and DALYs for stroke in 2019 and percentage change of age-standardised rates for 1990-2019, by location 48
Supplement Table 2. Absolute number (in millions) and age-standardised rates per 100,000 people per year, with 95% uncertainty intervals (UI), of incident and prevalent stroke, deaths from stroke and DALYs due to stroke in 2019 and percentage change in the metrics for 1990-2019, by sex and pathological types of stroke....64
Supplement Table 3. Incident case, death, prevalent cases, and DALYs for ischaemic stroke in 2019 and percentage change of age-standardised rates for 1990-2019, by location 66
Supplement Table 4. Incident case, death, prevalent cases, and DALYs for intracerebral haemorrhage in 2019 and percentage change of age-standardised rates for 1990-2019, by location 82
Supplement Table 5. Incident case, death, prevalent cases, and DALYs for subarachnoid haemorrhage in 2019 and percentage change of age-standardised rates for 1990-2019, by location 98
Supplement Table 6. Ischaemic stroke related DALYs (absolute numbers [in millions with 95% UI] and percentage [with 95% UI]) associated with risk factors and their clusters in 2019, by World Bank country income level, all ages, both sexes....114

Supplement Table 7. Intracerebral haemorrhage related DALYs (absolute numbers [in millions with 95% UI] and percentage [with 95% UI]) associated with risk factors and their clusters in 2019, by World Bank country income level, all ages, both sexes 117

Supplement Table 8. Subarachnoid haemorrhage related DALYs (absolute numbers [in millions with 95% UI] and percentage [with 95% UI]) associated with risk factors and their clusters in 2019, by World Bank country income level, all ages, both sexes 120

Supplement Table 9a. Percent of DALYs (with 95% uncertainty intervals) due to stroke for high systolic blood pressure, high body-mass index, high fasting plasma glucose, high LDL cholesterol, low glomerular filtration rate, diet low in vegetable, diet low in fruits and diet high in sodium by location for both sexes combined in 2019, all ages 122

Supplement Table 9b. Percent of DALYs (with 95% uncertainty intervals) due to stroke for diet low in whole grains, alcohol use, low physical activity, smoking, second-hand smoking, ambient particulate matter$_{2.5}$ pollution, household air pollution from solid fuels and low ambient temperature by location for both sexes combined in 2019, all ages 137

Supplement Table 10a. Percent of DALYs (with 95% uncertainty intervals) due to ischaemic stroke for high systolic blood pressure, high body-mass index, high fasting plasma glucose, high LDL cholesterol, low glomerular filtration rate, diet low in vegetable, diet low in fruits and diet high in sodium by location for both sexes combined in 2019, all ages 151

Supplement Table 10b. Percent of DALYs (with 95% uncertainty intervals) due to ischaemic stroke for diet low in whole grains, alcohol use, low physical activity, smoking, second-hand smoking, ambient particulate matter$_{2.5}$ pollution, household air pollution from solid fuels and low ambient temperature by location for both sexes combined in 2019, all ages 166

Supplement Table 11a. Percent of DALYs (with 95% uncertainty intervals) due to intracerebral haemorrhage for high systolic blood pressure, high body-mass index, high fasting plasma glucose, high LDL cholesterol, low glomerular filtration rate, diet low in vegetable, diet low in fruits and diet high in sodium by location for both sexes combined in 2019, all ages 180

Supplement Table 11b. Percent of DALYs (with 95% uncertainty intervals) due to intracerebral haemorrhage for diet low in whole grains, alcohol use, low physical activity, smoking, second-hand smoking, ambient particulate matter$_{2.5}$ pollution, household air pollution from solid fuels and low ambient temperature by location for both sexes combined in 2019, all ages........194

Supplement Table 12a. Percent of DALYs (with 95% uncertainty intervals) due to subarachnoid haemorrhage for high systolic blood pressure, high body-mass index, high fasting plasma glucose, high LDL cholesterol, low glomerular filtration rate, diet low in vegetable, diet low in fruits and diet high in sodium by location for both sexes combined in 2019, all ages 203

Supplement Table 12b. Percent of DALYs (with 95% uncertainty intervals) due to subarachnoid haemorrhage for diet low in whole grains, alcohol use, low physical activity, smoking, second-hand smoking, ambient particulate matter$_{2.5}$ pollution, household air pollution from solid fuels and low ambient temperature by location for both sexes combined in 2019, all ages........212

Supplement Table 13. Age-standardised rates (per 100,000 people) and percentages of DALYs (with 95% uncertainty intervals) due to stroke in 2019 attributable to metabolic, behavioural and environmental/occupational risk clusters and all risk factors combined in low-income and middle-income countries, high-income countries and globally by sex........221

Supplement Table 14. Age-standardised rates (per 100,000 people) and percentages of DALYs (with 95% uncertainty intervals) due to ischaemic stroke in 2019 attributable to metabolic, behavioural and environmental/occupational risk clusters and all risk factors combined in low-income and middle-income countries, high-income countries and globally by sex........222

Supplement Table 15. Age-standardised rates (per 100,000 people) and percentages of DALYs (with 95% uncertainty intervals) due to intracerebral haemorrhage in 2019 attributable to metabolic, behavioural and environmental/occupational risk clusters and all risk factors combined in low-income and middle-income countries, high-income countries and globally by sex 223

Supplement Table 16. Age-standardised rates (per 100,000 people) and percentages of DALYs (with 95% uncertainty intervals) due to subarachnoid haemorrhage in 2019 attributable to metabolic, behavioural and environmental/occupational risk clusters and all risk factors combined in low-income and middle-income countries, high-income countries and globally by sex 224

Section 6. Supplementary figures 225

6.1 Supplement Figure 1. 21 GBD regions 225

6.2 Supplement Figure 2. Global number of deaths and DALYs by causes in 2019, both sexes, all ages ... 227

6.3 Supplement Figure 3. Absolute number (in millions) incident and prevalent strokes, deaths from stroke and DALYs due to stroke (shadow areas are 95% uncertainty intervals) by year from 1990 to 2019 228

6.4 Supplement Figure 4. Age-standardized stroke death rates per 100,000 people in the world in 2019 230

6.5 Supplement Figure 5. Age-standardized stroke prevalence per 100,000 people in the world in 2019 231

6.6 Supplement Figure 6. Age-standardized stroke DALYs rates per 100,000 people in the world in 2019 232

6.7 Supplement Figure 7. Annual stroke incidence rate and prevalence per 100,000 people aged <70 years and people aged 55-89 years (shadow areas are 95% uncertainty intervals) 233

6.8 Supplement Figure 8. Annual age-standardised incidence rates of ischaemic stroke (IS), intracerebral haemorrhage (ICH) and subarachnoid haemorrhage (SAH) per 100,000 people from 1990 to 2019 (shadow areas are 95% uncertainty intervals) 234

6.9 Supplement Figure 9. Annual age-standardised incidence rates of ischaemic stroke (IS), intracerebral haemorrhage (ICH) and subarachnoid haemorrhage (SAH) per 100,000 people in high-income countries (HIC) and low- to middle-income countries (LMIC) from 1990 to 2019 by sex (shadow areas are 95% uncertainty intervals) 235

6.10 Supplement Figure 10. Annual age-standardised incidence rates of ischaemic stroke (IS), intracerebral haemorrhage (ICH) and subarachnoid haemorrhage (SAH) per 100,000 people in males and females from 1990 to 2019 (shadow areas are 95% uncertainty intervals) 236

6.11 Supplement Figure 11. Age-standardised stroke prevalence, death and DALYs rates per 100,000 people by stroke type and country, for both sexes, 2019 237

6.12 Supplement Figure 12. Age-standardised population-attributable fraction of DALYs of ischaemic stroke due to the three risk clusters and five main risk factors, for both sexes, 2019 240

6.13. Supplement Figure 13. Age-standardised population-attributable fraction of DALYs of intracerebral haemorrhage due to the three risk clusters and five main risk factors, for both sexes, 2019 242

6.14. Supplement Figure 14. Age-standardised population-attributable fraction of DALYs of subarachnoid haemorrhage due to the three risk clusters and five main risk factors, for both sexes, 2019 244

6.15 Supplement Figure 15. Global stroke related DALYs attributable to risk factors in 1990 and 2019 (rank based on total number of all ages DALYs) 246

6.16 Supplement Figure 16. Global ischaemic stroke related DALYs attributable to risk factors in 1990 and 2019 (rank based on total number of all ages DALYs) 247

6.17 Supplement Figure 17. Global intracerebral haemorrhage related DALYs attributable to risk factors in 1990 and 2019 (rank based on total number of all ages DALYs) 248
6.18 Supplement Figure 18. Global subarachnoid haemorrhage related DALYs attributable to risk factors in 1990 and 2019 (rank based on total number of all ages DALYs) 249
6.19 Supplement Figure 19. Age-standardised population-attributable fraction of DALYs of stroke due to low daily ambient temperature, for both sexes, 2019 250
6.20 Supplement Figure 20. Age-standardised population-attributable fraction of DALYs of stroke due to high daily ambient temperature, for both sexes, 2019 251
6.21 Supplement Figure 21. Stroke DALYs attributable to all risk factors combined, all ages, both sexes, 1990-2019 252
6.22 Supplement Figure 22. Age-standardised percentage of stroke DALYs (with 95% uncertainty intervals) attributable to all risk factors in males and females, 2019 253
6.23 Supplement Figure 23. Age-standardised percentage of ischaemic stroke DALYs (with 95% uncertainty intervals) attributable to all risk factors in males and females, 2019 255
6.24 Supplement Figure 24. Age-standardised percentage of intracerebral haemorrhage DALYs (with 95% uncertainty intervals) attributable to all risk factors in males and females, 2019 257
6.25 Supplement Figure 25. Age-standardised percentage of subarachnoid haemorrhage DALYs (with 95% uncertainty intervals) attributable to all risk factors in males and females, 2019 259
6.26 Supplement Figure 26. Years of healthy life lost (DALYs) in millions, 2019 261
REFERENCES 262

Authors' Contributions

Managing the estimation or publications process
Catherine Owens Johnson, Gregory A Roth, Catherine Bisignano, and Christopher J L Murray.

Writing the first draft of the manuscript
Valery L Feigin and Catherine Bisignano.

Primary responsibility for applying analytical methods to produce estimates
Benjamin A Stark, Gregory A Roth, and Christopher J L Murray.

Primary responsibility for seeking, cataloguing, extracting, or cleaning data; designing or coding figures and tables
Valery L Feigin, and Benjamin A Stark. Valery L. Feigin and Gregory A. Roth verified the data underlying this study.

Providing data or critical feedback on data sources
Gdiom Gebreheat Abady, Mitra Abbasifard, Ahmed Abualhasan, Niveen ME Abu-Rmeileh, Oladimeji M Adebayo, Bright Opoku Ahinkorah, Sohail Ahmad, Sepideh Ahmadi, Yusra Ahmed Salih, Budi Aji, Hanadi Al Hamad, Fares Alahdab, Sheikh Mohammad Alif, Vahid Alipour, Syed Mohamed Aljunid, Sami Almustanyir, Nelson Alvis-Guzman, Deanna Anderlini, Jalal Arabloo, Kurnia Dwi Artanti, Zahra Aryan, Alok Atreya, Marcel Ausloos, Atif Amin Baig, Ovidiu Constantin Baltatu, Maciej Banach, Miguel A Barboza, Till Winfried Bärnighausen, Sanjay Basu, Gholamreza Bazmandegan, Yannick Béjot, Derrick A Bennett, Woldesellassie M Mequanint Bezabhe, Akshaya Srikanth Bhagavathula, Boris Bikbov, Michael Brauer, Florentino Luciano Caetano dos Santos, Ismael R Campos-Nonato, Juan J Carrero, Carlos A Castañeda-Orjuela, Dinh-Toi Chu, Vera Marisa Costa, Michael H Criqui, Albertino Antonio Moura Damasceno, Emanuele D'Amico, Lalit Dandona, Rakhi Dandona, Kairat Davletov, Rupak Desai, Samath Dhamminda Dharmaratne, Mandira Lamichhane Dhimal, Meghnath Dhimal, Martin Dichgans, Klara Dokova, Rajkumar Doshi, Abdel Douiri, Bruce B Duncan, Michael Ekholuenetale, Nevine El Nahas, Islam Y Elgendy, Aman Yesuf Endries, Farshad Farzadfar, David Flood, Mohamed M Gad, Shilpa Gaidhane, Reza Ghanei Gheshlagh, Ahmad Ghashghaee, Nermin Ghith, Sherief Ghozy, Simona Giampaoli, Syed Amir Gilani, Paramjit Singh Gill, Mahaveer Golechha, Vivek Kumar Gupta, Pradip Gyanwali, Nima Hafezi-Nejad, Treska S Hassan, Hamid Yimam Hassen, Rasmus J Havmoeller, Mohamed I Hegazy, Claudiu Herteliu, Mowafa Househ, Segun Emmanuel Ibitoye, Seyed Sina Naghibi Irvani, Sheikh Mohammed Shariful Islam, Nahlah Elkudssiah Ismail, Gaetano Isola, Vardhmaan Jain, Sathish Kumar Jayapal, Shubha Jayaram, Panniyammakal Jeemon, Jost B Jonas, Jacek Jerzy Jozwiak, Mikk Jürisson, Rizwan Kalani, Rohollah Kalhor, Zahra Kamiab, Himal Kandel, André Karch, Patrick DMC Katoto, Gbenga A Kayode, Yousef Saleh Khader, Ejaz Ahmad Khan, Maseer Khan, Mahalaqua Nazli Khatib, Jagdish Khubchandani, Gyu Ri Kim, Min Seo Kim, Yun Jin Kim, Adnan Kisa, Sezer Kisa, Kewal Krishan, Vijay Krishnamoorthy, G Anil Kumar, Dian Kusuma, Hassan Mehmood Lak, Savita Lasrado, Ro-Ting Lin, Xuefeng Liu, Stefan Lorkowski, Hassan Magdy Abd El Razek, Alaa Makki, Reza Malekzadeh, Navid Manafi, Mohammad Ali Mansournia, Lorenzo Giovanni Mantovani, Man Mohan Mehndiratta, Ritesh G Menezes, Atte Meretoja, Bartosz Miazgowski, Irmina Maria Michalek, Erkin M Mirrakhimov, Abdollah Mohammadian-Hafshejani, Shafiu Mohammed, Ali H Mokdad, Mariam Molokhia, Mohammad Ali Ali Moni, Jakub Morze, Walter Muruet, Kamarul Imran Musa, Ahamarshan Jayaraman Nagarajan, Mohsen Naghavi, Sreenivas Narasimha Swamy, Bruno Ramos Nascimento, Ruxandra Irina Negoi, Sandhya Neupane Kandel, Trang Huyen Nguyen, Bo Norrving, Jean Jacques Noubiap, Vincent Ebuka Nwatah, Bogdan Oancea, Andrew T Olagunju, Hans Orru, Mayowa O Owolabi, Jagadish Rao Padubidri, Adrian Pana, Eun-Cheol Park, Fatemeh Pashazadeh Kan, Mona Pathak, Mario F P Peres, Arokiasamy Perianayagam, Vivek Podder, Suzanne Polinder, Maarten J Postma, Alireza Rafiei, Fakher Rahim, Vafa Rahimi-Movaghar, Amir Masoud Rahmani, Chythra R Rao, Sowmya J Rao, Priya Rathi, David Laith Rawaf, Salman Rawaf, Vishnu Renjith, Andre M N Renzaho, Jefferson Antonio Buendia Rodriguez, Leonardo Roever, Andrzej Rynkiewicz, Rehab Salah, Abdallah M Samy, Milena M Santric-Milicevic, Brijesh Sathian, Maria Inês Schmidt, Sadaf G Sepanlou, Allen Seylani, Masood Ali Shaikh, Mohammed Shannawaz, Sara Sheikhbahaei, Soraya Siabani, Jasvinder A Singh, Jitendra

Kumar Singh, Valentin Yurievich Skryabin, Anna Aleksandrovna Skryabina, Mohamad-Hani Temsah, Marcello Tonelli, Marcos Roberto Tovani-Palone, Bach Xuan Tran, Anayat Ullah, Bhaskaran Unnikrishnan, Alireza Vakilian, Sahel Valadan Tahbaz, Tommi Juhani Vasankari, Narayanaswamy Venketasubramanian, Bay Vo, Giang Thu Vu, Linh Gia Vu, Yasir Waheed, Tissa Wijeratne, Charles D A Wolfe, Mark Woodward, Sarah Wulf Hanson, Ali Yadollahpour, Seyed Hossein Yahyazadeh Jabbari, Naohiro Yonemoto, Chuanhua Yu, Alireza Zandifar, Zhi-Jiang Zhang, Yves Miel H Zuniga and Christopher J L Murray.

Developing methods or computational machinery
Benjamin A Stark, Yusra Ahmed Salih, Michael Brauer, Dana Bryazka, Xiaochen Dai, Farshad Farzadfar, Shilpa Gaidhane, Reza Ghanei Gheshlagh, Mahaveer Golechha, Mowafa Househ, Gaetano Isola, Mahalaqua Nazli Khatib, Adnan Kisa, Sezer Kisa, Alaa Makki, Ali H Mokdad, Mohsen Naghavi, Amir Masoud Rahmani, Marissa B Reitsma, Abdallah M Samy, Bay Vo, Charles D A Wolfe, Sarah Wulf Hanson, Yves Miel H Zuniga, and Christopher J L Murray.

Providing critical feedback on methods or results
Benjamin A Stark, Gdiom Gebreheat Abady, Mitra Abbasifard, Foad Abd-Allah, Ahmed Abualhasan, Niveen ME Abu-Rmeileh, Abdelrahman I Abushouk, Oladimeji M Adebayo, Gina Agarwal, Pradyumna Agasthi, Bright Opoku Ahinkorah, Sohail Ahmad, Yusra Ahmed Salih, Budi Aji, Samaneh Akbarpour, Rufus Olusola Akinyemi, Hanadi Al Hamad, Fares Alahdab, Sheikh Mohammad Alif, Vahid Alipour, Syed Mohamed Aljunid, Sami Almustanyir, Rajaa M Al-Raddadi, Rustam Al-Shahi Salman, Nelson Alvis-Guzman, Robert Ancuceanu, Deanna Anderlini, Jalal Arabloo, Johan Ärnlöv, Zahra Aryan, Samaneh Asgari, Tahira Ashraf, Mohammad Athar, Alok Atreya, Marcel Ausloos, Atif Amin Baig, Ovidiu Constantin Baltatu, Maciej Banach, Miguel A Barboza, Suzanne Lyn Barker-Collo, Till Winfried Bärnighausen, Mark Thomaz Ugliara Barone, Sanjay Basu, Gholamreza Bazmandegan, Ettore Beghi, Mahya Beheshti, Yannick Béjot, Arielle Wilder Bell, Derrick A Bennett, Isabela M Bensenor, Woldesellassie M Mequanint Bezabhe, Yihienew Mequanint Bezabih, Akshaya Srikanth Bhagavathula, Pankaj Bhardwaj, Krittika Bhattacharyya, Ali Bijani, Boris Bikbov, Mulugeta M Birhanu, Archith Boloor, Aime Bonny, Hermann Brenner, Dana Bryazka, Zahid A Butt, Florentino Luciano Caetano dos Santos, Ismael R Campos-Nonato, Carlos Cantu-Brito, Carlos A Castañeda-Orjuela, Promit Ananyo Chakraborty, Jaykaran Charan, Sonali Gajanan Choudhari, Enayet Karim Chowdhury, Dinh-Toi Chu, Sheng-Chia Chung, David Colozza, Vera Marisa Costa, Simona Costanzo, Michael H Criqui, Omid Dadras, Baye Dagnew, Xiaochen Dai, Koustuv Dalal, Emanuele D'Amico, Lalit Dandona, Rakhi Dandona, Jiregna Darega Gela, Kairat Davletov, Vanessa De la Cruz-Góngora, Rupak Desai, Deepak Dhamnetiya, Samath Dhamminda Dharmaratne, Mandira Lamichhane Dhimal, Meghnath Dhimal, Daniel Diaz, Martin Dichgans, Klara Dokova, Rajkumar Doshi, Abdel Douiri, Sahar Eftekharzadeh, Michael Ekholuenetale, Nevine El Nahas, Islam Y Elgendy, Muhammed Elhadi, Aman Yesuf Endries, Daniel Asfaw Erku, Emerito Jose A Faraon, Umar Farooque, Farshad Farzadfar, Abdullah Hamid Feroze, Irina Filip, Florian Fischer, David Flood, Mohamed M Gad, Shilpa Gaidhane, Reza Ghanei Gheshlagh, Nermin Ghith, Ghozali Ghozali, Sherief Ghozy, Alessandro Gialluisi, Paramjit Singh Gill, Elena V Gnedovskaya, Mahaveer Golechha, Alessandra C Goulart, Yuming Guo, Rajeev Gupta, Veer Bala Gupta, Vivek Kumar Gupta, Pradip Gyanwali, Nima Hafezi-Nejad, Samer Hamidi, Asif Hanif, Graeme J Hankey, Arief Hargono, Abdiwahab Hashi, Treska S Hassan, Hamid Yimam Hassen, Rasmus J Havmoeller, Simon I Hay, Khezar Hayat, Mohamed I Hegazy, Claudiu Herteliu, Ramesh Holla, Mowafa Househ, Ayesha Humayun, Bing-Fang Hwang, Licia Iacoviello, Segun Emmanuel Ibitoye, Olayinka Stephen Ilesanmi, Irena M Ilic, Milena D Ilic, Usman Iqbal, Seyed Sina Naghibi Irvani, Sheikh Mohammed Shariful Islam, Nahlah Elkudssiah Ismail, Gaetano Isola, Masao Iwagami, Louis Jacob, Vardhmaan Jain, Sung-In Jang, Sathish Kumar Jayapal, Shubha Jayaram, Ranil Jayawardena, Panniyammakal Jeemon, Ravi Prakash Jha, Walter D Johnson, Jost B Jonas, Jacek Jerzy Jozwiak, Mikk Jürisson, Rizwan Kalani, Rohollah Kalhor, Yogeshwar Kalkonde, Ashwin Kamath, Zahra Kamiab, Tanuj Kanchan, Himal Kandel, André Karch, Patrick DMC Katoto, Gbenga A Kayode, Pedram Keshavarz, Yousef Saleh Khader, Ejaz Ahmad Khan, Imteyaz A Khan, Maseer Khan, Moien AB Khan, Mahalaqua Nazli Khatib, Jagdish Khubchandani, Gyu Ri Kim, Min Seo Kim, Yun Jin Kim, Adnan Kisa, Sezer Kisa, Mika Kivimäki, Dhaval Kolte, Ali Koolivand, Sindhura Lakshmi Koulmane Laxminarayana, Ai Koyanagi, Kewal Krishan, Vijay Krishnamoorthy, G Anil Kumar, Dian Kusuma, Carlo La Vecchia, Hassan Mehmood Lak, Tea Lallukka, Savita Lasrado, Pablo M Lavados, Matilde Leonardi, Bingyu Li, Shanshan Li, Hualiang Lin, Ro-Ting Lin, Xuefeng Liu,

Stefan Lorkowski, Giancarlo Lucchetti, Ricardo Lutzky Saute, Hassan Magdy Abd El Razek, Francesca Giulia Magnani, Preetam Bhalchandra Mahajan, Azeem Majeed, Alaa Makki, Reza Malekzadeh, Ahmad Azam Malik, Navid Manafi, Mohammad Ali Mansournia, Lorenzo Giovanni Mantovani, Santi Martini, Giampiero Mazzaglia, Man Mohan Mehndiratta, Ritesh G Menezes, Atte Meretoja, Amanual Getnet Mersha, Junmei Miao Jonasson, Tomasz Miazgowski, Irmina Maria Michalek, Erkin M Mirrakhimov, Yousef Mohammad, Abdollah Mohammadian-Hafshejani, Shafiu Mohammed, Ali H Mokdad, Yaser Mokhayeri, Mariam Molokhia, Mohammad Ali Ali Moni, Ahmed Al Montasir, Lidia Morawska, Jakub Morze, Walter Muruet, Kamarul Imran Musa, Ahamarshan Jayaraman Nagarajan, Mohsen Naghavi, Sreenivas Narasimha Swamy, Bruno Ramos Nascimento, Ruxandra Irina Negoi, Sandhya Neupane Kandel, Trang Huyen Nguyen, Bo Norrving, Jean Jacques Noubiap, Vincent Ebuka Nwatah, Bogdan Oancea, Andrew T Olagunju, Hans Orru, Mayowa O Owolabi, Jagadish Rao Padubidri, Adrian Pana, Tarang Parekh, Eun-Cheol Park, Mona Pathak, Mario F P Peres, Arokiasamy Perianayagam, Truong-Minh Pham, Michael A Piradov, Vivek Podder, Suzanne Polinder, Maarten J Postma, Akram Pourshams, Amir Radfar, Alireza Rafiei, Alberto Raggi, Fakher Rahim, Vafa Rahimi-Movaghar, Mosiur Rahman, Muhammad Aziz Rahman, Amir Masoud Rahmani, Nazanin Rajai, Priyanga Ranasinghe, Chythra R Rao, Sowmya J Rao, Priya Rathi, David Laith Rawaf, Salman Rawaf, Marissa B Reitsma, Vishnu Renjith, Andre M N Renzaho, Aziz Rezapour, Jefferson Antonio Buendia Rodriguez, Leonardo Roever, Michele Romoli, Simona Sacco, Sahar Saeedi Moghaddam, KM Saif-Ur-Rahman, Rehab Salah, Mehrnoosh Samaei, Abdallah M Samy, Itamar S Santos, Milena M Santric-Milicevic, Nizal Sarrafzadegan, Brijesh Sathian, Davide Sattin, Silvia Schiavolin, Markus P Schlaich, Aletta Elisabeth Schutte, Sadaf G Sepanlou, Allen Seylani, Feng Sha, Saeed Shahabi, Masood Ali Shaikh, Mohammed Shannawaz, Sara Sheikhbahaei, Kenji Shibuya, Soraya Siabani, Diego Augusto Santos Silva, Jasvinder A Singh, Jitendra Kumar Singh, Valentin Yurievich Skryabin, Anna Aleksandrovna Skryabina, Badr Hasan Sobaih, Stefan Stortecky, Saverio Stranges, Eyayou Girma Tadesse, Ingan Ukur Tarigan, Mohamad-Hani Temsah, Amanda G Thrift, Marcello Tonelli, Marcos Roberto Tovani-Palone, Bach Xuan Tran, Manjari Tripathi, Gebiyaw Wudie Tsegaye, Anayat Ullah, Bhaskaran Unnikrishnan, Alireza Vakilian, Sahel Valadan Tahbaz, Narayanaswamy Venketasubramanian, Dominique Vervoort, Bay Vo, Victor Volovici, Kia Vosoughi, Giang Thu Vu, Linh Gia Vu, Yasir Waheed, Yanzhong Wang, Tissa Wijeratne, Andrea Sylvia Winkler, Charles D A Wolfe, Mark Woodward, Xiaoyue Xu, Ali Yadollahpour, Seyed Hossein Yahyazadeh Jabbari, Kazumasa Yamagishi, Hiroshi Yatsuya, Naohiro Yonemoto, Chuanhua Yu, Ismaeel Yunusa, Sojib Bin Zaman, Maryam Zamanian, Alireza Zandifar, Mikhail Sergeevich Zastrozhin, Anasthasia Zastrozhina, Yunquan Zhang, Zhi-Jiang Zhang, Chenwen Zhong, Yves Miel H Zuniga, and Christopher J L Murray.

Drafting the work or revising is critically for important intellectual content

Catherine Bisignano, Mohsen Abbasi-Kangevari, Foad Abd-Allah, Vida Abedi, Ahmed Abualhasan, Niveen ME Abu-Rmeileh, Abdelrahman I Abushouk, Oladimeji M Adebayo, Gina Agarwal, Pradyumna Agasthi, Bright Opoku Ahinkorah, Sepideh Ahmadi, Budi Aji, Rufus Olusola Akinyemi, Fares Alahdab, Sami Almustanyir, Rustam Al-Shahi Salman, Nelson Alvis-Guzman, Robert Ancuceanu, Deanna Anderlini, Ippazio Cosimo Antonazzo, Jalal Arabloo, Johan Ärnlöv, Kurnia Dwi Artanti, Zahra Aryan, Tahira Ashraf, Alok Atreya, Marcel Ausloos, Atif Amin Baig, Ovidiu Constantin Baltatu, Maciej Banach, Miguel A Barboza, Suzanne Lyn Barker-Collo, Till Winfried Bärnighausen, Mark Thomaz Ugliara Barone, Sanjay Basu, Yannick Béjot, Arielle Wilder Bell, Derrick A Bennett, Isabela M Bensenor, Woldesellassie M Mequanint Bezabhe, Yihienew Mequanint Bezabih, Krittika Bhattacharyya, Boris Bikbov, Florentino Luciano Caetano dos Santos, Carlos Cantu-Brito, Juan J Carrero, Carlos A Castañeda-Orjuela, Alberico L Catapano, Enayet Karim Chowdhury, Dinh-Toi Chu, David Colozza, Vera Marisa Costa, Simona Costanzo, Baye Dagnew, Koustuv Dalal, Albertino Antonio Moura Damasceno, Rupak Desai, Deepak Dhamnetiya, Samath Dhamminda Dharmaratne, Mandira Lamichhane Dhimal, Meghnath Dhimal, Daniel Diaz, Klara Dokova, Rajkumar Doshi, Abdel Douiri, Bruce B Duncan, Sahar Eftekharzadeh, Islam Y Elgendy, Muhammed Elhadi, Shaimaa I El-Jaafary, Matthias Endres, Aman Yesuf Endries, Daniel Asfaw Erku, Emerito Jose A Faraon, Abdullah Hamid Feroze, Irina Filip, Florian Fischer, David Flood, Mohamed M Gad, Shilpa Gaidhane, Reza Ghanei Gheshlagh, Nermin Ghith, Sherief Ghozy, Alessandro Gialluisi, Paramjit Singh Gill, Elena V Gnedovskaya, Mahaveer Golechha, Alessandra C Goulart, Yuming Guo, Rajeev Gupta, Veer Bala Gupta, Vivek Kumar Gupta, Pradip Gyanwali, Nima Hafezi-Nejad, Asif Hanif, Graeme J Hankey, Abdiwahab Hashi, Treska S Hassan, Hamid Yimam Hassen, Rasmus J Havmoeller, Simon I Hay, Claudiu Herteliu, Ramesh Holla, Sorin Hostiuc, Mowafa

Househ, Junjie Huang, Ayesha Humayun, Licia Iacoviello, Ivo Iavicoli, Segun Emmanuel Ibitoye, Olayinka Stephen Ilesanmi, Irena M Ilic, Milena D Ilic, Usman Iqbal, Seyed Sina Naghibi Irvani, Sheikh Mohammed Shariful Islam, Nahlah Elkudssiah Ismail, Hiroyasu Iso, Gaetano Isola, Louis Jacob, Vardhmaan Jain, Sathish Kumar Jayapal, Shubha Jayaram, Panniyammakal Jeemon, Ravi Prakash Jha, Walter D Johnson, Jost B Jonas, Nitin Joseph, Jacek Jerzy Jozwiak, Mikk Jürisson, Rizwan Kalani, Himal Kandel, André Karch, Patrick DMC Katoto, Gbenga A Kayode, Pedram Keshavarz, Yousef Saleh Khader, Ejaz Ahmad Khan, Maseer Khan, Moien AB Khan, Mahalaqua Nazli Khatib, Jagdish Khubchandani, Gyu Ri Kim, Yun Jin Kim, Adnan Kisa, Sezer Kisa, Mika Kivimäki, Dhaval Kolte, Sindhura Lakshmi Koulmane Laxminarayana, Ai Koyanagi, Kewal Krishan, Rita V Krishnamurthi, Dian Kusuma, Carlo La Vecchia, Ben Lacey, Hassan Mehmood Lak, Tea Lallukka, Savita Lasrado, Pablo M Lavados, Matilde Leonardi, Shanshan Li, Hualiang Lin, Warren David Lo, Stefan Lorkowski, Giancarlo Lucchetti, Ricardo Lutzky Saute, Hassan Magdy Abd El Razek, Francesca Giulia Magnani, Preetam Bhalchandra Mahajan, Azeem Majeed, Reza Malekzadeh, Ahmad Azam Malik, Navid Manafi, Lorenzo Giovanni Mantovani, Giampiero Mazzaglia, Ritesh G Menezes, Atte Meretoja, Tomasz Miazgowski, Irmina Maria Michalek, Yousef Mohammad, Abdollah Mohammadian-Hafshejani, Shafiu Mohammed, Ali H Mokdad, Mariam Molokhia, Mohammad Ali Ali Moni, Ahmed Al Montasir, Rahmatollah Moradzadeh, Jakub Morze, Kamarul Imran Musa, Mohsen Naghavi, Sreenivas Narasimha Swamy, Bruno Ramos Nascimento, Ruxandra Irina Negoi, Sandhya Neupane Kandel, Trang Huyen Nguyen, Bo Norrving, Jean Jacques Noubiap, Vincent Ebuka Nwatah, Bogdan Oancea, Oluwakemi Ololade Odukoya, Andrew T Olagunju, Hans Orru, Mayowa O Owolabi, Jagadish Rao Padubidri, Adrian Pana, Eun-Cheol Park, Mona Pathak, Mario F P Peres, Arokiasamy Perianayagam, Truong-Minh Pham, Michael A Piradov, Vivek Podder, Suzanne Polinder, Maarten J Postma, Amir Radfar, Alireza Rafiei, Alberto Raggi, Fakher Rahim, Vafa Rahimi-Movaghar, Priyanga Ranasinghe, Chythra R Rao, Sowmya J Rao, David Laith Rawaf, Salman Rawaf, Vishnu Renjith, Andre M N Renzaho, Jefferson Antonio Buendia Rodriguez, Leonardo Roever, Andrzej Rynkiewicz, Simona Sacco, Masoumeh Sadeghi, Amirhossein Sahebkar, KM Saif-Ur-Rahman, Rehab Salah, Mehrnoosh Samaei, Abdallah M Samy, Itamar S Santos, Milena M Santric-Milicevic, Davide Sattin, Silvia Schiavolin, Markus P Schlaich, Maria Inês Schmidt, Aletta Elisabeth Schutte, Sadaf G Sepanlou, Allen Seylani, Saeed Shahabi, Mohammed Shannawaz, Md Shajedur Rahman Shawon, Aziz Sheikh, Sara Sheikhbahaei, Kenji Shibuya, Diego Augusto Santos Silva, Jasvinder A Singh, Valentin Yurievich Skryabin, Anna Aleksandrovna Skryabina, Stefan Stortecky, Saverio Stranges, Eyayou Girma Tadesse, Mohamad-Hani Temsah, Yvonne Teuschl, Amanda G Thrift, Marcello Tonelli, Marcos Roberto Tovani-Palone, Bach Xuan Tran, Manjari Tripathi, Anayat Ullah, Brigid Unim, Bhaskaran Unnikrishnan, Sahel Valadan Tahbaz, Tommi Juhani Vasankari, Narayanaswamy Venketasubramanian, Dominique Vervoort, Victor Volovici, Giang Thu Vu, Linh Gia Vu, Hatem A Wafa, Yasir Waheed, Yanzhong Wang, Tissa Wijeratne, Andrea Sylvia Winkler, Mark Woodward, Jason H Wu, Lalit Yadav, Seyed Hossein Yahyazadeh Jabbari, Kazumasa Yamagishi, Hiroshi Yatsuya, Naohiro Yonemoto, Muhammed Shahriar Zaman, Sojib Bin Zaman, Maryam Zamanian, Ramin Zand, Alireza Zandifar, Mikhail Sergeevich Zastrozhin, Anasthasia Zastrozhina, Chenwen Zhong, and Christopher J L Murray.

Extracting, cleaning, or cataloging data; designing or coding figures and tables
Benjamin A Stark, Mitra Abbasifard, Jason A Anderson, Adnan Ansar, Gholamreza Bazmandegan, Dana Bryazka, Michael H Criqui, Shilpa Gaidhane, Reza Ghanei Gheshlagh, Mowafa Househ, Gaetano Isola, Zahra Kamiab, Mahalaqua Nazli Khatib, Adnan Kisa, Sezer Kisa, Hassan Magdy Abd El Razek, Ali H Mokdad, Mohsen Naghavi, Abdallah M Samy, Anayat Ullah, Alireza Vakilian, Sarah Wulf Hanson, Ali Yadollahpour, Naohiro Yonemoto, Mikhail Sergeevich Zastrozhin, and Christopher J L Murray.

Section 1. Summary of GBD methods for stroke burden estimates

This section provides further methodological detail for "Global, regional and national burden of stroke and its risk factors, 1990-2019: A systematic analysis for the Global Burden of Disease Study 2019." This study complies with the Guidelines for Accurate and Transparent Health Estimates Reporting (GATHER) recommendations.[8] It includes detailed tables and information on data to maximize transparency in our estimation processes and provides a comprehensive description of analytical steps. We intend this supplementary material to be a living document, to be updated with each iteration of the Global Burden of Disease Study (GBD).

We produced estimates for 204 countries and territories that were grouped into 21 regions and seven super-regions (Supplement Figure 1). The seven super-regions are central Europe, eastern Europe, and central Asia; high income; Latin America and the Caribbean; north Africa and the Middle East; south Asia; southeast Asia, east Asia, and Oceania; and sub-Saharan Africa. For GBD 2019, nine countries and territories (Cook Islands, Monaco, San Marino, Nauru, Niue, Palau, Saint Kitts and Nevis, Tokelau, and Tuvalu) were added, such that the GBD location hierarchy now includes all WHO member states. This round, GBD includes subnational analyses for several new countries and continues to analyse at subnational levels countries that were added in previous cycles. Subnational estimation in GBD 2019 includes five new countries (Italy, Nigeria, Pakistan, the Philippines, and Poland) and 16 countries previously estimated at subnational levels (Brazil, China, Ethiopia, India, Indonesia, Iran, Japan, Kenya, Mexico, New Zealand, Norway, Russia, South Africa, Sweden, the UK, and the USA).

Results from GBD 2019 are available through an interactive data downloading tool on the Global Health Data Exchange (GHDx). The GHDx is the world's most comprehensive catalogue of surveys, censuses, vital statistics, and other health-related data. Results are measured in terabytes. The latest version of the data download tool, available here: http://ghdx.healthdata.org/GBD-resultstool, contains core summary results for GBD 2019. These results include deaths, years of life lost (YLLs), YLDs, disability-adjusted life-years (DALYs), prevalence, incidence, and rate of change. The GHDx includes data for causes, risks, cause-risk attribution, aetiologies, and impairments. Data above a certain size cannot be viewed online but can be downloaded. Depending on the size of the download, users may need to enter an email address; a download location will be sent to them when the files are prepared. All GBD 2019 online data visualisations are available at http://vizhub.healthdata.org/GBD-compare, which provides results for all GBD health metrics.

1.1 Input data

For the GBD 2019 study, in order to better represent population-level disease incidence for IS, adjustments for alternative study methods and case definitions were applied to data prior to analysis in DisMod-MR. These adjustments were performed using the MR-BRT modelling tool. We adjusted for several study-specific factors such as whether the data were from a hospital and whether the data included both first-ever and recurrent ischaemic strokes. We updated our methods for redistributing deaths due to unspecified stroke (ICD-10 codes I62 and I64) to the three modelled stroke subtypes included in GBD.

Verbal autopsy and vital registration data were used to model cerebrovascular disease (stroke). We reassigned deaths from verbal autopsy reports for cerebrovascular disease to the parent cardiovascular disease for both sexes for those under 20 years of age. We outliered non-representative subnational verbal autopsy datapoints. We also outliered ICD8, ICD9BTL, and tabulated ICD10 datapoints which were inconsistent with the rest of the data and created implausible time trends. Datapoints from sources which were implausibly low in all age groups and data points that were causing the regional estimates to be improbably high were outliered.

Tables 1a, 1b, and 1c display source count information for non-fatal ischaemic stroke, intracerebral haemorrhage, and subarachnoid haemorrhage respectively.

Table 1a: Source counts for ischaemic stroke models.

Measure	Total sources	Countries with data

All measures	523	76
Prevalence	117	24
Incidence	332	62
Excess mortality rate	141	47
Case fatality rate	50	22

Table 1b: Source counts for intracerebral haemorrhage models.

Measure	Total sources	Countries with data
All measures	502	74
Prevalence	117	24
Incidence	322	61
Excess mortality rate	125	41
Case fatality rate	40	18

Table 1c: Source counts for subarachnoid haemorrhage models.

Measure	Total sources	Countries with data
All measures	435	63
Prevalence	117	24
Incidence	260	47
Excess mortality rate	88	28

1.2 Search terms for systematic review

A systematic review was not performed for GBD 2019. However, a systematic review was performed for GBD 2017. Search terms, dates of search, and databases queried follow:

1) Ischaemic stroke
   a. Google scholar: ("ischemic stroke" OR “cerebral infarction” OR “ischaemic stroke”) AND (incidence OR prevalence OR mortality OR epidemiology). Reviewed first 1000 hits, sorted by relevance
   b. Global Index Medicus search: (tw:("ischemic stroke") OR tw:(“cerebral infarction” OR tw:(“ischaemic stroke”)) AND (tw:(incidence) OR tw:(prevalence) OR tw:(mortality) OR tw:(epidemiology)) AND NOT (tw:(rats) OR tw:(mice) OR tw:(dogs) OR tw:(apes) OR tw:(monkeys)). Dates of search: 01Jan2010 – 31Aug2017
2) Intracerebral haemorrhage
   a. Google scholar: ("hemorrhagic stroke" OR “intracerebral hemorrhage” OR “haemorrhagic stroke” OR “intracerebral haemorrhage”) AND (incidence OR prevalence OR mortality OR epidemiology). Reviewed first 1000 hits, sorted by relevance
   b. GIM search: (tw:("intracerebral hemorrhage") OR tw:(“intracerebral haemorrhage”) OR tw:(“hemorrhagic stroke”) OR tw:(“haemorrhagic stroke”)) AND (tw:(incidence) OR tw:(prevalence) OR tw:(mortality) OR tw:(epidemiology)) AND NOT (tw:(rats) OR tw:(mice) OR tw:(dogs) OR tw:(apes) OR tw:(monkeys)). Dates of search: 01Jan2010 – 31Aug2017
3) Subarachnoid haemorrhage
   a. Google scholar search: ("subarachnoid hemorrhage" OR “subarachnoid haemorrhage”) AND (incidence OR prevalence OR mortality OR epidemiology). Reviewed first 1000 hits, sorted by relevance.
   b. GIM search: (tw:("subarachnoid hemorrhage") OR tw:(“subarachnoid haemorrhage”)) AND (tw:(incidence) OR tw:(prevalence) OR tw:(mortality) OR tw:(epidemiology)) AND NOT (tw:(rats) OR tw:(mice) OR tw:(dogs) OR tw:(apes) OR tw:(monkeys)). Dates of search: 01Jan2010 – 31Aug2017

We included inpatient hospital data, adjusted for readmission and primary to any diagnosis using correction factors estimated from US claims data. We excluded data for locations where the data points were implausibly low (Vietnam, Philippines, India). In addition, we included unpublished stroke registry data for acute ischaemic stroke, acute intracerebral haemorrhage, and acute subarachnoid haemorrhage. We also included survey data for chronic stroke. These surveys were identified based on expert opinion and review of major survey series focused on world health that included questions regarding self- reported history of stroke. For GBD 2019, we split unspecified strokes (ICD-10 I64) into ischaemic stroke, intracerebral haemorrhage, and subarachnoid haemorrhage according to the proportions of subtype- specific coded strokes in the original data. We also split ICD-10 I62 into intracerebral haemorrhage, and subarachnoid haemorrhage using the same approach.

As with many models in GBD, the diversity of data sources available means that we needed to adjust available data to our reference case definition. We thus crosswalked incidence and excess mortality data that did not meet our reference case definitions using MR- BRT, a Bayesian meta-regression tool develop for the GBD. More information on MR-BRT can be found elsewhere in the supplementary materials.

We adjusted data points for first and recurrent strokes combined, using data for first strokes only as reference. For ischaemic stroke and intracerebral haemorrhage, we also adjusted data points that reported all stroke subtypes combined, using as reference studies with subtype-specific information. We also adjusted data which included only persons who survived to hospital admission, using as reference data on both fatal and nonfatal strokes. In addition, we adjusted subtype-specific, inpatient clinical informatics data using subtype-specific literature estimates as a reference. These adjustments can be examined more closely in Table 2. The coefficients in Tables 2a, 2b, and 2c below can be used to calculate adjustment factors for alternative definitions. The formula for computing adjustment factors is given in equation 1 below. We also included a standardized age variable (age scaled) and a sex variable to the crosswalking procedure to adjust for the possibly of bias.

Equation 1: Calculation of adjustment factors:

Estimated Reference Def = invogit(logit(Alternative Def) – $\text{Beta}_{\text{Alternative Def}}$ – Beta_{Sex} * Sex – Beta $_{\text{Age scaled}}$ * Age Scaled)

Table 2a: MR-BRT Crosswalk Adjustment Factors for Ischaemic stroke

	Data input	Measure	Reference or alternative case definition	Gamma	Beta Coefficient, Logit (95% CI)
Ischaemic stroke	First-ever, subtype-specific, fatal and nonfatal events	Incidence	Ref	---	---
Ischaemic stroke	Hospital data	Incidence	Alt	0.97	-0.26 (-2.22 to 1.70)
Ischaemic stroke	Any stroke	Incidence	Alt		0.02 (-1.94 to 1.98)
Ischaemic stroke	Acute first-ever stroke	Incidence	Alt		0.22 (-1.67 to 2.12)
Ischaemic stroke	Inpatient clinical informatics	Incidence	Alt		0.70 (-1.26 to 2.66)
Ischaemic stroke	Sex (male)	Incidence	Alt		0.07 (-1.82 to 1.96)
Ischaemic stroke	Age scaled	Incidence	Alt		0.28 (-1.61 to 2.17)

Table 2b: MR-BRT Crosswalk Adjustment Factors for Intracerebral Haemorrhage

	Data input	Measure	Reference or alternative case definition	Gamma	Beta Coefficient, Logit (95% CI)
Intracerebral Haemorrhage	First-ever, subtype-specific, fatal and nonfatal events	Incidence	Ref	---	---
Intracerebral Haemorrhage	Hospital data	Incidence	Alt	0.50	0.04 (-0.93 to 1.02)
Intracerebral Haemorrhage	Any stroke	Incidence	Alt		1.78 (0.80 to 2.76)
Intracerebral Haemorrhage	Acute first-ever stroke	Incidence	Alt		0.15 (-0.83 to 1.13)
Intracerebral Haemorrhage	Inpatient clinical informatics	Incidence	Alt		1.40 (0.41 to 2.38)
Intracerebral Haemorrhage	Age scaled	Incidence	Alt		0.09 (-0.88 to 1.07)
Intracerebral Haemorrhage	Sex (male)	Incidence	Alt		0.10 (-0.88 to 1.06)

Table 2c: MR-BRT Crosswalk Adjustment Factors for Subarachnoid Haemorrhage

	Data input	Measure	Reference or alternative case definition	Gamma	Beta Coefficient, Logit (95% CI)
Subarachnoid Haemorrhage	First-ever, subtype-specific, fatal and nonfatal events	Incidence	Ref	---	---
Subarachnoid Haemorrhage	Aneurysmal subarachnoid haemorrhage only	Incidence	Alt	0.76	-0.79 (-2.28 to 0.70)
Subarachnoid Haemorrhage	Age scaled	Incidence	Alt		-0.11 (-1.59 to 1.38)
Subarachnoid Haemorrhage	Sex (male)	Incidence	Alt		-0.07 (-1.56 to 1.42)

1.3 Severity split inputs

The table below illustrates the severity level, lay description, and disability weights for GBD 2019. In previous iterations of GBD, severity splits for stroke were based on the standard approach described elsewhere (3). For GBD 2016, we undertook a review to identify epidemiologic literature which reported the degree of disability at

28 days (for acute stroke) or one year (for chronic stroke) using the modified Rankin scale (mRS) and the Mini-Mental State Examination (MMSE) or the Montreal Cognitive Assessment (MoCA). The mRS assesses functional capabilities, while the MMSE and MoCA tests provide evaluations of cognitive functioning. We then mapped these measures to the existing GBD categories as indicated below. This approach allowed us to include location-specific information and can be updated as more data on functional or cognitive status become available.

1.3.1 Acute stroke severity splits

Table 3a. Severity distribution, details on the severity levels for Acute Stroke in GBD 2019 and the associated disability weight (DW) with that severity.

Severity level	Lay description	Modified Rankin score	Cognitive status	DW (95% CI)
Stroke, mild	Has some difficulty in moving around and some weakness in one hand, but is able to walk without help.	1	N/A	0.019 (0.01–0.032)
Stroke, moderate	Has some difficulty in moving around, and in using the hands for lifting and holding things, dressing, and grooming.	2, 3	MoCA>=24 or MMSE>=26	0.07 (0.046–0.099)
Stroke, moderate plus cognition problems	Has some difficulty in moving around, in using the hands for lifting and holding things, dressing and grooming, and in speaking. The person is often forgetful and confused.	2, 3	MoCA<24 or MMSE<26	0.316 (0.206–0.437)
Stroke, severe	Is confined to bed or a wheelchair, has difficulty speaking, and depends on others for feeding, toileting, and dressing.	4, 5	MoCA>=24 or MMSE>=26	0.552 (0.377–0.707)
Stroke, severe plus cognition problems	Is confined to bed or a wheelchair, depends on others for feeding, toileting, and dressing, and has difficulty speaking, thinking clearly, and remembering things.		MoCA<24 or MMSE<26	0.588 (0.411–0.744)

1.3.2 Chronic stroke severity splits

Table 3b. Severity distribution, details on the severity levels for Chronic Stroke in GBD 2019 and the associated disability weight (DW) with that severity.

Severity level	Lay description	Modified Rankin score	Cognitive status	DW (95% CI)
Stroke, asymptomatic		0	N/A	N/A

Stroke, long-term consequences, mild	Has some difficulty in moving around and some weakness in one hand, but is able to walk without help.	1	N/A	0.019 (0.01–0.032)
Stroke, long-term consequences, moderate	Has some difficulty in moving around, and in using the hands for lifting and holding things, dressing, and grooming.	2, 3	MoCA>=24 or MMSE>=26	0.07 (0.046–0.099)
Stroke, long-term consequences, moderate plus cognition problems	Has some difficulty in moving around, in using the hands for lifting and holding things, dressing and grooming, and in speaking. The person is often forgetful and confused.	2, 3	MoCA<24 or MMSE<26	0.316 (0.206–0.437)
Stroke, long-term consequences, severe	Is confined to bed or a wheelchair, has difficulty speaking, and depends on others for feeding, toileting, and dressing.	4, 5	MoCA>=24 or MMSE>=26	0.552 (0.377–0.707)
Stroke, long-term consequences, severe plus cognition problems	Is confined to bed or a wheelchair, depends on others for feeding, toileting, and dressing, and has difficulty speaking, thinking clearly, and remembering things.	4, 5	MoCA<24 or MMSE<26	0.588 (0.411–0.744)

Table 4: Data input counts for the estimation process for the custom severity splits.

	Acute proportion	Chronic proportion
Site-years (total)	9	16
Number of countries with data	6	13
Number of GBD regions with data (out of 21 regions)	6	7
Number of GBD super-regions with data (out of 7 super-regions)	4	5

We used DisMod-MR, a Bayesian meta-regression tool, to model the six severity levels, with an independent proportion model for each. Reports which grouped mRS scores differently than our mapping (eg, 0-2) were adjusted in DisMod by estimating the association between these alternate groupings and our preferred mappings. These statistical associations were used to adjust data points to the referent category as necessary. The six models were scaled such that the sum of the proportions for all levels equaled 1.

1.4 Modelling strategy

The general approach employed for all of the components of the stroke modelling process is detailed in the table below.

- Data points were adjusted from alternative to reference case definitions using estimates from statistical models generated by MR-BRT (discussed elsewhere in the supplementary materials) for the acute models. Coefficients for these crosswalks can be found in Table 2a, 2b, and 2c.

- The GBD summary exposure values (SEV), which are the relative risk-weighted prevalence of exposure, were included as covariates for the ischaemic stroke or intracerebral haemorrhage models as appropriate, and a covariate for country income was used as a country-level covariate for both models (4). Subarachnoid haemorrhage did not include an SEV covariate but did include a covariate for country income for excess mortality. Coefficients for these covariates can be found in Table 5a, 5b, 5c for fixed effects located below.

- We used the ratio of acute:chronic cause-specific mortality estimated by the final GBD 2017 dismod model estimates to divide GBD 2019 stroke deaths into acute and chronic stroke deaths, using the global average for the proportion of acute:chronic stroke mortality. The acute and chronic models were then run using the same incidence, prevalence, and case fatality data as well as the custom cause-specific mortality rates as input data.

- We ran the first-ever acute subtype-specific models with CSMR as derived from FauxCorrect and epidemiological data as described above using Dismod-MR.

- We then calculated the rate of surviving until 28 days after an acute event for all three subtypes using the modelled estimates of excess mortality and incidence from the acute stroke models.

- Twenty-eight-day survivorship data was uploaded into the chronic subtype-specific with CSMR models. These chronic models also use CSMR as derived from FauxCorrect and epidemiological data as described above. Models were evaluated based on expert opinion, comparison with previous iterations, and model fit.

Table 5a, 5b, 5c below indicate the covariates used by cause in the estimation process, as well as the beta and exponentiated beta values.

Table 5a: Coefficients for covariates used in the acute and chronic ischemic stroke DisMod-MR models

Model	Variable name	Measure	beta	Exponentiated beta
First-ever acute ischaemic stroke with CSMR	Log-transformed age-standardised SEV scalar: Ischaemic stroke	Incidence	0.90 (0.85 to 0.95)	2.46 (2.34 to 2.58)
First-ever acute ischaemic stroke with CSMR	Healthcare access and quality index	Excess mortality rate	-0.035 (-0.035 to -0.035)	0.97 (0.97 to 0.97)
Chronic ischaemic stroke with CSMR	Log-transformed SEV scalar: Ischaemic stroke	Prevalence	0.85 (0.78 to 0.92)	2.34 (2.18 to 2.51)
Chronic ischaemic stroke with CSMR	LDI (I$ per capita)	Excess mortality rate	-0.41 (-0.46 to -0.36)	0.67 (0.63 to 0.70)

Table 5b: Coefficients for covariates used in the acute and chronic intracerebral haemorrhage DisMod-MR models

Model	Variable name	Measure	beta	Exponentiated beta

First-ever acute intracerebral haemorrhage with CSMR	Log-transformed SEV scalar: Intracerebral Haemorrhage	Incidence	0.76 (0.75 to 0.77)	2.13 (2.12 to 2.15)
First-ever acute intracerebral haemorrhage with CSMR	Healthcare access and quality index	Excess mortality rate	-0.07 (-0.07 to -0.069)	0.93 (0.93 to 0.93)
Chronic intracerebral haemorrhage with CSMR	Log-transformed SEV scalar: Intracerebral haemorrhage	Prevalence	0.75 (0.75 to 0.76)	2.12 (2.12 to 2.14)
Chronic intracerebral haemorrhage with CSMR	LDI (I$ per capita)	Excess mortality rate	-0.5 (-0.5 to -0.5)	0.61 (0.61 to 0.61)

Table 5a: Coefficients for covariates used in the acute and chronic subarachnoid DisMod-MR models

Model	Variable name	Measure	beta	Exponentiated beta
First-ever acute subarachnoid haemorrhage with CSMR	LDI (I$ per capita)	Excess mortality rate	-0.3 (-0.49 to -0.11)	0.74 (0.61 to 0.90)

1.5 Data sources and types

GBD 2019 synthesises a large and growing number of data input sources including surveys, censuses, vital statistics, and other health-related data sources. The data from these sources are used to estimate morbidity; illness, and injury; and attributable risk for 204 countries and territories from 1990 to 2019; mortality deaths are estimated from 1980 to 2019. The input sources are accessible through an interactive citation tool available in the GHDx. Citations for specific GBD components, causes and risks, and locations can be found through the Data Input Sources Tool in GHDx: http://ghdx.healthdata.org/gbd-2019/data-input-sources. This tool allows users to view and access GHDx records for input sources and export a comma-separated value (CSV) file that includes metadata, citations, and information about where the data were used in GBD. As required by GATHER, additional metadata for input sources are available through the citation tool as well.

The CoD database contains seven types of data sources (table S4): vital registration (VR), verbal autopsy (VA), cancer registry, police records, sibling history, surveillance, survey/census, and minimally invasive tissue sample (MITS) diagnoses. In countries with complete VR systems, there is no need to use any other data source. Less than half the world's population has deaths captured in a VR system, therefore, for countries with incomplete VR systems, vital statistics for causes of death may be supplemented with other data types.

All-cause mortality rates are estimated from vital registration data in countries with complete coverage. For other countries, the probabilities of death before age 5 and between ages 15 and 60 are estimated from censuses and surveys asking mothers to provide a history of children ever born and those still alive, and surveys asking adults about siblings who are alive or have passed away. Using model life tables, these probabilities of death are transformed into age-specific death rates by location, year, and sex. GBD has collated a large database of cause of death data from vital registrations and verbal autopsy surveys in which relatives are asked a standard set of questions to ascertain the likely cause of death, supplemented with police and mortuary data for injury deaths in countries with no other data. For countries with vital registration data, the completeness is assessed with demographic methods based on comparing recorded deaths with population counts between two successive censuses. The cause of death information is provided in a large number of different classification systems based on versions of the International Classification of Diseases or bespoke classifications in some countries. All data are mapped into the disease and injury categories of GBD. All classification systems contain codes that are less informative because they lack a specific diagnosis (eg, unspecified cancer) or refer to codes that cannot be underlying cause of death (eg, low back pain or senility) or are intermediate causes (eg, heart failure or sepsis). Such deaths are redistributed to more precise underlying causes of death.[4] After these redistributions and

corrections for under-registration, the data are analysed in CODEm (cause of death ensemble model), a highly systematised tool that runs many different models on the same data and chooses an ensemble of models that best reflects all the available input data. Models are chosen with variations in the statistical approach ("mixed effects" of spatiotemporal Gaussian Process Regression), in the unit of analysis (rates or cause fractions), and the choice of predictive covariates. The statistical performance of all models is tested by holding out 30% of the data and checking how well a model covers the data that were held out. To enforce consistency from CODEm, the sum of all cause-specific mortality rates is scaled to that of the all-cause mortality rates in each age, sex, location, and year category.

Non-fatal estimates are based on systematic reviews of published papers and unpublished documents, survey microdata, administrative records of health encounters, registries, and disease surveillance systems. Our Global Health Data Exchange (GHDx, http://ghdx.healthdata.org/) is the largest repository of health data globally. We first set a reference case definition and/or study method that best quantifies each disease or injury or consequence thereof. If there is evidence of a systematic bias in data that used different case definitions or methods compared to reference data, we adjust those data points to reflect what its value would have been if measured as the reference. This is a necessary step if one wants to use all data pertaining to a particular quantity of interest rather than choosing a small subset of data of the highest quality only. DisMod-MR 2.1, a Bayesian meta-regression tool, is our main method of analysing non-fatal data. It is designed as a geographical cascade where a first model is run on all the world's data, which produces an initial global fit and estimates coefficients for predictor variables and the adjustments for alternative study characteristics. The global fit adjusted by the values of random effects for each of seven GBD super-regions, the coefficients on sex and country predictors, are passed down as data to a model for each super-region together with the input data for that geography. The same steps are repeated going from super-region to 21 region fits and then to 195 fits by country and where applicable a further level down to subnational units. Below the global fit, all models are run separately by sex and for six time periods: 1990, 1995, 2000, 2005, 2010, and 2016. During each fit all data on prevalence, incidence, remission (ie, cure rate) and mortality are forced to be internally consistent. For most diseases, the bulk of data on prevalence or incidence is at the disease level with fewer studies providing data on the proportions of cases of disease in each of the sequelae defined for the disease. The proportions in each sequela are pooled using DisMod-MR 2.1 or meta-analysis or derived from analyses of patient-level datasets. The multiplication of prevalent cases for each disease sequela and the appropriate disability weight produces YLD estimates that do not yet take into account comorbidity. To correct for comorbidity, these data are used in a simulation to create hypothetical individuals in each age, sex, location, and year combination who experience no, one, or multiple sequelae simultaneously. We assume that disability weights are multiplicative rather than additive as this avoids assigning a combined disability weight value in any individual to exceed 1, ie, be worse than a "year lost due to death". This comorbidity adjustment leads to an average scaling down of disease- specific YLDs ranging from about 2% in young children up to 17% in oldest ages.

All our estimates of causes of death are categorical: each death is assigned to a single underlying cause. This has the attractive property that all estimates add to 100%. For risks, we use a different, "counterfactual" approach, ie, answering the question: "what would the burden have been if the population had been exposed to a theoretical minimum level of exposure to a risk". Thus, we need to define what level of exposure to a risk factor leads to the lowest amount of disease. We then analyse data on the prevalence of exposure to a risk and derive relative risks for any risk-outcome pair for which we find sufficient evidence of a causal relationship. Prevalence of exposure is estimated in DisMod-MR 2.1, using spatiotemporal Gaussian Process Regression, or from satellite imagery in the case of ambient air pollution. Relative risk data are pooled using meta-analysis of cohort, case-control and/or intervention studies. For each risk and outcome pair, we evaluate the evidence and judge if the evidence falls into the categories of "convincing" or "probable" as defined by the World Cancer Research Fund.[5] From the prevalence and relative risk results, population attributable fractions are estimated relative to the theoretical minimum risk exposure level (TMREL). When we aggregate estimates for clusters of risks, eg, metabolic or behavioural risks, we use a multiplicative function rather than simple addition and take into account how much of each risk is mediated through another risk. For instance, some of the risk of high body mass index is directly onto stroke as an outcome but much of its impact is mediated through high blood pressure, high

cholesterol, or high fasting plasma glucose, and we would not want to double count the mediated effects when we estimate aggregates across risk factors.

We included inpatient hospital data, adjusted for readmission and primary to any diagnosis using correction factors estimated from US claims data. We excluded data for locations where the data points were implausibly low (Vietnam, Philippines, India). In addition, we included unpublished stroke registry data for acute ischaemic and acute haemorrhagic strokes. We also included survey data for chronic cerebrovascular disease. These surveys were identified based on expert opinion and review of major survey series focused on world health that included questions regarding self-reported history of stroke.

As with many models in GBD, the diversity of data sources available means that we needed to adjust available data to our preferred or reference case definition (2). For the first ever acute stroke models we used DisMod to estimate the statistical association between measurements taken using different case definitions and then used these estimates to adjust the non-referent datapoints. We included study-level covariates to adjust data points for first and recurrent strokes combined, using data for first strokes only as reference. We also included study-level covariates to adjust ischaemic and haemorrhagic strokes combined (all stroke), using as reference studies with subtype-specific information.

Uncertainty is propagated throughout all these calculations by creating 1,000 values for each prevalence, death, YLL, YLD, or DALY estimate and performing aggregations across causes and locations at the level of each of the 1,000 values for all intermediate steps in the calculation. The lower and upper bounds of the 95% uncertainty interval are the 25th and 975th values of the ordered 1,000 values. For all age-standardised rates, GBD uses a standard population calculated as the non-weighted average across all countries of the percentage of the population in each five-year age group for the years 2010 to 2035 from the United Nations Population Division's World Population Prospects (2012 revision).

1.6 Stroke definition

Stroke was defined according to WHO criteria – rapidly developing clinical signs of focal (at times global) disturbance of cerebral function lasting more than 24 hours or leading to death with no apparent cause other than that of vascular origin.[9] Data on transient ischaemic attack (TIA) were not included.
Acute stroke: Stroke cases are considered acute from the day of incidence of a first-ever stroke through day 28 following the event.
Chronic stroke: Stroke cases are considered chronic beginning 28 days following the occurrence of an event. Chronic stroke includes the sequelae of an acute stroke AND all recurrent stroke events. GBD 2019 adopts this broader definition of chronic stroke than was used in prior iterations in order to model acute strokes using only first-ever incident events.
Ischaemic stroke: Incident ischaemic stroke is defined as the occurrence of first-ever ischaemic stroke, based on clinical diagnosis by a physician using diagnostic imaging. Ischaemic strokes are considered to include all vascular events leading to limited blood flow to brain tissue, with resulting infarction, including atherosclerotic and thromboembolic strokes but excluding strokes in which the underlying cause is intracranial haemorrhage.
Haemorrhagic or other strokes: This cause includes all non-ischaemic strokes of a vascular cause including subarachnoid and stroke due to intracranial haemorrhage. Intracerebral haemorrhage (ICH) was defined as stroke with a focal collection of blood in the brain not due to trauma. Subarachnoid haemorrhage (SAH) was defined as non-traumatic stroke due to bleeding into the subarachnoid space of the brain. ICD codes used for inclusion of hospital and claims data can be found in Supplement Table 2.

1.7 Modelling strategy

In GBD 2019, stroke incidence is reported as the instantaneous hazard of first-ever stroke while deaths are per capita and per year, with the employment of two separate models, an "acute" model of 30 day mortality and a "chronic" model of survivors beyond 30 days, based on the preponderance of available data. Thus, the incidence rate represents new events in a given year while the death rate represents those that occurred in that year

regardless of when the stroke occurred. Therefore, while uncommon, in some cases death rates can be equal or even greater than incidence rates. Imprecision in this particular ratio of acute to long-term survival (which is estimated separately by year, age, sex, and location) will impact the relationship between incidence and deaths in a given year. There is limited literature on the proportion of deaths occurring within 30-days vs 1-year or longer that would be a perfect comparison with GBD, but some studies we have seen suggest that our estimated proportions may be too low, especially for older ages.[10-12] We intend to reassess this for GBD 2021.

For GBD 2019, we used a standard CODEm approach to model deaths from stroke. The covariates included in the ensemble modelling process are listed in the table below. For GBD 2019, adjusted dietary covariates for consumption of fruits, omega-3 fatty acids, vegetables, nuts and seeds, and polyunsaturated fatty acids (PUFA) were replaced with the summary exposure value scalars for diet low in each of these factors. The direction for each dietary covariate was changed from -1 to 1 to as our a priori assumption is that low levels of intake of these dietary factors are associated with increasing mortality risk from stroke. We dropped the dietary covariate for whole grains (kcal/capita, adjusted) and the socio-demographic index covariate as exploratory analyses indicated that these variables were not predictive of stroke mortality. In addition, we changed the direction of the alcohol consumption covariate from 0 to 1 to reflect the expected direction of the association for this risk factor with stroke mortality. Apart from these covariate changes, there are no substantive changes from the approach used in GBD 2017.

Table: Selected covariates for CODEm models, stroke

Covariate	Transformation	Level	Direction
Summary exposure variable, stroke	None	1	1
Cholesterol (total, mean per capita)	None	1	1
Smoking prevalence	None	1	1
Systolic blood pressure (mmHg)	None	1	1
Mean BMI	None	2	1
Elevation over 1,500m (proportion)	None	2	-1
Fasting plasma glucose	None	2	1
Outdoor pollution ($PM_{2.5}$)	None	2	1
Indoor air pollution	None	2	1
Healthcare Access and Quality Index	None	2	-1
Lag distributed income per capita (I$)	Log	3	-1
Summary exposure value, omega-3	None	3	1
Summary exposure value, fruits	None	3	1
Summary exposure value, vegetables	None	3	1
Summary exposure value, nuts and seeds	None	3	1
Pulses/legumes (kcal/capita, unadjusted)	None	3	-1
Summary exposure value, PUFA adjusted (percent)	None	3	1
Alcohol (litres per capita)	None	3	1
Trans fatty acid	None	3	1

1.7.1 Ischaemic stroke

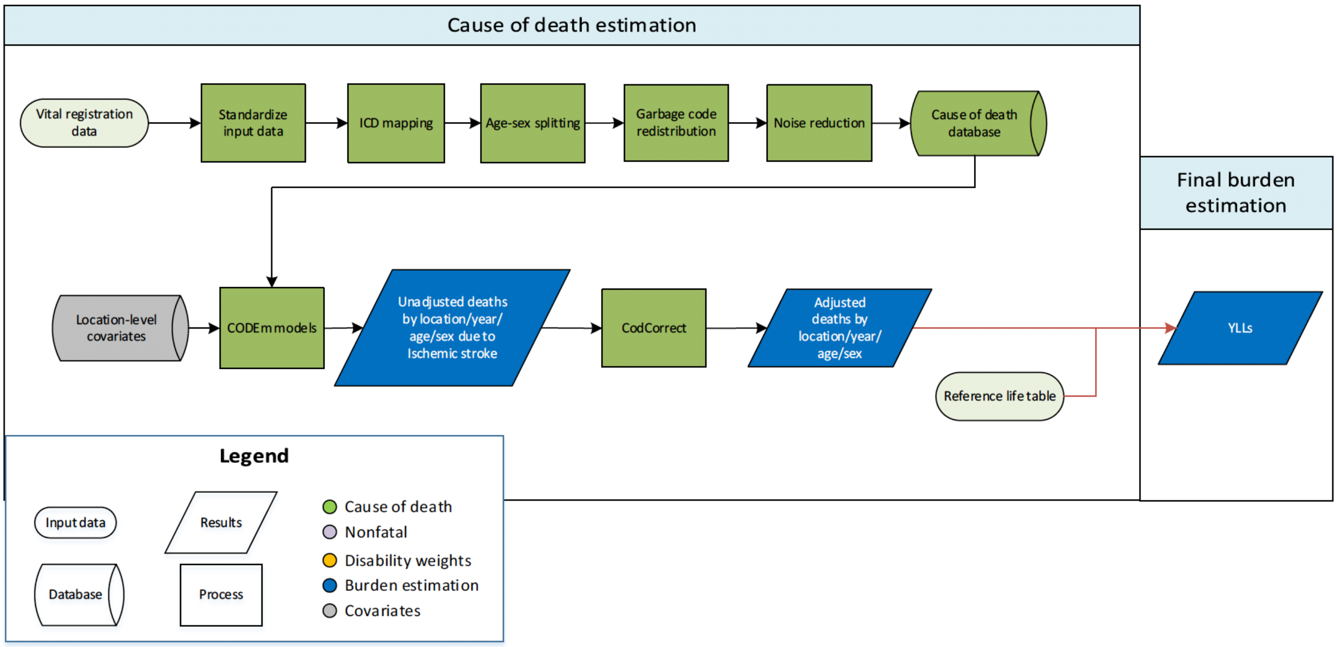

1.7.1.1 Input data

Vital registration data were used to model deaths from ischaemic stroke. We outliered ICD8 data points which were inconsistent with the rest of the data and created implausible time trends. We also outliered ICD10 data points in The Republic of Tajikistan due to unstable and implausible estimates in similar age groups.

1.7.1.2 Modelling strategy

We used a standard CODEm approach to model deaths from ischemic stroke. For GBD 2019, adjusted dietary covariates for consumption of fruits, omega-3 fatty acids, vegetables, nuts and seeds, and polyunsaturated fatty acids were replaced with the summary exposure value scalars for diet low in each of these factors. The direction for each dietary covariate was changed from -1 to 1 to as our *a priori* assumption is that low levels of intake of these dietary factors are associated with increasing mortality risk from ischaemic stroke. In addition, the dietary covariate for whole grains (kcal/capita, adjusted) and the socio-demographic index covariate were dropped as exploratory analyses indicated that the covariates were not predictive of the outcome. In addition, we changed the direction of the alcohol variable from 0 to 1 to reflect our *a priori* hypothesis about the expected direction of the association between this risk factor and mortality risk of ischaemic stroke. We also changed the level of the trans fatty acid covariate from 1 to 3. Besides these covariate changes, there are no other substantive changes from the approach used in GBD 2017.

Table: Selected covariates for CODEm models, ischaemic stroke

Covariate	Transformation	Level	Direction
Summary exposure value, ischaemic stroke	None	1	1
Cholesterol (total, mean per capita)	None	1	1
Smoking prevalence	None	1	1
Systolic blood pressure (mmHg)	None	1	1
Mean BMI	None	2	1
Elevation over 1500m (proportion)	None	2	-1
Fasting plasma glucose	None	2	1
Outdoor pollution ($PM_{2.5}$)	None	2	1
Indoor air pollution	None	2	1

Healthcare access and quality index	None	2	-1
Lag distributed income per capita (I$)	Log	3	-1
Summary exposure value, omega-3	None	3	1
Summary exposure value, fruits	None	3	1
Summary exposure value, vegetables	None	3	1
Summary exposure value, nuts and seeds	None	3	1
Pulses/legumes (kcal/capita, unadjusted)	None	3	-1
Summary exposure value PUFA adjusted	None	3	1
Alcohol (litres per capita)	None	3	1
Trans fatty acid	None	3	1

1.7.2 Intracerebral haemorrhage

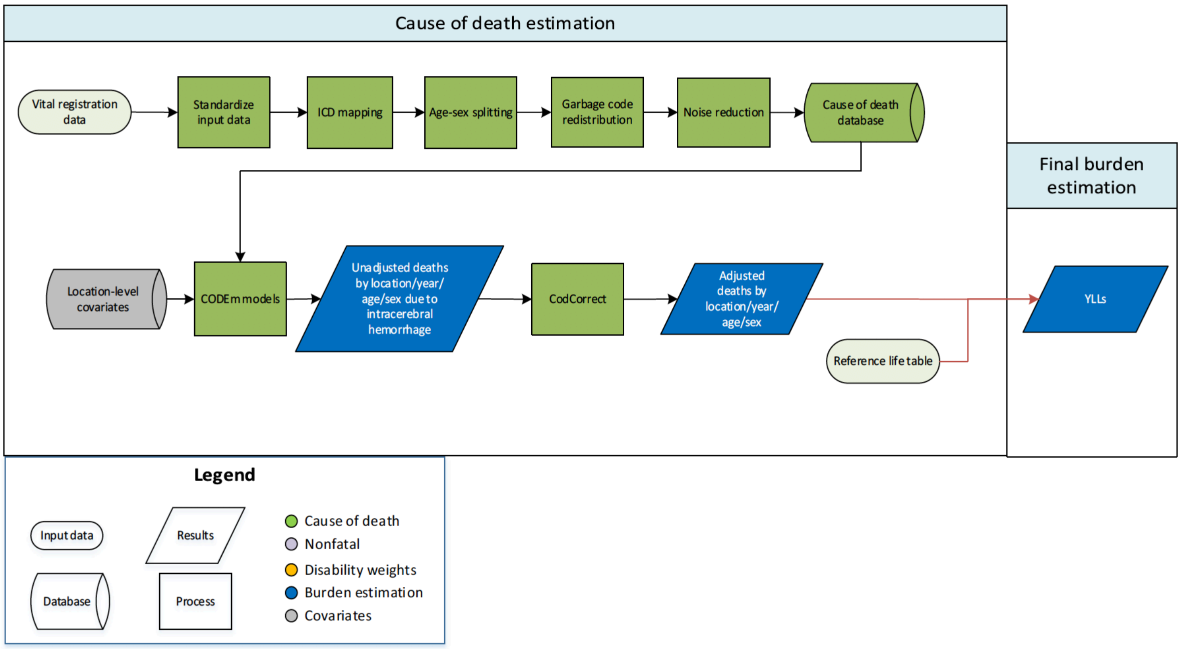

1.7.2.1 Input data

Vital registration data were used to model intracerebral haemorrhage. We outliered ICD8 data points which were inconsistent with the rest of the data and created implausible time trends. In addition, we outliered vital registration data points in certain countries in Latin American countries due to implausibly high values at the oldest age groups resulting in inconsistencies in time trends.

1.7.2.2 Modelling strategy

We used a standard CODEm approach to model deaths from intracerebral haemorrhage. For GBD 2019, adjusted dietary covariates for consumption of fruits, omega-3 fatty acids, vegetables, nuts and seeds, and polyunsaturated fatty acids were replaced with the summary exposure value scalars for diet low in each of these factors. The direction for each dietary covariate was changed from -1 to 1 to as our *a priori* assumption is that low levels of intake of these dietary factors are associated with increasing mortality risk from intracerebral haemorrhage. In addition, the dietary covariate for whole grains (kcal/capita, adjusted) and the social demographic index covariate were dropped as exploratory analyses indicated that these covariates were not predictive of the mortality risk from intracerebral haemorrhage. We changed the direction of the covariate for alcohol from 0 to 1 due to our *a priori* hypothesis about the direction of the association for this covariate. We also changed the level of the cholesterol covariate from 1 to 3 and the direction from 0 to -1 to reflect the mixed and inconclusive evidence regarding cholesterol levels and risk of intracerebral haemorrhage. In addition, we

changed the level of the trans fatty acid from covariate from 1 to 3 in accordance with the expected importance of this risk factor on mortality from intracerebral haemorrhage. Besides these covariate changes, there are no other substantive changes from the approach used in GBD 2017.

1.7.3 Subarachnoid haemorrhage

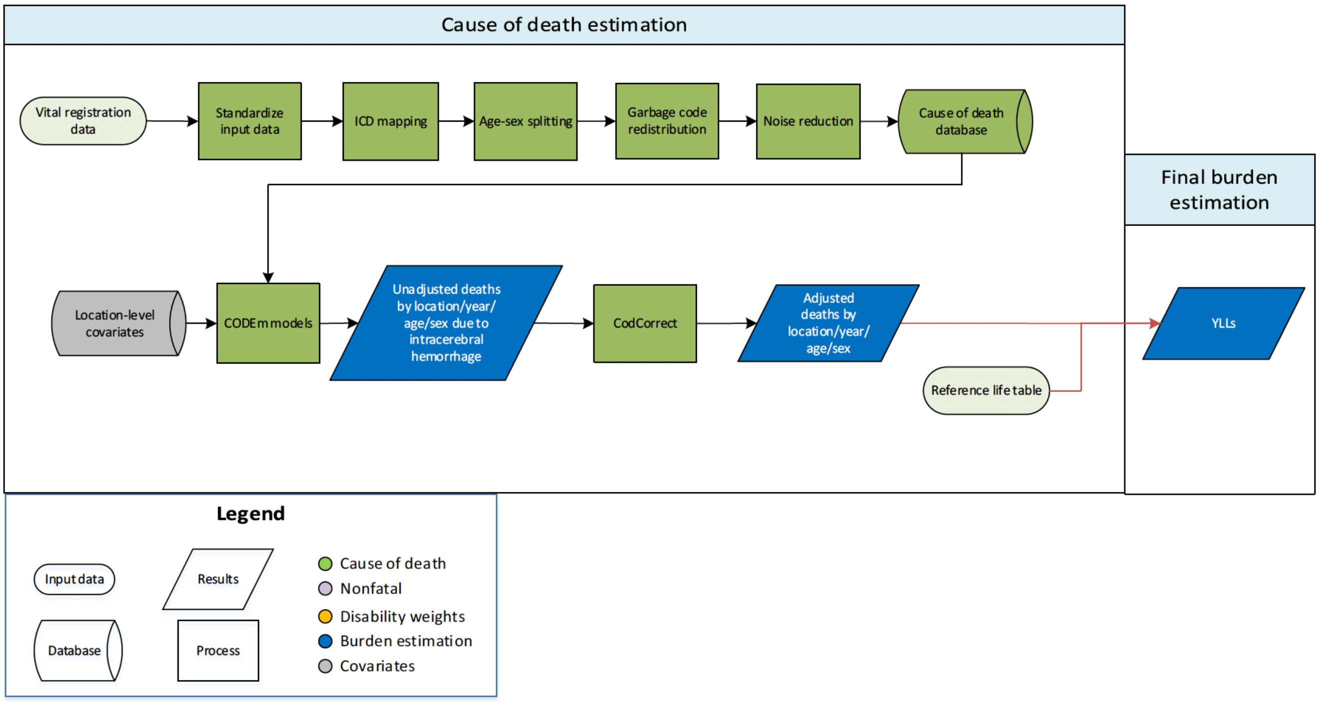

1.7.3.1 Input data

Vital registration data were used to model subarachnoid haemorrhage. We outliered ICD8 datapoints which were inconsistent with the rest of the data and created implausible time trends. In addition, we outliered vital registration data in Tibet that was implausibly high for all years and age groups.

1.7.3.2 Modelling strategy

We used a standard CODEm approach to model deaths from subarachnoid haemorrhage. The covariates chosen for inclusion in the ensemble modelling process are listed in the table below. For GBD 2019, we dropped the Socio-demographic Index covariate as exploratory analyses indicated that it was not predictive of the outcome. We also changed the direction of the alcohol covariate from 0 to 1 to reflect the expected direction of the association of this risk factor with mortality risk. Apart from these changes to the covariates, there are no substantive changes from the approach used in GBD 2017.

Table: Selected covariates for CODEm models, subarachnoid haemorrhage

Level	Covariate	Transformation	Direction
1	Smoking prevalence	None	1
1	Systolic blood pressure (mmHg)	None	1
2	Healthcare access and quality index	None	-1
3	Lag distributed income per capita (I$)	Log	-1
3	Alcohol (litres per capita)	None	1

1.8 Methods tables

1.8.1 Table 1. ICD Codes used in fatal and non-fatal analysis

Fatal analysis	ICD10	ICD9
Cerebrovascular disease	G45-G46.8, I60-I63.9, I65-I66.9, I67.0-I67.3, I67.5-I67.6, I68.1-I68.2, I69.0-I69.3	430-435.9, 437.0-437.2, 437.5-437.8
Ischaemic stroke	G45-G46.8, I63-I63.9, I65-I66.9, I67.2-I67.3, I67.5-I67.6, I69.3	433-435.9, 437.0-437.1, 437.5-437.8
Haemorrhagic stroke	I60-I62.9, I67.0-I67.1, I68.1- I68.2, I69.0-I69.2	430-432.9, 437.2

Nonfatal analysis	ICD10	ICD9
Cerebrovascular disease	I60-I63.9, I65-I66.9, I67.0-I67.3, I67.5-I67.6, I68.1-I68.2, I69.0- I69.3	430-434.9, 437.0-437.2, 437.5-437.8
Ischaemic stroke	I63-I63.9, I65-I66.9, I67.2-I67.3, I67.5-I67.6, I69.3	433-434.9, 437.0-437.1, 437.5-437.8
Haemorrhagic stroke	I60-I62.9, I67.0-I67.1, I68.1- I68.2, I69.0-I69.2	430-432.9, 437.2

1.8.2 Table 2. Selected covariates for CODEm models, overall stroke and subtypes

CODEm is an analytical tool which explores a large variety of possible models to estimates trends in causes of death. This tool explores a large variety of possible models to estimate trends in causes of death. Possible models are identified using a covariate selection algorithm that yields many plausible combinations of covariates, which are then run through four model classes. The model classes include mixed effects linear models and spatial-temporal Gaussian Process Regression models for cause fractions and death rates. All models for each cause of death are then assessed using out-of-sample predictive validity and combined into an ensemble with optimal out-of-sample predictive performance. Ensemble models for cause of death estimation outperform any single component model in tests of root mean square error, frequency of predicting correct temporal trends, and achieving 95% coverage of the prediction interval.[13]

Covariate	Level	Direction, Stroke	Direction, Ischaemic stroke	Direction, Haemorrhagic stroke
Summary exposure variable	1	+	+	+
Cholesterol (total, mean per capita)	1	+	+	0
Smoking prevalence	1	+	+	+
Systolic blood pressure (mmHg)	1	+	+	+
Trans fatty acid	1	+	+	+
Mean BMI	2	+	+	+

Elevation over 1500m (proportion)	2	-	-	-
Fasting plasma glucose	2	+	+	+
Outdoor pollution ($PM_{2.5}$)	2	+	+	+
Indoor air pollution	2	+	+	+
Healthcare access and quality index	2	-	-	-
Lag distributed income per capita (I$)*	3	-	-	-
Socio-demographic Index	3	0	0	0
Omega-3 (kcal/capita, adjusted) *	3	-	-	-
Fruits (kcal/capita, adjusted)	3	-	-	-
Vegetables (kcal/capita, adjusted)	3	-	-	-
Nuts and seeds (kcal/capita, adjusted)	3	-	-	-
Whole grains (kcal/capita, adjusted)	3	-	-	-
Pulses/legumes (kcal/capita, adjusted)	3	-	-	-
PUFA adjusted (percent)	3	-	-	-
Alcohol (litres per capita)	3	0	0	0

*Variables were log-transformed

1.8.3 Table 3. Counts of data used by measure and stroke model for GBD 2019

First-ever acute ischaemic stroke:

Region Name	Incidence	Prevalence	Remission	Mortality	Hospital Claims
East Asia	5	0	0	0	1
Southeast Asia	0	0	0	0	1
Oceania	0	0	0	0	0
Central Asia	1	0	0	0	1
Central Europe	3	0	0	2	14
Eastern Europe	5	0	0	1	5
High-income Asia Pacific	4	0	0	0	0
Australasia	8	0	0	0	4
Western Europe	27	0	0	6	31
Southern Latin America	2	0	0	1	0
High-income North America	1	0	0	0	5
Caribbean	0	0	0	0	0
Andean Latin America	0	0	0	0	0
Central Latin America	0	0	0	0	0
Tropical Latin America	2	0	0	0	0
North Africa and Middle East	9	0	0	7	2
South Asia	4	0	0	4	0
Central Sub-Saharan Africa	0	0	0	0	0

Eastern Sub-Saharan Africa	1	0	0	0	0
Southern Sub-Saharan Africa	1	0	0	0	0
Western Sub-Saharan Africa	0	0	0	0	0
Total	73	0	0	21	64

First-ever acute haemorrhagic stroke:

Region Name	Incidence	Prevalence	Remission	Mortality	Hospital Claims
East Asia	5	0	0	0	1
Southeast Asia	0	0	0	0	1
Oceania	0	0	0	0	0
Central Asia	1	0	0	1	1
Central Europe	3	0	0	1	14
Eastern Europe	5	0	0	2	5
High-income Asia Pacific	3	0	0	0	0
Australasia	9	0	0	3	4
Western Europe	29	0	0	17	31
Southern Latin America	2	0	0	2	0
High-income North America	2	0	0	0	5
Caribbean	0	0	0	0	0
Andean Latin America	0	0	0	0	0
Central Latin America	0	0	0	0	0
Tropical Latin America	0	0	0	0	0
North Africa and Middle East	8	0	0	13	2
South Asia	4	0	0	6	0
Central Sub-Saharan Africa	0	0	0	0	0
Eastern Sub-Saharan Africa	1	0	0	1	0
Southern Sub-Saharan Africa	1	0	0	0	0
Western Sub-Saharan Africa	0	0	0	0	0
Total	73	0	0	46	64

Chronic ischaemic stroke:

Region Name	Incidence	Prevalence	Remission	Mortality	Hospital Claims
East Asia	0	4	0	0	0
Southeast Asia	0	3	0	0	0
Oceania	0	0	0	0	0
Central Asia	0	0	0	0	0
Central Europe	0	0	0	1	0
Eastern Europe	0	0	0	0	0
High-income Asia Pacific	0	2	0	0	0
Australasia	0	1	0	2	0
Western Europe	0	42	0	7	0
Southern Latin America	0	1	0	0	0
High-income North America	0	39	0	0	0
Caribbean	0	2	0	0	0

Andean Latin America	0	1	0	0	0
Central Latin America	0	4	0	0	0
Tropical Latin America	0	1	0	0	0
North Africa and Middle East	0	2	0	0	0
South Asia	0	11	0	0	0
Central Sub-Saharan Africa	0	0	0	0	0
Eastern Sub-Saharan Africa	0	2	0	0	0
Southern Sub-Saharan Africa	0	0	0	0	0
Western Sub-Saharan Africa	0	4	0	0	0
Total	0	119	0	10	0

Chronic haemorrhagic stroke:

Region Name	Incidence	Prevalence	Remission	Mortality	Hospital Claims
East Asia	0	4	0	0	0
Southeast Asia	0	3	0	0	0
Oceania	0	0	0	0	0
Central Asia	0	0	0	0	0
Central Europe	0	0	0	1	0
Eastern Europe	0	0	0	0	0
High-income Asia Pacific	0	2	0	0	0
Australasia	0	1	0	2	0
Western Europe	0	42	0	7	0
Southern Latin America	0	1	0	0	0
High-income North America	0	39	0	0	0
Caribbean	0	2	0	0	0
Andean Latin America	0	1	0	0	0
Central Latin America	0	4	0	0	0
Tropical Latin America	0	1	0	0	0
North Africa and Middle East	0	2	0	0	0
South Asia	0	10	0	0	0
Central Sub-Saharan Africa	0	0	0	0	0
Eastern Sub-Saharan Africa	0	2	0	0	0
Southern Sub-Saharan Africa	0	0	0	0	0
Western Sub-Saharan Africa	0	4	0	0	0
Total	0	118	0	10	0

1.8.4 Table 4. DisMod covariates

Step 1:

Cause	Variable name	Measure	beta	Exponentiated beta
Chronic ischaemic stroke	Log-transformed SEV scalar: Isch Stroke	Prevalence	0.83 (0.75 — 1.03)	2.29 (2.12 — 2.80)

Chronic ischaemic stroke	LDI (I$ per capita)	Excess mortality rate	-0.16 (-0.29 — -0.1)	0.85 (0.75 — 0.90)
Chronic haemorrhagic stroke	Log-transformed SEV scalar: Hem Stroke	Prevalence	0.79 (0.75 — 0.92)	2.21 (2.12 — 2.50)
Chronic haemorrhagic stroke	LDI (I$ per capita)	Excess mortality rate	-0.12 (-0.16 — -0.1)	0.89 (0.85 — 0.90)
First ever acute haemorrhagic stroke	Hospital data	Incidence	0.54 (0.54 – 0.54)	1.71 (1.71 – 1.72)
First ever acute haemorrhagic stroke	Any stroke	Incidence	1.27 (1.27 – 1.28)	3.57 (3.56 – 3.59)
First ever acute haemorrhagic stroke	First-ever acute stroke, ischaemic or hemorrhagic	Incidence	0.52 (0.52 – 0.53)	1.69 (1.68 – 1.71)
First ever acute haemorrhagic stroke	Log-transformed age-standardized SEV scalar: hemorrhagic stroke	Incidence	0.77 (0.75 – 0.82)	2.17 (2.12 – 2.27)
First ever acute haemorrhagic stroke	Any stroke	Excess mortality rate	-0.48 (-0.66 – -0.32)	0.62 (0.52 – 0.73)
First ever acute haemorrhagic stroke	First-ever acute stroke, ischaemic or hemorrhagic	Excess mortality rate	-0.081 (-0.3 – 0.16)	0.62 (0.52 – 0.73)
First ever acute ischaemic stroke	Hospital data	Incidence	0.38 (0.37 – 0.38)	1.46 (1.45 – 1.46)
First ever acute ischaemic stroke	Any stroke	Incidence	0.31 (0.29 – 0.33)	1.37 (1.34 – 1.39)
First ever acute ischaemic stroke	First-ever acute stroke, ischaemic or hemorrhagic	Incidence	0.37 (0.36 – 0.38)	1.44 (1.43 – 1.46)
First ever acute ischaemic stroke	Log-transformed age-standardized SEV scalar: ischaemic stroke	Incidence	1.16 (1.09 – 1.22)	3.21 (2.99 – 3.39)

Step 2:

Cause	Variable name	Measure	beta	Exponentiated beta
Chronic ischaemic stroke with CSMR	Log-transformed SEV scalar: Ischaemic stroke	Prevalence	0.89 (0.75 – 1.19)	2.44 (2.13 – 3.27)
Chronic ischaemic stroke with CSMR	LDI (I$ per capita)	Excess mortality rate	-0.49 (-0.5 – -0.46)	0.61 (0.61 – 0.63)
Chronic haemorrhagic stroke with CSMR	Log-transformed SEV scalar: Haemorrhagic stroke	Prevalence	0.88 (0.75 – 1.15)	2.40 (2.13 – 3.17)
Chronic haemorrhagic stroke with CSMR	LDI (I$ per capita)	Excess mortality rate	-0.48 (-0.5 – -0.44)	0.62 (0.61 – 0.64)

First-ever acute haemorrhagic stroke with CSMR	Any stroke	Incidence	1.27 (1.27 – 1.29)	3.58 (3.56 – 3.62)
First-ever acute haemorrhagic stroke with CSMR	First-ever acute stroke, ischaemic or hemorrhagic	Incidence	0.52 (0.52 – 0.54)	1.69 (1.68 – 1.71)
First-ever acute haemorrhagic stroke with CSMR	Log-transformed SEV scalar: Hem stroke	Incidence	1.11 (1.01 – 1.20)	3.03 (2.74 – 3.33)
First-ever acute haemorrhagic stroke with CSMR	Any stroke	Excess mortality rate	-0.37 (-0.49 – -0.27)	0.69 (0.62 – 0.77)
First-ever acute haemorrhagic stroke with CSMR	First-ever acute stroke, ischaemic or hemorrhagic	Excess mortality rate	0.023 (-0.2 – 0.23)	1.02 (0.82 – 1.25)
First-ever acute ischaemic stroke with CSMR	Any stroke	Incidence	0.32 (0.30 – 0.33)	1.38 (1.35 – 1.39)
First-ever acute ischaemic stroke with CSMR	First-ever acute stroke, ischaemic or hemorrhagic	Incidence	0.37 (0.36 – 0.38)	1.44 (1.43 – 1.46)
First-ever acute ischaemic stroke with CSMR	Log-transformed age-standardized SEV scalar: Ischaemic stroke	Incidence	1.11 (1.05 – 1.18)	3.04 (2.86 – 3.26)
First-ever acute ischaemic stroke with CSMR	Any stroke	Excess mortality rate	-0.34 (-0.45 – -0.24)	0.71 (0.64 – 0.79)
First-ever acute ischaemic stroke with CSMR	First-ever acute stroke, ischaemic or hemorrhagic	Excess mortality rate	-0.69 (-0.82 – -0.56)	0.51 (0.44 – 0.57)

1.8.5 Table 5. Sequelae and disability weights for ischaemic stroke, intracerebral haemorrhage and subarachnoid haemorrhage

Sequela	Health state lay description	Disability weight
Asymptomatic chronic stroke		N/A
Acute and chronic stroke, severity level 1	Has some difficulty in moving around and some weakness in one hand, but is able to walk without help	0.019 (0.01 – 0.032)
Acute and chronic stroke, severity level 2	Has some difficulty in moving around and in using the hands for lifting and holding things, dressing and grooming	0.07 (0.046 – 0.099)
Acute and chronic stroke, severity level 3	Has some difficulty in moving around, in using the hands for lifting and holding things, dressing and grooming, and in speaking. The person is often forgetful and confused.	0.316 (0.205 – 0.438)

Acute and chronic stroke, severity level 4	Is confined to bed or a wheelchair, has difficulty speaking and depends on others for feeding, toileting and dressing.	0.552 (0.376 – 0.707)
Acute and chronic stroke, severity level 5	Is confined to bed or a wheelchair, depends on others for feeding, toileting and dressing, and has difficulty speaking, thinking clearly and remembering things.	0.588 (0.411 – 0.745)

Section 2. Summary of GBD methods for estimates of burden of risk factors

A complete set of risk-specific exposures, relative risks (RRs), theoretical minimum-risk exposure levels (TMRELs), and population attributable fractions (PAFs) were computed for the years 1990–2019. The current version of the data download tool is available in the GHDx and contains core summary results for the GBD 2019: http://ghdx.healthdata.org/gbd-results-tool. The core summary results include deaths, years of life lost (YLLs), years lived with disability (YLDs), disability-adjusted life-years (DALYs), and summary exposure values (SEVs). The GHDx includes data for causes, risks, cause-risk attribution, aetiologies, and impairments.

GBD 2019 incorporated a large number and wide variety of input sources to estimate mortality, causes of death and illness, and risk factors for 204 countries and territories from 1990-2019. These input sources are accessible through an interactive citation tool available in the GHDx.

Users can retrieve citations for a specific GBD component, cause or risk, and location by choosing from the available selection boxes. They can then view and access GHDx records for input sources and export a comma-separated value (CSV) file that includes the GHDx metadata, citations, and information about where the data were used in GBD. Additional metadata for each input source are available through the citation tool as required by the GATHER statement. The citation tool is accessible through the GHDx at http://ghdx.healthdata.org/gbd-2019/data-input- sources.

We also assessed the following three clusters of risks: behavioural (smoking [including second-hand smoking], dietary [diet high in sodium, diet high in red meat, diet low in fruits, diet low in vegetables, diet low in whole grains, and alcohol use], low physical activity), environmental and occupational (ambient particulate matter [$PM_{2.5}$] pollution, household air pollution, suboptimal temperature, and lead exposure), and metabolic (high BMI, high fasting plasma glucose, high SBP, high LDL cholesterol, kidney dysfunction). Tobacco smoke (smoking and second-hand smoking) was considered as a separate cluster but was also included in the behavioural cluster.

2.1 Definition of risk factors and their estimation

As explained in detail elsewhere,[2, 7] ambient $PM_{2.5}$ pollution was defined as an annual average daily exposure to outdoor air concentrations of $PM_{2.5}$ more than 8.8 μg/m^3. Household air pollution was defined as any exposure to indoor concentration of $PM_{2.5}$. Lead exposure was defined as blood lead concentration of more than 20 μg/L. Diet high in sodium was defined as consumption of sodium more than 5 g/day. Diet low in fruits was defined as consumption of less than 200 g/day. Diet low in vegetables was defined as consumption of less than 350 g/day. Diet low in whole grains was defined as consumption of less than 100 g/day. Alcohol use was defined as any amount. Low physical activity was defined as average weekly work, home, transport-related, and recreational physical activity of less than 8000 metabolic equivalent of task-min. Smoking was defined as any previous or current tobacco smoking. Second-hand smoking was defined as daily indoor exposure to second-hand smoking. High body-mass index (BMI) was defined as body-mass index greater than 23.0 kg/m^2. High fasting plasma glucose was defined as serum fasting plasma glucose greater than 5.4 mmol/L. High systolic blood pressure (SBP) was defined as systolic blood pressure greater than 110-115 mm Hg. High (low density lipoprotein) LDL cholesterol was estimated in units of mmol/L (we used a TMREL with a uniform distribution between 0·7 and 1·3

mmol/L). Low glomerular filtration rate was defined as glomerular filtration rate less than 60 mL/min per $1.73m^2$ (excluding end-stage renal disease). Theoretical minimum risk exposure levels were described elsewhere.[7, 14] The GBD risk factor list continues to evolve to reflect the policy relevance, public health, and medical care importance of major risk factors. Three risks were added to the list for GBD 2019: non-optimal temperature, high temperature, and low temperature.

GBD 19 analytical method for estimating PAF has six analytical steps. (1) We included 19 risk–outcome pairs that met criteria for convincing or probable evidence on the basis of research studies. (2) Relative risks were estimated as a function of exposure based on published systematic reviews, GBD review and meta-regression. (3) Levels of exposure in each age-sex-location-year included in the study were estimated based on all available data sources using spatiotemporal Gaussian process regression, DisMod-MR 2.1, a Bayesian meta-regression method, or alternative methods. (4) We determined, from published trials or cohort studies, the level of exposure associated with minimum risk, called the theoretical minimum risk exposure level. (5) Attributable DALYs were computed by multiplying PAFs by the relevant outcome quantity for each age sex-location-year. (6) PAFs and attributable burden for combinations of risk factors were estimated taking into account mediation of different risk factors through other risk factors.

Estimates of attributable burden for each risk–outcome pair were established by multiplying the relevant cause measure by the PAF. All estimates for risk groupings or all risk factors combined are generated via an aggregation process that accounts for the fact that the effect of one risk factor might be partly or completely mediated through the effect of another. This mediation analysis is informed by individual-level data from prospective cohort studies on the joint effects of combinations of risk factors.

The comparative risk assessment (CRA) conceptual framework was developed by Murray and Lopez,[15] who established a causal web of hierarchically organised risks or causes that contribute to health outcomes, which allows for quantification of risks or causes at any level in the framework. In GBD 2019, as in previous iterations of the GBD study, we evaluated a set of behavioural, environmental and occupational, and metabolic risks, in which risk-outcome pairs were included based on evidence rules. These risks were organised in four hierarchical Levels, where Level 1 represents the overarching categories (behavioural, environmental and occupational, and metabolic) nested within Level 1 risks; Level 2 contains both single risks and risk clusters (such as child and maternal malnutrition); Level 3 contains the disaggregated single risks from within Level 2 risk clusters (such as low birthweight and short gestation); and Level 4 details risks with the most granular disaggregation, such as for specific occupational carcinogens, the subcomponents of child growth failure (stunting, wasting, underweight), and suboptimal breastfeeding (discontinued and non-exclusive breastfeeding). At each level of risk, we evaluated whether risk combinations were additive, multiplicative, or shared common pathways for intervention. This approach allows the quantification of the proportion of risk-attributable burden shared with another risk or combination of risks and the measurement of potential overlaps between behavioural, environmental and occupational, and metabolic risks. We do provide some insights into the potential magnitude of distal social, cultural, and economic factors through an analysis of the relationship between risk exposures and development measured by using the Socio-demographic Index (SDI).

Two types of risk assessments are possible within the CRA framework: attributable burden and avoidable burden. Attributable burden is the reduction in current disease burden that would have been possible if past population exposure had shifted to an alternative or counterfactual distribution of risk exposure. Avoidable burden is the potential reduction in future disease burden that could be achieved by changing the current distribution of exposure to a counterfactual distribution of exposure. Murray and Lopez identified four types of counterfactual exposure distributions: (1) theoretical minimum risk; (2) plausible minimum risk; (3) feasible minimum risk; and (4) cost-effective minimum risk.[16] The TMREL is the level of risk exposure that minimises risk at the population level or the level of risk that captures the maximum attributable burden. Other possible forms of risk quantification include plausible minimum risk – which reflects the distribution of risk that is conceivably possible and would minimise population-level risk if achieved – whereas feasible minimum risk describes the lowest risk distribution that has been attained within a population and cost-effective minimum risk is the lowest risk distribution for a population that can be attained in a cost-effective manner. Because no robust set of forecasts

for all components of GBD is available, in this study we focus on quantifying attributable burden by using the theoretical minimum risk counterfactual distribution. According to the definition of avoidable burden, risk reversibility would be incorporated into this type of assessment because it would involve reducing risk to the counterfactual for the index year, given a history of past risk exposure. Given the focus in this study on attributable burden, risk reversibility is not a criterion used in estimation here.

In general, this analysis follows the CRA methods used since GBD 2015.[17] The methods described here provide a high-level overview of the analytical logic and focus on areas of notable change from the methods employed in GBD 2015. Here we aim to provide sufficient detail on the methods and overall structure of the estimation process. This study complies with the GATHER recommendations proposed by the World Health Organization (WHO) and others, which include recommendations on documentation of data sources, estimation methods, and statistical analysis.[18]

2.2 Effect size estimation

2.2.1 Criteria for inclusion of risk-outcome pairs

Since GBD 2010 we have included risk-outcome pairs that we have assessed as meeting the World Cancer Research Fund (WCRF) grades of convincing or probable evidence.[19] In this framework, convincing evidence consists of biologically plausible associations between exposure and disease established from multiple epidemiological studies in different populations. Evidentiary studies must be substantial, include prospective observational studies, and, where relevant, randomised controlled trials (RCTs) of sufficient size, duration, and quality that show consistent effects. Probable evidence is similarly based on epidemiological studies with consistent associations between exposure and disease but for which shortcomings in the evidence exist, such as insufficient available trials (or prospective observational studies).

2.2.2 The World Cancer Research Fund grading system

2.2.2.1 Convincing evidence

Convincing evidence is evidence based on epidemiological studies showing consistent associations between exposure and disease and includes little or no evidence to the contrary. The available evidence is based on a substantial number of studies including prospective observational studies and, where relevant, RCTs of sufficient size, duration, and quality that show consistent effects. The association should be biologically plausible.

2.2.2.2 Probable evidence

Probable evidence is evidence based on epidemiological studies showing fairly consistent associations between exposure and disease, but for which perceived shortcomings in the available evidence exist or some evidence to the contrary precludes a more definite judgment. Shortcomings in the evidence may be any of the following: insufficient duration of trials (or studies); insufficient trials (or studies) available; inadequate sample sizes; or incomplete follow-up. Laboratory evidence is usually supportive. The association should be biologically plausible.

2.2.2.3 Possible evidence

Possible evidence is evidence based mainly on findings from case-control and cross-sectional studies. Insufficient RCTs, observational studies, or non-randomised controlled trials are available. Evidence based on non-epidemiological studies, such as clinical and laboratory investigations, is supportive. More trials are needed to support the tentative associations, which should be biologically plausible.

2.2.2.4 Insufficient evidence

Insufficient evidence is evidence based on findings of a few studies which are suggestive but insufficient to establish an association between exposure and disease. Little or no evidence is available from RCTs. More well-designed research is needed to support the tentative association.

2.3 Determine relative risks

The relative risk (RR) by level of exposure or by cause for mortality or morbidity can be found in published and unpublished primary studies or in secondary studies that summarise RRs. In Step 1a of the analytical process (figure S1), we collated information from RCTs, cohort, pooled cohort, and case- control studies, and in Step 1b, used these data to determine the RR for the risk-outcome pairs included in GBD 2019 (table S4). For most risks, data from pooled cohorts or meta-analyses of cohorts were used; in the case of the risk of cataracts from household air pollution (HAP), cohort data were not available, and instead we used case-control data. We estimated RRs of mortality and morbidity for 67 risk factors for which we determined attributable burden by using RR and exposure. We incorporated RRs from studies that controlled for confounding but not for factors along the causal pathway between exposure and outcome. For risk-outcome pairs with evidence available for only one element of mortality or morbidity, we generally assumed that the estimated RRs applied equally to both. Given evidence of statistically different RRs for mortality and morbidity, we incorporated different RRs for each. We did not find that RRs were consistently higher or lower for mortality compared with morbidity. Details and citation information for the data sources used for RRs are provided in searchable form through a web- tool (http://ghdx.healthdata.org/). Available data sources for determining RRs varied across risks.

For the following risks estimated from a continuous exposure distribution in which the effect size was reported by categories in pooled or meta-analysis studies, we converted those categories to RR per unit increase in exposure and assumed a linear increase in the log of the RR and exposure: ambient ozone pollution, radon, lead, high fasting plasma glucose, high LDL cholesterol, high systolic blood pressure, high body-mass index, low bone mineral density. Many meta-analyses convert RRs to per unit increase for convenience, particularly when studies choose different categories that could not otherwise be compared. If samples in the primary studies at high levels of exposure were sufficient to inform the shape of the tail of the distribution, we applied a cap to the maximum RR by using the midpoint of the last category for which an RR was reported.

In GBD 2019, for a selected set of continuous risk factors, we modelled RRs using meta-regression— Bayesian, regularised, trimmed (MR-BRT), relaxing the log-linear assumption to allow for monotonically increasing or decreasing but non-linear functions using cubic splines. The MR-BRT program is a set of wrappers customized for global health problems that use the open source mixed effects package (https://github.com/zhengp0/limetr).[20] Risk factors for which we undertook this re-analysis include: all dietary risk factors, low physical activity, kidney dysfunction, and air pollution. Because knot placement can affect the shape of the risk function when modelling with a cubic spline, we generated a wide range of knot placements and created an ensemble across these different knot placements. We also included in the final estimation 10% trimming of the data to avoid the results being sensitive to outliers.

For GBD 2019, we conducted systematic literature reviews for 18 risks. For other risk factors, only a small fraction of the existing data appears in the published literature, and other sources predominate, such as survey data and satellite data. Data were systematically screened from household surveys archived in the GHDx (http://ghdx.healthdata.org), including Demographic and Health Surveys, Multiple Indicator Cluster Surveys, Living Standards Measurement Surveys, and Reproductive Health Surveys. Other national health surveys were identified based on survey series that had yielded usable data for past rounds of GBD, sources suggested to us by in-country collaborators, and surveys identified in major multinational survey data catalogues, such as the International Household Survey Network and the WHO Central Data Catalog, as well as through country Ministry of Health and Central Statistical Office websites. Citations for all data sources used for risk factor estimation in GBD 2019 are provided in searchable form through a web-tool (http://ghdx.healthdata.org.

Information on systematic reviews were managed by using Research Electronic Data Capture (REDCap) electronic data capture tools hosted at the University of Washington.[21] REDCap is a secure, web-based application designed to support data capture for research studies that provides 1) an intuitive interface for validated data entry; 2) audit trails for tracking data manipulation and export procedures; 3) automated export procedures for seamless data downloads to common statistical packages; and 4) procedures for importing data from external sources.

2.4 Search terms and data preparation

Search terms for updates of systematic reviews for GBD 2019 are shown by risk factor in appendix section 4 Supplementary appendix 1 of Supplement to: "GBD 2019 Risk Factors Collaborators. Global burden of 87 risk factors in 204 countries and territories, 1990-2019: a systematic analysis for the Global Burden of Disease Study 2019".[2]

Survey data constitutes a substantial part of the underlying data used in the estimation process. During extraction, we concentrated on demographic variables (such as location, gender, age), survey design variables (such as sampling strategy and sampling weights), and the variables used to define the population estimate (such a prevalence or a proportion) and a measure of uncertainty (standard error, confidence interval or sample size and number of cases).

Several adjustments were applied to extracted exposure sources to make the data more consistent and suitable for modelling. In GBD 2019, we implemented adjustments of risk exposure data to deal with alternative case definitions or study methods prior to entering data into our main analytical tools of DisMod-MR 2.1 and ST-GPR. This decision also included the adjustment of data presented for both sexes to a male and female equivalent. The starting point was to explicitly state the reference case definition and study method and identify alternative definitions and study characteristics that fall within our inclusion criteria. We compiled data from both within-study comparisons (ie, data that used alternative and reference definitions in the same population) and between-study comparisons (ie, data that used an alternative definition in one population and a reference definition in another population that overlap in location, time, age and sex) of different case definitions. For between-study comparisons, we allowed a maximum calendar year difference between studies of five years. Where validation studies (ie, those carried out at the introduction of a new set of diagnostic criteria comparing to previous criteria) were available, we extracted data on the comparison of alternative to reference. For quantities of interest with multiple alternative definitions/methods we also look for pairs comparing two alternatives. In a network analysis, if A is the reference and B and C are two alternatives, a comparison of A vs B and B vs C provides an indirect comparison of the alternative C against the reference A.

We pooled either the logit difference between alternative and reference or the natural log of the ratio of alternative to reference. From simulations we found that the two methods provide almost identical results for quantities that after adjustment do not exceed a value of 0.5 (eg, prevalence or proportion). The logit difference method much better dealt with higher values and avoided prevalence or proportions to exceed one. If the values of either the reference or alternative were zero, we aggregated values across age groups until both values had non-zero observations. We used the delta method to compute the standard error of the reference and alternative measures in logit space. The standard error of the logit difference was computed as the square root of the sum of the variances of each data point in a pair.

2.5 Data analysis

We used a network random effects meta-regression in MR-BRT (see section 2.2.2). In a network analysis, if A is the reference and B and C are two alternatives, a comparison of A vs B and B vs C provides an indirect comparison of the alternative C against the reference A. To implement the network, we included dummy variables with a particular structure. This was implemented as follows, where A is the reference definition/method:

- Create *k* dummy variables where *k* are all definitions/methods other than A (eg, *k* = B, C)
- Code dummy *k* as
  - 1 if the first term of the logit difference is *k*;
  - -1 if k is second term of the logit difference;
  - 0 otherwise

  For example:

Study	Comparison	DummyB	DummyC
1	logit(B)-logit(A)	1	0
2	logit(B)-logit(A)	1	0
3	logit(C)-logit(A)	0	1
4	logit(C)-logit(A)	0	1
5	logit(C)-logit(B)	-1	1
6	logit(C)-logit(B)	-1	1

The coding structure outlined above in step 1 assumes that all case definitions are mutually exclusive. In some cases, however, individual case definitions are a function of different components or dimensions. For example, case definitions may vary by the type of symptoms that a respondent experiences as well as the recall period over which those symptoms are experienced. In the presence of sparse data, it may be difficult to find both direct and indirect comparisons of all individual case definitions. In this case, an alternative approach is to assume different dimensions of case definitions have a multiplicative effect. In other words, the effect of recall period has the same relative effect across different categories of symptoms reported by respondents. To implement this coding scheme:

- Create *k* dummy variable columns for each case definition dimension
- For each dummy variable k:
  - Add 1 if *k* is a component of the first term in the logit difference
  - Subtract 1 if *k* is a component of the second term in the logit difference

In MR-BRT, we ran random effects meta-regression of the logit difference (or log ratio) with all the *k* dummy variables as covariates, omitting the intercept in the meta-regression. We used a study_id variable for be the unique combination of the NIDs of the reference and alternative studies (or alternative1 to alternative2). The coefficients on the *k* dummy variables represent the pooled logit difference of the *k* alternative definition to the reference taking into account evidence from both direct and indirect comparisons. In the example above, the coefficient on DummyA is the pooled logit difference of B minus A; the coefficient on DummyB is the pooled logit difference of C minus A. The standard error of the pooled logit difference incorporating the between study variance was calculated as:

$$se(\text{logit}(difference_k)) = \sqrt{var_k + \gamma^2}$$

Where: $se(\text{logit}(difference_k)$= standard error of the pooled logit difference of alternative k to the reference
var_k=variance of the coefficient on dummy variable *k*
γ^2 = between-study variance

If both between and within study pairs were available, we examine whether there was a systematic difference between these. If there was a significant difference, we made judgement call as to whether within-study or between study data comparisons were most appropriate. In general, this was the within-study data, however, there were important measurement or conceptual reasons for choosing between-study data. For example, for crosswalks between self-reported height and weight compared to measured height and weight, between-study comparisons may be preferable if respondents knew they would be measured and, therefore, were less likely to misreport their height and weight.

We also examined whether there were systematic differences in the adjustments by key demographics (age, sex, geographic location, year) and other potential factors that may lead to variation in crosswalks. This could only be done at present in a direct comparison model and not in a network. We did this when there was a strong rationale, eg, biological plausibility, for variation by such characteristics. After obtaining the pooled logit difference or log ratio estimates, we predicted adjustments based on the statistical model, including uncertainty in the adjustment and sampling error of each data point. For non-significant logit differences or log ratios we still applied the adjustments if there was a conceptual reason to believe that the alternative definition is biased. This expands the variance of these alternative definition data points.

2.6 Estimates exposure

We used systematic literature reviews to identify risk factor exposure studies published or identified since GBD 2017 and combined these with existing data from household and health examination surveys and census, morbidity, or satellite imagery and ground sensor data (used for estimation of particulate matter <2.5 μm in diameter [$PM_{2.5}$]). Certain risks, such as poor diet and excessive alcohol consumption, also incorporated administrative record systems. Data sources used in estimating risk factor exposure can be accessed through the data source tool at http://ghdx.healthdata.org/.

Once data were collected and compiled, the analytical flowchart describes the adjustments applied, where necessary, to correct for bias. Examples of these adjustments include use of urban studies for lead; crosswalks between different measurements, methods, and definitions, such as for self- report of obesity and glycated haemoglobin (HbA_{1C}) for diabetes; and age-sex splitting of data, such as for fasting plasma glucose (FPG) level, cholesterol level, and systolic blood pressure that may be reported from broad age-groups.

For the GBD, we developed two modelling approaches, a Bayesian meta-regression model (DisMod-MR 2.1) and a spatiotemporal Gaussian process regression model (ST-GPR), to pool data from different sources, control and adjust for bias in data, and incorporate other types of information such as country- level covariates. DisMod-MR 2.1 and ST-GPR are mixed effect models that borrow information across age, time, and locations to synthesise multiple data sources into unified estimates of levels and trends. A detailed description of the likelihood used for estimation and a full description of improvements made for DisMod-MR 2.1 were detailed by Vos and colleagues,[22] who provided additional detail in the supplementary materials to that paper.[8] The ST-GPR model has three main hyper-parameters that control for smoothing across time, age, and location. Values for these hyper-parameters were selected on the basis of cross- validation. Cross-validation tests were conducted for different combinations of the hyper-parameters for three types of models: one data-sparse model, one data-moderate model, and one data-dense model. In each test, 20% of the data were held out, and the performance of each combination of hyper- parameters was evaluated on the held-out data. For each hyper-parameter combination, 10 cross-validation tests were conducted. The performance of each model in predicting the withheld 20% of the data was evaluated by using a combined measure based on root mean square error (RMSE) and uncertainty interval (UI) coverage. A detailed description of the ST-GPR process regression can be found below.

The main difference between these methods is their power to include unstructured types of data by sex and age group and their degree of flexibility. DisMod-MR 2.1 is the preferred tool in these cases because of its ability to integrate over age and adjust for different exposure definitions in the data; however, the use of Bayesian Markov Chain Monte Carlo (MCMC) simulations with large volumes of data renders the analysis computationally intensive and reduces the number of iterations that are possible. If standard age-group data are available – as is generally the case for metabolic risks – using ST-GPR becomes the preferred approach.

In some cases, we adapted our methods of modelling exposure to risks where necessary to account for complexities in the risk-outcome relationship or the need for particular handling of data, for example, dietary risks and ambient air pollution (see appendix section 4 to: "GBD 2019 Risk Factors Collaborators. Global burden of 87 risk factors in 204 countries and territories, 1990-2019: a systematic analysis for the Global Burden of Disease Study 2019"[2] for more detail). A complete list of risks is reported in table S2, and additional details for adjustments or adaptations to particular risk models are provided in appendix section 4 ("GBD 2019 Risk Factors

Collaborators. Global burden of 87 risk factors in 204 countries and territories, 1990-2019: a systematic analysis for the Global Burden of Disease Study 2019”).[2]

2.6.1 Theoretical minimum-risk exposure level (TMREL)

In this and all previous GBD studies, the counterfactual level of risk exposure used is the risk exposure that is both theoretically possible and minimizes risk in the exposed population that consequently captures the maximum population-attributable burden.[16] For each risk evaluated in GBD 2019, Step 4 of the analytical flowchart describes the use of the best available epidemiological evidence from published and unpublished RRs by level of exposure and the lowest observed level of exposure from cohorts, used to select a single level of risk exposure that minimises risk from all causes of deaths combined to establish the TMREL. In principle, the TMREL for a given risk may vary by age, sex, and location if supported by clear evidence. Based on the available evidence, the TMREL itself can be uncertain, which is reflected in the 95% UIs in table S6. In GBD 2019, we updated the process of estimating TMREL for dietary risks. We set the TMREL to zero for all harmful dietary risk factors with monotonically increasing risk functions (eg, processed meat intake); this excludes sodium. For protective risks with monotonically declining risk functions with exposure (eg, fruit intake), we first determined the 85th percentile of exposure in the cohorts or trials used in the meta-regression of each outcome that was associated with the risk. Then, we determined the TMREL by weighting each risk-outcome pair by the relative global magnitude of each outcome.

2.6.2 Estimate population-attributable fractions

Risks are categorised on the basis of how exposure was measured: dichotomous, polytomous, and continuous. High low-density lipoprotein (LDL) cholesterol level is an example of a risk measured on a continuous scale. The PAF, which represents the proportion of risk that would be reduced in a given year if the exposure to a risk factor in the past were reduced to an ideal exposure scenario, is defined for a continuous risk factor as:[21]

$$PAF_{joasgt} = \frac{\int_{x=l}^{u} RR_{joasg}(x)P_{jasgt}(x)dx - RR_{joasg}(TMREL_{jas})}{\int_{x=l}^{u} RR_{joasg}(x)P_{jasgt}(x)dx}$$

Where PAF_{joasgt} is the PAF for cause o due to risk factor j for age group a, sex s, location g, and year t.

$RR_{joasg}(x)$ is the RR as a function of exposure level x for risk factor j for cause o, age group a, sex s, and location g with the lowest level of observed exposure as l and the highest as u; $P_{jasgt}(x)$ is the distribution of exposure at x for age group a, sex s, location g, and year t; and $TMREL_{jas}$ is the TMREL for risk factor j, age group a, and sex s.

The PAF_{joasgt} for dichotomous and polytomous risk factors for every country is defined as:

$$PAF_{joasgt} = \frac{\sum_{x=1}^{u} RR_{joast}(x)P_{jasgt}(x) - RR_{joasg}(TMRE_{jas})}{\sum_{x=1}^{u} RR_{joas}(x)P_{jasgt}(x)}$$

where PAF_{joasgt} is the PAF for cause o due to risk factor j for age group a, sex s, location g, and year t.

$RR_{joasg}(x)$ is the RR as a function of exposure level x for risk factor j for cause o, age group a, sex s, and location g on a plausible range of exposure levels from *l* to *u;* $P_{jasgt}(x)$ is the proportion of the population in risk group (prevalence) for age group a, sex s, location g, and year t; and $TMREL_{jas}$ is the TMREL for risk factor j, age group a, and sex s.

2.6.3 Estimate summary exposure values

$$SEV_{rc} = \frac{\frac{PAF_{rc}}{1 - PAF_{rc}}}{RR_{max} - 1}$$

We first calculate risk, r, and cause, c, for specific SEVs by using the following equation, for each most-detailed age, sex, location, year, and outcome. PAF is the YLL (expect for occupational noise, bullying victimization, and occupational ergonomic factors, which are YLD only and thus use the YLD) PAF. RR_{max} for categorical risks is the RR at the highest category of exposure. For continuous risks, this is

otherwise, and for custom modelled risks like ambient particulate matter pollution, HAP from solid fuels, alcohol, smoking, bullying, and activity, the modeller provides draws of RR_{max}. Generally, RRs do not vary across time and space. However, exceptions exist, such as risks from second-hand smoke (SHS) or HAP for which the RR is based on the integrated exposure response (IER) curve. In these cases, the RR is averaged across location and year to ensure no time or space variation. If the PAF is negative, which signifies a protective effect for that outcome, the PAF is set to 0 and the SEV is then also 0 because the SEV is univariate and constrained to be a value between 0 and 1. Once we obtained a set of risk-cause specific SEVs at the most-detailed risk, cause, age, sex, and location for all years, we averaged across causes to produce the final risk specific SEV_r, where

$$RR_{max} = RR^{\frac{TMREL-1^{st}exposure}{RR_{scalar}}} \text{ if protective, or}$$

$$= RR^{\frac{99^{th}exposure-TMREL}{RR_{scalar}}}$$

$$SEV_r = \frac{1}{N(c)}\sum_c SEV_{rc}$$

$N(c)$is the total number of outcomes for a risk.

2.6.4 Mediation

The portion of the burden of disease that is attributable to various combinations of risk factors or to all risk factors combined has been a topic of broad interest.[30] In GBD 2010, we only aggregated the burden of risk factors for some clusters of risks, including access to improved water and sanitation, child and maternal malnutrition, tobacco smoking, alcohol use, dietary risk factors, occupational risk factors, and sexual abuse and violence. We did not aggregate air pollution and metabolic risk factors. For GBD 2013 onwards, we aggregated all risk factors into three large categories—behavioural, environmental and occupational, and metabolic risks—and aggregated all GBD risk factors into a single attributable fraction for each disease and eventually for all causes of burden.

Aggregating risk factors at different levels shares three essential challenges:

1. Risk factor coexistence or aggregation: for example, metabolic risk factors often occur together, or high-risk behaviours such as drug abuse and unsafe sex are related.
2. Mediation: a risk factor may affect another risk factor that lies in the physiological pathway to a disease outcome. It can be inside a cluster of risk factors, such as the effect of obesity through an increase in FPG level and later cardiovascular disease (CVD) outcomes, or between clusters of risk factors, such as the effect of fibre on cholesterol.
3. The formula used to calculate the aggregated PAF.

The aggregation method is conceptually applicable to other aggregations such as socioeconomic factors, education, homelessness, and refugee status that are being considered for inclusion in future GBD iterations. In the next section, we explain our approach to dealing with these challenges.

There are three patterns of associations between risk factors to consider (Figure C). The first concerns confounding; risk B affects risk A and outcome C (Pattern 1 in *Patterns of associations between risk factors*). In these cases, the RR for A should be adjusted for B; for example, the fruit RR is adjusted for smoking. If part of the effect of A is through B, a mediator, we do not adjust the effect of A for B. For example, we do not adjust the RR of body-mass index (BMI) for cholesterol because cholesterol lies in the biological pathway between BMI and cardiovascular outcomes (Pattern 2 in in *Patterns of associations between risk factors*). The third pattern occurs when risks A and B are proxies of a third variable Z and aggregation aims to estimate the total effect of a latent variable Z on C. An example is child growth failure, which is measured by stunting, wasting, and underweight as proxies.

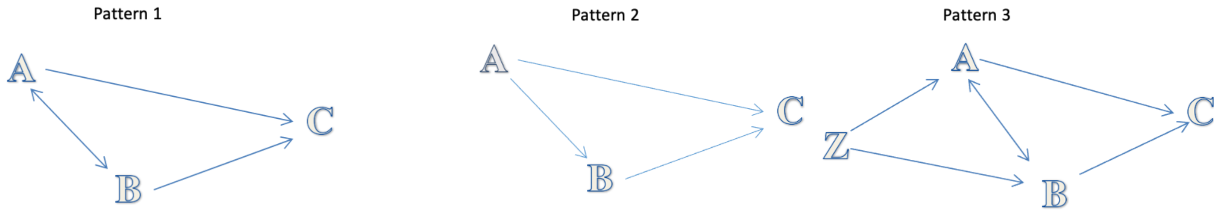

2.6.5 Calculating the burden of multiple risk factors

Validation studies have reported congruency between the true risk associated with multiple risk factors affecting the same outcome and a multiplicative aggregation of the PAFs of the individual risk factors (formula below).[22]

$$PAF_{1..i} = 1 - \prod_{i=1}^{n}(1 - PAF_i)$$

where *PAF* is the population attributable fraction and *i* is each individual risk factor. The same validation studies also found that the overestimation from ignoring the covariance between risk factors is small. This small overestimation was important to note because few data sources exist from which we can draw information on covariance. We endeavoured to evaluate RRs that were controlled for confounders. However, because we had to rely on the literature for many RRs, we did not always have full control over the choice of confounders controlled for in each study.

2.6.6 Adjusting for mediation

When aggregating the effects of multiple risk factors, we included an MF if a part of the effect of one risk factor was included in the effect estimated for in the mediator. First, we prepared a list of possible mediations, and especially between behavioural risks and metabolic risk factors with cardiometabolic outcomes. We did not assume any mediation effect between risk factors for cancers. Danaei and colleagues assumed that part of the effect of BMI on ischaemic heart disease (IHD) is through high systolic blood pressure (SBP), cholesterol level, and FPG.[23] The proportion of the BMIeffect that can be explained by other metabolic risk factors is the amount of mediation. The difference between the crude RR of BMI on IHD with the RR adjusted for SBP, FPG, and cholesterol level reflects the amount of BMI effect on IHD that is mediated and already included in SBP, FPG, and cholesterol level:

$$MF = \frac{RR_{crude} - RR_{adjusted}}{RR_{crude} - 1}$$

So, to aggregate the PAF of multiple risk factors, we first calculated the part of the excess risk (RR-1) of every risk factor that is not mediated, re-compute the PAF so that it only includes the non-mediated risk then aggregated PAFs by assuming they are independent. Therefore, if MF is the mediation factor of R2 through R1, the adjusted RR for R2 including only the non- mediated component of risk is The PAF accounting for mediation is then computed using the adjusted RR and the joint PAF computed. For every paired risk factor and outcome, the matrix of possible mediations was calculated and used.

$$RR_{1.2} = MF_{2/1}(RR_2 - 1) + 1$$

2.6.7 Calculating mediation factor

The best example is the mediation of BMI through SBP, FPG, and cholesterol level reported by Danaei et al.[23] In their meta-analysis, they report the adjusted and unadjusted RR of BMI on IHD and stroke based on combined data from individual cohorts. They calculated the MF by using the following equation, and we used it directly as the MF in risk factor aggregation. Using individual-level data from cohort studies, we estimated the MF for other metabolic risk factors and some dietary risks.

$$MF = \frac{RR_{crude} - RR_{adjusted}}{RR_{crude} - 1}$$

For many other risk factors, no data are available to enable the use of the first method. Instead, we searched studies to estimate the effect of the risk factor on the mediator and, finally, the expected increase in IHD risk. We pooled available studies to calculate the unit increase in the mediator per unit increase in the risk factor to

calculate the size of the IHD RR.

2.6.8 Uncertainty of aggregated and mediated PAFs

We generated 1000 draws of the posterior distribution of the MF calculated by different methods to use beside draws of other inputs to the PAF aggregation.

2.6.9 Important assumptions in aggregating risk factors and including mediation

1 – The MFs or PAF adjustments are similar across countries, age, sex, and years. Although the size of mediation is probably different in different populations, little data is available to inform the covariance between different risk factors or the MF amount by age and country. For example, in some countries, the size of the mediated BMI-IHD PAF exerted through cholesterol level, as calculated by the MF, was even bigger than the total burden of cholesterol level. This finding indicated that less of the effect of BMI is mediated through cholesterol level and MFs are not similar across countries.

2 – For many risk-mediator-outcome pairs, no data are available, so we assumed the mediation is zero.

3 – Because the covariance between undernutrition indicators differs by location (and across time, but results were not reported), and an interaction exists between these indicators, the total burden might be underestimated.

4 – We assumed no significant covariance between PAFs, which might not be true between some risk factors, such as metabolic risk factors. Although this overestimation can be controlled by using adjusted RRs, using crude RRs for BMI and other metabolic risk factors may cause significant overestimation of the aggregated metabolic risks burden.

2.6.10 Estimate attributable burden

Four key components are included in the estimation of the burden attributable to a given risk factor: the metric of burden being assessed (the number of deaths, YLLs, YLDs, or DALYs [the sum of YLLs and YLDs]); the exposure levels for a risk factor; the RR of a given outcome due to exposure; and the counterfactual level of risk factor exposure. Estimates of attributable burden as DALYs for risk-outcome pairs were generated by using the following model:

$$AB_{jasgt} = \sum_{o=1}^{w} DALY_{joasgt} PAF_{joasgt}$$

where AB_{jasgt} is the attributable burden for risk factor j for age group a, sex s, location g, and year t;

$DALY_{joasgt}$ is total DALYs for cause o (of w relevant outcomes for risk factor j) for age group a, sex s, location g, and year t; and PAF_{joasgt} is the PAF for cause o due to risk factor j for age group a, sex s, location g, and year t. The proportions of deaths, YLLs, or YLDs attributable to a given risk factor or risk factor cluster were analogously computed by sequentially substituting each metric in place of DALYs in the equation provided.

2.6.11 Decomposition analysis of deaths and DALYs

We conducted a decomposition analysis of changes in DALYs from 2010 to 2019, decomposing changes in all-age cause-specific DALYs attributable to all risk factors and individual risk factors due to changes in population growth, population age structure, exposure to the given risk for a disease, and risk-deleted death and DALY rates. In this case, risk-deleted rates are the rates obtained after removing the effect of a risk factor or combination of risk factors — in other words, observed DALY rates multiplied by one minus the PAF for the risk or set of risks. Our decomposition analyses draw from methods developed by Das Gupta[24] to provide a computationally tractable solution for

$$E_A = (A_{19} - A_{10})\left(\frac{B_{10}C_{10} + B_{19}C_{19}}{3} + \frac{B_{10}C_{19} + B_{19}C_{10}}{6}\right)$$

isolating drivers of burden changes whereby all combinations of possible pathways are averaged across factors. Attributable burden was determined, following the methods of Das Gupta, as a product of three factors such that: where T_{asgt} represents the attributable burden at year t; $A_{sgt}$is the age-specific population size for a given age group a, sex s, and location g at year t; B_{asgt} is the underlying rate of the outcome unrelated to the risk factor or observed rate, multiplied by $1 - PAF$ for a given age group a, sex s, and location g at year t; and C_{asgt} is the ratio of the attributable burden to the underlying rate, which reflects the risk exposure effect for a given age group a, sex s, and location g at year t defined as $PAF/(1 - PAF)$when decomposing attributable burden to a risk. Risk exposure effects for individual risk factors are scaled such that they sum to the all-risk exposure effect by location, age, sex, and cause accounting for mediation. This process allows for aggregation of risks; the exposure for all risks for a disease can be split into exposure to metabolic, behavioural, and environmental risks. The contribution of each factor to total change in attributable burden was determined by changing the level of one factor from time t_0 to t_1 – here 2010 to 2019 – with all other factors held constant. Thus, the effect of any of the three factors, for example A_{asgt}on the change of the attributable burden between 2010 (A_{10}) and 2019 (A_{17}) is calculated as:

where E_A is the proportion of change due to factor A, and the subscripts for each factor in the equation denote the year for each estimate. Because the effect depends on the order of entry of the factor, we calculated the average of all combinations of the three factors.[35] The proportion of change due to factorA_{sgt}, the age-specific population size for a given age group a, sex s, and location g at year t, is then further split, setting change in population growth equal to the percentage change in the all-age population from time t_0 to t_1 and change in population age structure to the residual, giving four factors.

This three-factor decomposition method does not work for risks for which the PAF, by definition, is 100% (such as high FPG level and type 2 diabetes) or for which the PAF is directly estimated (such as for unsafe sex and HIV). In the cases of child underweight and protein-energy malnutrition, child wasting and protein-energy malnutrition, short gestation for birthweight and neonatal preterm birth complications, low birthweight for gestation and neonatal preterm birth complications, iron deficiency (ID) and iron-deficiency anaemia (IDA), alcohol use and liver cancer due to alcohol use, alcohol use and cirrhosis and other chronic liver diseases due to alcohol use, alcohol use and alcohol use disorders, alcohol use and alcoholic cardiomyopathy, drug use and drug use disorders, occupational particulate matter, gases, and fumes and other pneumoconiosis, occupational particulate matter, gases, and fumes and coal workers pneumoconiosis, occupational exposure to asbestos and asbestosis, and occupational exposure to silica and silicosis, we used a two-factor decomposition method that examines the contribution of population, ageing, and risk exposure. Effectively, we assumed trends in these cases are driven by exposure, not change in the risk-deleted rates. Conversely, for unsafe sex and sexually transmitted diseases excluding HIV, we used a two-factor decomposition method that examines the contribution of population, ageing, and risk-deleted death and DALY rates and assumed that trends in these cases are driven by risk-deleted rates, not change in exposure. For high FPG level and type 1 and 2 diabetes, high FPG level and CKD due to type 1 and 2 diabetes, high SBP and hypertensive heart disease, high SBP and CKD due to hypertension, and impaired kidney function and CKD, we used GBD estimates of SEVs for the given risk and the case-fatality rate to decompose trends into an estimate of the contribution of the three factors. Similarly, for unsafe sex and cervical cancer, we used GBD estimates of the incidence of cervical cancer and the case-fatality rate to decompose trends into an estimate of the contribution of the three factors. For unsafe sex and HIV, we used spectrum counterfactual and CD4 risk-weighted prevalence.

Section 3. Changes in the modelling of stroke for GBD 2019

Several changes were made to the modelling strategy for stroke for the GBD 2019 study. In GBD 2017 and prior, chronic stroke was modelled for both subtypes (ischaemic and haemorrhagic or other) together to estimate the total prevalence of chronic stroke. For the GBD 2019 study, each stroke subtype was modelled independently, resulting in separate acute and chronic stroke models for ischaemic stroke (IS), intracerebral haemorrhage (ICH) and subarachnoid haemorrhage (SAH) type separately. This change was made in order to simplify the stroke modelling process and to ensure that all major pathological types were estimated correctly. In the GBD2017 and

prior studies, severity splits were based on estimates derived from standard GBD analysis of the U.S. Medical Expenditure Panel Survey. For the GBD2019 study, a review of studies reporting modified Rankin scores following stroke was performed and disability weights were applied using a model of modified Rankin level by age and sex as described above. In GBD 2019, we updated the regressions for stroke and diabetes. We dropped the proportion of garbage from the regression formula and ran regression on high-quality, low proportion garbage data (4/5 stars, < 50% GC). We also included all covariates included in the CODEm models for both stroke and diabetes.

For the GBD 2019 study, in order to better represent population-level disease incidence for IS, adjustments for alternative study methods and case definitions were applied to data prior to analysis in DisMod-MR. These adjustments were performed using the MR-BRT modelling tool. We adjusted for several study-specific factors such as whether the data were from a hospital and whether the data included both first-ever and recurrent ischaemic strokes. We updated our methods for redistributing deaths due to unspecified stroke (ICD-10 codes I62 and I64) to the three modelled stroke subtypes included in GBD. All available data on incidence, prevalence and mortality were used to estimate stroke burden. Mortality was estimated using vital registration, verbal autopsy reports and trained informants, while incidence data were estimated using the DisMod-MR meta-regression modelling tool.[3]

The tables below indicate the covariates used by cause in the estimation process, as well as the beta and exponentiated beta values.

Step 1:

Cause	Variable name	Measure	beta	Exponentiated beta
Chronic ischaemic stroke	Log-transformed SEV scalar: Ischaemic Stroke	Prevalence	0.83 (0.75 — 1.03)	2.29 (2.12 — 2.80)
Chronic ischaemic stroke	LDI (I$ per capita)	Excess mortality rate	-0.16 (-0.29 — -0.1)	0.85 (0.75 — 0.90)
Chronic haemorrhagic stroke	Log-transformed SEV scalar: Hem Stroke	Prevalence	0.79 (0.75 — 0.92)	2.21 (2.12 — 2.50)
Chronic haemorrhagic stroke	LDI (I$ per capita)	Excess mortality rate	-0.12 (-0.16 — -0.1)	0.89 (0.85 — 0.90)
First ever acute haemorrhagic stroke	Hospital data	Incidence	0.54 (0.54 – 0.54)	1.71 (1.71 – 1.72)
First ever acute haemorrhagic stroke	Any stroke	Incidence	1.27 (1.27 – 1.28)	3.57 (3.56 – 3.59)
First ever acute haemorrhagic stroke	First-ever acute stroke, ischemic or haemorrhagic	Incidence	0.52 (0.52 – 0.53)	1.69 (1.68 – 1.71)
First ever acute haemorrhagic stroke	Log-transformed age-standardized SEV scalar: haemorrhagic stroke	Incidence	0.77 (0.75 – 0.82)	2.17 (2.12 – 2.27)
First ever acute haemorrhagic stroke	Any stroke	Excess mortality rate	-0.48 (-0.66 – -0.32)	0.62 (0.52 – 0.73)
First ever acute haemorrhagic stroke	First-ever acute stroke, ischemic or haemorrhagic	Excess mortality rate	-0.081 (-0.3 – 0.16)	0.62 (0.52 – 0.73)
First ever acute ischaemic stroke	Hospital data	Incidence	0.38 (0.37 – 0.38)	1.46 (1.45 – 1.46)
First ever acute ischaemic stroke	Any stroke	Incidence	0.31 (0.29 – 0.33)	1.37 (1.34 – 1.39)
First ever acute ischaemic stroke	First-ever acute stroke, ischemic or haemorrhagic	Incidence	0.37 (0.36 – 0.38)	1.44 (1.43 – 1.46)

First ever acute ischaemic stroke	Log-transformed age-standardized SEV scalar: ischemic stroke	Incidence	1.16 (1.09 – 1.22)	3.21 (2.99 – 3.39)

Step 2:

Cause	Variable name	Measure	beta	Exponentiated beta
Chronic ischemic stroke with CSMR	Log-transformed SEV scalar: Ischaemic stroke	Prevalence	0.89 (0.75 – 1.19)	2.44 (2.13 – 3.27)
Chronic ischemic stroke with CSMR	LDI (I$ per capita)	Excess mortality rate	-0.49 (-0.5 – -0.46)	0.61 (0.61 – 0.63)
Chronic haemorrhagic stroke with CSMR	Log-transformed SEV scalar: Haemorrhagic stroke	Prevalence	0.88 (0.75 – 1.15)	2.40 (2.13 – 3.17)
Chronic haemorrhagic stroke with CSMR	LDI (I$ per capita)	Excess mortality rate	-0.48 (-0.5 – -0.44)	0.62 (0.61 – 0.64)
First-ever acute haemorrhagic stroke with CSMR	Any stroke	Incidence	1.27 (1.27 – 1.29)	3.58 (3.56 – 3.62)
First-ever acute haemorrhagic stroke with CSMR	First-ever acute stroke, ischemic or haemorrhagic	Incidence	0.52 (0.52 – 0.54)	1.69 (1.68 – 1.71)
First-ever acute haemorrhagic stroke with CSMR	Log-transformed SEV scalar: Hem stroke	Incidence	1.11 (1.01 – 1.20)	3.03 (2.74 – 3.33)
First-ever acute haemorrhagic stroke with CSMR	Any stroke	Excess mortality rate	-0.37 (-0.49 – -0.27)	0.69 (0.62 – 0.77)
First-ever acute haemorrhagic stroke with CSMR	First-ever acute stroke, ischemic or haemorrhagic	Excess mortality rate	0.023 (-0.2 – 0.23)	1.02 (0.82 – 1.25)
First-ever acute ischaemic stroke with CSMR	Any stroke	Incidence	0.32 (0.30 – 0.33)	1.38 (1.35 – 1.39)
First-ever acute ischaemic stroke with CSMR	First-ever acute stroke, ischemic or haemorrhagic	Incidence	0.37 (0.36 – 0.38)	1.44 (1.43 – 1.46)
First-ever acute ischaemic stroke with CSMR	Log-transformed age-standardized SEV scalar: Ischemic stroke	Incidence	1.11 (1.05 – 1.18)	3.04 (2.86 – 3.26)
First-ever acute ischaemic stroke with CSMR	Any stroke	Excess mortality rate	-0.34 (-0.45 – -0.24)	0.71 (0.64 – 0.79)
First-ever acute ischaemic stroke with CSMR	First-ever acute stroke, ischemic or haemorrhagic	Excess mortality rate	-0.69 (-0.82 – -0.56)	0.50 (0.44 – 0.57)

In GBD 2019, no substantial changes were made to Dismod-MR 2.1 but we made more substantial changes to how we use the tool. First, we added the year 2019 as an additional year of estimation. Second, we also included the option again to have random effects on cause-specific mortality rates (csmr) and excess mortality rates

(EMR). This functionality had been dropped a couple of GBD rounds earlier. Third, as we did all our adjustments for alternative case definition and study methods as well as adjustments to both sex data points prior to entering data into DisMod-MR 2.1, we no longer used the functionality in DisMod-MR 2.1 to estimate coefficients for study covariates. Fourth, based on simulation testing we found that coverage improved, and errors reduced when passing down priors with a wider setting of minimum coefficient of variation (which determines the uncertainty around priors and hence how 'informative' the priors are) than had generally been used in past GBD iterations. We settled on a default value of 0.8 where in the past values of 0.4 or less had been more commonly used. We made some exceptions for high prevalent conditions where a lower minimum CV setting achieved the task of making priors less informative but not completely uninformative.

Fifth, we changed our approach to estimating excess mortality rates, the key link in the model between cause-specific mortality rates (CSMR) and incidence and prevalence. In the past two GBD rounds we calculated priors on excess mortality and entered these as data points by matching sex-specific prevalence data with an age width of 20 or less with the corresponding CSMR for the same location and year. For stability sake, we excluded calculation of EMR for prevalence data points of less than 1 in a million. EMR is simply calculated as CSMR divided by prevalence. As with previous GBD years, for diseases with an average duration of less than a year (as indicated by a setting of remission greater thanone), we ran an initial global model to get an equivalent prevalence and used the following formula to calculate EMR:

EMR = CSMR * (remission + (ACMR - CSMR) + EMR_pred) / incidence

where, ACMR = all-cause mortality rate and EMR_pred = EMR fit from an initial global DISMOD model.

Section 4. Details of the Results

4.1 Overall stroke burden

There was a significant heterogeneity in the amount and even direction of changes in age-standardised rates and absolute number of people who developed stroke, died from or remained disabled from stroke in GBD regions (Supplement Figure 1), countries and country-income level groups from 1990 to 2019 (Supplement Table 1). While the overall age-standardised stroke incidence, prevalence, mortality and DALYs rates decreased from 1990 to 2019, over the last decade age-standardised stroke incidence and particularly prevalence rates plateaued or even showed a trend towards increasing across all seven GBD super regions (Figure 2). While the number of deaths from stroke in high-income countries (HIC) decreased by 9.0% (-16.0; -4.0), it did increase by 72-78.0% (52.0-100.0) in LIC/LMIC and by 46% in upper middle-income countries (UMIC) (Supplement Table 1). The number of DALYs decreased by 16.0% (-20.0; -13.0) in HIC and increased by 28-65.0% (12.0-80.0) in LIC/LMIC. The number of prevalent and incident strokes increased both in HIC (39.0% [36.0-43.0] and 11.0% [8.0-15.0], respectively) and LIC/LMIC/UMIC (100-110.0% [92.0-112.0] and 83-98.0% [75.0-101.0], respectively).

In 2019, there were almost 6-fold between-country variations (59-300/100,000 range) in age-standardised stroke incidence rates (Figure 1a, Supplement Table 1), with the highest rates observed in Southern Asia, particularly in Indonesia (293.3/100,00 [262.3-331.6]), and lowest in Australasia, particularly in Australia (65.0/100,000 [58.6-72.0]). Age-standardised rates of stroke death (Supplement Figures 4-6) were highest in Central Asia (particularly in Mongolia 220/100,00 [180-277]) and lowest in Australasia (particularly in Australia 26.2/100,000 [21.9-28.9]) (almost 15-fold between-country differences). Two to four-fold between-country differences were observed in age-standardised DALYs rates (highest in Central Asia, particularly in Mongolia 4,747/100,00 [3,782-6,035]); lowest in Australasia, particularly in Australia 445/100,000 [399-488]) and prevalence (highest in North Africa and Middle East, particularly in Iraq and Saudi Arabia 1,968/100,000 [1,818-2,144]; lowest in Australasia, particularly in Australia 651.1/100,000 [597.0-707.5], and Western Europe, particularly in Switzerland 608.3/100,000 [561.6-662.2]) (see Supplement Section 4.1 for details).

While there was continuous linear decline in stroke incidence rates in people aged 70+ years from 1990 to 2019 (-19.0% [-21.0; -17.0]), this slowed down in 2010-2019 (-4.0% [-6.0; -1.0]) compared to 1990-2010 (-16.0% [-19.0; -13.0]), and stroke incidence rates in people aged <70 years (Supplement Figure 7) did not change from

1990 to 2010 (1.0% [-2.0; 3.0]) but increased significantly by 14% (11.0-17.0) from 2010 to 2019. Reductions in age-standardised rates of stroke incidence, prevalence, mortality and DALYs have not kept pace with population growth and ageing of the population. As a result, the absolute number of incident and prevalent stroke cases, deaths from stroke and DALYs due to stroke have increased almost linearly each year since 1990 (Supplement Figure 2).

Although age-standardised rates of stroke incidence, prevalence, deaths and DALYs declined significantly from 1990 to 2019 (Table 1), there was a slower rate of decline of age-standardised incidence, deaths and DALYs in 2010-2019 study period (-1.0% [-3.0; 0.0]; -15.0% [-20.0; -10.0]; -14.0 [-19.0; -8.0], respectively) compared with 1990-2010 study period (-16.0% [-17.0; -14.0]; -26.0% [-30.0; -22.0]; -25.0% [-29.0; -21.0], respectively), while age-standardised prevalence increased from 2010 to 2019 by 2.0% (1.0-3.0) but decreased from 1990 to 2010 by 8.0% (-9.0; -6.0).

4.2 Burden of pathological types of stroke

IS, ICH and SAH cases constituted 64.5% (61.4-67.4), 15.8% (15.5-16.2) and 19.7% (18.4-21.0) of all stroke cases in HIC, and 62.6% (59.4-65.3), 29.5% (28.4-30.3) and 7.9% (8.5-8.3), respectively, in UMIC/LMIC. Age-standardised incidence rates of IS in LMIC/UMIC (79.7-126.6/100,000 [69.3-150.0]; 76.9-123.6 [66.8-146.4] in males and 82.0-128.9 [71.4-152.6] in females) were significantly greater than that in HIC (57.9/100,000 [50.0-66.8]; 55.2 [47.9-63.9] in males and 59.5 [51.2-68.6] in females). There was a significant reduction of age-standardised IS incidence rates in both male and females in HIC from 1990 to 2019 but mostly insignificant changes in age-standardised IS incidence rates in LIC/UMIC/LMIC (Supplement Figure 9). Age-standardised rates of ICH in LIC/UMIC/LMIC were almost 2-4 times greater (39.6-71.2/100,000 [34.1-77.9]) than that in HIC (14.9/100,000 [13.3-16.9]) with a trend towards reduction of ICH incidence rates in both HIC and LMIC regardless of sex. Contrary to ICH, age-standardised incidence rates of SAH in HIC (19.8/100,000 [16.7-23.1]; 16.2 [13.7-18.9] in males and 23.1 in females [19.5-27.0]) were significantly greater than that in LIC/UMIC/LMIC (10.5-13.5/100,000 [8.7-15.9]; 10.7-12.6 [8.8-14.6] in males and 10.4-14.4 [8.7-16.9] in females), with the trend towards increasing SAH incidence rates in females (8.0% [5.0-12.0] and males (6.0% [3.0-8.0]) in HIC, but decreasing rates in females (-15.0% to -24.0% [-17.0; -22.0]) and males (-18.0% to - 30.0% [-19.0; -27.0]) in LIC/UMIC/LMIC (Supplement Figure 10).

The highest age-standardised incidence rates of IS were in North Africa and Middle East and East Asia, particularly in United Arab Emirates (208.2/100,000 [180.3-240.9]) and China (144.8/100,000 [121.6-173.2]), the lowest – in Australasia, particularly in New Zealand (40.4/100,000 [34.7-46.6]). The highest age-standardised incidence rates of ICH were in Central and Southeast Asia, particularly in Mongolia (166.5/100,00 [156.5-179.1]), and the lowest – in Australasia, particularly in New Zealand (8.8/100,000 [7.6-10.3]). For age-standardised incidence rates of SAH, the highest values were observed in High-Income Asia Pacific region, particularly in Japan (63.4/100,000 [53.1-74.8]), the lowest – in Southern Sub-Saharan Africa, particularly in South Africa (6.5/100,000 [5.3-7.9]).

Age-standardised IS prevalence was highest in North Africa and the Middle East, particularly in Iraq (1,656.7/100,000; 1,500.2-1,820.0), and lowest in South Asia, especially in Nepal [376.7/100,00; 337.1-417.4]). Age-standardised IS death rates were highest in Eastern Europe, especially in North Macedonia [205.8/100,000; 172.7-240.5], and lowest in High-Income North America, especially in Canada [14.6/100,000; 11.9-16.5]). Age-standardised DALYs rates in Eastern Europe, especially in North Macedonia [2,856.2/100,000; 2,411.2-3,341.0] were more than 10 times greater than that in Australasian region, especially in Australia (252.8/100,000; 218.7-282.9). Similar large variations were observed for age-standardised ICH prevalence (max. values in Southeast Asia region, especially in Indonesia [663.9/100,000; 557.1-777.1], lowest values in Australasian region, especially Australia [74.6/100,000; 65.8-83.7]). Even greater geographical variations were observed for age-standardised ICH death rates (max. value in Central Asia, especially Mongolia [178.5/100,000; 144.6-225.0], lowest value in Australasia, especially Australia [6.7/100,00; 5.9-7.5]), and age-standardised ICH DALYs rates (maximum value in Southeast and Central Asia, especially Mongolia [3,841.0/100,000; 3,025.9-4,888.8] and Indonesia [2,166.2/100,000; 1,778.0-2556.4], and lowest value in Australasia, especially Australia [120.3/100,00;108.6-131.5]). We observed almost 10-fold geographical variations in age-standardised prevalence of SAH (max. values in High-Income Asia Pacific region, especially in Japan (372.9/100,000; 298.9-455.5), lowest values in Southern

Sub-Saharan Africa region, especially in South Africa (39.6/100,000; 32.8-47.6). Highest age-standardised rates of death from SAH were observed in Central Asia, especially in Mongolia (14.7/100,000; 11.0-19.4), and lowest rates - in Western Sub-Saharan Africa, especially in Nigeria (1.35/100,000; 0.7-2.6). We observed highest age-standardised rates of DALYs due to SAH in Eastern Europe, especially in North Macedonia (296.3/100,000; 232.3-271.4), and lowest rates in Western Sub-Saharan Africa, especially in Nigeria (44.7/100,000; 27.4-85.0).

4.3 Stroke related DALYs attributable to risk factors

Although the ranking of risk clusters for stroke-related DALYs did not change from 1990 to 2019 (metabolic risks were followed by behavioural risks and environmental risks), the share of DALYs due to risk clusters did change significantly from 1990 to 2019: metabolic risk risks increased by 5.5% (4.2-7.0) while environmental and behavioural risks decreased by 7.5% (-10.4; - 5.0) and 2.9% (-4.1; -1.8), respectively. From 1990 to 2019, stroke burden attributable to all risk factors combined increased in East Asia (from 29.6 million DALYs [26.2-33.8] to 42.0 million [36.4-47.7]), South Asia (from 12.0 million [10.7-13.4] to 21.3 million [18.9-23.8]), Southeast Asia (from 8.3 million [7.5-9.1] to 16.1 million [14.5-17.7]), North Africa and Middle East (from 3.7 million [3.3-4.2] to 6.8 million [6.0-7.7]), but decreased in Western Europe (from 7.1 million [6.6-7.5] to 4.4 million [3.9-4.9]), with no significant changes in other GBD regions (Supplement Figure 22). Similar geographical patterns in trends were observed for IS, ICH and SAH, but in East Asia there was a noticeable decrease in SAH related DALYs due to all risk factors combined (from 4.7 million [2.9-5.7] to 2.1 million [1.7-2.6]). Although there were no significant gender differences in the age-standardised percentage of DALYs due to metabolic and environmental/occupational risks for stroke and its pathological types, the share of DALYs due to behavioural risks was significantly higher for males than for females across all pathological types (Supplement Figures 24-26).

While the five leading risk factors for IS (Figure 5; Supplement Figure 16) were high SBP (32.5 million [51.2%]), ambient $PM_{2.5}$ pollution (16.6 million [18.8%]), high fasting plasma glucose (13.9 million [21.9%]), high LDL cholesterol (13.7 million [21.6%]), and high BMI (11.0 million [17.3%]); for ICH (Supplement Figure 17) these were high SBP (40.7 million [59.3%]), ambient $PM_{2.5}$ pollution (23.5 million [34.2%]), high BMI (19.9 million [29.0%]) and high fasting plasma glucose (13.1 million [19.0%]); for SAH (Supplement Figure 18) these were high SBP (6.4 million [56.9%]), high BMI (4.0 million [36.2%]), ambient $PM_{2.5}$ pollution (3.4 million [30.5%]), smoking (2.1 million [19.1%]) and high fasting plasma glucose (2.0 million [17.5%]). The effects of low daily temperatures on the burden of stroke (PAF of DALYs 5.8%) was almost six times greater than the effects of high daily temperatures (PAF DALYs 0.8%), and the effects of low daily temperatures on the burden of IS (6.0%), ICH (5.7%) and SAH (5.6%) were similar. The stroke burden associated with suboptimal temperature was primarily driven by low temperature in northern hemisphere, particularly in Eastern and Central Europe, Central and East Asia, and high temperature in southern hemisphere, particularly in Sub-Saharan Africa and South Asia (Supplement Figures 19-20). Metabolic risks accounted for the largest proportion of stroke-related DALYs (71.0% [64.6-77.1]), followed by behavioural (47.4% [41.3-54.4]) and environmental (37.8% [35.0-41.0]) risks, with no significant differences between clusters in age-standardised rates of DALYs by stroke pathological types (Supplement Tables 13-16).

Section 5. Supplementary tables

Supplement Table 1. Incident case, death, prevalent cases, and DALYs for stroke in 2019 and percentage change of age-standardised rates for 1990-2019, by location

Country, region	Incident cases (95% uncertainty interval)		Deaths (95% uncertainty interval)		Prevalent cases (95% uncertainty interval)		DALYs (95% uncertainty interval)	
	2019 counts	% change in age-standardised rates, 1990-2019	2019 counts	% change in age-standardised rates, 1990-2019	2019 counts	% change in age-standardised rates, 1990-2019	2019 counts	% change in age-standardised rates, 1990-2019
Countries categorised by the World Bank income level								
World Bank High Income	1,939,748 (1,756,753 - 2,152,249)	-33.4 (-35.4; -31.4)	915,274 (786,210 - 989,536)	-56.5 (-58.9; -54.4)	21,469,998 (19,663,626 - 23,527,138)	-13.7 (-15.7; -11.7)	15,753,691 (14,247,606 - 17,024,046)	-52.4 (-54.5; -50.4)
World Bank Low Income	612,041 (564,884 - 666,622)	-12.5 (-13.8; -11.1)	321,535 (273,078 - 372,234)	-17.6 (-26.3; -7.8)	4,946,391 (4,592,820 - 5,310,563)	-8.2 (-9.5; -7.0)	8,585,871 (7,346,520 - 9,955,933)	-21.8 (-30.5; -12.0)
World Bank Lower Middle Income	3,782,161 (3,447,570 - 4,190,679)	-11.6 (-12.6; -10.8)	2,076,761 (1,908,601 - 2,248,676)	-26.4 (-34.6; -17.0)	29,398,616 (26,844,817 - 31,983,436)	-3.7 (-4.7; -2.8)	51,615,008 (47,355,109 - 55,856,691)	-25.6 (-33.1; -17.8)
World Bank Upper Middle Income	5,884,090 (5,209,975 - 6,712,203)	-17.4 (-20.6; -14.1)	3,235,695 (2,883,733 - 3,569,740)	-40.6 (-47.9; -33.2)	45,603,348 (41,291,491 - 50,255,823)	-2.9 (-5.9; 0.2)	67,195,706 (60,574,579 - 73,873,713)	-42.0 (-48.7; -34.9)
GBD regions								
High-income Asia Pacific	506,162 (458,159 - 562,656)	-29.1 (-33.1; -25.1)	182,893 (146,663 - 203,865)	-68.9 (-71.7; -66.4)	5,134,296 (4,679,315 - 5,605,361)	-15.0 (-18.3; -12.2)	3,245,486 (2,834,640 - 3,581,420)	-61.8 (-64.3; -58.6)
Central Asia	143,310 (134,325 - 153,635)	-16.2 (-18.4; -13.9)	92,032 (84,415 - 100,195)	1.0 (-6.4; 9.4)	996,197 (920,977 - 1,078,354)	-11.9 (-14.6; -9.3)	2,204,424 (2,014,444 - 2,418,525)	-5.5 (-13.1; 3.1)

East Asia	4,068,858 (3,557,533 - 4,727,907)	-9.8 (-15.8; -3.9)	2,259,565 (1,949,809 - 2,589,414)	-39.9 (-50.4; -29.1)	29,726,595 (26,506,336 - 33,247,911)	12.0 (6.7; 17.5)	47,598,099 (41,485,659 - 54,050,918)	-41.5 (-50.6; -30.8)
Southeast Asia	1,328,397 (1,215,682 - 1,470,865)	-7.2 (-8.5; -5.8)	731,836 (657,500 - 796,580)	-14.6 (-24.8; -2.0)	10,423,817 (9,576,927 - 11,358,766)	-2.1 (-3.5; -0.9)	18,184,273 (16,289,870 - 19,884,314)	-16.8 (-26.5; -6.8)
South Asia	1,698,563 (1,524,228 - 1,894,096)	-10.2 (-11.4; -9.1)	978,851 (864,811 - 1,095,389)	-32.6 (-43.7; -20.5)	12,784,111 (11,530,963 - 14,080,878)	-0.6 (-2.0; 0.8)	24,119,348 (21,416,431 - 26,859,939)	-30.6 (-40.6; -19.8)
Southeast Asia, East Asia, and Oceania	5,415,436 (4,789,429 - 6,219,412)	-8.0 (-12.8; -3.2)	2,999,627 (2,667,721 - 3,332,462)	-34.5 (-45.0; -24.7)	40,296,566 (36,493,824 - 44,443,561)	9.3 (5.2; 13.7)	66,049,968 (59,613,210 - 72,976,502)	-35.8 (-44.5; -27.0)
Oceania	18,180 (17,015 - 19,501)	-6.8 (-8.8; -4.4)	8,225 (6,315 - 10,506)	-8.7 (-22.1; 7.6)	146,154 (137,845 - 154,960)	-2.2 (-4.5; 0.1)	267,596 (208,538 - 340,992)	-7.8 (-22.1; 9.4)
Australasia	30,178 (27,220 - 33,371)	-40.3 (-43.5; -36.6)	15,574 (13,088 - 17,165)	-57.2 (-60.5; -54.3)	287,249 (262,742 - 313,437)	-27.7 (-31.3; -24.3)	231,104 (206,435 - 252,045)	-57.2 (-59.7; -54.8)
Central Europe, Eastern Europe, and Central Asia	1,080,935 (973,641 - 1,205,856)	-27.9 (-28.9; -26.8)	761,667 (691,189 - 817,950)	-33.5 (-37.9; -29.2)	7,627,357 (6,942,936 - 8,379,498)	-19.2 (-20.5; -17.8)	14,351,863 (13,236,352 - 15,424,074)	-32.4 (-36.6; -28.0)
Central Europe	307,697 (277,953 - 341,580)	-34.6 (-36.0; -33.1)	214,399 (188,051 - 240,057)	-43.0 (-48.9; -36.8)	2,312,078 (2,099,093 - 2,553,976)	-24.7 (-26.4; -23.1)	3,687,846 (3,236,211 - 4,114,623)	-45.7 (-51.1; -39.8)
Eastern Europe	629,928 (557,738 - 715,756)	-26.5 (-27.7; -25.2)	455,235 (405,555 - 498,061)	-33.3 (-38.6; -27.4)	4,319,081 (3,898,590 - 4,800,849)	-17.4 (-19.2; -15.7)	8,459,592 (7,598,493 - 9,321,472)	-30.2 (-35.8; -23.9)
Western Europe	602,981 (545,934 - 666,264)	-42.2 (-44.2; -40.0)	370,209 (317,396 - 402,160)	-60.4 (-62.7; -58.6)	5,595,567 (5,119,468 - 6,111,661)	-26.5 (-28.8; -24.3)	5,280,690 (4,757,434 - 5,650,037)	-59.8 (-61.4; -58.2)
Central Latin America	217,716 (198,723 - 239,098)	-31.3 (-32.8; -29.8)	88,138 (76,011 - 101,402)	-41.6 (-48.8; -33.5)	2,415,449 (2,221,148 - 2,627,857)	-22.0 (-23.8; -20.4)	1,941,065 (1,698,856 - 2,228,842)	-40.8 (-48.1; -32.6)
Tropical Latin America	303,521 (271,617 - 340,464)	-43.0 (-44.2; -41.7)	134,334 (122,219 - 142,436)	-57.4 (-60.0; -55.2)	3,060,627 (2,787,822 - 3,359,816)	-33.8 (-35.4; -32.0)	2,934,469 (2,749,810 - 3,090,539)	-58.4 (-60.6; -56.4)
Southern Latin America	72,611 (67,102 - 78,896)	-35.2 (-37.5; -32.7)	40,136 (36,578 - 43,059)	-52.5 (-55.5; -49.4)	616,239 (571,043 - 665,583)	-26.0 (-28.6; -23.2)	789,879 (733,097 - 841,017)	-54.5 (-57.2; -51.9)
Andean Latin America	50,905 (46,863 - 55,293)	-23.7 (-26.1; -21.3)	20,214 (16,663 - 24,566)	-48.9 (-58.2; -36.1)	561,790 (522,419 - 600,216)	-17.5 (-19.7; -15.2)	470,939 (393,705 - 572,696)	-50.8 (-59.7; -39.9)

Latin America and Caribbean	638,076 (581,316 - 703,192)	-35.5 (-36.6; - 34.3)	281,734 (252,948 - 304,759)	-49.3 (-53.1; - 45.4)	6,641,685 (6,100,911 - 7,213,021)	-26.5 (-27.9; - 25.2)	6,196,637 (5,710,445 - 6,688,830)	-50.3 (-53.8; - 46.7)
High-income North America	498,551 (440,176 - 569,317)	-27.9 (-30.1; - 25.6)	207,980 (181,340 - 225,802)	-34.7 (-37.2; - 30.9)	7,703,036 (6,967,262 - 8,537,862)	-3.2 (-6.4; 0.6)	4,156,207 (3,766,200 - 4,520,027)	-29.5 (-32.0; - 26.8)
North Africa and Middle East	829,803 (758,352 - 912,812)	-5.4 (-7.4; -3.3)	312,220 (278,450 - 349,726)	-27.8 (-35.4; - 16.0)	7,323,421 (6,794,727 - 7,863,138)	-0.5 (-2.3; 1.1)	7,946,004 (7,060,209 - 8,870,766)	-32.0 (-39.1; - 23.3)
Sub-Saharan Africa	851,257 (777,108 - 933,624)	-13.9 (-15.2; - 12.7)	401,833 (353,062 - 455,389)	-18.3 (-28.0; - 8.0)	7,465,031 (6,847,818 - 8,100,991)	-8.4 (-9.4; - 7.5)	10,864,998 (9,479,712 - 12,395,068)	-22.8 (-31.8; - 12.2)
Central Sub-Saharan Africa	98,796 (90,494 - 108,019)	-16.6 (-18.9; - 14.2)	49,587 (38,629 - 61,829)	-18.6 (-33.5; - 0.4)	803,555 (743,583 - 865,491)	-13.3 (-15.7; - 10.9)	1,363,973 (1,090,394 - 1,684,468)	-23.0 (-37.0; - 5.6)
Southern Sub-Saharan Africa	91,734 (81,994 - 104,140)	-2.0 (-4.5; 0.2)	40,273 (36,950 - 43,592)	-3.5 (-12.6; 8.6)	845,201 (763,849 - 926,378)	-5.9 (-8.2; - 3.4)	945,582 (867,586 - 1,028,078)	-13.1 (-21.1; - 4.2)
Eastern Sub-Saharan Africa	309,262 (282,036 - 340,679)	-20.6 (-22.1; - 18.8)	154,975 (129,199 - 181,905)	-20.0 (-31.4; - 6.6)	2,771,568 (2,532,786 - 3,028,409)	-11.4 (-12.9; - 10.0)	4,185,931 (3,500,348 - 4,904,229)	-26.1 (-36.9; - 12.6)
Western Sub-Saharan Africa	351,464 (321,420 - 383,682)	-10.7 (-12.0; - 9.5)	156,998 (134,635 - 181,172)	-20.3 (-35.3; - 3.6)	3,044,707 (2,796,874 - 3,301,565)	-4.8 (-5.8; - 3.7)	4,369,513 (3,707,618 - 5,124,470)	-22.1 (-35.7; - 6.5)
Countries in alphabetical order								
Afghanistan	36,785 (33,645 - 40,296)	-7.8 (-11.8; - 3.2)	16,816 (11,747 - 21,934)	-18.7 (-36.1; - 1.6)	281,177 (260,881 - 302,740)	3.8 (-1.2; 8.0)	507,837 (375,947 - 659,772)	-21.8 (-39.4; - 3.6)
Albania	6,410 (5,956 - 6,974)	-15.2 (-18.7; - 11.7)	5,605 (4,345 - 7,057)	-22.8 (-39.7; - 3.8)	39,915 (36,763 - 43,250)	-8.2 (-12.1; - 4.0)	90,638 (71,163 - 113,569)	-28.1 (-43.7; - 10.2)
Algeria	61,052 (55,145 - 67,937)	-16.9 (-21.1; - 12.3)	24,811 (19,995 - 30,219)	-43.4 (-54.9; - 30.2)	549,736 (506,627 - 592,960)	-10.7 (-14.5; - 7.0)	543,945 (450,965 - 649,230)	-44.6 (-55.0; - 31.9)
American Samoa	91 (84 - 99)	-7.8 (-11.7; - 3.7)	41 (35 - 47)	-25.4 (-36.9; - 11.7)	871 (823 - 931)	-6.0 (-9.3; - 2.8)	1,106 (944 - 1,287)	-23.6 (-35.5; - 9.6)
Andorra	95 (85 - 106)	-30.9 (-34.8; - 27.1)	36 (28 - 47)	-41.2 (-57.4; - 22.1)	885 (818 - 966)	-18.9 (-22.6; - 15.1)	606 (489 - 748)	-39.0 (-54.3; - 21.6)
Angola	22,923 (20,919 - 25,326)	-24.0 (-27.4; - 20.4)	9,878 (8,034 - 12,289)	-22.9 (-38.6; - 0.4)	197,618 (181,778 - 213,195)	-13.7 (-17.1; - 10.3)	285,830 (231,089 - 353,003)	-29.6 (-44.5; - 9.6)
Antigua and Barbuda	106 (97 - 116)	-21.6 (-25.2; - 18.0)	64 (56 - 74)	-32.3 (-41.7; - 22.2)	1,101 (1,020 - 1,186)	-12.5 (-16.2; - 8.8)	1,314 (1,134 - 1,517)	-36.8 (-45.5; - 26.6)
Argentina	46,827 (43,243 - 51,001)	-36.8 (-39.6; - 33.8)	26,066 (23,537 - 28,163)	-53.3 (-57.0; - 49.4)	389,404 (360,319 - 421,852)	-26.7 (-30.2; - 23.1)	532,666 (490,060 - 572,433)	-55.0 (-58.1; - 51.6)

Armenia	4,624 (4,242 - 5,051)	-39.3 (-42.7; -35.9)	2,667 (2,239 - 3,111)	-46.4 (-54.2; -38.2)	36,150 (33,216 - 39,160)	-26.9 (-30.9; -22.5)	51,584 (44,336 - 59,853)	-46.4 (-53.7; -38.5)
Australia	25,524 (22,906 - 28,301)	-41.7 (-45.3; -37.4)	12,763 (10,612 - 14,186)	-58.2 (-61.8; -54.9)	242,448 (221,937 - 263,215)	-28.9 (-32.8; -25.2)	188,907 (168,628 - 207,363)	-58.0 (-60.7; -55.3)
Austria	13,290 (11,975 - 14,741)	-43.1 (-47.1; -38.5)	5,374 (4,601 - 5,972)	-71.8 (-74.4; -69.6)	130,632 (120,882 - 140,833)	-24.9 (-30.1; -19.3)	85,504 (76,441 - 93,113)	-67.8 (-70.2; -65.5)
Azerbaijan	18,000 (16,638 - 19,394)	10.3 (5.9; 14.8)	10,723 (8,893 - 12,677)	43.1 (21.4; 71.7)	123,039 (113,407 - 134,041)	0.1 (-4.9; 5.5)	240,728 (201,898 - 283,087)	13.8 (-3.2; 34.4)
Bahamas	434 (398 - 475)	-17.9 (-21.5; -14.3)	218 (180 - 262)	-29.2 (-42.0; -15.1)	4,466 (4,146 - 4,791)	-13.0 (-16.8; -9.4)	5,171 (4,285 - 6,291)	-28.6 (-41.7; -13.1)
Bahrain	1,236 (1,090 - 1,394)	-32.8 (-36.8; -28.7)	245 (200 - 310)	-48.5 (-58.4; -36.5)	14,539 (13,369 - 15,844)	-24.2 (-27.5; -20.8)	7,987 (6,663 - 9,690)	-52.5 (-61.2; -42.5)
Bangladesh	182,856 (169,382 - 198,508)	-2.7 (-6.3; 0.9)	158,806 (121,873 - 193,530)	-22.8 (-41.1; -3.2)	1,417,979 (1,303,988 - 1,554,853)	1.3 (-3.1; 5.8)	3,493,100 (2,725,562 - 4,277,692)	-29.0 (-46.0; -10.5)
Barbados	478 (436 - 527)	-16.1 (-20.2; -12.2)	326 (272 - 382)	-36.4 (-45.9; -25.5)	4,904 (4,531 - 5,325)	-10.7 (-14.7; -6.8)	5,828 (4,891 - 6,856)	-36.3 (-46.3; -24.6)
Belarus	28,386 (25,558 - 31,480)	-16.5 (-21.4; -11.3)	16,906 (13,621 - 20,842)	-18.7 (-34.9; 0.8)	192,844 (176,409 - 209,989)	-9.5 (-14.5; -3.4)	325,915 (264,784 - 400,857)	-21.6 (-36.6; -2.8)
Belgium	15,219 (13,827 - 16,848)	-40.6 (-44.1; -36.8)	9,326 (7,973 - 10,374)	-56.9 (-60.7; -53.4)	138,000 (127,150 - 150,212)	-25.7 (-29.7; -21.6)	134,777 (121,465 - 146,813)	-56.2 (-59.1; -53.4)
Belize	309 (282 - 340)	-11.3 (-14.8; -7.3)	138 (119 - 158)	-26.4 (-36.7; -15.3)	3,232 (2,978 - 3,489)	-2.6 (-6.9; 1.5)	3,302 (2,880 - 3,763)	-23.6 (-34.2; -11.8)
Benin	9,772 (8,983 - 10,647)	-9.9 (-13.3; -6.6)	4,810 (3,894 - 5,944)	-17.0 (-32.4; 2.4)	81,620 (75,187 - 87,902)	-8.3 (-11.8; -4.8)	128,910 (101,775 - 164,140)	-20.0 (-35.7; 0.2)
Bermuda	94 (85 - 104)	-29.4 (-33.0; -26.1)	45 (37 - 54)	-56.7 (-63.5; -47.8)	1,051 (971 - 1,139)	-19.7 (-23.4; -15.7)	766 (650 - 905)	-56.4 (-63.0; -48.4)
Bhutan	576 (529 - 627)	-25.8 (-28.8; -22.6)	338 (264 - 421)	-32.2 (-49.8; -5.2)	4,767 (4,410 - 5,149)	-15.3 (-18.9; -11.4)	7,511 (5,884 - 9,344)	-37.1 (-53.0; -13.2)
Bolivia (Plurinational State of)	9,481 (8,695 - 10,312)	-26.8 (-29.7; -23.9)	5,289 (3,866 - 6,992)	-37.1 (-50.6; -18.9)	97,552 (90,979 - 104,909)	-20.7 (-23.7; -17.2)	125,389 (92,479 - 165,195)	-43.2 (-55.2; -25.9)
Bosnia and Herzegovina	11,483 (10,173 - 13,037)	1.4 (-4.9; 6.9)	7,533 (6,158 - 9,245)	-9.5 (-25.3; 8.0)	84,587 (76,114 - 93,244)	3.8 (-2.3; 9.2)	132,205 (109,609 - 162,195)	-19.8 (-32.8; -4.0)

Botswana	2,594 (2,352 - 2,854)	-11.3 (-15.7; -6.5)	1,361 (1,003 - 1,795)	-18.8 (-39.6; 10.9)	23,325 (21,623 - 25,222)	-6.4 (-10.2; -2.3)	34,707 (25,139 - 45,727)	-22.1 (-43.0; 5.8)
Brazil	295,510 (264,161 - 331,954)	-43.5 (-44.7; -42.2)	131,007 (119,135 - 139,018)	-57.8 (-60.4; -55.5)	2,985,012 (2,716,617 - 3,280,844)	-34.2 (-35.8; -32.5)	2,861,723 (2,683,070 - 3,012,806)	-58.8 (-61.0; -56.8)
Brunei Darussalam	519 (471 - 573)	-43.6 (-46.3; -40.7)	142 (127 - 161)	-54.1 (-60.1; -44.1)	5,258 (4,844 - 5,775)	-32.5 (-35.7; -28.6)	4,151 (3,700 - 4,697)	-55.6 (-61.0; -47.1)
Bulgaria	32,386 (29,075 - 35,789)	-18.1 (-22.6; -13.2)	28,661 (23,655 - 34,062)	-23.7 (-36.8; -8.9)	211,510 (187,084 - 237,844)	-8.3 (-13.8; -2.8)	486,589 (401,134 - 581,039)	-28.2 (-40.5; -13.0)
Burkina Faso	14,367 (13,116 - 15,808)	-1.2 (-4.8; 2.3)	7,398 (6,104 - 8,851)	4.5 (-16.5; 29.2)	119,294 (109,867 - 129,409)	-3.4 (-6.9; 0.4)	218,935 (176,976 - 266,866)	0.9 (-19.6; 25.2)
Burundi	8,886 (8,082 - 9,827)	-36.7 (-39.4; -33.7)	4,604 (3,490 - 6,086)	-34.2 (-49.7; -13.7)	78,004 (71,332 - 85,009)	-29.2 (-32.0; -26.2)	130,610 (99,794 - 173,132)	-40.6 (-55.8; -19.8)
CÃ´te d'Ivoire	23,273 (21,463 - 25,205)	-10.4 (-14.0; -6.5)	8,617 (6,615 - 10,732)	-17.7 (-33.2; -0.2)	198,683 (184,245 - 214,035)	-10.6 (-14.0; -7.0)	262,129 (197,469 - 331,454)	-20.5 (-37.2; -2.4)
Cabo Verde	601 (555 - 654)	-4.0 (-7.8; 0.3)	337 (280 - 418)	26.8 (4.7; 61.7)	5,798 (5,391 - 6,215)	-2.7 (-6.5; 1.3)	7,089 (5,958 - 8,604)	3.5 (-13.6; 28.8)
Cambodia	24,922 (23,080 - 27,009)	-14.2 (-17.8; -10.5)	14,738 (12,026 - 17,130)	-17.7 (-35.3; -0.7)	171,429 (160,064 - 184,042)	-8.5 (-11.9; -4.6)	354,977 (291,936 - 419,668)	-26.0 (-41.8; -11.3)
Cameroon	23,849 (22,020 - 25,931)	2.8 (-0.8; 6.4)	10,838 (8,295 - 14,119)	-6.7 (-29.2; 22.4)	198,131 (183,725 - 212,515)	2.7 (-1.2; 6.7)	314,471 (236,111 - 414,416)	-7.2 (-31.0; 24.5)
Canada	42,740 (38,183 - 47,441)	-37.6 (-41.6; -33.2)	18,478 (15,467 - 20,623)	-50.5 (-54.8; -46.1)	610,452 (538,934 - 689,336)	-26.5 (-31.4; -21.7)	328,851 (289,879 - 364,015)	-47.6 (-51.1; -44.3)
Caribbean	65,933 (61,054 - 71,394)	-8.8 (-10.7; -7.0)	39,048 (33,390 - 44,746)	-23.2 (-32.7; -13.3)	603,818 (563,262 - 648,304)	-6.2 (-8.3; -4.2)	850,164 (726,573 - 984,569)	-23.4 (-33.6; -12.1)
Central African Republic	5,221 (4,818 - 5,683)	-4.8 (-8.3; -1.0)	2,858 (2,099 - 3,798)	-9.4 (-29.5; 16.8)	38,309 (35,603 - 41,053)	-4.9 (-8.5; -1.2)	87,822 (66,145 - 115,057)	-12.7 (-32.4; 13.7)
Chad	11,968 (10,903 - 13,098)	-1.8 (-5.5; 2.0)	5,711 (4,570 - 7,240)	-1.2 (-19.4; 20.5)	95,943 (88,473 - 103,851)	0.8 (-2.8; 4.5)	172,283 (136,973 - 217,559)	-2.8 (-22.1; 20.0)
Chile	20,287 (18,565 - 22,211)	-32.6 (-36.2; -28.5)	10,546 (9,280 - 11,477)	-51.2 (-55.5; -47.1)	181,806 (168,173 - 198,067)	-25.6 (-29.6; -21.4)	198,926 (183,187 - 214,311)	-53.8 (-57.2; -50.5)
China	3,935,182 (3,431,718 - 4,579,875)	-9.3 (-15.5; -3.3)	2,189,175 (1,885,901 - 2,513,772)	-39.8 (-50.7; -28.6)	28,760,186 (25,600,008 - 32,213,485)	13.2 (7.7; 19.1)	45,949,134 (39,813,511 - 52,335,529)	-41.6 (-50.9; -30.7)

Colombia	43,823 (40,101 - 47,778)	-44.6 (-47.5; -41.5)	16,943 (13,053 - 21,270)	-59.6 (-68.2; -49.0)	465,940 (430,706 - 502,987)	-33.8 (-36.8; -30.5)	348,758 (276,617 - 435,754)	-59.1 (-67.6; -48.5)
Comoros	866 (792 - 950)	-31.1 (-34.2; -27.6)	450 (350 - 569)	-27.3 (-43.6; 6.3)	7,909 (7,340 - 8,536)	-25.1 (-28.0; -22.0)	10,668 (8,226 - 13,517)	-32.4 (-48.7; 8.5)
Congo	5,402 (4,947 - 5,907)	-23.0 (-26.2; -19.4)	2,543 (1,982 - 3,252)	-29.8 (-44.2; -11.7)	46,386 (43,111 - 49,885)	-20.0 (-23.1; -16.8)	69,047 (53,625 - 89,661)	-36.0 (-49.7; -18.5)
Cook Islands	41 (37 - 45)	-8.1 (-12.4; -3.4)	15 (12 - 17)	-42.3 (-53.4; -26.0)	405 (379 - 433)	-1.5 (-4.9; 2.1)	366 (311 - 429)	-39.9 (-50.7; -24.1)
Costa Rica	4,180 (3,804 - 4,616)	-21.6 (-25.0; -17.7)	1,470 (1,137 - 1,817)	-44.4 (-55.9; -31.4)	46,766 (43,396 - 50,474)	-11.4 (-14.9; -7.5)	28,789 (23,364 - 35,317)	-43.1 (-53.6; -29.8)
Croatia	10,988 (9,891 - 12,276)	-37.4 (-41.1; -33.6)	7,558 (6,183 - 9,177)	-51.2 (-59.8; -41.0)	81,664 (74,063 - 89,623)	-27.7 (-32.1; -23.2)	122,493 (101,714 - 146,751)	-55.1 (-62.8; -46.2)
Cuba	18,889 (17,160 - 20,688)	-18.1 (-21.9; -14.5)	10,836 (8,857 - 13,019)	-25.5 (-37.9; -11.3)	180,551 (166,315 - 195,150)	-14.9 (-19.3; -10.7)	193,864 (160,048 - 230,908)	-31.4 (-42.7; -18.0)
Cyprus	1,222 (1,117 - 1,346)	-39.5 (-42.5; -36.1)	745 (643 - 921)	-62.5 (-68.1; -55.4)	12,405 (11,097 - 13,954)	-22.9 (-27.2; -18.3)	12,020 (10,563 - 14,057)	-62.6 (-67.6; -56.4)
Czechia	22,203 (19,765 - 24,863)	-52.2 (-55.7; -48.8)	11,530 (9,513 - 13,648)	-71.2 (-75.8; -66.1)	192,928 (176,849 - 209,159)	-38.0 (-42.0; -33.9)	194,218 (164,694 - 226,622)	-70.6 (-74.8; -65.9)
Democratic People's Republic of Korea	89,309 (81,605 - 97,969)	3.7 (-1.1; 8.9)	55,468 (47,128 - 64,489)	-9.4 (-25.5; 11.1)	552,253 (512,284 - 596,814)	-1.0 (-5.7; 3.6)	1,298,906 (1,082,994 - 1,532,006)	-8.1 (-26.0; 13.8)
Democratic Republic of the Congo	62,595 (57,377 - 68,549)	-14.4 (-17.7; -11.0)	33,103 (24,281 - 42,609)	-16.3 (-34.1; 6.3)	496,216 (457,261 - 538,270)	-13.9 (-17.3; -10.3)	890,800 (682,571 - 1,128,121)	-19.7 (-36.6; 2.0)
Denmark	7,749 (6,979 - 8,609)	-46.0 (-49.3; -42.8)	4,540 (3,901 - 5,033)	-52.3 (-56.8; -47.7)	71,554 (65,368 - 78,157)	-35.8 (-39.1; -32.5)	70,006 (63,125 - 75,976)	-54.9 (-58.3; -51.3)
Djibouti	1,151 (1,042 - 1,270)	-18.9 (-22.3; -15.0)	485 (361 - 654)	-17.5 (-36.7; 9.6)	11,012 (10,144 - 11,875)	-12.2 (-15.2; -8.7)	13,961 (10,156 - 18,992)	-22.5 (-40.8; 5.2)
Dominica	102 (93 - 112)	-7.2 (-10.9; -3.2)	67 (56 - 79)	-18.8 (-32.9; -2.6)	933 (861 - 1,012)	-6.0 (-9.9; -2.3)	1,218 (1,020 - 1,438)	-21.7 (-35.4; -4.5)
Dominican Republic	14,107 (13,043 - 15,354)	18.9 (14.3; 23.7)	8,140 (6,377 - 10,486)	12.7 (-13.0; 46.4)	126,630 (118,452 - 135,718)	12.5 (8.2; 16.8)	186,458 (141,549 - 244,767)	11.3 (-15.3; 47.2)
Ecuador	15,743 (14,519 - 17,126)	-15.2 (-19.0; -11.9)	6,091 (4,924 - 7,631)	-35.6 (-47.9; -19.1)	171,656 (159,370 - 184,809)	-12.9 (-16.3; -9.6)	143,379 (116,846 - 179,336)	-41.0 (-52.4; -25.4)

Egypt	152,702 (136,448 - 170,214)	9.6 (3.5; 16.5)	45,767 (33,557 - 63,157)	-22.6 (-42.6; 1.6)	1,269,834 (1,170,811 - 1,374,639)	18.5 (12.9; 23.9)	1,493,970 (1,146,402 - 1,946,512)	-35.5 (-50.0; -17.0)
El Salvador	5,153 (4,722 - 5,628)	-20.7 (-24.2; -16.8)	2,193 (1,669 - 2,838)	-52.2 (-64.0; -35.5)	52,018 (48,450 - 56,160)	-10.6 (-14.2; -6.8)	44,576 (34,607 - 57,965)	-57.0 (-67.2; -41.5)
Equatorial Guinea	876 (790 - 970)	-33.1 (-36.6; -29.0)	348 (249 - 465)	-42.4 (-61.5; -15.1)	8,662 (7,963 - 9,469)	-15.5 (-19.1; -11.6)	9,101 (6,570 - 12,560)	-51.2 (-66.9; -30.3)
Eritrea	5,145 (4,717 - 5,635)	-25.5 (-29.0; -22.0)	2,765 (2,107 - 3,559)	-12.9 (-36.6; 19.8)	44,951 (41,283 - 48,677)	-18.0 (-21.6; -14.4)	81,898 (61,623 - 105,927)	-23.2 (-43.2; 2.4)
Estonia	2,333 (2,107 - 2,600)	-56.2 (-59.2; -52.6)	1,193 (924 - 1,590)	-77.1 (-81.9; -69.4)	18,955 (17,284 - 20,598)	-40.4 (-45.1; -35.7)	21,096 (17,106 - 26,873)	-74.9 (-79.6; -68.5)
Eswatini	1,130 (1,024 - 1,247)	-8.0 (-12.4; -3.6)	599 (444 - 802)	-11.3 (-35.2; 20.6)	9,599 (8,939 - 10,401)	-2.2 (-6.0; 2.1)	15,327 (11,179 - 20,591)	-14.1 (-37.0; 18.4)
Ethiopia	56,717 (50,257 - 64,537)	-34.9 (-37.7; -31.8)	30,705 (24,056 - 37,442)	-40.6 (-57.6; -21.0)	553,518 (486,109 - 627,303)	-21.0 (-24.2; -17.9)	772,436 (612,458 - 930,617)	-49.9 (-64.8; -32.4)
Fiji	1,363 (1,251 - 1,495)	-11.9 (-15.8; -8.0)	626 (510 - 768)	-25.0 (-42.4; -1.0)	13,238 (12,360 - 14,191)	-5.2 (-8.6; -1.7)	18,098 (14,685 - 22,140)	-26.5 (-42.7; -4.3)
Finland	10,007 (9,061 - 10,977)	-29.5 (-34.0; -24.8)	5,551 (4,712 - 6,137)	-55.5 (-59.6; -51.2)	92,719 (85,833 - 101,067)	-22.6 (-27.6; -17.3)	82,574 (73,885 - 90,218)	-57.4 (-60.7; -54.0)
France	79,428 (71,593 - 87,544)	-30.3 (-35.0; -25.8)	44,813 (37,076 - 50,592)	-60.1 (-63.9; -56.7)	741,738 (681,457 - 804,592)	-19.2 (-24.2; -14.2)	637,048 (559,734 - 698,992)	-55.9 (-58.9; -52.9)
Gabon	1,781 (1,624 - 1,957)	-17.3 (-21.2; -13.2)	857 (672 - 1,033)	-26.9 (-42.6; -9.2)	16,364 (15,176 - 17,644)	-12.4 (-15.6; -8.9)	21,373 (16,743 - 26,214)	-31.6 (-47.2; -15.5)
Gambia	2,063 (1,892 - 2,237)	-1.7 (-5.4; 2.3)	955 (768 - 1,151)	5.5 (-18.3; 36.5)	17,777 (16,483 - 19,172)	-3.1 (-6.5; 0.6)	24,817 (19,481 - 30,301)	1.9 (-23.2; 34.5)
Georgia	10,693 (10,047 - 11,435)	-21.2 (-24.4; -17.8)	10,322 (8,721 - 11,979)	-29.1 (-40.3; -16.1)	66,134 (60,296 - 72,761)	-11.4 (-16.0; -7.0)	182,044 (155,407 - 210,639)	-28.5 (-39.2; -16.3)
Germany	135,705 (122,078 - 150,613)	-40.2 (-44.4; -36.4)	71,654 (62,278 - 78,561)	-63.0 (-66.2; -59.9)	1,317,688 (1,212,550 - 1,428,861)	-25.0 (-29.4; -20.9)	1,104,316 (994,301 - 1,207,389)	-61.1 (-63.8; -58.4)
Ghana	36,366 (33,496 - 39,551)	6.6 (2.0; 11.4)	17,201 (14,314 - 20,767)	-9.4 (-26.9; 12.1)	316,896 (294,357 - 340,317)	5.7 (1.3; 10.5)	457,633 (373,826 - 555,142)	-11.6 (-29.1; 10.9)
Greece	21,906 (19,939 - 24,071)	-41.2 (-44.7; -37.5)	20,416 (17,345 - 22,283)	-55.4 (-58.5; -52.3)	162,441 (144,573 - 183,029)	-27.9 (-32.0; -23.3)	261,346 (235,579 - 281,659)	-54.2 (-56.8; -51.3)

Greenland	86 (78 - 95)	-41.1 (-44.6; -37.4)	43 (36 - 50)	-51.4 (-60.0; -41.5)	1,149 (1,003 - 1,340)	-27.8 (-32.1; -23.3)	1,016 (854 - 1,187)	-51.4 (-59.5; -42.0)
Grenada	147 (135 - 161)	-20.9 (-25.0; -16.9)	91 (82 - 100)	-42.5 (-48.6; -36.2)	1,466 (1,344 - 1,595)	-11.5 (-15.5; -7.1)	1,948 (1,764 - 2,143)	-44.9 (-50.9; -38.7)
Guam	308 (282 - 338)	-4.0 (-8.8; 0.9)	93 (79 - 110)	-47.0 (-55.9; -35.0)	3,335 (3,136 - 3,558)	4.5 (0.7; 8.4)	2,758 (2,367 - 3,180)	-28.3 (-38.7; -15.2)
Guatemala	11,176 (10,271 - 12,188)	-31.1 (-34.3; -27.6)	4,749 (3,821 - 5,820)	-26.4 (-40.2; -8.9)	116,629 (107,069 - 126,514)	-19.9 (-23.8; -15.4)	115,586 (94,121 - 141,415)	-33.5 (-46.6; -16.9)
Guinea	10,922 (10,104 - 11,820)	8.0 (4.3; 11.7)	5,841 (4,662 - 7,201)	0.9 (-20.7; 27.4)	83,816 (77,802 - 89,952)	6.5 (2.3; 10.7)	159,322 (126,320 - 197,733)	-1.3 (-23.1; 25.8)
Guinea-Bissau	1,922 (1,781 - 2,059)	-8.8 (-12.3; -5.4)	874 (685 - 1,086)	-9.5 (-31.5; 21.7)	15,142 (14,038 - 16,288)	-5.8 (-8.8; -2.1)	27,101 (21,227 - 33,782)	-14.9 (-36.5; 14.5)
Guyana	1,202 (1,116 - 1,297)	-27.6 (-30.8; -24.5)	837 (663 - 1,048)	-45.4 (-57.6; -31.2)	10,338 (9,510 - 11,338)	-18.7 (-23.0; -14.1)	20,353 (15,927 - 25,854)	-48.9 (-60.9; -34.7)
Haiti	15,707 (14,573 - 16,850)	-5.6 (-8.9; -2.4)	9,997 (6,611 - 14,466)	-24.3 (-41.0; -1.9)	119,912 (110,805 - 131,482)	-6.7 (-10.4; -2.1)	270,977 (182,413 - 387,090)	-29.1 (-45.8; -7.1)
Honduras	6,075 (5,528 - 6,729)	-13.9 (-17.9; -9.9)	5,508 (4,427 - 7,078)	28.3 (2.0; 66.8)	63,697 (58,654 - 69,123)	-8.3 (-11.9; -4.3)	126,299 (100,271 - 162,179)	7.8 (-15.3; 39.6)
Hungary	26,262 (23,219 - 29,698)	-43.1 (-47.2; -38.9)	13,091 (10,871 - 15,385)	-61.0 (-67.2; -54.4)	214,386 (194,865 - 232,753)	-33.9 (-37.6; -29.5)	242,256 (205,012 - 283,147)	-60.8 (-66.3; -54.6)
Iceland	360 (323 - 402)	-41.0 (-44.8; -36.6)	148 (122 - 169)	-60.5 (-65.1; -55.6)	3,382 (3,126 - 3,669)	-26.1 (-29.3; -22.6)	2,294 (2,019 - 2,554)	-60.1 (-63.9; -56.0)
India	1,291,245 (1,150,668 - 1,453,046)	-8.8 (-10.0; -7.5)	699,078 (593,766 - 806,599)	-35.8 (-47.2; -23.4)	9,650,716 (8,640,566 - 10,685,387)	0.3 (-1.1; 1.8)	17,332,326 (14,914,500 - 19,804,175)	-33.4 (-44.0; -21.2)
Indonesia	642,943 (573,041 - 729,347)	7.8 (5.6; 10.0)	331,349 (282,004 - 367,066)	10.8 (-7.9; 29.8)	4,918,487 (4,417,107 - 5,483,594)	5.9 (3.9; 8.0)	8,407,229 (7,152,083 - 9,450,909)	-0.4 (-15.8; 14.8)
Iran (Islamic Republic of)	102,778 (90,115 - 117,821)	-16.7 (-18.1; -15.1)	40,912 (36,741 - 43,849)	-45.1 (-50.6; -35.4)	963,512 (859,232 - 1,079,662)	-13.3 (-15.9; -10.7)	884,768 (812,248 - 943,655)	-45.7 (-51.0; -38.3)
Iraq	60,403 (54,854 - 66,101)	-11.7 (-16.4; -6.7)	26,256 (21,422 - 31,075)	-13.7 (-30.2; 4.5)	520,023 (483,072 - 557,268)	-9.6 (-13.3; -5.4)	682,943 (548,026 - 826,272)	-19.4 (-35.5; -0.8)
Ireland	4,225 (3,859 - 4,621)	-46.6 (-50.2; -42.9)	2,363 (2,009 - 2,622)	-63.2 (-67.1; -59.7)	44,036 (39,576 - 49,773)	-30.9 (-35.3; -26.2)	36,872 (32,817 - 40,320)	-63.9 (-67.0; -61.3)

Israel	7,701 (6,895 - 8,552)	-40.6 (-44.1; -36.4)	3,350 (2,854 - 3,697)	-58.4 (-62.4; -54.6)	72,690 (66,706 - 78,991)	-24.7 (-28.2; -20.9)	55,440 (49,734 - 60,349)	-58.7 (-61.7; -55.9)
Italy	94,074 (83,223 - 106,720)	-44.6 (-48.4; -41.2)	67,293 (56,409 - 74,600)	-58.6 (-62.2; -56.4)	772,098 (689,726 - 873,841)	-27.2 (-30.2; -23.9)	869,625 (771,306 - 939,553)	-59.9 (-62.4; -58.2)
Jamaica	4,106 (3,783 - 4,454)	1.8 (-2.8; 6.6)	2,858 (2,334 - 3,440)	-22.4 (-36.5; -6.0)	35,728 (33,105 - 38,526)	6.5 (1.1; 11.6)	52,001 (42,476 - 62,991)	-24.6 (-38.9; -7.6)
Japan	405,022 (363,744 - 454,269)	-12.0 (-18.2; -6.1)	144,662 (114,371 - 162,568)	-65.3 (-68.2; -63.5)	4,027,066 (3,632,870 - 4,425,113)	-0.8 (-5.2; 3.6)	2,465,284 (2,128,349 - 2,727,502)	-52.8 (-55.5; -50.3)
Jordan	15,457 (13,809 - 17,262)	-26.2 (-30.9; -20.8)	3,367 (2,758 - 3,983)	-49.8 (-59.4; -39.2)	134,580 (123,260 - 145,467)	-23.4 (-27.7; -19.5)	86,118 (72,852 - 100,150)	-50.2 (-58.7; -41.1)
Kazakhstan	34,281 (31,568 - 37,312)	-26.1 (-30.0; -21.9)	23,367 (20,133 - 26,505)	-3.8 (-14.9; 8.4)	246,446 (227,207 - 268,784)	-19.4 (-23.8; -14.8)	512,353 (445,828 - 583,403)	-11.2 (-21.9; 1.5)
Kenya	39,746 (35,317 - 44,616)	-18.5 (-19.7; -17.2)	17,910 (13,423 - 21,924)	-3.4 (-14.3; 10.1)	389,572 (345,848 - 434,586)	-8.8 (-10.1; -7.7)	482,516 (372,271 - 590,749)	-5.6 (-17.0; 8.2)
Kiribati	241 (228 - 257)	-12.0 (-15.2; -8.7)	146 (116 - 178)	-16.5 (-34.0; 4.4)	1,865 (1,750 - 1,978)	-12.2 (-15.1; -9.2)	4,971 (3,955 - 6,052)	-20.9 (-37.4; -0.6)
Kuwait	4,005 (3,598 - 4,495)	-2.7 (-8.2; 3.3)	920 (757 - 1,093)	-9.6 (-24.2; 7.7)	42,739 (39,334 - 46,356)	-5.9 (-10.4; -1.4)	25,463 (21,787 - 29,824)	-11.7 (-24.0; 3.3)
Kyrgyzstan	7,851 (7,273 - 8,505)	-35.8 (-39.0; -32.6)	4,590 (4,059 - 5,201)	-36.1 (-43.6; -28.0)	53,369 (48,902 - 57,576)	-31.1 (-34.3; -27.0)	116,085 (102,459 - 131,889)	-35.8 (-43.1; -27.7)
Lao People's Democratic Republic	11,178 (10,412 - 12,078)	-20.0 (-23.4; -16.4)	6,197 (5,034 - 7,710)	-13.7 (-31.9; 9.6)	79,720 (74,529 - 85,761)	-16.2 (-19.4; -12.6)	165,435 (132,617 - 208,161)	-19.5 (-37.1; 2.2)
Latvia	6,990 (6,265 - 7,750)	-31.9 (-36.1; -27.6)	4,876 (4,114 - 5,776)	-45.2 (-52.7; -35.1)	42,211 (37,798 - 46,723)	-21.0 (-25.4; -16.8)	76,490 (65,754 - 89,911)	-46.6 (-53.5; -37.6)
Lebanon	7,800 (7,030 - 8,706)	-5.2 (-9.9; 0.3)	1,764 (1,236 - 2,298)	-39.5 (-55.5; -19.2)	74,841 (69,385 - 80,771)	2.2 (-1.8; 6.6)	39,216 (30,874 - 48,709)	-33.7 (-47.6; -16.0)
Lesotho	2,570 (2,382 - 2,788)	10.1 (5.5; 15.1)	1,758 (1,341 - 2,263)	24.2 (-7.2; 61.0)	19,914 (18,555 - 21,414)	17.8 (12.7; 23.2)	43,675 (33,387 - 56,560)	23.9 (-7.8; 62.5)
Liberia	3,814 (3,499 - 4,153)	-12.9 (-16.3; -9.7)	1,604 (1,241 - 2,080)	-21.1 (-37.7; -1.0)	32,976 (30,529 - 35,457)	-11.7 (-15.1; -8.3)	45,037 (34,458 - 58,444)	-26.2 (-41.5; -6.4)
Libya	9,358 (8,475 - 10,432)	7.9 (2.1; 14.6)	3,086 (2,364 - 4,016)	-17.9 (-36.4; 7.9)	90,351 (83,440 - 96,922)	14.0 (8.9; 18.7)	82,320 (64,181 - 104,686)	-21.3 (-37.5; 1.4)

Lithuania	9,369 (8,400 - 10,449)	-19.7 (-24.7; -14.0)	4,886 (4,079 - 5,794)	-21.5 (-33.1; -7.3)	58,188 (51,035 - 65,987)	-14.7 (-20.5; -9.2)	82,044 (69,915 - 96,553)	-29.0 (-38.7; -16.3)
Luxembourg	596 (536 - 658)	-54.4 (-57.9; -50.8)	348 (289 - 399)	-73.1 (-76.5; -69.2)	5,986 (5,435 - 6,670)	-41.1 (-45.3; -36.8)	5,258 (4,575 - 5,956)	-72.2 (-75.2; -68.7)
Madagascar	33,471 (31,037 - 36,260)	-7.6 (-11.2; -3.8)	15,710 (11,331 - 20,089)	-3.5 (-24.7; 24.9)	266,167 (246,785 - 285,034)	-7.5 (-10.6; -3.9)	484,557 (361,455 - 612,815)	-10.9 (-31.4; 14.9)
Malawi	13,333 (12,035 - 14,754)	-11.7 (-15.6; -8.1)	6,604 (5,288 - 8,095)	-17.4 (-32.7; 1.1)	123,528 (112,510 - 134,586)	-6.7 (-10.4; -2.8)	174,305 (139,279 - 214,896)	-20.7 (-36.2; -0.9)
Malaysia	47,911 (43,757 - 52,839)	-29.4 (-32.7; -26.0)	19,928 (15,909 - 25,000)	-42.4 (-53.6; -27.2)	443,995 (414,703 - 476,838)	-17.3 (-20.7; -14.1)	512,726 (420,450 - 629,695)	-45.0 (-54.5; -31.9)
Maldives	414 (380 - 455)	-45.3 (-48.0; -42.1)	155 (129 - 184)	-58.0 (-66.0; -48.3)	4,108 (3,782 - 4,433)	-33.5 (-36.5; -30.6)	3,948 (3,360 - 4,597)	-62.6 (-69.0; -55.5)
Mali	15,697 (14,369 - 17,080)	-17.0 (-20.0; -14.0)	7,878 (6,395 - 9,792)	-16.4 (-31.9; 1.9)	127,672 (118,236 - 137,634)	-13.5 (-16.9; -9.8)	226,520 (178,841 - 286,951)	-21.5 (-36.1; -2.5)
Malta	608 (548 - 676)	-39.0 (-42.6; -34.9)	329 (275 - 374)	-61.8 (-66.6; -56.8)	5,354 (4,800 - 6,068)	-29.0 (-33.4; -24.5)	5,096 (4,466 - 5,733)	-62.0 (-66.2; -57.5)
Marshall Islands	107 (99 - 114)	-5.6 (-8.8; -2.2)	50 (37 - 66)	-14.6 (-32.1; 6.0)	833 (784 - 884)	-2.3 (-5.4; 0.8)	1,665 (1,248 - 2,155)	-12.7 (-30.5; 9.5)
Mauritania	3,643 (3,338 - 3,964)	-26.0 (-29.3; -22.8)	1,425 (1,127 - 1,762)	-38.6 (-51.0; -23.7)	33,449 (31,016 - 35,994)	-21.7 (-24.7; -18.5)	35,642 (27,755 - 45,070)	-43.7 (-55.4; -28.6)
Mauritius	2,262 (2,073 - 2,503)	-48.3 (-51.1; -45.0)	1,031 (849 - 1,224)	-59.6 (-66.4; -52.4)	21,709 (20,223 - 23,298)	-35.5 (-38.2; -32.4)	24,722 (20,669 - 29,223)	-58.8 (-65.1; -51.7)
Mexico	107,719 (96,524 - 120,512)	-28.3 (-29.7; -26.8)	37,897 (32,830 - 42,992)	-41.6 (-48.9; -34.3)	1,269,751 (1,148,401 - 1,403,166)	-19.5 (-21.7; -17.6)	859,830 (752,346 - 973,650)	-38.1 (-45.3; -30.8)
Micronesia (Federated States of)	181 (169 - 194)	-16.8 (-19.6; -14.0)	107 (75 - 137)	-15.5 (-38.2; 12.5)	1,450 (1,364 - 1,541)	-13.9 (-16.9; -11.0)	3,278 (2,215 - 4,265)	-18.1 (-41.7; 10.8)
Monaco	73 (64 - 83)	-15.1 (-19.6; -10.6)	44 (34 - 53)	-55.8 (-65.2; -38.9)	648 (597 - 716)	-8.9 (-12.8; -4.5)	613 (501 - 734)	-51.9 (-61.6; -35.0)
Mongolia	6,175 (5,803 - 6,535)	-30.0 (-32.6; -27.2)	4,172 (3,285 - 5,387)	19.5 (-8.8; 53.4)	37,060 (34,081 - 40,146)	-15.6 (-19.9; -11.0)	119,585 (93,368 - 153,705)	4.9 (-20.2; 35.3)
Montenegro	2,135 (1,985 - 2,300)	-3.2 (-6.8; 0.6)	1,882 (1,611 - 2,141)	3.4 (-12.7; 19.6)	11,646 (10,733 - 12,690)	-4.9 (-8.9; -1.0)	31,759 (27,258 - 36,253)	-5.5 (-20.9; 9.9)

Morocco	63,692 (57,107 - 70,794)	-4.4 (-9.8; 1.5)	29,033 (23,331 - 35,135)	-11.9 (-29.1; 8.0)	557,119 (515,485 - 599,727)	3.4 (-0.7; 8.2)	666,284 (534,574 - 808,810)	-17.2 (-33.9; 0.9)
Mozambique	32,487 (29,742 - 35,544)	7.9 (3.8; 12.2)	15,979 (12,539 - 19,890)	8.5 (-16.2; 37.4)	255,218 (235,699 - 274,795)	13.0 (8.8; 17.7)	438,898 (340,278 - 550,913)	10.1 (-15.4; 41.4)
Myanmar	75,852 (69,687 - 82,564)	-18.6 (-22.1; -15.1)	82,399 (71,596 - 95,580)	-24.5 (-38.1; -8.3)	576,567 (536,016 - 620,546)	-8.7 (-12.7; -4.7)	1,951,121 (1,639,305 - 2,308,453)	-33.2 (-46.8; -16.2)
Namibia	2,349 (2,129 - 2,618)	-27.1 (-30.9; -23.1)	1,511 (1,236 - 1,857)	-21.4 (-38.2; 3.6)	20,928 (19,321 - 22,693)	-18.4 (-21.8; -14.8)	32,854 (26,367 - 41,257)	-26.8 (-43.6; -1.6)
Nauru	14 (13 - 15)	-3.5 (-7.3; 0.3)	7 (6 - 9)	-12.9 (-27.2; 4.8)	118 (111 - 125)	-4.6 (-7.7; -1.4)	287 (221 - 365)	-11.8 (-26.3; 5.9)
Nepal	19,035 (17,383 - 20,822)	-18.6 (-21.3; -15.4)	15,189 (11,964 - 18,675)	-25.2 (-44.9; -2.4)	146,771 (134,958 - 159,685)	-12.4 (-16.5; -8.4)	346,766 (272,933 - 432,724)	-31.4 (-48.6; -11.0)
Netherlands	23,174 (20,762 - 25,871)	-37.1 (-41.2; -33.1)	12,511 (10,733 - 13,992)	-49.0 (-53.4; -44.6)	216,712 (199,536 - 234,794)	-22.5 (-26.1; -18.8)	191,285 (171,363 - 209,122)	-49.6 (-52.9; -46.0)
New Zealand	4,654 (4,197 - 5,178)	-32.7 (-37.2; -28.1)	2,811 (2,343 - 3,136)	-51.7 (-56.5; -47.1)	44,801 (39,959 - 50,417)	-21.2 (-26.6; -15.5)	42,197 (37,643 - 46,161)	-53.2 (-56.6; -49.6)
Nicaragua	4,350 (3,940 - 4,770)	-28.3 (-31.9; -25.1)	1,939 (1,638 - 2,243)	-24.6 (-37.2; -7.9)	48,788 (45,019 - 52,500)	-18.6 (-22.2; -14.7)	41,428 (34,829 - 48,666)	-35.0 (-46.2; -20.4)
Niger	15,281 (13,938 - 16,792)	-11.7 (-15.1; -8.4)	7,143 (5,522 - 9,239)	-13.8 (-30.9; 7.0)	121,259 (111,188 - 131,350)	-11.8 (-15.1; -8.7)	220,243 (166,891 - 290,194)	-19.4 (-35.7; 2.1)
Nigeria	148,480 (132,163 - 166,210)	-17.5 (-19.0; -16.0)	63,432 (50,613 - 79,002)	-31.1 (-50.4; -5.0)	1,341,003 (1,196,421 - 1,484,666)	-6.1 (-7.6; -4.5)	1,706,180 (1,360,781 - 2,138,594)	-32.2 (-50.4; -7.7)
Niue	4 (4 - 4)	-9.0 (-13.0; -4.9)	2 (2 - 3)	-27.1 (-42.2; -9.6)	36 (33 - 38)	-4.8 (-8.1; -1.4)	56 (45 - 68)	-26.1 (-41.4; -7.0)
North Macedonia	7,529 (6,748 - 8,424)	-13.6 (-18.4; -8.8)	6,911 (5,784 - 8,206)	-5.4 (-20.2; 12.6)	50,331 (44,712 - 57,181)	-11.1 (-16.2; -5.8)	124,286 (102,852 - 148,837)	-16.1 (-29.8; 0.9)
Northern Ireland	2,217 (2,014 - 2,449)	-49.4 (-52.8; -45.8)	1,454 (1,259 - 1,605)	-55.3 (-60.1; -51.0)	19,556 (17,828 - 21,671)	-37.0 (-40.5; -33.3)	21,268 (19,159 - 23,071)	-57.6 (-61.2; -54.3)
Northern Mariana Islands	90 (83 - 99)	-17.4 (-20.8; -13.8)	39 (33 - 44)	-38.9 (-48.7; -27.6)	883 (827 - 945)	-13.9 (-17.1; -11.2)	1,136 (972 - 1,316)	-37.9 (-48.6; -25.7)
Norway	7,988 (7,048 - 9,139)	-35.3 (-38.3; -32.2)	3,311 (2,834 - 3,642)	-61.5 (-63.8; -58.6)	95,196 (85,083 - 106,535)	-5.6 (-8.7; -2.5)	53,377 (47,436 - 58,239)	-58.1 (-60.6; -55.5)

Oman	3,701 (3,308 - 4,161)	-10.3 (-15.9; -4.6)	1,030 (895 - 1,196)	-28.8 (-45.0; -3.7)	37,722 (34,606 - 41,220)	-8.5 (-12.4; -4.5)	30,859 (27,010 - 36,651)	-38.7 (-51.9; -20.7)
Pakistan	204,852 (183,765 - 227,527)	-7.8 (-10.0; -5.7)	105,440 (90,082 - 126,572)	-7.6 (-23.7; 18.6)	1,563,878 (1,412,116 - 1,723,323)	-0.9 (-3.8; 2.1)	2,939,645 (2,518,138 - 3,459,313)	-6.7 (-22.8; 18.7)
Palau	44 (40 - 48)	-0.5 (-5.0; 4.0)	21 (17 - 26)	-19.3 (-41.1; 8.0)	414 (387 - 443)	1.3 (-2.1; 4.9)	637 (512 - 786)	-18.1 (-40.1; 8.5)
Palestine	4,745 (4,261 - 5,288)	-7.6 (-13.7; -1.7)	2,019 (1,740 - 2,286)	-29.1 (-43.5; -10.1)	41,305 (37,846 - 45,067)	-3.7 (-8.5; 1.4)	44,902 (39,457 - 50,763)	-32.5 (-46.4; -15.0)
Panama	4,229 (3,886 - 4,596)	-25.9 (-29.7; -22.0)	1,808 (1,401 - 2,254)	-43.2 (-55.1; -28.6)	41,963 (39,100 - 45,182)	-13.1 (-16.6; -9.3)	34,108 (26,984 - 42,566)	-44.5 (-55.6; -30.3)
Papua New Guinea	12,835 (11,954 - 13,768)	-6.1 (-9.1; -2.6)	5,354 (3,675 - 7,396)	-1.3 (-20.5; 22.6)	100,401 (94,494 - 106,437)	-0.5 (-3.8; 3.0)	180,343 (126,283 - 244,402)	-1.8 (-21.6; 23.9)
Paraguay	8,011 (7,380 - 8,769)	-17.8 (-21.6; -14.0)	3,327 (2,539 - 4,341)	-38.4 (-52.6; -17.7)	75,615 (70,862 - 80,952)	-10.6 (-14.1; -7.5)	72,745 (56,703 - 95,374)	-38.6 (-52.6; -17.6)
Peru	25,681 (23,486 - 28,022)	-27.3 (-30.6; -23.8)	8,833 (6,557 - 11,906)	-59.0 (-69.9; -42.8)	292,582 (270,881 - 313,256)	-19.2 (-22.5; -15.8)	202,171 (156,558 - 265,849)	-59.0 (-69.1; -44.7)
Philippines	157,897 (141,489 - 178,001)	-4.2 (-5.3; -3.0)	72,488 (58,640 - 85,338)	27.2 (1.2; 53.2)	1,267,640 (1,150,933 - 1,393,790)	-2.1 (-3.7; -0.4)	2,042,700 (1,653,997 - 2,403,540)	48.9 (11.1; 79.3)
Poland	74,455 (65,183 - 86,225)	-34.5 (-36.0; -32.9)	45,105 (37,607 - 52,029)	-47.2 (-54.4; -39.8)	623,986 (558,862 - 704,818)	-24.3 (-26.5; -22.0)	808,827 (696,069 - 920,259)	-48.7 (-55.4; -42.0)
Portugal	18,186 (16,590 - 19,936)	-63.5 (-65.8; -60.9)	16,695 (14,403 - 18,231)	-73.0 (-75.1; -70.9)	166,193 (149,033 - 187,354)	-47.2 (-50.9; -42.9)	228,158 (206,863 - 245,265)	-72.9 (-74.6; -71.1)
Puerto Rico	4,229 (3,820 - 4,687)	-25.0 (-28.9; -20.5)	1,902 (1,459 - 2,340)	-50.3 (-60.7; -38.8)	55,064 (50,985 - 59,620)	-8.2 (-12.6; -3.8)	32,291 (25,866 - 39,541)	-44.7 (-55.2; -32.8)
Qatar	1,889 (1,659 - 2,171)	-26.1 (-30.3; -21.7)	181 (136 - 239)	-36.7 (-54.0; -15.7)	22,335 (20,296 - 24,525)	-22.2 (-25.6; -18.9)	8,998 (7,254 - 11,097)	-44.7 (-57.5; -29.5)
Republic of Korea	92,934 (83,777 - 102,520)	-64.3 (-66.5; -62.1)	36,509 (31,326 - 44,085)	-79.5 (-82.2; -72.2)	1,019,292 (935,025 - 1,128,951)	-50.5 (-53.3; -47.0)	735,531 (652,396 - 868,110)	-79.5 (-81.4; -72.3)
Republic of Moldova	9,413 (8,538 - 10,370)	-21.3 (-25.9; -17.2)	5,623 (4,938 - 6,341)	-41.4 (-48.2; -34.2)	59,557 (54,566 - 65,096)	-13.3 (-18.1; -8.8)	115,359 (102,368 - 129,792)	-38.7 (-45.5; -31.3)
Romania	66,930 (60,010 - 73,775)	-34.9 (-38.6; -31.0)	52,826 (44,794 - 61,798)	-34.3 (-43.5; -22.8)	452,322 (400,470 - 504,624)	-23.7 (-29.2; -19.2)	880,086 (757,348 - 1,031,383)	-36.3 (-45.5; -25.0)

Russian Federation	445,959 (391,471 - 510,396)	-28.1 (-29.2; -26.9)	327,885 (284,560 - 367,996)	-35.5 (-42.4; -28.2)	3,020,719 (2,721,682 - 3,381,698)	-20.0 (-21.7; -18.2)	5,981,008 (5,287,602 - 6,717,492)	-33.3 (-40.1; -25.3)
Rwanda	9,530 (8,730 - 10,462)	-47.6 (-50.1; -45.0)	5,339 (3,902 - 7,003)	-44.0 (-58.4; -29.2)	84,724 (77,758 - 92,200)	-37.3 (-40.3; -34.1)	136,689 (101,309 - 180,328)	-52.2 (-63.9; -39.1)
Saint Kitts and Nevis	95 (87 - 103)	-35.0 (-38.0; -31.8)	66 (56 - 76)	-51.8 (-58.1; -43.9)	953 (865 - 1,044)	-25.7 (-29.8; -21.4)	1,435 (1,173 - 1,699)	-54.2 (-61.8; -45.6)
Saint Lucia	273 (249 - 297)	-31.3 (-34.8; -28.2)	172 (147 - 200)	-47.6 (-54.7; -40.1)	2,681 (2,463 - 2,946)	-23.5 (-28.0; -19.3)	3,334 (2,860 - 3,877)	-47.9 (-55.4; -39.7)
Saint Vincent and the Grenadines	176 (162 - 192)	13.5 (8.4; 18.9)	109 (95 - 123)	-26.5 (-35.2; -16.6)	1,659 (1,535 - 1,790)	9.0 (4.2; 14.5)	2,212 (1,939 - 2,502)	-25.8 (-34.8; -14.6)
Samoa	333 (310 - 360)	-12.3 (-15.8; -8.4)	177 (145 - 216)	-21.6 (-36.8; -0.2)	2,880 (2,706 - 3,068)	-6.5 (-9.7; -3.4)	4,737 (3,772 - 5,832)	-20.0 (-37.4; 4.1)
San Marino	45 (41 - 50)	-24.7 (-28.5; -20.8)	29 (20 - 38)	-37.7 (-57.4; -13.3)	402 (369 - 440)	-15.1 (-18.9; -11.4)	398 (292 - 523)	-35.9 (-54.5; -12.8)
Sao Tome and Principe	285 (265 - 307)	5.2 (1.1; 9.6)	104 (84 - 130)	1.1 (-18.8; 29.4)	2,499 (2,332 - 2,676)	3.8 (0.3; 7.3)	2,802 (2,255 - 3,431)	-1.3 (-20.8; 27.6)
Saudi Arabia	41,061 (37,416 - 45,161)	11.9 (6.7; 18.0)	12,669 (9,750 - 15,359)	-32.5 (-49.1; -10.0)	480,500 (442,193 - 523,176)	7.9 (-0.1; 15.2)	417,599 (326,188 - 509,857)	-29.1 (-46.4; -5.6)
Scotland	9,071 (8,132 - 10,138)	-44.7 (-48.4; -40.2)	5,885 (5,125 - 6,407)	-53.9 (-57.7; -49.5)	120,587 (104,521 - 135,272)	-12.2 (-19.3; -4.9)	92,767 (84,266 - 101,047)	-53.0 (-56.4; -49.2)
Senegal	13,117 (12,038 - 14,334)	-14.2 (-17.5; -10.9)	6,030 (4,835 - 7,393)	-13.3 (-29.5; 7.5)	117,703 (108,765 - 126,600)	-13.0 (-16.5; -9.6)	157,808 (124,886 - 192,234)	-18.4 (-35.3; 2.0)
Serbia	30,995 (27,488 - 35,164)	-26.1 (-30.5; -21.7)	25,768 (21,693 - 30,515)	-28.3 (-41.6; -14.7)	218,355 (193,242 - 247,332)	-17.6 (-23.1; -12.4)	427,280 (360,197 - 510,891)	-38.5 (-50.1; -26.7)
Seychelles	194 (176 - 215)	-15.4 (-19.2; -11.2)	69 (60 - 81)	-35.8 (-43.3; -25.4)	1,701 (1,591 - 1,824)	-9.9 (-13.1; -6.6)	1,800 (1,579 - 2,076)	-36.5 (-43.3; -27.8)
Sierra Leone	8,228 (7,616 - 8,887)	-2.4 (-5.9; 1.4)	3,598 (2,718 - 4,729)	-8.2 (-27.7; 17.1)	69,156 (63,996 - 74,286)	-4.8 (-8.3; -1.3)	107,789 (79,985 - 143,399)	-10.4 (-30.8; 16.1)
Singapore	7,687 (6,845 - 8,612)	-56.3 (-60.1; -52.2)	1,580 (1,345 - 1,745)	-75.5 (-78.2; -73.2)	82,680 (76,610 - 89,734)	-45.8 (-48.6; -42.7)	40,519 (35,478 - 45,102)	-72.7 (-74.9; -70.6)
Slovakia	12,293 (10,975 - 13,884)	-30.5 (-35.1; -26.4)	5,881 (4,744 - 7,118)	-46.9 (-56.6; -35.4)	101,260 (93,163 - 108,846)	-22.3 (-25.8; -18.5)	115,130 (94,972 - 139,021)	-48.8 (-57.6; -38.2)

Slovenia	3,629 (3,268 - 4,076)	-48.5 (-51.9; -44.8)	2,050 (1,568 - 2,655)	-66.0 (-75.1; -54.9)	29,187 (26,809 - 31,902)	-38.9 (-42.5; -35.4)	32,080 (25,932 - 39,806)	-67.1 (-75.2; -57.6)
Solomon Islands	986 (922 - 1,059)	-9.3 (-12.3; -6.1)	792 (646 - 967)	4.5 (-18.6; 31.1)	6,916 (6,498 - 7,377)	-7.0 (-10.2; -3.7)	24,847 (20,075 - 30,436)	-1.4 (-23.7; 25.8)
Somalia	16,135 (14,671 - 17,873)	-21.5 (-24.5; -18.6)	7,851 (5,717 - 10,706)	-15.5 (-35.7; 14.2)	124,220 (113,848 - 135,025)	-15.9 (-19.0; -12.4)	240,029 (178,462 - 322,344)	-21.0 (-40.4; 8.4)
South Africa	70,796 (62,291 - 81,244)	-5.0 (-7.7; -2.6)	29,770 (27,397 - 31,794)	-3.5 (-13.0; 10.1)	664,089 (595,153 - 737,237)	-9.8 (-12.6; -7.1)	677,721 (624,202 - 726,582)	-17.1 (-24.8; -8.6)
South Sudan	7,748 (7,039 - 8,571)	-16.9 (-20.5; -13.4)	2,970 (2,041 - 4,058)	-25.9 (-45.0; 1.1)	72,034 (66,521 - 78,027)	-9.6 (-12.8; -6.4)	81,691 (57,503 - 111,636)	-29.8 (-47.9; -4.2)
Spain	61,102 (54,810 - 68,024)	-49.4 (-52.9; -45.6)	37,092 (30,981 - 42,048)	-67.5 (-70.1; -64.6)	552,068 (507,107 - 599,160)	-34.4 (-38.2; -30.5)	512,380 (455,521 - 562,180)	-66.2 (-68.4; -63.8)
Sri Lanka	32,480 (29,336 - 36,011)	-22.7 (-26.8; -18.2)	14,364 (10,917 - 18,505)	-42.6 (-56.1; -26.0)	300,522 (278,438 - 326,897)	-11.2 (-14.4; -7.3)	317,412 (248,001 - 401,417)	-39.7 (-52.8; -24.0)
Suriname	963 (891 - 1,040)	-6.7 (-10.4; -2.4)	573 (483 - 675)	-16.2 (-29.2; -1.2)	8,421 (7,833 - 9,075)	-2.7 (-6.6; 0.9)	12,949 (10,835 - 15,351)	-17.4 (-30.8; -1.8)
Sweden	15,721 (13,680 - 18,089)	-31.6 (-34.8; -27.8)	8,402 (7,274 - 9,444)	-47.3 (-51.5; -42.0)	176,295 (157,874 - 198,034)	-6.0 (-11.2; -0.4)	123,167 (111,061 - 134,983)	-46.8 (-50.2; -43.2)
Switzerland	9,792 (8,705 - 10,864)	-45.8 (-49.4; -42.1)	4,881 (3,995 - 5,629)	-64.1 (-67.8; -59.6)	93,345 (86,302 - 101,899)	-30.9 (-34.6; -26.9)	69,043 (60,495 - 76,970)	-62.6 (-65.7; -59.3)
Syrian Arab Republic	22,164 (20,152 - 24,408)	-18.6 (-22.5; -14.7)	9,186 (7,117 - 11,838)	-32.2 (-50.2; -8.6)	199,599 (184,381 - 215,112)	-13.9 (-17.3; -10.5)	238,333 (184,920 - 304,410)	-40.2 (-55.3; -19.9)
Taiwan (Province of China)	44,367 (39,859 - 49,520)	-44.7 (-48.5; -40.9)	14,923 (11,918 - 18,521)	-70.3 (-76.1; -63.0)	414,156 (384,049 - 447,445)	-23.7 (-27.7; -19.7)	350,060 (286,818 - 422,817)	-65.3 (-71.0; -58.3)
Tajikistan	7,734 (7,054 - 8,394)	-3.1 (-7.0; 0.7)	5,907 (4,785 - 7,220)	36.4 (12.5; 64.1)	50,985 (46,786 - 55,210)	-6.3 (-10.9; -1.6)	146,642 (117,926 - 178,933)	17.7 (-3.4; 42.4)
Thailand	123,118 (112,514 - 134,892)	-34.5 (-37.8; -31.2)	51,025 (38,708 - 65,560)	-50.4 (-62.6; -34.6)	1,124,258 (1,048,888 - 1,205,450)	-19.2 (-22.5; -15.7)	1,273,783 (985,697 - 1,609,779)	-44.3 (-56.6; -27.9)
Timor-Leste	2,122 (1,956 - 2,317)	1.5 (-2.5; 5.7)	1,135 (875 - 1,464)	20.3 (-5.4; 50.5)	15,405 (14,366 - 16,546)	4.2 (0.7; 8.1)	28,395 (21,811 - 36,615)	15.8 (-11.0; 44.0)
Togo	7,814 (7,232 - 8,458)	-6.4 (-10.0; -2.9)	3,200 (2,520 - 4,033)	-10.8 (-27.5; 11.9)	65,849 (61,114 - 71,061)	-8.2 (-11.3; -4.7)	94,740 (73,301 - 119,862)	-13.2 (-29.7; 9.6)

Tokelau	2 (2 - 3)	-13.0 (-16.7; -9.5)	1 (1 - 2)	-29.5 (-44.4; -8.8)	21 (20 - 23)	-4.9 (-8.0; -1.3)	35 (27 - 44)	-28.9 (-44.5; -6.1)
Tonga	124 (113 - 135)	-11.9 (-15.9; -8.3)	58 (48 - 69)	-12.8 (-30.7; 12.3)	1,163 (1,086 - 1,245)	-7.1 (-10.7; -3.7)	1,358 (1,143 - 1,619)	-13.5 (-30.0; 9.5)
Trinidad and Tobago	2,113 (1,936 - 2,326)	-27.5 (-30.7; -24.2)	1,189 (919 - 1,526)	-48.7 (-60.2; -34.5)	22,619 (20,898 - 24,507)	-17.6 (-21.0; -13.7)	23,993 (18,676 - 30,879)	-48.3 (-59.6; -33.3)
Tunisia	19,659 (17,619 - 22,111)	-1.2 (-6.6; 4.3)	8,713 (6,627 - 11,147)	-25.0 (-43.8; -0.6)	159,928 (147,442 - 173,396)	22.1 (16.3; 28.2)	176,891 (135,395 - 225,499)	-24.7 (-42.8; -2.0)
Turkey	125,345 (114,547 - 138,123)	-2.8 (-7.6; 1.9)	48,947 (39,204 - 59,511)	-16.5 (-39.5; 6.2)	1,080,380 (1,002,616 - 1,164,932)	-8.7 (-12.4; -4.7)	993,082 (820,881 - 1,177,528)	-23.5 (-42.5; -5.2)
Turkmenistan	9,529 (8,785 - 10,347)	0.5 (-3.8; 4.9)	5,263 (4,280 - 6,433)	8.6 (-11.2; 31.8)	63,998 (59,612 - 68,712)	9.2 (4.8; 14.0)	142,895 (115,819 - 173,399)	11.0 (-9.1; 34.8)
Tuvalu	21 (19 - 22)	-9.4 (-12.8; -6.0)	14 (11 - 18)	-25.6 (-43.8; -0.1)	174 (164 - 185)	-5.8 (-9.2; -2.6)	376 (295 - 477)	-27.6 (-45.1; -1.6)
Uganda	26,295 (23,670 - 29,014)	-23.7 (-27.2; -20.1)	12,053 (9,486 - 14,789)	-13.9 (-32.2; 6.1)	237,405 (217,386 - 261,003)	-13.0 (-16.4; -9.1)	326,455 (261,865 - 404,967)	-17.6 (-34.2; 4.0)
Ukraine	127,477 (112,612 - 145,969)	-24.3 (-27.1; -21.4)	93,866 (81,076 - 108,146)	-29.0 (-37.7; -18.6)	926,607 (830,577 - 1,026,787)	-11.3 (-17.4; -5.0)	1,857,680 (1,621,488 - 2,135,813)	-20.0 (-29.7; -7.3)
United Arab Emirates	14,482 (12,788 - 16,501)	-14.8 (-19.2; -9.9)	2,168 (1,544 - 3,026)	-50.0 (-62.0; -34.9)	136,808 (125,534 - 148,340)	-7.5 (-11.6; -3.6)	95,518 (70,890 - 128,945)	-46.0 (-58.5; -30.5)
United Kingdom	74,187 (66,075 - 83,805)	-45.0 (-47.0; -42.8)	50,633 (44,199 - 54,038)	-56.0 (-58.4; -53.4)	718,222 (644,317 - 801,010)	-27.9 (-30.5; -25.5)	734,883 (670,862 - 780,578)	-56.4 (-58.3; -54.3)
United Republic of Tanzania	45,517 (41,341 - 50,347)	-5.3 (-9.4; -0.6)	22,759 (18,011 - 28,916)	-8.8 (-25.6; 15.4)	408,522 (375,486 - 443,166)	3.4 (-0.6; 7.6)	579,724 (453,914 - 737,212)	-16.7 (-33.0; 5.3)
United States of America	455,717 (401,258 - 521,734)	-26.8 (-29.1; -24.3)	189,456 (165,594 - 205,369)	-32.8 (-35.5; -29.0)	7,091,313 (6,410,184 - 7,849,128)	-0.5 (-4.1; 3.6)	3,826,274 (3,474,482 - 4,163,113)	-27.4 (-30.0; -24.6)
United States Virgin Islands	171 (156 - 189)	-2.7 (-6.4; 1.7)	96 (84 - 109)	-15.7 (-29.5; 0.8)	1,654 (1,525 - 1,792)	0.4 (-3.8; 4.4)	1,949 (1,684 - 2,210)	-18.1 (-32.6; -1.0)
Uruguay	5,493 (4,981 - 6,080)	-25.5 (-29.8; -21.6)	3,523 (3,073 - 3,865)	-45.4 (-49.7; -40.9)	44,997 (41,188 - 49,640)	-20.9 (-24.8; -16.6)	58,247 (52,842 - 62,994)	-47.8 (-51.6; -43.7)
Uzbekistan	44,423 (40,922 - 48,282)	-2.5 (-7.7; 3.1)	25,022 (21,394 - 29,330)	39.7 (21.7; 60.3)	319,017 (291,316 - 350,035)	-1.6 (-7.5; 4.6)	692,508 (591,463 - 808,671)	21.3 (5.0; 39.7)

Vanuatu	537 (504 - 576)	-10.0 (-13.0; -6.9)	293 (223 - 400)	-5.3 (-24.9; 24.9)	4,247 (3,992 - 4,534)	-7.6 (-10.7; -4.3)	8,905 (6,726 - 12,036)	-3.2 (-24.8; 28.5)
Venezuela (Bolivarian Republic of)	31,011 (28,485 - 33,920)	-25.5 (-28.9; -22.3)	15,632 (12,268 - 19,933)	-25.9 (-42.0; -6.5)	309,898 (289,103 - 332,949)	-19.0 (-22.2; -15.6)	341,691 (267,994 - 436,702)	-28.5 (-43.7; -8.9)
Viet Nam	205,362 (189,887 - 222,504)	2.0 (-2.2; 6.2)	135,999 (110,645 - 160,272)	-10.6 (-31.0; 17.8)	1,484,621 (1,380,218 - 1,604,865)	7.6 (3.5; 12.4)	3,076,200 (2,515,064 - 3,673,875)	-13.0 (-34.3; 11.9)
Yemen	32,051 (29,342 - 34,986)	-9.5 (-14.0; -4.7)	14,375 (10,909 - 18,865)	-22.1 (-38.2; 0.7)	260,915 (242,029 - 280,821)	0.8 (-4.2; 5.7)	388,486 (302,567 - 500,074)	-24.5 (-41.0; -1.9)
Zambia	11,987 (10,789 - 13,284)	-13.5 (-17.3; -9.7)	8,668 (7,004 - 10,662)	15.7 (-11.5; 46.9)	112,567 (102,745 - 122,710)	-7.2 (-11.0; -3.1)	228,148 (182,185 - 283,513)	9.5 (-16.2; 40.4)
Zimbabwe	12,295 (11,215 - 13,584)	23.6 (17.1; 30.6)	5,273 (4,170 - 6,754)	7.8 (-12.7; 35.0)	107,347 (98,146 - 115,803)	17.6 (12.1; 22.7)	141,298 (112,474 - 179,524)	12.1 (-9.4; 39.1)

Supplement Table 2. Absolute number (in millions) and age-standardised rates per 100,000 people per year, with 95% uncertainty intervals (UI), of incident and prevalent stroke, deaths from stroke and DALYs due to stroke in 2019 and percentage change in the metrics for 1990-2019, by sex and pathological types of stroke

Pathological types of stroke by sex		Incidence (95% UI)		Deaths (95% UI)		Prevalence (95% UI)		DALYs (95% UI)	
		Metric in 2019	Percentage change, 1990-2019	Metric in 2019	Percentage change, 1990-2019	Metric in 2019	Percentage change, 1990-2019	Metric in 2019	Percentage change, 1990-2019
Ischaemic stroke<br><br>Males	Absolute number	3.45 (2.96-4.03)	98.0% (93.0-103.0)	1.57 (1.42-1.72)	78.0% (54.0-95.0)	33.22 (29.47-37.57)	103.0% (99.0-109.0)	31.81 (28.61-34.75)	69.0% (49.0-85.0)
	Age-standardised rate	90.9 (78.5-106.6)	-7.0% (-8.0; -5.0)	48.4 (43.7-52.6)	-29.0% (-37.0; -23.0)	863.5 (768.0-974.6)	1.0% (-1.0; 2.0)	878.5 (793.5-956.7)	-25.0% (-34.0; -18.0)
Females	Absolute number	4.19 (3.61-4.91)	80.0% (75.0-84.0)	1.72 (1.51-1.89)	48.0% (34.0-65.0)	43.98 (39.35-49.10)	90.0% (86.0-93.0)	31.67 (28.28-34.77)	46.0% (34.0-60.0)
	Age-standardised rate	97.2 (84.1-113.9)	-12.0% (-14.0; -9.0)	39.1 (34.3-43.0)	-38.0% (-43.0; -31.0)	1,025.5 (918.5-1,144.7)	-3.0% (-5.0; -1.0)	726.3 (648.7-798.3)	-32.0% (-37.0; -25.0)
Intracerebral haemorrhage<br><br>Males	Absolute number	1.83 (1.60-2.10)	47.0% (44.0-50.0)	1.57 (1.41-1.72)	46.0% (27.0-64.0)	10.71 (9.30-12.17)	63.0% (61.0-65.0)	39.34 (35.42-42.95)	33.0% (17.0-50.0)
	Age-standardised rate	47.2 (41.4-53.9)	-28.0% (-30.0; -27.0)	42.9 (38.6-46.9)	-33.0% (-41.0; -24.0)	262.7 (229.4-298.1)	-15.0% (-16.0; -13.0)	1,001.1 (902.0-1,092.0)	-33.0% (-42.0; -25.0)
Females	Absolute number	1.58 (1.38-1.81)	39.0% (36.0-41.0)	1.32 (1.17-1.45)	29.0% (12.0-48.0)	9.96 (8.70-11.26)	52.0% (50.0-54.0)	29.24 (26.33-32.33)	16.0% (2.0-32.0)
	Age-standardised rate	36.8 (32.2-42.2)	-31.0% (-32.0; -29.0)	30.1 (26.8-33.2)	-39.0% (-47.0; -30.0)	235.2 (205.8-265.8)	-19.0% (-20.0; -17.0)	676.8 (610.1-741.1)	-41.0% (-48.0; -33.0)
Subarachnoid haemorrhage	Absolute number	0.51 (0.43-0.60)	60.0% (56.0-64.0)	0.19 (0.16-0.23)	-7.0% (-28.0; 89.0)	3.33 (2.83-3.92)	64.0% (61.0-67.0)	5.73 (4.91-7.08)	-10.0% (-28.0; 64.0)

Males	Age-standardised rate	13.0 (11.1-15.3)	-17.0% (-19.0; -15.0)	5.0 (4.3-6.1)	-55.0% (-65.0; -8.0)	82.2 (70.1-96.5)	-11.0% (-13.0; -10.0)	144.7 (123.9-178.4)	-52.0 (-62.0; -11.0)
Females	Absolute number	0.67 (0.57-0.79)	61.0% (57.0-65.0)	0.19 (0.17-0.21)	-16.0% (-29.0; 1.0)	5.06 (4.34-5.93)	65.0% (60.0-69.0)	5.45 (4.85-6.14)	-17.0% (-28.0; -2.0)
	Age-standardised rate	15.7 (13.4-18.3)	-17.0% (-19.0; -15.0)	4.3 (3.8-4.9)	-59.0% (-65.0; -51.0)	118.5 (101.6-138.4)	-14.0% (-17.0; -12.0)	128.2 (113.9-144.4)	-56.0% (-62.0; -48.0)
Total stroke<br><br>Males	Absolute number	5.79 (5.24-6.45)	75.0% (72.0-79.0)	3.33 (3.04-3.62)	54.0% (37.0-71.0)	45.04 (41.13-49.26)	91.0% (88.0-95.0)	76.88 (70.21-83.45)	40.0% (25.0-55.0)
	Age-standardised rate	151.1 (136.9-167.5)	-15.0% (-17.0; -14.0)	96.4 (87.6-104.2)	-33.0% (-40.0; -26.0)	1,150.2 (1,052.7-1,259.3)	-4.0% (-5.0; -3.0)	2,024.3 (1,852.4-2,195.6)	-32.0% (-39.0; -25.0)
Females	Absolute number	6.44 (5.81-7.17)	66.0% (62.0-69.0)	3.22 (2.86-3.54)	34.0% (21.0-50.0)	56.44 (51.95-61.53)	81.0% (78.0-84.0)	66.36 (60.53-72.26)	24.0% (13.0-37.0)
	Age-standardised rate	149.8 (135.6-166.6)	-18.0% (-19.0; -16.0)	73.5 (65.2-80.7)	-40.0% (-46.0; -33.0)	1,316.7 (1,210.7-1,433.7)	-7.0% (-9.0; -6.0)	1,531.3 (1,397.1-1,667.6)	-39.0% (-44.0; -32.0)

Supplement Table 3. Incident case, death, prevalent cases, and DALYs for ischaemic stroke in 2019 and percentage change of age-standardised rates for 1990-2019, by location

Country, region	Incident cases (95% uncertainty interval)		Deaths (95% uncertainty interval)		Prevalent cases (95% uncertainty interval)		DALYs (95% uncertainty interval)	
	2019 counts	% change in age-standardised rates, 1990-2019	2019 counts	% change in age-standardised rates, 1990-2019	2019 counts	% change in age-standardised rates, 1990-2019	2019 counts	% change in age-standardised rates, 1990-2019
Countries categorised by the World Bank income level								
World Bank High Income	1,250,175 (1,079,490 - 1,449,816)	-39.3 (-41.3; -37.0)	589,915 (496,345 - 644,595)	-61.0 (-63.6; -59.0)	16,808,338 (14,986,111 - 18,843,157)	-16.1 (-18.7; -13.5)	9,137,365 (8,085,309 - 10,025,961)	-55.8 (-58.2; -53.5)
World Bank Low Income	324,784 (282,894 - 372,576)	-0.3 (-2.1; 1.6)	105,226 (88,398 - 125,146)	0.0 (-11.9; 12.7)	3,605,103 (3,238,263 - 3,957,265)	-1.7 (-3.2; -0.4)	2,455,709 (2,117,328 - 2,893,725)	-2.3 (-12.7; 8.5)
World Bank Lower Middle Income	1,890,653 (1,634,224 - 2,191,231)	-6.6 (-8.2; -5.0)	888,453 (792,058 - 983,989)	-20.4 (-30.4; -8.5)	19,886,816 (17,496,012 - 22,232,177)	0.7 (-0.6; 2.1)	18,608,312 (16,612,978 - 20,667,519)	-16.9 (-26.7; -6.5)
World Bank Upper Middle Income	4,161,520 (3,508,892 - 4,931,224)	2.1 (-0.9; 5.1)	1,708,178 (1,522,406 - 1,878,177)	-28.4 (-37.8; -20.3)	36,852,495 (32,321,863 - 41,736,487)	7.3 (3.1; 12.1)	33,246,047 (29,880,152 - 36,557,845)	-26.0 (-35.1; -18.2)
GBD regions								
High-income Asia Pacific	257,816 (221,003 - 303,061)	-43.4 (-46.6; -39.8)	114,703 (89,698 - 129,657)	-71.6 (-74.6; -69.3)	3,497,268 (3,061,550 - 3,990,297)	-21.1 (-24.9; -17.7)	1,796,502 (1,518,306 - 2,028,424)	-63.6 (-66.5; -60.6)
Central Asia	74,165 (65,232 - 83,701)	-14.4 (-18.2; -10.0)	41,335 (37,635 - 45,234)	1.8 (-11.9; 15.7)	641,824 (573,076 - 712,970)	-10.9 (-14.4; -7.2)	883,961 (807,145 - 973,323)	-4.2 (-17.0; 8.3)
East Asia	2,946,609 (2,441,806 - 3,547,534)	32.6 (27.6; 37.9)	1,055,199 (906,555 - 1,202,264)	-4.8 (-26.1; 14.1)	24,924,020 (21,474,813 - 28,619,790)	31.5 (23.9; 39.9)	21,980,475 (19,272,756 - 24,994,300)	-5.1 (-23.1; 10.5)

Southeast Asia	690,481 (589,908 - 806,834)	4.0 (2.0; 6.0)	301,162 (257,707 - 336,635)	4.0 (-11.3; 23.4)	6,685,553 (5,898,978 - 7,569,000)	4.4 (3.0; 5.9)	6,327,312 (5,438,881 - 7,129,649)	3.2 (-9.7; 18.1)
South Asia	708,147 (601,267 - 831,177)	-4.5 (-6.0; -3.0)	377,820 (321,486 - 443,288)	-22.8 (-37.9; -4.8)	8,311,773 (7,164,911 - 9,433,859)	4.6 (3.0; 6.0)	7,745,870 (6,612,010 - 9,077,243)	-19.4 (-34.1; -4.0)
Southeast Asia, East Asia, and Oceania	3,643,279 (3,051,972 - 4,351,987)	26.8 (23.0; 30.9)	1,357,923 (1,194,252 - 1,518,966)	-3.1 (-24.2; 14.4)	31,681,746 (27,554,149 - 36,036,363)	25.9 (19.8; 32.5)	28,355,440 (25,100,159 - 31,773,471)	-3.2 (-21.4; 10.0)
Oceania	6,189 (5,344 - 7,096)	-1.8 (-6.0; 2.3)	1,562 (1,217 - 2,090)	-1.0 (-15.8; 17.2)	72,173 (65,863 - 78,552)	0.4 (-2.0; 2.7)	47,652 (38,121 - 61,541)	-1.1 (-13.2; 13.6)
Australasia	20,944 (18,122 - 23,902)	-44.4 (-48.7; -39.3)	10,508 (8,578 - 11,732)	-60.9 (-64.3; -57.7)	240,057 (215,685 - 266,066)	-29.6 (-33.2; -25.7)	139,296 (120,713 - 155,056)	-60.5 (-63.2; -57.7)
Central Europe, Eastern Europe, and Central Asia	755,310 (650,289 - 876,055)	-28.5 (-29.8; -27.1)	546,087 (490,738 - 589,336)	-35.0 (-39.9; -30.2)	5,913,428 (5,217,472 - 6,686,764)	-20.5 (-22.1; -18.7)	9,122,635 (8,354,959 - 9,868,186)	-35.2 (-39.8; -30.7)
Central Europe	230,353 (200,284 - 264,675)	-31.1 (-32.9; -29.0)	152,785 (133,281 - 170,806)	-39.6 (-46.2; -32.9)	1,928,993 (1,705,347 - 2,178,555)	-23.1 (-25.3; -21.3)	2,423,176 (2,123,325 - 2,705,068)	-41.3 (-47.7; -35.2)
Eastern Europe	450,791 (379,646 - 535,332)	-28.6 (-30.0; -27.0)	351,967 (310,837 - 386,333)	-35.6 (-41.1; -29.7)	3,342,611 (2,915,670 - 3,832,752)	-20.0 (-22.1; -17.9)	5,815,498 (5,227,855 - 6,413,662)	-34.8 (-40.2; -29.2)
Western Europe	415,104 (361,634 - 477,298)	-46.6 (-49.2; -43.7)	249,532 (210,818 - 272,171)	-64.9 (-67.2; -63.1)	4,512,426 (4,054,385 - 5,023,750)	-27.7 (-30.6; -25.2)	3,235,784 (2,856,947 - 3,510,423)	-63.6 (-65.5; -61.9)
Central Latin America	130,608 (113,342 - 151,298)	-33.7 (-35.7; -31.6)	43,524 (36,818 - 50,027)	-44.3 (-51.8; -35.8)	1,774,078 (1,579,058 - 1,967,317)	-22.8 (-25.1; -20.8)	772,210 (677,144 - 874,615)	-43.5 (-50.2; -35.9)
Tropical Latin America	183,912 (155,734 - 219,355)	-42.2 (-43.7; -40.4)	75,702 (66,328 - 81,466)	-57.0 (-60.4; -53.9)	2,090,491 (1,829,004 - 2,383,547)	-34.0 (-36.2; -31.7)	1,297,906 (1,186,652 - 1,387,325)	-57.4 (-60.6; -54.5)
Southern Latin America	42,268 (36,995 - 48,136)	-30.0 (-33.9; -26.0)	21,621 (18,981 - 23,568)	-49.4 (-55.6; -42.7)	447,785 (405,032 - 495,865)	-22.4 (-25.3; -19.2)	344,547 (312,866 - 375,538)	-49.4 (-54.8; -43.1)

Andean Latin America	28,799 (25,232 - 32,753)	-16.3 (-20.4; -12.3)	9,813 (8,027 - 11,862)	-39.5 (-52.5; -22.4)	378,101 (343,907 - 413,162)	-11.9 (-14.3; -9.4)	172,762 (146,497 - 204,786)	-39.9 (-51.3; -25.6)
Latin America and Caribbean	382,561 (331,285 - 448,234)	-35.0 (-36.6; -33.4)	148,996 (129,760 - 162,013)	-48.9 (-53.5; -44.0)	4,697,982 (4,169,824 - 5,255,695)	-26.2 (-28.1; -24.3)	2,582,822 (2,358,513 - 2,802,390)	-49.4 (-53.4; -45.0)
High-income North America	339,483 (284,891 - 407,863)	-33.1 (-35.4; -30.2)	120,956 (102,533 - 133,752)	-45.8 (-48.8; -41.6)	6,396,582 (5,601,347 - 7,278,525)	-5.0 (-9.1; -0.3)	2,258,091 (1,964,709 - 2,535,862)	-37.8 (-41.3; -34.6)
North Africa and Middle East	602,510 (531,317 - 682,539)	8.8 (6.3; 11.4)	210,108 (187,141 - 234,048)	-9.1 (-20.7; 4.0)	5,998,774 (5,473,980 - 6,566,718)	5.9 (3.6; 8.0)	4,751,376 (4,227,735 - 5,271,899)	-8.8 (-19.6; 2.2)
Sub-Saharan Africa	463,381 (399,749 - 536,598)	-2.1 (-3.1; -1.0)	135,143 (118,271 - 155,850)	-0.5 (-14.8; 13.3)	5,494,678 (4,870,378 - 6,108,412)	-2.5 (-3.4; -1.6)	3,145,910 (2,777,382 - 3,620,055)	-3.8 (-16.1; 7.4)
Central Sub-Saharan Africa	50,945 (43,721 - 59,014)	-8.2 (-12.2; -4.0)	15,635 (11,894 - 20,344)	-4.0 (-21.9; 17.4)	567,785 (508,463 - 625,600)	-8.4 (-11.0; -6.0)	364,782 (293,032 - 456,486)	-8.3 (-23.2; 10.9)
Southern Sub-Saharan Africa	61,925 (52,790 - 73,843)	6.8 (4.2; 9.5)	21,679 (19,635 - 23,344)	15.2 (3.7; 32.6)	685,560 (602,577 - 765,390)	-1.7 (-4.4; 1.1)	436,448 (395,726 - 474,281)	7.8 (-1.7; 20.2)
Eastern Sub-Saharan Africa	170,607 (145,784 - 198,519)	-3.7 (-5.5; -2.0)	47,406 (38,987 - 56,514)	9.0 (-10.3; 29.8)	2,089,578 (1,845,096 - 2,330,562)	-2.6 (-3.8; -1.3)	1,118,062 (944,649 - 1,335,310)	3.4 (-13.1; 20.8)
Western Sub-Saharan Africa	179,904 (155,868 - 208,529)	-2.5 (-4.2; -0.9)	50,423 (43,303 - 58,745)	-10.6 (-29.1; 8.6)	2,151,755 (1,904,235 - 2,394,733)	-0.8 (-2.1; 0.4)	1,226,619 (1,053,474 - 1,441,991)	-12.0 (-27.6; 3.5)
Countries in alphabetical order								
Afghanistan	22,218 (19,362 - 25,555)	18.9 (10.7; 27.7)	8,673 (6,211 - 11,616)	23.6 (-3.3; 55.2)	208,005 (188,770 - 227,010)	18.2 (12.4; 23.2)	224,570 (170,361 - 298,131)	27.3 (-0.6; 58.5)
Albania	2,894 (2,502 - 3,400)	-1.9 (-9.2; 5.5)	1,289 (951 - 1,690)	-16.7 (-37.8; 7.8)	26,258 (23,447 - 29,073)	-2.7 (-7.4; 2.4)	23,509 (18,260 - 29,478)	-17.1 (-34.8; 3.2)
Algeria	46,561 (40,719 - 53,253)	-1.4 (-7.3; 5.6)	18,650 (14,812 - 22,930)	-27.9 (-42.2; -10.5)	469,983 (427,467 - 513,763)	-2.6 (-6.7; 1.8)	372,749 (302,683 - 448,803)	-24.7 (-38.7; -8.3)
American Samoa	44 (38 - 51)	-1.5 (-8.5; 6.1)	13 (11 - 15)	-17.8 (-32.4; 0.2)	532 (487 - 580)	-2.1 (-6.7; 2.2)	317 (271 - 362)	-14.0 (-26.0; -0.5)

Andorra	63 (54 - 74)	-34.5 (-39.2; -29.6)	24 (18 - 31)	-45.8 (-60.5; -28.5)	665 (598 - 740)	-20.1 (-23.9; -16.0)	352 (285 - 429)	-42.7 (-55.4; -28.3)
Angola	12,670 (10,843 - 14,749)	-7.0 (-13.0; -0.3)	3,316 (2,718 - 4,133)	7.3 (-18.9; 44.3)	142,033 (127,318 - 156,902)	-4.7 (-8.6; -0.7)	83,386 (70,156 - 100,433)	-0.9 (-21.4; 24.1)
Antigua and Barbuda	64 (55 - 73)	-15.1 (-20.9; -9.4)	31 (27 - 36)	-23.2 (-35.2; -10.2)	817 (742 - 902)	-9.4 (-13.1; -5.4)	520 (454 - 589)	-26.8 (-37.5; -15.0)
Argentina	26,584 (23,193 - 30,633)	-29.7 (-34.7; -24.6)	12,807 (11,145 - 14,210)	-50.1 (-57.6; -40.9)	279,884 (252,522 - 308,570)	-21.0 (-25.0; -16.8)	209,973 (187,085 - 231,959)	-48.7 (-55.7; -40.4)
Armenia	2,972 (2,604 - 3,375)	-37.5 (-42.7; -32.0)	1,776 (1,491 - 2,072)	-44.6 (-54.8; -33.5)	26,000 (23,349 - 28,904)	-27.7 (-32.3; -22.7)	31,329 (27,048 - 36,176)	-45.3 (-54.2; -34.9)
Australia	17,984 (15,472 - 20,567)	-45.7 (-50.6; -40.0)	8,585 (6,997 - 9,647)	-62.1 (-65.6; -58.6)	204,410 (184,415 - 225,603)	-30.7 (-34.5; -26.9)	114,238 (98,586 - 127,019)	-61.3 (-64.2; -58.5)
Austria	8,695 (7,415 - 10,056)	-44.1 (-50.0; -37.5)	3,636 (3,029 - 4,109)	-76.1 (-78.3; -73.9)	100,261 (90,120 - 110,034)	-26.7 (-32.4; -20.6)	53,126 (46,624 - 58,824)	-71.9 (-74.4; -69.5)
Azerbaijan	8,027 (7,010 - 9,201)	18.0 (9.9; 27.8)	3,617 (2,837 - 4,280)	77.1 (39.4; 121.0)	72,259 (64,429 - 80,019)	4.4 (-1.1; 10.1)	76,935 (63,353 - 90,322)	39.5 (13.7; 69.3)
Bahamas	255 (223 - 295)	-14.8 (-20.9; -8.4)	97 (80 - 114)	-27.9 (-41.5; -13.8)	3,294 (3,011 - 3,625)	-12.2 (-15.9; -8.2)	1,800 (1,519 - 2,132)	-27.5 (-39.9; -13.5)
Bahrain	854 (724 - 1,019)	-30.1 (-35.1; -24.2)	136 (111 - 177)	-41.4 (-53.2; -27.1)	11,186 (10,069 - 12,387)	-23.8 (-27.5; -20.2)	4,155 (3,482 - 5,019)	-43.9 (-53.5; -33.0)
Bangladesh	72,392 (62,321 - 83,082)	9.5 (2.9; 16.2)	61,482 (45,248 - 79,277)	-1.2 (-27.3; 29.1)	768,325 (682,372 - 860,210)	7.5 (3.5; 12.1)	1,056,724 (793,191 - 1,381,291)	-6.5 (-30.5; 19.8)
Barbados	319 (279 - 364)	-14.9 (-20.6; -9.0)	202 (168 - 236)	-36.5 (-46.8; -25.1)	3,869 (3,501 - 4,285)	-10.5 (-14.4; -6.2)	3,046 (2,580 - 3,526)	-35.5 (-45.9; -23.5)
Belarus	20,424 (17,683 - 23,399)	-14.9 (-22.0; -7.8)	12,531 (9,778 - 15,740)	-17.2 (-35.0; 2.5)	148,210 (132,031 - 165,050)	-8.8 (-14.9; -1.0)	214,424 (168,402 - 267,693)	-20.6 (-36.9; -1.4)
Belgium	10,143 (8,765 - 11,639)	-45.9 (-50.4; -41.1)	5,833 (4,928 - 6,556)	-63.6 (-67.0; -60.2)	108,515 (98,167 - 120,257)	-28.6 (-33.0; -24.4)	76,881 (68,357 - 84,692)	-61.8 (-64.9; -58.8)
Belize	183 (158 - 211)	-5.0 (-10.9; 1.8)	59 (50 - 68)	-24.9 (-37.1; -10.2)	2,408 (2,174 - 2,650)	-0.3 (-5.1; 4.0)	1,111 (964 - 1,263)	-21.8 (-35.0; -7.6)
Benin	4,726 (4,074 - 5,442)	-7.6 (-13.5; -1.6)	1,430 (1,167 - 1,744)	-13.5 (-31.0; 9.1)	56,837 (50,983 - 62,918)	-6.7 (-10.4; -2.8)	33,549 (27,921 - 40,748)	-16.7 (-31.8; 2.8)

Bermuda	64 (56 - 74)	-29.1 (-33.8; -24.6)	30 (24 - 36)	-56.3 (-63.4; -47.5)	826 (744 - 911)	-19.8 (-23.7; -15.7)	444 (377 - 520)	-55.2 (-62.1; -46.5)
Bhutan	272 (235 - 316)	-9.3 (-14.9; -3.3)	151 (118 - 190)	-7.7 (-37.6; 30.9)	3,112 (2,785 - 3,441)	-5.4 (-8.9; -1.8)	2,829 (2,293 - 3,477)	-11.5 (-37.6; 20.0)
Bolivia (Plurinational State of)	4,915 (4,243 - 5,654)	-17.9 (-22.9; -13.0)	2,186 (1,583 - 2,914)	-22.3 (-42.0; 3.1)	63,437 (57,616 - 69,767)	-14.5 (-17.7; -10.7)	40,193 (30,343 - 52,411)	-27.0 (-43.8; -6.1)
Bosnia and Herzegovina	9,404 (8,117 - 10,918)	5.8 (-2.3; 13.3)	6,102 (4,996 - 7,465)	-3.6 (-20.4; 17.0)	75,016 (66,032 - 84,104)	6.6 (-0.3; 13.0)	103,408 (85,567 - 126,640)	-13.1 (-27.8; 4.7)
Botswana	1,647 (1,413 - 1,902)	-2.2 (-9.1; 5.3)	630 (466 - 830)	-4.8 (-30.4; 31.4)	18,033 (16,364 - 19,810)	-0.9 (-5.5; 3.5)	13,609 (10,399 - 17,563)	-7.1 (-30.4; 23.0)
Brazil	179,197 (151,358 - 214,373)	-42.7 (-44.3; -41.0)	73,921 (64,819 - 79,593)	-57.6 (-60.9; -54.4)	2,040,377 (1,784,220 - 2,330,526)	-34.5 (-36.7; -32.2)	1,268,106 (1,157,552 - 1,356,042)	-57.9 (-61.0; -55.0)
Brunei Darussalam	274 (235 - 323)	-44.4 (-48.6; -40.1)	58 (51 - 66)	-49.3 (-56.6; -37.7)	3,513 (3,122 - 3,991)	-34.4 (-38.1; -28.9)	1,577 (1,371 - 1,811)	-49.9 (-56.3; -40.3)
Bulgaria	24,139 (20,848 - 27,424)	0.0 (-7.9; 7.8)	20,225 (16,806 - 23,978)	-3.2 (-19.3; 14.7)	178,274 (152,797 - 206,400)	-0.6 (-8.1; 7.2)	318,647 (264,891 - 377,996)	-6.5 (-22.1; 11.7)
Burkina Faso	6,703 (5,677 - 7,890)	-0.9 (-6.7; 4.9)	1,640 (1,299 - 2,088)	3.4 (-17.2; 29.8)	80,812 (72,097 - 89,871)	-3.1 (-6.7; 0.4)	43,065 (34,537 - 54,067)	-0.3 (-17.9; 21.1)
Burundi	4,751 (4,001 - 5,570)	-22.8 (-28.0; -16.4)	1,187 (921 - 1,585)	-12.1 (-34.5; 18.5)	57,916 (51,599 - 64,902)	-20.3 (-23.5; -17.0)	29,603 (23,716 - 37,583)	-20.0 (-39.2; 4.7)
CÃ´te d'Ivoire	11,064 (9,556 - 12,708)	-8.8 (-14.9; -2.4)	2,462 (1,970 - 3,119)	-15.9 (-31.2; 2.7)	134,944 (121,030 - 149,028)	-9.1 (-12.7; -5.0)	67,594 (53,760 - 84,247)	-18.1 (-32.5; -2.8)
Cabo Verde	301 (263 - 343)	11.0 (4.4; 18.6)	135 (108 - 171)	77.0 (38.6; 136.0)	3,915 (3,537 - 4,283)	4.0 (0.0; 8.3)	2,408 (2,021 - 2,914)	42.0 (17.6; 78.3)
Cambodia	11,331 (9,693 - 13,071)	-0.7 (-8.4; 7.0)	5,121 (4,116 - 6,105)	4.7 (-23.4; 31.2)	105,046 (95,246 - 116,640)	-0.4 (-4.6; 4.6)	102,759 (84,532 - 121,156)	-2.5 (-24.0; 17.2)
Cameroon	10,848 (9,408 - 12,502)	10.9 (5.0; 17.4)	2,843 (2,129 - 3,727)	5.0 (-22.8; 44.1)	130,705 (117,753 - 144,212)	6.3 (2.3; 10.2)	72,354 (56,214 - 94,234)	4.1 (-21.0; 37.4)
Canada	29,153 (24,834 - 33,674)	-41.7 (-46.9; -35.6)	11,986 (9,786 - 13,616)	-55.6 (-60.2; -51.1)	524,129 (451,482 - 603,179)	-28.3 (-33.8; -22.6)	201,550 (172,579 - 229,415)	-50.7 (-54.4; -47.3)
Caribbean	39,242 (34,770 - 44,315)	-5.2 (-8.2; -2.4)	19,957 (17,097 - 23,012)	-19.7 (-29.1; -8.6)	455,313 (413,965 - 498,698)	-4.1 (-6.4; -1.8)	339,944 (295,597 - 390,545)	-18.0 (-27.5; -6.7)

Central African Republic	2,216 (1,904 - 2,586)	0.8 (-5.7; 7.8)	687 (455 - 1,006)	5.9 (-17.4; 37.1)	24,140 (21,733 - 26,566)	-2.1 (-6.5; 2.1)	17,900 (13,197 - 25,090)	1.2 (-19.9; 29.2)
Chad	5,722 (4,890 - 6,694)	-0.7 (-7.3; 6.1)	1,437 (1,080 - 1,927)	-2.1 (-20.4; 22.5)	65,158 (58,420 - 71,841)	1.1 (-2.9; 5.0)	37,794 (29,831 - 49,329)	-4.4 (-20.7; 15.9)
Chile	12,085 (10,515 - 13,782)	-30.1 (-35.9; -23.2)	6,434 (5,512 - 7,121)	-47.4 (-53.3; -41.7)	131,993 (118,977 - 147,784)	-25.2 (-29.9; -20.0)	100,663 (90,440 - 109,990)	-50.0 (-54.5; -45.4)
China	2,869,438 (2,375,792 - 3,458,736)	34.7 (29.4; 40.4)	1,029,318 (881,394 - 1,176,348)	-3.3 (-25.6; 16.4)	24,183,153 (20,804,403 - 27,869,456)	33.5 (25.6; 42.4)	21,393,857 (18,720,952 - 24,375,891)	-4.0 (-22.8; 12.6)
Colombia	25,360 (22,085 - 29,165)	-47.4 (-51.5; -42.6)	8,296 (6,267 - 10,478)	-62.8 (-70.9; -53.1)	335,213 (302,924 - 369,666)	-34.8 (-38.5; -31.1)	137,162 (110,328 - 166,980)	-62.7 (-69.9; -54.5)
Comoros	503 (435 - 578)	-19.7 (-25.5; -13.3)	181 (139 - 227)	-5.7 (-26.6; 27.1)	6,042 (5,520 - 6,615)	-19.1 (-22.4; -15.4)	3,759 (3,034 - 4,643)	-12.3 (-28.7; 17.0)
Congo	2,958 (2,551 - 3,408)	-9.6 (-16.2; -2.6)	951 (741 - 1,223)	-8.5 (-28.2; 18.0)	33,498 (30,360 - 36,615)	-12.0 (-15.5; -7.8)	22,096 (17,658 - 27,589)	-14.4 (-31.2; 6.5)
Cook Islands	22 (19 - 26)	-2.0 (-9.0; 5.6)	6 (5 - 7)	-29.2 (-44.7; -7.0)	266 (243 - 291)	2.0 (-1.9; 6.1)	141 (119 - 165)	-23.9 (-37.6; -7.1)
Costa Rica	2,578 (2,224 - 2,963)	-21.4 (-26.6; -15.4)	843 (632 - 1,056)	-42.3 (-54.9; -27.3)	33,858 (30,719 - 37,208)	-10.5 (-14.0; -6.6)	13,294 (10,699 - 16,049)	-40.0 (-50.6; -26.9)
Croatia	8,396 (7,323 - 9,647)	-33.4 (-38.6; -27.9)	5,583 (4,560 - 6,730)	-48.4 (-57.4; -38.2)	67,536 (59,561 - 75,556)	-25.5 (-31.2; -20.1)	82,120 (68,222 - 97,372)	-50.6 (-58.6; -41.9)
Cuba	12,923 (11,265 - 14,613)	-11.3 (-16.8; -5.7)	7,057 (5,737 - 8,467)	-16.1 (-30.6; 0.3)	143,103 (128,535 - 157,589)	-11.6 (-16.6; -6.5)	109,450 (91,869 - 130,019)	-18.5 (-32.4; -3.1)
Cyprus	694 (598 - 810)	-39.1 (-44.0; -34.0)	474 (403 - 601)	-61.7 (-68.3; -53.5)	8,792 (7,518 - 10,295)	-22.5 (-27.9; -16.2)	6,700 (5,786 - 8,057)	-61.6 (-67.9; -54.7)
Czechia	16,974 (14,560 - 19,540)	-54.9 (-58.9; -50.6)	8,951 (7,342 - 10,602)	-72.4 (-76.7; -67.5)	160,221 (144,645 - 176,995)	-40.4 (-45.0; -35.4)	139,674 (119,041 - 162,175)	-71.8 (-75.5; -67.6)
Democratic People's Republic of Korea	48,400 (41,341 - 56,595)	11.5 (2.7; 20.7)	19,093 (15,924 - 22,867)	-3.1 (-22.5; 23.0)	408,797 (368,366 - 452,265)	1.7 (-4.1; 7.5)	431,190 (363,600 - 506,659)	-1.9 (-19.3; 19.2)
Democratic Republic of the Congo	31,526 (26,819 - 36,720)	-9.9 (-15.8; -4.2)	10,163 (7,243 - 13,949)	-7.0 (-26.4; 17.4)	349,459 (311,106 - 387,020)	-10.7 (-14.4; -7.3)	230,130 (174,755 - 299,234)	-10.4 (-27.0; 12.1)
Denmark	5,420 (4,632 - 6,229)	-48.6 (-52.9; -44.4)	2,996 (2,546 - 3,352)	-53.1 (-58.3; -48.0)	57,558 (51,904 - 64,670)	-37.4 (-41.1; -33.8)	41,937 (37,367 - 45,984)	-54.9 (-58.7; -51.1)

Djibouti	689 (594 - 801)	-4.2 (-10.0; 1.9)	178 (136 - 236)	13.2 (-15.1; 52.4)	8,330 (7,508 - 9,178)	-5.7 (-9.2; -2.0)	4,564 (3,546 - 5,962)	6.3 (-17.6; 38.9)
Dominica	65 (57 - 74)	0.2 (-5.3; 6.6)	40 (33 - 47)	-8.9 (-24.4; 9.3)	728 (659 - 804)	-3.1 (-7.2; 0.6)	608 (516 - 707)	-10.3 (-25.2; 8.0)
Dominican Republic	8,263 (7,241 - 9,378)	23.4 (16.0; 31.0)	3,692 (2,939 - 4,699)	22.7 (-3.6; 57.7)	93,867 (85,763 - 101,801)	13.9 (9.5; 18.9)	67,521 (53,634 - 85,909)	24.0 (-3.5; 59.2)
Ecuador	9,022 (7,872 - 10,322)	-12.2 (-17.9; -6.8)	3,007 (2,443 - 3,757)	-24.8 (-40.5; -4.9)	118,475 (107,545 - 130,097)	-10.2 (-13.6; -6.4)	54,755 (44,812 - 66,655)	-30.8 (-44.9; -13.6)
Egypt	116,369 (100,531 - 133,786)	35.8 (25.7; 46.0)	30,078 (21,909 - 41,497)	15.3 (-17.3; 51.5)	1,089,819 (991,038 - 1,194,962)	31.7 (24.9; 38.1)	863,083 (669,024 - 1,115,487)	13.7 (-15.5; 43.8)
El Salvador	3,047 (2,657 - 3,477)	-11.7 (-17.9; -4.4)	1,086 (826 - 1,373)	-37.9 (-54.8; -15.9)	37,650 (34,256 - 41,472)	-3.9 (-7.3; -0.5)	17,579 (13,953 - 21,750)	-38.2 (-53.9; -18.7)
Equatorial Guinea	520 (445 - 604)	-9.4 (-16.9; -1.7)	145 (106 - 194)	-8.1 (-43.6; 37.9)	6,527 (5,838 - 7,218)	-2.0 (-6.3; 3.0)	3,312 (2,578 - 4,325)	-17.7 (-45.9; 14.0)
Eritrea	2,572 (2,195 - 3,004)	-7.5 (-14.7; -0.1)	686 (516 - 911)	28.9 (-12.3; 103.0)	32,321 (28,773 - 35,820)	-8.2 (-12.2; -4.3)	17,671 (14,085 - 22,919)	11.3 (-19.4; 54.2)
Estonia	1,614 (1,407 - 1,844)	-59.3 (-62.7; -54.7)	929 (716 - 1,237)	-78.5 (-83.0; -71.4)	14,327 (12,723 - 15,902)	-42.2 (-47.7; -36.4)	14,956 (12,161 - 18,943)	-76.7 (-80.9; -70.9)
Eswatini	667 (569 - 780)	-3.0 (-10.2; 4.0)	252 (191 - 330)	1.8 (-26.6; 37.7)	7,253 (6,579 - 7,977)	-0.4 (-5.1; 4.4)	5,422 (4,227 - 6,892)	-2.0 (-26.8; 30.7)
Ethiopia	31,237 (25,777 - 37,738)	-6.9 (-10.1; -3.4)	8,306 (6,679 - 10,449)	0.6 (-31.9; 42.1)	421,880 (358,584 - 491,150)	-3.8 (-6.7; -0.7)	187,550 (156,926 - 229,110)	-10.4 (-37.1; 20.4)
Fiji	737 (635 - 854)	-2.0 (-9.1; 5.7)	224 (182 - 274)	-9.1 (-29.6; 22.7)	8,661 (7,907 - 9,460)	0.3 (-3.8; 5.3)	6,110 (5,038 - 7,331)	-9.6 (-27.1; 16.1)
Finland	6,774 (5,879 - 7,682)	-38.1 (-43.2; -32.3)	4,019 (3,382 - 4,476)	-58.1 (-62.2; -54.1)	71,786 (64,948 - 80,232)	-28.1 (-33.8; -21.7)	53,683 (47,516 - 59,156)	-59.7 (-63.0; -56.3)
France	55,516 (48,349 - 63,404)	-32.2 (-38.3; -25.7)	29,127 (23,504 - 33,215)	-64.5 (-68.4; -61.0)	603,204 (541,740 - 661,999)	-17.9 (-24.2; -12.9)	376,573 (325,696 - 419,131)	-58.6 (-61.8; -55.3)
Gabon	1,054 (908 - 1,224)	-5.0 (-11.7; 2.1)	373 (301 - 450)	-3.6 (-25.3; 23.4)	12,128 (10,965 - 13,289)	-6.0 (-9.5; -1.4)	7,957 (6,545 - 9,469)	-7.7 (-26.6; 12.8)
Gambia	1,035 (901 - 1,187)	-0.4 (-6.5; 6.5)	316 (256 - 386)	7.2 (-14.1; 34.4)	12,606 (11,348 - 13,891)	-2.4 (-6.1; 1.5)	7,294 (5,935 - 8,813)	2.6 (-17.2; 27.3)

Georgia	5,225 (4,635 - 5,835)	-8.2 (-14.9; -0.9)	4,867 (4,035 - 5,701)	-24.4 (-38.2; -6.8)	40,094 (35,020 - 46,119)	-4.6 (-10.3; 2.0)	74,655 (63,818 - 86,560)	-19.6 (-33.9; -2.7)
Germany	99,537 (86,089 - 114,580)	-43.1 (-48.3; -38.3)	51,675 (44,395 - 57,171)	-65.7 (-69.0; -62.6)	1,092,376 (988,258 - 1,201,366)	-26.2 (-31.0; -21.7)	743,273 (658,532 - 817,760)	-62.9 (-65.8; -59.7)
Ghana	19,995 (17,363 - 22,819)	13.4 (5.7; 21.6)	7,242 (6,087 - 8,628)	4.3 (-16.5; 28.4)	231,794 (211,721 - 253,195)	8.3 (2.6; 13.6)	168,541 (141,291 - 201,136)	2.0 (-16.5; 24.2)
Greece	14,143 (12,250 - 16,142)	-44.4 (-49.1; -38.9)	13,284 (11,126 - 14,593)	-58.4 (-61.7; -55.2)	125,223 (107,435 - 145,035)	-30.1 (-35.1; -24.3)	151,349 (133,367 - 164,482)	-58.8 (-61.6; -55.8)
Greenland	51 (44 - 59)	-46.9 (-50.8; -42.4)	19 (15 - 22)	-55.7 (-63.5; -46.6)	958 (806 - 1,159)	-30.1 (-35.1; -24.8)	400 (339 - 466)	-53.6 (-60.1; -45.9)
Grenada	95 (83 - 108)	-16.0 (-22.1; -9.7)	50 (44 - 56)	-38.9 (-48.0; -27.9)	1,145 (1,025 - 1,275)	-9.8 (-14.2; -4.6)	904 (810 - 996)	-40.6 (-49.0; -29.3)
Guam	172 (149 - 197)	-4.1 (-11.8; 3.2)	39 (33 - 46)	-49.1 (-58.4; -36.6)	2,245 (2,062 - 2,453)	4.8 (0.4; 9.0)	1,096 (925 - 1,274)	-28.1 (-37.8; -16.4)
Guatemala	6,032 (5,164 - 6,936)	-27.6 (-32.7; -21.6)	1,955 (1,558 - 2,397)	-25.7 (-43.7; 0.0)	80,210 (71,884 - 88,655)	-12.8 (-17.5; -8.1)	34,557 (28,379 - 41,479)	-31.2 (-47.2; -6.7)
Guinea	4,958 (4,294 - 5,731)	9.5 (2.4; 16.1)	1,657 (1,314 - 2,079)	4.1 (-18.3; 30.0)	55,968 (50,580 - 61,255)	6.9 (2.5; 11.3)	37,761 (30,416 - 46,340)	1.7 (-17.5; 24.6)
Guinea-Bissau	833 (726 - 955)	-1.8 (-8.2; 5.0)	219 (170 - 276)	3.7 (-21.8; 39.8)	9,827 (8,850 - 10,808)	-1.6 (-5.4; 2.4)	6,036 (4,910 - 7,455)	-2.9 (-26.2; 27.1)
Guyana	663 (583 - 760)	-15.2 (-20.9; -9.3)	374 (299 - 463)	-33.9 (-48.6; -15.7)	7,610 (6,792 - 8,535)	-15.0 (-21.1; -8.8)	7,349 (5,868 - 9,045)	-38.5 (-52.5; -21.3)
Haiti	7,425 (6,486 - 8,458)	4.9 (-2.0; 11.9)	3,714 (2,525 - 5,676)	-11.0 (-30.4; 17.6)	81,741 (73,059 - 93,007)	-0.7 (-5.3; 4.3)	76,530 (54,375 - 115,643)	-14.6 (-33.4; 11.8)
Honduras	3,658 (3,165 - 4,260)	-11.4 (-17.5; -5.4)	2,568 (2,088 - 3,360)	45.9 (11.8; 95.2)	47,077 (42,501 - 51,913)	-6.5 (-10.2; -2.2)	46,571 (38,718 - 58,891)	29.5 (1.9; 70.0)
Hungary	21,030 (18,085 - 24,275)	-40.2 (-45.7; -34.7)	9,921 (8,208 - 11,671)	-57.1 (-63.7; -49.8)	185,861 (166,151 - 204,661)	-33.1 (-37.4; -27.5)	172,559 (146,569 - 200,189)	-56.7 (-62.4; -50.6)
Iceland	250 (213 - 289)	-44.7 (-49.4; -38.9)	101 (80 - 115)	-61.7 (-66.4; -56.7)	2,675 (2,434 - 2,950)	-27.9 (-31.1; -24.2)	1,403 (1,210 - 1,584)	-60.4 (-64.2; -56.3)
India	535,703 (453,187 - 631,773)	-4.2 (-6.0; -2.4)	271,240 (227,811 - 320,725)	-27.0 (-42.4; -11.4)	6,465,682 (5,540,992 - 7,378,042)	5.7 (4.2; 7.1)	5,689,264 (4,821,067 - 6,649,548)	-23.1 (-38.4; -8.3)

Indonesia	331,149 (274,629 - 396,011)	21.8 (18.6; 24.8)	136,176 (106,337 - 160,307)	42.5 (16.5; 69.1)	3,105,438 (2,658,401 - 3,616,207)	14.4 (12.1; 16.8)	2,957,904 (2,298,331 - 3,475,194)	31.4 (11.3; 50.3)
Iran (Islamic Republic of)	82,457 (69,393 - 97,578)	-14.2 (-16.0; -12.5)	33,151 (29,508 - 35,673)	-42.7 (-49.7; -32.3)	849,042 (736,693 - 971,863)	-13.1 (-16.1; -10.1)	668,904 (607,976 - 717,571)	-42.8 (-48.2; -34.9)
Iraq	42,447 (36,923 - 48,014)	-5.0 (-11.7; 2.3)	16,678 (13,716 - 19,426)	-1.9 (-21.6; 19.0)	414,374 (378,236 - 449,645)	-7.6 (-11.8; -2.6)	373,159 (305,766 - 438,919)	-7.0 (-25.1; 13.7)
Ireland	2,595 (2,246 - 2,974)	-50.4 (-55.2; -45.1)	1,624 (1,351 - 1,829)	-65.1 (-69.1; -61.4)	32,833 (28,612 - 38,140)	-32.3 (-37.3; -27.0)	21,903 (19,020 - 24,312)	-66.0 (-69.1; -63.1)
Israel	5,212 (4,456 - 5,989)	-43.1 (-47.8; -37.5)	1,951 (1,626 - 2,186)	-59.3 (-64.6; -54.1)	57,554 (51,571 - 63,379)	-26.9 (-30.4; -23.1)	29,557 (25,831 - 32,763)	-57.9 (-62.4; -53.8)
Italy	61,057 (50,826 - 73,202)	-52.9 (-56.2; -49.5)	45,390 (37,334 - 50,883)	-64.5 (-68.0; -62.5)	620,331 (535,543 - 719,536)	-28.9 (-32.4; -25.0)	527,796 (456,053 - 577,588)	-65.7 (-68.4; -63.7)
Jamaica	2,422 (2,136 - 2,736)	-0.1 (-6.5; 6.7)	1,603 (1,287 - 1,903)	-23.2 (-38.4; -4.5)	26,095 (23,510 - 28,730)	5.0 (-1.8; 10.6)	21,969 (18,250 - 26,015)	-23.2 (-37.7; -3.7)
Japan	196,244 (165,924 - 233,315)	-35.6 (-40.4; -31.2)	91,176 (69,971 - 103,724)	-71.9 (-75.0; -69.9)	2,678,191 (2,314,695 - 3,079,537)	-11.7 (-16.5; -6.9)	1,360,714 (1,131,347 - 1,540,307)	-61.1 (-64.2; -58.2)
Jordan	12,026 (10,423 - 13,812)	-24.9 (-30.9; -18.1)	2,472 (2,020 - 2,919)	-46.9 (-57.8; -35.2)	113,823 (102,391 - 124,390)	-23.9 (-28.6; -19.4)	59,926 (50,568 - 69,639)	-46.3 (-56.1; -36.4)
Kazakhstan	20,220 (17,608 - 23,022)	-26.9 (-32.4; -20.6)	13,555 (11,765 - 15,427)	-6.2 (-20.6; 9.6)	173,563 (154,399 - 194,809)	-19.5 (-25.2; -13.3)	260,682 (229,951 - 294,014)	-14.2 (-28.4; 0.0)
Kenya	21,824 (18,485 - 25,873)	-10.2 (-11.7; -8.8)	5,451 (4,258 - 6,686)	13.2 (-0.6; 29.3)	291,934 (250,613 - 335,670)	-5.4 (-6.7; -4.0)	132,482 (109,993 - 159,069)	7.5 (-3.2; 20.2)
Kiribati	79 (69 - 91)	-0.5 (-7.8; 7.5)	31 (24 - 37)	2.8 (-21.1; 28.5)	875 (799 - 960)	-3.6 (-7.4; 0.6)	906 (741 - 1,083)	-4.2 (-23.7; 17.1)
Kuwait	2,745 (2,374 - 3,199)	-4.0 (-10.7; 4.2)	617 (504 - 742)	-10.5 (-25.6; 6.9)	32,970 (29,762 - 36,320)	-6.5 (-11.0; -2.0)	15,225 (12,984 - 17,974)	-12.0 (-24.0; 2.4)
Kyrgyzstan	4,426 (3,915 - 5,018)	-32.3 (-37.6; -27.1)	2,522 (2,214 - 2,851)	-36.2 (-44.5; -27.1)	35,656 (31,666 - 39,544)	-28.8 (-33.1; -23.4)	53,950 (47,821 - 60,948)	-34.9 (-42.8; -25.9)
Lao People's Democratic Republic	5,048 (4,394 - 5,853)	-6.8 (-13.9; 0.9)	2,138 (1,703 - 2,690)	3.5 (-21.2; 33.4)	47,455 (42,950 - 52,291)	-6.8 (-11.2; -2.0)	46,822 (37,699 - 58,747)	-2.9 (-21.8; 20.4)
Latvia	5,553 (4,821 - 6,327)	-26.9 (-32.8; -20.6)	4,079 (3,436 - 4,818)	-40.1 (-48.2; -29.2)	34,569 (30,097 - 39,006)	-19.2 (-24.7; -14.2)	59,292 (51,026 - 69,570)	-41.2 (-48.7; -31.4)

Lebanon	6,161 (5,384 - 7,048)	7.9 (1.1; 15.5)	1,470 (986 - 1,903)	-25.0 (-48.0; -1.1)	65,698 (59,943 - 71,554)	8.4 (4.0; 13.1)	31,068 (24,587 - 38,151)	-15.1 (-34.7; 4.6)
Lesotho	1,254 (1,089 - 1,447)	10.7 (3.1; 19.4)	597 (441 - 790)	37.7 (5.7; 79.9)	13,391 (12,119 - 14,764)	15.7 (10.6; 21.0)	12,135 (9,095 - 15,853)	32.8 (4.2; 70.9)
Liberia	1,827 (1,587 - 2,100)	-10.2 (-16.2; -4.3)	481 (378 - 626)	-16.5 (-34.2; 4.8)	22,631 (20,337 - 25,016)	-9.2 (-13.2; -5.5)	11,746 (9,289 - 14,924)	-20.3 (-35.1; -1.4)
Libya	7,177 (6,299 - 8,209)	25.3 (16.7; 34.8)	2,301 (1,732 - 3,073)	5.7 (-18.7; 38.2)	76,359 (69,400 - 82,982)	23.5 (17.7; 29.4)	55,357 (43,126 - 71,016)	11.2 (-12.0; 42.4)
Lithuania	7,491 (6,531 - 8,559)	-18.9 (-25.2; -11.7)	4,054 (3,364 - 4,770)	-18.9 (-30.5; -4.8)	47,473 (40,153 - 55,624)	-14.9 (-22.1; -8.5)	62,345 (53,383 - 73,070)	-26.8 (-36.8; -14.7)
Luxembourg	383 (331 - 439)	-59.1 (-63.5; -54.3)	224 (182 - 256)	-76.7 (-79.9; -73.6)	4,511 (3,985 - 5,163)	-44.9 (-49.3; -40.2)	3,010 (2,589 - 3,401)	-75.6 (-78.4; -72.7)
Madagascar	15,900 (13,811 - 18,262)	6.3 (-0.9; 13.7)	3,918 (2,936 - 5,143)	23.1 (-5.5; 63.2)	179,446 (161,690 - 196,811)	0.2 (-4.0; 4.6)	103,036 (81,075 - 132,268)	15.1 (-9.4; 46.1)
Malawi	7,998 (6,820 - 9,250)	-1.6 (-8.4; 4.8)	2,468 (1,934 - 3,107)	-0.5 (-20.2; 26.5)	98,559 (88,205 - 109,121)	-2.5 (-6.6; 1.5)	56,397 (46,263 - 68,582)	-2.9 (-21.2; 20.5)
Malaysia	27,115 (23,378 - 31,592)	-20.4 (-26.1; -13.6)	8,202 (6,443 - 10,435)	-24.6 (-41.4; -2.6)	296,653 (270,066 - 325,983)	-11.1 (-15.3; -7.0)	198,477 (162,122 - 241,254)	-26.4 (-39.5; -10.0)
Maldives	223 (192 - 260)	-35.0 (-39.8; -29.5)	68 (55 - 81)	-41.1 (-56.6; -23.1)	2,612 (2,360 - 2,869)	-26.9 (-30.6; -23.4)	1,501 (1,268 - 1,753)	-43.1 (-54.2; -30.9)
Mali	7,436 (6,396 - 8,645)	-11.7 (-17.6; -6.1)	2,112 (1,678 - 2,617)	-11.3 (-28.5; 9.4)	86,140 (77,303 - 94,944)	-10.0 (-13.4; -6.5)	51,828 (42,680 - 63,108)	-15.9 (-30.7; 1.7)
Malta	395 (340 - 457)	-42.4 (-47.2; -36.8)	229 (189 - 263)	-60.7 (-66.0; -55.3)	4,014 (3,493 - 4,715)	-31.6 (-36.9; -25.8)	3,173 (2,740 - 3,583)	-61.9 (-66.3; -57.4)
Marshall Islands	38 (33 - 44)	1.3 (-4.9; 8.3)	10 (7 - 13)	-4.0 (-24.4; 20.4)	420 (384 - 458)	0.9 (-3.2; 5.5)	305 (241 - 393)	-3.7 (-22.1; 17.7)
Mauritania	1,942 (1,687 - 2,229)	-22.3 (-28.1; -17.4)	536 (434 - 651)	-32.8 (-46.7; -16.9)	24,178 (21,821 - 26,454)	-19.6 (-22.9; -16.0)	12,303 (9,990 - 15,056)	-36.6 (-48.2; -23.0)
Mauritius	1,389 (1,200 - 1,607)	-49.6 (-53.9; -44.5)	562 (459 - 666)	-59.2 (-65.9; -51.6)	15,084 (13,749 - 16,588)	-37.9 (-41.2; -34.3)	11,190 (9,456 - 13,024)	-59.8 (-65.5; -53.7)
Mexico	67,906 (57,417 - 80,365)	-32.2 (-33.9; -30.5)	20,166 (17,007 - 23,052)	-46.2 (-53.0; -38.8)	966,611 (842,958 - 1,093,635)	-21.4 (-24.2; -19.0)	380,772 (333,788 - 433,774)	-43.3 (-49.4; -36.6)

Micronesia (Federated States of)	65 (56 - 75)	-7.7 (-13.4; -1.4)	23 (17 - 29)	-0.5 (-29.6; 37.7)	739 (675 - 810)	-7.9 (-11.7; -4.0)	627 (468 - 803)	-4.8 (-30.9; 25.3)
Monaco	56 (47 - 65)	-16.6 (-22.5; -10.3)	34 (26 - 41)	-56.0 (-65.3; -40.2)	544 (491 - 615)	-9.6 (-13.5; -5.6)	434 (358 - 518)	-51.4 (-60.8; -36.1)
Mongolia	1,476 (1,275 - 1,698)	-5.4 (-11.6; 1.7)	433 (338 - 566)	58.9 (14.3; 110.0)	14,964 (13,273 - 16,663)	-1.0 (-6.1; 3.7)	12,772 (10,293 - 16,014)	27.9 (-2.5; 61.3)
Montenegro	851 (733 - 989)	17.8 (9.0; 26.9)	401 (331 - 468)	37.6 (12.5; 66.4)	7,266 (6,505 - 8,083)	3.3 (-1.4; 8.2)	6,949 (5,918 - 8,017)	26.1 (6.1; 47.6)
Morocco	48,382 (42,016 - 55,500)	14.9 (5.8; 23.9)	21,220 (16,956 - 25,800)	19.0 (-6.7; 43.8)	476,785 (435,246 - 521,362)	13.5 (8.4; 19.4)	451,674 (364,060 - 544,462)	18.5 (-6.7; 42.2)
Mozambique	17,359 (14,992 - 20,070)	22.3 (14.3; 31.1)	5,486 (4,262 - 7,048)	29.1 (-5.5; 67.6)	187,636 (168,612 - 206,149)	17.5 (12.4; 22.5)	131,494 (105,148 - 165,179)	29.9 (0.0; 65.8)
Myanmar	37,665 (32,314 - 43,723)	-4.9 (-11.2; 2.6)	32,091 (26,898 - 37,478)	-2.2 (-20.5; 22.6)	368,740 (331,904 - 406,125)	0.6 (-3.9; 5.5)	597,175 (498,287 - 700,052)	-9.8 (-26.7; 12.9)
Namibia	1,507 (1,300 - 1,748)	-18.9 (-24.9; -12.3)	741 (608 - 896)	-7.9 (-28.6; 22.0)	16,411 (14,808 - 18,094)	-14.8 (-18.5; -10.5)	13,646 (11,317 - 16,384)	-13.0 (-31.5; 12.7)
Nauru	6 (5 - 6)	3.3 (-4.0; 10.7)	2 (1 - 2)	-2.1 (-19.6; 19.2)	65 (59 - 70)	-0.3 (-4.4; 3.9)	60 (46 - 77)	-0.9 (-16.7; 18.0)
Nepal	8,435 (7,227 - 9,884)	-8.4 (-13.6; -2.7)	5,958 (4,273 - 8,298)	-7.0 (-33.7; 28.0)	95,029 (84,928 - 105,719)	-6.2 (-9.2; -2.8)	114,408 (83,375 - 159,404)	-12.1 (-35.4; 16.9)
Netherlands	16,590 (14,218 - 19,239)	-38.6 (-44.1; -33.1)	8,484 (7,159 - 9,549)	-51.9 (-56.4; -47.3)	175,363 (158,614 - 193,614)	-23.0 (-26.9; -19.1)	118,813 (104,893 - 131,675)	-50.6 (-54.3; -46.9)
New Zealand	2,960 (2,541 - 3,426)	-36.9 (-42.4; -30.3)	1,923 (1,580 - 2,168)	-54.6 (-59.5; -49.9)	35,647 (30,951 - 41,196)	-23.2 (-29.4; -16.8)	25,058 (21,652 - 27,871)	-55.9 (-59.8; -51.8)
Nicaragua	2,598 (2,231 - 2,978)	-26.6 (-32.3; -21.7)	1,002 (851 - 1,148)	-9.7 (-26.1; 10.5)	34,864 (31,312 - 38,485)	-17.5 (-21.0; -13.3)	17,921 (15,272 - 20,637)	-19.3 (-32.7; -0.8)
Niger	7,463 (6,315 - 8,718)	-9.9 (-15.1; -4.1)	1,774 (1,270 - 2,397)	-10.3 (-29.8; 13.2)	82,996 (73,904 - 91,572)	-9.8 (-13.6; -6.4)	48,163 (36,710 - 63,566)	-15.8 (-31.0; 3.0)
Nigeria	80,350 (67,703 - 95,410)	-4.7 (-6.5; -3.1)	22,055 (17,507 - 26,971)	-18.1 (-43.1; 13.2)	975,046 (841,767 - 1,115,741)	0.0 (-1.7; 1.9)	525,144 (428,259 - 646,332)	-17.8 (-39.5; 9.5)
Niue	2 (2 - 2)	-2.2 (-9.3; 5.6)	1 (1 - 1)	-18.2 (-35.6; 1.1)	23 (21 - 26)	-1.4 (-5.5; 2.6)	19 (15 - 23)	-14.8 (-30.0; 1.8)

North Macedonia	5,425 (4,628 - 6,312)	-8.9 (-15.8; -1.5)	4,945 (4,139 - 5,858)	2.0 (-14.1; 21.5)	40,581 (34,754 - 47,940)	-8.8 (-15.3; -2.0)	80,514 (67,161 - 95,299)	-7.7 (-22.0; 9.9)
Northern Ireland	1,474 (1,266 - 1,690)	-56.1 (-60.1; -51.9)	986 (839 - 1,099)	-60.7 (-65.2; -56.6)	15,384 (13,649 - 17,562)	-41.2 (-44.9; -37.1)	12,700 (11,223 - 13,914)	-63.1 (-66.5; -59.9)
Northern Mariana Islands	42 (36 - 49)	-15.1 (-20.9; -9.4)	10 (8 - 11)	-37.6 (-49.4; -23.1)	503 (457 - 556)	-12.1 (-15.9; -8.6)	284 (242 - 330)	-32.5 (-42.9; -21.1)
Norway	5,416 (4,508 - 6,520)	-39.4 (-42.9; -35.4)	2,268 (1,900 - 2,523)	-65.9 (-68.1; -63.3)	75,077 (65,284 - 86,571)	-7.2 (-11.1; -3.4)	33,991 (29,547 - 37,704)	-60.7 (-63.5; -58.0)
Oman	2,666 (2,292 - 3,116)	3.3 (-4.6; 11.8)	650 (557 - 758)	-7.8 (-30.7; 24.4)	29,829 (26,974 - 32,984)	-2.9 (-7.6; 1.9)	17,267 (14,836 - 19,890)	-18.3 (-35.2; 6.7)
Pakistan	91,344 (77,252 - 108,044)	6.0 (2.3; 9.2)	38,988 (32,247 - 51,899)	6.8 (-15.4; 43.5)	979,624 (843,708 - 1,121,097)	6.1 (2.5; 9.5)	882,645 (743,169 - 1,132,980)	8.3 (-13.2; 42.2)
Palau	24 (20 - 28)	6.2 (-1.7; 14.7)	8 (6 - 9)	-5.1 (-30.7; 30.7)	267 (243 - 293)	5.4 (1.3; 9.9)	217 (177 - 264)	-3.7 (-27.2; 25.7)
Palestine	3,632 (3,175 - 4,171)	2.5 (-5.9; 11.2)	1,511 (1,288 - 1,720)	-15.9 (-33.0; 6.2)	35,217 (31,813 - 38,979)	-0.6 (-5.9; 4.6)	31,202 (27,203 - 35,384)	-17.8 (-34.6; 3.0)
Panama	2,545 (2,212 - 2,895)	-24.9 (-30.6; -18.8)	992 (742 - 1,235)	-38.7 (-52.7; -22.8)	30,151 (27,368 - 33,115)	-12.0 (-15.6; -8.1)	14,547 (11,652 - 17,894)	-40.3 (-52.8; -25.6)
Papua New Guinea	3,931 (3,364 - 4,547)	0.6 (-6.5; 7.0)	834 (527 - 1,302)	12.5 (-12.7; 44.9)	45,959 (41,690 - 50,359)	3.0 (-0.7; 6.8)	27,779 (20,223 - 39,253)	8.6 (-9.3; 30.8)
Paraguay	4,715 (4,109 - 5,412)	-10.5 (-16.7; -4.4)	1,781 (1,348 - 2,315)	-28.6 (-46.1; -4.3)	50,114 (45,427 - 55,059)	-6.0 (-9.9; -1.7)	29,799 (23,594 - 38,242)	-26.3 (-43.4; -0.7)
Peru	14,862 (12,895 - 16,992)	-18.4 (-24.3; -12.7)	4,619 (3,392 - 6,125)	-50.6 (-64.8; -29.2)	196,189 (178,048 - 214,728)	-12.5 (-15.9; -9.3)	77,814 (60,799 - 99,898)	-48.8 (-61.6; -30.9)
Philippines	76,066 (63,873 - 90,902)	2.7 (0.8; 4.6)	24,336 (20,469 - 28,435)	13.6 (-7.6; 32.9)	780,696 (678,255 - 890,431)	2.1 (-0.6; 4.7)	587,348 (491,512 - 680,488)	29.0 (4.8; 49.8)
Poland	56,055 (46,715 - 67,731)	-30.4 (-32.4; -28.1)	32,970 (27,310 - 38,064)	-43.1 (-50.7; -35.2)	517,474 (449,492 - 597,632)	-21.7 (-24.4; -18.9)	530,258 (457,391 - 606,224)	-44.6 (-51.1; -38.0)
Portugal	12,554 (11,035 - 14,245)	-65.7 (-68.7; -62.1)	11,621 (9,881 - 12,831)	-75.0 (-77.3; -72.7)	133,416 (115,888 - 155,569)	-49.7 (-54.4; -44.8)	141,331 (125,693 - 154,008)	-75.3 (-77.1; -73.4)
Puerto Rico	2,756 (2,366 - 3,205)	-21.6 (-27.9; -14.6)	1,123 (841 - 1,390)	-54.8 (-64.0; -44.3)	44,779 (40,687 - 49,226)	-4.1 (-8.8; 0.5)	16,443 (13,387 - 19,800)	-46.2 (-54.8; -36.9)

Qatar	1,165 (949 - 1,435)	-24.8 (-30.6; -18.4)	70 (53 - 97)	-28.6 (-47.9; -4.3)	15,886 (14,141 - 17,914)	-20.9 (-24.8; -17.5)	3,791 (2,990 - 4,599)	-35.5 (-49.9; -19.3)
Republic of Korea	57,138 (48,608 - 65,636)	-63.4 (-66.5; -59.9)	22,579 (18,898 - 27,429)	-73.4 (-78.0; -62.0)	759,142 (674,522 - 872,825)	-50.2 (-53.8; -45.5)	413,615 (360,534 - 480,454)	-73.1 (-76.6; -62.9)
Republic of Moldova	6,182 (5,356 - 7,067)	-0.3 (-8.9; 7.7)	3,606 (3,127 - 4,078)	-27.1 (-36.9; -16.3)	43,316 (38,339 - 48,471)	-1.3 (-7.7; 5.4)	65,498 (58,038 - 74,260)	-17.5 (-28.2; -5.4)
Romania	48,906 (42,074 - 55,705)	-32.5 (-38.0; -26.8)	36,479 (30,761 - 42,489)	-34.1 (-44.6; -22.2)	374,646 (319,240 - 429,109)	-23.3 (-30.2; -17.8)	551,541 (473,101 - 638,896)	-35.3 (-45.0; -24.0)
Russian Federation	321,938 (268,901 - 383,844)	-30.3 (-31.6; -29.0)	254,664 (220,032 - 285,169)	-37.6 (-44.2; -30.7)	2,354,055 (2,044,582 - 2,725,485)	-22.9 (-24.9; -20.9)	4,143,601 (3,685,649 - 4,651,105)	-37.5 (-43.8; -30.7)
Rwanda	4,979 (4,245 - 5,821)	-30.2 (-35.7; -24.8)	1,449 (1,045 - 1,914)	-19.7 (-42.6; 6.0)	61,941 (55,511 - 68,655)	-25.3 (-28.9; -21.4)	32,580 (25,496 - 41,696)	-28.5 (-46.5; -9.2)
Saint Kitts and Nevis	58 (51 - 66)	-26.9 (-31.9; -21.4)	34 (30 - 39)	-47.2 (-54.4; -38.9)	727 (647 - 814)	-23.3 (-28.4; -17.9)	607 (527 - 698)	-48.3 (-55.3; -39.2)
Saint Lucia	173 (151 - 196)	-28.2 (-33.7; -23.0)	98 (83 - 113)	-47.3 (-55.4; -36.5)	2,065 (1,841 - 2,324)	-23.5 (-28.9; -18.4)	1,524 (1,305 - 1,751)	-47.5 (-55.6; -36.6)
Saint Vincent and the Grenadines	109 (95 - 125)	4.3 (-2.0; 11.2)	58 (51 - 66)	-40.7 (-47.8; -32.5)	1,275 (1,153 - 1,419)	4.9 (0.5; 10.0)	971 (852 - 1,095)	-43.2 (-50.2; -35.1)
Samoa	145 (127 - 166)	-4.5 (-12.0; 2.2)	52 (41 - 63)	-11.2 (-29.3; 14.8)	1,690 (1,549 - 1,846)	-2.8 (-6.7; 1.4)	1,208 (994 - 1,443)	-10.5 (-26.2; 10.0)
San Marino	33 (28 - 37)	-27.5 (-32.4; -22.2)	21 (15 - 28)	-38.3 (-57.1; -15.6)	321 (289 - 357)	-16.7 (-20.1; -13.0)	268 (200 - 346)	-36.6 (-53.6; -15.5)
Sao Tome and Principe	143 (126 - 162)	11.0 (3.7; 18.0)	38 (31 - 50)	11.1 (-13.1; 42.0)	1,687 (1,540 - 1,841)	6.5 (2.4; 11.2)	915 (753 - 1,140)	8.8 (-10.7; 34.1)
Saudi Arabia	28,087 (24,748 - 32,005)	20.7 (12.7; 29.8)	7,034 (5,262 - 8,441)	-22.8 (-41.9; 2.1)	321,122 (290,893 - 356,283)	13.5 (1.9; 23.8)	207,936 (160,132 - 249,405)	-17.4 (-36.3; 5.8)
Scotland	6,444 (5,554 - 7,459)	-50.1 (-54.7; -44.4)	4,092 (3,512 - 4,508)	-59.2 (-63.1; -55.3)	105,416 (88,102 - 120,724)	-11.2 (-19.8; -2.5)	59,378 (52,907 - 65,696)	-56.9 (-60.5; -53.0)
Senegal	6,758 (5,846 - 7,809)	-13.8 (-19.1; -8.6)	1,997 (1,641 - 2,439)	-11.3 (-28.4; 9.5)	83,571 (75,475 - 91,815)	-12.4 (-16.0; -8.1)	46,474 (38,404 - 55,709)	-16.7 (-31.3; 0.0)
Serbia	24,038 (20,603 - 28,104)	-18.9 (-24.8; -12.4)	20,110 (16,960 - 23,691)	-19.4 (-35.3; -4.6)	187,464 (160,195 - 218,248)	-14.3 (-20.9; -7.8)	313,709 (265,681 - 370,741)	-27.7 (-42.5; -14.0)

Seychelles	114 (98 - 133)	-12.1 (-18.1; -5.1)	35 (29 - 41)	-28.2 (-38.3; -15.6)	1,166 (1,063 - 1,275)	-8.4 (-12.2; -4.6)	776 (657 - 913)	-27.6 (-35.9; -17.5)
Sierra Leone	4,057 (3,563 - 4,626)	-2.5 (-8.3; 3.4)	1,119 (855 - 1,463)	-7.9 (-26.6; 14.6)	47,950 (43,369 - 52,569)	-4.7 (-8.5; -1.2)	28,705 (22,332 - 37,072)	-10.7 (-28.0; 10.8)
Singapore	4,160 (3,514 - 4,905)	-64.1 (-68.1; -59.8)	890 (730 - 1,003)	-77.3 (-80.2; -74.8)	56,421 (50,888 - 62,449)	-51.2 (-54.4; -47.9)	20,596 (17,456 - 23,673)	-74.1 (-76.5; -71.8)
Slovakia	9,617 (8,322 - 11,153)	-25.9 (-32.0; -20.2)	4,318 (3,484 - 5,243)	-38.2 (-49.6; -24.8)	85,355 (77,352 - 93,285)	-20.5 (-24.4; -16.2)	79,048 (65,499 - 94,040)	-39.1 (-48.8; -27.9)
Slovenia	2,624 (2,287 - 3,032)	-51.8 (-56.1; -47.2)	1,492 (1,136 - 1,939)	-69.1 (-77.3; -59.4)	23,040 (20,770 - 25,578)	-41.8 (-45.9; -37.5)	21,240 (17,202 - 26,157)	-69.6 (-76.9; -61.4)
Solomon Islands	298 (259 - 343)	-0.8 (-7.8; 6.2)	136 (106 - 176)	23.9 (-6.0; 56.9)	3,241 (2,936 - 3,539)	-1.0 (-4.8; 3.5)	3,722 (2,974 - 4,697)	13.9 (-11.9; 41.7)
Somalia	7,928 (6,665 - 9,419)	-8.7 (-14.7; -2.0)	1,848 (1,269 - 2,705)	9.2 (-18.8; 47.1)	89,374 (79,967 - 100,075)	-8.5 (-11.8; -5.0)	50,035 (36,888 - 68,046)	0.8 (-22.4; 33.7)
South Africa	48,614 (40,779 - 59,036)	4.7 (1.8; 7.6)	16,702 (15,076 - 17,906)	18.2 (4.2; 38.0)	542,188 (472,100 - 615,187)	-5.0 (-8.1; -1.8)	329,002 (297,574 - 356,567)	7.6 (-3.1; 20.9)
South Sudan	4,404 (3,792 - 5,089)	-4.9 (-10.8; 1.1)	934 (596 - 1,378)	-5.2 (-29.5; 27.3)	53,292 (48,042 - 58,731)	-3.9 (-7.4; -0.3)	23,216 (16,878 - 32,904)	-8.6 (-29.0; 18.9)
Spain	42,020 (35,910 - 49,177)	-53.6 (-58.4; -48.8)	23,256 (19,130 - 26,828)	-73.0 (-75.5; -70.2)	437,307 (394,145 - 483,105)	-36.6 (-41.1; -32.3)	290,083 (252,227 - 321,866)	-71.0 (-73.3; -68.8)
Sri Lanka	21,892 (18,788 - 25,261)	-17.2 (-23.7; -10.0)	8,992 (6,796 - 11,598)	-37.0 (-53.6; -17.6)	224,553 (202,352 - 250,046)	-8.7 (-12.6; -4.3)	177,960 (140,903 - 223,337)	-33.0 (-48.4; -14.9)
Suriname	535 (470 - 609)	-0.2 (-6.5; 7.4)	249 (208 - 293)	-12.4 (-26.6; 4.3)	6,117 (5,564 - 6,710)	0.7 (-3.6; 4.7)	4,475 (3,758 - 5,202)	-12.1 (-25.5; 3.8)
Sweden	11,265 (9,325 - 13,660)	-36.0 (-39.8; -31.7)	5,948 (5,062 - 6,762)	-49.8 (-54.4; -44.3)	149,392 (130,640 - 172,173)	-6.1 (-12.2; 0.5)	81,892 (72,485 - 90,906)	-46.8 (-50.9; -42.4)
Switzerland	6,556 (5,573 - 7,517)	-49.6 (-54.3; -44.5)	3,449 (2,776 - 4,041)	-65.8 (-69.8; -61.3)	71,273 (64,276 - 79,239)	-33.4 (-37.4; -29.2)	44,320 (38,034 - 50,140)	-63.6 (-66.9; -60.2)
Syrian Arab Republic	14,837 (12,917 - 16,977)	-9.9 (-16.4; -3.4)	4,954 (3,849 - 6,348)	-20.6 (-42.8; 6.2)	156,735 (142,891 - 171,185)	-8.4 (-12.3; -4.5)	116,537 (92,554 - 145,609)	-25.1 (-44.7; -2.2)
Taiwan (Province of China)	28,771 (24,528 - 33,687)	-37.1 (-43.4; -30.6)	6,788 (5,346 - 8,370)	-68.0 (-74.4; -60.6)	332,070 (301,585 - 363,133)	-19.7 (-24.6; -14.7)	155,429 (128,707 - 183,907)	-59.9 (-65.3; -53.8)

Tajikistan	2,713 (2,265 - 3,198)	14.6 (7.5; 22.0)	1,051 (858 - 1,308)	96.0 (48.6; 164.0)	26,939 (23,769 - 30,343)	8.0 (1.1; 15.4)	24,033 (19,642 - 30,097)	68.8 (32.9; 116.0)
Thailand	67,702 (58,646 - 78,864)	-26.5 (-32.0; -20.4)	20,242 (15,222 - 26,415)	-35.3 (-52.7; -13.6)	743,350 (676,391 - 818,179)	-15.7 (-19.5; -11.6)	464,145 (368,546 - 572,098)	-31.3 (-46.4; -13.5)
Timor-Leste	1,078 (931 - 1,256)	14.1 (6.1; 22.4)	425 (310 - 612)	41.9 (8.4; 84.9)	10,017 (9,052 - 11,070)	12.1 (7.4; 17.3)	9,293 (6,994 - 12,804)	35.3 (7.0; 68.3)
Togo	3,743 (3,266 - 4,276)	-6.4 (-12.5; 0.4)	926 (744 - 1,152)	-9.6 (-26.3; 12.7)	44,961 (40,530 - 49,532)	-7.5 (-11.1; -3.8)	24,927 (20,381 - 30,261)	-12.1 (-27.4; 6.9)
Tokelau	1 (1 - 1)	-0.1 (-8.1; 7.0)	0 (0 - 1)	-8.6 (-29.7; 20.8)	13 (12 - 15)	1.9 (-2.3; 6.2)	10 (9 - 12)	-7.7 (-26.4; 16.4)
Tonga	64 (55 - 73)	-4.1 (-11.4; 2.7)	23 (18 - 27)	2.8 (-20.2; 35.5)	761 (696 - 833)	-3.2 (-7.5; 1.1)	489 (410 - 574)	-0.4 (-17.3; 21.4)
Trinidad and Tobago	1,432 (1,258 - 1,627)	-25.0 (-29.8; -20.2)	718 (553 - 908)	-47.6 (-59.4; -33.6)	18,180 (16,452 - 20,002)	-17.2 (-21.0; -13.3)	12,296 (9,761 - 15,440)	-46.6 (-58.5; -32.6)
Tunisia	15,194 (13,233 - 17,660)	15.9 (7.6; 23.8)	6,748 (5,045 - 8,677)	-2.2 (-29.8; 27.8)	141,938 (129,035 - 155,655)	25.6 (20.1; 31.5)	128,086 (98,885 - 163,158)	4.1 (-22.0; 34.2)
Turkey	81,599 (71,499 - 93,648)	4.7 (-3.0; 12.8)	30,216 (24,111 - 36,742)	-3.6 (-32.5; 26.5)	838,412 (764,091 - 920,762)	-5.2 (-9.5; -0.7)	551,064 (459,967 - 649,248)	-8.0 (-32.7; 14.3)
Turkmenistan	5,123 (4,488 - 5,926)	8.9 (1.4; 17.0)	2,572 (2,050 - 3,171)	16.6 (-7.5; 46.4)	41,398 (37,640 - 45,624)	12.8 (7.4; 18.7)	60,520 (48,085 - 74,942)	20.5 (-4.8; 49.7)
Tuvalu	9 (8 - 10)	4.8 (-1.9; 12.9)	4 (3 - 5)	-1.0 (-27.2; 37.9)	103 (94 - 113)	2.5 (-2.0; 6.9)	93 (75 - 114)	-5.6 (-26.9; 24.3)
Uganda	15,869 (13,425 - 18,439)	-12.4 (-18.1; -5.8)	3,742 (2,803 - 4,652)	4.0 (-19.6; 29.6)	189,986 (169,792 - 211,658)	-7.5 (-11.1; -3.6)	89,402 (71,121 - 107,725)	-1.5 (-20.2; 19.4)
Ukraine	87,590 (73,263 - 105,375)	-28.4 (-31.9; -24.8)	72,105 (62,041 - 82,492)	-33.8 (-42.6; -23.4)	700,661 (606,684 - 800,958)	-14.7 (-22.0; -7.1)	1,255,384 (1,097,926 - 1,431,911)	-28.7 (-37.7; -17.6)
United Arab Emirates	10,857 (9,258 - 12,752)	-5.0 (-11.4; 2.3)	1,240 (891 - 1,675)	-41.1 (-55.1; -23.6)	108,862 (98,572 - 119,869)	-3.3 (-7.9; 1.1)	54,428 (41,443 - 69,792)	-33.9 (-48.7; -15.7)
United Kingdom	49,376 (41,557 - 58,684)	-50.8 (-53.3; -48.1)	33,648 (28,979 - 36,138)	-62.7 (-65.1; -60.5)	575,501 (502,104 - 656,348)	-29.0 (-32.2; -25.8)	431,116 (386,414 - 464,261)	-63.4 (-65.2; -61.7)
United Republic of Tanzania	27,570 (23,636 - 31,977)	14.7 (6.7; 23.0)	8,864 (6,597 - 11,765)	33.0 (-0.6; 79.1)	321,799 (288,285 - 352,467)	11.9 (7.3; 16.4)	196,258 (153,059 - 260,307)	24.7 (-3.5; 62.8)

United States of America	310,274 (259,206 - 375,080)	-32.1 (-34.4; -29.1)	108,949 (92,436 - 120,305)	-44.7 (-47.7; -40.2)	5,871,392 (5,137,554 - 6,685,063)	-2.1 (-6.5; 2.9)	2,056,104 (1,787,995 - 2,311,781)	-36.3 (-39.9; -32.9)
United States Virgin Islands	109 (94 - 126)	3.4 (-2.7; 10.1)	49 (43 - 56)	-9.5 (-25.6; 9.3)	1,243 (1,117 - 1,371)	2.6 (-1.3; 6.7)	862 (754 - 970)	-9.2 (-24.4; 7.3)
Uruguay	3,597 (3,137 - 4,156)	-28.8 (-34.4; -22.9)	2,378 (2,026 - 2,631)	-49.6 (-54.2; -44.1)	35,886 (32,251 - 40,452)	-23.0 (-27.3; -18.2)	33,894 (30,325 - 37,006)	-51.7 (-55.8; -47.0)
Uzbekistan	23,984 (20,672 - 27,529)	-4.3 (-12.4; 4.7)	10,942 (9,296 - 12,839)	42.6 (7.8; 74.3)	210,950 (185,033 - 238,508)	-3.2 (-10.3; 4.2)	289,084 (246,589 - 338,017)	23.7 (-2.9; 50.0)
Vanuatu	217 (189 - 248)	-3.3 (-9.3; 2.6)	75 (58 - 105)	2.7 (-19.1; 33.2)	2,400 (2,194 - 2,643)	-3.1 (-7.2; 1.3)	2,019 (1,600 - 2,639)	2.5 (-16.5; 28.6)
Venezuela (Bolivarian Republic of)	16,885 (14,595 - 19,542)	-24.8 (-30.4; -19.5)	6,617 (5,071 - 8,335)	-23.5 (-41.5; -0.6)	208,444 (188,138 - 229,050)	-18.3 (-21.8; -14.9)	109,807 (88,337 - 136,179)	-25.8 (-42.6; -4.6)
Viet Nam	108,805 (95,711 - 124,277)	17.4 (9.4; 26.0)	62,380 (50,288 - 73,515)	10.9 (-17.6; 51.2)	975,984 (883,223 - 1,082,719)	16.3 (10.7; 22.1)	1,163,673 (948,468 - 1,373,610)	10.1 (-18.1; 43.5)
Yemen	21,937 (19,200 - 24,782)	17.1 (9.1; 25.7)	8,921 (6,871 - 11,744)	16.4 (-7.7; 47.9)	210,911 (192,387 - 230,501)	15.9 (9.8; 21.5)	212,195 (168,265 - 276,241)	19.8 (-4.2; 51.1)
Zambia	6,889 (5,783 - 8,084)	1.1 (-5.5; 7.8)	2,670 (2,097 - 3,505)	46.8 (7.0; 96.3)	87,451 (78,117 - 97,211)	-0.3 (-4.0; 4.0)	59,119 (47,799 - 76,136)	37.2 (2.7; 75.2)
Zimbabwe	8,237 (7,185 - 9,433)	23.8 (14.9; 33.6)	2,758 (2,178 - 3,453)	13.1 (-9.7; 42.7)	88,284 (79,933 - 96,815)	17.1 (10.9; 22.4)	62,635 (50,528 - 77,417)	17.1 (-4.7; 43.9)

Supplement Table 4. Incident case, death, prevalent cases, and DALYs for intracerebral haemorrhage in 2019 and percentage change of age-standardised rates for 1990-2019, by location

Country, region	Incident cases (95% uncertainty interval)		Deaths (95% uncertainty interval)		Prevalent cases (95% uncertainty interval)		DALYs (95% uncertainty interval)	
	2019 counts	% change in age-standardised rates, 1990-2019	2019 counts	% change in age-standardised rates, 1990-2019	2019 counts	% change in age-standardised rates, 1990-2019	2019 counts	% change in age-standardised rates, 1990-2019
Countries categorised by the World Bank income level								
World Bank High Income	308,378 (271,880 - 348,654)	-40.5 (-42.0; -39.1)	250,995 (222,030 - 270,977)	-50.8 (-53.9; -48.2)	2,638,555 (2,332,603 - 2,962,446)	-16.4 (-18.8; -13.8)	4,627,021 (4,293,340 - 4,918,180)	-52.6 (-54.9; -50.3)
World Bank Low Income	247,185 (226,092 - 269,771)	-24.0 (-25.2; -22.8)	201,622 (171,478 - 236,399)	-25.5 (-33.8; -15.5)	1,390,519 (1,258,048 - 1,533,941)	-22.2 (-23.5; -20.9)	5,606,083 (4,742,940 - 6,615,200)	-28.8 (-37.6; -18.5)
World Bank Lower Middle Income	1,572,081 (1,362,821 - 1,817,697)	-15.5 (-16.8; -14.4)	1,063,780 (948,967 - 1,176,098)	-30.4 (-39.5; -21.5)	8,916,902 (7,678,004 - 10,185,253)	-10.3 (-11.2; -9.2)	28,752,186 (25,833,614 - 31,579,253)	-29.5 (-37.8; -21.5)
World Bank Upper Middle Income	1,279,232 (1,097,024 - 1,488,336)	-46.7 (-48.8; -44.5)	1,368,182 (1,210,082 - 1,529,733)	-45.1 (-54.1; -37.0)	7,703,470 (6,686,299 - 8,783,711)	-28.4 (-30.8; -25.9)	29,543,708 (26,176,015 - 33,096,898)	-47.7 (-55.7; -39.7)
High-income Asia Pacific	67,322 (58,750 - 77,001)	-53.3 (-55.7; -50.7)	50,350 (41,616 - 56,156)	-68.6 (-71.3; -65.4)	790,742 (698,770 - 894,059)	-27.7 (-30.3; -25.0)	914,146 (819,289 - 1,000,461)	-67.5 (-69.7; -63.8)
GBD regions								
Central Asia	55,139 (51,619 - 59,536)	-20.4 (-22.5; -18.1)	46,335 (42,039 - 51,002)	-0.9 (-11.4; 13.5)	304,575 (270,195 - 343,820)	-15.3 (-19.6; -10.6)	1,180,304 (1,063,959 - 1,305,921)	-7.3 (-16.7; 5.1)
East Asia	895,820 (750,140 - 1,061,516)	-52.2 (-55.5; -49.2)	1,110,098 (962,980 - 1,277,569)	-48.0 (-58.5; -37.9)	4,568,148 (3,880,482 - 5,283,908)	-31.7 (-34.8; -28.6)	23,174,853 (19,912,136 - 26,698,566)	-49.4 (-59.2; -39.1)

Southeast Asia	545,387 (481,347 - 615,944)	-17.9 (-19.2; -16.6)	395,691 (351,745 - 444,295)	-25.2 (-35.1; -15.0)	3,440,092 (2,987,671 - 3,920,596)	-12.5 (-14.0; -10.8)	10,675,277 (9,465,511 - 11,929,104)	-25.4 (-35.0; -15.4)
South Asia	815,704 (692,848 - 959,240)	-13.1 (-14.9; -11.3)	521,307 (442,787 - 608,642)	-38.2 (-48.2; -27.7)	4,142,714 (3,526,590 - 4,806,011)	-7.6 (-9.1; -6.0)	13,740,566 (11,774,657 - 15,890,550)	-35.4 (-45.5; -25.1)
Southeast Asia, East Asia, and Oceania	1,451,138 (1,252,262 - 1,686,500)	-42.4 (-45.1; -39.6)	1,511,641 (1,346,189 - 1,680,829)	-42.7 (-52.4; -34.2)	8,073,451 (6,957,888 - 9,260,076)	-23.4 (-25.9; -20.9)	34,034,347 (30,443,361 - 37,734,769)	-43.2 (-52.3; -34.5)
Oceania	9,931 (9,106 - 10,761)	-9.3 (-12.2; -6.4)	5,853 (4,419 - 7,604)	-9.5 (-24.4; 7.6)	65,211 (59,581 - 71,166)	-4.9 (-7.9; -1.8)	184,217 (141,778 - 237,200)	-8.3 (-23.8; 10.1)
Australasia	4,511 (4,024 - 5,056)	-39.6 (-43.0; -36.1)	3,726 (3,220 - 4,106)	-50.8 (-55.5; -46.4)	28,536 (25,251 - 31,890)	-23.6 (-27.1; -20.3)	59,529 (53,673 - 64,797)	-55.7 (-59.1; -52.0)
Central Europe, Eastern Europe, and Central Asia	209,077 (187,998 - 234,323)	-33.1 (-34.4; -31.9)	179,993 (163,452 - 194,210)	-33.2 (-39.4; -26.6)	1,221,154 (1,069,318 - 1,390,048)	-19.2 (-21.3; -17.3)	4,222,961 (3,867,177 - 4,548,815)	-30.2 (-36.4; -24.2)
Central Europe	50,169 (46,190 - 54,479)	-50.4 (-51.6; -49.1)	51,023 (44,453 - 57,689)	-52.9 (-59.1; -46.5)	265,589 (232,991 - 302,773)	-37.2 (-39.9; -34.8)	996,536 (868,194 - 1,125,032)	-55.5 (-61.4; -49.5)
Eastern Europe	103,769 (88,158 - 121,871)	-29.7 (-31.7; -27.8)	82,636 (73,062 - 92,611)	-30.2 (-38.7; -20.5)	650,991 (561,703 - 746,363)	-14.0 (-16.7; -11.5)	2,046,122 (1,818,373 - 2,291,724)	-23.9 (-33.2; -13.4)
Western Europe	102,863 (92,103 - 115,215)	-43.7 (-45.5; -41.6)	95,748 (84,497 - 103,419)	-52.5 (-56.2; -49.7)	638,028 (564,607 - 715,324)	-29.0 (-31.2; -26.8)	1,481,012 (1,356,134 - 1,575,799)	-58.3 (-60.7; -56.2)
Central Latin America	50,913 (45,293 - 57,528)	-37.9 (-39.6; -36.2)	33,436 (28,779 - 39,029)	-44.9 (-52.7; -35.6)	495,863 (438,123 - 556,744)	-24.6 (-26.3; -22.7)	813,578 (699,129 - 945,837)	-45.7 (-53.4; -36.1)
Tropical Latin America	76,852 (64,825 - 91,015)	-51.8 (-53.2; -50.1)	45,045 (41,807 - 47,658)	-62.4 (-65.0; -59.6)	778,562 (678,137 - 888,906)	-37.4 (-40.1; -34.6)	1,185,846 (1,120,648 - 1,249,061)	-63.8 (-66.3; -61.3)
Southern Latin America	18,105 (16,700 - 19,746)	-46.9 (-49.1; -44.4)	14,674 (13,521 - 15,950)	-57.9 (-61.9; -52.4)	133,354 (119,764 - 148,148)	-35.1 (-38.4; -31.3)	331,376 (307,858 - 355,587)	-60.3 (-63.8; -56.3)
Andean Latin America	12,355 (11,181 - 13,723)	-39.4 (-42.0; -36.6)	7,393 (6,017 - 9,142)	-59.7 (-68.0; -48.0)	131,118 (117,948 - 144,035)	-30.3 (-32.8; -27.6)	193,266 (156,232 - 238,397)	-60.6 (-69.1; -49.1)

Latin America and Caribbean	160,490 (140,673 - 182,951)	-44.4 (-45.7; -43.0)	102,260 (93,111 - 112,149)	-54.4 (-58.5; -49.6)	1,530,336 (1,349,382 - 1,720,936)	-31.7 (-33.9; -29.5)	2,611,251 (2,385,680 - 2,852,042)	-55.9 (-59.8; -51.5)
High-income North America	78,270 (65,082 - 93,140)	-18.3 (-19.8; -16.7)	64,359 (58,276 - 69,748)	-15.2 (-19.1; -9.8)	714,328 (621,859 - 813,971)	2.4 (-0.9; 5.8)	1,274,981 (1,195,203 - 1,369,691)	-17.9 (-21.2; -13.9)
North Africa and Middle East	163,240 (149,166 - 179,814)	-32.6 (-34.5; -30.6)	88,542 (77,871 - 101,100)	-51.4 (-58.1; -41.3)	1,301,257 (1,169,230 - 1,441,443)	-21.1 (-23.1; -19.2)	2,702,244 (2,343,718 - 3,120,307)	-51.7 (-58.2; -43.8)
Sub-Saharan Africa	338,403 (303,912 - 377,171)	-26.0 (-27.0; -24.9)	253,595 (217,310 - 292,845)	-27.4 (-36.4; -16.8)	2,089,990 (1,864,754 - 2,330,113)	-21.4 (-22.5; -20.1)	7,200,086 (6,126,620 - 8,346,991)	-30.4 (-39.5; -20.2)
Central Sub-Saharan Africa	41,167 (37,433 - 45,206)	-26.2 (-28.7; -23.7)	32,118 (25,030 - 41,047)	-26.8 (-40.9; -9.5)	247,049 (223,375 - 274,825)	-24.5 (-27.0; -21.6)	931,118 (731,595 - 1,171,990)	-29.6 (-43.7; -12.0)
Southern Sub-Saharan Africa	25,342 (21,821 - 29,309)	-19.2 (-22.2; -16.5)	17,480 (15,998 - 19,071)	-22.7 (-30.7; -13.0)	172,436 (150,163 - 195,857)	-20.5 (-23.6; -17.4)	469,094 (426,794 - 514,923)	-28.6 (-36.0; -20.6)
Eastern Sub-Saharan Africa	118,819 (106,849 - 132,361)	-36.4 (-37.6; -35.0)	101,232 (83,168 - 120,864)	-31.2 (-41.3; -18.6)	725,338 (646,883 - 809,738)	-30.1 (-31.6; -28.3)	2,836,113 (2,356,450 - 3,399,186)	-35.3 (-45.5; -21.8)
Western Sub-Saharan Africa	153,074 (137,381 - 171,048)	-17.8 (-19.2; -16.3)	102,766 (86,656 - 120,418)	-25.2 (-38.9; -8.7)	945,167 (841,126 - 1,053,605)	-12.7 (-13.8; -11.5)	2,963,762 (2,462,104 - 3,538,782)	-26.2 (-39.7; -10.0)
Countries in alphabetical order								
Afghanistan	11,653 (10,651 - 12,823)	-37.6 (-40.2; -34.7)	6,961 (4,904 - 9,333)	-47.4 (-60.0; -33.1)	74,850 (67,151 - 82,123)	-27.0 (-30.2; -23.5)	241,903 (176,658 - 324,979)	-46.0 (-59.2; -29.6)
Albania	2,922 (2,697 - 3,168)	-26.5 (-30.1; -23.0)	4,143 (3,233 - 5,166)	-24.6 (-41.3; -5.1)	10,356 (8,898 - 12,076)	-20.8 (-26.6; -15.3)	62,920 (48,454 - 79,015)	-31.9 (-47.3; -13.6)
Algeria	9,769 (8,701 - 10,901)	-49.8 (-53.1; -46.8)	5,276 (4,129 - 6,725)	-69.3 (-76.8; -60.1)	78,698 (70,922 - 86,722)	-39.9 (-42.7; -37.1)	142,325 (112,671 - 177,630)	-67.9 (-75.6; -58.4)
American Samoa	39 (35 - 43)	-14.8 (-19.3; -10.4)	26 (22 - 30)	-29.6 (-40.6; -15.3)	305 (275 - 335)	-13.5 (-17.0; -9.3)	693 (584 - 817)	-28.5 (-40.5; -13.9)
Andorra	13 (12 - 15)	-32.8 (-36.8; -28.8)	8 (6 - 12)	-34.2 (-55.3; -8.2)	107 (95 - 120)	-19.2 (-22.8; -15.8)	152 (111 - 209)	-37.2 (-56.2; -12.7)

Angola	8,894 (7,994 - 9,835)	-40.4 (-43.7; -37.0)	6,229 (4,794 - 7,948)	-37.4 (-51.1; -18.7)	59,191 (53,372 - 65,901)	-31.4 (-35.0; -27.7)	189,241 (145,900 - 240,563)	-40.6 (-54.6; -21.9)
Antigua and Barbuda	31 (28 - 34)	-34.1 (-37.8; -29.9)	28 (24 - 33)	-42.0 (-51.4; -31.3)	230 (205 - 256)	-22.5 (-26.1; -18.6)	651 (552 - 766)	-45.0 (-54.3; -35.1)
Argentina	12,262 (11,305 - 13,397)	-48.2 (-51.0; -44.9)	10,765 (9,752 - 11,775)	-57.0 (-61.9; -50.3)	86,542 (77,366 - 97,099)	-38.2 (-42.2; -33.5)	246,058 (224,984 - 268,013)	-59.6 (-64.0; -54.6)
Armenia	1,085 (978 - 1,219)	-49.3 (-52.9; -45.7)	730 (613 - 864)	-52.9 (-63.4; -39.3)	7,166 (6,448 - 7,943)	-30.5 (-34.7; -26.3)	15,609 (13,161 - 18,452)	-52.2 (-62.6; -40.5)
Australia	3,885 (3,465 - 4,372)	-39.8 (-43.7; -35.8)	3,106 (2,684 - 3,455)	-51.5 (-56.3; -46.7)	24,150 (21,443 - 26,956)	-24.4 (-28.0; -20.8)	49,262 (44,117 - 54,076)	-56.2 (-60.2; -52.0)
Austria	1,919 (1,729 - 2,144)	-57.3 (-60.9; -53.4)	1,280 (1,101 - 1,434)	-64.2 (-68.0; -60.3)	14,781 (13,107 - 16,397)	-33.6 (-37.4; -29.8)	20,893 (18,831 - 22,964)	-67.9 (-70.8; -64.8)
Azerbaijan	8,409 (7,726 - 9,273)	5.6 (0.2; 11.7)	6,943 (5,709 - 8,254)	29.3 (7.5; 55.9)	44,341 (38,089 - 52,098)	-5.9 (-14.1; 3.9)	156,726 (129,464 - 185,750)	4.2 (-12.9; 23.6)
Bahamas	137 (126 - 149)	-25.9 (-30.2; -21.8)	105 (86 - 130)	-32.0 (-45.9; -15.6)	1,012 (914 - 1,117)	-17.8 (-21.3; -14.0)	2,821 (2,287 - 3,499)	-31.5 (-45.3; -14.0)
Bahrain	230 (202 - 261)	-46.4 (-50.1; -43.0)	88 (70 - 113)	-61.2 (-70.0; -50.1)	2,862 (2,545 - 3,203)	-31.2 (-34.4; -28.1)	2,952 (2,394 - 3,662)	-64.2 (-72.0; -54.2)
Bangladesh	87,749 (79,199 - 97,109)	-11.5 (-16.3; -6.7)	82,323 (58,971 - 103,864)	-33.4 (-50.0; -15.3)	581,562 (506,564 - 680,541)	-6.9 (-12.1; -1.1)	1,967,203 (1,452,603 - 2,501,197)	-37.3 (-52.9; -19.6)
Barbados	111 (101 - 124)	-22.2 (-26.6; -17.7)	103 (85 - 124)	-38.2 (-50.5; -23.0)	824 (739 - 911)	-13.6 (-17.4; -9.9)	2,214 (1,808 - 2,684)	-39.3 (-51.6; -24.7)
Belarus	4,615 (4,187 - 5,154)	-26.4 (-30.4; -22.3)	3,604 (2,597 - 5,030)	-24.9 (-44.9; 1.0)	28,866 (26,024 - 31,962)	-14.9 (-18.8; -10.8)	86,858 (63,474 - 117,432)	-25.9 (-44.7; -1.9)
Belgium	2,798 (2,534 - 3,112)	-41.9 (-45.4; -37.6)	2,846 (2,448 - 3,175)	-46.4 (-52.7; -40.5)	17,040 (14,888 - 19,552)	-23.4 (-28.6; -17.6)	43,107 (38,658 - 47,316)	-53.9 (-58.3; -49.2)
Belize	95 (86 - 106)	-21.3 (-25.5; -17.0)	69 (60 - 80)	-28.5 (-40.4; -14.4)	731 (651 - 817)	-9.2 (-13.5; -4.5)	1,829 (1,582 - 2,112)	-25.1 (-37.5; -10.6)
Benin	4,523 (4,106 - 4,986)	-12.1 (-16.2; -7.7)	3,260 (2,556 - 4,120)	-18.4 (-35.5; 2.7)	26,149 (23,656 - 28,787)	-12.2 (-15.8; -7.9)	89,834 (67,872 - 116,881)	-21.2 (-38.1; 1.6)
Bermuda	18 (16 - 20)	-39.4 (-44.2; -34.7)	12 (10 - 15)	-60.7 (-69.7; -50.4)	160 (141 - 177)	-26.1 (-29.9; -22.3)	239 (198 - 290)	-61.7 (-70.1; -51.9)

Bhutan	235 (208 - 264)	-38.1 (-42.2; -34.3)	159 (117 - 208)	-45.1 (-60.1; -20.6)	1,488 (1,339 - 1,640)	-28.9 (-32.2; -25.6)	3,823 (2,828 - 5,042)	-47.2 (-62.0; -24.8)
Bolivia (Plurinational State of)	2,566 (2,292 - 2,877)	-41.8 (-45.1; -38.2)	2,197 (1,568 - 2,989)	-49.4 (-61.0; -32.7)	24,406 (21,852 - 26,992)	-33.3 (-36.9; -29.7)	55,647 (39,517 - 76,087)	-53.2 (-64.8; -37.6)
Bosnia and Herzegovina	1,142 (1,042 - 1,249)	-11.3 (-16.0; -6.0)	1,067 (848 - 1,333)	-24.4 (-40.7; -1.4)	6,082 (5,346 - 6,904)	-4.8 (-8.9; -0.1)	20,288 (16,210 - 25,643)	-33.9 (-48.5; -14.3)
Botswana	835 (759 - 923)	-26.2 (-29.8; -21.8)	705 (505 - 947)	-31.2 (-51.0; -3.7)	5,702 (5,120 - 6,400)	-21.9 (-25.8; -17.6)	20,117 (13,981 - 27,267)	-31.9 (-52.9; -3.4)
Brazil	74,671 (62,812 - 88,636)	-52.1 (-53.5; -50.5)	43,826 (40,717 - 46,438)	-62.6 (-65.3; -59.7)	757,903 (659,245 - 867,100)	-37.7 (-40.5; -34.9)	1,154,165 (1,091,358 - 1,217,146)	-64.0 (-66.6; -61.6)
Brunei Darussalam	106 (95 - 120)	-51.3 (-54.2; -48.3)	62 (54 - 71)	-60.2 (-66.4; -50.9)	1,189 (1,012 - 1,400)	-36.5 (-42.3; -29.8)	1,738 (1,514 - 1,998)	-62.2 (-68.0; -52.4)
Bulgaria	6,261 (5,839 - 6,731)	-52.2 (-54.5; -50.0)	7,608 (6,193 - 9,250)	-51.7 (-61.1; -40.4)	28,217 (24,421 - 32,875)	-36.6 (-41.0; -31.6)	147,116 (118,490 - 181,021)	-51.9 (-61.9; -40.1)
Burkina Faso	6,800 (6,128 - 7,518)	-1.0 (-5.7; 4.2)	5,551 (4,488 - 6,759)	5.7 (-17.1; 31.2)	40,416 (36,503 - 44,675)	-4.0 (-8.1; 0.7)	165,666 (130,757 - 206,271)	2.0 (-20.6; 28.0)
Burundi	3,572 (3,219 - 3,975)	-49.4 (-52.0; -46.4)	3,220 (2,352 - 4,402)	-42.3 (-56.9; -22.7)	21,294 (19,120 - 23,673)	-47.1 (-50.2; -43.1)	93,725 (69,264 - 127,978)	-46.9 (-61.6; -26.7)
CÃ´te d'Ivoire	11,106 (10,070 - 12,249)	-12.3 (-16.6; -7.7)	5,933 (4,459 - 7,568)	-18.5 (-35.6; 1.1)	67,132 (60,816 - 73,873)	-14.4 (-18.5; -9.7)	183,565 (134,814 - 236,468)	-21.5 (-40.0; 0.0)
Cabo Verde	262 (235 - 293)	-16.9 (-21.2; -12.1)	197 (164 - 246)	7.3 (-12.3; 36.8)	1,860 (1,680 - 2,043)	-13.7 (-17.6; -9.6)	4,496 (3,719 - 5,518)	-8.7 (-25.5; 14.6)
Cambodia	11,830 (10,794 - 13,019)	-23.4 (-27.1; -19.4)	8,974 (7,227 - 10,783)	-26.9 (-43.5; -11.2)	61,597 (55,771 - 68,080)	-19.6 (-23.7; -14.5)	230,370 (185,738 - 278,810)	-32.9 (-48.0; -17.4)
Cameroon	11,808 (10,723 - 12,976)	-2.5 (-7.2; 2.3)	7,733 (5,831 - 10,248)	-11.2 (-33.5; 17.3)	70,906 (64,185 - 77,773)	-2.8 (-7.7; 1.6)	229,744 (169,139 - 307,773)	-10.6 (-35.4; 21.8)
Canada	6,518 (5,811 - 7,344)	-35.6 (-40.1; -30.2)	4,610 (4,029 - 5,151)	-41.1 (-47.1; -34.9)	50,403 (44,583 - 56,164)	-21.3 (-25.3; -17.1)	80,313 (72,669 - 88,566)	-44.0 (-48.9; -38.4)
Caribbean	20,369 (18,910 - 22,016)	-16.3 (-18.2; -14.4)	16,386 (13,840 - 19,150)	-27.8 (-38.5; -16.1)	124,793 (112,206 - 137,553)	-13.3 (-15.3; -11.1)	418,562 (349,078 - 496,819)	-28.4 (-39.9; -15.5)
Central African Republic	2,720 (2,468 - 2,997)	-9.7 (-13.6; -5.6)	2,060 (1,512 - 2,764)	-16.5 (-35.6; 8.3)	14,785 (13,421 - 16,205)	-10.4 (-14.3; -6.5)	65,842 (48,208 - 87,864)	-17.8 (-37.1; 7.6)

Chad	5,639 (5,133 - 6,212)	-2.3 (-7.1; 2.3)	4,106 (3,232 - 5,223)	-0.2 (-19.9; 23.0)	32,803 (29,775 - 36,223)	1.1 (-3.3; 5.5)	126,202 (97,206 - 160,361)	-1.6 (-23.0; 24.0)
Chile	4,843 (4,350 - 5,395)	-46.6 (-50.2; -42.4)	3,110 (2,772 - 3,424)	-62.0 (-66.6; -57.4)	39,853 (35,766 - 43,989)	-30.6 (-34.3; -27.1)	69,628 (63,274 - 76,201)	-63.6 (-67.3; -59.9)
China	847,224 (702,065 - 1,009,803)	-53.1 (-56.4; -49.8)	1,069,121 (924,665 - 1,236,579)	-48.1 (-58.9; -37.8)	4,357,457 (3,691,416 - 5,053,182)	-31.9 (-35.2; -28.6)	22,210,555 (18,994,318 - 25,780,051)	-49.8 (-59.8; -38.9)
Colombia	8,944 (8,009 - 9,954)	-55.8 (-59.1; -52.4)	5,390 (4,136 - 6,905)	-67.0 (-74.3; -57.3)	89,100 (79,495 - 98,849)	-38.8 (-42.1; -35.1)	117,942 (91,591 - 150,688)	-68.3 (-75.5; -59.0)
Comoros	306 (277 - 339)	-45.5 (-48.3; -43.0)	253 (190 - 327)	-38.9 (-54.0; -3.5)	1,884 (1,697 - 2,078)	-41.3 (-44.3; -38.2)	6,385 (4,688 - 8,352)	-41.7 (-57.7; 5.0)
Congo	2,153 (1,949 - 2,363)	-37.5 (-40.7; -33.8)	1,520 (1,140 - 1,979)	-42.2 (-55.0; -26.5)	13,603 (12,289 - 15,105)	-36.6 (-39.8; -33.1)	44,257 (33,214 - 58,890)	-45.8 (-58.8; -29.2)
Cook Islands	15 (14 - 17)	-18.2 (-22.7; -13.2)	8 (7 - 10)	-48.3 (-58.1; -34.4)	121 (109 - 133)	-10.1 (-13.8; -5.8)	199 (165 - 240)	-46.6 (-57.9; -30.8)
Costa Rica	873 (781 - 980)	-30.6 (-35.3; -25.9)	450 (349 - 567)	-52.0 (-63.6; -38.1)	9,380 (8,389 - 10,342)	-15.8 (-19.5; -11.9)	10,106 (7,950 - 12,772)	-51.6 (-62.7; -38.4)
Croatia	1,536 (1,420 - 1,656)	-57.2 (-59.3; -54.8)	1,607 (1,276 - 1,995)	-60.7 (-68.7; -50.9)	8,919 (7,680 - 10,499)	-43.3 (-47.9; -38.3)	30,931 (24,690 - 38,578)	-65.6 (-72.8; -56.9)
Cuba	4,260 (3,908 - 4,688)	-34.4 (-38.2; -30.2)	3,263 (2,661 - 3,927)	-38.7 (-50.8; -24.7)	29,129 (26,152 - 32,299)	-27.5 (-31.3; -23.7)	69,760 (56,218 - 84,520)	-43.4 (-54.5; -30.9)
Cyprus	263 (237 - 291)	-47.6 (-50.8; -44.1)	216 (183 - 265)	-64.8 (-70.9; -57.3)	2,027 (1,804 - 2,256)	-26.4 (-30.8; -22.3)	3,884 (3,360 - 4,628)	-65.7 (-71.7; -58.2)
Czechia	2,687 (2,390 - 2,990)	-53.9 (-57.4; -50.1)	1,931 (1,572 - 2,318)	-70.5 (-75.9; -64.2)	18,328 (16,255 - 20,429)	-38.4 (-42.3; -34.4)	37,269 (30,647 - 44,743)	-72.8 (-77.5; -67.1)
Democratic People's Republic of Korea	36,472 (33,597 - 39,416)	-2.0 (-6.2; 2.5)	33,948 (28,750 - 40,322)	-10.1 (-27.4; 10.0)	140,518 (124,629 - 157,038)	-6.9 (-12.7; -1.1)	802,522 (654,434 - 965,792)	-8.4 (-28.6; 15.0)
Democratic Republic of the Congo	26,475 (23,991 - 29,161)	-20.8 (-24.6; -16.8)	21,650 (16,124 - 28,530)	-22.0 (-39.1; -0.5)	152,655 (137,951 - 170,132)	-22.1 (-25.6; -18.3)	613,685 (464,749 - 802,151)	-24.4 (-41.8; -2.3)
Denmark	1,220 (1,097 - 1,358)	-49.4 (-52.7; -45.7)	1,178 (1,029 - 1,316)	-53.4 (-59.0; -47.2)	8,319 (7,294 - 9,606)	-33.9 (-37.7; -29.9)	19,552 (17,537 - 21,786)	-57.5 (-62.2; -52.2)
Djibouti	387 (345 - 432)	-37.6 (-41.1; -33.9)	287 (205 - 394)	-34.4 (-51.6; -9.2)	2,726 (2,454 - 3,030)	-29.9 (-33.4; -26.3)	8,619 (5,960 - 12,105)	-35.5 (-53.7; -8.2)

Dominica	27 (25 - 30)	-20.7 (-25.1; -16.6)	23 (19 - 29)	-31.2 (-45.8; -12.3)	172 (153 - 192)	-15.3 (-19.3; -11.4)	506 (413 - 622)	-32.1 (-46.2; -13.7)
Dominican Republic	4,614 (4,236 - 5,079)	13.4 (8.1; 18.9)	3,967 (2,976 - 5,303)	3.2 (-22.9; 38.1)	28,846 (25,803 - 31,840)	10.3 (5.0; 15.2)	102,305 (73,920 - 137,644)	3.1 (-24.9; 40.7)
Ecuador	3,573 (3,245 - 3,982)	-28.5 (-33.0; -24.2)	2,041 (1,615 - 2,599)	-50.8 (-61.4; -35.6)	37,280 (33,396 - 41,427)	-21.8 (-25.4; -17.7)	53,390 (42,093 - 68,391)	-53.1 (-63.8; -38.5)
Egypt	27,106 (24,428 - 30,214)	-34.6 (-38.2; -30.4)	13,654 (9,489 - 19,503)	-56.1 (-69.1; -38.9)	191,761 (173,529 - 212,078)	-21.7 (-25.2; -18.5)	525,944 (380,648 - 714,428)	-59.8 (-70.7; -45.5)
El Salvador	1,324 (1,197 - 1,482)	-35.6 (-39.6; -31.7)	883 (662 - 1,182)	-61.7 (-72.1; -46.5)	11,478 (10,323 - 12,729)	-24.0 (-27.6; -20.7)	19,787 (14,844 - 26,771)	-65.2 (-74.8; -49.9)
Equatorial Guinea	303 (269 - 340)	-54.1 (-57.2; -50.6)	195 (128 - 279)	-57.6 (-72.4; -35.8)	2,371 (2,121 - 2,666)	-39.1 (-42.9; -34.3)	5,442 (3,563 - 7,942)	-62.8 (-76.1; -44.0)
Eritrea	2,227 (2,035 - 2,457)	-40.1 (-43.4; -36.5)	1,962 (1,467 - 2,540)	-26.0 (-45.9; 0.2)	13,120 (11,813 - 14,520)	-36.5 (-40.2; -32.5)	59,777 (43,800 - 79,523)	-32.0 (-50.2; -8.1)
Estonia	316 (283 - 358)	-64.2 (-67.3; -60.7)	201 (154 - 269)	-76.1 (-81.7; -67.9)	2,457 (2,187 - 2,756)	-44.2 (-47.8; -40.6)	4,065 (3,178 - 5,347)	-76.2 (-81.5; -69.3)
Eswatini	414 (378 - 456)	-15.5 (-19.4; -11.4)	334 (239 - 455)	-22.0 (-43.7; 9.4)	2,572 (2,338 - 2,845)	-7.1 (-12.0; -2.1)	9,437 (6,601 - 13,199)	-21.3 (-44.7; 13.1)
Ethiopia	20,784 (17,711 - 24,353)	-53.7 (-55.6; -51.4)	20,932 (15,749 - 26,061)	-50.8 (-65.5; -32.8)	141,601 (120,169 - 164,012)	-47.2 (-49.6; -44.5)	534,314 (410,425 - 666,008)	-57.8 (-71.4; -41.7)
Fiji	461 (419 - 510)	-24.4 (-28.4; -19.9)	325 (260 - 405)	-34.9 (-50.4; -11.9)	3,734 (3,349 - 4,174)	-16.8 (-20.7; -12.4)	9,237 (7,410 - 11,515)	-35.9 (-51.2; -13.2)
Finland	1,644 (1,461 - 1,852)	-23.4 (-29.3; -17.1)	1,153 (998 - 1,287)	-46.5 (-54.7; -39.6)	12,853 (11,647 - 14,098)	-16.1 (-21.8; -10.7)	19,180 (17,083 - 21,456)	-52.7 (-59.0; -47.2)
France	13,954 (12,620 - 15,566)	-40.3 (-45.2; -35.6)	12,110 (10,266 - 13,606)	-57.3 (-61.9; -52.8)	86,922 (77,656 - 96,786)	-32.3 (-36.2; -27.9)	180,449 (159,830 - 198,072)	-61.4 (-65.1; -57.9)
Gabon	623 (560 - 694)	-33.7 (-37.2; -29.9)	463 (345 - 578)	-41.7 (-55.3; -26.9)	4,446 (3,995 - 4,919)	-27.9 (-31.0; -24.6)	12,651 (9,286 - 16,102)	-43.5 (-57.6; -28.0)
Gambia	927 (843 - 1,019)	-2.8 (-7.4; 2.3)	618 (483 - 759)	5.0 (-21.7; 39.2)	5,438 (4,928 - 5,988)	-4.7 (-9.0; -0.1)	16,602 (12,480 - 20,991)	2.2 (-26.1; 39.8)
Georgia	4,138 (3,858 - 4,444)	-38.5 (-42.1; -35.2)	4,577 (3,842 - 5,367)	-38.7 (-50.2; -26.1)	20,506 (17,703 - 23,746)	-26.3 (-31.9; -20.4)	88,182 (74,389 - 103,107)	-40.1 (-50.5; -27.5)

Germany	19,015 (16,931 - 21,410)	-43.9 (-48.0; -39.8)	15,533 (13,528 - 17,349)	-57.7 (-64.5; -51.6)	128,023 (114,543 - 143,027)	-27.9 (-31.3; -24.2)	253,506 (226,661 - 277,949)	-62.0 (-66.7; -57.4)
Ghana	14,715 (13,360 - 16,188)	-0.8 (-5.7; 4.5)	9,602 (7,779 - 11,708)	-19.6 (-36.3; 2.5)	89,973 (81,244 - 99,176)	0.2 (-5.2; 5.1)	273,767 (216,264 - 337,153)	-19.5 (-37.7; 4.4)
Greece	5,732 (5,236 - 6,273)	-45.4 (-48.3; -42.2)	6,476 (5,711 - 7,078)	-52.7 (-56.6; -48.6)	26,935 (23,644 - 30,677)	-31.5 (-35.7; -27.1)	95,320 (87,016 - 102,893)	-52.5 (-56.1; -48.5)
Greenland	20 (19 - 22)	-39.7 (-42.9; -36.6)	18 (15 - 22)	-45.0 (-56.0; -32.4)	143 (122 - 167)	-28.1 (-32.8; -23.4)	425 (347 - 514)	-48.5 (-59.2; -35.7)
Grenada	39 (36 - 44)	-32.0 (-35.7; -28.0)	35 (31 - 39)	-48.7 (-57.1; -38.7)	278 (246 - 310)	-18.1 (-22.2; -13.7)	862 (767 - 966)	-50.6 (-58.7; -41.5)
Guam	102 (92 - 114)	-7.7 (-12.9; -2.2)	47 (40 - 57)	-45.1 (-55.2; -30.7)	923 (826 - 1,017)	1.6 (-4.7; 7.5)	1,382 (1,171 - 1,630)	-29.2 (-41.6; -11.6)
Guatemala	3,634 (3,317 - 4,011)	-40.6 (-44.1; -36.7)	2,366 (1,866 - 2,962)	-29.9 (-47.0; -8.0)	32,544 (28,780 - 36,839)	-34.3 (-39.0; -28.1)	62,924 (50,312 - 79,266)	-39.4 (-53.4; -21.0)
Guinea	5,390 (4,941 - 5,869)	8.1 (3.2; 13.5)	4,025 (3,202 - 4,988)	0.2 (-22.5; 28.6)	29,269 (26,666 - 32,152)	6.3 (1.3; 11.2)	114,177 (88,675 - 143,242)	-1.5 (-25.1; 28.3)
Guinea-Bissau	1,006 (923 - 1,095)	-13.6 (-17.4; -9.9)	633 (494 - 791)	-14.6 (-35.7; 16.5)	5,593 (5,098 - 6,116)	-12.5 (-16.3; -8.3)	20,038 (15,551 - 25,468)	-18.5 (-40.7; 11.2)
Guyana	453 (423 - 486)	-42.2 (-44.9; -39.1)	418 (317 - 528)	-55.4 (-66.2; -42.6)	2,561 (2,240 - 2,892)	-30.9 (-35.2; -26.5)	11,361 (8,659 - 14,569)	-56.3 (-67.2; -42.9)
Haiti	6,640 (6,090 - 7,237)	-16.4 (-19.8; -13.1)	5,229 (3,397 - 7,560)	-33.2 (-49.3; -12.0)	34,170 (30,613 - 37,844)	-19.9 (-23.8; -15.6)	153,918 (100,427 - 222,367)	-36.3 (-53.1; -14.0)
Honduras	1,471 (1,308 - 1,680)	-21.2 (-25.8; -16.2)	2,259 (1,753 - 3,003)	12.8 (-13.7; 48.8)	13,873 (12,310 - 15,521)	-13.1 (-16.9; -9.2)	57,338 (43,839 - 77,445)	-3.7 (-27.5; 29.0)
Hungary	3,347 (3,031 - 3,684)	-61.0 (-63.7; -58.2)	2,528 (2,086 - 3,022)	-73.2 (-77.8; -68.0)	20,946 (18,627 - 23,303)	-47.4 (-50.6; -44.2)	52,184 (43,512 - 62,548)	-72.4 (-77.0; -66.9)
Iceland	49 (44 - 55)	-46.1 (-49.7; -42.0)	34 (29 - 39)	-62.6 (-67.5; -56.8)	359 (315 - 407)	-26.5 (-30.4; -22.4)	563 (494 - 632)	-65.6 (-69.7; -60.7)
India	626,276 (523,149 - 748,003)	-10.1 (-12.2; -8.1)	374,136 (304,425 - 449,387)	-40.9 (-51.8; -29.5)	2,954,314 (2,497,072 - 3,437,391)	-6.8 (-8.3; -5.1)	9,879,696 (8,226,605 - 11,827,658)	-37.8 (-49.0; -26.0)
Indonesia	274,202 (231,783 - 322,944)	-4.3 (-6.0; -2.6)	181,933 (148,176 - 216,522)	-7.5 (-23.5; 8.5)	1,730,840 (1,449,818 - 2,029,835)	-5.5 (-8.0; -2.7)	4,977,770 (4,114,408 - 5,860,543)	-13.7 (-27.7; 1.3)

Iran (Islamic Republic of)	12,798 (10,809 - 15,134)	-28.0 (-30.2; -25.5)	6,308 (5,792 - 6,705)	-52.5 (-59.6; -43.2)	115,797 (99,985 - 132,722)	-16.3 (-19.5; -13.1)	172,626 (162,359 - 183,216)	-53.6 (-59.9; -47.4)
Iraq	14,714 (13,481 - 16,029)	-22.7 (-26.6; -18.3)	9,012 (7,054 - 11,186)	-28.3 (-45.3; -7.8)	110,425 (101,055 - 120,024)	-16.0 (-19.1; -12.4)	287,202 (221,961 - 364,476)	-30.0 (-47.0; -9.1)
Ireland	737 (654 - 825)	-46.0 (-49.7; -42.0)	492 (425 - 552)	-62.0 (-66.2; -57.7)	6,132 (5,452 - 6,861)	-26.6 (-30.2; -22.7)	8,672 (7,714 - 9,630)	-64.6 (-68.0; -61.0)
Israel	1,283 (1,162 - 1,428)	-51.4 (-54.6; -48.1)	1,172 (1,029 - 1,295)	-60.2 (-64.9; -55.0)	9,056 (7,939 - 10,376)	-27.2 (-31.7; -22.2)	20,205 (18,265 - 22,050)	-63.2 (-66.9; -59.3)
Italy	16,554 (13,794 - 19,860)	-41.7 (-45.1; -37.6)	18,306 (15,760 - 19,941)	-44.1 (-48.6; -41.1)	80,380 (69,649 - 91,460)	-29.4 (-32.7; -25.9)	265,919 (241,475 - 282,505)	-53.3 (-56.2; -51.1)
Jamaica	1,279 (1,170 - 1,397)	4.7 (-1.1; 11.1)	1,088 (873 - 1,333)	-21.7 (-39.2; -0.4)	8,080 (7,238 - 8,930)	12.7 (7.8; 18.0)	24,881 (19,692 - 31,018)	-24.3 (-41.1; -4.1)
Japan	50,870 (43,197 - 59,528)	-39.7 (-44.0; -34.9)	38,979 (31,743 - 43,154)	-56.9 (-60.0; -54.8)	620,775 (540,521 - 707,770)	-11.3 (-14.6; -7.6)	674,766 (597,868 - 732,610)	-51.8 (-54.0; -49.9)
Jordan	2,162 (1,916 - 2,472)	-39.6 (-43.5; -35.0)	820 (676 - 980)	-58.3 (-66.7; -47.5)	19,651 (17,293 - 22,289)	-29.2 (-33.2; -25.0)	22,856 (19,316 - 27,054)	-60.0 (-67.8; -50.4)
Kazakhstan	10,742 (9,937 - 11,735)	-27.6 (-31.8; -23.1)	8,563 (7,287 - 9,837)	-0.6 (-20.1; 27.5)	61,522 (55,417 - 69,290)	-22.1 (-26.6; -16.3)	212,589 (180,324 - 246,854)	-8.5 (-25.8; 13.0)
Kenya	15,404 (13,074 - 18,070)	-28.5 (-29.7; -27.2)	11,768 (8,658 - 14,976)	-11.8 (-22.6; 1.9)	102,689 (86,751 - 119,304)	-18.3 (-19.6; -16.9)	325,037 (243,367 - 414,416)	-11.5 (-23.4; 3.8)
Kiribati	138 (128 - 149)	-19.0 (-22.6; -15.6)	102 (80 - 126)	-23.2 (-39.5; -3.4)	894 (803 - 978)	-22.1 (-25.9; -18.1)	3,474 (2,732 - 4,288)	-26.0 (-42.4; -5.6)
Kuwait	704 (626 - 803)	-0.5 (-7.3; 7.0)	263 (207 - 319)	-3.7 (-26.2; 21.5)	7,685 (6,691 - 8,702)	-5.9 (-11.0; -1.2)	8,432 (6,893 - 10,043)	-4.2 (-23.3; 18.0)
Kyrgyzstan	2,430 (2,224 - 2,671)	-46.0 (-49.4; -42.1)	1,762 (1,520 - 2,046)	-38.4 (-48.0; -26.6)	13,716 (12,391 - 15,150)	-41.8 (-45.3; -38.0)	50,600 (43,427 - 58,892)	-39.9 (-48.7; -29.2)
Lao People's Democratic Republic	5,368 (4,935 - 5,836)	-30.2 (-33.4; -26.7)	3,775 (3,001 - 4,755)	-22.0 (-40.2; 0.7)	30,389 (27,598 - 33,436)	-29.3 (-32.8; -25.0)	107,760 (84,639 - 137,374)	-25.7 (-43.8; -2.8)
Latvia	801 (725 - 883)	-55.8 (-58.9; -52.5)	672 (557 - 813)	-63.1 (-69.9; -54.8)	4,637 (4,091 - 5,229)	-35.7 (-39.7; -31.3)	13,242 (11,039 - 16,157)	-61.8 (-68.5; -53.1)
Lebanon	979 (866 - 1,103)	-43.7 (-47.2; -40.1)	257 (188 - 351)	-69.1 (-78.1; -55.1)	8,994 (8,003 - 9,978)	-25.4 (-29.0; -21.9)	6,732 (5,020 - 8,959)	-64.0 (-73.7; -51.0)

Lesotho	1,198 (1,106 - 1,301)	10.8 (5.4; 16.3)	1,117 (833 - 1,461)	16.7 (-14.8; 54.2)	6,837 (6,221 - 7,477)	23.8 (15.0; 33.2)	30,170 (22,372 - 39,672)	21.0 (-13.1; 62.4)
Liberia	1,758 (1,596 - 1,939)	-16.0 (-20.0; -12.2)	1,076 (814 - 1,432)	-23.3 (-40.4; -2.6)	10,594 (9,559 - 11,731)	-17.5 (-21.1; -13.2)	31,221 (22,887 - 41,946)	-28.3 (-45.2; -6.5)
Libya	1,434 (1,285 - 1,592)	-31.6 (-35.3; -27.9)	678 (497 - 936)	-52.9 (-65.2; -35.6)	13,165 (11,865 - 14,473)	-21.3 (-24.3; -18.0)	22,583 (16,898 - 30,609)	-51.2 (-63.2; -34.3)
Lithuania	979 (893 - 1,090)	-29.4 (-34.1; -24.7)	663 (547 - 793)	-32.6 (-44.7; -18.1)	6,255 (5,604 - 6,919)	-17.6 (-22.4; -12.9)	14,140 (11,616 - 17,109)	-37.7 (-48.5; -24.2)
Luxembourg	105 (96 - 115)	-58.9 (-61.7; -55.9)	103 (86 - 119)	-66.7 (-71.7; -61.4)	848 (728 - 995)	-37.1 (-41.6; -32.4)	1,687 (1,450 - 1,943)	-71.0 (-75.1; -66.5)
Madagascar	15,682 (14,384 - 17,198)	-19.1 (-22.5; -15.6)	11,051 (7,973 - 14,449)	-13.5 (-33.3; 13.1)	88,879 (80,519 - 97,611)	-21.4 (-24.8; -17.7)	351,782 (256,736 - 460,146)	-18.5 (-38.4; 6.4)
Malawi	4,504 (4,065 - 5,010)	-25.6 (-29.7; -21.6)	3,893 (2,943 - 4,893)	-27.9 (-42.5; -8.9)	26,942 (24,296 - 29,984)	-20.2 (-23.6; -16.6)	108,839 (82,260 - 140,131)	-29.6 (-46.1; -7.5)
Malaysia	17,232 (15,682 - 19,260)	-40.3 (-43.7; -36.6)	10,792 (8,541 - 13,477)	-51.4 (-61.2; -37.9)	133,211 (120,677 - 147,595)	-30.0 (-33.6; -26.7)	281,125 (225,294 - 347,130)	-53.5 (-62.3; -41.2)
Maldives	145 (130 - 161)	-58.5 (-61.1; -55.8)	78 (64 - 93)	-67.1 (-73.3; -59.7)	1,229 (1,102 - 1,370)	-49.3 (-51.9; -46.4)	2,106 (1,739 - 2,476)	-71.3 (-76.5; -65.2)
Mali	7,360 (6,722 - 8,048)	-21.4 (-25.1; -17.5)	5,525 (4,392 - 6,924)	-18.4 (-34.6; 1.6)	43,496 (39,145 - 48,411)	-19.6 (-23.5; -15.1)	162,992 (126,576 - 208,830)	-23.3 (-39.1; -2.6)
Malta	114 (102 - 129)	-46.0 (-49.6; -42.7)	86 (73 - 99)	-66.0 (-70.7; -61.0)	744 (660 - 829)	-30.1 (-33.8; -26.1)	1,517 (1,314 - 1,729)	-65.9 (-70.5; -61.0)
Marshall Islands	58 (53 - 63)	-8.3 (-12.4; -3.5)	36 (26 - 48)	-16.3 (-33.9; 6.5)	366 (334 - 400)	-5.0 (-9.0; -0.4)	1,161 (848 - 1,542)	-13.9 (-32.9; 11.1)
Mauritania	1,502 (1,355 - 1,675)	-30.8 (-34.2; -26.8)	858 (664 - 1,087)	-42.0 (-55.0; -26.2)	9,631 (8,648 - 10,643)	-28.0 (-31.5; -24.2)	22,058 (16,414 - 28,828)	-47.3 (-60.3; -30.8)
Mauritius	666 (602 - 744)	-51.6 (-54.5; -48.8)	393 (320 - 479)	-63.5 (-70.8; -54.8)	5,438 (4,897 - 6,031)	-37.0 (-40.9; -33.3)	10,865 (8,828 - 13,212)	-62.2 (-69.4; -53.6)
Mexico	23,267 (19,686 - 27,359)	-32.0 (-33.3; -30.4)	13,214 (11,367 - 15,172)	-42.5 (-50.4; -34.1)	239,367 (207,712 - 273,612)	-18.4 (-20.5; -16.5)	332,727 (286,516 - 384,253)	-41.2 (-49.5; -32.3)
Micronesia (Federated States of)	98 (90 - 107)	-21.7 (-25.1; -18.2)	75 (52 - 98)	-18.2 (-41.5; 9.9)	635 (577 - 694)	-21.2 (-24.6; -17.5)	2,321 (1,549 - 3,091)	-19.8 (-44.9; 10.0)

Monaco	10 (9 - 11)	-15.0 (-19.6; -9.5)	8 (6 - 10)	-54.9 (-66.2; -35.2)	67 (59 - 74)	-7.4 (-10.6; -3.8)	135 (107 - 167)	-53.2 (-65.4; -32.5)
Mongolia	4,088 (3,827 - 4,395)	-37.7 (-40.9; -34.7)	3,457 (2,706 - 4,455)	15.3 (-13.2; 48.0)	20,003 (17,515 - 22,852)	-25.7 (-31.3; -19.4)	98,265 (76,696 - 127,640)	2.0 (-23.6; 33.4)
Montenegro	1,170 (1,084 - 1,269)	-14.1 (-17.7; -9.9)	1,456 (1,241 - 1,662)	-3.5 (-19.4; 12.5)	3,879 (3,312 - 4,498)	-16.9 (-21.8; -11.9)	24,098 (20,572 - 27,780)	-11.9 (-27.1; 3.9)
Morocco	10,826 (9,693 - 12,112)	-40.0 (-43.3; -36.3)	6,700 (5,090 - 8,430)	-51.2 (-63.6; -34.6)	81,150 (73,393 - 90,013)	-29.0 (-31.8; -26.0)	181,455 (138,916 - 232,630)	-51.0 (-64.1; -34.3)
Mozambique	13,630 (12,382 - 15,010)	-4.1 (-8.5; 0.6)	9,990 (7,490 - 12,823)	-1.5 (-25.0; 30.0)	72,748 (65,876 - 80,103)	5.2 (0.1; 10.2)	289,416 (216,516 - 371,292)	2.8 (-23.5; 37.8)
Myanmar	32,277 (29,550 - 35,720)	-31.9 (-35.5; -27.8)	46,992 (39,655 - 56,055)	-35.1 (-48.3; -17.1)	188,608 (170,916 - 209,217)	-24.5 (-28.7; -19.9)	1,237,216 (1,012,481 - 1,508,356)	-40.9 (-54.6; -22.1)
Namibia	727 (661 - 804)	-40.5 (-44.0; -36.9)	740 (576 - 951)	-32.7 (-48.7; -8.0)	4,892 (4,287 - 5,614)	-29.8 (-34.9; -23.9)	18,255 (13,947 - 23,802)	-35.4 (-52.2; -9.5)
Nauru	7 (6 - 8)	-9.2 (-13.3; -5.2)	5 (4 - 7)	-17.4 (-32.8; 0.9)	50 (45 - 54)	-11.9 (-15.4; -8.1)	197 (148 - 256)	-15.5 (-31.5; 3.9)
Nepal	8,098 (7,201 - 9,089)	-26.8 (-30.5; -22.7)	7,854 (5,742 - 10,214)	-34.1 (-53.3; -10.4)	47,969 (42,725 - 53,419)	-21.5 (-24.8; -18.0)	191,376 (142,270 - 247,290)	-38.4 (-55.5; -16.7)
Netherlands	3,720 (3,352 - 4,164)	-38.2 (-42.3; -33.5)	3,145 (2,741 - 3,505)	-43.0 (-48.6; -36.8)	25,553 (22,615 - 28,441)	-20.0 (-23.7; -15.9)	50,948 (45,889 - 55,987)	-49.1 (-53.6; -44.0)
New Zealand	626 (532 - 722)	-39.5 (-45.5; -33.1)	620 (530 - 689)	-47.0 (-52.5; -41.0)	4,386 (3,795 - 5,003)	-19.6 (-25.8; -13.2)	10,267 (9,172 - 11,289)	-53.1 (-57.6; -48.3)
Nicaragua	1,082 (968 - 1,234)	-37.6 (-41.7; -33.4)	781 (645 - 933)	-39.7 (-51.2; -24.6)	11,113 (9,943 - 12,322)	-24.9 (-28.4; -21.2)	18,186 (14,876 - 22,093)	-47.1 (-57.6; -32.9)
Niger	6,944 (6,286 - 7,697)	-13.6 (-17.7; -9.4)	5,122 (3,893 - 6,701)	-15.2 (-32.4; 6.5)	40,536 (36,737 - 44,660)	-16.3 (-19.7; -12.4)	159,491 (117,184 - 213,519)	-20.5 (-38.0; 3.0)
Nigeria	60,092 (51,125 - 70,405)	-28.6 (-29.9; -27.5)	39,992 (31,129 - 51,487)	-37.8 (-56.3; -14.0)	391,260 (332,179 - 451,330)	-18.3 (-19.5; -16.8)	1,116,322 (863,417 - 1,458,048)	-38.3 (-55.6; -13.8)
Niue	2 (1 - 2)	-15.1 (-19.4; -10.6)	1 (1 - 2)	-30.1 (-45.1; -11.4)	11 (10 - 12)	-10.7 (-14.3; -6.3)	33 (26 - 41)	-29.5 (-46.0; -9.5)
North Macedonia	1,386 (1,286 - 1,509)	-31.0 (-34.0; -27.6)	1,648 (1,315 - 1,998)	-26.1 (-40.7; -8.5)	6,883 (5,962 - 7,971)	-24.1 (-29.5; -18.7)	34,980 (27,894 - 43,073)	-33.0 (-46.3; -16.1)

Northern Ireland	364 (329 - 404)	-34.4 (-38.3; -30.5)	345 (302 - 382)	-40.0 (-46.9; -33.3)	2,300 (2,027 - 2,587)	-17.4 (-21.7; -12.5)	5,628 (5,049 - 6,192)	-48.6 (-54.2; -43.0)
Northern Mariana Islands	40 (35 - 45)	-20.8 (-25.2; -16.2)	26 (22 - 30)	-39.3 (-50.0; -27.2)	330 (296 - 367)	-19.6 (-23.5; -15.8)	743 (627 - 868)	-40.5 (-51.5; -27.3)
Norway	1,051 (868 - 1,269)	-28.5 (-32.1; -24.7)	756 (662 - 834)	-49.4 (-53.1; -45.4)	8,072 (6,978 - 9,183)	-1.4 (-5.2; 2.3)	11,895 (10,915 - 12,906)	-55.6 (-58.5; -52.8)
Oman	669 (590 - 756)	-39.3 (-43.1; -35.4)	323 (276 - 408)	-56.6 (-67.7; -40.0)	7,278 (6,350 - 8,298)	-30.2 (-33.6; -26.2)	10,843 (9,315 - 13,404)	-59.8 (-69.5; -46.5)
Pakistan	93,345 (79,368 - 109,990)	-16.5 (-18.9; -14.1)	56,835 (47,311 - 69,008)	-15.5 (-31.6; 9.3)	557,382 (469,864 - 647,582)	-9.7 (-12.7; -6.7)	1,698,469 (1,418,193 - 2,051,124)	-12.5 (-30.2; 13.1)
Palau	17 (15 - 19)	-10.0 (-14.6; -5.2)	12 (10 - 15)	-27.6 (-47.4; -2.6)	131 (119 - 145)	-7.7 (-11.6; -3.4)	377 (297 - 478)	-25.2 (-46.6; 2.2)
Palestine	727 (650 - 832)	-38.8 (-42.9; -34.8)	468 (395 - 549)	-55.6 (-65.6; -42.4)	6,105 (5,301 - 7,123)	-21.6 (-26.1; -16.1)	12,183 (10,390 - 14,189)	-56.7 (-66.5; -43.0)
Panama	1,016 (917 - 1,139)	-36.3 (-40.4; -32.5)	622 (475 - 797)	-52.9 (-65.5; -38.4)	9,031 (8,161 - 9,989)	-20.1 (-23.9; -16.5)	13,976 (10,846 - 18,060)	-53.2 (-65.2; -38.8)
Papua New Guinea	7,397 (6,760 - 8,061)	-8.7 (-12.7; -4.8)	3,964 (2,703 - 5,587)	-2.8 (-23.8; 22.0)	48,047 (43,906 - 52,550)	-3.1 (-7.3; 1.2)	126,735 (88,220 - 173,954)	-2.2 (-24.4; 26.0)
Paraguay	2,181 (1,968 - 2,434)	-33.8 (-38.1; -29.3)	1,219 (926 - 1,620)	-51.4 (-64.2; -33.0)	20,659 (18,705 - 22,673)	-21.8 (-25.5; -18.0)	31,680 (24,168 - 42,289)	-49.9 (-63.2; -30.3)
Peru	6,217 (5,610 - 6,957)	-43.5 (-46.7; -39.6)	3,155 (2,365 - 4,247)	-67.9 (-77.4; -53.5)	69,432 (62,567 - 76,363)	-33.2 (-36.4; -29.9)	84,228 (62,568 - 113,046)	-67.3 (-76.9; -53.5)
Philippines	70,640 (59,564 - 82,830)	-10.8 (-11.9; -9.7)	44,662 (35,321 - 53,185)	36.8 (3.8; 68.7)	451,992 (384,158 - 524,842)	-8.9 (-10.7; -7.0)	1,311,982 (1,043,209 - 1,555,693)	60.3 (10.1; 98.4)
Poland	11,144 (9,258 - 13,262)	-49.5 (-50.8; -48.0)	9,891 (8,283 - 11,495)	-57.7 (-64.5; -50.7)	67,976 (58,914 - 78,001)	-35.0 (-37.9; -32.0)	208,587 (174,286 - 244,451)	-57.3 (-64.4; -50.2)
Portugal	3,623 (3,333 - 3,940)	-67.8 (-69.6; -65.9)	4,236 (3,757 - 4,658)	-71.1 (-73.9; -68.3)	23,177 (20,095 - 26,769)	-47.2 (-51.3; -42.6)	69,582 (63,062 - 75,687)	-72.8 (-75.1; -70.4)
Puerto Rico	939 (847 - 1,051)	-37.8 (-42.1; -33.3)	632 (489 - 794)	-46.6 (-59.1; -31.5)	7,140 (6,274 - 7,978)	-24.7 (-29.3; -20.5)	12,181 (9,422 - 15,584)	-46.5 (-58.7; -31.3)
Qatar	385 (328 - 454)	-34.0 (-38.3; -29.3)	77 (57 - 106)	-49.3 (-64.0; -28.1)	5,075 (4,419 - 5,756)	-28.6 (-32.2; -24.8)	3,518 (2,730 - 4,574)	-56.3 (-67.8; -41.8)

Republic of Korea	15,113 (13,645 - 17,001)	-77.2 (-78.9; -75.5)	10,760 (9,332 - 13,439)	-87.0 (-88.8; -81.8)	153,285 (136,106 - 172,451)	-63.7 (-66.2; -60.9)	223,519 (198,962 - 283,064)	-87.1 (-88.6; -81.7)
Republic of Moldova	2,186 (2,024 - 2,387)	-53.5 (-56.1; -50.5)	1,877 (1,626 - 2,161)	-58.0 (-64.8; -51.0)	11,572 (10,346 - 13,059)	-42.7 (-46.6; -38.5)	44,805 (39,096 - 51,579)	-56.1 (-62.8; -49.0)
Romania	12,286 (11,485 - 13,220)	-48.0 (-50.6; -45.5)	12,916 (10,776 - 15,405)	-40.1 (-52.5; -23.2)	59,382 (51,609 - 68,546)	-33.8 (-38.9; -28.3)	256,687 (213,016 - 310,221)	-42.9 (-54.7; -28.8)
Russian Federation	70,428 (59,016 - 83,399)	-32.8 (-34.4; -31.3)	58,086 (49,731 - 66,985)	-34.7 (-43.3; -25.3)	439,730 (375,310 - 510,371)	-16.3 (-18.4; -14.1)	1,401,942 (1,207,058 - 1,621,975)	-28.9 (-38.6; -16.4)
Rwanda	3,841 (3,484 - 4,245)	-61.3 (-63.7; -58.8)	3,670 (2,552 - 5,024)	-51.9 (-65.3; -37.9)	23,292 (20,603 - 26,659)	-57.9 (-61.4; -53.0)	96,466 (67,820 - 130,977)	-58.5 (-70.1; -45.3)
Saint Kitts and Nevis	29 (27 - 32)	-49.1 (-51.7; -46.5)	28 (23 - 34)	-58.4 (-67.0; -49.3)	203 (178 - 231)	-36.3 (-39.7; -31.8)	721 (562 - 879)	-60.3 (-69.8; -50.4)
Saint Lucia	76 (71 - 83)	-40.1 (-43.4; -36.7)	65 (55 - 77)	-49.5 (-59.2; -38.9)	525 (467 - 593)	-26.9 (-30.3; -23.3)	1,528 (1,283 - 1,814)	-50.1 (-59.3; -39.5)
Saint Vincent and the Grenadines	55 (50 - 60)	42.2 (34.4; 50.2)	46 (40 - 52)	14.4 (-1.8; 33.8)	346 (310 - 385)	32.0 (25.0; 40.7)	1,093 (938 - 1,260)	10.8 (-6.4; 30.9)
Samoa	156 (144 - 171)	-17.6 (-21.6; -13.5)	113 (91 - 138)	-24.8 (-40.1; -2.9)	1,058 (958 - 1,172)	-11.8 (-15.7; -7.5)	3,067 (2,359 - 3,833)	-22.6 (-40.8; 3.1)
San Marino	7 (6 - 8)	-27.2 (-31.4; -22.4)	6 (4 - 9)	-37.3 (-59.9; -8.8)	47 (42 - 52)	-14.0 (-17.5; -10.2)	99 (67 - 139)	-37.5 (-60.3; -7.0)
Sao Tome and Principe	129 (118 - 142)	0.4 (-4.4; 5.7)	64 (50 - 79)	-4.7 (-24.8; 24.1)	834 (756 - 918)	-0.8 (-5.1; 4.0)	1,794 (1,403 - 2,245)	-5.5 (-26.7; 26.4)
Saudi Arabia	9,675 (8,851 - 10,619)	-6.0 (-11.1; -0.8)	5,292 (3,981 - 6,647)	-46.3 (-60.6; -22.4)	153,819 (128,262 - 183,295)	-5.3 (-10.8; 0.0)	192,664 (149,104 - 243,126)	-41.3 (-57.6; -15.5)
Scotland	1,438 (1,290 - 1,593)	-32.7 (-37.0; -28.1)	1,356 (1,212 - 1,496)	-36.6 (-43.4; -29.4)	9,340 (8,296 - 10,595)	-20.5 (-25.4; -15.8)	22,753 (20,721 - 25,056)	-45.0 (-50.3; -39.2)
Senegal	5,639 (5,087 - 6,240)	-15.5 (-19.7; -10.8)	3,892 (3,074 - 4,861)	-14.2 (-32.0; 8.8)	35,444 (31,887 - 39,188)	-15.6 (-19.2; -11.7)	105,268 (81,024 - 133,040)	-18.9 (-38.3; 5.5)
Serbia	4,261 (3,932 - 4,638)	-50.0 (-52.8; -47.1)	4,459 (3,625 - 5,474)	-53.0 (-63.0; -40.9)	21,923 (18,807 - 25,443)	-35.2 (-40.9; -30.1)	85,105 (68,658 - 104,441)	-59.5 (-68.4; -49.0)
Seychelles	66 (60 - 74)	-23.8 (-27.8; -19.7)	32 (27 - 38)	-42.6 (-51.0; -32.1)	483 (434 - 535)	-16.4 (-20.2; -12.3)	933 (806 - 1,094)	-42.5 (-50.5; -33.1)

Sierra Leone	3,788 (3,476 - 4,147)	-2.1 (-6.7; 3.1)	2,384 (1,754 - 3,191)	-8.1 (-28.7; 20.4)	22,165 (20,034 - 24,343)	-5.9 (-10.2; -1.8)	74,347 (53,260 - 101,412)	-10.0 (-32.6; 20.5)
Singapore	1,233 (1,106 - 1,392)	-62.0 (-65.1; -58.2)	550 (484 - 606)	-74.7 (-77.4; -72.0)	15,492 (13,898 - 17,438)	-44.4 (-48.6; -40.3)	14,123 (12,615 - 15,528)	-74.9 (-77.3; -72.6)
Slovakia	1,514 (1,386 - 1,660)	-50.7 (-53.8; -47.6)	1,308 (1,031 - 1,631)	-62.8 (-70.8; -52.6)	9,688 (8,549 - 10,967)	-37.9 (-41.8; -34.2)	28,346 (22,337 - 35,212)	-63.8 (-71.8; -53.7)
Slovenia	512 (465 - 571)	-54.4 (-57.5; -51.0)	460 (354 - 605)	-58.4 (-70.8; -42.3)	3,009 (2,607 - 3,458)	-40.4 (-45.0; -35.3)	8,025 (6,247 - 10,264)	-66.3 (-76.3; -53.3)
Solomon Islands	602 (552 - 655)	-13.0 (-16.9; -9.6)	598 (481 - 742)	1.5 (-22.1; 29.6)	3,423 (3,124 - 3,724)	-13.0 (-17.4; -8.8)	18,904 (14,987 - 23,922)	-2.7 (-26.7; 26.3)
Somalia	7,196 (6,564 - 7,933)	-32.3 (-35.2; -29.5)	5,538 (3,863 - 7,702)	-25.2 (-45.0; 3.7)	36,627 (33,009 - 40,267)	-31.2 (-34.7; -27.2)	172,727 (119,967 - 237,835)	-28.3 (-48.0; 1.7)
South Africa	19,042 (15,976 - 22,672)	-24.0 (-27.6; -20.6)	12,441 (11,492 - 13,367)	-25.5 (-33.5; -15.8)	132,316 (113,988 - 151,595)	-26.0 (-29.4; -22.5)	328,098 (301,983 - 354,517)	-34.6 (-41.3; -26.9)
South Sudan	2,857 (2,558 - 3,159)	-30.9 (-34.6; -26.8)	1,925 (1,319 - 2,629)	-35.3 (-52.6; -10.7)	19,496 (17,500 - 21,581)	-23.7 (-27.1; -19.7)	54,365 (36,959 - 75,970)	-37.9 (-55.6; -11.9)
Spain	11,186 (10,127 - 12,385)	-52.2 (-55.4; -48.9)	11,171 (9,597 - 12,626)	-60.0 (-63.7; -55.5)	70,271 (61,527 - 80,241)	-36.6 (-40.1; -32.2)	167,589 (149,850 - 183,438)	-65.4 (-68.3; -62.1)
Sri Lanka	7,254 (6,538 - 8,063)	-34.8 (-38.5; -30.9)	4,020 (2,919 - 5,366)	-50.8 (-64.9; -32.3)	60,030 (53,858 - 66,452)	-20.1 (-23.4; -16.1)	103,583 (77,762 - 137,317)	-47.5 (-61.6; -29.8)
Suriname	353 (325 - 386)	-14.3 (-18.5; -10.3)	294 (244 - 354)	-19.3 (-33.8; -2.8)	2,081 (1,877 - 2,310)	-10.8 (-14.6; -6.5)	7,476 (6,169 - 9,093)	-20.2 (-35.0; -2.7)
Sweden	2,802 (2,285 - 3,417)	-26.2 (-30.2; -22.1)	1,920 (1,698 - 2,139)	-41.3 (-46.4; -35.4)	19,769 (17,155 - 22,468)	-3.8 (-9.2; 1.6)	29,965 (27,250 - 32,821)	-46.3 (-50.3; -42.2)
Switzerland	1,368 (1,213 - 1,538)	-53.5 (-57.6; -49.8)	1,035 (871 - 1,219)	-63.9 (-70.0; -56.9)	10,112 (8,967 - 11,294)	-32.7 (-36.3; -29.0)	15,387 (13,373 - 17,487)	-67.2 (-71.8; -62.0)
Syrian Arab Republic	5,745 (5,231 - 6,338)	-34.0 (-37.8; -30.4)	3,895 (2,952 - 5,174)	-42.3 (-58.3; -20.1)	42,182 (38,241 - 46,305)	-30.1 (-33.2; -26.4)	106,469 (80,729 - 141,598)	-48.5 (-62.7; -28.8)
Taiwan (Province of China)	12,124 (10,894 - 13,473)	-60.6 (-63.5; -57.8)	7,028 (5,614 - 8,893)	-74.8 (-79.8; -68.0)	70,173 (60,705 - 79,824)	-43.8 (-48.2; -39.1)	161,776 (128,933 - 203,518)	-72.5 (-77.9; -65.2)
Tajikistan	4,023 (3,639 - 4,494)	-11.6 (-16.6; -6.2)	4,727 (3,774 - 5,802)	25.2 (3.5; 52.2)	20,731 (18,371 - 23,422)	-20.6 (-25.7; -14.0)	117,387 (93,577 - 145,141)	9.2 (-10.9; 33.0)

Thailand	39,880 (36,214 - 43,865)	-47.1 (-50.5; -43.8)	23,739 (17,798 - 31,055)	-59.9 (-70.4; -46.3)	300,248 (270,997 - 331,238)	-29.4 (-33.2; -25.4)	621,631 (467,737 - 811,195)	-52.8 (-64.9; -37.1)
Timor-Leste	909 (834 - 997)	-7.8 (-12.3; -3.2)	663 (493 - 884)	10.3 (-16.5; 40.8)	5,188 (4,695 - 5,738)	-5.4 (-9.5; -1.2)	17,394 (12,949 - 23,483)	9.4 (-20.3; 41.0)
Togo	3,683 (3,354 - 4,050)	-6.5 (-10.8; -2.2)	2,193 (1,697 - 2,793)	-11.3 (-29.0; 12.8)	21,653 (19,604 - 23,817)	-10.1 (-14.0; -6.0)	66,137 (49,756 - 84,906)	-13.4 (-32.2; 11.8)
Tokelau	1 (1 - 1)	-21.3 (-25.2; -17.4)	1 (1 - 1)	-35.8 (-50.1; -15.6)	7 (6 - 8)	-12.8 (-16.8; -8.9)	21 (16 - 27)	-34.2 (-50.7; -11.5)
Tonga	46 (42 - 51)	-21.3 (-25.3; -17.3)	31 (26 - 38)	-19.8 (-38.1; 5.4)	332 (300 - 367)	-15.8 (-19.1; -12.4)	735 (602 - 904)	-19.2 (-37.2; 5.7)
Trinidad and Tobago	479 (436 - 530)	-39.1 (-42.5; -35.2)	385 (287 - 505)	-54.0 (-65.8; -38.5)	3,736 (3,348 - 4,158)	-23.0 (-26.6; -19.0)	9,143 (6,784 - 12,169)	-54.4 (-66.6; -38.4)
Tunisia	2,884 (2,533 - 3,290)	-37.5 (-41.4; -33.6)	1,692 (1,254 - 2,247)	-59.4 (-71.5; -41.3)	16,645 (14,281 - 18,819)	2.0 (-6.9; 12.1)	40,607 (29,901 - 53,483)	-56.5 (-69.3; -38.5)
Turkey	30,027 (26,923 - 33,453)	-12.4 (-18.3; -6.4)	15,611 (12,430 - 19,184)	-30.5 (-49.0; -9.9)	212,849 (190,914 - 233,873)	-15.9 (-19.7; -11.7)	350,826 (284,141 - 427,496)	-36.9 (-54.1; -20.0)
Turkmenistan	3,342 (3,063 - 3,671)	-13.4 (-17.8; -8.8)	2,308 (1,823 - 2,939)	-3.5 (-23.9; 25.4)	18,807 (17,117 - 20,771)	0.9 (-3.7; 5.8)	68,527 (54,173 - 86,418)	-0.6 (-21.8; 27.2)
Tuvalu	10 (9 - 10)	-17.9 (-21.5; -14.0)	9 (7 - 11)	-31.9 (-49.1; -8.2)	62 (56 - 68)	-16.1 (-19.5; -12.5)	246 (190 - 314)	-32.1 (-49.9; -6.5)
Uganda	8,819 (7,963 - 9,872)	-38.4 (-41.8; -34.7)	7,844 (5,826 - 10,045)	-22.0 (-39.3; -1.8)	53,409 (47,817 - 59,867)	-28.9 (-32.6; -24.7)	219,355 (166,180 - 283,595)	-23.8 (-41.7; -0.6)
Ukraine	24,442 (20,541 - 29,097)	-15.7 (-20.0; -11.0)	17,533 (14,901 - 20,934)	-7.3 (-26.1; 26.7)	157,473 (135,396 - 180,493)	-2.5 (-9.6; 4.8)	481,070 (406,460 - 574,787)	3.9 (-16.0; 35.9)
United Arab Emirates	2,561 (2,257 - 2,883)	-41.9 (-45.3; -38.6)	812 (534 - 1,257)	-67.8 (-77.7; -54.3)	24,981 (22,321 - 28,054)	-30.3 (-33.4; -27.3)	35,021 (23,807 - 53,069)	-64.1 (-75.4; -49.2)
United Kingdom	13,603 (11,523 - 16,078)	-33.4 (-35.9; -30.6)	12,394 (11,150 - 13,236)	-36.8 (-40.3; -33.4)	85,877 (74,742 - 97,371)	-21.5 (-23.7; -19.5)	199,515 (184,841 - 212,121)	-44.3 (-47.0; -42.2)
United Republic of Tanzania	15,125 (13,588 - 16,915)	-26.8 (-30.6; -22.7)	13,112 (9,965 - 17,066)	-27.4 (-42.6; -8.4)	92,605 (83,648 - 102,595)	-17.8 (-21.5; -13.8)	354,693 (268,158 - 470,397)	-31.5 (-47.4; -11.2)
United States of America	71,731 (58,955 - 86,204)	-16.4 (-18.0; -14.7)	59,731 (54,339 - 64,894)	-12.3 (-16.5; -6.7)	663,770 (577,066 - 758,827)	4.9 (1.4; 8.4)	1,194,222 (1,119,887 - 1,285,472)	-15.3 (-18.9; -11.1)

United States Virgin Islands	45 (40 - 49)	-11.0 (-15.6; -6.2)	40 (34 - 46)	-19.8 (-35.2; -0.4)	342 (305 - 385)	-3.3 (-7.6; 0.9)	894 (757 - 1,047)	-20.8 (-37.0; 0.1)
Uruguay	999 (908 - 1,103)	-27.8 (-32.0; -23.3)	798 (710 - 878)	-41.1 (-48.0; -32.8)	6,953 (6,243 - 7,748)	-16.1 (-20.6; -11.5)	15,673 (14,220 - 17,077)	-46.5 (-52.5; -39.1)
Uzbekistan	16,884 (15,692 - 18,335)	-0.7 (-6.1; 4.8)	13,269 (11,219 - 15,708)	36.9 (15.5; 113.0)	97,782 (85,260 - 111,698)	2.6 (-6.1; 11.3)	372,418 (315,417 - 442,897)	19.5 (-0.3; 63.9)
Vanuatu	275 (254 - 299)	-14.4 (-18.3; -10.2)	196 (147 - 265)	-7.8 (-28.9; 22.4)	1,702 (1,548 - 1,858)	-14.1 (-17.9; -9.8)	5,989 (4,456 - 8,074)	-4.8 (-27.8; 31.0)
Venezuela (Bolivarian Republic of)	9,303 (8,469 - 10,298)	-31.7 (-35.3; -27.9)	7,471 (5,704 - 9,679)	-29.7 (-46.9; -6.8)	79,976 (72,258 - 87,793)	-22.9 (-26.1; -19.1)	180,593 (136,617 - 235,794)	-31.4 (-48.3; -9.0)
Viet Nam	84,203 (77,602 - 91,469)	-10.7 (-14.9; -6.5)	69,121 (55,195 - 85,539)	-23.5 (-42.4; 1.5)	466,333 (420,977 - 521,235)	-3.9 (-8.7; 2.5)	1,758,557 (1,408,285 - 2,184,317)	-22.6 (-42.7; 3.3)
Yemen	7,447 (6,770 - 8,165)	-45.8 (-48.6; -42.9)	4,607 (3,395 - 6,206)	-55.8 (-66.7; -40.7)	51,230 (46,578 - 56,482)	-37.0 (-39.8; -33.6)	146,684 (108,214 - 195,409)	-53.7 (-65.8; -36.8)
Zambia	4,390 (3,980 - 4,845)	-28.9 (-32.3; -25.0)	5,703 (4,464 - 7,109)	2.2 (-22.8; 30.9)	27,446 (24,792 - 30,636)	-24.3 (-28.0; -20.3)	158,344 (122,374 - 199,845)	0.0 (-25.6; 29.3)
Zimbabwe	3,126 (2,826 - 3,479)	23.9 (17.5; 30.5)	2,143 (1,645 - 2,787)	-1.0 (-23.9; 27.9)	20,119 (18,178 - 22,246)	17.8 (8.6; 25.5)	63,017 (48,215 - 81,480)	5.1 (-19.3; 36.0)

Supplement Table 5. Incident case, death, prevalent cases, and DALYs for subarachnoid haemorrhage in 2019 and percentage change of age-standardised rates for 1990-2019, by location

Country, region	Incident cases (95% uncertainty interval)		Deaths (95% uncertainty interval)		Prevalent cases (95% uncertainty interval)		DALYs (95% uncertainty interval)	
	2019 counts	% change in age-standardised rates, 1990-2019	2019 counts	% change in age-standardised rates, 1990-2019	2019 counts	% change in age-standardised rates, 1990-2019	2019 counts	% change in age-standardised rates, 1990-2019
Countries categorised by the World Bank income level								
World Bank High Income	381,195 (322,942 - 451,001)	6.6 (3.9; 9.4)	74,363 (66,257 - 78,990)	-31.6 (-36.0; -27.5)	2,887,399 (2,461,560 - 3,411,651)	3.7 (-0.1; 6.2)	1,989,305 (1,819,308 - 2,157,535)	-34.2 (-37.4; -29.0)
World Bank Low Income	40,071 (33,772 - 47,591)	-13.3 (-15.1; -11.4)	14,688 (7,869 - 28,454)	-31.4 (-44.3; -9.7)	249,558 (213,482 - 291,548)	-7.9 (-9.3; -6.2)	524,079 (302,774 - 1,007,106)	-30.0 (-42.5; -7.8)
World Bank Lower Middle Income	319,427 (270,733 - 378,327)	-20.9 (-22.4; -19.5)	124,528 (89,660 - 161,772)	-38.6 (-52.3; -6.4)	2,141,555 (1,822,598 - 2,537,867)	-14.5 (-16.1; -13.2)	4,254,510 (3,188,429 - 5,488,936)	-36.9 (-49.9; -6.0)
World Bank Upper Middle Income	443,338 (376,972 - 522,669)	-29.0 (-32.4; -26.5)	159,335 (134,282 - 184,578)	-73.8 (-79.6; -55.7)	3,113,531 (2,653,502 - 3,684,058)	-20.1 (-22.7; -18.0)	4,405,952 (3,829,615 - 5,013,783)	-70.4 (-76.6; -52.7)
GBD regions								
High-income Asia Pacific	181,024 (151,954 - 213,838)	40.2 (34.0; 46.7)	17,840 (15,141 - 19,476)	-49.4 (-53.9; -42.1)	1,062,715 (873,335 - 1,285,312)	24.4 (12.6; 30.0)	534,837 (470,756 - 602,453)	-39.9 (-44.8; -32.3)
Central Asia	14,005 (12,074 - 16,147)	-6.8 (-9.7; -3.6)	4,363 (3,913 - 4,936)	15.4 (-6.4; 46.8)	97,489 (84,450 - 111,863)	-7.4 (-9.1; -5.3)	140,160 (125,197 - 163,706)	0.3 (-15.6; 21.2)
East Asia	226,429 (188,326 - 268,949)	-38.6 (-43.3; -34.6)	94,268 (74,041 - 115,717)	-83.6 (-88.1; -69.0)	1,636,763 (1,371,456 - 1,970,140)	-21.4 (-25.9; -18.4)	2,442,771 (1,960,969 - 2,961,307)	-80.9 (-86.1; -65.4)

Southeast Asia	92,529 (78,937 - 108,500)	-14.7 (-16.4; -12.7)	34,984 (26,711 - 47,028)	-34.1 (-53.0; -4.1)	762,971 (656,218 - 888,463)	-9.6 (-10.9; -8.2)	1,181,683 (926,136 - 1,519,820)	-31.1 (-48.1; -3.2)
South Asia	174,712 (148,022 - 208,144)	-18.7 (-20.3; -17.0)	79,723 (48,576 - 110,151)	-42.0 (-56.0; -1.4)	1,068,255 (895,579 - 1,292,807)	-9.8 (-11.5; -8.2)	2,632,912 (1,702,982 - 3,567,125)	-36.6 (-51.2; 5.5)
Southeast Asia, East Asia, and Oceania	321,018 (270,969 - 378,053)	-32.5 (-36.8; -28.9)	130,064 (106,080 - 156,483)	-79.0 (-84.7; -61.9)	2,415,642 (2,052,001 - 2,857,368)	-17.6 (-21.1; -15.2)	3,660,182 (3,083,398 - 4,334,717)	-74.8 (-81.2; -56.6)
Oceania	2,060 (1,806 - 2,342)	-10.6 (-13.9; -6.8)	811 (470 - 1,271)	-22.2 (-38.4; 0.6)	15,908 (13,951 - 18,050)	-6.4 (-9.1; -3.6)	35,727 (21,097 - 54,038)	-17.4 (-34.3; 5.0)
Australasia	4,723 (4,045 - 5,500)	-16.7 (-21.7; -11.6)	1,340 (1,185 - 1,475)	-40.6 (-46.1; -33.7)	31,699 (27,370 - 36,811)	-10.7 (-15.0; -7.1)	32,279 (29,426 - 35,579)	-44.7 (-49.4; -37.9)
Central Europe, Eastern Europe, and Central Asia	116,548 (99,195 - 137,375)	-10.9 (-12.4; -9.5)	35,586 (31,744 - 38,942)	-4.3 (-17.5; 12.3)	784,397 (668,127 - 930,683)	-11.2 (-12.4; -9.9)	1,006,267 (909,492 - 1,111,849)	-11.2 (-21.5; 1.0)
Central Europe	27,175 (23,492 - 31,433)	-21.7 (-23.6; -19.6)	10,591 (9,190 - 11,966)	-26.3 (-37.6; -13.1)	204,641 (176,376 - 238,289)	-17.3 (-18.9; -15.2)	268,135 (234,984 - 300,965)	-33.7 (-42.9; -23.0)
Eastern Europe	75,368 (62,845 - 90,599)	-4.9 (-6.9; -3.0)	20,632 (17,674 - 23,381)	8.3 (-9.5; 30.0)	482,267 (404,522 - 580,911)	-6.2 (-7.6; -4.6)	597,972 (525,846 - 678,797)	3.2 (-10.3; 19.1)
Western Europe	85,014 (72,981 - 98,808)	-11.6 (-14.1; -9.0)	24,929 (22,269 - 26,757)	-34.0 (-38.8; -29.1)	660,613 (568,754 - 765,436)	-9.8 (-11.6; -8.2)	563,894 (521,270 - 611,089)	-39.9 (-43.7; -33.8)
Central Latin America	36,195 (31,406 - 42,150)	-3.6 (-6.5; -0.7)	11,178 (9,309 - 13,225)	-2.9 (-25.4; 19.0)	257,314 (221,236 - 300,473)	-6.4 (-8.2; -4.2)	355,278 (301,008 - 420,491)	-12.7 (-29.1; 5.6)
Tropical Latin America	42,758 (35,905 - 51,520)	-21.9 (-24.8; -18.9)	13,587 (12,358 - 14,473)	-30.2 (-36.3; -23.4)	314,654 (262,549 - 378,996)	-21.1 (-23.9; -18.3)	450,717 (422,094 - 480,905)	-39.0 (-43.6; -31.7)
Southern Latin America	12,238 (10,739 - 13,924)	-30.1 (-34.3; -25.8)	3,841 (3,502 - 4,171)	-44.0 (-52.3; -33.8)	65,153 (56,581 - 74,589)	-21.8 (-25.5; -16.9)	113,957 (105,656 - 122,842)	-47.7 (-54.3; -39.5)
Andean Latin America	9,751 (8,588 - 11,140)	-18.5 (-22.0; -14.6)	3,008 (2,402 - 3,758)	-41.6 (-59.3; -19.1)	80,209 (70,521 - 90,709)	-15.8 (-18.5; -12.4)	104,911 (84,531 - 128,821)	-42.6 (-58.4; -23.2)
Latin America and Caribbean	95,025 (81,542 - 111,625)	-14.1 (-16.2; -11.9)	30,478 (27,236 - 33,714)	-23.2 (-34.3; -12.2)	700,389 (599,465 - 821,301)	-14.5 (-16.4; -12.6)	1,002,564 (911,044 - 1,107,432)	-30.6 (-38.3; -21.8)

High-income North America	80,798 (66,837 - 97,503)	-10.9 (-13.1; -8.8)	22,664 (20,086 - 24,081)	-7.6 (-15.9; -2.6)	909,123 (760,977 - 1,078,890)	-0.2 (-3.1; 3.0)	623,135 (572,971 - 671,138)	-18.5 (-21.6; -15.7)
North Africa and Middle East	64,053 (54,559 - 75,578)	-26.4 (-29.2; -23.3)	13,570 (11,176 - 16,789)	-59.0 (-70.7; -31.6)	376,883 (320,184 - 444,637)	-18.1 (-26.3; -14.1)	492,385 (408,280 - 617,218)	-62.8 (-71.6; -41.0)
Sub-Saharan Africa	49,473 (41,036 - 59,647)	-11.4 (-12.7; -10.0)	13,095 (7,158 - 27,487)	-24.7 (-38.1; -1.5)	321,670 (272,601 - 380,981)	-6.3 (-7.5; -5.2)	519,003 (315,761 - 1,049,048)	-24.5 (-37.9; 1.6)
Central Sub-Saharan Africa	6,684 (5,490 - 8,035)	-5.1 (-9.2; -1.4)	1,834 (837 - 3,949)	-10.8 (-38.1; 25.5)	40,725 (34,895 - 47,850)	-2.6 (-5.7; 0.7)	68,073 (34,663 - 141,435)	-11.4 (-36.7; 22.1)
Southern Sub-Saharan Africa	4,467 (3,705 - 5,402)	-3.6 (-5.7; -1.4)	1,114 (938 - 1,534)	-14.8 (-25.3; -3.1)	28,309 (23,780 - 33,728)	-2.0 (-3.5; -0.5)	40,041 (33,628 - 53,481)	-15.5 (-26.1; -4.0)
Eastern Sub-Saharan Africa	19,836 (16,417 - 23,820)	-15.7 (-17.5; -14.1)	6,338 (2,897 - 14,779)	-26.8 (-42.1; 12.9)	121,152 (102,883 - 143,860)	-9.9 (-11.6; -8.2)	231,757 (118,892 - 530,857)	-27.1 (-42.0; 11.3)
Western Sub-Saharan Africa	18,486 (15,290 - 22,293)	-11.6 (-13.2; -10.1)	3,810 (2,284 - 7,778)	-32.1 (-46.4; -11.6)	131,483 (111,275 - 155,350)	-5.4 (-6.8; -4.0)	179,132 (113,491 - 340,702)	-29.1 (-43.1; -5.8)
Countries in alphabetical order								
Afghanistan	2,914 (2,471 - 3,422)	-19.2 (-24.0; -12.1)	1,183 (363 - 2,053)	-42.7 (-58.3; -11.8)	16,373 (13,652 - 19,482)	-10.6 (-14.9; -6.4)	41,364 (13,929 - 70,872)	-43.8 (-59.4; -14.3)
Albania	594 (498 - 711)	-6.7 (-12.2; -0.5)	173 (107 - 249)	-22.5 (-55.1; 13.5)	4,855 (4,188 - 5,656)	-2.3 (-6.7; 2.0)	4,208 (3,035 - 5,710)	-21.7 (-49.1; 6.7)
Algeria	4,722 (3,970 - 5,563)	-33.0 (-38.1; -27.6)	885 (658 - 1,175)	-64.6 (-75.9; -42.9)	27,321 (21,829 - 32,379)	-24.2 (-43.0; -17.1)	28,872 (22,118 - 38,126)	-64.6 (-74.6; -44.4)
American Samoa	8 (7 - 9)	-4.8 (-10.4; 0.7)	3 (2 - 3)	-21.2 (-40.2; 2.6)	74 (65 - 83)	-2.8 (-6.9; 1.3)	96 (77 - 120)	-17.1 (-36.7; 7.7)
Andorra	18 (15 - 21)	-13.1 (-18.7; -6.1)	4 (3 - 5)	-21.9 (-47.9; 11.6)	148 (129 - 171)	-9.1 (-13.2; -4.8)	102 (76 - 133)	-28.0 (-48.9; -2.8)
Angola	1,359 (1,131 - 1,629)	-18.2 (-22.8; -13.1)	333 (184 - 673)	-28.6 (-51.0; 22.3)	8,585 (7,288 - 10,139)	-10.9 (-15.3; -6.5)	13,203 (7,899 - 25,921)	-27.6 (-50.0; 20.6)
Antigua and Barbuda	12 (10 - 14)	-15.2 (-20.8; -8.5)	5 (4 - 6)	-25.6 (-41.5; -5.1)	97 (84 - 111)	-9.9 (-13.4; -6.3)	143 (119 - 172)	-28.7 (-43.6; -9.7)
Argentina	7,982 (6,988 - 9,116)	-36.1 (-41.2; -31.0)	2,494 (2,266 - 2,769)	-50.1 (-59.0; -37.0)	43,087 (37,424 - 49,222)	-25.8 (-30.4; -19.5)	76,636 (70,465 - 83,617)	-52.4 (-59.5; -41.5)
Armenia	568 (486 - 662)	-21.5 (-28.7; -13.9)	161 (132 - 192)	-27.9 (-50.0; 4.1)	4,364 (3,779 - 5,036)	-12.6 (-16.5; -8.0)	4,646 (3,880 - 5,488)	-25.8 (-44.3; -1.4)

Australia	3,655 (3,102 - 4,283)	-17.5 (-24.0; -11.0)	1,071 (937 - 1,193)	-40.5 (-46.8; -33.0)	25,036 (21,606 - 28,912)	-10.7 (-15.7; -6.1)	25,407 (22,961 - 28,191)	-44.8 (-50.2; -37.7)
Austria	2,676 (2,252 - 3,201)	-22.9 (-31.1; -16.1)	459 (399 - 511)	-30.0 (-38.7; -22.1)	20,365 (17,199 - 24,239)	-4.6 (-14.9; 8.7)	11,484 (10,226 - 12,808)	-35.1 (-41.1; -28.4)
Azerbaijan	1,564 (1,236 - 2,026)	0.3 (-6.8; 6.8)	163 (111 - 259)	-12.5 (-44.0; 27.9)	11,659 (9,892 - 13,621)	-2.0 (-6.1; 2.5)	7,067 (5,407 - 10,011)	-16.1 (-37.1; 7.4)
Bahamas	42 (36 - 50)	-6.7 (-12.4; 0.1)	16 (12 - 19)	-15.2 (-36.4; 12.3)	339 (295 - 392)	-4.0 (-8.1; 0.2)	550 (433 - 684)	-14.2 (-35.1; 13.0)
Bahrain	153 (121 - 196)	-11.1 (-19.6; 0.8)	20 (15 - 31)	-52.9 (-69.4; -32.1)	1,059 (873 - 1,262)	-6.4 (-11.9; -1.2)	880 (677 - 1,193)	-58.1 (-70.1; -41.3)
Bangladesh	22,714 (19,498 - 26,471)	-0.8 (-6.9; 5.0)	15,001 (7,897 - 23,811)	-35.1 (-56.7; 30.9)	137,533 (118,858 - 157,160)	3.7 (-0.8; 8.4)	469,173 (262,168 - 720,367)	-33.2 (-55.8; 32.2)
Barbados	48 (41 - 56)	-7.8 (-13.9; -1.6)	21 (17 - 25)	-25.8 (-44.7; -2.0)	377 (328 - 431)	-5.6 (-9.4; -1.9)	568 (455 - 697)	-27.4 (-44.4; -6.2)
Belarus	3,348 (2,845 - 3,903)	-9.2 (-16.4; -1.8)	772 (509 - 1,125)	-8.5 (-34.1; 31.8)	22,746 (19,545 - 26,471)	-7.7 (-13.1; -2.5)	24,633 (17,797 - 33,226)	-13.9 (-34.4; 15.9)
Belgium	2,277 (1,942 - 2,675)	-4.8 (-10.6; 1.4)	647 (555 - 728)	-11.2 (-28.9; 3.1)	17,753 (15,373 - 20,527)	-5.3 (-10.0; -1.6)	14,790 (13,284 - 16,302)	-24.4 (-34.0; -15.3)
Belize	32 (27 - 37)	-12.1 (-18.1; -6.1)	10 (8 - 12)	-21.4 (-37.9; -0.4)	267 (231 - 305)	-6.7 (-10.9; -2.7)	362 (309 - 422)	-21.7 (-37.0; -1.4)
Benin	523 (434 - 628)	-9.3 (-14.6; -4.2)	121 (63 - 249)	-29.3 (-48.7; -0.8)	3,705 (3,129 - 4,332)	-4.7 (-8.8; -0.2)	5,527 (3,109 - 10,583)	-26.5 (-47.1; 6.1)
Bermuda	12 (10 - 14)	-8.8 (-16.7; 1.1)	3 (3 - 4)	-41.5 (-56.8; -21.2)	98 (84 - 113)	-4.6 (-9.2; -0.2)	83 (68 - 102)	-42.6 (-56.2; -25.0)
Bhutan	69 (59 - 81)	-30.8 (-34.9; -25.8)	28 (17 - 42)	-51.2 (-68.0; 2.1)	469 (399 - 542)	-19.1 (-23.4; -14.3)	858 (549 - 1,289)	-49.3 (-67.1; 3.3)
Bolivia (Plurinational State of)	2,000 (1,750 - 2,295)	-22.0 (-27.5; -15.7)	906 (599 - 1,276)	-35.4 (-56.3; 0.0)	14,929 (13,136 - 16,826)	-18.6 (-22.7; -13.5)	29,549 (19,807 - 41,318)	-39.6 (-58.7; -10.2)
Bosnia and Herzegovina	937 (797 - 1,083)	-16.4 (-22.6; -9.6)	364 (269 - 466)	-38.5 (-60.1; 2.0)	6,894 (6,015 - 7,917)	-11.3 (-15.1; -6.9)	8,510 (6,713 - 10,693)	-42.0 (-59.8; -12.9)
Botswana	112 (92 - 137)	-15.2 (-21.1; -9.2)	27 (14 - 52)	-37.8 (-65.0; -0.5)	753 (630 - 893)	-6.9 (-11.0; -3.0)	981 (537 - 1,866)	-34.7 (-62.3; 5.4)

Brazil	41,643 (34,891 - 50,229)	-22.2 (-25.1; -19.3)	13,260 (12,016 - 14,156)	-30.3 (-36.2; -23.6)	306,335 (255,287 - 369,755)	-21.3 (-24.3; -18.6)	439,451 (411,002 - 468,439)	-39.2 (-43.8; -31.8)
Brunei Darussalam	139 (118 - 160)	-30.9 (-37.1; -24.1)	23 (19 - 28)	-52.2 (-62.7; -34.6)	822 (712 - 930)	-19.7 (-26.6; -5.1)	836 (712 - 1,001)	-52.4 (-61.6; -37.6)
Bulgaria	1,986 (1,707 - 2,290)	2.6 (-4.7; 10.5)	827 (647 - 1,026)	17.4 (-11.3; 48.5)	13,658 (11,820 - 15,760)	-1.0 (-5.2; 3.5)	20,827 (16,501 - 26,038)	2.3 (-19.5; 27.3)
Burkina Faso	864 (705 - 1,058)	-5.1 (-10.6; 0.5)	208 (114 - 435)	-17.2 (-38.4; 22.5)	6,086 (5,134 - 7,216)	-2.6 (-6.9; 1.8)	10,204 (5,427 - 19,829)	-14.8 (-37.1; 24.5)
Burundi	564 (463 - 694)	-15.4 (-21.4; -9.6)	197 (75 - 540)	-27.1 (-56.1; 15.9)	3,351 (2,843 - 3,931)	-11.3 (-15.4; -6.3)	7,281 (3,051 - 19,217)	-28.9 (-57.0; 13.4)
CÃ´te d'Ivoire	1,103 (908 - 1,348)	-5.1 (-10.7; 1.0)	221 (118 - 446)	-24.5 (-46.8; 5.5)	7,629 (6,492 - 9,044)	-2.6 (-6.5; 1.5)	10,970 (6,226 - 20,992)	-22.6 (-47.5; 9.2)
Cabo Verde	38 (31 - 48)	-6.6 (-12.8; -0.7)	5 (2 - 10)	-20.0 (-48.2; 32.3)	286 (244 - 333)	-4.5 (-8.7; -0.1)	186 (114 - 356)	-29.1 (-50.8; 8.0)
Cambodia	1,762 (1,522 - 2,074)	-23.9 (-28.4; -18.9)	643 (449 - 951)	-44.9 (-63.9; -11.7)	14,149 (12,120 - 16,347)	-15.4 (-19.7; -10.8)	21,848 (16,049 - 30,786)	-44.5 (-61.6; -11.8)
Cameroon	1,193 (985 - 1,456)	-5.9 (-11.3; 0.0)	262 (142 - 567)	-24.9 (-48.5; 18.7)	8,359 (7,079 - 9,801)	-2.4 (-6.2; 2.1)	12,373 (7,261 - 26,105)	-19.5 (-45.7; 25.2)
Canada	7,070 (5,985 - 8,383)	-18.6 (-25.3; -10.6)	1,882 (1,620 - 2,126)	-34.0 (-42.0; -25.6)	59,273 (51,642 - 68,137)	-14.9 (-18.7; -10.3)	46,988 (42,240 - 52,309)	-40.8 (-46.6; -33.5)
Caribbean	6,321 (5,473 - 7,304)	-4.3 (-7.4; -0.8)	2,706 (2,039 - 3,604)	-17.9 (-33.5; 2.5)	48,211 (42,089 - 54,920)	-4.7 (-6.7; -2.9)	91,658 (67,059 - 123,044)	-17.1 (-32.3; 1.6)
Central African Republic	285 (236 - 344)	0.8 (-6.3; 6.7)	110 (38 - 244)	2.0 (-33.0; 42.1)	1,601 (1,358 - 1,891)	1.9 (-2.3; 6.4)	4,080 (1,534 - 8,884)	0.4 (-31.9; 40.6)
Chad	608 (507 - 734)	-6.2 (-11.3; 0.2)	167 (84 - 336)	-17.5 (-37.9; 14.2)	4,151 (3,533 - 4,886)	-4.3 (-8.3; -0.3)	8,287 (4,081 - 15,636)	-16.9 (-39.4; 13.2)
Chile	3,359 (2,877 - 3,873)	-10.2 (-16.8; -2.4)	1,001 (870 - 1,121)	-17.6 (-33.5; -4.7)	18,054 (15,636 - 20,703)	-9.8 (-13.8; -5.0)	28,635 (25,788 - 31,612)	-27.0 (-36.9; -17.1)
China	218,520 (181,673 - 259,677)	-39.3 (-44.0; -35.2)	90,736 (70,370 - 112,972)	-84.1 (-88.7; -69.4)	1,581,082 (1,323,102 - 1,909,002)	-21.9 (-26.4; -18.8)	2,344,722 (1,850,358 - 2,869,725)	-81.5 (-86.6; -66.1)
Colombia	9,520 (8,376 - 10,836)	-11.0 (-18.1; -3.7)	3,258 (2,428 - 4,276)	-7.0 (-34.9; 24.2)	63,352 (54,503 - 72,514)	-12.0 (-16.5; -6.9)	93,654 (71,595 - 122,026)	-18.7 (-39.9; 6.9)

Comoros	57 (47 - 70)	-19.3 (-24.2; -14.2)	16 (7 - 34)	-28.5 (-53.5; 37.9)	350 (301 - 406)	-12.8 (-17.2; -7.5)	523 (243 - 1,063)	-27.5 (-53.2; 52.3)
Congo	290 (240 - 351)	-14.4 (-19.3; -8.8)	72 (39 - 151)	-30.0 (-51.8; 6.5)	1,781 (1,524 - 2,082)	-10.3 (-14.3; -6.3)	2,694 (1,541 - 5,595)	-29.2 (-52.1; 10.0)
Cook Islands	4 (3 - 5)	7.2 (-11.6; 21.0)	1 (0 - 1)	-54.0 (-68.8; -32.0)	34 (30 - 39)	5.2 (0.8; 9.9)	25 (19 - 35)	-48.1 (-63.9; -24.9)
Costa Rica	729 (626 - 847)	-8.2 (-13.7; -1.6)	176 (128 - 229)	-27.8 (-46.0; -5.8)	5,664 (4,960 - 6,450)	-5.8 (-9.6; -1.8)	5,389 (4,119 - 6,859)	-28.9 (-45.4; -7.8)
Croatia	1,055 (925 - 1,210)	-21.4 (-27.9; -14.1)	367 (290 - 461)	-31.4 (-46.4; -13.0)	8,244 (7,165 - 9,443)	-17.2 (-21.2; -12.6)	9,442 (7,556 - 11,664)	-38.3 (-50.7; -22.5)
Cuba	1,706 (1,456 - 2,013)	-14.5 (-20.8; -8.0)	515 (414 - 637)	-31.2 (-47.9; -8.2)	14,494 (12,610 - 16,528)	-10.4 (-14.3; -6.7)	14,654 (11,735 - 18,162)	-37.0 (-50.8; -16.7)
Cyprus	265 (224 - 318)	-29.6 (-35.4; -23.8)	56 (47 - 65)	-59.7 (-69.7; -41.3)	2,109 (1,816 - 2,462)	-20.1 (-24.5; -14.5)	1,436 (1,245 - 1,621)	-58.2 (-67.6; -44.0)
Czechia	2,542 (2,175 - 2,993)	-25.4 (-32.9; -18.0)	648 (524 - 809)	-47.4 (-58.1; -32.6)	21,073 (18,208 - 24,376)	-17.2 (-21.3; -13.0)	17,274 (14,204 - 21,608)	-50.4 (-59.1; -36.6)
Democratic People's Republic of Korea	4,438 (3,797 - 5,166)	-19.3 (-24.7; -14.0)	2,426 (1,548 - 3,795)	-37.0 (-56.3; -10.8)	28,258 (24,317 - 32,513)	-12.0 (-16.1; -7.3)	65,194 (42,745 - 99,134)	-33.7 (-53.2; -8.1)
Democratic Republic of the Congo	4,594 (3,750 - 5,554)	-0.4 (-6.3; 5.2)	1,290 (520 - 3,021)	-3.7 (-37.8; 43.9)	27,754 (23,812 - 32,611)	0.5 (-4.0; 4.9)	46,985 (21,269 - 103,094)	-4.4 (-35.9; 41.5)
Denmark	1,108 (953 - 1,291)	-26.2 (-32.6; -18.6)	367 (319 - 410)	-41.6 (-49.8; -32.2)	8,391 (7,274 - 9,750)	-21.7 (-25.8; -16.5)	8,517 (7,619 - 9,459)	-49.1 (-55.4; -41.0)
Djibouti	75 (62 - 92)	-17.8 (-23.0; -12.3)	20 (9 - 39)	-25.5 (-53.0; 20.9)	490 (417 - 571)	-11.4 (-15.6; -6.7)	778 (369 - 1,500)	-24.3 (-52.0; 21.4)
Dominica	9 (8 - 11)	-8.7 (-14.6; -3.0)	4 (3 - 5)	-19.9 (-38.7; 5.5)	69 (60 - 80)	-8.5 (-12.5; -4.3)	104 (81 - 132)	-20.2 (-39.1; 4.0)
Dominican Republic	1,230 (1,071 - 1,403)	11.3 (3.8; 19.5)	481 (340 - 661)	22.0 (-21.1; 79.5)	9,533 (8,341 - 10,864)	5.3 (0.6; 10.3)	16,632 (11,820 - 23,072)	15.5 (-23.4; 68.7)
Ecuador	3,148 (2,787 - 3,581)	-4.6 (-12.0; 2.2)	1,043 (797 - 1,324)	-25.2 (-48.8; 8.4)	23,930 (21,215 - 26,963)	-9.8 (-13.6; -5.6)	35,234 (27,712 - 44,387)	-30.7 (-49.7; -4.0)
Egypt	9,227 (7,905 - 10,872)	-31.6 (-36.3; -26.4)	2,035 (1,418 - 2,867)	-71.4 (-81.7; -44.7)	54,368 (45,386 - 64,247)	-21.5 (-33.0; -16.4)	104,942 (74,559 - 145,019)	-77.4 (-85.6; -52.9)

El Salvador	782 (684 - 906)	-21.3 (-26.7; -16.0)	224 (161 - 307)	-55.3 (-71.5; -32.0)	5,715 (4,982 - 6,591)	-15.9 (-19.9; -11.7)	7,211 (5,348 - 9,753)	-59.8 (-73.2; -39.3)
Equatorial Guinea	53 (43 - 65)	-32.3 (-37.3; -25.9)	9 (5 - 16)	-62.7 (-80.9; -8.7)	356 (298 - 418)	-20.1 (-26.7; -14.8)	347 (206 - 626)	-61.5 (-79.4; -10.9)
Eritrea	346 (287 - 415)	-19.5 (-24.5; -14.3)	117 (55 - 235)	-27.0 (-51.1; 25.5)	2,142 (1,824 - 2,519)	-13.1 (-17.4; -8.1)	4,450 (2,246 - 8,830)	-27.8 (-51.0; 22.6)
Estonia	403 (330 - 509)	-23.9 (-33.2; -10.2)	64 (47 - 104)	-45.1 (-60.6; -21.7)	2,821 (2,416 - 3,272)	-23.5 (-32.8; -14.9)	2,075 (1,614 - 2,902)	-49.0 (-60.6; -30.8)
Eswatini	49 (41 - 60)	-9.6 (-15.4; -3.6)	13 (9 - 23)	-22.8 (-47.8; 16.5)	321 (269 - 380)	-2.7 (-6.6; 1.6)	468 (305 - 814)	-21.1 (-47.5; 21.6)
Ethiopia	4,696 (3,899 - 5,662)	-24.9 (-27.4; -22.4)	1,467 (614 - 3,729)	-43.8 (-63.3; 26.5)	28,956 (24,172 - 34,939)	-18.5 (-21.4; -15.6)	50,572 (23,947 - 122,415)	-46.2 (-63.0; 15.9)
Fiji	165 (146 - 190)	-11.4 (-17.8; -5.2)	77 (59 - 99)	-27.5 (-50.1; 1.4)	1,390 (1,222 - 1,580)	-8.1 (-12.6; -3.4)	2,751 (2,118 - 3,578)	-27.0 (-48.6; 2.8)
Finland	1,589 (1,266 - 1,707)	12.4 (-10.6; 26.3)	379 (334 - 426)	-52.8 (-60.6; -34.2)	11,318 (9,782 - 12,985)	10.9 (3.7; 25.9)	9,711 (8,724 - 10,792)	-55.1 (-61.8; -37.8)
France	9,958 (8,612 - 11,622)	1.4 (-5.0; 7.9)	3,576 (3,029 - 4,291)	-19.8 (-30.5; -9.3)	82,057 (71,675 - 94,769)	-0.9 (-5.2; 3.7)	80,026 (70,796 - 92,972)	-20.1 (-29.1; -11.9)
Gabon	103 (84 - 126)	-9.6 (-14.6; -3.9)	21 (13 - 37)	-28.7 (-52.3; 12.6)	649 (553 - 762)	-6.0 (-9.9; -1.8)	765 (500 - 1,318)	-27.2 (-51.4; 13.5)
Gambia	100 (84 - 121)	-3.3 (-8.3; 2.4)	21 (10 - 46)	-9.5 (-39.4; 32.3)	693 (586 - 815)	-1.0 (-5.0; 3.2)	920 (499 - 1,899)	-11.6 (-40.9; 33.0)
Georgia	1,329 (1,167 - 1,507)	10.9 (-0.3; 21.7)	879 (647 - 1,048)	37.4 (7.6; 73.2)	7,930 (6,815 - 9,123)	7.9 (-5.0; 14.8)	19,207 (15,882 - 22,599)	29.9 (4.5; 62.2)
Germany	17,154 (14,665 - 20,198)	-15.2 (-21.6; -7.8)	4,446 (3,904 - 4,980)	-44.9 (-53.4; -32.7)	145,759 (125,493 - 170,597)	-7.7 (-11.6; -3.8)	107,537 (96,053 - 119,240)	-47.2 (-54.2; -36.3)
Ghana	1,656 (1,381 - 2,017)	-1.4 (-7.2; 4.2)	357 (217 - 628)	-22.3 (-42.2; 15.8)	11,570 (9,913 - 13,478)	-0.2 (-4.1; 4.1)	15,325 (9,734 - 27,163)	-19.5 (-41.0; 16.2)
Greece	2,031 (1,744 - 2,385)	5.2 (-1.2; 12.1)	657 (567 - 743)	-7.0 (-20.6; 4.8)	15,956 (13,672 - 18,464)	2.0 (-2.2; 6.4)	14,676 (13,127 - 16,179)	-10.8 (-20.5; -2.1)
Greenland	15 (12 - 17)	-2.4 (-31.9; 14.3)	6 (5 - 8)	-48.9 (-60.5; -35.2)	101 (88 - 115)	-6.1 (-11.6; 0.4)	191 (149 - 233)	-51.4 (-62.6; -37.9)

Grenada	13 (11 - 15)	-16.9 (-22.8; -11.1)	6 (5 - 7)	-36.4 (-51.1; -16.6)	100 (86 - 115)	-11.0 (-15.0; -6.5)	182 (155 - 213)	-36.0 (-49.5; -18.1)
Guam	34 (27 - 42)	10.1 (-10.1; 24.1)	7 (6 - 9)	-45.9 (-57.3; -29.7)	308 (267 - 352)	7.5 (2.3; 12.8)	279 (235 - 347)	-24.9 (-38.3; -7.6)
Guatemala	1,510 (1,305 - 1,749)	-13.4 (-19.3; -7.4)	428 (328 - 553)	2.0 (-31.7; 56.3)	11,360 (9,803 - 13,085)	-11.3 (-15.4; -7.0)	18,105 (14,072 - 23,127)	-3.1 (-33.0; 40.7)
Guinea	574 (478 - 693)	-4.2 (-9.6; 1.5)	159 (79 - 312)	-20.2 (-44.2; 14.2)	3,825 (3,257 - 4,464)	-2.1 (-5.6; 1.7)	7,384 (3,876 - 13,356)	-19.1 (-44.8; 19.5)
Guinea-Bissau	83 (68 - 99)	-9.6 (-14.9; -4.2)	22 (11 - 49)	-22.4 (-50.4; 22.4)	559 (476 - 654)	-5.0 (-8.9; -0.7)	1,026 (519 - 2,195)	-23.6 (-53.2; 23.6)
Guyana	86 (75 - 98)	-17.2 (-22.6; -10.9)	45 (34 - 59)	-30.5 (-50.6; -6.1)	597 (517 - 688)	-9.6 (-14.3; -4.7)	1,644 (1,220 - 2,163)	-30.3 (-50.1; -4.2)
Haiti	1,642 (1,411 - 1,907)	-1.6 (-7.9; 7.6)	1,054 (488 - 1,896)	-30.8 (-52.5; 6.1)	10,097 (8,773 - 11,549)	-2.9 (-6.9; 1.2)	40,529 (19,508 - 70,919)	-28.9 (-50.6; 5.2)
Honduras	946 (821 - 1,103)	-11.1 (-17.1; -5.2)	681 (467 - 931)	16.3 (-17.8; 74.0)	6,877 (5,934 - 8,055)	-9.9 (-14.4; -5.4)	22,390 (15,211 - 30,976)	-3.5 (-31.3; 41.3)
Hungary	1,885 (1,619 - 2,212)	-17.3 (-24.2; -9.9)	643 (525 - 775)	-28.1 (-41.7; -11.1)	15,118 (13,081 - 17,553)	-16.4 (-20.4; -12.2)	17,513 (14,556 - 20,814)	-36.1 (-47.1; -20.1)
Iceland	61 (52 - 73)	-15.3 (-21.2; -8.0)	13 (11 - 15)	-42.0 (-51.2; -31.8)	508 (437 - 587)	-9.4 (-13.3; -4.7)	329 (288 - 374)	-45.2 (-52.4; -37.2)
India	129,266 (108,773 - 154,663)	-20.1 (-21.9; -18.4)	53,701 (32,509 - 75,336)	-44.5 (-59.3; -4.0)	793,638 (657,324 - 966,644)	-11.8 (-13.8; -10.0)	1,763,367 (1,140,343 - 2,424,675)	-39.9 (-55.2; 0.1)
Indonesia	37,592 (31,605 - 45,043)	-11.1 (-12.7; -9.4)	13,240 (7,861 - 19,959)	-23.4 (-47.5; 11.5)	304,178 (253,793 - 365,700)	-8.8 (-10.5; -7.3)	471,554 (300,115 - 671,796)	-26.1 (-46.1; 5.7)
Iran (Islamic Republic of)	7,523 (6,200 - 9,169)	-20.9 (-23.4; -17.9)	1,453 (1,269 - 1,653)	-60.6 (-73.8; -34.9)	45,366 (37,280 - 54,943)	-7.1 (-8.9; -5.2)	43,238 (38,360 - 50,622)	-55.7 (-68.1; -31.7)
Iraq	3,242 (2,740 - 3,831)	-40.3 (-45.7; -34.7)	566 (410 - 796)	-68.4 (-80.4; -40.9)	21,047 (16,904 - 24,891)	-26.1 (-45.7; -19.5)	22,582 (16,870 - 30,599)	-64.1 (-76.6; -41.4)
Ireland	892 (763 - 1,043)	-33.4 (-39.3; -26.8)	247 (216 - 276)	-50.1 (-56.5; -42.8)	7,049 (6,152 - 8,127)	-24.7 (-29.0; -17.8)	6,296 (5,597 - 6,996)	-54.4 (-59.6; -47.1)
Israel	1,205 (1,001 - 1,450)	-2.4 (-8.9; 5.0)	226 (171 - 261)	-29.1 (-44.1; -13.4)	10,076 (8,730 - 11,622)	-2.7 (-9.0; 1.6)	5,678 (4,957 - 6,376)	-38.8 (-47.3; -29.1)

Italy	16,463 (13,815 - 19,563)	6.7 (1.8; 11.8)	3,598 (3,126 - 3,945)	-30.1 (-36.6; -25.6)	100,827 (84,013 - 121,929)	-7.0 (-10.0; -3.6)	75,910 (68,668 - 85,613)	-38.0 (-41.5; -32.7)
Jamaica	405 (353 - 463)	5.1 (-1.5; 13.5)	167 (130 - 208)	-19.8 (-41.1; 5.7)	3,020 (2,633 - 3,417)	1.4 (-2.5; 5.9)	5,152 (4,005 - 6,543)	-30.9 (-49.1; -8.2)
Japan	157,908 (132,653 - 187,295)	77.0 (67.6; 87.4)	14,508 (12,055 - 15,953)	-39.2 (-43.3; -33.6)	901,895 (719,609 - 1,097,844)	48.3 (34.0; 57.4)	429,804 (372,212 - 488,176)	-24.5 (-30.0; -16.7)
Jordan	1,270 (1,034 - 1,570)	-2.3 (-12.5; 7.5)	76 (55 - 94)	-54.9 (-69.5; -30.4)	7,841 (6,569 - 9,273)	-1.1 (-5.6; 8.6)	3,335 (2,693 - 4,084)	-49.8 (-63.8; -27.7)
Kazakhstan	3,319 (2,860 - 3,838)	-12.1 (-18.7; -4.6)	1,249 (1,004 - 1,772)	7.3 (-25.4; 50.5)	22,091 (18,996 - 25,495)	-9.8 (-14.4; -5.1)	39,081 (31,384 - 57,081)	0.4 (-26.7; 35.6)
Kenya	2,518 (2,095 - 3,039)	-11.8 (-12.9; -10.7)	690 (355 - 1,505)	-2.6 (-19.4; 20.5)	15,759 (13,143 - 18,905)	-5.6 (-6.8; -4.3)	24,997 (13,887 - 53,018)	-2.5 (-17.8; 22.2)
Kiribati	24 (21 - 27)	-7.3 (-12.5; -1.0)	13 (10 - 17)	-16.6 (-38.4; 13.4)	180 (159 - 204)	-4.7 (-8.7; -0.3)	591 (449 - 774)	-15.9 (-38.3; 16.1)
Kuwait	556 (438 - 711)	3.1 (-6.4; 15.2)	40 (33 - 51)	-24.8 (-43.0; 3.4)	3,852 (3,238 - 4,565)	2.1 (-3.0; 9.9)	1,807 (1,476 - 2,200)	-34.9 (-48.1; -11.3)
Kyrgyzstan	996 (863 - 1,143)	-15.4 (-21.6; -8.7)	307 (255 - 363)	-11.5 (-30.5; 14.8)	6,889 (5,998 - 7,898)	-13.8 (-18.2; -9.2)	11,534 (9,665 - 13,397)	-15.3 (-32.2; 8.8)
Lao People's Democratic Republic	763 (657 - 889)	-23.6 (-27.7; -18.9)	285 (185 - 523)	-35.2 (-56.5; 12.1)	6,041 (5,242 - 6,976)	-16.6 (-20.4; -12.4)	10,853 (7,306 - 18,161)	-30.8 (-52.0; 15.2)
Latvia	635 (535 - 736)	-22.4 (-29.5; -15.4)	124 (100 - 155)	-37.4 (-52.0; -8.6)	4,494 (3,871 - 5,159)	-15.2 (-19.8; -10.0)	3,956 (3,264 - 4,864)	-36.0 (-48.9; -9.6)
Lebanon	660 (539 - 834)	-15.3 (-24.8; -4.5)	37 (21 - 54)	-72.7 (-86.7; -47.5)	3,628 (3,015 - 4,328)	-9.6 (-18.9; -4.4)	1,416 (991 - 1,898)	-64.3 (-78.7; -41.8)
Lesotho	118 (98 - 144)	-3.0 (-9.3; 3.4)	44 (26 - 75)	-0.1 (-34.9; 52.9)	704 (592 - 829)	0.2 (-4.1; 4.5)	1,370 (813 - 2,273)	-1.1 (-36.8; 53.4)
Liberia	230 (190 - 284)	-6.7 (-11.6; -1.6)	47 (19 - 118)	-28.6 (-56.8; 10.6)	1,630 (1,389 - 1,899)	-3.1 (-6.9; 1.3)	2,071 (995 - 4,677)	-34.1 (-64.4; 5.7)
Libya	747 (617 - 917)	-19.8 (-26.1; -12.7)	107 (73 - 156)	-60.9 (-73.2; -36.3)	4,772 (3,877 - 5,658)	-11.4 (-27.1; -6.2)	4,380 (3,189 - 6,255)	-66.7 (-77.7; -42.9)
Lithuania	900 (758 - 1,058)	-13.3 (-20.0; -6.1)	169 (137 - 207)	-22.6 (-39.5; -3.3)	6,442 (5,524 - 7,477)	-9.5 (-14.0; -4.2)	5,560 (4,591 - 6,705)	-23.8 (-37.7; -7.1)

Luxembourg	108 (89 - 128)	-15.5 (-22.5; -8.7)	21 (18 - 26)	-41.0 (-50.0; -30.5)	881 (768 - 1,016)	-12.0 (-16.6; -7.9)	561 (485 - 656)	-44.5 (-51.4; -36.0)
Madagascar	1,890 (1,573 - 2,289)	-9.6 (-14.9; -3.3)	741 (306 - 1,701)	-13.1 (-39.9; 22.3)	11,256 (9,626 - 13,084)	-6.6 (-10.5; -1.6)	29,739 (13,089 - 68,791)	-13.9 (-41.8; 21.6)
Malawi	831 (683 - 1,017)	-4.4 (-9.7; 1.3)	243 (96 - 549)	-15.5 (-40.8; 15.3)	4,955 (4,191 - 5,800)	-1.7 (-5.7; 2.7)	9,069 (4,090 - 20,006)	-13.1 (-40.3; 21.5)
Malaysia	3,564 (3,032 - 4,201)	-28.2 (-34.7; -19.0)	935 (694 - 1,261)	-56.8 (-71.5; -32.0)	33,295 (28,894 - 38,315)	-16.8 (-21.5; -12.1)	33,124 (25,895 - 43,645)	-53.2 (-66.6; -31.3)
Maldives	47 (39 - 57)	-32.6 (-37.7; -25.6)	10 (7 - 12)	-64.9 (-79.7; -18.2)	464 (399 - 538)	-16.2 (-21.4; -11.7)	342 (272 - 442)	-63.5 (-77.1; -19.2)
Mali	901 (752 - 1,091)	-12.3 (-17.0; -6.4)	241 (125 - 523)	-26.1 (-46.3; 14.6)	6,240 (5,297 - 7,351)	-8.8 (-12.9; -3.9)	11,699 (6,183 - 22,402)	-25.8 (-47.7; 16.1)
Malta	99 (82 - 119)	-11.7 (-17.9; -4.4)	14 (12 - 16)	-44.6 (-53.1; -35.2)	792 (675 - 919)	-8.5 (-12.9; -4.0)	407 (353 - 470)	-41.8 (-49.5; -33.5)
Marshall Islands	11 (10 - 13)	-15.6 (-19.9; -11.3)	5 (3 - 7)	-31.4 (-48.4; -3.2)	85 (75 - 96)	-9.9 (-13.7; -6.0)	199 (116 - 297)	-23.1 (-41.9; 5.9)
Mauritania	199 (163 - 243)	-15.5 (-21.1; -9.8)	31 (14 - 59)	-46.6 (-65.5; -11.6)	1,409 (1,205 - 1,643)	-10.3 (-14.6; -5.7)	1,281 (713 - 2,332)	-45.9 (-65.0; -14.4)
Mauritius	208 (181 - 239)	-15.6 (-21.0; -10.0)	76 (60 - 94)	-25.8 (-43.1; -5.6)	1,933 (1,680 - 2,217)	-8.8 (-12.7; -4.5)	2,667 (2,159 - 3,290)	-25.7 (-41.8; -5.9)
Mexico	16,547 (13,936 - 19,851)	7.7 (5.6; 9.8)	4,517 (3,710 - 5,252)	12.8 (-20.8; 35.8)	119,746 (99,968 - 143,879)	-0.3 (-2.2; 1.3)	146,332 (123,141 - 168,678)	2.7 (-20.3; 21.1)
Micronesia (Federated States of)	18 (16 - 21)	-21.7 (-25.6; -17.2)	9 (4 - 13)	-35.5 (-56.5; -2.5)	143 (125 - 162)	-15.2 (-19.0; -10.9)	331 (176 - 488)	-32.8 (-54.6; -2.0)
Monaco	8 (6 - 9)	-6.6 (-12.5; -0.3)	2 (1 - 2)	-56.7 (-70.7; -34.6)	60 (51 - 70)	-4.2 (-8.5; 0.0)	44 (34 - 56)	-51.1 (-64.6; -29.3)
Mongolia	612 (523 - 708)	-5.8 (-17.8; 7.2)	282 (210 - 385)	17.5 (-24.8; 89.3)	3,796 (3,280 - 4,378)	-7.1 (-11.7; -1.7)	8,548 (6,311 - 12,248)	5.9 (-30.1; 64.5)
Montenegro	114 (96 - 136)	-0.3 (-6.3; 6.2)	25 (20 - 32)	1.0 (-25.2; 33.0)	954 (823 - 1,094)	-2.0 (-6.3; 2.2)	712 (581 - 877)	-6.8 (-24.8; 14.7)
Morocco	4,485 (3,794 - 5,253)	-32.8 (-37.3; -28.1)	1,113 (763 - 1,621)	-56.8 (-71.3; -21.3)	24,641 (20,517 - 29,395)	-22.9 (-34.4; -17.4)	33,155 (23,008 - 48,586)	-58.6 (-72.4; -30.7)

Mozambique	1,498 (1,227 - 1,833)	-8.2 (-12.9; -2.4)	503 (222 - 1,121)	-22.3 (-47.0; 39.4)	8,562 (7,346 - 10,074)	-4.3 (-8.1; 0.3)	17,988 (8,617 - 39,925)	-16.8 (-44.3; 44.0)
Myanmar	5,910 (4,881 - 7,068)	-9.6 (-20.0; -3.2)	3,316 (2,532 - 5,158)	-48.3 (-66.2; -11.1)	47,697 (41,240 - 55,267)	-4.9 (-9.0; -1.2)	116,730 (87,692 - 170,136)	-46.8 (-64.6; -10.5)
Namibia	115 (95 - 139)	-23.4 (-29.0; -17.7)	30 (21 - 51)	-35.9 (-59.3; 2.0)	767 (645 - 898)	-12.8 (-18.1; -7.7)	954 (657 - 1,600)	-36.0 (-58.8; 1.8)
Nauru	1 (1 - 1)	-7.7 (-12.9; -1.9)	1 (0 - 1)	-25.7 (-44.7; 0.4)	10 (8 - 11)	-3.0 (-6.9; 1.3)	29 (20 - 41)	-18.5 (-37.8; 10.1)
Nepal	2,501 (2,134 - 2,905)	-19.8 (-24.5; -14.7)	1,377 (638 - 2,442)	-42.2 (-60.5; -5.9)	16,331 (13,872 - 19,080)	-10.5 (-14.5; -6.2)	40,981 (20,875 - 69,954)	-42.3 (-60.0; -9.2)
Netherlands	2,864 (2,413 - 3,379)	-26.4 (-32.8; -19.5)	882 (767 - 1,069)	-40.3 (-46.9; -30.7)	23,952 (20,775 - 27,661)	-20.0 (-23.7; -15.3)	21,524 (19,195 - 24,807)	-46.0 (-51.6; -37.2)
New Zealand	1,068 (922 - 1,243)	-12.9 (-18.8; -6.2)	269 (236 - 300)	-40.0 (-46.8; -31.8)	6,663 (5,545 - 8,037)	-9.3 (-13.7; -5.1)	6,872 (6,237 - 7,577)	-43.6 (-48.9; -36.3)
Nicaragua	669 (572 - 785)	-15.1 (-21.9; -8.1)	156 (123 - 194)	-27.2 (-47.8; -2.6)	5,446 (4,734 - 6,241)	-10.5 (-14.5; -6.1)	5,321 (4,203 - 6,626)	-34.6 (-52.2; -15.0)
Niger	874 (722 - 1,059)	-7.3 (-12.6; -1.4)	246 (104 - 540)	-21.1 (-46.7; 13.7)	6,087 (5,171 - 7,160)	-4.0 (-7.6; 0.7)	12,590 (5,267 - 25,913)	-25.0 (-53.1; 10.1)
Nigeria	8,038 (6,609 - 9,738)	-17.8 (-20.1; -15.4)	1,384 (785 - 2,808)	-43.3 (-61.2; -13.2)	58,716 (48,294 - 70,843)	-7.9 (-10.1; -6.0)	64,713 (42,343 - 127,277)	-39.0 (-57.3; -7.9)
Niue	0 (0 - 0)	-15.6 (-22.2; -3.8)	0 (0 - 0)	-45.2 (-64.8; -20.0)	3 (2 - 3)	-9.3 (-13.5; -5.2)	4 (3 - 5)	-38.5 (-58.5; -11.5)
North Macedonia	717 (623 - 822)	-4.3 (-11.1; 2.1)	318 (244 - 404)	2.0 (-31.4; 36.4)	5,044 (4,439 - 5,743)	-8.1 (-13.0; -3.6)	8,792 (6,841 - 11,084)	-9.9 (-35.2; 16.5)
Northern Ireland	379 (325 - 442)	-25.7 (-31.6; -19.2)	122 (108 - 137)	-38.7 (-46.5; -29.8)	2,749 (2,382 - 3,161)	-19.8 (-24.1; -14.8)	2,940 (2,622 - 3,236)	-46.5 (-52.6; -38.1)
Northern Mariana Islands	8 (7 - 10)	-11.9 (-17.3; -6.6)	3 (2 - 4)	-41.4 (-56.3; -20.3)	81 (72 - 93)	-7.8 (-11.5; -4.2)	109 (87 - 137)	-36.0 (-52.7; -11.9)
Norway	1,522 (1,271 - 1,832)	-23.7 (-26.1; -21.3)	287 (256 - 309)	-47.8 (-51.9; -38.8)	15,941 (13,191 - 19,337)	-0.1 (-4.7; 4.9)	7,491 (6,712 - 8,311)	-49.7 (-53.9; -40.8)
Oman	366 (301 - 451)	-26.9 (-32.7; -21.1)	58 (37 - 98)	-58.1 (-77.1; -20.2)	2,619 (2,167 - 3,072)	-18.9 (-29.7; -13.9)	2,749 (1,961 - 4,314)	-62.4 (-76.8; -29.6)

Pakistan	20,162 (17,132 - 23,689)	-19.6 (-22.0; -17.3)	9,617 (5,692 - 14,137)	-25.3 (-44.2; 15.0)	120,283 (99,239 - 144,420)	-11.3 (-14.3; -8.8)	358,532 (216,016 - 523,549)	-19.5 (-39.0; 19.5)
Palau	3 (3 - 4)	5.0 (-10.2; 19.3)	1 (1 - 1)	-30.3 (-53.7; 10.0)	31 (26 - 36)	3.4 (-1.4; 8.1)	43 (32 - 55)	-24.4 (-49.4; 13.6)
Palestine	386 (316 - 475)	-9.4 (-16.3; -0.8)	40 (32 - 48)	-49.4 (-66.2; -22.8)	2,509 (2,061 - 2,972)	-4.3 (-8.9; 0.6)	1,516 (1,277 - 1,778)	-46.0 (-61.6; -22.2)
Panama	668 (584 - 761)	-8.0 (-14.9; -1.8)	195 (144 - 254)	-19.0 (-42.4; 11.3)	4,752 (4,126 - 5,423)	-4.1 (-7.7; -0.3)	5,584 (4,299 - 7,188)	-22.2 (-43.3; 4.3)
Papua New Guinea	1,507 (1,316 - 1,709)	-11.6 (-15.9; -6.6)	556 (266 - 954)	-18.0 (-37.9; 17.1)	11,436 (9,998 - 12,963)	-6.7 (-10.6; -2.6)	25,829 (13,196 - 42,197)	-14.5 (-35.9; 19.0)
Paraguay	1,115 (973 - 1,267)	-7.1 (-15.3; 0.3)	326 (237 - 448)	-23.6 (-47.6; 14.2)	8,320 (7,332 - 9,450)	-8.5 (-13.1; -3.7)	11,266 (8,332 - 15,310)	-27.4 (-49.3; 6.5)
Peru	4,603 (3,984 - 5,350)	-24.9 (-29.7; -19.6)	1,059 (740 - 1,516)	-54.9 (-71.5; -30.7)	41,350 (36,139 - 46,954)	-18.2 (-21.8; -13.8)	40,129 (29,128 - 55,194)	-52.4 (-67.8; -30.7)
Philippines	11,191 (9,446 - 13,343)	-7.0 (-8.0; -6.0)	3,490 (2,781 - 4,605)	62.6 (13.3; 125.0)	96,476 (81,611 - 115,505)	-4.9 (-6.0; -3.6)	143,370 (113,577 - 176,768)	75.9 (21.3; 129.0)
Poland	7,255 (5,984 - 8,786)	-32.3 (-34.2; -30.6)	2,244 (1,865 - 2,700)	-42.1 (-52.6; -28.6)	60,620 (50,482 - 73,231)	-26.7 (-29.2; -24.2)	69,982 (58,557 - 81,969)	-43.7 (-52.6; -31.5)
Portugal	2,009 (1,734 - 2,347)	-25.9 (-32.1; -19.4)	838 (723 - 951)	-35.4 (-43.2; -26.2)	15,412 (13,387 - 17,816)	-17.8 (-21.8; -13.5)	17,245 (15,424 - 19,630)	-43.6 (-49.7; -34.5)
Puerto Rico	534 (443 - 649)	-12.7 (-19.5; -4.3)	147 (110 - 188)	-29.8 (-48.3; -5.6)	4,960 (4,289 - 5,726)	-7.0 (-11.2; -2.8)	3,667 (2,857 - 4,630)	-32.4 (-48.6; -11.8)
Qatar	339 (274 - 412)	-18.7 (-25.3; -13.0)	34 (24 - 46)	-51.2 (-72.8; -12.8)	2,422 (1,952 - 2,857)	-17.4 (-38.8; -11.6)	1,689 (1,246 - 2,192)	-54.2 (-72.3; -26.8)
Republic of Korea	20,684 (17,088 - 24,477)	-43.8 (-49.7; -37.8)	3,171 (2,757 - 3,797)	-73.4 (-79.2; -61.0)	145,932 (125,653 - 168,945)	-33.5 (-42.8; -25.0)	98,397 (86,957 - 115,529)	-69.9 (-75.3; -58.8)
Republic of Moldova	1,045 (859 - 1,273)	1.1 (-10.2; 11.6)	140 (118 - 170)	-28.4 (-44.0; -4.9)	6,800 (5,753 - 7,858)	-2.3 (-6.2; 1.9)	5,056 (4,331 - 6,006)	-24.6 (-38.3; -1.4)
Romania	5,737 (4,959 - 6,605)	-13.0 (-18.9; -6.7)	3,431 (2,743 - 4,168)	-0.9 (-26.8; 41.6)	35,748 (31,061 - 41,528)	-9.9 (-14.2; -5.9)	71,858 (59,787 - 87,194)	-11.4 (-33.5; 19.2)
Russian Federation	53,593 (44,508 - 64,362)	-1.0 (-3.7; 1.7)	15,135 (12,871 - 17,507)	23.9 (4.0; 44.8)	339,236 (282,385 - 410,179)	-6.9 (-8.7; -4.8)	435,466 (369,659 - 505,757)	8.3 (-5.8; 24.3)

Rwanda	710 (580 - 859)	-27.5 (-31.8; -22.4)	220 (92 - 519)	-43.9 (-61.9; 4.8)	4,379 (3,723 - 5,138)	-18.2 (-22.5; -12.4)	7,643 (3,575 - 17,682)	-45.2 (-63.3; -0.1)
Saint Kitts and Nevis	7 (6 - 9)	-23.5 (-28.6; -17.7)	3 (3 - 4)	-39.7 (-55.6; -17.9)	57 (50 - 66)	-16.7 (-20.3; -12.7)	107 (74 - 140)	-43.0 (-60.6; -17.4)
Saint Lucia	24 (21 - 28)	-19.6 (-25.4; -11.9)	9 (8 - 11)	-34.6 (-49.6; -15.6)	189 (164 - 219)	-13.4 (-17.5; -8.5)	282 (234 - 338)	-35.4 (-49.6; -17.3)
Saint Vincent and the Grenadines	13 (11 - 15)	4.6 (-2.3; 11.7)	5 (4 - 5)	-38.3 (-54.2; -23.8)	102 (89 - 117)	-0.4 (-4.4; 3.7)	148 (125 - 172)	-41.9 (-56.3; -28.1)
Samoa	32 (28 - 36)	-18.3 (-22.4; -13.6)	13 (9 - 18)	-33.4 (-54.4; -2.4)	266 (235 - 302)	-11.7 (-15.8; -7.1)	461 (313 - 641)	-27.6 (-51.4; 8.6)
San Marino	6 (5 - 7)	-5.8 (-11.3; 0.2)	1 (1 - 2)	-29.3 (-57.3; 6.0)	50 (43 - 58)	-3.6 (-8.2; 0.4)	31 (22 - 43)	-26.8 (-51.0; 6.7)
Sao Tome and Principe	13 (11 - 15)	-6.0 (-11.1; -0.6)	2 (1 - 4)	-23.3 (-48.1; 15.3)	91 (77 - 105)	-4.5 (-8.0; -0.4)	92 (54 - 186)	-25.1 (-49.4; 11.5)
Saudi Arabia	3,299 (2,654 - 4,136)	-5.9 (-13.1; 4.2)	344 (235 - 473)	-57.5 (-76.8; -25.9)	23,492 (19,521 - 27,716)	-3.1 (-9.2; 1.0)	16,999 (12,299 - 22,487)	-52.5 (-71.9; -22.6)
Scotland	1,189 (1,021 - 1,379)	-22.4 (-29.0; -15.6)	437 (386 - 485)	-40.8 (-47.8; -31.1)	10,531 (9,159 - 12,098)	-15.7 (-19.6; -10.8)	10,636 (9,612 - 11,744)	-46.7 (-52.3; -36.2)
Senegal	720 (592 - 872)	-6.6 (-11.8; -1.4)	141 (66 - 303)	-22.4 (-47.4; 12.9)	5,092 (4,320 - 5,949)	-3.0 (-7.1; 1.7)	6,066 (3,382 - 12,332)	-25.9 (-54.8; 10.3)
Serbia	2,695 (2,325 - 3,105)	-24.3 (-29.7; -17.5)	1,199 (933 - 1,507)	-27.8 (-48.1; -2.5)	18,227 (15,700 - 20,998)	-20.8 (-24.7; -15.6)	28,467 (22,604 - 35,487)	-39.9 (-55.2; -21.5)
Seychelles	14 (11 - 17)	5.3 (-13.0; 20.7)	2 (1 - 4)	-52.1 (-66.7; -33.7)	119 (102 - 138)	1.3 (-3.4; 5.9)	92 (54 - 131)	-44.2 (-56.1; -28.3)
Sierra Leone	383 (320 - 459)	-3.9 (-9.7; 2.0)	95 (45 - 214)	-15.0 (-44.2; 23.7)	2,637 (2,241 - 3,096)	-1.4 (-5.7; 2.9)	4,738 (2,316 - 9,924)	-15.1 (-46.1; 28.9)
Singapore	2,293 (1,834 - 2,892)	-12.0 (-25.9; 2.0)	139 (120 - 184)	-57.5 (-62.9; -47.0)	14,066 (11,914 - 16,702)	-10.6 (-16.1; -4.8)	5,800 (4,918 - 7,107)	-51.4 (-56.9; -41.8)
Slovakia	1,162 (983 - 1,380)	-27.3 (-34.0; -19.5)	255 (193 - 345)	-55.4 (-66.5; -37.0)	10,057 (8,697 - 11,639)	-14.8 (-18.7; -10.7)	7,737 (6,080 - 10,230)	-51.7 (-61.8; -35.8)
Slovenia	493 (414 - 594)	-16.3 (-23.6; -6.9)	98 (74 - 126)	-42.6 (-60.9; -19.5)	4,149 (3,591 - 4,781)	-14.9 (-18.8; -10.5)	2,815 (2,264 - 3,498)	-45.7 (-60.6; -28.5)

Solomon Islands	86 (75 - 99)	-13.1 (-17.8; -8.2)	59 (34 - 93)	-17.9 (-40.4; 12.3)	626 (550 - 715)	-7.5 (-11.6; -3.2)	2,221 (1,347 - 3,355)	-19.2 (-41.5; 11.7)
Somalia	1,012 (829 - 1,253)	-7.8 (-12.7; -3.1)	464 (131 - 1,399)	-6.0 (-44.4; 37.5)	5,965 (5,084 - 6,970)	-3.6 (-7.7; 1.5)	17,267 (5,707 - 49,479)	-7.6 (-43.8; 35.7)
South Africa	3,140 (2,562 - 3,860)	-6.5 (-9.0; -4.0)	627 (531 - 711)	-19.0 (-28.0; -8.5)	20,350 (16,887 - 24,504)	-4.9 (-6.6; -3.1)	20,621 (17,698 - 23,425)	-26.8 (-35.2; -17.0)
South Sudan	487 (402 - 597)	-11.3 (-16.9; -5.7)	110 (46 - 245)	-25.1 (-52.2; 13.3)	3,108 (2,671 - 3,634)	-6.2 (-10.3; -2.1)	4,109 (2,076 - 8,912)	-26.0 (-52.3; 11.8)
Spain	7,896 (6,650 - 9,287)	-4.4 (-10.5; 1.5)	2,665 (2,277 - 3,006)	-7.0 (-27.5; 6.6)	64,761 (56,568 - 74,824)	-5.5 (-9.8; -1.5)	54,708 (49,344 - 61,132)	-24.9 (-32.7; -17.7)
Sri Lanka	3,333 (2,826 - 3,947)	-24.4 (-29.2; -19.0)	1,352 (926 - 1,903)	-52.1 (-71.3; -20.6)	28,618 (24,914 - 33,045)	-11.6 (-15.4; -7.1)	35,869 (25,822 - 53,167)	-46.0 (-64.6; -16.6)
Suriname	74 (64 - 85)	-12.2 (-18.1; -6.7)	30 (24 - 38)	-17.8 (-37.9; 7.3)	557 (487 - 638)	-8.8 (-12.4; -4.8)	998 (794 - 1,275)	-20.7 (-38.3; 4.6)
Sweden	1,654 (1,360 - 1,991)	-4.8 (-10.3; 0.7)	534 (472 - 590)	-41.7 (-47.7; -28.9)	12,899 (10,702 - 15,690)	-13.0 (-18.0; -7.9)	11,309 (10,293 - 12,440)	-48.0 (-53.0; -36.0)
Switzerland	1,868 (1,559 - 2,243)	-20.9 (-27.7; -13.4)	397 (335 - 450)	-45.5 (-52.8; -36.2)	15,817 (13,616 - 18,335)	-11.6 (-15.8; -7.2)	9,336 (8,247 - 10,424)	-47.0 (-53.2; -39.2)
Syrian Arab Republic	1,583 (1,311 - 1,935)	-22.3 (-28.4; -15.2)	336 (250 - 438)	-59.5 (-73.3; -35.3)	9,383 (7,415 - 11,295)	-15.5 (-32.3; -9.8)	15,327 (11,694 - 19,102)	-65.7 (-76.0; -47.7)
Taiwan (Province of China)	3,471 (2,914 - 4,195)	-3.0 (-11.7; 7.2)	1,107 (829 - 1,410)	-10.3 (-33.9; 17.7)	27,423 (23,571 - 31,306)	6.2 (0.6; 10.7)	32,855 (25,678 - 41,338)	-9.3 (-29.9; 15.8)
Tajikistan	998 (780 - 1,244)	-0.6 (-6.4; 5.6)	129 (75 - 191)	22.8 (-12.3; 80.4)	7,258 (6,022 - 8,496)	-1.9 (-6.9; 2.5)	5,221 (3,407 - 7,182)	12.8 (-11.8; 46.4)
Thailand	15,536 (13,614 - 17,873)	-21.8 (-28.2; -15.2)	7,044 (5,075 - 9,379)	-43.4 (-63.2; -14.4)	119,847 (105,272 - 136,113)	-11.2 (-15.3; -6.9)	188,007 (139,379 - 246,432)	-35.1 (-55.6; -3.3)
Timor-Leste	136 (117 - 158)	-17.2 (-21.9; -12.1)	47 (28 - 88)	-20.3 (-48.1; 35.0)	1,095 (951 - 1,258)	-11.1 (-14.7; -7.1)	1,709 (1,004 - 2,962)	-14.7 (-42.2; 36.4)
Togo	388 (320 - 473)	-5.2 (-10.5; 1.1)	81 (40 - 185)	-21.0 (-43.7; 10.0)	2,716 (2,321 - 3,173)	-1.7 (-5.9; 2.6)	3,676 (1,979 - 8,112)	-20.5 (-43.3; 10.1)
Tokelau	0 (0 - 0)	-26.4 (-30.8; -21.8)	0 (0 - 0)	-47.9 (-66.8; -16.0)	2 (2 - 2)	-17.1 (-20.7; -13.1)	3 (2 - 4)	-42.6 (-63.0; -7.9)

Tonga	14 (12 - 18)	-10.8 (-19.9; 3.4)	4 (3 - 6)	-28.2 (-47.7; 0.6)	124 (108 - 142)	-6.4 (-11.0; -2.0)	134 (96 - 180)	-22.6 (-42.1; 5.0)
Trinidad and Tobago	202 (174 - 233)	-8.0 (-14.7; -1.2)	86 (63 - 115)	-24.7 (-48.6; 5.7)	1,499 (1,299 - 1,723)	-6.2 (-9.8; -2.5)	2,555 (1,873 - 3,419)	-26.9 (-49.6; 2.2)
Tunisia	1,581 (1,313 - 1,909)	-26.8 (-31.7; -20.9)	273 (186 - 385)	-64.2 (-79.3; -35.6)	8,671 (7,021 - 10,281)	3.7 (-19.0; 15.8)	8,198 (5,771 - 11,166)	-62.6 (-76.7; -38.2)
Turkey	13,719 (11,850 - 15,875)	-18.8 (-24.8; -12.2)	3,120 (2,421 - 3,942)	-39.1 (-63.8; 20.4)	74,005 (62,671 - 86,497)	-19.2 (-23.3; -14.1)	91,192 (72,042 - 114,240)	-39.9 (-61.8; 5.4)
Turkmenistan	1,064 (930 - 1,204)	13.7 (5.3; 23.4)	383 (293 - 510)	36.9 (4.0; 88.7)	6,709 (5,803 - 7,651)	8.2 (3.0; 13.8)	13,848 (10,883 - 18,222)	34.2 (4.1; 80.0)
Tuvalu	2 (2 - 2)	-20.7 (-25.1; -16.4)	1 (1 - 2)	-45.1 (-64.4; -10.2)	16 (14 - 18)	-14.5 (-18.2; -10.6)	37 (25 - 55)	-40.7 (-60.4; -5.8)
Uganda	1,606 (1,333 - 1,950)	-16.7 (-21.4; -11.7)	467 (204 - 996)	-25.4 (-45.9; 17.9)	10,056 (8,512 - 11,813)	-8.7 (-12.7; -4.6)	17,698 (8,728 - 37,149)	-22.6 (-43.4; 20.8)
Ukraine	15,445 (12,830 - 18,582)	-12.1 (-16.5; -7.3)	4,228 (3,135 - 5,032)	-14.3 (-36.3; 27.3)	99,727 (83,086 - 121,251)	-3.2 (-7.9; 1.1)	121,227 (100,761 - 141,949)	-0.7 (-21.2; 31.7)
United Arab Emirates	1,064 (849 - 1,323)	-25.2 (-29.9; -20.7)	117 (53 - 208)	-65.2 (-78.1; -42.5)	8,045 (6,737 - 9,531)	-15.4 (-23.2; -10.8)	6,068 (3,360 - 9,834)	-64.4 (-76.8; -43.5)
United Kingdom	11,208 (9,520 - 13,306)	-26.0 (-28.2; -23.8)	4,591 (4,147 - 4,847)	-35.0 (-38.7; -29.0)	87,157 (74,443 - 103,448)	-23.2 (-25.3; -21.1)	104,252 (97,766 - 110,183)	-43.6 (-46.7; -33.9)
United Republic of Tanzania	2,822 (2,315 - 3,431)	-9.3 (-15.0; -4.2)	783 (327 - 1,682)	-26.0 (-46.6; 16.3)	17,226 (14,701 - 20,281)	-4.3 (-8.3; -0.3)	28,773 (13,980 - 61,342)	-26.1 (-48.0; 14.8)
United States of America	73,712 (60,842 - 88,992)	-9.9 (-12.2; -7.8)	20,776 (18,379 - 22,076)	-4.5 (-13.7; 0.7)	849,734 (706,672 - 1,013,293)	1.4 (-1.8; 4.8)	575,947 (528,817 - 620,844)	-16.0 (-19.0; -13.1)
United States Virgin Islands	18 (15 - 21)	-13.2 (-19.0; -7.3)	7 (6 - 9)	-31.1 (-49.0; -6.7)	125 (108 - 145)	-8.3 (-12.0; -4.3)	193 (153 - 236)	-34.6 (-51.8; -10.5)
Uruguay	896 (780 - 1,017)	-7.8 (-15.8; -0.5)	346 (308 - 384)	-23.3 (-47.2; -6.4)	4,008 (3,494 - 4,586)	-9.4 (-13.8; -0.5)	8,680 (7,881 - 9,636)	-35.0 (-51.6; -22.1)
Uzbekistan	3,555 (2,991 - 4,238)	1.9 (-5.6; 10.2)	810 (669 - 995)	35.3 (-0.7; 113.0)	26,794 (23,280 - 30,939)	-2.1 (-6.8; 7.9)	31,007 (25,903 - 37,268)	13.4 (-13.0; 58.3)
Vanuatu	46 (40 - 53)	-13.1 (-17.2; -8.4)	22 (12 - 36)	-15.4 (-38.3; 18.9)	350 (306 - 396)	-8.2 (-12.0; -4.1)	898 (530 - 1,423)	-7.9 (-33.1; 27.6)

Venezuela (Bolivarian Republic of)	4,824 (4,249 - 5,541)	-12.6 (-19.4; -6.2)	1,543 (1,144 - 2,049)	-17.0 (-41.6; 16.0)	34,403 (29,900 - 39,398)	-10.9 (-14.7; -6.3)	51,292 (37,989 - 68,415)	-23.3 (-44.4; 5.6)
Viet Nam	12,353 (10,519 - 14,541)	-21.4 (-26.4; -16.6)	4,498 (2,793 - 7,416)	-46.4 (-70.4; -6.5)	108,060 (93,912 - 123,997)	-13.6 (-17.6; -9.4)	153,970 (101,124 - 239,740)	-42.1 (-65.4; -2.9)
Yemen	2,667 (2,281 - 3,109)	-26.3 (-31.3; -20.2)	846 (418 - 1,437)	-49.2 (-63.4; -17.8)	15,013 (12,661 - 17,634)	-17.6 (-21.9; -12.6)	29,608 (15,705 - 48,366)	-48.9 (-63.9; -20.0)
Zambia	708 (589 - 864)	-14.4 (-19.6; -9.0)	294 (164 - 607)	-1.4 (-34.3; 59.9)	4,500 (3,790 - 5,294)	-6.6 (-10.9; -2.1)	10,685 (6,180 - 21,825)	1.1 (-32.0; 62.5)
Zimbabwe	932 (806 - 1,074)	20.4 (11.5; 29.0)	372 (250 - 694)	9.3 (-18.3; 46.6)	5,416 (4,607 - 6,283)	17.1 (11.0; 23.0)	15,647 (10,882 - 25,703)	17.9 (-11.8; 59.5)

Supplement Table 6. Ischaemic stroke related DALYs (absolute numbers [in millions with 95% UI] and percentage [with 95% UI]) associated with risk factors and their clusters in 2019, by World Bank country income level, all ages, both sexes

	Word Bank High Income countries		World Bank Low Income countries		World Bank Low Middle Income countries		World Bank Upper Middle-Income countries		Globally	
	Absolute number	Percentage (95% UI)	Absolute number	Percentage (95% UI)	Absolute number	Percentage (95% UI)	Absolute number	Percentage (95% UI)	Absolute number	Percentage (95% UI)
Air pollution and environmental risks										
Ambient $PM_{2.5}$ pollution	11.90 (9.74-14.00)	18.7 (15.8-21.4)	0.81 (0.62-1.03)	8.9 7.0-11.0)	0.21 (0.12-0.33)	8.6 (5.2-13.0)	3.52 (2.78-4.25)	18.9 (15.2-22.3)	7.36 (6.05-8.68)	22.1 (19.0-24.7)
Household air pollution from solid fuels	4.68 (3.07-6.58)	7.4 (4.9-10.4)	0.02 (0.01-0.04)	0.2 (0.1-0.5)	0.78 (0.60-1.00)	31.6 (25.9-37.5)	2.36 (1.57-3.24)	12.7 (8.8-17.4)	1.52 (0.77-2.59)	4.6 (2.4-7.8)
Low ambient temperature	3.81 (2.83-4.97)	6.0 (4.5-7.8)	0.69 (0.51-0.89)	7.6 (5.5-9.8)	0.10 (0.06-0.14)	4.0 (2.6-5.6)	0.49 (0.23-0.74)	2.6 (1.2-4.0)	2.53 (1.86-3.42)	7.6 (5.7-10.2)
High ambient temperature	0.36 (0.06-0.76)	0.6 (0.1-1.2)	0.01 (0.00-0.03)	0.2 (0.0-0.3)	0.02 (0.01-0.06)	0.9 (0.2-2.3)	0.26 (0.02-0.53)	1.4 (0.1-2.9)	0.06 (0.00-0.16)	0.2 (0.0-0.5)
Lead exposure	2.60 (1.47-3.92)	4.1 2.4-6.1)	0.14 (0.04-0.28)	1.5 (0.4-3.1)	0.12 (0.07-0.18)	4.8 (2.8-6.8)	0.95 (0.56-1.41)	5.1 (3.1-7.3)	1.39 (0.78-2.10)	4.2 (2.4-6.2)
Dietary risks										
Diet high in sodium	6.79 (2.07-13.70)	10.7 (3.3-21.0)	0.53 (0.08-1.37)	5.8 (0.9-14.7)	0.19 (0.03-0.48)	7.6 (1.1-19.3)	1.29 (0.17-3.15)	6.9 (0.9-17.1)	4.79 (1.76-8.78)	14.4 (5.4-25.7)
Diet low in fruits	2.88 (0.81-5.31)	4.5 (1.3-8.3)	0.33 (0.09-0.63)	3.6 (0.9-6.8)	0.15 (0.04-0.28)	6.0 (1.7-10.6)	1.12 (0.34-2.02)	6.0 (1.9-10.5)	1.28 (0.32-2.44)	3.8 (0.9-7.2)
Diet low in vegetables	1.03 (0.24-1.87)	1.6 (0.4-2.9)	0.14 (0.02-0.26)	1.5 (0.3-2.8)	0.10 (0.02-0.18)	4.1 (0.8-7.2)	0.52 (0.09-0.96)	2.8 (0.5-5.1)	0.27 (0.08-0.47)	0.8 (0.2-1.4)

Diet low in whole grains	3.26 (0.98-4.76)	5.1 (1.6-7.5)	0.42 (0.12-0.62)	4.7 (1.4-6.7)	0.13 (0.04-0.20)	5.5 (1.6-7.9)	0.96 (0.31-1.43)	5.2 (1.7-7.5)	1.73 (0.48-2.57)	5.2 (1.5-7.6)
Alcohol use	1.39 (0.19-2.56)	2.2 (0.3-4.0)	0.22 (-0.03-0.47)	2.4 (-0.3-5.2)	0.01 (-0.02-0.04)	0.5 (-0.8-1.8)	0.20 (-0.02-0.42)	1.1 (-0.1-2.3)	0.96 (0.25-1.70)	2.9 (0.7-5.1)
Physical activity										
Low physical activity	2.41 (0.43-6.38)	3.8 (0.7-10.2)	0.46 (0.07-1.26)	5.1 (0.8-13.6)	0.07 (0.01-0.20)	2.7 (0.5-8.1)	0.65 (0.12-1.78)	3.5 (0.6-9.7)	1.23 (0.23-3.29)	3.7 (0.7-9.9)
Tobacco smoking										
Smoking	9.95 (8.84-11.10)	15.7 (14.6-16.8)	1.24 (1.10-1.41)	13.6 (12.6-14.8)	0.22 (0.18-0.27)	9.1 (8.1-10.2)	2.49 (2.10-2.89)	13.4 (12.1-14.6)	5.99 (5.19-6.86)	18.0 (16.7-19.4)
Second-hand smoking	1.92 (1.40-2.46)	3.0 (2.2-3.8)	0.14 (0.10-0.17)	1.5 (1.1-1.9)	0.06 (0.04-0.08)	2.3 (1.7-2.9)	0.59 (0.42-0.77)	3.2 (2.3-4.0)	1.13 (0.83-1.46)	3.4 (2.5-4.3)
Physiological factors										
High BMI	11.00 (6.70-16.20)	17.3 (10.5-24.7)	1.60 (1.00-2.31)	17.5 (11.0-25.3)	0.34 (0.19-0.54)	13.9 (8.0-21.2)	3.28 (2.10-4.70)	17.6 (11.6-24.6)	5.73 (3.31-8.64)	17.2 (10.0-25.4)
High fasting plasma glucose	13.90 (7.34-24.90)	21.9 (11.8-40.1)	2.34 (1.15-4.73)	25.6 (12.9-52.1)	0.47 (0.26-0.88)	19.2 (10.3-35.6)	4.60 (2.47-8.30)	24.8 (13.1-45.6)	6.48 (3.42-11.50)	19.5 (10.6-34.6)
High systolic blood pressure	32.50 (25.70-39.10)	51.2 (40.8-60.6)	4.11 (3.12-5.20)	45.0 (34.9-55.7)	1.18 (0.88-1.48)	47.9 (38.9-55.8)	9.99 (7.96-12.00)	53.7 (43.9-61.9)	17.20 (13.50-20.90)	51.8 (40.4-61.5)
High LDL cholesterol	13.70 (7.72-23.40)	21.6 (12.3-37.1)	2.02 (0.87-3.91)	22.1 (9.6-42.5)	0.50 (0.31-0.77)	20.2 (13.0-31.3)	3.84 (2.37-6.23)	20.6 (12.9-33.5)	7.34 (4.07-12.50)	22.1 (12.5-38.4)
Kidney dysfunction	5.88 (4.38-7.48)	9.3 (6.9-11.6)	0.68 (0.38-0.97)	7.5 (4.0-10.5)	0.19 (0.14-0.24)	7.7 (6.1-9.3)	1.93 (1.48-2.42)	10.4 (8.2-12.7)	3.08 (2.32-3.90)	9.3 (7.0-11.5)
Cluster of risk factors										

Air pollution*	16.60 (14.50-18.90)	26.1 (23.6-28.8)	0.83 (0.64-1.05)	9.1 (7.2-11.3)	0.99 (0.82-1.20)	40.2 (36.9-43.6)	5.87 (4.99-6.83)	31.6 (28.6-34.6)	8.88 (7.51-10.30)	26.7 (24.1-29.4)
Tobacco smoke†	11.60 (10.30-12.90)	18.2 (17.0-19.4)	1.36 (1.20-1.53)	14.8 (13.7-16.0)	0.27 (0.23-0.33)	11.2 (9.9-12.3)	3.00 (2.56-3.48)	16.1 (14.7-17.5)	6.93 (6.00-7.87)	20.8 (19.4-22.3)
Dietary risks‡	17.30 (12.00-23.40)	27.2 (19.2-36.3)	2.13 (1.45-2.91)	23.3 (16.2-31.2)	0.62 (0.40-0.90)	25.2 (16.6-35.1)	4.57 (3.06-6.33)	24.6 (16.7-33.3)	9.97 (6.84-13.60)	30.0 (20.8-40.1)
Behavioural risks§	27.20 (22.40-32.60)	42.8 (35.8-50.0)	3.53 (2.84-4.32)	38.7 (31.6-46.2)	0.86 (0.64-1.14)	34.9 (27.1-44.1)	7.23 (5.66-9.03)	38.8 (32.1-46.4)	15.60 (12.70-18.70)	46.8 (39.1-54.4)
Environmental and occupational risks¶	21.40 (18.90-24.00)	33.7 (30.9-36.9)	1.59 (1.30-1.89)	17.4 (14.6-20.7)	1.12 (0.94-1.35)	45.6 (42.3-49.1)	6.98 (5.99-8.14)	37.5 (34.4-40.9)	11.70 (10.10-13.30)	35.1 (32.1-38.6)
Metabolic risks¦	45.10 (38.60-51.60)	71.1 (62.3-79.9)	6.25 (5.05-7.60)	68.4 (57.1-81.6)	1.63 (1.33-1.95)	66.3 (58.3-74.4)	13.60 (11.50-15.50)	73.1 (64.9-81.2)	23.60 (19.80-27.40)	71.0 (61.6-79.9)
Combined risk factors										
All factors	54.40 (49.00-59.70)	85.7 (81.2-90.3)	7.42 (6.39-8.47)	81.2 (74.0-89.3)	2.03 (1.72-2.39)	82.6 (78.9-86.6)	16.10 (14.10-18.00)	86.5 (82.6-90.5)	28.80 (25.50-32.10)	86.7 (82.3-91.0)

Percentages and number of DALYs are not mutually exclusive. The sum of percentages and number of DALYs in the columns exceeds the totals for all risk factors combined because of overlap between various risk factors. 0% represents very low numbers. *Air pollution cluster includes ambient PM2.5 pollution and household air pollution. †Tobacco smoke includes smoking and second-hand smoking. ‡Dietary risks cluster includes diet high in sodium, diet low in fruits, diet low in vegetables, and diet low in whole grains. §Behavioural risks cluster includes smoking (including second-hand smoking), dietary risks (diet high in sodium, diet high in red meat, diet low in fruits, diet low in vegetables, diet low in whole grains, and alcohol use), and low physical activity. ¶Environmental risks cluster includes air pollution cluster, suboptimal ambient temperature and lead exposure. ¦Metabolic risks cluster includes high fasting plasma glucose, high LDL cholesterol, high systolic blood pressure, high body-mass index, and low kidney dysfunction. **Age-standardised total percentage of DALYs due to all risk factors combined.

Supplement Table 7. Intracerebral haemorrhage related DALYs (absolute numbers [in millions with 95% UI] and percentage [with 95% UI]) associated with risk factors and their clusters in 2019, by World Bank country income level, all ages, both sexes

	Word Bank High Income countries		World Bank Low Income countries		World Bank Low Middle Income countries		World Bank Upper Middle-Income countries		Globally	
	Absolute number	Percentage (95% UI)	Absolute number	Percentage (95% UI)	Absolute number	Percentage (95% UI)	Absolute number	Percentage (95% UI)	Absolute number	Percentage (95% UI)
Environmental and occupational risks										
Ambient $PM_{2.5}$ pollution	14.60 (11.70-17.20)	21.3 (17.3-24.6)	0.54 (0.42-0.67)	11.6 (9.3-14.3)	0.49 (0.27-0.77)	8.7 (5.0-13.6)	5.88 (4.46-7.25)	20.4 (15.9-24.9)	7.67 (6.23-9.10)	25.9 (22.2-28.9)
Household air pollution from solid fuels	8.90 (6.20-12.00)	13.0 (9.2-17.5)	0.01 (0.00-0.02)	0.2 (0.1-0.5)	2.01 (1.53-2.55)	35.8 (29.8-41.6)	5.00 (3.44-6.79)	17.4 (12.3-23.2)	1.88 (0.96-3.14)	6.3 (3.4-10.4)
Low ambient temperature	3.93 (2.83-5.07)	5.7 (4.3-7.4)	0.43 (0.32-0.54)	9.2 (7.0-11.6)	0.23 (0.14-0.33)	4.0 (2.5-5.7)	0.67 (0.20-1.11)	2.3 (0.7-3.8)	2.61 (1.94-3.42)	8.8 (6.8-11.4)
High ambient temperature	0.64 (0.04-1.40)	0.9 (0.1-2.0)	0.01 (0.00-0.03)	0.3 (0.1-0.6)	0.07 (0.02-0.21)	1.3 (0.3-3.5)	0.48 (-0.01-1.01)	1.7 (0.0-3.7)	0.07 (0.00-0.20)	0.2 (0.0-0.7)
Lead exposure	3.66 (2.09-5.47)	5.3 (3.0-7.9)	0.08 (0.02-0.18)	1.8 (0.4-3.8)	0.28 (0.14-0.44)	5.0 (2.6-7.5)	1.74 (1.01-2.60)	6.0 (3.5-8.8)	1.56 (0.86-2.37)	5.3 (3.0-7.9)
Dietary factors										
Diet high in sodium	9.59 (3.15-19.10)	14.0 (4.8-27.5)	0.35 (0.05-0.91)	7.5 (1.0-19.1)	0.47 (0.06-1.32)	8.3 (1.0-22.9)	2.70 (0.35-6.55)	9.4 (1.2-22.6)	6.07 (2.65-10.50)	20.5 (9.4-34.8)
Diet low in fruits	6.49 (3.60-10.60)	9.5 (5.1-15.4)	0.32 (0.16-0.56)	6.8 (3.3-12.0)	0.60 (0.32-0.99)	10.8 (5.8-17.2)	3.47 (1.98-5.50)	12.1 (6.8-18.9)	2.09 (1.04-3.80)	7.1 (3.5-12.4)
Diet low in vegetables	2.65 (0.62-4.92)	3.9 (0.9-7.2)	0.11 (0.02-0.25)	2.4 (0.3-5.4)	0.46 (0.14-0.78)	8.1 (2.4-13.9)	1.72 (0.35-3.22)	6.0 (1.3-11.1)	0.36 (0.09-0.67)	1.2 (0.3-2.3)
Diet low in whole grains										
Alcohol use	7.15 (5.04-9.32)	10.4 (7.4-13.5)	0.78 (0.57-0.99)	16.8 (12.3-21.1)	0.41 (0.25-0.58)	7.2 (4.8-9.8)	1.93 (1.31-2.60)	6.7 (4.6-8.9)	4.03 (2.83-5.41)	13.6 (9.9-17.9)
Physical activity										
Low physical activity										
Tobacco Smoking										

Smoking	13.20 (11.70-14.80)	19.2 (17.8-20.8)	0.96 (0.89-1.04)	20.7 (19.5-22.1)	0.60 (0.49-0.74)	10.7 (9.4-11.9)	4.69 (4.05-5.36)	16.3 (14.7-17.8)	6.94 (5.85-8.18)	23.5 (21.4-25.5)
Second-hand smoking	2.73 (1.98-3.53)	4.0 (3.0-5.1)	0.12 (0.09-0.15)	2.5 (1.9-3.2)	0.15 (0.10-0.21)	2.7 (2.0-3.6)	1.16 (0.82-1.55)	4.0 (2.9-5.2)	1.29 (0.95-1.69)	4.4 (3.3-5.5)
Physiological factors										
High BMI	19.90 (12.60-27.70)	29.0 (18.4-39.8)	1.59 (1.11-2.11)	34.4 (24.1-45.3)	1.39 (0.77-2.13)	24.8 (14.7-36.0)	8.58 (5.49-11.80)	29.9 (19.4-40.2)	8.30 (4.92-12.10)	28.1 (16.9-40.0)
High fasting plasma glucose	13.10 (8.23-18.80)	19.0 (12.1-27.2)	1.13 (0.72-1.62)	24.5 (15.4-35.4)	0.83 (0.50-1.24)	14.8 (9.2-21.7)	5.98 (3.60-8.78)	20.8 (12.5-29.7)	5.10 (3.28-7.35)	17.3 (11.2-24.8)
High systolic blood pressure	40.70 (33.80-47.80)	59.3 (50.0-67.6)	2.51 (2.03-2.99)	54.3 (43.9-63.7)	3.13 (2.38-3.91)	55.7 (46.2-64.2)	17.60 (14.40-20.70)	61.1 (51.2-69.5)	17.40 (13.80-20.80)	59.1 (49.3-68.3)
High LDL cholesterol										
Kidney dysfunction	6.07 (4.91-7.35)	8.8 (7.3-10.6)	0.38 (0.30-0.48)	8.3 (6.5-10.1)	0.37 (0.28-0.48)	6.7 (5.5-8.0)	2.77 (2.23-3.42)	9.6 (8.0-11.6)	2.54 (2.01-3.11)	8.6 (7.1-10.2)
Clusters										
Air pollution*	23.50 (20.70-26.30)	34.2 (31.4-37.1)	0.55 (0.43-0.68)	11.8 (9.4-14.5)	2.50 (2.05-3.00)	44.6 (41.5-47.9)	10.90 (9.46-12.50)	37.8 (34.5-41.0)	9.54 (8.05-11.10)	32.3 (29.2-35.2)
Tobacco smoke†	15.40 (13.70-17.20)	22.5 (20.8-24.2)	1.05 (0.97-1.13)	22.6 (21.2-24.0)	0.73 (0.60-0.90)	13.1 (11.6-14.6)	5.69 (4.91-6.46)	19.8 (18.0-21.5)	7.97 (6.75-9.30)	26.9 (24.8-29.0)
Dietary risks‡	22.80 (15.80-30.40)	33.3 (23.6-43.9)	1.28 (0.86-1.75)	27.7 (18.8-37.6)	1.65 (1.04-2.40)	29.5 (19.4-41.0)	9.03 (6.10-12.40)	31.4 (21.4-42.2)	10.80 (7.38-14.60)	36.7 (26.2-47.8)
Behavioural risks§	35.50 (30.00-41.60)	51.7 (44.7-59.5)	2.41 (2.09-2.76)	52.0 (45.7-58.7)	2.37 (1.75-3.12)	42.2 (34.1-51.7)	13.60 (11.00-16.50)	47.4 (39.7-55.7)	17.00 (14.10-20.50)	57.6 (50.4-65.3)
Environmental and occupational risks¶	28.60 (25.30-31.80)	41.7 (38.7-45.0)	1.00 (0.84-1.19)	21.7 (18.4-25.4)	2.80 (2.30-3.33)	50.0 (46.7-53.4)	12.50 (10.90-14.40)	43.6 (40.2-47.4)	12.30 (10.50-14.20)	41.5 (38.1-44.9)

Metabolic risks¦	49.00 (43.00-55.20)	71.4 (63.7-78.0)	3.28 (2.85-3.67)	70.8 (62.5-77.9)	3.69 (2.93-4.48)	65.8 (57.6-72.9)	21.10 (18.10-23.90)	73.4 (65.7-79.5)	20.90 (17.50-24.30)	70.7 (62.7-77.9)
Combined risk factors										
All factors	60.80 (56.10-65.70)	88.7 (85.8-91.0)	4.01 (3.68-4.31)	86.8 (82.6-90.4)	4.75 (3.98-5.64)	84.8 (81.6-87.4)	25.60 (22.90-28.10)	88.9 (86.0-91.1)	26.40 (23.40-29.80)	89.5 (86.3-92.1)

Percentages and number of DALYs are not mutually exclusive. The sum of percentages and number of DALYs in the columns exceeds the totals for all risk factors combined because of overlap between various risk factors. 0% represents very low numbers. *Air pollution cluster includes ambient PM2.5 pollution and household air pollution. †Tobacco smoke includes smoking and second-hand smoking. ‡Dietary risks cluster includes diet high in sodium, diet low in fruits, diet low in vegetables, and diet low in whole grains. §Behavioural risks cluster includes smoking (including second-hand smoking), dietary risks (diet high in sodium, diet high in red meat, diet low in fruits, diet low in vegetables, diet low in whole grains, and alcohol use), and low physical activity. ¶Environmental risks cluster includes air pollution cluster, suboptimal ambient temperature and lead exposure. ¦Metabolic risks cluster includes high fasting plasma glucose, high LDL cholesterol, high systolic blood pressure, high body-mass index, and low kidney dysfunction. **Age-standardised total percentage of DALYs due to all risk factors combined.

Supplement Table 8. Subarachnoid haemorrhage related DALYs (absolute numbers [in millions with 95% UI] and percentage [with 95% UI]) associated with risk factors and their clusters in 2019, by World Bank country income level, all ages, both sexes

	Word Bank High Income countries		World Bank Low Income countries		World Bank Low Middle Income countries		World Bank Upper Middle-Income countries		Globally	
	Absolute number	Percentage (95% UI)	Absolute number	Percentage (95% UI)	Absolute number	Percentage (95% UI)	Absolute number	Percentage (95% UI)	Absolute number	Percentage (95% UI)
Environmental and occupational risks										
Ambient $PM_{2.5}$ pollution	2.26 (1.78-2.73)	20.2 (16.7-23.4)	0.22 (0.16-0.28)	11.0 (8.4-13.8)	0.05 (0.02-0.10)	9.0 (5.2-13.7)	0.94 (0.62-1.29)	22.0 (17.0-26.6)	1.05 (0.83-1.28)	23.8 (20.3-26.9)
Household air pollution from solid fuels	1.16 (0.73-1.71)	10.3 (6.9-14.3)	0.00 (0.00-0.01)	0.2 (0.1-0.4)	0.17 (0.09-0.34)	32.7 (26.5-38.5)	0.74 (0.45-1.10)	17.3 (12.2-22.9)	0.24 (0.12-0.41)	5.5 (2.9-9.2)
Low ambient temperature	0.62 (0.45-0.80)	5.5 (4.1-7.3)	0.16 (0.12-0.21)	8.2 (6.1-10.5)	0.02 (0.01-0.04)	4.3 (2.6-6.0)	0.11 (0.03-0.20)	2.5 (0.6-4.2)	0.33 (0.23-0.45)	7.4 (5.5-10.0)
High ambient temperature	0.10 (0.01-0.21)	0.9 (0.1-1.8)	0.00 (0.00-0.01)	0.1 (0.0-0.3)	0.01 (0.00-0.02)	1.1 (0.2-2.7)	0.08 (0.01-0.17)	1.8 (0.3-3.5)	0.01 (0.00-0.04)	0.3 (-0.1-0.8)
Lead exposure	0.48 (0.24-0.78)	4.3 (2.2-6.7)	0.03 (0.00-0.06)	1.3 (0.2-3.1)	0.02 (0.01-0.05)	4.7 (2.3-7.3)	0.25 (0.11-0.42)	5.8 (3.1-8.8)	0.18 (0.09-0.30)	4.1 (2.0-6.5)
Dietary risks										
Diet high in sodium	1.30 (0.38-2.72)	11.6 (3.4-24.2)	0.15 (0.02-0.39)	7.7 (0.9-19.7)	0.03 (0.00-0.12)	6.7 (0.8-18.9)	0.34 (0.04-0.89)	8.1 (0.9-20.4)	0.76 (0.31-1.43)	17.3 (7.5-30.6)
Diet high in red meat	0.95 (0.51-1.32)	8.5 (4.4-12.0)	0.25 (0.14-0.33)	12.4 (7.1-16.7)	0.02 (0.00-0.05)	3.8 (0.6-6.1)	0.14 (0.04-0.22)	3.2 (0.9-5.2)	0.55 (0.30-0.78)	12.4 (7.0-16.7)
Diet low in fruits	1.13 (0.61-1.86)	10.1 (5.7-16.2)	0.16 (0.08-0.28)	8.1 (4.0-14.2)	0.06 (0.02-0.13)	10.6 (5.7-17.0)	0.58 (0.28-0.98)	13.6 (7.6-20.9)	0.33 (0.16-0.60)	7.5 (3.6-13.1)
Diet low in vegetables	0.47 (0.10-0.90)	4.2 (0.9-7.9)	0.05 (0.01-0.12)	2.6 (0.3-5.8)	0.04 (0.01-0.10)	8.0 (2.2-13.5)	0.27 (0.05-0.53)	6.4 (1.4-11.8)	0.10 (0.02-0.20)	2.3 (0.4-4.5)
Tobacco smoking										
Smoking	2.13 (1.87-2.47)	19.1 (17.5-20.8)	0.47 (0.43-0.52)	23.8 (22.1-25.6)	0.05 (0.03-0.11)	9.9 (8.1-12.0)	0.63 (0.45-0.87)	14.8 (12.9-17.5)	0.97 (0.79-1.18)	22.1 (19.8-24.1)
Second-hand smoking	0.45 (0.33-0.59)	4.0 (3.0-5.1)	0.06 (0.04-0.07)	3.0 (2.2-3.8)	0.01 (0.01-0.03)	2.9 (2.0-3.9)	0.18 (0.12-0.26)	4.3 (3.1-5.7)	0.19 (0.14-0.26)	4.4 (3.3-5.6)
Physiological factors										

High BMI	4.04 (2.72-5.49)	36.2 (24.9-47.3)	0.80 (0.57-1.04)	40.1 (28.9-51.3)	0.14 (0.06-0.29)	26.4 (15.4-38.7)	1.44 (0.88-2.16)	34.0 (22.6-45.2)	1.66 (1.10-2.28)	37.8 (25.2-50.1)
High fasting plasma glucose	1.96 (1.23-2.81)	17.5 (11.3-24.8)	0.42 (0.27-0.58)	20.9 (13.8-29.2)	0.07 (0.03-0.13)	12.6 (7.7-18.8)	0.73 (0.41-1.15)	17.2 (10.7-25.0)	0.74 (0.48-1.04)	16.8 (11.0-23.4)
High systolic blood pressure	6.36 (5.07-7.62)	56.9 (47.7-65.3)	1.08 (0.88-1.30)	54.5 (45.3-63.0)	0.26 (0.14-0.50)	49.8 (39.4-58.3)	2.42 (1.68-3.20)	56.9 (46.7-65.4)	2.58 (2.09-3.18)	58.7 (49.4-67.1)
Cluster of risk factors										
Air pollution*	3.42 (2.78-4.15)	30.5 (27.3-33.7)	0.22 (0.17-0.29)	11.2 (8.5-14.0)	0.22 (0.12-0.42)	41.6 (37.1-45.4)	1.68 (1.19-2.24)	39.3 (35.8-42.8)	1.30 (1.06-1.56)	29.4 (26.1-32.5)
Tobacco smoke†	2.50 (2.18-2.88)	22.4 (20.6-24.4)	0.52 (0.47-0.57)	25.9 (24.2-27.8)	0.07 (0.04-0.14)	12.4 (10.6-14.5)	0.79 (0.57-1.09)	18.6 (16.6-21.0)	1.13 (0.91-1.34)	25.6 (23.4-27.6)
Dietary risks‡	3.71 (2.60-4.97)	33.2 (23.9-42.9)	0.60 (0.42-0.80)	30.4 (21.3-40.3)	0.15 (0.07-0.30)	27.9 (18.8-38.0)	1.36 (0.83-2.04)	31.8 (22.3-41.7)	1.60 (1.10-2.17)	36.3 (26.3-46.4)
Behavioural risks§	5.25 (4.30-6.44)	47.0 (40.0-54.6)	0.94 (0.80-1.11)	47.4 (40.8-54.9)	0.19 (0.10-0.37)	36.0 (28.0-45.1)	1.86 (1.26-2.59)	43.5 (35.7-51.5)	2.26 (1.78-2.81)	51.3 (43.6-59.2)
Environmental and occupational risks¶	4.20 (3.47-4.99)	37.5 (33.9-41.1)	0.39 (0.32-0.46)	19.6 (16.2-23.5)	0.25 (0.14-0.47)	47.0 (42.4-50.9)	1.92 (1.35-2.55)	44.9 (41.0-48.8)	1.64 (1.34-1.96)	37.2 (33.6-41.0)
Metabolic risks¦	7.56 (6.31-8.91)	67.6 (59.4-74.8)	1.36 (1.16-1.54)	68.2 (59.5-75.4)	0.30 (0.16-0.58)	57.8 (47.5-66.2)	2.84 (2.01-3.74)	66.8 (58.0-74.2)	3.05 (2.52-3.67)	69.3 (60.5-76.4)
Combined risk factors										
All factors	9.46 (8.28-10.80)	84.6 (81.3-87.6)	1.67 (1.51-1.83)	83.8 (79.3-87.5)	0.40 (0.22-0.77)	76.3 (67.8-81.3)	3.57 (2.62-4.64)	83.9 (80.1-86.9)	3.81 (3.28-4.40)	86.6 (83.1-89.4)

Percentages and number of DALYs are not mutually exclusive. The sum of percentages and number of DALYs in the columns exceeds the totals for all risk factors combined because of overlap between various risk factors. 0% represents very low numbers. *Air pollution cluster includes ambient PM2.5 pollution and household air pollution. †Tobacco smoke includes smoking and second-hand smoking. ‡Dietary risks cluster includes diet high in sodium, diet low in fruits, diet low in vegetables, and diet low in whole grains. §Behavioural risks cluster includes smoking (including second-hand smoking), dietary risks (diet high in sodium, diet high in red meat, diet low in fruits, diet low in vegetables, diet low in whole grains, and alcohol use), and low physical activity. ¶Environmental risks cluster includes air pollution cluster, suboptimal ambient temperature and lead exposure. ¦Metabolic risks cluster includes high fasting plasma glucose, high LDL cholesterol, high systolic blood pressure, high body-mass index, and low kidney dysfunction. **Age-standardised total percentage of DALYs due to all risk factors combined.

Supplement Table 9a. Percent of DALYs (with 95% uncertainty intervals) due to stroke for high systolic blood pressure, high body-mass index, high fasting plasma glucose, high LDL cholesterol, low glomerular filtration rate, diet low in vegetable, diet low in fruits and diet high in sodium by location for both sexes combined in 2019, all ages

Country, region	High systolic blood pressure	High body-mass index	High fasting plasma glucose	High LDL cholesterol	Low glomerular filtration rate	Diet low in vegetables	Diet high in red meat	Diet low in fruits	Diet high in sodium
GBD regions									
High-income Asia Pacific	47.6 (40.0-55.5)	11.7 (5.0-19.4)	17.0 (10.5-28.9)	11.6 (4.5-22.9)	7.0 (4.7-9.2)	0.8 (0.3-1.8)	5.5 (2.9-7.6)	6.0 (3.3-9.2)	10.1 (1.6-21.8)
Central Asia	62.2 (54.4-68.4)	40.2 (28.6-51.4)	25.8 (17.4-36.3)	10.1 (6.7-15.7)	9.4 (8.0-10.8)	0.9 (0.3-1.5)	11.2 (7.4-14.2)	7.5 (4.2-12.1)	7.0 (0.6-19.0)
East Asia	54.5 (45.9-62.2)	17.6 (8.6-27.5)	16.4 (11.1-23.8)	9.6 (5.5-16.7)	8.0 (6.7-9.3)	0.3 (0.2-0.6)	10.6 (7.2-13.5)	5.7 (3.0-9.3)	21.5 (9.9-34.5)
Southeast Asia	61.6 (54.2-68.1)	25.0 (15.8-34.1)	19.5 (13.4-28.0)	7.3 (4.5-12.2)	10.6 (8.9-12.3)	5.1 (1.5-8.5)	4.2 (1.8-6.3)	8.0 (4.5-12.7)	13.0 (2.3-26.8)
South Asia	55.8 (47.7-62.4)	22.1 (13.3-31.3)	24.7 (17.1-34.4)	5.9 (3.4-9.8)	8.4 (7.0-9.8)	5.7 (1.8-9.6)	1.5 (0.6-2.4)	12.8 (7.7-18.2)	7.1 (0.6-19.0)
Southeast Asia, East Asia, and Oceania	56.4 (48.3-63.5)	19.7 (10.9-29.4)	17.3 (11.9-24.8)	9.0 (5.2-15.5)	8.7 (7.4-10.2)	1.7 (0.6-2.8)	8.8 (5.8-11.5)	6.3 (3.4-10.1)	19.1 (7.8-32.3)
Oceania	44.2 (35.9-52.8)	33.0 (20.3-46.2)	31.3 (20.7-43.2)	4.6 (3.1-6.8)	8.8 (7.2-10.5)	7.7 (2.5-13.0)	6.1 (2.0-9.2)	10.1 (5.6-16.3)	6.6 (0.6-17.3)
Australasia	47.6 (39.0-56.7)	26.5 (18.3-35.2)	19.5 (10.8-36.2)	14.4 (5.5-28.8)	6.1 (3.5-8.5)	2.4 (0.7-4.5)	13.2 (10.1-15.7)	5.3 (2.6-8.5)	2.6 (0.2-9.9)
Central Europe, Eastern Europe, and Central Asia	59.3 (51.5-66.1)	33.8 (23.7-43.5)	20.4 (13.5-30.5)	15.7 (8.3-27.5)	8.8 (7.2-10.5)	1.5 (0.5-2.6)	8.8 (5.5-11.3)	6.2 (3.2-9.7)	8.9 (1.8-19.7)
Central Europe	57.3 (48.7-64.7)	30.2 (21.0-39.4)	25.5 (15.5-40.9)	14.9 (6.8-28.3)	7.5 (5.8-9.2)	0.9 (0.3-1.6)	9.0 (6.0-11.3)	4.9 (2.4-7.9)	16.1 (5.2-28.8)
Eastern Europe	59.4 (51.7-66.7)	33.6 (23.5-43.3)	16.8 (11.2-25.0)	17.5 (9.3-30.8)	9.2 (7.5-11.2)	1.9 (0.6-3.3)	8.1 (4.8-10.6)	6.4 (3.1-10.0)	6.3 (0.6-16.3)
Western Europe	50.0 (41.7-58.9)	20.3 (12.6-29.0)	25.6 (15.0-46.6)	13.8 (4.9-28.2)	5.5 (3.2-7.6)	2.1 (0.7-3.7)	9.6 (6.7-12.0)	4.1 (2.1-6.5)	4.0 (0.3-12.4)

Central Latin America	52.8 (45.8-59.3)	36.6 (25.2-47.6)	29.6 (20.6-41.5)	9.3 (5.3-15.9)	10.3 (8.6-12.0)	4.8 (1.4-8.2)	7.6 (4.1-10.4)	5.7 (3.0-9.4)	7.9 (0.9-19.5)
Tropical Latin America	55.9 (48.7-62.5)	38.8 (28.2-49.2)	22.9 (15.3-34.5)	10.8 (5.9-18.8)	6.9 (5.7-8.1)	5.0 (1.5-8.3)	14.3 (10.4-17.5)	4.4 (2.2-7.2)	7.0 (0.4-18.7)
Southern Latin America	52.2 (44.3-59.5)	32.6 (21.5-43.6)	23.4 (15.4-34.8)	9.6 (4.5-17.9)	6.7 (5.1-8.1)	3.0 (0.8-5.7)	14.4 (10.7-17.5)	4.4 (2.3-7.0)	6.8 (0.4-18.2)
Andean Latin America	41.9 (34.8-48.8)	41.6 (30.0-52.1)	18.1 (12.1-26.5)	8.4 (4.9-14.1)	6.4 (5.2-7.5)	5.9 (2.0-9.6)	6.9 (3.4-9.6)	5.6 (2.9-9.3)	6.1 (0.3-17.1)
Latin America and Caribbean	53.7 (46.8-60.2)	37.2 (26.4-47.6)	25.2 (17.2-36.2)	9.8 (5.5-16.8)	8.1 (6.7-9.4)	5.1 (1.6-8.4)	10.3 (6.9-13.1)	5.0 (2.6-8.2)	6.8 (0.6-18.0)
High-income North America	45.0 (37.2-52.6)	34.3 (24.5-43.3)	28.8 (19.4-43.7)	10.6 (5.0-19.8)	7.1 (5.1-9.1)	2.5 (0.9-4.4)	11.1 (7.9-13.7)	5.3 (2.7-8.6)	5.1 (0.3-15.1)
North Africa and Middle East	53.5 (46.7-59.6)	39.4 (29.1-48.8)	25.7 (17.2-38.5)	15.8 (10.7-23.6)	10.5 (8.7-12.3)	1.9 (0.9-3.1)	4.2 (1.6-6.2)	4.5 (2.4-7.0)	2.0 (0.3-7.7)
Sub-Saharan Africa	56.7 (49.0-62.7)	27.8 (18.5-37.4)	15.2 (10.5-21.8)	5.4 (3.4-8.6)	6.2 (5.2-7.2)	6.7 (2.6-10.8)	3.7 (1.4-5.7)	9.1 (5.4-13.8)	6.2 (0.3-19.9)
Central Sub-Saharan Africa	58.2 (49.7-64.9)	23.4 (13.3-33.9)	19.4 (13.3-27.1)	5.0 (3.0-8.0)	6.0 (5.0-7.1)	8.3 (3.5-13.1)	2.9 (1.2-4.4)	9.4 (5.4-15.1)	3.2 (0.2-14.0)
Southern Sub-Saharan Africa	62.1 (54.6-68.6)	39.7 (30.1-48.8)	26.5 (17.8-37.9)	8.9 (5.2-15.0)	8.1 (6.8-9.5)	6.0 (2.4-9.3)	7.6 (4.2-10.4)	10.4 (6.2-15.3)	4.2 (0.2-16.4)
Eastern Sub-Saharan Africa	55.3 (47.6-61.8)	24.7 (15.2-34.6)	12.9 (8.7-18.8)	4.5 (2.7-7.4)	5.9 (4.9-6.9)	7.7 (3.0-12.2)	3.4 (1.0-5.4)	9.6 (5.8-14.5)	8.7 (0.4-24.5)
Western Sub-Saharan Africa	56.4 (49.1-62.1)	29.6 (20.0-39.2)	13.7 (9.3-19.9)	5.6 (3.6-8.6)	6.1 (5.2-7.1)	5.4 (1.8-9.2)	3.5 (1.1-5.3)	8.2 (4.9-12.6)	5.1 (0.2-18.4)
Countries in alphabetical order									
Afghanistan	49.5 (40.3-58.3)	35.8 (24.2-47.1)	27.7 (18.8-39.1)	13.8 (9.6-19.3)	8.8 (7.4-10.2)	8.7 (3.9-13.0)	5.7 (2.2-8.6)	10.5 (6.0-16.2)	2.0 (0.2-7.8)
Albania	59.4 (48.9-68.9)	26.0 (16.2-36.4)	13.6 (9.2-19.9)	5.8 (2.9-10.5)	7.0 (5.5-8.4)	0.3 (0.2-0.5)	8.4 (4.8-11.4)	2.2 (1.1-3.5)	18.1 (5.9-31.7)
Algeria	50.8 (42.7-59.2)	37.2 (26.9-47.0)	29.6 (18.9-47.3)	16.3 (10.3-25.8)	11.0 (8.6-13.3)	2.2 (0.6-4.1)	3.9 (1.4-5.9)	4.4 (1.9-7.1)	1.9 (0.3-7.2)
American Samoa	59.8 (51.3-67.1)	53.5 (42.5-62.6)	34.8 (24.1-46.7)	6.8 (4.4-10.6)	11.8 (10.0-13.8)	6.9 (2.4-11.3)	7.9 (3.6-11.3)	7.4 (3.9-12.2)	3.5 (0.2-12.6)

Andorra	55.3 (46.7-64.0)	26.2 (16.9-35.6)	20.8 (12.0-35.7)	14.4 (6.2-27.6)	5.0 (3.0-6.8)	1.6 (0.4-3.4)	11.6 (8.3-14.3)	4.0 (2.0-6.5)	3.5 (0.2-12.0)
Angola	60.5 (51.7-67.6)	25.1 (14.3-35.8)	19.4 (12.5-27.8)	5.5 (3.5-8.8)	6.0 (5.0-7.1)	5.9 (1.6-10.0)	4.8 (1.6-7.3)	8.0 (4.2-13.4)	4.4 (0.2-17.4)
Antigua and Barbuda	54.0 (45.0-62.0)	34.4 (23.5-45.2)	32.8 (22.3-45.7)	8.3 (4.6-14.8)	9.5 (7.8-11.1)	5.2 (1.5-8.6)	4.8 (1.8-7.2)	5.0 (2.5-8.4)	4.2 (0.2-14.3)
Argentina	50.2 (42.1-58.3)	32.8 (21.5-44.2)	21.8 (14.3-32.3)	8.7 (4.0-16.1)	6.5 (5.1-7.8)	3.3 (0.8-6.4)	15.7 (11.7-19.0)	3.7 (2.0-6.0)	6.4 (0.3-17.8)
Armenia	58.6 (50.4-66.3)	33.6 (23.6-43.2)	28.5 (17.0-46.7)	11.2 (6.2-19.6)	8.4 (6.8-10.0)	0.2 (0.2-0.2)	7.3 (4.1-9.9)	3.4 (1.6-5.5)	6.6 (0.5-17.8)
Australia	47.5 (38.6-56.9)	26.7 (18.6-35.3)	20.1 (10.9-37.0)	14.5 (5.5-29.0)	6.1 (3.5-8.5)	2.4 (0.7-4.5)	13.2 (10.1-15.8)	5.4 (2.7-8.8)	2.4 (0.2-9.2)
Austria	53.4 (44.4-62.9)	21.0 (12.8-29.7)	22.5 (12.6-41.4)	15.4 (5.9-30.5)	5.8 (3.5-8.1)	2.1 (0.6-4.0)	10.5 (7.3-13.0)	3.2 (1.6-5.1)	6.3 (0.3-16.8)
Azerbaijan	57.2 (47.8-65.8)	40.3 (27.2-52.1)	27.1 (17.5-40.1)	7.6 (4.8-12.1)	9.5 (8.0-11.0)	0.3 (0.2-0.5)	6.5 (2.8-9.3)	5.3 (2.6-9.0)	6.7 (0.5-18.5)
Bahamas	56.7 (47.6-64.4)	45.1 (32.3-56.4)	29.1 (19.8-40.3)	8.3 (5.2-13.3)	9.0 (7.5-10.6)	3.4 (0.8-6.9)	9.8 (5.3-13.2)	6.9 (3.6-11.2)	4.3 (0.2-14.7)
Bahrain	58.9 (50.7-66.4)	53.5 (40.6-63.5)	30.2 (21.0-41.8)	18.0 (12.8-24.9)	10.7 (8.9-12.4)	0.8 (0.2-2.0)	6.9 (3.1-9.9)	3.6 (1.7-6.0)	2.4 (0.3-9.2)
Bangladesh	55.5 (46.8-63.0)	17.2 (9.4-25.9)	17.3 (11.4-25.5)	4.3 (2.1-8.0)	6.3 (5.2-7.5)	7.4 (3.0-11.7)	1.1 (0.3-1.9)	11.5 (6.7-16.9)	6.3 (0.3-18.0)
Barbados	55.7 (46.8-63.6)	32.4 (22.0-42.8)	28.8 (19.1-43.1)	10.7 (5.3-20.4)	8.8 (7.1-10.5)	4.1 (1.2-6.7)	5.5 (2.5-7.9)	7.3 (3.9-11.5)	3.0 (0.2-11.5)
Belarus	63.6 (54.6-71.7)	33.1 (22.8-43.4)	13.1 (8.7-20.2)	14.5 (7.8-24.5)	8.5 (6.8-10.3)	0.6 (0.2-1.3)	12.4 (9.1-15.3)	6.1 (2.9-9.9)	3.6 (0.2-12.3)
Belgium	54.5 (45.1-64.0)	19.9 (12.4-28.3)	22.0 (12.4-40.2)	12.3 (4.4-25.4)	5.7 (3.4-7.9)	1.3 (0.3-2.8)	10.5 (7.4-13.1)	4.7 (2.3-7.8)	5.6 (0.3-15.9)
Belize	51.9 (42.8-59.5)	46.1 (33.4-57.0)	22.3 (15.0-31.8)	8.0 (5.1-12.6)	8.8 (7.4-10.3)	6.6 (2.2-10.7)	4.0 (1.2-6.3)	1.4 (0.7-2.4)	4.2 (0.2-14.3)
Benin	54.7 (46.6-61.4)	34.2 (23.9-44.5)	17.9 (11.4-26.3)	4.9 (3.1-7.9)	6.1 (5.1-7.1)	5.1 (1.2-8.9)	2.0 (0.5-3.4)	9.7 (5.6-14.8)	6.0 (0.2-20.8)
Bermuda	50.4 (41.7-58.2)	32.6 (22.4-42.5)	23.2 (14.1-37.8)	12.1 (5.5-23.0)	8.5 (6.5-10.3)	2.5 (0.7-4.6)	9.8 (6.5-12.5)	5.8 (3.0-9.3)	4.3 (0.2-14.3)

Bhutan	53.5 (44.9-61.1)	25.8 (15.2-36.1)	21.0 (13.6-32.0)	8.6 (4.8-14.7)	7.8 (6.5-9.1)	5.4 (1.6-9.0)	2.0 (0.6-3.4)	8.7 (5.0-13.7)	6.3 (0.3-18.0)
Bolivia (Plurinational State of)	38.5 (30.2-46.8)	41.2 (29.4-52.6)	20.3 (13.3-29.7)	6.7 (3.9-11.6)	6.1 (5.1-7.2)	6.7 (2.4-10.8)	9.2 (4.9-12.6)	6.9 (3.5-11.8)	6.2 (0.3-17.5)
Bosnia and Herzegovina	57.1 (47.0-66.4)	26.0 (17.2-35.0)	33.6 (19.6-56.7)	17.8 (8.9-32.8)	8.0 (6.1-9.8)	0.8 (0.2-1.6)	3.5 (1.3-5.2)	4.2 (1.7-7.1)	16.6 (5.1-29.9)
Botswana	63.1 (54.9-70.0)	41.8 (31.3-51.8)	26.3 (17.7-37.7)	6.3 (3.7-10.9)	8.5 (7.2-9.9)	6.8 (2.6-10.7)	7.4 (3.5-10.4)	12.0 (7.2-17.4)	4.7 (0.2-18.1)
Brazil	55.9 (48.7-62.5)	38.8 (28.2-49.2)	22.9 (15.3-34.5)	10.8 (5.9-18.8)	6.9 (5.7-8.1)	5.0 (1.4-8.2)	14.3 (10.4-17.5)	4.4 (2.2-7.2)	7.0 (0.4-18.7)
Brunei Darussalam	56.3 (47.8-63.8)	25.9 (14.2-37.4)	34.5 (24.0-46.6)	10.6 (6.9-16.2)	8.5 (7.1-9.8)	5.5 (1.5-9.3)	5.8 (2.3-8.5)	9.6 (5.5-15.0)	12.9 (2.1-26.7)
Bulgaria	54.1 (44.2-62.4)	28.9 (19.5-38.2)	24.8 (14.1-42.3)	14.4 (6.7-27.5)	8.2 (6.4-9.9)	0.4 (0.2-0.8)	10.4 (7.2-13.0)	5.7 (2.8-9.2)	17.0 (5.6-30.1)
Burkina Faso	47.4 (39.0-55.4)	24.8 (14.4-36.2)	15.6 (10.0-23.2)	2.1 (1.2-3.5)	5.5 (4.6-6.6)	7.7 (3.0-12.3)	4.7 (1.3-7.2)	13.4 (8.0-19.1)	4.9 (0.2-18.0)
Burundi	55.3 (46.5-63.6)	16.2 (7.4-26.6)	11.6 (7.7-17.8)	3.8 (2.3-6.0)	5.7 (4.7-6.8)	8.2 (3.2-13.0)	1.0 (0.3-1.8)	4.8 (2.4-7.9)	8.5 (0.3-25.1)
CÃ´te d'Ivoire	60.5 (51.9-67.6)	34.9 (23.8-45.4)	15.3 (10.1-22.2)	5.6 (3.8-8.2)	6.3 (5.2-7.4)	6.8 (2.2-11.0)	4.7 (1.5-7.2)	7.7 (3.9-12.9)	5.8 (0.2-20.4)
Cabo Verde	63.4 (55.1-70.5)	33.6 (23.9-43.9)	22.4 (15.2-33.5)	6.8 (3.9-11.4)	6.7 (5.5-7.8)	2.6 (0.6-5.6)	4.1 (1.4-6.2)	8.9 (5.0-14.0)	5.6 (0.2-20.3)
Cambodia	40.7 (31.9-49.8)	16.8 (8.5-26.6)	26.4 (17.3-38.0)	5.9 (3.3-10.3)	9.5 (8.0-11.1)	7.7 (3.1-12.2)	4.1 (1.2-6.4)	11.3 (6.6-16.9)	11.9 (1.5-25.7)
Cameroon	57.7 (49.0-64.5)	42.8 (31.3-54.4)	12.8 (8.9-18.0)	2.5 (1.4-4.2)	7.1 (5.9-8.2)	3.8 (0.7-7.6)	3.9 (1.1-6.2)	6.3 (3.1-10.8)	5.3 (0.2-19.7)
Canada	44.8 (35.6-53.9)	27.5 (18.6-36.5)	20.6 (11.4-37.2)	13.1 (5.4-25.0)	5.7 (3.6-7.6)	2.1 (0.6-4.0)	9.6 (6.4-12.2)	4.8 (2.2-7.9)	5.1 (0.3-15.1)
Caribbean	54.3 (46.8-61.2)	30.5 (20.2-40.5)	26.8 (18.4-37.7)	8.6 (5.0-14.5)	8.0 (6.7-9.4)	5.3 (2.1-8.7)	4.5 (1.9-6.8)	5.2 (2.8-8.4)	4.1 (0.2-14.0)
Central African Republic	59.7 (50.0-67.6)	16.7 (7.3-27.7)	19.6 (12.3-28.1)	3.9 (2.4-6.2)	5.9 (4.8-7.2)	9.6 (3.9-15.0)	10.3 (5.1-14.2)	10.6 (6.0-17.1)	4.4 (0.2-17.9)

Chad	46.6 (38.4-54.2)	18.2 (9.6-27.4)	13.1 (8.6-18.9)	4.1 (2.6-6.3)	5.3 (4.4-6.3)	8.0 (3.4-12.3)	4.5 (1.4-6.9)	11.9 (7.0-17.3)	4.7 (0.2-17.7)
Chile	58.0 (49.6-66.0)	33.7 (22.7-44.2)	29.3 (18.8-46.6)	11.0 (5.2-20.5)	7.2 (5.3-9.1)	1.8 (0.4-4.0)	11.4 (7.8-14.4)	5.9 (2.9-9.8)	7.6 (0.4-19.7)
China	54.8 (46.2-62.6)	17.8 (8.7-27.6)	16.4 (11.1-23.7)	9.6 (5.5-16.8)	8.0 (6.7-9.3)	0.3 (0.2-0.4)	10.8 (7.3-13.7)	5.7 (2.9-9.2)	21.7 (10.1-34.8)
Colombia	47.8 (39.8-55.4)	33.8 (22.9-44.3)	27.6 (18.4-40.7)	9.2 (4.8-16.3)	7.6 (6.2-9.0)	5.0 (1.4-8.4)	7.5 (3.7-10.4)	5.1 (2.6-8.6)	12.0 (2.7-24.4)
Comoros	56.6 (48.4-63.7)	25.9 (15.9-35.4)	13.4 (8.0-22.3)	7.1 (4.1-12.3)	6.2 (5.2-7.2)	8.1 (3.5-12.3)	2.1 (0.6-3.6)	6.4 (3.1-11.0)	9.3 (0.3-26.5)
Congo	64.0 (54.8-71.5)	39.6 (28.7-49.3)	20.9 (14.1-29.5)	6.5 (4.1-10.3)	6.7 (5.5-7.8)	8.6 (3.7-13.2)	4.8 (1.6-7.3)	8.9 (4.9-14.6)	4.6 (0.2-18.3)
Cook Islands	55.2 (45.8-63.2)	44.5 (32.9-55.0)	33.8 (23.8-45.5)	9.2 (5.6-15.3)	11.4 (9.7-13.3)	5.7 (1.9-9.3)	8.0 (4.2-11.0)	5.8 (2.9-9.8)	9.0 (0.7-21.5)
Costa Rica	61.5 (53.4-68.7)	34.5 (23.4-45.0)	28.3 (18.9-42.6)	11.1 (5.7-19.6)	10.4 (8.4-12.5)	5.6 (2.0-8.9)	6.2 (2.8-8.8)	4.6 (2.3-7.6)	8.6 (0.7-20.6)
Croatia	57.5 (47.8-66.7)	27.4 (18.2-36.5)	29.6 (16.8-51.6)	14.2 (5.2-28.8)	7.8 (5.7-9.8)	2.3 (0.7-4.0)	6.1 (3.3-8.3)	4.1 (1.9-6.7)	17.0 (5.6-29.8)
Cuba	46.2 (37.7-54.1)	30.2 (20.3-40.3)	27.6 (17.6-43.9)	11.1 (5.7-20.4)	8.3 (6.7-9.8)	1.9 (0.5-3.9)	6.1 (3.0-8.5)	3.9 (2.0-6.3)	4.1 (0.2-14.1)
Cyprus	52.9 (43.1-62.2)	19.0 (11.7-27.0)	30.3 (18.0-52.2)	13.0 (4.7-26.7)	6.5 (4.2-8.7)	2.8 (0.9-5.1)	7.7 (4.7-10.2)	4.4 (2.1-7.3)	4.2 (0.2-13.6)
Czechia	48.2 (38.5-57.0)	29.3 (19.7-38.8)	33.0 (19.7-54.8)	16.2 (6.8-31.1)	7.1 (5.2-8.9)	2.7 (0.9-4.6)	8.5 (5.4-11.0)	5.4 (2.4-8.5)	16.1 (5.4-28.8)
Democratic People's Republic of Korea	47.0 (37.7-55.5)	8.9 (1.9-19.8)	14.7 (10.2-21.1)	8.2 (5.3-13.6)	8.7 (7.3-10.2)	2.7 (0.6-6.0)	3.2 (0.9-5.1)	7.5 (3.8-12.7)	16.7 (4.9-31.3)
Democratic Republic of the Congo	56.7 (47.7-64.2)	21.7 (11.6-32.7)	19.1 (13.1-27.0)	4.7 (2.8-7.7)	5.9 (4.9-7.0)	9.0 (3.6-13.9)	1.2 (0.4-2.0)	10.0 (5.8-15.8)	2.6 (0.2-12.0)
Denmark	56.5 (47.3-65.2)	20.7 (13.0-28.9)	19.2 (11.0-33.8)	13.3 (5.1-26.8)	5.8 (3.8-7.7)	1.9 (0.5-3.6)	10.3 (7.2-12.8)	3.6 (1.8-5.8)	4.4 (0.2-14.3)
Djibouti	58.9 (50.0-66.6)	26.5 (15.5-37.9)	13.5 (8.7-20.7)	6.0 (3.8-9.8)	6.4 (5.4-7.6)	6.3 (1.9-10.4)	6.3 (2.4-9.2)	13.1 (7.8-19.0)	8.6 (0.3-24.9)

Dominica	50.7 (42.2-59.2)	33.5 (22.9-43.6)	34.2 (23.2-49.7)	8.9 (4.2-17.4)	9.3 (7.5-11.0)	3.4 (1.0-6.1)	5.2 (2.3-7.5)	0.4 (0.2-0.5)	4.2 (0.2-14.4)
Dominican Republic	53.9 (44.4-61.6)	37.9 (25.5-49.7)	17.6 (11.3-26.8)	8.8 (5.5-14.3)	8.0 (6.7-9.3)	5.0 (1.2-8.6)	5.5 (2.1-8.3)	1.8 (1.0-2.9)	4.3 (0.2-14.6)
Ecuador	38.9 (31.0-46.4)	45.8 (34.8-55.1)	22.5 (14.6-34.2)	9.0 (5.3-14.8)	6.9 (5.7-8.1)	5.9 (2.1-9.6)	8.9 (4.9-12.0)	3.2 (1.6-5.0)	6.1 (0.3-16.9)
Egypt	48.6 (40.8-55.9)	43.4 (31.8-53.5)	21.4 (13.0-33.9)	15.3 (10.4-22.1)	10.0 (8.0-11.9)	0.2 (0.2-0.2)	4.6 (1.8-6.8)	2.9 (1.4-4.6)	1.9 (0.2-7.2)
El Salvador	51.7 (43.6-59.5)	34.6 (23.5-44.8)	28.3 (19.3-41.3)	9.3 (4.9-16.5)	9.8 (8.1-11.4)	4.8 (1.3-8.2)	2.8 (0.8-4.4)	6.9 (3.6-11.4)	7.7 (0.6-19.5)
Equatorial Guinea	62.0 (53.5-68.6)	41.0 (30.5-51.1)	21.4 (14.5-31.3)	6.9 (4.0-11.5)	6.4 (5.3-7.5)	7.0 (3.0-10.9)	6.3 (2.7-9.0)	5.1 (2.6-8.3)	4.4 (0.2-16.9)
Eritrea	50.1 (40.5-58.3)	20.0 (10.3-30.5)	12.2 (7.8-18.7)	4.0 (2.6-6.3)	6.0 (5.0-7.2)	8.8 (3.4-14.0)	3.4 (0.8-5.6)	10.5 (6.0-16.8)	8.6 (0.3-25.1)
Estonia	56.3 (47.3-65.4)	31.5 (22.3-40.7)	15.2 (9.6-24.0)	18.0 (8.6-32.7)	8.8 (6.8-10.7)	1.8 (0.5-3.5)	7.3 (4.1-9.7)	5.7 (2.6-9.2)	2.2 (0.3-8.2)
Eswatini	61.0 (52.4-68.0)	46.1 (33.7-57.3)	25.5 (17.1-36.5)	5.2 (2.9-9.2)	8.4 (7.1-9.9)	7.7 (3.3-11.7)	7.2 (3.3-10.2)	8.9 (5.1-14.1)	4.6 (0.2-18.3)
Ethiopia	43.9 (36.7-51.0)	18.9 (10.1-28.7)	10.1 (7.0-14.6)	3.2 (1.8-5.5)	5.5 (4.6-6.5)	7.8 (3.0-12.3)	2.8 (0.8-4.6)	13.3 (8.0-19.1)	8.4 (0.4-24.2)
Fiji	63.6 (55.8-70.7)	48.9 (36.6-58.8)	33.3 (23.8-44.7)	9.4 (6.5-14.1)	10.7 (9.2-12.4)	6.3 (2.1-10.3)	9.1 (4.6-12.4)	11.3 (6.7-16.8)	8.3 (0.6-20.3)
Finland	54.4 (44.5-64.0)	20.5 (12.7-29.3)	29.2 (16.8-52.2)	13.6 (4.9-28.0)	4.9 (2.9-6.7)	2.7 (0.9-4.7)	8.9 (5.7-11.3)	5.3 (2.5-8.6)	4.1 (0.3-13.0)
France	51.2 (42.5-61.1)	20.2 (12.9-28.2)	12.3 (7.5-20.6)	14.1 (5.3-27.9)	5.0 (2.8-7.1)	2.9 (1.0-5.1)	11.7 (8.6-14.1)	5.5 (2.8-8.9)	3.6 (0.2-12.3)
Gabon	59.2 (50.7-66.6)	39.6 (28.2-50.9)	26.2 (17.9-36.9)	7.2 (4.2-12.1)	7.0 (5.8-8.2)	7.0 (2.8-10.9)	11.0 (6.7-14.4)	3.4 (1.8-5.3)	4.4 (0.2-17.2)
Gambia	60.5 (52.3-67.5)	32.8 (22.1-43.2)	15.4 (9.9-23.3)	6.3 (4.1-9.6)	6.3 (5.3-7.3)	7.7 (3.2-12.0)	2.0 (0.5-3.5)	13.7 (8.6-19.2)	5.3 (0.2-19.2)
Georgia	61.2 (52.1-69.1)	30.2 (20.5-39.8)	31.0 (20.5-46.2)	7.3 (3.5-14.0)	7.8 (6.6-9.1)	2.5 (0.6-5.1)	4.7 (1.9-7.0)	7.0 (3.7-11.4)	7.2 (0.6-18.6)
Germany	54.5 (45.0-64.5)	21.5 (13.4-30.1)	30.5 (17.7-56.7)	15.8 (6.1-32.2)	6.0 (3.6-8.3)	2.5 (0.8-4.3)	10.0 (6.8-12.3)	4.7 (2.1-7.7)	4.3 (0.3-13.3)

Ghana	60.1 (51.9-67.1)	41.9 (30.6-52.2)	18.5 (12.5-27.1)	9.7 (6.5-14.5)	6.8 (5.7-7.9)	6.1 (2.0-9.9)	2.8 (0.8-4.6)	3.7 (1.9-6.1)	8.1 (0.2-25.3)
Greece	46.3 (37.1-56.5)	18.0 (10.5-26.4)	21.3 (12.1-38.8)	12.0 (3.2-26.5)	6.2 (3.5-8.9)	0.4 (0.2-0.7)	9.8 (6.8-12.3)	1.6 (0.9-2.8)	3.8 (0.2-12.5)
Greenland	48.0 (38.9-56.6)	39.4 (26.6-51.0)	22.9 (14.3-34.9)	8.8 (4.9-15.2)	5.8 (4.7-6.9)	1.6 (0.3-3.9)	13.8 (9.6-17.1)	5.5 (2.7-8.9)	6.0 (0.3-17.2)
Grenada	53.0 (44.2-60.7)	34.9 (24.0-45.4)	34.5 (23.3-48.0)	9.6 (5.5-16.7)	10.4 (8.6-12.1)	6.3 (2.6-9.8)	3.3 (1.1-5.2)	4.6 (2.3-7.5)	4.2 (0.2-14.3)
Guam	58.2 (49.8-65.3)	46.1 (32.8-57.1)	24.7 (17.3-34.5)	10.5 (6.9-16.0)	10.6 (8.9-12.3)	5.3 (1.5-8.8)	9.8 (5.5-13.1)	5.3 (2.8-8.8)	8.0 (0.6-19.7)
Guatemala	52.6 (45.4-59.6)	31.3 (19.8-42.5)	27.1 (18.7-37.7)	6.2 (3.4-10.7)	9.3 (7.8-11.0)	5.4 (1.4-9.3)	2.8 (0.7-4.5)	7.6 (4.0-12.6)	7.4 (0.4-18.6)
Guinea	51.4 (42.7-58.3)	24.3 (14.8-34.0)	14.0 (9.1-20.9)	4.7 (2.9-7.5)	5.7 (4.8-6.6)	6.5 (2.2-10.6)	2.6 (0.7-4.2)	6.3 (3.1-10.9)	4.9 (0.2-17.9)
Guinea-Bissau	58.3 (49.7-65.4)	26.9 (15.4-38.7)	14.6 (9.5-20.9)	5.0 (3.4-7.4)	6.3 (5.2-7.5)	9.1 (3.7-14.0)	5.3 (1.6-8.1)	9.9 (5.6-15.9)	5.3 (0.2-19.9)
Guyana	53.2 (43.4-61.2)	38.9 (26.3-51.1)	35.7 (24.6-48.1)	8.1 (5.1-13.1)	9.7 (8.2-11.5)	5.3 (1.4-9.1)	1.9 (0.5-3.2)	8.6 (4.7-13.8)	4.2 (0.2-14.7)
Haiti	61.2 (52.2-68.3)	21.3 (11.2-31.9)	29.0 (19.7-40.3)	6.2 (3.9-10.0)	7.3 (6.0-8.9)	8.3 (3.5-12.9)	3.1 (0.8-5.0)	7.8 (4.1-13.0)	3.8 (0.2-13.6)
Honduras	52.8 (43.8-60.9)	32.7 (20.8-44.4)	30.2 (20.4-42.2)	8.1 (4.7-14.3)	9.5 (7.9-11.2)	5.5 (1.6-9.0)	3.5 (1.1-5.7)	6.8 (3.5-11.5)	8.1 (0.6-20.2)
Hungary	52.7 (43.3-61.6)	32.3 (22.7-41.2)	29.7 (17.3-50.6)	16.3 (8.2-29.4)	7.7 (6.0-9.4)	1.1 (0.3-2.3)	7.9 (4.6-10.5)	5.6 (2.5-9.0)	19.9 (7.9-32.7)
Iceland	49.8 (40.5-59.2)	23.7 (15.2-32.4)	21.6 (12.6-39.4)	15.3 (5.9-30.1)	4.5 (2.6-6.3)	3.2 (1.1-5.4)	11.8 (8.8-14.4)	5.3 (2.6-8.6)	4.8 (0.2-14.5)
India	55.3 (47.1-62.0)	22.3 (13.6-31.2)	26.9 (18.4-37.6)	6.1 (3.6-10.2)	8.9 (7.5-10.5)	5.0 (1.4-8.8)	1.0 (0.4-1.6)	13.6 (8.4-19.2)	7.3 (0.6-19.4)
Indonesia	69.0 (61.3-75.0)	27.6 (18.2-36.8)	17.5 (11.9-25.6)	6.5 (3.9-11.1)	10.7 (9.0-12.5)	5.8 (1.8-9.6)	2.0 (0.6-3.3)	8.3 (4.6-13.2)	13.6 (2.4-27.5)
Iran (Islamic Republic of)	49.9 (42.3-56.9)	32.2 (23.4-41.0)	26.7 (16.6-43.6)	20.2 (13.1-30.9)	10.9 (8.5-13.2)	0.6 (0.2-1.1)	3.7 (1.3-5.6)	2.0 (0.9-3.2)	2.0 (0.3-7.8)
Iraq	62.8 (55.5-68.6)	43.4 (31.1-54.8)	27.8 (19.0-38.7)	14.3 (9.5-22.3)	11.8 (9.8-13.7)	0.9 (0.2-2.1)	1.9 (0.6-3.2)	7.1 (3.5-11.4)	2.3 (0.3-8.9)

Ireland	53.9 (44.8-62.7)	24.1 (15.6-33.3)	22.8 (13.1-40.5)	12.5 (4.7-25.1)	5.7 (3.6-7.6)	1.8 (0.5-3.7)	13.3 (10.2-15.9)	5.0 (2.4-8.3)	3.1 (0.2-11.1)
Israel	55.3 (47.1-63.9)	24.3 (15.2-33.9)	26.0 (15.3-44.0)	11.6 (5.0-22.4)	6.6 (4.5-8.6)	0.3 (0.2-0.4)	7.2 (4.0-9.7)	2.0 (1.1-3.3)	4.5 (0.3-14.2)
Italy	44.7 (35.9-54.5)	16.2 (9.2-24.1)	27.4 (15.5-51.3)	13.2 (3.9-28.7)	5.4 (3.0-7.7)	1.5 (0.5-2.9)	8.8 (6.1-11.2)	2.4 (1.2-3.9)	5.0 (0.4-14.1)
Jamaica	51.2 (42.7-59.4)	36.2 (26.2-46.0)	32.6 (21.8-48.1)	7.9 (3.6-16.0)	8.4 (6.9-10.0)	4.0 (1.1-7.1)	3.3 (1.1-5.2)	5.5 (2.7-9.4)	4.1 (0.2-13.9)
Japan	49.4 (41.4-58.0)	10.1 (4.0-17.5)	15.3 (9.3-26.5)	11.5 (4.1-23.4)	7.1 (4.6-9.4)	0.8 (0.3-1.7)	4.4 (2.1-6.4)	6.2 (3.5-9.7)	9.1 (1.1-20.5)
Jordan	53.6 (45.3-61.1)	45.0 (33.3-54.4)	28.2 (18.2-41.1)	19.7 (13.1-30.5)	12.8 (10.5-14.9)	2.1 (0.6-4.0)	4.1 (1.5-6.2)	6.0 (2.6-9.9)	2.0 (0.3-7.9)
Kazakhstan	67.8 (59.3-74.6)	41.7 (30.0-52.1)	29.1 (19.0-42.5)	11.8 (7.2-19.1)	9.0 (7.6-10.5)	0.3 (0.2-0.6)	14.1 (10.3-17.3)	7.1 (3.6-11.7)	7.1 (0.5-19.2)
Kenya	57.6 (49.8-64.5)	33.8 (23.7-43.7)	12.8 (8.8-18.3)	4.3 (2.6-7.0)	6.0 (5.0-7.1)	5.8 (1.6-9.9)	4.9 (1.8-7.4)	9.2 (5.2-14.6)	5.7 (0.6-16.7)
Kiribati	47.6 (37.5-57.2)	49.4 (33.5-63.6)	32.5 (22.0-45.1)	5.0 (3.5-7.2)	10.3 (8.5-12.4)	8.6 (2.9-14.1)	5.0 (1.3-7.9)	9.9 (5.2-16.8)	6.5 (0.5-17.3)
Kuwait	52.5 (45.0-60.0)	53.1 (41.4-62.0)	26.7 (18.5-37.7)	20.8 (15.0-28.5)	9.7 (7.8-11.4)	0.4 (0.2-1.0)	10.9 (6.5-14.4)	6.8 (3.1-11.1)	4.4 (0.2-13.9)
Kyrgyzstan	45.7 (36.6-54.5)	38.1 (26.2-49.9)	11.3 (7.6-16.5)	11.3 (7.5-17.4)	8.4 (7.1-9.9)	0.6 (0.2-1.5)	10.8 (6.4-14.1)	9.0 (4.9-14.0)	6.7 (0.5-18.1)
Lao People's Democratic Republic	52.8 (43.6-60.8)	26.5 (14.9-37.6)	23.9 (16.4-32.7)	6.2 (3.8-10.1)	10.6 (9.0-12.4)	5.2 (1.3-9.1)	5.7 (1.9-8.5)	7.4 (3.7-12.9)	12.2 (1.8-25.9)
Latvia	60.2 (49.7-69.7)	27.7 (18.6-37.0)	15.4 (9.4-24.9)	18.2 (7.8-35.0)	8.4 (6.4-10.5)	1.7 (0.4-3.1)	6.6 (3.5-8.9)	6.0 (2.7-9.4)	4.2 (0.3-12.9)
Lebanon	51.9 (43.6-60.0)	33.2 (22.9-42.8)	31.0 (18.8-52.8)	21.7 (12.6-35.3)	12.2 (8.9-14.9)	0.2 (0.2-0.3)	5.9 (2.6-8.3)	2.0 (0.9-3.4)	1.8 (0.3-7.0)
Lesotho	55.6 (46.2-63.8)	37.5 (26.2-48.6)	25.4 (16.3-36.1)	3.2 (1.7-5.8)	7.7 (6.4-9.0)	8.0 (3.1-12.6)	5.7 (2.0-8.5)	12.6 (7.4-18.4)	4.6 (0.2-18.1)
Liberia	58.6 (50.1-65.9)	41.6 (31.0-51.7)	18.4 (12.4-26.6)	5.7 (3.8-8.6)	6.2 (5.2-7.2)	8.1 (3.3-12.7)	2.0 (0.5-3.4)	10.0 (5.7-15.6)	5.2 (0.2-19.1)

Libya	59.2 (50.9-66.3)	45.3 (33.8-55.2)	29.7 (19.1-46.0)	19.8 (13.9-28.5)	10.7 (8.8-12.6)	2.2 (0.5-4.1)	4.7 (1.7-7.1)	5.4 (2.2-9.0)	2.0 (0.3-7.8)
Lithuania	60.5 (50.9-69.5)	28.4 (18.6-38.1)	12.0 (7.3-20.0)	17.7 (8.1-33.4)	8.0 (6.1-10.0)	1.6 (0.4-3.0)	9.1 (5.8-11.6)	5.5 (2.5-8.9)	4.4 (0.2-14.4)
Luxembourg	55.0 (46.1-64.1)	21.9 (14.0-30.5)	30.2 (18.2-51.6)	13.4 (5.2-26.4)	6.0 (3.8-8.1)	2.1 (0.6-4.1)	12.0 (8.8-14.7)	5.0 (2.5-8.2)	4.4 (0.3-13.6)
Madagascar	57.8 (47.8-66.0)	26.0 (14.6-37.3)	9.8 (6.6-14.3)	4.1 (2.6-6.5)	5.8 (4.8-7.0)	9.3 (3.7-14.5)	4.4 (1.2-6.9)	10.9 (6.3-17.2)	8.6 (0.2-25.4)
Malawi	62.3 (54.4-69.0)	24.4 (14.0-35.1)	18.4 (11.8-27.0)	6.0 (3.6-9.8)	5.9 (5.0-7.0)	7.5 (3.2-11.6)	2.2 (0.6-3.7)	8.1 (4.4-13.4)	8.6 (0.3-24.8)
Malaysia	65.0 (56.9-71.7)	34.4 (24.2-43.8)	24.2 (16.7-34.2)	11.4 (7.5-17.9)	10.9 (9.3-12.4)	4.9 (1.3-8.3)	3.7 (1.2-5.8)	6.5 (3.2-11.0)	12.6 (1.7-26.1)
Maldives	56.2 (48.1-63.1)	26.2 (16.2-36.0)	19.8 (13.2-29.4)	10.9 (7.0-16.5)	10.3 (8.7-11.9)	4.0 (1.0-7.4)	1.8 (0.5-3.0)	8.7 (4.7-13.9)	11.6 (1.6-24.1)
Mali	42.5 (33.6-50.5)	23.6 (14.2-33.3)	13.3 (8.2-19.8)	4.1 (2.5-6.5)	5.2 (4.3-6.2)	6.7 (2.5-10.6)	6.4 (2.6-9.2)	8.8 (5.0-13.8)	4.4 (0.2-16.9)
Malta	53.6 (44.4-63.3)	19.4 (11.7-28.0)	31.3 (18.2-54.3)	14.2 (5.0-28.6)	6.5 (4.2-8.6)	0.8 (0.2-1.5)	8.0 (4.9-10.3)	4.7 (2.2-7.8)	5.4 (0.3-15.1)
Marshall Islands	46.7 (37.4-56.5)	43.9 (28.3-59.4)	39.5 (24.9-55.6)	5.7 (3.9-8.2)	10.4 (8.6-12.4)	9.2 (3.4-14.7)	7.4 (2.6-11.0)	10.9 (6.1-17.9)	6.9 (0.5-17.9)
Mauritania	56.5 (48.5-63.7)	38.3 (28.2-48.2)	11.3 (7.3-17.1)	7.3 (4.6-11.5)	6.9 (5.8-8.0)	7.3 (3.2-11.4)	6.9 (3.1-9.7)	12.2 (7.4-17.6)	5.2 (0.2-19.3)
Mauritius	54.3 (44.9-62.8)	32.5 (22.1-42.3)	29.8 (20.9-40.7)	11.0 (6.4-18.6)	13.1 (11.2-15.2)	4.0 (1.1-7.1)	4.2 (1.5-6.5)	9.7 (5.8-14.8)	12.4 (1.7-26.4)
Mexico	52.0 (44.3-58.8)	39.4 (27.7-50.1)	30.6 (21.6-42.5)	11.2 (6.7-18.3)	11.6 (9.8-13.5)	4.3 (1.2-7.3)	9.5 (5.8-12.4)	5.0 (2.6-8.1)	5.5 (0.3-16.2)
Micronesia (Federated States of)	45.9 (36.5-54.7)	51.9 (37.6-64.4)	34.4 (23.4-46.9)	5.5 (3.7-8.1)	11.4 (9.5-13.8)	8.9 (3.3-14.4)	6.9 (2.3-10.4)	10.4 (5.9-17.1)	7.3 (0.6-18.9)
Monaco	53.0 (43.2-63.1)	22.0 (13.4-30.8)	20.1 (11.3-37.7)	16.8 (5.7-34.2)	5.7 (3.1-8.0)	0.3 (0.2-0.6)	11.8 (8.9-14.2)	1.3 (0.6-2.2)	3.8 (0.2-12.7)
Mongolia	69.0 (59.5-77.0)	44.7 (30.9-57.6)	7.9 (5.0-11.3)	3.6 (2.6-5.1)	9.2 (7.4-11.2)	7.1 (1.5-12.7)	20.5 (14.4-25.8)	16.0 (9.1-23.4)	8.2 (0.6-21.1)

Montenegro	61.7 (50.8-71.1)	34.3 (23.5-45.4)	31.6 (20.4-46.0)	5.0 (2.3-9.3)	8.8 (6.9-10.5)	0.3 (0.2-0.5)	9.6 (5.5-12.8)	2.2 (1.1-3.6)	17.8 (5.7-31.9)
Morocco	62.1 (53.2-69.5)	35.7 (24.3-46.6)	31.3 (19.9-48.9)	17.7 (11.4-27.5)	11.6 (9.4-13.6)	1.1 (0.3-2.4)	4.5 (1.7-6.8)	2.6 (1.2-4.5)	2.1 (0.3-8.1)
Mozambique	65.5 (56.8-72.4)	23.8 (13.6-34.6)	16.0 (10.3-23.6)	6.0 (3.7-9.5)	6.3 (5.3-7.5)	8.2 (3.3-12.7)	2.2 (0.6-3.7)	11.7 (6.8-17.3)	8.9 (0.3-25.6)
Myanmar	56.7 (48.4-64.7)	21.2 (11.9-30.9)	23.4 (16.0-33.3)	6.3 (3.7-10.7)	10.2 (8.6-11.9)	4.6 (1.1-8.3)	4.3 (1.4-6.7)	10.0 (5.8-15.3)	12.3 (1.6-25.8)
Namibia	56.4 (47.0-64.3)	31.9 (23.1-40.9)	23.7 (15.0-35.1)	6.4 (3.4-11.6)	7.6 (6.3-8.9)	6.2 (2.5-9.7)	6.9 (3.2-9.7)	10.3 (6.2-15.2)	4.2 (0.2-16.2)
Nauru	63.1 (53.4-71.1)	57.4 (42.7-69.5)	28.3 (18.0-40.4)	7.5 (5.5-9.9)	11.5 (9.4-13.9)	8.7 (3.1-14.1)	9.2 (3.8-13.3)	9.9 (5.4-16.3)	5.5 (0.4-15.7)
Nepal	47.9 (38.8-55.7)	20.2 (10.9-30.2)	19.8 (13.2-29.4)	5.5 (2.9-9.9)	7.4 (6.1-8.8)	5.2 (1.4-8.9)	4.0 (1.3-6.3)	8.6 (4.8-13.9)	6.4 (0.3-18.4)
Netherlands	52.7 (42.9-62.2)	20.1 (12.0-29.0)	19.6 (11.0-36.4)	14.7 (5.5-28.8)	6.0 (3.8-8.1)	2.9 (0.9-5.0)	10.5 (7.4-13.0)	4.4 (2.1-7.1)	3.8 (0.2-12.7)
New Zealand	48.0 (39.5-57.5)	25.6 (16.9-34.3)	17.1 (9.8-34.2)	13.8 (5.2-27.9)	6.0 (3.7-8.3)	2.3 (0.7-4.3)	12.9 (9.8-15.6)	4.6 (2.2-7.7)	3.7 (0.2-12.4)
Nicaragua	55.6 (47.2-63.2)	32.5 (21.5-43.3)	31.2 (20.9-44.2)	9.6 (5.3-16.7)	11.8 (9.9-13.7)	7.2 (3.3-10.7)	2.3 (0.7-3.8)	10.0 (6.1-14.9)	8.3 (0.6-20.8)
Niger	48.9 (40.7-56.7)	19.4 (10.7-29.1)	11.8 (7.0-18.3)	3.8 (2.4-6.0)	5.2 (4.3-6.2)	4.4 (0.9-8.1)	5.9 (2.3-8.6)	10.7 (6.2-15.8)	4.7 (0.2-17.3)
Nigeria	59.3 (52.1-65.5)	26.7 (17.2-36.6)	11.9 (7.8-17.9)	6.3 (4.0-9.6)	6.2 (5.2-7.2)	4.2 (1.1-7.5)	2.7 (0.8-4.4)	7.6 (4.2-12.1)	4.3 (0.2-16.7)
Niue	60.3 (52.3-67.2)	42.0 (30.3-53.0)	35.0 (25.2-46.9)	7.0 (4.2-11.9)	11.6 (9.8-13.6)	6.6 (2.5-10.5)	6.8 (3.0-9.8)	7.1 (3.7-11.9)	9.0 (0.7-21.8)
North Macedonia	58.0 (48.5-67.1)	31.0 (21.1-41.0)	34.3 (21.3-53.3)	13.6 (6.1-26.6)	8.8 (6.9-10.6)	0.3 (0.2-0.5)	4.5 (1.9-6.5)	3.7 (1.7-6.0)	17.2 (5.4-30.4)
Northern Ireland	52.7 (43.3-62.0)	24.6 (15.7-33.7)	24.8 (14.1-45.7)	11.6 (4.1-24.1)	4.9 (3.0-6.8)	2.4 (0.8-4.3)	7.6 (4.6-10.0)	6.0 (3.2-9.4)	3.4 (0.2-11.6)
Northern Mariana Islands	60.7 (50.3-68.8)	53.0 (39.3-64.6)	33.7 (22.9-46.6)	6.8 (4.5-10.3)	12.3 (10.2-14.8)	6.6 (1.9-11.0)	9.0 (4.2-12.8)	6.6 (3.4-11.4)	9.2 (0.8-22.5)
Norway	53.8 (45.0-62.6)	17.9 (10.7-25.4)	26.0 (14.8-47.5)	15.4 (5.8-30.0)	4.8 (2.9-6.8)	3.2 (1.1-5.2)	9.3 (6.2-11.6)	3.9 (1.9-6.3)	3.7 (0.3-11.8)

Oman	54.9 (47.3-61.5)	47.7 (36.1-57.8)	26.0 (17.7-38.5)	18.0 (12.8-25.7)	10.1 (8.3-11.7)	1.9 (0.4-4.0)	8.8 (4.9-11.9)	3.1 (1.5-5.1)	2.1 (0.2-8.2)
Pakistan	59.6 (51.6-65.9)	27.2 (15.9-38.9)	21.0 (14.8-29.0)	6.5 (4.2-10.3)	7.8 (6.5-9.1)	7.2 (2.8-11.5)	4.6 (1.5-7.1)	10.2 (6.0-15.8)	6.6 (0.4-18.0)
Palau	58.6 (48.8-66.5)	52.7 (39.2-64.3)	36.7 (24.9-49.2)	9.8 (6.8-14.3)	12.9 (10.9-15.2)	7.4 (2.8-11.7)	8.5 (4.1-12.2)	7.9 (4.1-13.2)	8.4 (0.7-20.9)
Palestine	47.2 (39.0-54.6)	32.8 (22.5-43.0)	32.8 (20.7-51.1)	17.6 (11.1-28.1)	12.3 (9.8-14.6)	1.8 (0.5-3.5)	2.5 (0.8-4.0)	5.6 (2.5-9.0)	1.9 (0.3-7.4)
Panama	53.9 (45.5-61.8)	31.1 (21.3-41.5)	30.2 (20.0-45.2)	9.2 (4.2-17.3)	9.3 (7.5-11.1)	5.8 (2.2-9.2)	7.8 (4.1-10.6)	6.5 (3.4-10.7)	8.4 (0.6-20.6)
Papua New Guinea	40.5 (31.0-50.1)	27.6 (14.4-40.9)	31.1 (20.1-43.5)	3.9 (2.5-5.8)	8.0 (6.4-9.8)	7.3 (2.0-12.7)	5.8 (1.6-9.1)	10.1 (5.5-16.3)	6.4 (0.5-17.0)
Paraguay	55.7 (46.9-63.1)	37.0 (25.6-48.2)	23.7 (15.5-36.2)	9.8 (5.2-17.4)	7.6 (6.3-8.9)	5.9 (2.0-9.5)	13.8 (9.8-17.2)	5.3 (2.6-9.0)	6.9 (0.3-18.8)
Peru	46.1 (38.8-53.5)	38.8 (26.6-49.9)	13.6 (8.5-21.4)	8.9 (5.0-15.3)	6.1 (4.8-7.3)	5.3 (1.5-8.8)	4.0 (1.4-6.2)	6.6 (3.3-11.0)	6.1 (0.3-16.7)
Philippines	49.6 (40.9-57.5)	29.0 (17.5-39.3)	16.2 (11.2-22.9)	7.5 (4.9-11.7)	11.3 (9.6-13.2)	5.6 (1.5-9.6)	7.1 (3.0-10.2)	6.8 (3.6-11.1)	12.4 (2.0-25.8)
Poland	54.4 (46.3-62.5)	33.0 (22.9-43.0)	27.8 (17.2-43.6)	14.4 (7.0-26.5)	6.9 (5.3-8.6)	0.7 (0.2-1.4)	11.2 (7.9-13.8)	6.0 (3.0-9.5)	11.5 (2.1-23.7)
Portugal	47.7 (38.7-57.1)	17.8 (10.1-26.5)	29.5 (17.5-52.3)	14.1 (4.3-29.5)	5.6 (3.3-7.9)	0.8 (0.2-1.6)	11.1 (8.1-13.6)	3.8 (1.8-6.2)	3.9 (0.3-12.0)
Puerto Rico	52.3 (43.5-60.9)	35.0 (25.2-44.1)	29.6 (20.2-43.8)	10.7 (4.6-20.7)	8.9 (6.9-10.9)	5.6 (2.5-8.7)	4.7 (2.0-7.0)	4.2 (2.0-7.0)	4.2 (0.2-13.7)
Qatar	54.8 (45.9-63.3)	69.0 (56.0-77.9)	27.1 (17.7-38.3)	16.3 (11.7-21.5)	8.1 (6.9-9.3)	0.2 (0.2-0.4)	10.3 (5.4-14.3)	2.8 (1.2-4.9)	2.8 (0.2-10.2)
Republic of Korea	41.8 (33.8-49.4)	16.2 (7.8-25.0)	22.3 (13.4-38.1)	11.9 (5.7-21.8)	6.7 (5.0-8.3)	0.9 (0.3-2.0)	8.9 (5.6-11.6)	5.2 (2.5-8.5)	13.6 (2.9-26.6)
Republic of Moldova	67.1 (58.4-74.1)	36.6 (25.6-47.0)	16.2 (10.7-24.2)	11.6 (6.6-20.2)	8.8 (7.3-10.5)	2.7 (0.8-5.0)	4.6 (1.8-6.9)	6.8 (3.4-10.8)	3.7 (0.2-13.1)
Romania	62.5 (53.5-70.6)	29.9 (21.7-38.4)	16.1 (10.0-25.3)	14.4 (6.1-28.1)	7.1 (5.5-8.6)	0.2 (0.2-0.3)	10.4 (7.2-13.0)	4.8 (2.2-8.0)	17.6 (5.5-30.7)
Russian Federation	59.5 (51.4-66.9)	33.3 (23.2-43.3)	18.0 (11.9-27.1)	17.9 (9.4-31.4)	9.5 (7.7-11.4)	2.3 (0.7-4.0)	8.3 (4.9-10.9)	6.1 (3.0-9.7)	7.4 (0.8-18.1)

Rwanda	52.2 (43.6-60.1)	22.7 (12.3-33.9)	16.2 (9.7-24.9)	2.5 (1.3-4.4)	5.9 (4.9-7.0)	7.4 (2.7-12.1)	2.4 (0.6-3.9)	0.3 (0.2-0.4)	9.1 (0.3-25.9)
Saint Kitts and Nevis	59.2 (49.4-66.3)	39.1 (26.5-51.2)	33.9 (22.9-46.6)	9.5 (5.7-15.7)	10.9 (9.1-13.0)	5.3 (1.7-8.9)	5.6 (2.3-8.2)	9.6 (5.6-14.6)	4.6 (0.2-15.4)
Saint Lucia	55.3 (46.6-63.0)	34.3 (23.6-45.0)	33.9 (23.8-46.6)	8.3 (4.4-14.8)	9.2 (7.6-10.9)	6.2 (2.5-9.5)	6.9 (3.5-9.6)	5.8 (2.8-9.8)	4.3 (0.2-14.4)
Saint Vincent and the Grenadines	52.8 (44.5-60.7)	34.5 (23.3-45.3)	34.1 (23.5-48.1)	8.1 (4.3-14.4)	9.2 (7.7-10.8)	5.8 (2.1-9.2)	4.3 (1.6-6.5)	4.4 (2.2-7.0)	4.2 (0.2-14.5)
Samoa	53.3 (43.6-61.5)	48.1 (34.8-59.4)	34.5 (23.3-46.8)	5.4 (3.5-8.5)	10.8 (9.1-12.7)	8.8 (3.6-13.7)	7.9 (3.4-11.4)	9.0 (4.9-14.8)	2.3 (0.3-8.9)
San Marino	52.1 (42.2-61.6)	20.6 (12.6-29.1)	24.5 (13.4-47.2)	15.9 (5.5-32.0)	5.2 (3.0-7.3)	1.7 (0.5-3.2)	9.8 (6.6-12.3)	3.6 (1.6-6.0)	3.8 (0.2-12.6)
Sao Tome and Principe	64.6 (56.5-70.8)	40.1 (29.2-51.0)	17.4 (12.1-25.2)	7.2 (4.8-10.9)	7.4 (6.2-8.6)	7.5 (3.1-11.6)	1.3 (0.4-2.2)	4.3 (2.2-6.9)	5.4 (0.2-20.2)
Saudi Arabia	55.2 (46.7-62.9)	61.0 (48.0-70.8)	26.1 (18.2-35.3)	17.4 (12.4-23.4)	11.9 (10.2-13.6)	3.4 (0.9-6.7)	5.0 (1.8-7.6)	7.8 (3.7-12.8)	2.4 (0.2-9.3)
Scotland	50.5 (41.7-59.6)	25.8 (16.8-34.9)	30.8 (17.5-54.9)	13.9 (5.1-28.0)	5.4 (3.4-7.4)	2.5 (0.8-4.5)	6.9 (4.1-9.2)	5.8 (3.1-9.0)	2.2 (0.2-8.7)
Senegal	59.3 (51.4-65.9)	29.8 (19.8-39.8)	20.8 (13.9-29.6)	5.8 (3.7-9.0)	6.2 (5.2-7.2)	5.7 (1.6-9.6)	3.6 (1.1-5.7)	10.8 (6.3-16.0)	5.3 (0.2-19.2)
Serbia	60.8 (51.3-70.1)	27.8 (18.2-36.7)	32.9 (18.7-56.1)	18.2 (7.8-35.1)	8.3 (6.2-10.2)	1.7 (0.5-3.2)	5.0 (2.3-7.0)	2.9 (1.3-4.8)	17.1 (5.7-30.3)
Seychelles	59.6 (51.4-67.2)	35.2 (24.4-45.7)	28.7 (19.8-40.4)	12.0 (7.7-18.7)	12.1 (10.2-14.1)	6.7 (2.5-10.4)	3.1 (1.0-5.1)	7.3 (3.8-12.0)	9.8 (1.0-22.0)
Sierra Leone	58.5 (50.6-65.8)	22.6 (12.8-32.7)	8.2 (5.3-12.2)	5.2 (3.4-8.0)	5.6 (4.7-6.6)	6.3 (2.3-10.2)	1.5 (0.4-2.6)	9.4 (5.3-14.4)	4.9 (0.2-18.1)
Singapore	41.1 (32.5-48.7)	24.5 (15.6-33.4)	20.5 (13.8-31.3)	12.8 (7.1-21.6)	8.9 (7.1-10.6)	1.2 (0.3-2.9)	10.4 (6.6-13.5)	4.2 (2.0-6.7)	11.6 (1.9-24.4)
Slovakia	59.5 (50.5-68.5)	33.0 (22.6-43.0)	19.8 (12.2-32.8)	16.8 (8.9-30.1)	8.0 (6.3-9.7)	1.9 (0.5-3.7)	8.9 (5.4-11.6)	6.1 (2.8-9.8)	17.2 (5.7-30.0)
Slovenia	57.9 (48.1-67.4)	27.0 (18.3-36.0)	21.1 (12.4-36.7)	15.2 (5.9-30.2)	6.7 (4.6-8.5)	2.5 (0.8-4.3)	9.0 (5.9-11.4)	3.5 (1.7-5.8)	17.1 (5.6-30.2)
Solomon Islands	41.9 (31.4-52.1)	43.2 (27.7-58.2)	30.5 (19.9-43.4)	4.1 (2.8-6.1)	10.5 (8.6-12.8)	10.7 (4.2-16.9)	3.6 (0.7-6.0)	11.1 (6.1-18.3)	6.6 (0.5-18.1)

Somalia	53.0 (44.2-61.5)	10.5 (3.0-21.2)	12.2 (7.6-18.6)	3.4 (2.0-5.5)	5.6 (4.5-6.9)	9.4 (3.7-14.5)	6.2 (1.9-9.4)	13.0 (7.4-19.3)	8.4 (0.3-24.0)
South Africa	63.3 (55.7-70.3)	42.2 (32.5-51.6)	27.1 (18.0-39.7)	9.7 (5.7-16.5)	8.4 (7.1-9.8)	5.4 (1.9-8.5)	8.6 (5.0-11.5)	9.9 (5.9-14.7)	3.8 (0.2-15.1)
South Sudan	55.1 (46.3-62.9)	29.8 (19.3-39.9)	12.6 (8.2-18.8)	4.7 (2.8-7.8)	5.6 (4.7-6.7)	7.0 (2.7-11.2)	3.8 (1.2-6.1)	7.0 (3.7-11.8)	8.5 (0.2-24.5)
Spain	50.4 (41.8-58.9)	21.6 (13.5-30.4)	28.3 (16.6-50.8)	12.2 (4.2-25.2)	5.0 (2.7-7.3)	1.5 (0.4-3.0)	9.7 (6.5-12.3)	2.6 (1.3-4.2)	2.7 (0.3-9.1)
Sri Lanka	56.0 (47.1-63.7)	21.6 (12.7-30.2)	32.5 (21.3-49.3)	13.6 (7.6-23.9)	10.9 (9.0-12.8)	4.8 (1.7-7.6)	0.8 (0.3-1.3)	8.2 (4.5-12.4)	12.0 (1.6-25.1)
Suriname	48.7 (40.0-56.7)	38.5 (26.1-50.1)	33.7 (23.7-45.9)	8.4 (5.0-14.0)	9.4 (7.8-11.2)	6.0 (1.8-9.8)	2.8 (0.8-4.6)	6.9 (3.4-11.8)	4.3 (0.2-14.7)
Sweden	51.9 (42.5-61.1)	19.3 (11.7-27.6)	23.5 (12.8-44.5)	15.1 (5.6-30.0)	5.9 (3.9-7.8)	2.8 (1.0-4.6)	9.2 (6.3-11.5)	4.4 (2.1-7.2)	4.1 (0.2-12.7)
Switzerland	43.2 (34.3-53.5)	17.6 (10.7-25.4)	21.1 (11.7-41.7)	15.2 (5.3-31.3)	5.5 (2.9-7.9)	2.6 (0.8-4.5)	10.7 (7.7-13.3)	4.4 (2.1-7.4)	3.8 (0.2-12.4)
Syrian Arab Republic	51.6 (43.8-58.8)	38.9 (27.2-49.8)	28.0 (18.8-41.1)	13.1 (8.7-20.1)	10.4 (8.6-12.0)	3.0 (0.8-5.6)	4.2 (1.6-6.4)	5.1 (2.4-8.5)	2.0 (0.2-8.0)
Taiwan (Province of China)	49.2 (40.4-57.2)	23.6 (13.7-33.5)	21.1 (14.1-31.6)	11.6 (6.1-19.8)	9.7 (8.0-11.3)	1.1 (0.3-2.8)	13.2 (9.3-16.4)	4.2 (2.1-6.6)	8.2 (0.6-20.6)
Tajikistan	60.2 (49.8-70.0)	26.3 (15.1-37.9)	28.9 (18.4-41.9)	3.7 (2.3-5.8)	8.3 (6.9-9.8)	0.6 (0.2-2.0)	4.1 (1.0-6.5)	9.9 (5.4-16.2)	6.8 (0.5-18.6)
Thailand	47.5 (39.1-55.0)	30.4 (20.1-40.6)	18.8 (12.3-28.3)	10.3 (6.5-16.4)	10.7 (9.0-12.3)	6.0 (1.8-9.8)	4.8 (1.7-7.3)	5.0 (2.5-8.2)	11.7 (1.7-25.4)
Timor-Leste	60.0 (51.2-66.9)	11.2 (4.3-19.8)	24.2 (16.0-34.8)	5.7 (3.2-10.2)	10.1 (8.5-11.6)	7.4 (3.1-11.3)	3.4 (1.0-5.4)	11.6 (6.9-16.9)	12.8 (1.9-26.7)
Togo	58.2 (49.9-65.5)	31.0 (20.1-41.5)	9.1 (6.4-12.9)	6.2 (4.2-9.2)	6.5 (5.4-7.5)	8.5 (3.5-13.1)	2.2 (0.6-3.7)	14.0 (8.5-19.9)	5.4 (0.2-20.0)
Tokelau	53.5 (44.3-61.8)	42.3 (29.7-54.0)	33.5 (23.2-45.2)	7.0 (4.4-11.2)	10.8 (9.2-12.7)	7.4 (3.0-11.8)	6.8 (2.8-10.0)	8.2 (4.5-13.6)	8.2 (0.7-20.5)
Tonga	56.6 (48.5-63.6)	40.6 (29.9-50.2)	30.7 (21.5-41.8)	8.6 (5.1-14.1)	10.3 (8.7-11.8)	6.9 (2.9-10.8)	5.9 (2.7-8.7)	7.9 (4.4-12.8)	8.3 (0.7-20.0)
Trinidad and Tobago	59.1 (51.0-66.6)	38.4 (27.5-49.0)	34.6 (23.8-49.2)	11.8 (6.7-20.7)	9.2 (7.6-10.8)	5.5 (2.1-8.7)	3.8 (1.4-5.9)	7.4 (4.0-11.8)	4.6 (0.2-15.1)

Tunisia	50.4 (41.8-58.3)	33.2 (22.4-43.0)	32.0 (20.1-51.0)	17.7 (10.4-29.5)	11.7 (9.1-14.2)	0.6 (0.2-1.2)	3.5 (1.2-5.4)	3.7 (1.6-6.1)	1.9 (0.3-7.5)
Turkey	53.8 (45.5-61.2)	37.9 (26.5-47.6)	21.9 (13.7-34.8)	11.6 (6.3-20.5)	10.4 (8.1-12.6)	0.2 (0.2-0.2)	3.9 (1.5-6.0)	1.3 (0.7-2.0)	1.4 (0.3-5.7)
Turkmenistan	64.6 (56.3-71.6)	48.5 (35.3-60.4)	18.8 (11.5-28.9)	11.5 (7.8-17.4)	9.4 (8.0-11.0)	0.2 (0.2-0.3)	17.0 (12.7-20.5)	6.6 (3.1-11.3)	7.3 (0.6-19.1)
Tuvalu	53.4 (44.4-61.9)	41.4 (27.4-54.1)	34.0 (23.6-45.8)	6.0 (4.0-9.3)	10.5 (9.0-12.4)	8.3 (3.2-13.3)	6.5 (2.5-9.8)	9.7 (5.4-15.7)	7.8 (0.6-19.8)
Uganda	55.5 (46.9-62.5)	25.7 (15.3-36.1)	16.4 (10.6-23.4)	3.8 (2.1-6.3)	5.6 (4.6-6.6)	7.7 (3.0-12.1)	3.6 (1.0-5.8)	3.1 (1.6-5.0)	8.3 (0.3-23.8)
Ukraine	57.8 (48.9-65.6)	34.9 (24.7-44.4)	13.9 (9.2-20.7)	17.2 (9.5-29.3)	8.7 (7.0-10.5)	0.7 (0.2-1.5)	6.9 (3.6-9.5)	7.4 (3.7-11.4)	3.5 (0.2-12.1)
United Arab Emirates	58.9 (48.4-68.8)	69.8 (57.1-79.1)	25.1 (16.7-34.8)	29.0 (21.4-37.4)	10.2 (8.8-11.7)	3.0 (0.7-6.0)	10.3 (5.0-14.4)	6.7 (3.0-11.0)	3.0 (0.2-10.8)
United Kingdom	46.7 (38.9-54.7)	24.4 (15.6-33.4)	29.8 (18.4-51.6)	13.0 (4.6-26.3)	4.8 (2.9-6.7)	2.6 (0.9-4.6)	7.2 (4.4-9.6)	5.8 (3.1-9.0)	3.2 (0.3-11.1)
United Republic of Tanzania	62.3 (54.4-68.6)	29.5 (20.3-39.0)	12.5 (7.7-20.8)	6.8 (4.0-11.6)	6.1 (5.1-7.1)	6.5 (2.4-10.4)	2.8 (0.8-4.6)	6.9 (3.5-11.6)	12.1 (0.6-29.9)
United States of America	45.0 (37.3-52.6)	34.9 (25.0-43.8)	29.6 (20.0-44.4)	10.4 (4.9-19.4)	7.3 (5.2-9.2)	2.6 (0.9-4.4)	11.2 (7.9-13.8)	5.3 (2.7-8.7)	5.1 (0.3-15.1)
United States Virgin Islands	56.2 (46.7-64.6)	40.6 (28.7-51.2)	34.1 (23.0-47.6)	8.8 (4.5-16.5)	9.6 (8.0-11.2)	3.9 (1.1-6.8)	5.3 (2.3-7.8)	3.0 (1.5-5.1)	4.5 (0.2-15.1)
Uruguay	50.3 (41.6-59.2)	26.2 (16.9-35.9)	18.4 (11.3-30.4)	13.6 (5.8-25.8)	5.9 (4.1-7.5)	4.2 (1.4-6.7)	12.8 (9.6-15.6)	5.0 (2.4-8.3)	6.8 (0.4-17.9)
Uzbekistan	61.6 (52.8-68.9)	43.1 (30.4-55.4)	27.6 (18.4-39.9)	12.5 (8.7-18.2)	10.5 (9.0-12.2)	0.2 (0.2-0.2)	11.3 (7.0-14.8)	7.0 (3.3-11.9)	6.9 (0.5-18.7)
Vanuatu	64.7 (56.3-71.7)	41.0 (27.9-54.2)	29.3 (20.1-39.8)	6.2 (4.2-9.1)	9.9 (8.3-11.7)	8.2 (3.0-13.2)	10.1 (5.0-14.0)	8.4 (4.2-14.5)	7.7 (0.6-19.2)
Venezuela (Bolivarian Republic of)	58.8 (50.0-67.0)	36.9 (24.6-48.7)	29.9 (20.1-41.9)	6.1 (3.1-11.2)	10.5 (8.8-12.2)	5.3 (1.4-9.2)	7.4 (3.2-10.5)	6.2 (3.1-10.8)	9.3 (0.7-22.3)
Viet Nam	61.2 (52.5-68.5)	15.1 (7.2-24.3)	22.1 (14.4-33.9)	7.9 (4.4-14.0)	10.1 (8.5-11.7)	2.4 (0.6-5.3)	8.8 (4.4-12.0)	7.5 (3.9-12.5)	13.5 (2.0-27.8)

Yemen	47.9 (40.0-55.6)	25.0 (15.1-34.8)	19.4 (11.6-30.2)	14.8 (10.2-21.6)	8.7 (7.3-10.2)	6.2 (2.6-9.5)	3.1 (1.0-4.9)	9.1 (5.0-13.9)	2.0 (0.2-7.6)
Zambia	46.3 (37.2-54.6)	31.1 (20.2-41.8)	14.8 (9.5-22.0)	4.5 (2.6-7.7)	6.3 (5.3-7.4)	8.1 (3.2-12.8)	3.8 (1.0-6.0)	13.9 (8.4-20.0)	8.3 (0.3-24.6)
Zimbabwe	59.2 (50.7-66.1)	28.6 (19.2-38.5)	24.5 (16.4-36.4)	8.4 (5.2-13.4)	6.8 (5.6-8.0)	7.8 (3.7-11.5)	3.6 (1.2-5.7)	11.8 (7.3-16.8)	6.2 (0.2-21.3)

Supplement Table 9b. Percent of DALYs (with 95% uncertainty intervals) due to stroke for diet low in whole grains, alcohol use, low physical activity, smoking, second-hand smoking, ambient particulate matter$_{2.5}$ pollution, household air pollution from solid fuels and low ambient temperature by location for both sexes combined in 2019, all ages

Country, region	Diet low in whole grains	Alcohol use	Low physical activity	Smoking	Second-hand smoking	Ambient particulate matter$_{2.5}$ pollution	Household air pollution from solid fuels	Low ambient temperature
GBD regions								
High-income Asia Pacific	2.0 (0.5-3.0)	5.2 (2.9-7.5)	2.6 (0.3-7.9)	15.5 (14.3-16.8)	1.9 (1.4-2.4)	12.1 (9.0-15.5)	0.0 (0.0-0.1)	7.3 (5.5-9.0)
Central Asia	4.1 (2.2-5.3)	6.0 (4.0-8.0)	1.2 (0.2-3.5)	18.5 (17.2-19.8)	3.9 (2.9-5.0)	22.1 (16.2-28.7)	4.3 (2.2-7.6)	10.3 (7.4-13.9)
East Asia	2.3 (0.6-3.4)	8.1 (5.8-10.5)	1.4 (0.2-4.1)	21.9 (19.8-24.0)	4.2 (3.2-5.4)	27.6 (23.4-30.8)	7.5 (4.1-12.2)	8.7 (6.7-11.2)
Southeast Asia	1.3 (0.2-2.1)	5.1 (3.3-6.7)	1.0 (0.1-3.2)	19.4 (17.5-21.2)	4.1 (3.0-5.3)	15.1 (11.5-18.5)	13.9 (8.7-19.8)	1.0 (0.2-2.1)
South Asia	1.6 (0.5-2.4)	3.4 (2.1-4.9)	1.1 (0.2-2.9)	13.1 (11.8-14.5)	3.7 (2.7-4.8)	23.9 (18.4-29.0)	18.2 (13.0-24.3)	2.4 (-0.2-4.7)
Southeast Asia, East Asia, and Oceania	2.0 (0.5-3.0)	7.2 (5.2-9.3)	1.3 (0.2-3.9)	21.2 (19.3-23.0)	4.2 (3.2-5.3)	24.1 (20.2-27.1)	9.4 (5.6-14.3)	6.5 (5.1-8.5)
Oceania	1.2 (0.3-1.8)	2.4 (0.5-4.7)	0.7 (0.1-2.0)	19.8 (17.2-22.5)	5.0 (3.6-6.5)	4.8 (1.5-11.0)	34.3 (28.1-39.0)	1.8 (1.0-3.0)
Australasia	2.4 (0.6-3.6)	6.6 (3.9-9.4)	3.9 (0.6-10.0)	12.4 (11.2-13.6)	1.5 (1.1-1.8)	3.0 (0.7-5.4)	0.0 (0.0-0.1)	5.4 (3.8-7.1)
Central Europe, Eastern Europe, and Central Asia	3.6 (1.1-5.1)	6.7 (4.4-8.8)	2.1 (0.4-5.8)	20.4 (19.1-21.8)	3.0 (2.3-3.8)	13.1 (10.1-16.2)	1.5 (0.7-2.9)	10.7 (7.1-15.7)
Central Europe	3.0 (0.8-4.4)	7.1 (4.6-9.4)	2.4 (0.5-6.5)	20.3 (19.0-21.7)	3.0 (2.3-3.8)	15.1 (13.1-17.3)	2.4 (0.9-5.2)	10.4 (6.8-14.3)
Eastern Europe	3.7 (1.0-5.3)	6.6 (4.3-8.9)	2.2 (0.5-6.2)	20.9 (19.3-22.6)	2.8 (2.1-3.6)	9.8 (6.2-13.7)	0.4 (0.1-1.0)	10.8 (6.5-17.4)

Western Europe	3.1 (1.2-4.3)	7.7 (5.2-10.2)	3.7 (0.6-9.4)	15.4 (14.2-16.7)	1.6 (1.2-2.0)	7.5 (5.5-9.6)	0.0 (0.0-0.1)	10.0 (6.9-13.2)
Central Latin America	1.6 (0.3-2.4)	5.0 (3.5-6.4)	1.1 (0.1-3.6)	9.8 (8.7-11.0)	2.3 (1.6-3.0)	16.1 (12.8-19.2)	5.9 (3.9-8.3)	3.2 (2.0-4.5)
Tropical Latin America	1.9 (0.4-2.8)	5.8 (4.0-7.6)	4.2 (0.9-8.6)	17.2 (15.7-19.0)	3.0 (2.3-3.9)	9.7 (7.0-12.6)	3.5 (1.7-6.1)	1.8 (0.5-3.2)
Southern Latin America	2.1 (0.5-3.2)	9.7 (6.8-12.6)	1.0 (0.1-3.2)	20.6 (18.9-22.2)	3.3 (2.4-4.2)	12.7 (9.1-16.6)	0.7 (0.3-1.6)	9.8 (7.2-13.0)
Andean Latin America	1.8 (0.4-2.6)	4.1 (2.3-5.9)	1.2 (0.1-3.6)	6.3 (5.4-7.2)	1.6 (1.2-2.2)	19.2 (14.4-24.1)	5.8 (3.2-9.2)	5.8 (4.3-7.7)
Latin America and Caribbean	1.8 (0.4-2.7)	5.5 (3.8-7.0)	2.8 (0.6-6.2)	13.6 (12.4-14.8)	2.6 (1.9-3.3)	12.6 (10.0-15.2)	5.8 (3.9-8.1)	2.4 (1.3-3.6)
High-income North America	2.4 (0.6-3.5)	6.0 (3.8-8.1)	2.0 (0.2-6.0)	19.3 (17.8-20.9)	1.6 (1.2-2.0)	4.4 (2.4-6.7)	0.0 (0.0-0.0)	7.1 (5.4-9.0)
North Africa and Middle East	5.2 (2.4-7.0)	0.5 (0.2-0.8)	4.4 (0.9-10.2)	14.8 (13.7-15.9)	4.3 (3.3-5.4)	27.1 (23.9-30.2)	4.1 (2.9-5.6)	5.3 (3.2-7.6)
Sub-Saharan Africa	1.4 (0.3-2.2)	6.1 (4.2-8.2)	0.8 (0.1-2.4)	7.8 (7.0-8.8)	2.1 (1.5-2.7)	10.8 (7.3-15.1)	29.7 (24.4-35.1)	2.0 (1.0-3.3)
Central Sub-Saharan Africa	1.3 (0.3-2.0)	5.2 (2.6-8.0)	0.9 (0.1-2.7)	7.7 (6.5-8.9)	1.6 (1.1-2.2)	9.7 (4.8-15.9)	31.6 (25.1-37.6)	1.6 (0.1-3.4)
Southern Sub-Saharan Africa	1.8 (0.3-2.7)	7.9 (5.8-10.1)	2.0 (0.3-5.3)	13.1 (11.8-14.5)	3.0 (2.1-3.9)	18.8 (15.0-22.8)	9.5 (6.4-12.7)	5.7 (4.2-7.5)
Eastern Sub-Saharan Africa	1.3 (0.3-2.0)	6.1 (3.9-8.4)	0.4 (0.1-1.5)	8.4 (7.3-9.6)	2.0 (1.4-2.6)	6.2 (3.3-10.3)	37.0 (31.4-42.3)	2.6 (1.1-4.5)
Western Sub-Saharan Africa	1.5 (0.4-2.3)	6.0 (4.1-8.0)	0.8 (0.1-2.5)	6.2 (5.4-7.0)	2.1 (1.5-2.7)	13.8 (9.2-19.3)	26.6 (20.4-33.0)	0.8 (-0.5-2.0)
Countries in alphabetical order								
Afghanistan	5.0 (2.5-6.8)	0.1 (-0.1-0.2)	2.7 (0.5-7.3)	8.8 (7.5-10.3)	5.0 (3.6-6.4)	10.5 (4.7-18.8)	33.7 (25.5-40.9)	8.8 (6.2-12.0)
Albania	2.4 (1.4-3.1)	6.5 (3.7-9.7)	0.8 (0.2-2.2)	18.2 (16.6-19.9)	3.8 (2.8-4.8)	12.8 (10.3-15.4)	6.2 (2.8-11.9)	10.3 (7.5-13.1)
Algeria	5.9 (2.7-7.9)	0.5 (0.1-0.9)	5.5 (1.1-12.2)	12.5 (11.2-14.3)	5.2 (4.0-6.6)	25.5 (18.7-32.0)	0.1 (0.0-0.2)	4.0 (0.7-7.0)

American Samoa	1.6 (0.4-2.4)	0.9 (-0.4-2.4)	1.7 (0.3-4.3)	21.9 (19.3-24.7)	4.8 (3.5-6.3)	5.1 (2.0-9.7)	4.0 (1.0-9.6)	0.2 (-0.2-0.5)
Andorra	2.7 (0.7-4.0)	7.3 (4.3-10.2)	3.6 (0.5-9.4)	18.1 (15.7-20.7)	2.1 (1.6-2.6)	5.8 (3.2-8.5)	0.0 (0.0-0.0)	10.4 (4.6-18.2)
Angola	1.5 (0.4-2.4)	11.5 (8.1-15.1)	0.9 (0.1-2.8)	11.1 (9.7-12.5)	2.5 (1.8-3.2)	13.5 (6.8-21.2)	19.6 (13.9-25.6)	2.1 (0.2-4.5)
Antigua and Barbuda	3.2 (1.6-4.2)	6.4 (4.3-8.6)	2.4 (0.4-6.1)	10.3 (9.0-11.9)	2.1 (1.5-2.7)	15.1 (5.8-25.9)	0.2 (0.1-0.6)	0.1 (-0.3-0.7)
Argentina	2.0 (0.5-2.9)	10.5 (7.3-13.9)	0.6 (0.1-2.3)	21.8 (20.0-23.5)	3.2 (2.4-4.1)	11.3 (7.3-16.1)	0.6 (0.2-1.5)	9.5 (7.0-12.6)
Armenia	5.8 (3.3-7.3)	2.5 (0.9-4.1)	2.0 (0.4-5.5)	18.4 (17.0-19.8)	4.0 (3.0-5.1)	24.6 (17.5-31.4)	0.6 (0.2-1.5)	10.7 (6.6-16.3)
Australia	2.4 (0.6-3.6)	6.8 (3.8-9.8)	4.0 (0.6-10.2)	12.2 (11.0-13.5)	1.4 (1.1-1.8)	3.1 (0.8-5.7)	0.0 (0.0-0.1)	4.3 (2.6-6.0)
Austria	2.4 (0.6-3.6)	7.2 (4.5-10.0)	3.6 (0.6-9.1)	18.7 (17.1-20.4)	2.1 (1.6-2.6)	8.3 (6.3-10.5)	0.0 (0.0-0.1)	9.7 (6.2-14.3)
Azerbaijan	3.2 (1.8-4.2)	8.8 (5.0-12.4)	0.9 (0.2-2.8)	18.9 (16.2-21.6)	4.8 (3.5-6.2)	21.1 (13.2-29.8)	1.8 (0.6-3.9)	9.1 (7.3-10.7)
Bahamas	2.4 (0.9-3.4)	6.5 (1.8-10.7)	2.1 (0.3-5.4)	10.3 (8.7-12.2)	2.6 (1.9-3.4)	14.4 (4.3-26.8)	0.3 (0.1-0.8)	0.3 (-0.4-1.4)
Bahrain	4.4 (1.5-6.3)	1.5 (0.7-2.4)	4.1 (0.7-9.9)	15.9 (13.7-18.6)	3.8 (2.9-4.9)	40.2 (35.1-44.8)	0.1 (0.0-0.3)	2.1 (-1.4-5.3)
Bangladesh	0.9 (0.2-1.6)	0.5 (0.0-1.1)	1.1 (0.2-3.1)	14.8 (12.9-16.5)	3.8 (2.8-4.9)	18.8 (12.4-25.0)	23.6 (17.1-30.8)	1.6 (-0.4-3.6)
Barbados	2.5 (0.6-3.6)	6.5 (4.5-8.4)	3.9 (0.6-8.9)	7.5 (6.6-8.6)	1.5 (1.1-1.9)	17.0 (7.5-27.5)	0.0 (0.0-0.0)	0.2 (-0.3-0.8)
Belarus	4.7 (2.0-6.5)	8.8 (5.8-11.8)	2.2 (0.5-6.0)	21.0 (19.2-22.8)	2.7 (2.0-3.5)	13.8 (10.5-17.4)	0.2 (0.0-0.4)	10.4 (6.3-16.0)
Belgium	2.4 (0.6-3.6)	9.3 (6.0-12.8)	3.8 (0.6-9.5)	17.7 (16.3-19.3)	1.6 (1.2-2.0)	8.6 (6.6-10.9)	0.0 (0.0-0.0)	10.8 (5.6-16.5)
Belize	1.8 (0.5-2.7)	8.0 (5.6-10.4)	2.0 (0.3-5.2)	12.6 (11.0-14.2)	2.6 (1.9-3.4)	17.2 (6.9-29.5)	5.4 (2.6-9.5)	0.4 (-0.1-1.2)
Benin	1.2 (0.3-1.9)	4.5 (2.2-7.2)	0.7 (0.1-2.3)	6.6 (5.4-7.8)	2.0 (1.4-2.6)	8.3 (3.8-14.6)	32.5 (25.4-38.5)	0.3 (-0.2-0.9)
Bermuda	2.9 (0.8-4.2)	7.1 (4.5-9.9)	4.2 (0.7-9.9)	12.1 (10.8-13.6)	2.0 (1.4-2.6)	4.0 (0.7-7.7)	0.4 (0.0-1.1)	2.6 (-3.8-10.8)

Bhutan	1.6 (0.3-2.5)	1.3 (0.2-2.7)	1.9 (0.4-4.8)	8.2 (6.6-10.1)	2.2 (1.6-2.9)	15.6 (9.5-21.5)	19.8 (13.6-26.7)	11.3 (8.0-15.6)
Bolivia (Plurinational State of)	1.6 (0.4-2.3)	3.5 (1.5-5.7)	0.9 (0.1-2.8)	6.7 (5.6-7.9)	1.6 (1.1-2.1)	17.4 (11.1-24.1)	10.1 (5.8-15.4)	6.0 (4.3-7.9)
Bosnia and Herzegovina	3.2 (0.7-4.8)	3.2 (1.4-5.0)	2.2 (0.4-6.6)	25.4 (23.6-27.2)	3.7 (2.7-4.7)	19.4 (16.2-22.4)	6.3 (2.5-12.4)	10.8 (6.8-15.2)
Botswana	1.9 (0.4-2.9)	7.9 (5.1-11.1)	1.4 (0.2-4.1)	15.9 (13.9-18.1)	4.1 (2.9-5.4)	18.1 (12.6-24.2)	12.0 (6.3-19.1)	2.7 (-1.1-7.1)
Brazil	1.9 (0.4-2.8)	5.7 (3.9-7.6)	4.3 (0.9-8.8)	17.2 (15.7-19.0)	3.0 (2.2-3.9)	9.7 (7.0-12.7)	3.3 (1.5-5.9)	1.8 (0.5-3.2)
Brunei Darussalam	2.0 (0.4-3.0)	-0.1 (-0.7-0.5)	1.5 (0.1-4.8)	17.6 (15.4-20.3)	2.7 (2.0-3.5)	5.6 (1.6-9.9)	0.1 (0.0-0.3)	0.1 (-0.2-0.5)
Bulgaria	3.1 (0.8-4.6)	9.0 (6.0-11.7)	2.1 (0.4-5.7)	21.3 (19.7-23.1)	3.0 (2.2-3.8)	14.7 (12.5-17.0)	3.0 (0.9-6.9)	10.9 (6.8-15.1)
Burkina Faso	1.1 (0.2-1.6)	10.9 (6.0-16.0)	0.4 (0.1-1.3)	6.4 (5.1-7.8)	2.3 (1.6-3.1)	5.5 (1.9-11.5)	36.5 (28.6-43.7)	0.9 (-0.9-2.4)
Burundi	1.3 (0.3-2.1)	10.7 (7.0-14.7)	0.3 (0.1-1.3)	7.6 (6.1-9.2)	1.6 (1.1-2.2)	4.3 (1.4-9.7)	41.4 (34.1-47.8)	3.8 (0.1-8.6)
CÃ´te d'Ivoire	1.6 (0.4-2.3)	8.8 (4.7-13.4)	0.7 (0.1-2.4)	11.4 (9.9-13.2)	3.4 (2.4-4.3)	13.1 (6.4-21.3)	28.6 (20.6-35.9)	0.2 (-0.1-0.6)
Cabo Verde	1.5 (0.3-2.3)	8.9 (5.9-12.3)	1.1 (0.2-3.3)	7.3 (6.3-8.5)	2.1 (1.5-2.7)	27.2 (19.3-34.3)	8.0 (4.5-13.1)	2.1 (-2.1-7.5)
Cambodia	0.8 (0.1-1.4)	8.6 (5.3-12.0)	0.7 (0.1-2.4)	19.6 (17.4-22.1)	4.4 (3.2-5.6)	8.1 (4.0-13.4)	31.9 (25.9-37.3)	0.1 (-0.1-0.3)
Cameroon	1.2 (0.2-1.8)	10.3 (6.9-13.9)	0.7 (0.1-2.1)	8.4 (7.2-9.8)	1.9 (1.4-2.6)	19.6 (12.3-26.8)	21.5 (14.4-28.9)	1.0 (-0.3-2.9)
Canada	2.5 (0.6-3.8)	5.4 (3.0-7.8)	3.0 (0.4-8.5)	16.8 (15.2-18.4)	1.4 (1.1-1.8)	3.5 (1.7-5.7)	0.0 (0.0-0.0)	10.3 (3.2-18.1)
Caribbean	2.0 (0.5-3.0)	6.2 (4.3-8.0)	2.5 (0.4-6.2)	13.4 (11.9-14.9)	2.2 (1.7-2.9)	11.3 (6.2-17.6)	13.2 (9.2-17.8)	0.6 (-0.1-1.5)
Central African Republic	1.1 (0.3-1.8)	4.5 (1.1-8.5)	0.6 (0.1-2.0)	8.3 (6.6-10.2)	2.0 (1.4-2.8)	6.0 (1.8-13.2)	41.1 (32.7-48.3)	0.4 (-0.1-1.3)
Chad	2.1 (1.1-2.8)	5.1 (1.2-8.8)	0.5 (0.1-1.6)	7.2 (5.6-8.8)	2.1 (1.5-2.8)	5.3 (2.0-11.4)	35.6 (27.6-43.2)	2.4 (-1.4-5.9)

Chile	2.5 (0.6-3.6)	8.4 (5.8-11.3)	1.7 (0.2-5.3)	17.9 (16.2-19.9)	3.5 (2.5-4.4)	18.3 (14.9-21.9)	1.1 (0.3-2.5)	11.3 (7.7-16.1)
China	2.3 (0.6-3.4)	8.2 (5.8-10.6)	1.4 (0.2-4.2)	22.0 (19.8-24.1)	4.2 (3.2-5.4)	28.0 (23.7-31.2)	7.1 (3.7-11.8)	8.7 (6.7-11.1)
Colombia	1.7 (0.4-2.6)	3.1 (1.9-4.3)	1.2 (0.1-3.8)	9.5 (8.3-10.9)	2.4 (1.7-3.0)	17.4 (13.4-22.1)	3.3 (1.4-6.2)	2.9 (2.0-4.0)
Comoros	1.7 (0.4-2.6)	0.7 (-0.1-1.9)	0.4 (0.1-1.8)	7.6 (6.1-9.3)	3.1 (2.2-4.1)	5.8 (2.9-9.8)	33.1 (27.8-37.9)	0.3 (-0.8-2.0)
Congo	1.6 (0.4-2.5)	7.1 (2.3-11.9)	1.2 (0.2-3.6)	7.8 (6.5-9.3)	2.2 (1.6-3.0)	20.8 (11.9-31.1)	18.4 (11.1-26.5)	0.6 (-0.5-2.2)
Cook Islands	2.0 (0.5-3.0)	8.9 (5.3-12.5)	2.1 (0.4-5.5)	18.1 (15.8-20.6)	4.5 (3.2-5.8)	4.1 (0.8-9.1)	1.0 (0.1-2.8)	0.7 (-1.5-3.6)
Costa Rica	2.3 (0.7-3.5)	3.7 (2.0-5.4)	1.1 (0.1-3.8)	11.5 (10.3-12.7)	2.6 (2.0-3.3)	14.6 (11.2-18.2)	1.7 (0.6-3.5)	2.0 (0.7-3.5)
Croatia	2.8 (0.7-4.1)	6.4 (3.4-9.3)	2.4 (0.5-6.3)	19.6 (18.0-21.3)	3.6 (2.7-4.6)	13.6 (11.3-16.1)	0.8 (0.2-1.9)	10.0 (5.5-14.5)
Cuba	2.5 (0.6-3.7)	5.0 (3.2-6.7)	3.7 (0.5-9.1)	21.8 (20.0-23.8)	2.7 (2.0-3.4)	14.2 (7.3-23.3)	0.5 (0.2-1.1)	0.4 (-0.2-1.3)
Cyprus	2.9 (0.9-4.1)	7.2 (4.6-9.8)	2.8 (0.3-8.1)	19.3 (17.6-21.0)	2.5 (1.9-3.1)	11.6 (8.9-14.6)	0.0 (0.0-0.1)	4.9 (0.8-9.5)
Czechia	2.7 (0.5-4.0)	8.1 (5.0-11.1)	3.0 (0.6-7.8)	19.1 (17.6-20.9)	2.4 (1.8-3.0)	12.6 (10.4-15.0)	0.2 (0.0-0.4)	9.8 (6.0-14.4)
Democratic People's Republic of Korea	1.6 (0.3-2.4)	6.5 (4.0-9.0)	0.9 (0.1-3.0)	20.6 (18.2-23.0)	4.7 (3.4-6.0)	17.6 (11.0-24.4)	24.9 (17.4-32.0)	10.8 (6.9-16.0)
Democratic Republic of the Congo	1.2 (0.3-1.9)	2.9 (0.2-6.3)	0.9 (0.1-2.6)	6.5 (5.2-7.9)	1.2 (0.8-1.7)	7.4 (3.1-13.7)	36.6 (29.4-42.7)	1.7 (0.2-3.6)
Denmark	2.7 (0.7-3.9)	7.5 (4.2-10.9)	3.1 (0.4-8.6)	21.8 (20.0-23.7)	1.6 (1.2-2.1)	6.2 (3.9-8.8)	0.0 (0.0-0.0)	10.9 (4.8-17.9)
Djibouti	2.1 (0.5-3.2)	0.2 (-0.7-1.4)	0.5 (0.1-2.0)	15.1 (12.2-18.0)	3.8 (2.7-5.0)	24.7 (13.0-36.1)	10.3 (5.2-17.2)	1.4 (-1.4-3.8)
Dominica	4.5 (2.6-5.7)	6.7 (4.4-8.9)	3.1 (0.5-7.8)	8.5 (7.4-9.8)	1.8 (1.3-2.3)	14.7 (6.3-25.2)	1.9 (0.6-4.3)	0.4 (-0.8-2.2)
Dominican Republic	1.8 (0.4-2.7)	7.4 (5.0-10.0)	2.5 (0.4-6.1)	16.3 (14.3-18.4)	2.4 (1.8-3.1)	15.1 (7.1-25.5)	3.7 (1.6-7.0)	1.2 (0.0-2.6)

Ecuador	1.9 (0.5-2.9)	3.5 (2.1-5.0)	1.3 (0.1-3.7)	8.2 (7.0-9.4)	1.6 (1.1-2.2)	17.1 (12.1-22.3)	1.9 (0.8-4.0)	4.3 (3.1-5.7)
Egypt	3.3 (0.8-5.0)	0.2 (-0.1-0.5)	3.7 (0.7-9.4)	13.4 (11.6-16.5)	4.1 (3.0-5.3)	34.2 (28.7-39.1)	0.0 (0.0-0.1)	3.5 (-0.1-7.7)
El Salvador	1.7 (0.4-2.5)	3.2 (1.9-4.7)	0.9 (0.1-3.2)	7.4 (6.4-8.7)	1.6 (1.2-2.1)	16.3 (10.7-22.7)	5.3 (2.9-8.5)	0.2 (-0.1-0.7)
Equatorial Guinea	1.6 (0.3-2.6)	9.2 (5.3-13.3)	1.5 (0.2-4.5)	7.4 (5.7-9.3)	2.1 (1.5-2.8)	27.5 (17.8-36.9)	5.6 (2.3-10.7)	0.8 (-1.2-3.8)
Eritrea	1.1 (0.2-1.8)	2.5 (0.2-5.0)	0.3 (0.1-1.3)	7.1 (5.4-8.9)	2.1 (1.5-2.9)	11.6 (5.3-20.7)	31.3 (23.3-38.4)	1.4 (0.1-2.7)
Estonia	3.2 (0.8-4.7)	6.3 (3.4-9.4)	2.5 (0.5-6.6)	20.2 (18.5-22.1)	2.1 (1.6-2.7)	3.1 (1.2-5.1)	0.8 (0.1-2.4)	9.7 (4.9-17.0)
Eswatini	1.3 (0.2-2.1)	9.0 (6.3-12.0)	1.2 (0.2-3.7)	6.7 (5.4-8.1)	1.8 (1.2-2.4)	14.3 (8.9-20.0)	19.5 (12.4-27.3)	5.0 (2.0-8.7)
Ethiopia	1.3 (0.3-2.1)	4.5 (1.6-8.0)	0.3 (0.1-1.4)	3.4 (2.8-4.0)	1.1 (0.7-1.5)	6.0 (3.2-10.3)	36.7 (30.4-42.6)	3.6 (2.4-5.1)
Fiji	2.1 (0.5-3.1)	3.6 (2.1-5.2)	1.5 (0.2-4.3)	16.3 (14.3-18.3)	4.0 (2.8-5.3)	9.4 (3.0-20.3)	9.1 (3.9-16.3)	0.7 (-0.5-2.2)
Finland	2.7 (0.7-4.0)	4.1 (1.7-6.8)	3.5 (0.5-9.7)	14.0 (12.7-15.4)	1.2 (0.9-1.5)	1.8 (0.4-3.7)	0.0 (0.0-0.0)	10.9 (5.0-19.3)
France	3.0 (0.9-4.2)	9.2 (6.0-12.4)	4.5 (0.7-10.5)	14.6 (13.2-16.3)	1.3 (1.0-1.7)	7.5 (5.5-9.7)	0.0 (0.0-0.1)	10.0 (6.2-14.0)
Gabon	1.6 (0.3-2.5)	11.9 (7.6-16.4)	1.4 (0.2-4.0)	9.0 (7.4-10.7)	2.2 (1.5-2.9)	27.2 (17.5-37.0)	2.2 (0.9-4.3)	0.5 (-0.4-1.9)
Gambia	1.5 (0.3-2.2)	5.0 (2.4-8.0)	0.8 (0.1-2.6)	9.7 (8.2-11.2)	3.5 (2.6-4.5)	11.0 (5.4-18.3)	32.0 (24.2-39.0)	0.5 (-0.3-1.2)
Georgia	3.7 (2.2-4.7)	6.4 (4.1-8.7)	1.4 (0.3-3.7)	18.9 (17.5-20.4)	4.0 (3.0-5.0)	13.5 (9.9-17.6)	6.9 (2.8-12.9)	11.2 (7.5-16.3)
Germany	2.6 (0.6-4.0)	10.3 (6.9-13.7)	3.1 (0.4-9.4)	16.1 (14.5-17.7)	1.4 (1.0-1.7)	8.0 (5.9-10.2)	0.0 (0.0-0.0)	10.1 (5.5-15.1)
Ghana	2.1 (0.5-3.2)	6.3 (3.4-9.5)	1.1 (0.2-3.5)	4.8 (4.0-5.7)	1.6 (1.1-2.2)	22.2 (14.4-29.9)	19.6 (12.8-27.0)	0.2 (-0.1-0.5)
Greece	4.5 (2.7-5.6)	6.4 (4.0-8.9)	2.7 (0.3-8.3)	17.8 (16.3-19.5)	2.2 (1.7-2.8)	9.4 (7.2-11.7)	0.1 (0.0-0.2)	9.1 (6.9-11.2)

Greenland	1.9 (0.4-2.8)	7.2 (3.3-11.5)	1.4 (0.2-4.5)	31.5 (28.5-34.8)	4.0 (2.8-5.3)	3.8 (0.4-10.6)	0.2 (0.0-0.7)	14.5 (1.6-26.5)
Grenada	3.2 (1.3-4.5)	8.4 (6.2-10.7)	2.5 (0.3-6.5)	10.2 (8.9-11.7)	2.1 (1.4-2.8)	18.1 (7.2-30.7)	0.8 (0.3-1.7)	0.2 (-0.3-0.8)
Guam	2.3 (0.6-3.4)	2.4 (-0.5-6.7)	1.7 (0.2-4.7)	18.8 (16.1-21.7)	4.7 (3.4-6.1)	6.5 (2.8-11.0)	1.0 (0.2-2.9)	0.1 (-0.1-0.4)
Guatemala	1.4 (0.3-2.0)	2.8 (1.6-4.0)	0.3 (0.1-1.6)	7.2 (5.7-8.7)	1.9 (1.4-2.6)	13.7 (8.3-19.2)	19.1 (13.0-26.1)	2.4 (1.4-3.6)
Guinea	1.1 (0.2-1.7)	2.1 (0.8-3.7)	0.6 (0.1-1.9)	10.5 (8.7-12.3)	2.4 (1.8-3.2)	6.6 (2.8-12.7)	34.8 (27.6-41.4)	0.4 (-0.1-1.2)
Guinea-Bissau	1.1 (0.2-1.8)	6.5 (3.6-9.8)	0.6 (0.1-1.9)	5.0 (4.1-5.9)	2.8 (2.0-3.7)	8.4 (3.6-15.6)	36.9 (28.4-43.8)	0.4 (-0.3-1.1)
Guyana	1.8 (0.4-2.7)	8.2 (5.5-10.8)	1.9 (0.3-5.2)	11.8 (10.3-13.5)	2.8 (2.0-3.6)	17.4 (7.3-30.0)	2.3 (1.1-4.3)	0.6 (-0.2-1.6)
Haiti	1.6 (0.4-2.5)	6.3 (4.2-8.6)	1.3 (0.2-3.8)	6.0 (5.0-7.2)	1.7 (1.2-2.3)	5.5 (2.1-10.8)	36.1 (30.0-41.1)	0.5 (-0.4-1.8)
Honduras	1.6 (0.4-2.5)	3.2 (2.2-4.4)	0.6 (0.1-2.4)	10.6 (9.2-12.2)	3.0 (2.2-3.9)	10.3 (5.8-15.7)	22.6 (16.8-28.1)	1.6 (-0.4-4.2)
Hungary	3.5 (0.8-5.1)	7.0 (4.3-9.7)	2.1 (0.4-6.0)	21.5 (20.0-23.3)	3.4 (2.5-4.4)	13.0 (10.7-15.2)	2.5 (0.7-6.0)	9.7 (4.1-15.3)
Iceland	2.7 (0.7-4.0)	5.2 (2.5-7.9)	3.9 (0.5-10.1)	16.1 (14.4-17.9)	1.8 (1.3-2.2)	2.0 (0.4-4.1)	0.0 (0.0-0.0)	12.2 (5.6-20.3)
India	1.5 (0.4-2.3)	4.4 (2.7-6.3)	1.1 (0.2-2.8)	12.6 (11.1-14.2)	3.7 (2.7-4.7)	25.5 (20.2-30.3)	16.6 (11.7-22.5)	2.0 (-0.4-4.2)
Indonesia	1.3 (0.2-2.2)	0.2 (-0.4-1.1)	1.2 (0.1-3.8)	19.0 (16.2-21.8)	4.4 (3.2-5.8)	15.2 (11.5-19.2)	11.2 (6.0-18.2)	0.7 (0.0-1.7)
Iran (Islamic Republic of)	7.7 (4.2-9.8)	0.6 (0.4-0.9)	4.7 (0.9-12.0)	12.9 (11.8-14.1)	3.8 (2.8-4.7)	27.5 (24.3-30.9)	0.1 (0.0-0.1)	7.5 (5.6-9.3)
Iraq	4.2 (1.6-5.8)	0.6 (0.1-1.2)	4.2 (0.8-9.8)	19.1 (17.2-21.2)	5.0 (3.7-6.3)	33.2 (27.8-38.2)	0.1 (0.0-0.2)	4.5 (1.2-7.9)
Ireland	2.0 (0.4-3.1)	6.5 (4.0-8.9)	4.8 (0.8-10.7)	17.2 (15.6-18.8)	1.7 (1.3-2.2)	4.3 (2.2-6.6)	0.0 (0.0-0.1)	11.4 (4.8-18.6)
Israel	3.5 (1.6-4.8)	1.7 (0.1-3.4)	3.3 (0.5-8.4)	15.7 (14.3-17.2)	1.8 (1.3-2.3)	14.7 (12.4-17.2)	0.0 (0.0-0.1)	4.1 (0.2-8.7)

Italy	4.8 (2.9-6.1)	7.5 (5.1-10.0)	4.2 (0.6-10.2)	12.9 (11.7-14.2)	1.8 (1.4-2.3)	10.2 (8.2-12.3)	0.1 (0.0-0.1)	9.4 (7.1-11.7)
Jamaica	2.1 (0.6-3.0)	4.5 (2.7-6.3)	3.2 (0.5-7.2)	12.7 (11.4-14.1)	2.5 (1.8-3.1)	12.5 (8.2-17.8)	2.7 (1.0-5.6)	0.1 (-0.3-0.9)
Japan	2.1 (0.5-3.1)	4.1 (1.7-6.4)	2.6 (0.3-7.9)	15.1 (13.8-16.6)	1.8 (1.4-2.3)	9.3 (6.3-12.6)	0.0 (0.0-0.1)	7.0 (5.5-8.6)
Jordan	7.1 (3.9-9.1)	0.4 (-0.1-0.9)	4.2 (0.8-10.4)	20.8 (18.7-22.9)	4.4 (3.3-5.6)	25.0 (21.1-28.7)	0.0 (0.0-0.0)	4.8 (1.2-8.9)
Kazakhstan	5.1 (2.8-6.5)	5.5 (2.9-8.0)	1.9 (0.3-5.3)	19.6 (17.8-21.4)	3.5 (2.6-4.6)	17.4 (12.1-23.9)	2.5 (0.9-5.3)	10.1 (6.8-14.8)
Kenya	1.3 (0.3-2.1)	7.4 (4.3-10.8)	0.4 (0.1-1.5)	8.6 (7.2-10.1)	1.9 (1.3-2.5)	8.9 (5.3-13.4)	29.7 (23.7-35.2)	3.5 (1.9-5.3)
Kiribati	1.3 (0.3-2.0)	2.4 (-0.1-5.7)	0.7 (0.1-2.1)	34.2 (31.5-36.7)	6.3 (4.6-8.2)	4.6 (1.4-11.0)	32.2 (25.3-38.1)	0.0 (0.0-0.0)
Kuwait	4.2 (1.0-6.3)	0.0 (-0.1-0.2)	5.8 (1.1-12.9)	19.8 (17.8-21.6)	4.6 (3.5-5.8)	39.5 (35.6-43.3)	0.0 (0.0-0.0)	3.2 (-2.7-8.5)
Kyrgyzstan	4.8 (2.6-6.2)	6.5 (4.5-8.7)	1.2 (0.2-3.8)	24.5 (22.7-26.6)	4.7 (3.4-6.0)	18.4 (11.7-26.2)	9.9 (5.3-15.4)	13.2 (7.0-21.6)
Lao People's Democratic Republic	1.0 (0.1-1.6)	8.3 (4.2-12.7)	0.7 (0.1-2.3)	19.2 (16.7-21.6)	4.5 (3.3-5.8)	7.9 (3.9-12.6)	35.3 (28.9-40.7)	1.8 (-0.2-4.2)
Latvia	3.7 (1.0-5.4)	4.2 (1.3-6.9)	4.2 (1.0-10.1)	15.9 (14.0-17.9)	2.4 (1.8-3.2)	8.6 (6.1-11.4)	0.8 (0.2-2.1)	10.5 (6.2-16.9)
Lebanon	7.6 (4.1-9.7)	0.7 (0.2-1.2)	6.5 (1.4-14.4)	25.9 (23.4-28.8)	4.3 (3.2-5.4)	22.4 (17.7-27.0)	0.1 (0.0-0.2)	6.5 (4.4-8.7)
Lesotho	0.9 (0.1-1.5)	7.7 (3.7-11.6)	0.8 (0.1-2.3)	15.3 (12.7-17.5)	3.8 (2.7-4.9)	12.9 (7.3-19.5)	23.9 (17.1-30.4)	11.4 (7.2-15.8)
Liberia	1.4 (0.3-2.2)	7.3 (4.1-10.6)	0.8 (0.1-2.6)	6.8 (5.6-8.1)	2.0 (1.4-2.6)	8.9 (4.2-15.7)	34.7 (27.2-41.1)	0.3 (-0.5-1.6)
Libya	6.3 (2.8-8.6)	0.4 (0.0-0.6)	5.2 (1.0-12.1)	12.5 (10.6-14.9)	5.7 (4.4-7.1)	29.3 (22.0-35.9)	0.1 (0.0-0.2)	3.4 (-0.7-7.9)
Lithuania	3.5 (0.9-5.2)	5.8 (2.8-8.9)	4.1 (0.9-9.9)	14.9 (13.6-16.4)	2.1 (1.6-2.7)	7.3 (4.8-10.0)	0.4 (0.1-1.0)	10.2 (5.4-16.2)
Luxembourg	2.4 (0.6-3.5)	9.5 (6.1-12.8)	3.1 (0.4-8.6)	17.5 (15.1-20.0)	1.9 (1.4-2.4)	6.5 (4.4-8.8)	0.0 (0.0-0.0)	10.5 (4.6-17.1)

Madagascar	1.0 (0.2-1.6)	3.0 (0.7-5.9)	0.3 (0.1-1.3)	7.3 (5.9-8.7)	2.5 (1.7-3.5)	4.5 (2.1-8.2)	41.1 (35.8-45.9)	2.7 (1.0-4.7)
Malawi	1.2 (0.2-2.0)	4.8 (2.2-7.6)	0.5 (0.1-2.0)	9.9 (8.0-12.0)	1.9 (1.3-2.5)	4.4 (1.9-8.5)	38.3 (32.8-43.3)	2.8 (0.4-5.8)
Malaysia	2.0 (0.4-3.0)	2.2 (0.8-3.7)	1.8 (0.2-5.1)	16.2 (14.6-17.7)	4.1 (3.0-5.3)	15.2 (11.1-19.3)	0.2 (0.1-0.4)	0.4 (-0.2-1.2)
Maldives	2.3 (0.5-3.5)	1.2 (-0.8-4.0)	1.7 (0.2-4.9)	20.0 (18.1-21.7)	4.4 (3.3-5.5)	9.4 (6.3-13.0)	4.3 (1.8-8.2)	0.0 (0.0-0.0)
Mali	1.1 (0.3-1.7)	2.0 (0.9-3.1)	0.5 (0.1-1.6)	5.7 (4.5-6.9)	2.1 (1.5-2.8)	5.3 (1.8-11.2)	34.5 (26.4-42.3)	2.9 (-4.3-9.1)
Malta	4.8 (2.7-6.1)	6.0 (3.6-8.6)	5.9 (1.1-12.2)	15.5 (13.9-17.3)	1.7 (1.3-2.2)	9.1 (6.8-11.6)	0.0 (0.0-0.1)	5.1 (-2.1-13.0)
Marshall Islands	1.3 (0.3-2.0)	4.0 (1.1-7.8)	0.9 (0.1-2.5)	17.9 (15.1-20.9)	5.0 (3.6-6.6)	7.4 (2.8-15.0)	14.9 (9.1-21.7)	0.0 (0.0-0.0)
Mauritania	2.1 (0.5-3.1)	0.0 (0.0-0.0)	2.3 (0.4-5.7)	8.8 (7.3-10.6)	2.3 (1.6-3.1)	21.6 (13.2-30.0)	17.9 (10.9-25.3)	3.0 (-2.8-8.3)
Mauritius	2.2 (0.5-3.3)	5.0 (2.2-8.0)	2.0 (0.3-5.7)	15.2 (13.7-16.7)	3.9 (2.8-5.0)	13.3 (7.1-20.2)	0.3 (0.1-0.7)	1.2 (-2.1-6.2)
Mexico	1.7 (0.3-2.6)	5.7 (3.9-7.5)	1.5 (0.2-4.9)	9.3 (8.0-10.8)	2.0 (1.3-2.7)	16.0 (12.6-19.6)	4.6 (2.7-7.4)	4.8 (3.3-6.5)
Micronesia (Federated States of)	1.3 (0.3-2.0)	4.2 (2.1-6.5)	1.0 (0.2-2.7)	27.1 (23.4-31.2)	4.7 (3.3-6.4)	8.7 (2.9-18.7)	13.3 (6.9-21.1)	0.0 (0.0-0.0)
Monaco	3.0 (0.7-4.4)	2.6 (-2.8-9.0)	5.0 (0.7-12.2)	15.1 (12.8-17.7)	1.6 (1.2-2.1)	7.5 (4.5-10.7)	0.0 (0.0-0.0)	8.3 (6.5-10.3)
Mongolia	1.2 (0.6-1.7)	11.9 (7.8-16.1)	0.2 (0.0-0.9)	23.5 (21.3-25.7)	4.5 (3.1-5.9)	26.1 (19.0-32.4)	11.8 (5.8-19.5)	11.9 (5.3-20.9)
Montenegro	1.1 (0.3-1.6)	12.4 (8.4-16.6)	0.7 (0.1-2.0)	28.5 (26.0-31.0)	4.2 (3.1-5.3)	15.7 (12.8-18.9)	4.8 (1.6-10.0)	11.3 (8.2-15.2)
Morocco	6.8 (3.6-8.7)	0.3 (0.0-0.7)	5.0 (1.0-11.7)	9.7 (8.4-11.1)	3.7 (2.7-4.8)	26.3 (21.7-30.8)	1.7 (0.7-3.2)	6.3 (4.2-8.9)
Mozambique	1.6 (0.4-2.5)	2.7 (-0.1-5.9)	0.4 (0.1-1.7)	10.5 (8.7-12.6)	2.0 (1.3-2.7)	3.5 (1.4-7.2)	42.1 (36.5-47.5)	1.7 (-0.1-4.1)
Myanmar	1.2 (0.2-1.9)	5.2 (2.8-7.6)	0.9 (0.1-2.7)	16.2 (13.9-18.4)	3.5 (2.5-4.6)	13.3 (8.3-18.3)	26.5 (19.4-32.6)	2.8 (1.2-4.4)

Namibia	1.8 (0.4-2.8)	8.6 (4.5-12.9)	1.9 (0.3-5.3)	10.9 (9.5-12.5)	2.4 (1.6-3.2)	15.6 (9.8-21.9)	14.9 (8.6-22.6)	3.2 (0.3-6.5)
Nauru	1.7 (0.4-2.5)	7.9 (4.6-11.6)	0.8 (0.1-2.5)	26.2 (22.2-30.2)	5.6 (3.9-7.5)	4.5 (1.3-9.8)	1.5 (0.3-3.8)	0.1 (0.0-0.2)
Nepal	1.4 (0.3-2.2)	3.7 (0.8-7.1)	1.1 (0.2-3.1)	18.6 (16.6-20.6)	3.0 (2.1-4.0)	20.0 (12.4-27.3)	23.2 (15.6-31.5)	9.0 (6.2-12.4)
Netherlands	2.7 (0.7-3.9)	6.9 (4.1-10.2)	2.1 (0.2-6.5)	17.5 (15.8-19.3)	1.6 (1.2-2.0)	8.3 (6.1-10.7)	0.0 (0.0-0.0)	10.6 (4.6-17.1)
New Zealand	2.4 (0.6-3.6)	6.0 (3.2-8.8)	3.6 (0.5-9.5)	13.3 (11.9-14.9)	1.6 (1.1-2.0)	2.4 (0.4-4.8)	0.0 (0.0-0.1)	10.5 (7.4-13.9)
Nicaragua	1.8 (0.4-2.8)	3.8 (2.6-5.2)	0.8 (0.1-3.1)	8.8 (7.5-10.1)	2.6 (1.9-3.4)	10.9 (6.2-16.8)	18.9 (12.4-24.8)	0.5 (-0.3-1.4)
Niger	1.1 (0.3-1.8)	0.4 (-0.2-1.2)	0.5 (0.1-1.7)	4.2 (3.4-5.1)	2.0 (1.4-2.7)	4.5 (1.2-11.5)	37.5 (27.3-47.5)	2.7 (-2.9-7.8)
Nigeria	1.6 (0.4-2.4)	6.5 (4.0-9.4)	0.9 (0.1-2.8)	4.4 (3.6-5.3)	1.8 (1.3-2.3)	16.8 (11.0-23.2)	21.8 (15.5-28.5)	0.4 (-0.4-1.3)
Niue	1.8 (0.4-2.7)	5.3 (0.5-9.4)	1.7 (0.3-4.5)	16.3 (13.9-18.9)	5.0 (3.6-6.4)	4.6 (1.0-10.3)	1.0 (0.2-2.5)	0.3 (-0.8-1.9)
North Macedonia	3.5 (1.1-5.0)	5.4 (3.1-7.5)	2.0 (0.4-5.5)	24.3 (22.4-26.2)	4.2 (3.1-5.3)	21.1 (18.1-24.1)	4.2 (1.6-8.4)	11.1 (7.1-15.4)
Northern Ireland	2.5 (0.6-3.8)	4.4 (-0.2-9.3)	5.8 (1.2-11.8)	16.8 (15.2-18.6)	2.0 (1.4-2.6)	4.7 (2.2-7.3)	0.0 (0.0-0.0)	12.0 (5.6-18.5)
Northern Mariana Islands	1.5 (0.4-2.3)	2.8 (-0.4-8.0)	1.2 (0.2-3.4)	21.9 (19.0-25.0)	4.5 (3.1-6.1)	7.3 (3.7-11.4)	3.7 (0.9-8.9)	0.2 (-0.1-0.6)
Norway	3.2 (1.0-4.5)	3.4 (0.8-6.0)	3.6 (0.5-9.7)	13.0 (11.8-14.3)	1.4 (1.1-1.8)	2.8 (1.1-4.8)	0.0 (0.0-0.0)	9.9 (5.2-16.3)
Oman	3.7 (0.9-5.5)	0.2 (-0.2-0.6)	4.2 (0.7-10.0)	8.9 (7.7-10.4)	3.1 (2.3-3.9)	31.8 (25.3-38.0)	0.1 (0.0-0.3)	2.6 (-3.8-8.2)
Pakistan	2.8 (1.4-3.8)	1.1 (0.6-1.6)	1.2 (0.2-3.2)	13.5 (11.6-15.7)	4.0 (2.9-5.1)	20.7 (14.5-27.0)	20.8 (14.4-27.6)	4.6 (0.1-8.9)
Palau	2.2 (0.5-3.3)	4.4 (0.4-9.5)	1.5 (0.2-4.3)	20.0 (17.1-23.2)	5.4 (3.8-7.0)	4.6 (0.0-11.3)	0.0 (0.0-0.0)	0.0 (-0.1-0.0)
Palestine	6.3 (3.2-8.2)	1.4 (0.9-1.9)	4.8 (1.0-11.2)	15.9 (14.4-18.0)	4.8 (3.6-6.0)	24.4 (18.8-29.5)	0.4 (0.2-0.8)	4.6 (0.4-9.3)
Panama	1.5 (0.3-2.3)	6.3 (4.4-8.2)	0.9 (0.1-3.2)	7.6 (6.5-8.7)	1.6 (1.2-2.1)	10.6 (6.7-14.5)	2.5 (1.0-5.0)	0.7 (0.0-1.6)

Papua New Guinea	1.0 (0.2-1.6)	2.2 (0.0-4.9)	0.5 (0.1-1.6)	19.2 (15.9-22.6)	5.0 (3.6-6.6)	4.2 (1.1-11.2)	37.8 (31.1-42.9)	2.5 (1.4-3.9)
Paraguay	1.8 (0.4-2.6)	8.5 (5.7-11.2)	1.4 (0.1-4.3)	17.5 (15.2-20.0)	3.2 (2.4-4.1)	9.9 (6.5-14.1)	11.4 (5.9-18.1)	2.1 (-0.2-4.9)
Peru	1.8 (0.4-2.6)	5.0 (2.6-7.7)	1.3 (0.1-3.9)	4.7 (3.8-5.6)	1.7 (1.2-2.3)	21.8 (16.3-27.2)	5.9 (3.0-10.0)	6.8 (4.8-9.2)
Philippines	1.2 (0.2-1.9)	10.6 (7.0-14.0)	0.5 (0.1-1.9)	22.4 (19.8-25.0)	4.6 (3.3-5.9)	13.9 (10.3-17.4)	18.1 (11.6-24.6)	0.2 (0.0-0.5)
Poland	3.1 (0.8-4.6)	7.0 (4.4-9.6)	2.8 (0.6-7.3)	21.6 (19.7-23.6)	2.6 (1.9-3.2)	17.6 (15.4-20.0)	1.8 (0.5-4.2)	10.0 (5.4-15.3)
Portugal	2.5 (0.6-3.8)	9.0 (6.5-11.4)	4.4 (0.6-10.8)	10.8 (9.8-12.0)	1.9 (1.4-2.4)	4.5 (2.6-6.5)	0.1 (0.0-0.2)	9.1 (6.8-11.2)
Puerto Rico	2.3 (0.6-3.5)	4.9 (3.4-6.4)	3.6 (0.6-8.6)	10.9 (9.2-12.8)	1.7 (1.2-2.1)	3.4 (0.8-6.2)	0.0 (0.0-0.0)	0.3 (-0.5-1.7)
Qatar	4.2 (1.1-6.2)	1.0 (0.2-1.8)	3.4 (0.4-9.4)	16.4 (14.4-18.6)	4.5 (3.4-5.8)	46.8 (42.1-50.8)	0.0 (0.0-0.0)	2.5 (-3.2-7.6)
Republic of Korea	2.0 (0.4-3.0)	9.1 (6.1-12.3)	2.5 (0.3-7.5)	16.9 (15.4-18.6)	2.1 (1.6-2.7)	21.3 (17.6-25.1)	0.0 (0.0-0.0)	8.6 (5.5-11.8)
Republic of Moldova	2.7 (0.7-4.1)	9.2 (5.8-12.7)	1.7 (0.3-5.0)	19.5 (17.6-21.7)	2.5 (1.9-3.2)	11.6 (6.8-16.6)	1.6 (0.6-3.3)	10.7 (4.7-17.4)
Romania	2.6 (0.7-3.9)	8.0 (5.1-10.8)	2.7 (0.6-6.8)	16.7 (15.3-18.1)	3.0 (2.2-3.8)	12.0 (9.8-14.2)	2.0 (0.6-4.8)	10.7 (6.9-14.8)
Russian Federation	3.5 (0.9-5.2)	6.7 (4.2-9.0)	2.4 (0.5-6.4)	21.1 (19.2-23.2)	2.8 (2.1-3.6)	8.9 (5.1-12.8)	0.3 (0.1-0.8)	11.2 (5.8-19.1)
Rwanda	1.3 (0.3-2.0)	11.8 (7.0-16.8)	0.3 (0.1-1.4)	13.8 (11.3-16.4)	2.1 (1.4-2.7)	8.8 (3.7-15.9)	35.2 (27.1-41.4)	4.9 (1.7-9.0)
Saint Kitts and Nevis	2.5 (0.7-3.6)	3.7 (-0.4-10.3)	2.5 (0.4-6.3)	8.8 (7.5-10.1)	2.5 (1.7-3.3)	6.8 (3.1-11.0)	0.5 (0.1-1.4)	0.3 (-0.2-1.0)
Saint Lucia	2.9 (1.1-4.1)	8.9 (6.3-11.5)	2.9 (0.4-7.3)	11.7 (10.4-13.1)	1.9 (1.4-2.5)	17.5 (7.8-29.0)	1.0 (0.4-2.0)	0.2 (-0.3-0.8)
Saint Vincent and the Grenadines	2.4 (0.7-3.4)	8.6 (6.0-11.1)	2.7 (0.4-6.8)	10.0 (8.3-11.6)	2.2 (1.6-2.8)	17.4 (6.8-29.5)	1.2 (0.5-2.4)	0.2 (-0.3-0.8)
Samoa	1.6 (0.4-2.5)	3.9 (1.5-6.8)	1.3 (0.2-3.5)	24.6 (22.1-27.3)	5.8 (4.4-7.5)	7.2 (2.1-15.8)	24.7 (16.4-31.8)	0.1 (-0.4-1.1)
San Marino	3.0 (0.8-4.3)	6.4 (-1.9-11.6)	4.4 (0.6-11.2)	13.7 (11.6-16.3)	1.7 (1.3-2.1)	6.0 (2.5-9.7)	0.0 (0.0-0.1)	8.5 (6.6-10.5)

Sao Tome and Principe	2.1 (0.5-3.1)	8.9 (5.8-12.6)	0.9 (0.1-3.0)	5.2 (4.2-6.2)	1.3 (0.9-1.8)	13.8 (7.3-21.8)	23.1 (16.6-29.4)	0.2 (-0.5-1.5)
Saudi Arabia	4.0 (0.9-5.9)	0.3 (-0.1-1.0)	6.2 (1.2-12.8)	15.5 (13.3-18.0)	5.3 (4.0-6.8)	42.1 (37.4-46.3)	0.1 (0.0-0.2)	3.0 (-0.2-6.4)
Scotland	2.8 (0.7-4.1)	5.5 (0.5-10.6)	4.2 (0.6-10.4)	19.4 (17.8-21.2)	1.8 (1.3-2.4)	3.0 (0.9-5.5)	0.0 (0.0-0.0)	11.2 (6.6-16.2)
Senegal	1.5 (0.4-2.3)	0.9 (0.4-1.5)	1.0 (0.1-3.0)	8.3 (7.0-9.8)	3.6 (2.7-4.7)	11.7 (5.8-19.6)	29.8 (21.8-37.0)	0.7 (-0.6-1.8)
Serbia	3.4 (0.8-4.9)	5.2 (3.0-7.3)	2.4 (0.5-6.7)	21.9 (20.1-23.6)	3.3 (2.4-4.3)	17.9 (15.1-20.8)	3.4 (1.2-7.5)	10.8 (6.3-15.3)
Seychelles	1.9 (0.4-3.0)	10.0 (6.0-14.2)	1.4 (0.2-4.3)	19.6 (17.3-21.9)	3.4 (2.5-4.4)	14.5 (8.0-22.2)	0.1 (0.0-0.4)	0.1 (-0.2-0.5)
Sierra Leone	1.3 (0.3-2.0)	6.3 (3.7-9.3)	0.7 (0.1-2.3)	9.9 (8.1-11.8)	2.9 (2.0-3.8)	6.9 (3.0-12.9)	33.1 (26.3-39.7)	0.2 (-0.2-0.9)
Singapore	2.1 (0.4-3.2)	1.3 (-0.1-2.7)	2.2 (0.2-6.6)	12.2 (11.0-13.4)	1.7 (1.2-2.2)	16.1 (10.5-21.9)	0.0 (0.0-0.1)	0.1 (-0.1-0.4)
Slovakia	3.4 (0.8-5.0)	7.0 (4.0-9.8)	2.3 (0.5-6.3)	17.6 (16.2-19.2)	2.9 (2.1-3.8)	15.0 (12.6-17.8)	0.1 (0.0-0.4)	9.9 (6.6-14.0)
Slovenia	2.6 (0.6-3.9)	3.5 (-1.2-8.1)	2.6 (0.5-6.9)	17.8 (16.0-19.6)	2.4 (1.8-3.1)	12.4 (10.2-14.8)	1.0 (0.2-2.5)	10.0 (5.9-14.5)
Solomon Islands	1.0 (0.2-1.6)	2.0 (0.0-4.4)	0.6 (0.1-1.7)	24.1 (21.6-26.6)	5.6 (4.0-7.5)	3.8 (1.1-10.0)	42.8 (36.6-47.5)	0.1 (-0.1-0.4)
Somalia	1.2 (0.3-1.9)		0.3 (0.1-1.2)	8.6 (6.8-10.5)	2.3 (1.6-3.1)	1.5 (0.3-4.5)	49.0 (40.0-60.1)	0.7 (0.0-1.6)
South Africa	1.8 (0.3-2.7)	8.8 (6.4-10.9)	2.3 (0.4-6.2)	13.0 (11.6-14.6)	3.0 (2.1-3.9)	21.7 (17.8-26.1)	3.9 (1.9-6.4)	6.1 (4.7-7.8)
South Sudan	1.3 (0.3-2.1)	0.3 (-0.5-1.3)	0.4 (0.1-1.6)	8.3 (6.5-10.2)	2.1 (1.4-2.8)	7.1 (3.1-12.9)	35.7 (28.7-41.7)	0.3 (-0.1-0.8)
Spain	2.4 (0.6-3.5)	7.1 (4.8-9.5)	3.6 (0.5-9.3)	15.2 (13.8-16.8)	1.9 (1.4-2.4)	5.9 (4.0-7.9)	0.1 (0.0-0.3)	8.9 (6.6-11.1)
Sri Lanka	2.4 (0.5-3.6)	5.6 (3.7-7.6)	1.4 (0.2-4.7)	9.7 (8.3-11.2)	2.6 (1.8-3.4)	14.3 (9.9-19.0)	13.1 (6.5-22.1)	0.4 (-0.1-1.0)
Suriname	1.6 (0.4-2.4)	7.9 (5.4-10.5)	2.1 (0.3-5.2)	19.1 (17.0-21.3)	3.7 (2.7-4.8)	17.8 (8.6-28.8)	3.2 (1.2-6.1)	0.2 (-0.3-0.9)

Sweden	3.3 (1.1-4.6)	7.0 (3.7-10.2)	3.0 (0.4-9.0)	15.4 (13.7-17.1)	1.1 (0.9-1.4)	1.9 (0.5-3.7)	0.0 (0.0-0.0)	10.3 (5.9-16.5)
Switzerland	3.0 (0.9-4.4)	7.0 (3.4-10.3)	4.1 (0.6-10.5)	14.9 (13.5-16.6)	1.5 (1.1-1.9)	6.0 (4.0-8.1)	0.0 (0.0-0.0)	10.7 (6.8-15.6)
Syrian Arab Republic	4.6 (2.4-6.0)	0.8 (0.4-1.3)	3.5 (0.7-8.2)	18.1 (16.4-20.0)	4.1 (3.0-5.2)	24.5 (20.2-28.7)	0.0 (0.0-0.1)	6.0 (2.7-9.8)
Taiwan (Province of China)	2.2 (0.5-3.3)	7.0 (4.5-9.7)	1.9 (0.3-5.1)	17.9 (16.3-19.7)	3.4 (2.5-4.3)	19.8 (17.2-22.4)	0.8 (0.2-2.3)	3.5 (1.9-5.7)
Tajikistan	1.6 (0.9-2.3)	4.7 (2.4-7.1)	0.4 (0.1-1.2)	14.6 (12.6-16.8)	3.4 (2.4-4.3)	22.5 (12.2-33.2)	12.2 (6.6-18.6)	13.2 (7.3-20.4)
Thailand	1.4 (0.2-2.2)	9.7 (6.9-12.4)	1.2 (0.2-3.6)	17.5 (15.7-19.5)	3.0 (2.2-3.8)	22.3 (18.5-25.9)	5.1 (2.2-9.9)	0.5 (-0.1-1.2)
Timor-Leste	1.3 (0.2-2.1)	4.9 (2.3-7.9)	0.8 (0.1-2.7)	16.6 (13.6-20.2)	3.8 (2.6-5.0)	6.7 (3.0-12.2)	31.8 (24.8-37.3)	0.7 (-0.4-2.1)
Togo	1.5 (0.3-2.2)	5.2 (2.6-8.2)	0.7 (0.1-2.4)	10.7 (9.1-12.4)	2.6 (1.9-3.4)	11.1 (5.6-17.8)	32.6 (25.4-39.2)	0.2 (-0.1-0.5)
Tokelau	1.7 (0.4-2.5)	3.4 (1.0-5.8)	1.5 (0.2-4.0)	17.5 (14.7-20.4)	5.3 (3.9-6.8)	4.0 (0.0-10.9)	0.0 (0.0-0.0)	0.1 (-0.2-0.4)
Tonga	1.9 (0.5-2.9)	1.3 (-0.2-3.2)	1.9 (0.3-4.9)	17.7 (15.5-20.0)	4.5 (3.3-5.7)	7.9 (2.7-17.0)	12.4 (6.3-19.6)	0.6 (-0.5-2.1)
Trinidad and Tobago	3.0 (1.0-4.3)	6.0 (4.0-8.1)	3.7 (0.6-8.7)	13.6 (12.0-15.3)	2.8 (2.0-3.6)	18.0 (6.3-31.9)	0.0 (0.0-0.1)	0.0 (-0.2-0.4)
Tunisia	5.9 (2.7-7.8)	1.1 (0.6-1.7)	2.6 (0.4-7.4)	16.1 (14.3-18.0)	4.4 (3.3-5.6)	23.8 (18.1-29.7)	0.1 (0.0-0.2)	5.1 (1.3-9.3)
Turkey	5.2 (2.9-6.5)	1.3 (0.6-2.0)	3.8 (0.7-8.9)	19.9 (18.2-21.7)	3.6 (2.7-4.6)	21.0 (17.8-24.4)	0.1 (0.0-0.3)	9.1 (6.5-11.7)
Turkmenistan	4.6 (2.5-6.0)	6.7 (4.3-9.3)	1.2 (0.2-3.8)	20.2 (18.6-22.1)	5.5 (4.1-7.1)	23.9 (15.0-34.0)	0.0 (0.0-0.1)	8.1 (6.1-10.2)
Tuvalu	1.5 (0.4-2.2)	2.6 (0.5-4.9)	1.1 (0.2-3.0)	21.1 (18.1-24.1)	5.2 (3.8-6.7)	4.6 (2.0-9.4)	3.8 (1.3-7.5)	0.1 (-0.1-0.3)
Uganda	1.2 (0.2-1.9)	13.3 (9.3-17.6)	0.4 (0.1-1.6)	7.0 (5.6-8.6)	1.7 (1.2-2.3)	7.6 (3.7-13.2)	34.8 (28.5-40.5)	1.6 (-0.1-4.0)
Ukraine	4.0 (1.2-5.7)	6.2 (3.5-9.1)	1.5 (0.3-4.7)	20.8 (18.5-23.2)	3.0 (2.2-3.8)	12.3 (7.5-17.5)	0.8 (0.2-1.8)	10.0 (6.4-14.1)

United Arab Emirates	5.9 (1.5-8.8)	1.6 (0.2-3.2)	6.5 (0.8-15.5)	21.5 (18.5-24.6)	5.8 (4.1-7.6)	39.9 (33.0-46.9)	0.0 (0.0-0.0)	3.3 (-5.2-10.8)
United Kingdom	2.6 (0.6-3.8)	4.7 (1.7-7.6)	4.0 (0.6-9.8)	16.8 (15.4-18.4)	1.7 (1.3-2.2)	6.0 (4.0-8.2)	0.0 (0.0-0.0)	11.5 (6.3-17.3)
United Republic of Tanzania	1.3 (0.2-2.1)	9.6 (6.5-13.0)	0.5 (0.1-2.0)	12.6 (10.5-14.8)	2.5 (1.8-3.3)	6.1 (3.1-10.4)	35.8 (30.2-40.7)	2.4 (0.4-5.1)
United States of America	2.4 (0.6-3.5)	6.0 (3.8-8.2)	1.9 (0.2-5.9)	19.6 (18.0-21.2)	1.6 (1.2-2.0)	4.5 (2.4-6.8)	0.0 (0.0-0.0)	6.8 (5.2-8.6)
United States Virgin Islands	2.1 (0.5-3.1)	5.6 (-0.7-12.9)	1.8 (0.3-5.0)	10.9 (9.1-13.1)	2.2 (1.6-3.0)	6.5 (3.1-9.8)	0.2 (0.0-0.5)	0.2 (-0.3-0.9)
Uruguay	3.0 (0.9-4.3)	6.2 (3.7-8.6)	1.9 (0.2-6.7)	18.4 (16.8-20.1)	3.1 (2.3-3.9)	6.5 (3.4-10.3)	0.5 (0.1-1.1)	6.8 (3.8-9.8)
Uzbekistan	4.5 (2.4-6.0)	4.7 (2.6-6.9)	0.9 (0.2-3.2)	16.0 (14.7-17.4)	3.4 (2.4-4.4)	27.6 (18.0-37.1)	3.2 (1.3-6.5)	9.7 (6.9-12.2)
Vanuatu	1.5 (0.4-2.2)	3.0 (1.4-5.0)	0.6 (0.1-2.1)	14.3 (12.1-16.6)	4.0 (2.9-5.3)	5.6 (1.6-12.2)	37.1 (28.9-42.8)	0.5 (-0.4-1.8)
Venezuela (Bolivarian Republic of)	1.2 (0.3-1.9)	7.0 (4.6-9.3)	0.6 (0.1-2.4)	12.6 (10.9-14.4)	3.0 (2.1-3.9)	19.2 (13.1-25.6)	0.1 (0.1-0.3)	1.2 (0.3-2.4)
Viet Nam	1.3 (0.2-2.2)	12.5 (8.4-16.4)	0.9 (0.1-3.1)	22.8 (20.8-24.8)	3.9 (2.9-5.0)	15.1 (10.9-19.2)	13.0 (7.0-20.4)	1.7 (0.0-3.9)
Yemen	5.8 (3.1-7.6)	0.6 (0.3-0.9)	3.3 (0.6-8.3)	19.3 (17.3-21.6)	4.9 (3.7-6.2)	19.1 (9.9-28.8)	16.2 (10.0-23.3)	2.2 (-0.2-4.4)
Zambia	1.0 (0.2-1.7)	8.3 (4.5-12.2)	0.6 (0.1-2.1)	10.3 (8.6-12.1)	2.5 (1.6-3.3)	10.8 (5.7-17.2)	29.7 (22.8-35.9)	2.6 (-0.8-6.9)
Zimbabwe	2.0 (0.3-3.0)	3.8 (1.6-6.3)	0.8 (0.1-3.1)	13.2 (10.9-16.0)	2.9 (2.1-3.8)	7.9 (4.1-12.9)	28.8 (23.1-34.1)	3.1 (0.5-6.1)

Supplement Table 10a. Percent of DALYs (with 95% uncertainty intervals) due to ischaemic stroke for high systolic blood pressure, high body-mass index, high fasting plasma glucose, high LDL cholesterol, low glomerular filtration rate, diet low in vegetable, diet low in fruits and diet high in sodium by location for both sexes combined in 2019, all ages

Country, region	High systolic blood pressure	High body-mass index	High fasting plasma glucose	High LDL cholesterol	Low glomerular filtration rate	Diet low in vegetables	Diet high in red meat	Diet low in fruits	Diet high in sodium
GBD regions									
High-income Asia Pacific	42.9 (32.6-54.1)	7.2 (2.8-13.0)	18.1 (8.5-39.7)	20.9 (8.1-41.5)	8.0 (4.1-11.5)	0.7 (0.2-1.4)	4.2 (1.4-6.1)	3.9 (1.1-7.4)	9.1 (1.4-20.3)
Central Asia	57.4 (46.7-65.8)	30.3 (20.6-40.5)	28.2 (14.4-52.3)	25.2 (16.6-38.9)	10.6 (8.6-12.9)	0.5 (0.2-0.8)	10.0 (5.3-12.9)	5.1 (1.3-9.6)	6.1 (0.5-16.5)
East Asia	50.7 (39.2-60.6)	12.6 (5.9-20.7)	17.1 (9.4-30.9)	20.8 (11.9-36.0)	8.7 (6.7-10.8)	0.3 (0.2-0.5)	9.1 (5.0-11.8)	3.9 (1.0-7.5)	18.5 (7.5-31.4)
Southeast Asia	57.1 (47.3-65.6)	14.3 (8.6-20.9)	23.4 (11.9-44.7)	21.1 (13.1-34.4)	11.3 (8.9-13.9)	3.1 (0.5-5.7)	3.3 (0.9-4.9)	5.1 (1.4-9.5)	11.8 (2.0-24.2)
South Asia	50.3 (40.1-58.9)	13.2 (7.8-19.7)	27.3 (14.4-49.2)	18.3 (11.2-30.7)	9.8 (7.6-12.1)	3.4 (0.6-6.1)	1.2 (0.3-2.0)	7.6 (2.5-12.9)	5.7 (0.4-15.9)
Southeast Asia, East Asia, and Oceania	52.1 (41.0-61.6)	13.0 (6.6-20.6)	18.6 (10.0-33.8)	20.9 (12.1-35.7)	9.3 (7.1-11.5)	0.9 (0.3-1.6)	7.8 (4.1-10.2)	4.2 (1.1-7.9)	16.9 (6.4-29.6)
Oceania	40.1 (31.4-47.9)	22.3 (13.4-32.5)	32.5 (18.8-52.7)	25.8 (18.0-37.3)	10.3 (8.3-12.4)	4.5 (0.7-8.1)	5.6 (1.6-8.2)	6.6 (1.7-12.1)	7.1 (0.7-17.3)
Australasia	43.0 (31.9-56.7)	17.6 (10.8-25.6)	20.7 (9.3-47.6)	23.8 (9.0-47.6)	6.6 (2.6-10.2)	1.9 (0.3-3.5)	11.3 (7.7-13.9)	3.8 (0.9-7.2)	2.1 (0.2-8.1)
Central Europe, Eastern Europe, and Central Asia	55.2 (44.7-64.9)	25.7 (16.9-34.6)	20.5 (11.3-36.0)	24.7 (13.1-43.1)	9.5 (7.2-11.9)	1.3 (0.2-2.3)	7.4 (3.5-9.7)	4.5 (1.2-8.6)	8.0 (1.7-17.9)
Central Europe	53.7 (42.5-63.9)	23.1 (15.2-31.7)	26.7 (13.3-50.3)	22.7 (10.3-42.9)	8.2 (5.8-10.5)	0.8 (0.2-1.6)	7.5 (3.9-9.8)	3.7 (1.0-7.1)	15.2 (4.9-28.0)
Eastern Europe	55.6 (45.1-65.2)	26.0 (17.2-35.1)	16.8 (9.8-28.5)	25.5 (13.5-44.1)	9.9 (7.4-12.5)	1.6 (0.2-2.9)	6.9 (2.9-9.3)	4.8 (1.3-9.0)	5.3 (0.5-14.6)
Western Europe	45.9 (35.1-58.1)	13.7 (7.5-21.2)	26.5 (12.2-60.6)	22.6 (8.0-46.6)	5.7 (2.1-8.8)	1.7 (0.3-3.1)	8.0 (4.5-10.2)	3.0 (0.8-5.6)	3.4 (0.3-10.9)

Central Latin America	47.6 (37.9-57.1)	22.8 (14.4-31.6)	32.2 (16.8-59.5)	23.4 (13.2-39.6)	13.1 (9.9-16.0)	3.0 (0.5-5.5)	6.1 (2.5-8.3)	3.6 (0.9-6.7)	6.7 (0.8-17.1)
Tropical Latin America	50.3 (40.1-59.9)	23.3 (15.3-31.9)	26.7 (13.2-50.4)	24.4 (13.2-42.4)	8.5 (6.3-10.7)	3.0 (0.5-5.5)	11.3 (7.3-14.0)	2.7 (0.6-5.0)	6.4 (0.4-17.1)
Southern Latin America	47.9 (36.5-58.2)	19.2 (11.2-27.7)	26.2 (12.7-50.6)	22.1 (10.2-41.5)	8.0 (5.0-10.8)	2.1 (0.3-3.8)	11.2 (7.5-13.9)	2.8 (0.7-5.3)	5.9 (0.3-16.2)
Andean Latin America	39.0 (30.0-48.0)	24.9 (16.2-33.5)	22.2 (11.1-44.1)	22.8 (13.2-37.8)	8.7 (6.2-11.1)	3.5 (0.6-6.5)	5.1 (1.8-7.3)	3.5 (0.8-6.7)	5.6 (0.3-15.8)
Latin America and Caribbean	48.4 (38.7-57.7)	22.8 (14.8-31.2)	28.4 (14.6-53.0)	23.6 (13.1-40.4)	10.0 (7.5-12.4)	3.0 (0.5-5.6)	8.3 (4.7-10.7)	3.1 (0.8-5.8)	6.1 (0.5-16.4)
High-income North America	41.4 (31.4-52.0)	23.3 (15.3-31.7)	29.2 (15.2-54.8)	19.6 (9.1-36.9)	8.2 (4.7-11.5)	1.9 (0.3-3.5)	9.1 (5.3-11.7)	3.7 (1.0-7.0)	4.2 (0.3-12.7)
North Africa and Middle East	52.4 (43.0-60.4)	32.1 (22.6-42.0)	28.7 (15.7-49.4)	26.4 (17.7-39.3)	12.7 (9.9-15.4)	1.5 (0.4-2.5)	3.8 (0.7-5.8)	3.4 (0.9-6.3)	1.8 (0.3-7.2)
Sub-Saharan Africa	52.0 (42.8-59.9)	18.8 (12.3-26.2)	18.9 (9.8-36.3)	18.6 (11.8-29.4)	7.0 (5.5-8.7)	3.9 (0.7-7.0)	3.2 (0.8-4.8)	5.7 (1.7-10.2)	5.6 (0.3-17.4)
Central Sub-Saharan Africa	52.0 (41.9-60.4)	13.8 (7.7-20.9)	22.4 (11.6-41.7)	18.5 (11.6-29.5)	6.5 (5.0-8.1)	4.6 (1.0-8.1)	2.4 (0.7-3.6)	5.8 (1.5-10.8)	2.8 (0.2-12.0)
Southern Sub-Saharan Africa	57.2 (46.9-66.7)	27.7 (20.1-35.8)	29.6 (14.9-54.6)	19.2 (11.2-32.5)	8.9 (6.8-10.9)	3.7 (0.7-6.6)	6.1 (2.4-8.5)	6.6 (1.9-11.6)	3.4 (0.2-13.5)
Eastern Sub-Saharan Africa	49.0 (39.8-57.1)	14.8 (8.9-22.0)	15.6 (8.0-30.4)	16.8 (10.4-27.1)	6.3 (5.0-7.9)	4.2 (0.8-7.6)	2.6 (0.4-4.2)	5.7 (1.7-10.0)	8.5 (0.4-22.1)
Western Sub-Saharan Africa	52.9 (43.8-60.7)	20.7 (13.5-28.8)	17.0 (8.8-33.5)	20.1 (13.1-30.5)	7.1 (5.6-8.7)	3.5 (0.6-6.3)	2.9 (0.5-4.5)	5.4 (1.5-9.8)	4.6 (0.2-16.4)
Countries in alphabetical order									
Afghanistan	46.5 (36.9-55.5)	26.5 (17.1-36.4)	31.2 (17.9-52.0)	31.3 (22.4-42.9)	11.3 (9.3-13.4)	6.0 (1.2-10.6)	5.2 (0.8-8.1)	7.8 (1.9-14.4)	1.8 (0.2-6.9)
Albania	55.8 (44.2-66.8)	22.1 (13.6-31.3)	14.2 (7.7-25.6)	22.3 (11.3-40.8)	7.9 (5.8-10.0)	0.3 (0.2-0.4)	7.6 (3.6-10.1)	1.5 (0.4-2.9)	16.3 (5.2-29.4)
Algeria	49.0 (38.4-58.9)	30.0 (20.6-39.7)	31.7 (17.2-57.0)	23.9 (15.0-37.4)	12.5 (9.0-15.5)	2.0 (0.2-3.9)	3.5 (0.5-5.4)	3.5 (0.7-6.7)	1.7 (0.3-6.8)
American Samoa	52.1 (42.1-61.4)	36.1 (26.3-44.7)	33.3 (20.3-53.0)	23.7 (15.6-36.2)	13.3 (10.9-15.9)	4.0 (0.7-7.3)	6.4 (2.1-9.2)	4.9 (1.2-9.3)	3.7 (0.2-12.6)

Andorra	50.1 (38.3-62.4)	16.8 (9.6-24.8)	22.4 (10.1-48.0)	24.8 (10.6-47.4)	5.7 (2.5-8.6)	1.4 (0.2-2.8)	9.5 (5.6-12.3)	2.7 (0.6-5.2)	2.8 (0.2-9.8)
Angola	55.0 (45.0-63.7)	15.0 (8.1-22.9)	22.5 (11.6-42.5)	18.8 (12.0-29.3)	6.5 (5.1-8.1)	3.6 (0.6-6.6)	3.9 (0.6-6.0)	5.3 (1.3-10.1)	3.9 (0.2-15.0)
Antigua and Barbuda	47.3 (36.4-58.3)	21.0 (13.3-29.2)	33.9 (17.2-62.0)	21.0 (11.4-36.8)	10.9 (8.2-13.6)	3.1 (0.5-5.7)	3.6 (0.7-5.5)	3.2 (0.7-6.1)	3.6 (0.2-12.5)
Argentina	44.7 (33.6-55.3)	18.6 (10.8-27.5)	24.5 (11.9-46.9)	22.1 (10.3-41.4)	7.9 (5.1-10.5)	2.2 (0.3-4.1)	12.5 (8.6-15.2)	2.1 (0.5-4.2)	5.5 (0.3-15.6)
Armenia	54.2 (42.7-64.5)	25.8 (17.4-34.8)	29.4 (13.9-58.6)	18.5 (10.1-32.4)	9.4 (7.2-11.8)	0.2 (0.2-0.2)	6.3 (2.2-8.8)	2.5 (0.5-5.0)	6.0 (0.5-16.3)
Australia	42.8 (31.5-56.7)	17.9 (11.0-25.8)	21.1 (9.2-48.9)	23.9 (9.2-47.8)	6.5 (2.5-10.2)	1.9 (0.3-3.6)	11.4 (7.8-14.0)	3.9 (1.0-7.4)	1.8 (0.2-7.7)
Austria	49.1 (37.6-62.4)	14.7 (8.1-22.5)	23.7 (10.5-53.1)	24.8 (9.5-48.9)	6.4 (2.7-9.8)	1.7 (0.2-3.3)	8.8 (5.2-11.4)	2.3 (0.5-4.5)	5.8 (0.3-16.2)
Azerbaijan	51.8 (40.4-61.6)	30.0 (19.7-40.2)	28.5 (14.1-55.3)	23.6 (15.0-37.3)	10.6 (8.4-12.8)	0.3 (0.2-0.4)	5.6 (1.5-8.3)	3.7 (0.7-7.0)	5.9 (0.4-16.3)
Bahamas	49.2 (38.9-58.5)	27.6 (18.3-36.8)	31.9 (17.1-57.2)	23.9 (14.7-38.5)	10.4 (8.2-12.7)	2.4 (0.3-4.5)	7.5 (3.0-10.3)	4.3 (1.1-8.2)	3.7 (0.2-12.6)
Bahrain	54.9 (45.3-63.7)	42.7 (30.7-53.3)	32.2 (19.0-51.2)	34.5 (25.0-47.4)	13.7 (11.0-16.3)	0.8 (0.2-1.8)	6.2 (1.6-9.4)	2.7 (0.5-5.3)	2.1 (0.3-8.4)
Bangladesh	49.5 (39.2-58.9)	8.0 (3.9-13.5)	20.2 (9.8-40.9)	14.1 (7.3-25.6)	7.5 (5.6-9.5)	4.0 (0.7-7.0)	0.8 (0.2-1.4)	6.5 (1.8-11.4)	5.5 (0.3-15.7)
Barbados	50.7 (39.6-61.9)	21.2 (13.3-29.8)	29.9 (15.3-53.7)	20.6 (9.9-38.4)	9.7 (7.0-12.3)	2.7 (0.4-5.0)	4.3 (1.1-6.3)	4.9 (1.3-9.0)	2.6 (0.2-10.1)
Belarus	59.1 (48.0-69.5)	25.0 (16.4-34.2)	13.4 (7.7-23.5)	22.0 (12.0-38.6)	9.1 (6.7-11.5)	0.7 (0.2-1.4)	10.6 (6.4-13.3)	4.6 (1.1-8.8)	3.0 (0.2-11.1)
Belgium	49.8 (37.7-62.8)	13.2 (7.2-20.3)	23.2 (10.3-51.2)	21.5 (7.7-44.1)	5.9 (2.2-9.2)	1.2 (0.2-2.3)	8.6 (5.0-11.1)	3.4 (0.8-6.5)	5.0 (0.2-14.8)
Belize	45.8 (36.5-54.7)	29.1 (19.6-38.2)	25.2 (13.7-45.7)	23.8 (15.0-37.8)	10.3 (8.2-12.5)	3.9 (0.6-7.1)	3.1 (0.4-4.9)	0.9 (0.3-1.6)	3.7 (0.2-12.6)
Benin	49.5 (40.7-58.2)	22.3 (14.5-31.2)	22.0 (10.6-43.7)	18.6 (11.8-29.1)	7.0 (5.4-8.6)	3.3 (0.5-6.1)	1.7 (0.2-2.9)	6.4 (1.8-11.5)	5.4 (0.2-18.3)
Bermuda	45.8 (34.6-56.5)	22.6 (14.3-31.4)	24.6 (11.8-50.0)	20.8 (9.4-39.7)	9.3 (6.3-12.1)	2.0 (0.3-3.7)	7.9 (3.8-10.4)	4.0 (1.1-7.6)	3.9 (0.2-12.9)

Bhutan	48.2 (37.3-59.0)	15.6 (8.6-23.7)	23.3 (11.3-45.0)	22.8 (13.1-38.9)	9.2 (7.0-11.3)	3.3 (0.6-6.0)	1.6 (0.3-2.8)	5.6 (1.4-10.3)	5.7 (0.3-15.9)
Bolivia (Plurinational State of)	36.3 (26.2-46.0)	24.8 (16.4-33.8)	24.8 (12.2-46.8)	20.9 (12.0-35.9)	8.6 (6.5-10.7)	3.7 (0.6-6.8)	7.1 (2.9-9.7)	4.3 (1.0-8.4)	5.7 (0.3-15.9)
Bosnia and Herzegovina	55.1 (43.1-66.4)	22.6 (14.7-31.5)	34.2 (17.2-62.8)	22.7 (11.4-41.8)	8.7 (6.3-10.9)	0.8 (0.2-1.7)	3.2 (0.6-4.9)	3.6 (0.8-6.9)	16.2 (4.9-29.2)
Botswana	56.2 (45.7-65.7)	28.0 (20.1-36.2)	30.8 (15.1-55.9)	16.0 (9.5-27.1)	9.5 (7.5-11.6)	4.1 (0.7-7.3)	5.8 (1.7-8.5)	7.4 (2.1-12.6)	3.5 (0.2-14.5)
Brazil	50.4 (40.1-59.9)	23.4 (15.3-31.9)	26.7 (13.2-50.3)	24.4 (13.2-42.4)	8.5 (6.3-10.7)	3.0 (0.5-5.5)	11.3 (7.3-14.1)	2.6 (0.6-5.0)	6.4 (0.4-17.1)
Brunei Darussalam	50.2 (39.8-59.3)	16.6 (8.7-25.1)	35.9 (20.0-59.9)	27.8 (18.2-42.9)	11.4 (9.0-14.0)	3.5 (0.5-6.5)	4.7 (1.0-7.1)	6.3 (1.6-11.6)	11.5 (1.8-24.2)
Bulgaria	50.9 (38.9-61.6)	22.3 (14.3-30.8)	25.9 (11.7-51.7)	21.9 (10.1-41.6)	8.7 (6.3-11.0)	0.5 (0.2-0.9)	8.9 (5.0-11.6)	4.4 (1.1-8.2)	16.1 (5.1-28.8)
Burkina Faso	42.9 (33.8-51.8)	16.6 (9.0-25.6)	19.9 (9.7-40.2)	10.8 (6.2-17.4)	6.4 (5.0-7.9)	4.6 (0.9-8.1)	4.1 (0.7-6.4)	8.3 (2.7-13.9)	4.2 (0.2-15.8)
Burundi	47.8 (38.6-56.7)	9.2 (4.1-16.0)	14.1 (7.0-28.4)	16.6 (10.5-25.9)	6.0 (4.7-7.5)	4.5 (0.8-8.0)	0.8 (0.3-1.4)	2.9 (0.5-5.6)	8.2 (0.3-22.5)
CÃ´te d'Ivoire	55.2 (45.9-63.4)	23.5 (15.0-33.1)	19.2 (10.0-36.8)	21.6 (14.9-31.3)	7.3 (5.8-8.8)	4.3 (0.7-7.8)	4.2 (0.6-6.5)	5.6 (1.3-10.6)	5.1 (0.2-18.0)
Cabo Verde	54.9 (45.1-65.4)	20.7 (13.6-29.0)	25.6 (13.2-52.3)	20.0 (11.4-33.2)	6.9 (4.9-8.7)	2.1 (0.3-4.0)	3.2 (0.5-5.1)	5.8 (1.5-10.7)	4.6 (0.2-16.6)
Cambodia	36.8 (27.2-47.1)	8.9 (4.3-14.9)	30.1 (14.4-57.7)	20.2 (11.7-34.6)	10.0 (7.7-12.5)	4.2 (0.8-7.4)	3.1 (0.5-4.9)	6.5 (1.8-11.5)	10.4 (1.2-22.5)
Cameroon	52.2 (42.4-60.7)	29.4 (20.1-39.4)	15.5 (8.9-27.7)	10.7 (6.0-17.5)	8.5 (6.8-10.2)	2.8 (0.3-5.2)	3.3 (0.4-5.4)	4.3 (0.9-8.3)	4.5 (0.2-16.8)
Canada	41.9 (30.6-54.2)	19.2 (11.8-27.5)	21.6 (9.1-49.0)	21.3 (9.0-41.7)	6.4 (3.2-9.2)	1.8 (0.2-3.3)	8.1 (4.2-10.6)	3.5 (0.8-6.6)	4.5 (0.3-13.4)
Caribbean	47.5 (37.6-56.7)	19.6 (12.4-27.7)	29.2 (15.0-53.3)	21.4 (12.3-36.6)	9.4 (7.2-11.5)	3.0 (0.6-5.3)	3.8 (0.9-5.6)	3.2 (0.9-6.1)	3.8 (0.2-12.6)
Central African Republic	53.6 (43.2-62.5)	9.8 (4.2-17.0)	23.2 (12.0-40.2)	19.2 (12.4-29.6)	6.3 (5.0-7.8)	5.3 (1.2-9.3)	8.5 (3.5-11.6)	6.6 (1.7-12.1)	3.8 (0.2-15.3)

Chad	43.3 (34.0-51.5)	11.7 (6.0-18.6)	16.8 (8.7-32.5)	18.6 (12.1-28.4)	6.2 (4.8-7.6)	4.9 (1.0-8.5)	4.0 (0.6-6.2)	7.7 (2.3-13.0)	4.2 (0.2-15.9)
Chile	55.2 (43.3-66.6)	21.1 (12.6-29.9)	32.0 (15.1-64.2)	21.8 (10.2-40.8)	8.6 (5.2-11.7)	1.6 (0.2-3.0)	8.9 (4.8-11.5)	3.9 (0.9-7.6)	6.7 (0.3-18.1)
China	50.9 (39.4-60.9)	12.7 (6.0-20.8)	17.1 (9.4-30.8)	20.7 (11.8-35.9)	8.6 (6.6-10.8)	0.3 (0.2-0.4)	9.2 (5.0-12.0)	3.9 (0.9-7.5)	18.6 (7.6-31.6)
Colombia	42.2 (32.2-52.7)	19.7 (12.2-28.1)	30.8 (14.9-62.0)	23.3 (11.9-41.1)	10.4 (7.4-13.3)	3.0 (0.5-5.6)	5.6 (1.9-7.9)	3.2 (0.7-6.2)	10.7 (2.4-22.2)
Comoros	49.7 (38.9-59.2)	15.2 (8.8-22.4)	15.9 (6.5-35.9)	20.0 (11.6-34.3)	6.7 (5.2-8.4)	4.6 (1.0-8.0)	1.6 (0.3-2.8)	4.2 (0.9-8.0)	8.8 (0.3-24.4)
Congo	57.6 (46.5-66.0)	24.5 (16.7-32.6)	24.2 (12.5-44.4)	20.4 (13.0-32.3)	7.1 (5.5-8.7)	4.9 (1.0-8.5)	3.8 (0.6-5.8)	5.8 (1.4-10.9)	4.0 (0.2-16.1)
Cook Islands	49.4 (38.3-59.4)	30.7 (20.8-39.8)	33.6 (19.4-56.3)	23.9 (14.2-39.3)	12.4 (9.8-15.0)	3.5 (0.6-6.5)	6.5 (2.3-9.2)	3.9 (0.9-7.5)	8.8 (0.7-20.6)
Costa Rica	54.1 (43.3-64.7)	20.8 (12.7-29.4)	30.2 (15.6-58.7)	24.0 (12.1-42.5)	12.7 (8.7-16.3)	3.4 (0.6-6.1)	4.7 (1.3-6.9)	2.9 (0.6-5.5)	7.1 (0.5-18.2)
Croatia	53.6 (41.4-66.3)	19.8 (12.3-28.3)	30.5 (13.8-62.0)	21.1 (7.7-43.1)	8.4 (5.5-11.2)	1.8 (0.2-3.4)	5.0 (1.7-7.1)	3.0 (0.7-5.8)	16.0 (5.3-29.1)
Cuba	42.1 (31.3-52.0)	20.7 (13.0-29.1)	29.0 (14.7-53.8)	19.6 (10.0-36.0)	9.1 (6.7-11.4)	1.6 (0.2-3.2)	4.9 (1.4-7.0)	2.7 (0.6-5.2)	3.8 (0.2-13.0)
Cyprus	47.5 (35.1-60.6)	11.5 (6.2-17.6)	31.5 (13.7-69.8)	23.4 (8.5-46.7)	7.2 (3.3-10.7)	2.1 (0.3-3.8)	6.1 (2.6-8.4)	3.0 (0.6-5.8)	3.4 (0.2-11.5)
Czechia	44.8 (33.5-56.0)	23.3 (14.9-32.3)	33.3 (16.7-63.6)	22.5 (9.5-43.3)	7.8 (5.3-10.3)	2.2 (0.3-4.0)	7.4 (3.5-9.9)	4.2 (1.0-8.1)	15.3 (5.0-27.6)
Democratic People's Republic of Korea	43.0 (32.1-52.6)	6.0 (1.3-13.5)	15.7 (8.6-27.1)	24.8 (15.6-39.9)	9.6 (7.6-11.8)	2.1 (0.2-4.2)	2.6 (0.3-4.3)	5.1 (1.2-9.7)	15.2 (4.2-28.1)
Democratic Republic of the Congo	50.2 (39.7-59.1)	12.1 (6.1-18.9)	21.9 (11.5-41.4)	18.2 (11.2-29.2)	6.4 (4.9-8.0)	4.9 (1.1-8.5)	1.0 (0.3-1.6)	6.1 (1.5-11.2)	2.1 (0.2-10.1)
Denmark	52.3 (40.5-64.6)	14.0 (8.0-20.9)	20.0 (8.9-42.9)	22.3 (8.4-45.5)	6.3 (3.1-9.3)	1.6 (0.2-3.1)	8.6 (5.0-11.0)	2.6 (0.6-5.1)	3.8 (0.2-12.6)
Djibouti	52.7 (41.9-61.9)	16.5 (9.3-24.8)	16.4 (8.1-32.6)	18.4 (11.6-28.8)	7.1 (5.7-8.6)	3.9 (0.6-7.1)	5.2 (1.1-7.7)	8.1 (2.3-13.7)	8.5 (0.3-23.6)

Dominica	44.2 (33.3-55.5)	21.7 (13.6-30.3)	33.9 (17.4-63.1)	17.9 (8.5-33.8)	10.1 (7.2-12.8)	2.4 (0.3-4.4)	4.0 (0.9-5.9)	0.3 (0.2-0.5)	3.7 (0.2-12.8)
Dominican Republic	47.8 (37.5-57.5)	23.0 (13.9-32.6)	21.0 (10.3-41.5)	24.2 (15.0-38.6)	9.1 (7.1-11.1)	3.1 (0.4-5.8)	4.3 (0.9-6.6)	1.1 (0.3-2.2)	3.9 (0.2-12.8)
Ecuador	33.8 (24.8-42.4)	28.4 (19.8-36.7)	27.7 (13.5-54.4)	23.5 (13.8-38.6)	9.7 (7.0-12.3)	3.8 (0.7-6.9)	6.7 (2.6-9.3)	1.8 (0.4-3.5)	5.5 (0.3-15.4)
Egypt	51.8 (42.4-60.1)	39.7 (27.9-50.1)	26.3 (13.3-46.4)	26.5 (18.3-37.8)	13.1 (10.6-15.7)	0.2 (0.2-0.2)	4.6 (0.8-7.1)	2.4 (0.5-4.7)	1.9 (0.2-7.6)
El Salvador	46.9 (36.6-57.6)	20.5 (12.7-28.9)	31.5 (15.7-61.7)	23.4 (12.2-41.2)	11.7 (8.7-14.9)	3.0 (0.5-5.5)	2.0 (0.3-3.4)	4.4 (1.1-8.5)	6.8 (0.4-17.6)
Equatorial Guinea	56.5 (45.9-65.3)	27.0 (19.0-35.0)	23.8 (12.5-44.0)	18.7 (11.3-30.4)	6.9 (5.3-8.7)	4.1 (0.7-7.4)	5.0 (1.3-7.5)	3.4 (0.8-6.4)	3.9 (0.2-15.2)
Eritrea	42.5 (32.6-51.3)	12.5 (6.4-19.4)	14.9 (7.3-29.1)	18.4 (11.8-28.5)	6.5 (5.1-7.9)	4.8 (0.9-8.5)	2.6 (0.3-4.5)	6.4 (1.7-11.8)	8.5 (0.3-23.3)
Estonia	52.8 (41.4-64.2)	24.9 (16.7-34.0)	15.5 (8.4-27.4)	25.4 (12.1-46.4)	9.7 (7.2-12.2)	1.6 (0.2-3.2)	6.2 (2.4-8.7)	4.4 (1.1-8.3)	2.0 (0.2-7.6)
Eswatini	55.2 (43.9-65.0)	31.9 (22.1-41.0)	29.9 (15.5-52.9)	14.6 (8.2-25.7)	9.4 (7.5-11.5)	4.3 (0.8-7.7)	5.6 (1.6-8.1)	5.6 (1.4-10.4)	3.4 (0.2-14.2)
Ethiopia	36.0 (27.8-44.6)	10.5 (5.2-16.9)	11.2 (5.9-21.7)	13.1 (7.5-21.9)	5.9 (4.5-7.4)	4.2 (0.8-7.5)	2.2 (0.3-3.6)	7.5 (2.3-12.5)	7.6 (0.3-21.0)
Fiji	59.0 (48.8-67.4)	35.1 (24.3-44.6)	33.9 (21.0-53.1)	27.8 (19.0-41.1)	13.3 (10.9-15.8)	4.0 (0.6-7.3)	8.0 (3.0-11.0)	7.5 (2.1-13.3)	8.8 (0.7-20.7)
Finland	49.9 (37.4-63.0)	14.2 (7.9-21.9)	30.4 (13.9-63.7)	21.0 (7.5-42.9)	5.3 (2.3-7.9)	2.1 (0.3-3.9)	7.3 (3.5-9.8)	3.9 (1.0-7.5)	3.4 (0.2-11.0)
France	46.9 (35.2-59.6)	13.3 (7.5-20.6)	12.9 (6.5-26.9)	23.9 (8.9-48.7)	5.1 (1.6-8.3)	2.1 (0.3-4.0)	9.8 (6.0-12.4)	3.9 (1.0-7.6)	3.2 (0.2-10.9)
Gabon	52.9 (42.6-62.7)	25.9 (17.4-35.1)	27.7 (14.2-49.6)	19.4 (11.5-31.7)	7.5 (5.8-9.4)	4.1 (0.7-7.3)	8.8 (4.0-11.8)	2.1 (0.4-4.1)	4.0 (0.2-15.0)
Gambia	54.4 (44.9-63.4)	21.0 (13.4-29.3)	18.6 (9.1-36.1)	21.5 (13.8-33.1)	7.0 (5.5-8.7)	4.6 (0.8-8.1)	1.7 (0.2-2.9)	8.5 (2.8-14.1)	4.5 (0.2-16.4)
Georgia	54.9 (43.0-66.3)	19.7 (12.4-28.0)	31.7 (15.2-64.1)	17.8 (8.3-34.1)	8.8 (6.5-11.2)	1.8 (0.2-3.5)	3.6 (0.7-5.5)	4.5 (1.1-8.6)	6.3 (0.5-16.5)
Germany	51.5 (39.0-64.2)	15.8 (9.0-23.8)	31.2 (14.3-69.9)	23.5 (9.1-47.0)	6.4 (2.8-9.4)	2.0 (0.3-3.6)	8.6 (4.9-11.1)	3.6 (0.8-6.9)	3.7 (0.2-12.3)

Ghana	56.0 (46.2-64.5)	29.7 (21.0-39.0)	22.2 (11.4-41.9)	26.3 (17.8-39.2)	7.6 (6.0-9.2)	3.9 (0.6-7.3)	2.4 (0.3-4.0)	2.6 (0.6-4.9)	7.0 (0.2-21.8)
Greece	41.4 (29.1-57.0)	10.8 (5.0-18.0)	21.8 (9.5-52.5)	20.6 (5.4-46.0)	5.8 (1.7-9.8)	0.4 (0.2-0.8)	7.7 (4.3-10.1)	1.1 (0.3-2.4)	3.1 (0.2-10.8)
Greenland	42.9 (32.6-52.7)	24.9 (15.6-34.7)	25.9 (12.4-53.3)	22.3 (12.5-38.4)	7.2 (5.1-9.4)	1.3 (0.2-2.6)	11.1 (6.7-14.0)	3.4 (0.8-6.6)	4.8 (0.3-14.3)
Grenada	47.6 (36.4-57.7)	22.8 (14.8-31.5)	35.4 (18.4-62.0)	20.8 (12.0-35.8)	12.0 (9.4-14.6)	3.8 (0.6-6.9)	2.6 (0.4-4.2)	3.0 (0.6-5.7)	3.8 (0.2-12.9)
Guam	53.0 (42.8-61.6)	32.1 (20.8-42.3)	27.2 (15.5-46.8)	26.5 (17.6-40.0)	11.9 (9.6-14.3)	3.5 (0.6-6.4)	8.2 (3.6-11.4)	3.6 (0.8-7.0)	8.4 (0.7-19.2)
Guatemala	44.7 (35.0-55.2)	16.3 (9.4-24.2)	31.6 (16.2-60.9)	20.8 (11.5-35.8)	11.6 (8.4-14.7)	3.1 (0.5-5.8)	2.0 (0.3-3.4)	4.7 (1.1-8.9)	6.4 (0.4-16.4)
Guinea	46.9 (37.5-55.7)	15.7 (9.2-23.3)	17.9 (8.8-35.6)	19.7 (12.5-30.2)	6.7 (5.2-8.2)	4.0 (0.6-7.2)	2.2 (0.3-3.7)	4.5 (1.0-8.5)	4.3 (0.2-15.9)
Guinea-Bissau	52.6 (42.6-60.6)	17.4 (9.5-26.8)	18.5 (9.6-34.5)	22.4 (15.4-32.3)	7.3 (5.9-8.8)	5.5 (1.1-9.5)	4.6 (0.8-7.1)	6.8 (1.7-12.7)	4.6 (0.2-17.5)
Guyana	47.7 (36.3-57.4)	24.9 (15.8-34.8)	36.4 (20.2-62.3)	22.4 (14.2-35.8)	11.3 (9.2-13.7)	3.3 (0.5-6.1)	1.5 (0.3-2.6)	5.7 (1.4-10.5)	3.9 (0.2-13.3)
Haiti	55.5 (44.8-64.6)	12.2 (6.2-19.6)	33.3 (17.7-58.8)	22.1 (14.1-34.2)	9.3 (7.5-11.2)	4.8 (0.9-8.4)	2.5 (0.3-4.1)	5.2 (1.2-9.9)	3.6 (0.2-12.5)
Honduras	49.1 (38.3-59.1)	20.4 (11.9-29.9)	33.8 (17.0-61.4)	22.0 (13.0-37.2)	12.2 (9.6-14.8)	3.3 (0.5-6.1)	2.7 (0.4-4.4)	4.5 (1.1-8.7)	7.4 (0.5-18.4)
Hungary	50.1 (38.5-60.9)	25.9 (17.5-34.6)	31.0 (14.7-59.7)	22.8 (11.4-41.7)	8.4 (6.1-10.6)	1.1 (0.2-2.2)	6.9 (3.1-9.4)	4.4 (1.0-8.3)	18.8 (7.4-31.4)
Iceland	46.0 (33.6-58.6)	16.0 (9.2-24.2)	22.8 (10.3-51.3)	25.0 (9.7-49.0)	5.0 (2.1-7.7)	2.4 (0.3-4.3)	10.0 (6.3-12.5)	3.8 (1.0-7.4)	4.2 (0.2-13.0)
India	50.0 (39.5-58.7)	13.5 (8.0-19.9)	29.1 (15.3-52.1)	18.6 (11.4-30.9)	10.3 (8.0-12.6)	3.1 (0.5-5.7)	0.8 (0.3-1.4)	8.0 (2.6-13.4)	5.7 (0.5-15.9)
Indonesia	64.6 (54.0-73.3)	15.6 (9.6-22.6)	20.4 (10.3-39.7)	18.5 (11.4-30.3)	11.2 (8.7-13.8)	3.5 (0.6-6.4)	1.6 (0.3-2.6)	5.4 (1.4-10.1)	12.3 (2.1-25.3)
Iran (Islamic Republic of)	48.5 (39.1-56.9)	27.2 (18.9-35.9)	28.2 (15.1-50.3)	26.7 (17.3-40.9)	12.1 (9.1-14.9)	0.6 (0.2-1.2)	3.4 (0.6-5.4)	1.6 (0.4-3.1)	1.9 (0.3-7.3)
Iraq	61.3 (51.2-69.6)	33.3 (22.5-44.0)	31.2 (17.7-51.5)	26.2 (17.4-39.7)	14.5 (11.4-17.3)	0.8 (0.2-1.9)	1.6 (0.3-2.9)	5.4 (1.3-10.3)	2.0 (0.3-8.1)

Ireland	49.6 (37.4-62.8)	15.2 (8.6-23.0)	24.4 (11.2-54.1)	21.0 (7.8-42.2)	6.5 (3.1-9.6)	1.6 (0.2-3.0)	11.1 (7.5-13.7)	3.5 (0.8-6.8)	2.6 (0.2-9.6)
Israel	50.6 (39.2-62.9)	16.7 (9.5-25.0)	26.9 (12.2-58.3)	21.7 (9.5-41.9)	7.0 (3.4-10.4)	0.3 (0.2-0.5)	5.9 (2.4-8.2)	1.4 (0.4-2.9)	3.6 (0.2-12.1)
Italy	40.2 (28.8-53.8)	10.5 (5.1-17.5)	28.1 (11.8-68.0)	21.7 (6.4-47.4)	5.3 (1.6-8.8)	1.3 (0.2-2.4)	7.2 (4.0-9.5)	1.7 (0.5-3.3)	3.9 (0.3-12.0)
Jamaica	43.6 (32.8-54.8)	19.5 (12.6-27.0)	33.0 (15.8-66.3)	18.6 (8.4-36.5)	9.0 (6.3-11.7)	2.5 (0.4-4.6)	2.3 (0.3-3.7)	3.3 (0.7-6.5)	3.6 (0.2-12.4)
Japan	44.4 (33.6-56.4)	6.0 (2.1-11.5)	16.2 (7.6-35.7)	20.8 (7.4-42.2)	8.0 (3.8-11.8)	0.7 (0.2-1.3)	3.4 (0.9-5.1)	4.0 (1.1-7.5)	8.0 (1.0-19.0)
Jordan	51.7 (41.3-60.8)	38.7 (27.7-48.5)	29.6 (16.3-47.6)	28.3 (18.9-43.3)	14.3 (11.2-17.3)	2.0 (0.2-3.8)	3.7 (0.6-5.8)	5.0 (1.1-9.5)	1.9 (0.3-7.2)
Kazakhstan	63.5 (52.6-73.2)	30.8 (20.7-40.4)	31.3 (15.9-57.8)	23.1 (14.2-37.5)	10.0 (7.8-12.3)	0.4 (0.2-0.7)	12.2 (7.4-15.3)	5.1 (1.2-9.8)	6.3 (0.5-16.9)
Kenya	50.5 (40.8-58.7)	20.2 (13.1-28.3)	14.2 (7.4-26.5)	15.7 (9.7-25.2)	6.3 (4.9-7.8)	3.5 (0.6-6.3)	3.9 (0.8-5.9)	5.7 (1.5-10.5)	5.7 (0.5-15.5)
Kiribati	41.5 (31.6-50.6)	31.7 (19.5-44.4)	34.4 (21.0-53.2)	27.7 (19.3-38.3)	12.3 (10.4-14.5)	5.1 (0.8-9.2)	4.3 (0.6-6.9)	6.8 (1.6-12.9)	7.6 (0.6-18.3)
Kuwait	49.7 (40.8-58.7)	43.6 (32.3-53.5)	28.8 (17.8-47.3)	34.8 (24.8-48.0)	11.6 (8.9-14.2)	0.4 (0.2-1.0)	10.1 (4.2-13.6)	5.4 (1.2-10.4)	3.9 (0.2-12.7)
Kyrgyzstan	41.7 (31.2-51.4)	26.6 (17.1-36.7)	12.0 (6.8-21.6)	24.4 (16.1-37.3)	9.6 (7.7-11.6)	0.6 (0.2-1.4)	9.1 (4.0-12.3)	6.3 (1.6-11.7)	5.9 (0.4-16.2)
Lao People's Democratic Republic	47.4 (37.3-56.7)	14.1 (7.4-21.1)	29.3 (15.1-53.3)	21.9 (13.5-35.2)	11.6 (9.2-14.1)	3.2 (0.5-6.0)	4.3 (0.8-6.6)	4.8 (1.1-9.1)	11.2 (1.6-23.5)
Latvia	57.4 (45.0-69.1)	22.5 (14.2-31.6)	15.5 (8.6-28.2)	23.5 (10.0-45.0)	8.8 (6.3-11.3)	1.5 (0.2-2.9)	5.8 (2.2-8.1)	4.8 (1.2-8.9)	3.8 (0.3-11.9)
Lebanon	49.8 (39.8-59.3)	28.6 (19.2-37.9)	32.4 (17.1-59.1)	27.4 (15.7-44.5)	13.1 (9.2-16.6)	0.2 (0.2-0.3)	5.4 (1.5-7.9)	1.6 (0.4-3.1)	1.7 (0.3-6.6)
Lesotho	47.9 (36.7-58.0)	23.8 (15.9-32.3)	29.0 (13.3-57.2)	11.4 (6.2-20.9)	8.5 (6.7-10.5)	4.2 (0.8-7.5)	4.3 (0.9-6.5)	7.1 (2.0-12.2)	3.3 (0.2-13.7)
Liberia	52.0 (42.7-60.1)	26.9 (18.8-35.6)	22.6 (11.4-41.9)	21.9 (14.5-32.4)	7.0 (5.5-8.6)	4.9 (0.9-8.7)	1.7 (0.2-3.0)	6.7 (1.7-12.3)	4.5 (0.2-16.1)

Libya	57.7 (47.8-66.1)	38.8 (27.5-48.9)	32.4 (18.2-56.9)	29.5 (20.6-42.1)	12.5 (9.9-15.2)	2.1 (0.2-4.1)	4.4 (0.7-6.9)	4.5 (0.9-8.8)	1.9 (0.3-7.5)
Lithuania	57.1 (45.4-68.8)	22.9 (14.1-32.2)	12.1 (6.4-22.1)	23.3 (10.7-43.8)	8.5 (6.0-11.0)	1.5 (0.2-2.9)	8.0 (3.8-10.6)	4.4 (1.1-8.4)	4.1 (0.2-13.5)
Luxembourg	50.1 (37.9-64.1)	14.3 (8.2-21.6)	31.5 (14.2-70.3)	23.4 (9.1-46.2)	6.3 (2.7-9.6)	1.8 (0.3-3.3)	10.0 (6.3-12.5)	3.5 (0.9-6.7)	3.6 (0.2-11.8)
Madagascar	51.2 (41.3-59.9)	14.7 (8.0-22.3)	12.3 (6.7-23.2)	19.2 (12.6-28.8)	6.3 (5.0-7.7)	5.1 (1.0-9.0)	3.5 (0.5-5.6)	6.7 (1.8-12.4)	8.6 (0.3-23.8)
Malawi	55.1 (44.2-64.1)	15.1 (8.2-22.8)	21.7 (10.3-42.6)	18.5 (11.3-30.0)	6.5 (5.0-8.1)	4.2 (0.8-7.6)	1.7 (0.3-2.9)	5.3 (1.3-10.1)	8.5 (0.3-23.2)
Malaysia	60.7 (49.9-69.5)	23.1 (15.4-31.1)	27.1 (14.6-47.0)	29.5 (19.1-45.9)	12.4 (9.8-14.9)	3.2 (0.4-6.0)	3.0 (0.4-4.9)	4.5 (1.0-8.6)	11.6 (1.6-24.3)
Maldives	50.7 (41.2-60.0)	15.7 (8.9-23.2)	24.1 (12.3-48.0)	28.6 (18.5-43.2)	11.7 (9.0-14.3)	2.9 (0.3-5.4)	1.5 (0.3-2.6)	6.0 (1.6-11.2)	10.4 (1.4-22.0)
Mali	40.6 (31.6-49.2)	15.5 (8.8-23.4)	17.2 (8.4-33.6)	17.9 (11.4-28.0)	6.3 (4.9-7.8)	4.1 (0.7-7.3)	5.7 (1.6-8.3)	6.0 (1.5-11.0)	4.0 (0.2-14.9)
Malta	49.2 (37.3-62.6)	13.2 (7.2-20.7)	32.3 (14.4-68.7)	22.8 (8.0-46.2)	6.8 (3.4-10.0)	0.8 (0.2-1.6)	6.5 (2.9-8.9)	3.3 (0.8-6.4)	4.3 (0.3-13.4)
Marshall Islands	41.3 (31.4-50.6)	28.4 (16.9-41.2)	38.7 (23.0-59.8)	30.7 (21.8-42.1)	12.7 (10.7-14.9)	5.5 (1.0-9.8)	6.5 (1.6-9.7)	7.3 (1.9-13.5)	7.6 (0.6-18.4)
Mauritania	51.3 (41.7-60.4)	27.1 (18.7-35.9)	12.9 (6.7-25.8)	21.1 (13.3-33.0)	7.7 (6.0-9.6)	4.5 (0.8-7.9)	5.9 (1.6-8.6)	7.8 (2.5-13.2)	4.5 (0.2-16.4)
Mauritius	49.9 (38.1-60.2)	19.8 (12.6-27.5)	30.3 (17.6-50.7)	24.3 (14.0-41.5)	14.9 (11.7-18.1)	2.6 (0.3-4.9)	3.1 (0.5-4.9)	6.0 (1.7-10.8)	11.1 (1.4-23.5)
Mexico	47.9 (37.6-57.6)	26.1 (17.1-35.3)	32.3 (18.1-56.6)	25.3 (15.0-41.3)	14.6 (11.3-17.6)	2.9 (0.4-5.3)	7.7 (3.5-10.4)	3.4 (0.8-6.3)	5.0 (0.3-14.5)
Micronesia (Federated States of)	39.7 (30.6-49.0)	34.1 (22.8-45.4)	35.8 (21.1-57.9)	28.3 (19.9-40.3)	13.8 (11.5-16.1)	5.2 (0.9-9.3)	5.9 (1.4-9.0)	6.9 (1.7-12.8)	7.7 (0.7-18.9)
Monaco	49.6 (37.2-63.3)	16.2 (8.8-24.8)	20.7 (9.4-44.1)	23.7 (8.0-48.7)	5.8 (2.4-8.9)	0.3 (0.2-0.6)	10.4 (6.8-12.9)	1.0 (0.3-2.0)	3.3 (0.2-11.5)
Mongolia	60.3 (51.1-68.6)	31.0 (20.4-42.1)	8.2 (4.7-13.9)	34.1 (24.9-46.5)	10.9 (9.2-12.9)	4.5 (0.7-8.2)	21.1 (14.8-25.8)	10.0 (3.1-16.8)	6.5 (0.5-17.3)

Montenegro	56.0 (44.5-67.4)	25.5 (16.7-34.6)	33.1 (16.1-62.4)	22.6 (10.8-42.3)	9.4 (6.7-11.9)	0.3 (0.2-0.5)	7.9 (3.9-10.4)	1.4 (0.4-2.7)	16.3 (5.1-29.7)
Morocco	60.1 (49.0-69.6)	29.5 (19.2-40.3)	33.2 (17.4-59.2)	26.1 (16.8-39.9)	13.2 (10.3-15.9)	1.1 (0.2-2.4)	4.1 (0.7-6.3)	2.0 (0.4-4.0)	1.9 (0.3-7.6)
Mozambique	57.4 (46.8-66.4)	14.2 (7.5-21.6)	19.1 (9.4-36.5)	19.8 (12.7-31.2)	6.7 (5.3-8.3)	4.6 (0.9-8.2)	1.7 (0.3-3.0)	7.0 (2.0-12.3)	8.8 (0.3-24.2)
Myanmar	51.6 (40.8-62.1)	11.2 (5.7-17.7)	28.7 (14.1-53.4)	20.6 (11.9-35.0)	11.1 (8.6-13.7)	2.9 (0.4-5.4)	3.2 (0.5-5.1)	6.0 (1.7-10.9)	11.1 (1.4-23.0)
Namibia	49.7 (38.0-60.2)	19.7 (13.4-26.2)	27.0 (12.7-52.4)	15.3 (7.9-28.0)	8.2 (6.2-10.2)	3.6 (0.6-6.5)	5.3 (1.6-7.7)	6.2 (1.8-11.0)	3.1 (0.2-13.2)
Nauru	57.4 (47.7-65.8)	41.5 (28.1-54.4)	31.3 (18.0-49.2)	36.1 (26.3-46.9)	13.9 (11.9-16.1)	5.8 (0.9-10.3)	8.8 (3.0-12.9)	7.4 (1.8-13.7)	6.3 (0.4-16.6)
Nepal	42.0 (31.6-51.4)	11.5 (5.8-18.5)	21.8 (11.4-40.4)	16.7 (9.5-29.3)	9.1 (7.1-11.1)	3.1 (0.5-5.8)	3.1 (0.5-4.9)	5.4 (1.4-10.2)	5.7 (0.3-16.3)
Netherlands	48.9 (36.1-61.9)	14.1 (7.7-21.7)	20.7 (9.1-46.4)	23.6 (9.0-46.5)	6.5 (3.0-9.6)	2.2 (0.3-4.0)	8.9 (5.1-11.4)	3.2 (0.7-6.2)	3.2 (0.2-11.0)
New Zealand	43.8 (33.1-57.0)	16.4 (9.6-24.4)	18.4 (8.1-46.0)	23.3 (8.8-46.5)	6.9 (3.0-10.2)	1.8 (0.3-3.5)	10.9 (7.3-13.5)	3.2 (0.7-6.0)	3.0 (0.2-10.5)
Nicaragua	49.2 (39.0-59.1)	21.0 (12.9-29.4)	33.2 (16.4-61.2)	22.2 (12.3-38.6)	13.9 (10.7-16.9)	4.2 (0.9-7.4)	1.8 (0.3-3.1)	6.3 (1.8-11.1)	7.3 (0.5-18.5)
Niger	46.3 (37.2-55.0)	12.9 (6.8-20.5)	15.8 (6.7-33.9)	17.5 (11.2-26.7)	6.3 (4.9-7.7)	3.0 (0.4-5.7)	5.3 (1.3-7.9)	7.1 (2.0-12.4)	4.2 (0.2-15.5)
Nigeria	55.2 (45.7-63.0)	18.8 (11.7-26.5)	14.5 (7.3-31.2)	20.4 (13.1-31.6)	7.0 (5.5-8.7)	2.9 (0.4-5.5)	2.4 (0.3-3.9)	5.3 (1.4-10.0)	3.8 (0.2-14.6)
Niue	54.6 (43.7-63.8)	27.3 (17.9-36.6)	33.9 (20.1-57.5)	21.0 (12.3-34.7)	12.7 (10.2-15.5)	3.8 (0.6-6.9)	5.5 (1.5-8.0)	4.6 (1.1-8.8)	8.9 (0.7-20.8)
North Macedonia	54.7 (42.0-66.4)	23.4 (14.9-32.4)	35.1 (16.6-64.2)	21.0 (9.3-40.6)	9.7 (7.0-12.2)	0.3 (0.2-0.6)	3.7 (0.8-5.4)	2.7 (0.6-5.2)	16.6 (5.1-29.9)
Northern Ireland	48.3 (35.8-61.9)	15.8 (8.9-24.1)	26.4 (11.5-59.7)	19.4 (6.9-40.2)	5.4 (2.4-8.2)	1.8 (0.3-3.5)	6.0 (2.9-8.2)	4.2 (1.1-7.6)	3.3 (0.2-11.2)
Northern Mariana Islands	54.2 (43.0-63.4)	37.5 (25.9-48.4)	34.8 (19.2-58.9)	27.3 (18.6-40.5)	14.4 (11.9-16.9)	4.0 (0.6-7.3)	7.6 (2.7-11.0)	4.4 (1.0-8.6)	9.1 (0.8-21.1)
Norway	49.8 (37.9-62.1)	12.6 (6.8-19.3)	27.1 (12.1-60.5)	24.2 (9.1-47.2)	5.3 (2.3-8.1)	2.4 (0.4-4.4)	7.8 (4.3-10.2)	2.9 (0.7-5.6)	3.2 (0.3-10.6)

Oman	54.4 (45.2-62.5)	39.9 (28.3-50.5)	30.1 (16.7-50.3)	32.1 (22.7-45.7)	12.7 (10.2-15.1)	1.9 (0.2-3.8)	8.4 (3.1-11.6)	2.4 (0.5-4.6)	2.1 (0.2-8.2)
Pakistan	54.5 (44.2-62.9)	17.7 (10.1-26.6)	24.7 (13.4-42.2)	21.7 (14.3-34.4)	9.6 (7.7-11.7)	4.2 (0.7-7.7)	3.8 (0.7-5.8)	6.5 (1.7-11.8)	6.0 (0.3-16.5)
Palau	52.9 (42.6-62.0)	38.1 (25.8-49.4)	37.2 (21.7-58.8)	28.8 (20.0-41.2)	15.0 (12.6-17.6)	4.6 (0.7-8.4)	7.4 (2.4-10.9)	5.6 (1.3-10.5)	8.8 (0.8-21.1)
Palestine	45.6 (35.3-54.5)	26.9 (17.9-36.6)	34.5 (18.2-61.9)	25.4 (16.0-40.2)	13.8 (10.5-16.7)	1.7 (0.3-3.4)	2.2 (0.3-3.7)	4.6 (1.1-8.9)	1.8 (0.3-7.0)
Panama	47.7 (36.7-58.9)	16.8 (10.2-24.5)	32.1 (15.5-64.9)	21.6 (10.0-40.6)	10.7 (7.3-14.0)	3.3 (0.6-6.0)	5.6 (2.0-7.9)	4.1 (1.0-7.8)	7.0 (0.5-17.6)
Papua New Guinea	33.4 (24.8-41.8)	16.7 (8.3-27.0)	32.0 (17.8-52.7)	25.1 (17.5-35.7)	8.8 (7.1-10.7)	4.3 (0.7-7.9)	5.0 (1.0-7.8)	6.6 (1.6-12.3)	6.7 (0.6-16.6)
Paraguay	49.5 (38.2-59.3)	21.2 (13.4-30.1)	28.1 (13.6-57.0)	23.9 (12.7-41.7)	9.4 (6.9-12.0)	3.4 (0.6-6.1)	10.6 (6.3-13.3)	3.1 (0.6-6.1)	6.2 (0.3-17.1)
Peru	44.1 (34.4-54.1)	22.5 (13.7-31.7)	16.9 (8.3-35.0)	23.2 (13.0-38.8)	8.0 (5.5-10.4)	3.2 (0.5-6.0)	3.0 (0.4-4.7)	4.3 (1.0-8.3)	5.7 (0.3-16.1)
Philippines	44.7 (35.4-53.4)	16.8 (9.6-24.3)	20.2 (10.8-36.1)	26.0 (17.1-39.9)	12.5 (10.1-15.1)	3.4 (0.5-6.3)	5.6 (1.6-8.1)	4.3 (1.1-8.1)	11.0 (1.7-23.2)
Poland	50.1 (39.4-60.6)	24.6 (16.2-34.0)	29.0 (15.0-52.9)	21.9 (10.7-40.2)	7.6 (5.3-10.0)	0.7 (0.2-1.3)	9.5 (5.6-12.1)	4.5 (1.2-8.4)	10.6 (1.9-22.6)
Portugal	42.3 (31.0-55.4)	11.3 (5.6-18.5)	30.2 (13.8-63.7)	22.7 (6.8-48.7)	5.7 (2.1-9.0)	0.8 (0.2-1.6)	9.0 (5.5-11.5)	2.6 (0.5-5.2)	2.8 (0.2-9.6)
Puerto Rico	46.3 (34.9-58.3)	22.2 (14.5-30.6)	29.2 (16.1-54.5)	21.0 (9.0-41.0)	9.7 (6.5-12.8)	3.4 (0.6-6.1)	3.6 (0.8-5.6)	2.9 (0.6-5.6)	3.7 (0.2-12.3)
Qatar	51.6 (42.5-60.6)	58.5 (44.5-69.2)	29.5 (16.4-46.1)	38.7 (28.5-49.7)	12.1 (10.4-14.0)	0.2 (0.2-0.5)	10.2 (3.6-14.8)	2.2 (0.5-4.8)	2.4 (0.2-9.1)
Republic of Korea	38.2 (28.5-48.1)	10.6 (4.8-17.5)	24.2 (11.1-50.1)	21.1 (10.1-38.5)	7.8 (5.1-10.5)	0.9 (0.2-1.8)	6.9 (3.0-9.3)	3.8 (0.9-7.2)	12.5 (2.6-25.1)
Republic of Moldova	62.4 (50.6-71.7)	27.3 (18.2-36.5)	16.8 (9.1-30.0)	20.4 (11.5-35.9)	9.2 (6.9-11.6)	2.1 (0.2-4.1)	3.8 (0.7-5.8)	4.9 (1.2-9.3)	3.2 (0.2-11.1)
Romania	58.5 (46.0-69.7)	21.7 (14.3-29.4)	16.5 (8.5-30.3)	22.9 (9.7-44.3)	7.7 (5.4-10.0)	0.2 (0.2-0.3)	8.6 (4.6-11.1)	3.5 (0.7-6.8)	16.5 (5.0-29.3)
Russian Federation	55.9 (45.2-65.6)	25.9 (17.1-35.2)	17.9 (10.3-30.8)	25.8 (13.6-45.3)	10.2 (7.7-12.8)	1.8 (0.2-3.5)	7.1 (3.2-9.6)	4.6 (1.2-8.8)	6.2 (0.6-16.1)

Rwanda	44.8 (34.4-54.6)	13.5 (6.8-21.5)	19.9 (8.8-43.7)	10.3 (5.6-17.9)	6.4 (4.9-8.0)	4.0 (0.7-7.2)	1.8 (0.3-3.0)	0.2 (0.2-0.3)	8.5 (0.3-23.1)
Saint Kitts and Nevis	51.4 (40.5-61.3)	25.9 (16.8-35.3)	34.9 (18.5-62.1)	22.4 (13.3-36.8)	12.3 (9.7-15.0)	3.3 (0.6-6.1)	4.4 (0.9-6.6)	6.2 (1.8-11.3)	4.0 (0.2-13.2)
Saint Lucia	48.7 (37.5-59.3)	21.0 (13.5-29.3)	33.7 (18.0-61.0)	18.1 (9.5-32.5)	10.1 (7.4-12.7)	3.6 (0.6-6.6)	5.2 (1.6-7.5)	3.7 (0.8-7.2)	3.8 (0.2-12.9)
Saint Vincent and the Grenadines	46.0 (35.6-56.5)	21.3 (13.3-30.0)	34.4 (18.4-60.6)	18.5 (9.9-32.7)	10.0 (7.6-12.4)	3.4 (0.6-6.1)	3.2 (0.5-5.0)	2.8 (0.5-5.4)	3.7 (0.2-12.7)
Samoa	46.3 (36.7-55.9)	29.9 (19.8-39.7)	35.6 (20.3-61.3)	21.2 (13.6-32.7)	12.2 (9.8-14.6)	5.0 (1.0-8.7)	6.4 (2.0-9.2)	5.8 (1.4-10.8)	2.8 (0.2-9.8)
San Marino	48.4 (35.9-61.9)	14.6 (7.9-22.5)	25.7 (11.2-59.8)	23.6 (8.2-47.6)	5.3 (2.1-8.2)	1.5 (0.2-2.8)	8.3 (4.7-11.0)	2.6 (0.6-5.1)	3.3 (0.2-11.5)
Sao Tome and Principe	57.8 (48.4-65.9)	26.9 (18.2-37.0)	20.7 (11.0-38.7)	22.1 (14.5-32.8)	8.5 (6.8-10.4)	4.6 (0.7-8.1)	1.1 (0.3-1.9)	2.9 (0.5-5.6)	4.7 (0.2-17.3)
Saudi Arabia	51.8 (42.1-60.7)	50.2 (37.6-60.8)	28.3 (17.0-43.8)	34.9 (25.3-47.0)	15.0 (12.5-17.5)	3.0 (0.2-5.8)	4.6 (0.6-7.3)	6.2 (1.4-12.2)	2.2 (0.2-8.7)
Scotland	47.5 (35.8-59.7)	18.2 (10.7-26.6)	31.6 (14.2-68.7)	21.7 (8.0-44.5)	5.9 (2.8-8.9)	1.9 (0.3-3.5)	5.5 (2.5-7.7)	4.1 (1.1-7.6)	2.2 (0.2-8.7)
Senegal	55.2 (45.1-63.6)	20.1 (12.7-28.4)	24.4 (12.9-45.7)	19.8 (12.4-30.8)	6.9 (5.4-8.5)	3.6 (0.6-6.7)	3.0 (0.4-4.9)	7.0 (2.0-12.3)	4.6 (0.2-16.4)
Serbia	58.1 (45.9-69.6)	22.4 (14.2-31.0)	33.6 (15.5-65.1)	24.8 (10.4-47.7)	8.9 (6.3-11.5)	1.6 (0.2-3.0)	4.3 (1.1-6.3)	2.3 (0.5-4.5)	16.5 (5.3-29.6)
Seychelles	52.7 (42.4-61.9)	21.3 (13.7-29.8)	31.2 (18.2-52.9)	27.8 (17.9-42.9)	13.0 (10.2-15.7)	4.0 (0.7-7.3)	2.5 (0.4-4.1)	4.9 (1.2-9.2)	9.8 (0.9-21.3)
Sierra Leone	56.9 (48.3-64.9)	15.0 (8.0-23.1)	10.4 (5.5-19.2)	19.5 (12.9-29.1)	6.7 (5.2-8.2)	4.1 (0.7-7.5)	1.4 (0.2-2.4)	6.6 (1.9-11.9)	4.5 (0.2-16.2)
Singapore	36.8 (27.1-46.5)	15.6 (9.2-22.9)	21.3 (11.5-40.7)	25.2 (14.0-42.7)	10.4 (7.3-13.3)	1.1 (0.2-2.3)	8.6 (4.1-11.4)	2.8 (0.6-5.4)	10.0 (1.4-21.8)
Slovakia	56.5 (44.6-67.6)	26.5 (17.5-35.7)	20.5 (10.9-38.2)	24.5 (13.0-43.8)	8.7 (6.4-11.0)	1.7 (0.2-3.4)	7.7 (3.5-10.3)	4.7 (1.2-9.0)	16.5 (5.4-29.3)
Slovenia	54.0 (41.6-66.6)	19.8 (12.3-28.5)	21.7 (10.3-44.9)	23.0 (8.9-45.7)	7.1 (4.4-9.8)	2.0 (0.3-3.7)	7.6 (3.7-9.9)	2.6 (0.5-4.9)	16.0 (5.0-29.4)
Solomon Islands	36.4 (27.0-45.2)	26.1 (15.2-38.4)	32.3 (18.2-53.4)	27.2 (19.0-38.3)	11.8 (9.8-13.9)	6.1 (1.3-10.6)	3.1 (0.3-5.2)	7.3 (1.8-13.4)	7.3 (0.6-18.2)

Somalia	46.5 (37.0-55.8)	6.2 (1.6-13.0)	15.2 (7.2-29.9)	16.1 (10.3-25.3)	6.3 (4.9-7.7)	5.1 (1.1-8.9)	5.0 (1.0-7.5)	7.6 (2.2-13.1)	8.4 (0.3-22.8)
South Africa	57.8 (47.3-67.8)	29.4 (21.4-37.8)	29.8 (15.1-54.7)	19.9 (11.5-34.1)	9.0 (6.9-11.1)	3.4 (0.6-6.2)	6.8 (2.9-9.4)	6.4 (1.8-11.3)	3.0 (0.2-12.7)
South Sudan	48.6 (37.8-57.4)	18.3 (11.3-26.1)	15.0 (7.5-29.5)	16.5 (10.1-26.6)	6.0 (4.6-7.5)	4.0 (0.6-7.2)	3.0 (0.5-4.9)	4.6 (1.1-8.7)	8.1 (0.2-21.9)
Spain	44.9 (33.4-57.8)	14.0 (7.5-21.8)	29.7 (13.3-67.3)	21.5 (7.4-45.1)	5.0 (1.2-8.4)	1.3 (0.2-2.5)	7.9 (4.2-10.3)	1.8 (0.4-3.6)	1.8 (0.3-6.8)
Sri Lanka	52.4 (41.9-62.3)	14.6 (8.3-21.2)	34.2 (17.2-63.2)	24.2 (13.5-41.5)	12.5 (9.6-15.4)	3.3 (0.5-6.0)	0.7 (0.2-1.1)	5.7 (1.5-10.5)	11.0 (1.5-23.4)
Suriname	42.2 (32.2-52.3)	23.5 (14.7-32.8)	34.4 (18.4-60.1)	24.3 (14.5-40.8)	10.7 (8.3-13.1)	3.4 (0.6-6.3)	2.1 (0.3-3.5)	4.4 (1.0-8.4)	3.8 (0.2-12.4)
Sweden	48.3 (37.0-61.2)	14.3 (8.0-21.8)	24.1 (10.8-55.5)	22.7 (8.5-45.2)	6.2 (3.3-8.7)	2.2 (0.3-4.0)	8.0 (4.4-10.5)	3.4 (0.9-6.5)	3.6 (0.2-11.8)
Switzerland	40.2 (28.2-53.9)	12.1 (6.4-19.2)	22.2 (9.7-52.3)	23.7 (8.3-48.7)	5.9 (1.9-9.4)	2.0 (0.3-3.7)	9.1 (5.3-11.7)	3.2 (0.8-6.3)	3.3 (0.2-11.2)
Syrian Arab Republic	50.8 (40.3-60.1)	31.4 (21.0-42.0)	32.1 (17.1-57.1)	26.7 (17.7-40.5)	12.8 (10.0-15.5)	2.5 (0.3-4.8)	3.8 (0.6-6.0)	3.9 (0.8-7.6)	1.9 (0.2-7.5)
Taiwan (Province of China)	43.1 (32.2-53.8)	14.2 (7.7-21.7)	23.4 (11.9-45.9)	26.1 (14.0-44.8)	10.8 (7.8-13.5)	1.1 (0.2-2.1)	10.4 (6.2-13.2)	2.6 (0.5-5.0)	8.2 (0.7-19.7)
Tajikistan	52.9 (42.0-62.5)	16.7 (9.1-25.2)	29.5 (14.3-55.5)	22.4 (14.5-35.2)	9.1 (7.2-11.1)	0.6 (0.2-1.5)	3.3 (0.5-5.3)	6.3 (1.6-11.8)	5.6 (0.4-15.5)
Thailand	42.4 (32.9-51.6)	18.6 (11.1-26.5)	22.5 (11.6-43.0)	28.2 (18.1-44.0)	12.9 (10.1-15.7)	3.6 (0.6-6.5)	3.8 (0.6-5.8)	3.1 (0.6-6.0)	10.6 (1.5-22.6)
Timor-Leste	54.3 (42.5-64.1)	6.3 (2.2-11.9)	28.6 (13.2-56.4)	17.3 (10.0-30.6)	11.2 (8.7-13.8)	4.2 (0.8-7.5)	2.7 (0.4-4.3)	6.9 (2.0-12.1)	11.8 (1.8-24.3)
Togo	53.9 (44.8-62.5)	20.8 (13.1-29.5)	10.7 (6.2-18.7)	23.4 (15.8-33.8)	7.4 (5.9-8.9)	5.1 (1.0-9.0)	1.9 (0.2-3.2)	8.9 (2.7-14.9)	4.7 (0.2-17.6)
Tokelau	47.4 (37.3-57.3)	26.4 (17.0-36.2)	34.4 (19.5-58.1)	23.8 (15.1-37.5)	12.0 (9.6-14.5)	4.2 (0.7-7.6)	5.5 (1.5-8.2)	5.4 (1.4-10.2)	8.3 (0.7-19.6)
Tonga	50.2 (40.1-59.6)	26.3 (17.7-34.9)	31.7 (17.4-55.2)	23.9 (14.5-39.1)	11.4 (8.9-14.0)	4.1 (0.7-7.3)	4.8 (1.3-7.2)	5.3 (1.4-9.8)	8.3 (0.7-19.1)
Trinidad and Tobago	53.7 (42.9-63.2)	26.3 (17.3-35.4)	34.3 (18.5-60.2)	22.9 (12.9-39.8)	10.5 (8.0-12.9)	3.5 (0.6-6.5)	3.1 (0.5-4.8)	5.2 (1.3-9.7)	4.2 (0.2-13.7)

Tunisia	48.0 (38.0-58.0)	27.4 (17.7-36.9)	33.4 (17.7-59.5)	24.4 (14.5-40.2)	12.9 (9.4-16.1)	0.6 (0.2-1.3)	3.2 (0.5-5.0)	2.9 (0.6-5.6)	1.8 (0.3-7.0)
Turkey	50.0 (39.7-59.8)	28.1 (18.4-37.5)	23.8 (12.3-46.4)	20.9 (11.4-36.7)	12.1 (8.3-15.6)	0.2 (0.2-0.2)	3.2 (0.5-5.0)	0.9 (0.3-1.7)	1.1 (0.3-4.8)
Turkmenistan	59.7 (49.0-68.5)	36.1 (24.5-47.8)	20.9 (10.1-39.5)	27.0 (18.6-40.1)	11.0 (9.0-13.1)	0.2 (0.2-0.3)	15.3 (10.4-18.6)	4.7 (1.0-9.0)	6.2 (0.5-17.3)
Tuvalu	47.3 (37.4-56.9)	25.0 (14.9-35.5)	35.6 (20.0-60.0)	24.3 (16.0-37.5)	12.2 (9.9-14.7)	4.7 (0.8-8.3)	5.2 (1.3-7.8)	6.2 (1.6-11.5)	8.3 (0.7-19.3)
Uganda	49.2 (39.2-57.7)	15.2 (8.7-22.3)	19.5 (9.6-37.6)	13.7 (8.0-23.1)	5.9 (4.5-7.5)	4.2 (0.8-7.5)	2.8 (0.4-4.5)	1.9 (0.4-3.7)	8.1 (0.2-22.2)
Ukraine	53.4 (42.8-63.5)	26.7 (17.8-36.1)	14.2 (8.2-23.2)	25.5 (14.1-43.1)	9.0 (6.7-11.5)	0.7 (0.2-1.5)	5.8 (1.8-8.2)	5.5 (1.4-10.2)	3.0 (0.2-10.9)
United Arab Emirates	55.4 (43.6-66.3)	62.3 (49.3-72.9)	27.2 (15.1-41.5)	50.7 (38.7-63.0)	12.8 (11.1-14.7)	3.0 (0.2-6.0)	10.3 (2.8-14.8)	5.7 (1.1-11.1)	2.6 (0.2-10.0)
United Kingdom	42.3 (32.1-53.9)	15.8 (8.9-23.9)	30.4 (14.1-67.5)	22.1 (7.9-45.1)	5.2 (2.0-8.2)	2.0 (0.3-3.6)	5.8 (2.6-7.9)	4.0 (1.1-7.4)	2.9 (0.3-10.4)
United Republic of Tanzania	56.4 (45.8-66.2)	17.7 (11.3-25.0)	15.5 (7.1-32.9)	19.9 (12.2-32.7)	6.6 (5.2-8.2)	3.8 (0.6-6.9)	2.2 (0.3-3.7)	4.6 (1.1-9.0)	11.6 (0.6-27.3)
United States of America	41.3 (31.3-52.0)	23.7 (15.5-32.1)	30.0 (15.9-55.7)	19.4 (9.1-36.6)	8.4 (4.8-11.7)	1.9 (0.3-3.5)	9.2 (5.4-11.8)	3.7 (1.0-7.1)	4.2 (0.3-12.7)
United States Virgin Islands	50.4 (38.4-60.8)	28.2 (18.5-37.6)	34.5 (17.3-61.6)	20.0 (10.2-37.0)	11.1 (8.5-13.7)	2.6 (0.4-4.7)	4.1 (1.0-6.3)	1.9 (0.4-4.0)	4.0 (0.2-13.8)
Uruguay	45.8 (34.0-57.1)	16.7 (9.5-24.7)	19.4 (9.3-38.9)	23.3 (9.9-44.7)	6.8 (3.9-9.4)	2.8 (0.5-5.1)	10.6 (6.8-13.2)	3.4 (0.8-6.7)	6.1 (0.3-16.5)
Uzbekistan	57.0 (45.7-65.7)	33.7 (22.7-45.7)	29.5 (14.7-53.1)	29.9 (20.4-42.7)	12.1 (10.1-14.4)	0.2 (0.2-0.2)	10.4 (4.5-13.9)	5.3 (1.2-10.3)	6.0 (0.5-16.9)
Vanuatu	59.1 (49.2-67.5)	25.3 (15.6-36.3)	32.2 (19.0-54.0)	27.3 (18.8-40.1)	11.4 (9.4-13.7)	4.8 (0.7-8.6)	8.5 (3.2-11.8)	5.7 (1.3-10.8)	8.6 (0.8-19.9)
Venezuela (Bolivarian Republic of)	52.5 (41.9-63.5)	19.9 (12.2-28.9)	33.2 (16.1-62.8)	18.9 (9.7-34.5)	12.5 (9.2-15.6)	3.0 (0.5-5.4)	5.1 (1.5-7.3)	3.6 (0.8-6.9)	7.6 (0.5-18.8)
Viet Nam	55.3 (45.0-65.2)	8.5 (3.9-14.6)	26.9 (13.2-52.1)	20.9 (12.0-35.2)	10.4 (7.9-13.0)	1.9 (0.3-3.7)	6.7 (2.5-9.4)	4.9 (1.2-9.4)	11.7 (1.7-24.2)

Yemen	46.5 (36.5-56.0)	18.5 (10.6-26.8)	22.6 (11.6-41.4)	27.0 (18.4-39.3)	10.8 (8.6-13.0)	4.5 (0.7-8.1)	2.8 (0.3-4.6)	7.0 (1.9-12.9)	1.8 (0.2-7.1)
Zambia	39.5 (29.8-48.9)	18.0 (11.2-26.0)	17.5 (8.5-34.2)	17.1 (10.3-27.8)	6.9 (5.4-8.5)	4.4 (0.8-7.8)	2.8 (0.4-4.6)	7.8 (2.4-13.2)	8.2 (0.3-22.6)
Zimbabwe	58.0 (47.4-66.6)	20.7 (13.7-28.9)	28.9 (14.5-54.0)	18.8 (11.9-30.4)	8.1 (6.4-9.9)	5.0 (1.0-8.7)	3.0 (0.4-4.9)	7.9 (2.4-13.4)	5.3 (0.2-18.4)

Supplement Table 10b. Percent of DALYs (with 95% uncertainty intervals) due to ischaemic stroke for diet low in whole grains, alcohol use, low physical activity, smoking, second-hand smoking, ambient particulate matter$_{2.5}$ pollution, household air pollution from solid fuels and low ambient temperature by location for both sexes combined in 2019, all ages

Country, region	Diet low in whole grains	Alcohol use	Low physical activity	Smoking	Second-hand smoking	Ambient particulate matter$_{2.5}$ pollution	Household air pollution from solid fuels	Low ambient temperature
GBD regions								
High-income Asia Pacific	3.7 (0.8-5.6)	1.3 (-1.6-4.3)	4.7 (0.5-14.3)	12.0 (10.9-13.2)	1.4 (1.0-1.7)	10.7 (8.0-13.9)	0.0 (0.0-0.1)	6.7 (5.0-8.5)
Central Asia	10.3 (5.5-13.2)	1.1 (-0.8-2.8)	2.9 (0.5-8.7)	15.9 (14.8-17.1)	3.5 (2.5-4.4)	20.4 (14.8-26.5)	3.4 (1.7-6.1)	9.7 (7.0-12.8)
East Asia	5.0 (1.2-7.3)	3.3 (1.0-5.6)	3.1 (0.5-9.1)	19.4 (17.7-21.1)	3.8 (2.8-4.8)	26.5 (22.6-29.6)	6.6 (3.5-11.0)	7.7 (5.9-9.9)
Southeast Asia	3.8 (0.6-6.0)	1.2 (-0.3-2.7)	3.0 (0.4-9.2)	16.2 (14.2-18.3)	3.4 (2.5-4.4)	13.6 (10.4-16.7)	12.1 (7.6-17.4)	0.9 (0.2-2.0)
South Asia	4.9 (1.5-7.2)	1.2 (0.2-2.2)	3.3 (0.7-9.2)	11.6 (10.1-13.2)	3.1 (2.2-4.0)	22.3 (17.3-26.9)	16.0 (11.4-21.5)	2.1 (-0.2-4.2)
Southeast Asia, East Asia, and Oceania	4.7 (1.1-7.0)	2.8 (0.7-5.0)	3.0 (0.5-9.1)	18.7 (17.0-20.2)	3.7 (2.8-4.7)	23.6 (19.9-26.6)	7.9 (4.5-12.3)	6.1 (4.7-8.0)
Oceania	6.5 (1.5-9.6)	-0.6 (-2.0-0.9)	3.7 (0.5-10.8)	16.8 (14.8-19.1)	4.0 (2.9-5.2)	4.7 (1.6-10.7)	28.7 (23.3-33.3)	1.3 (0.6-2.2)
Australasia	4.0 (1.0-5.9)	2.1 (-1.4-5.8)	6.5 (0.9-16.7)	9.3 (8.3-10.4)	1.0 (0.8-1.3)	2.6 (0.6-4.8)	0.0 (0.0-0.1)	5.0 (3.5-6.6)
Central Europe, Eastern Europe, and Central Asia	5.6 (1.7-8.0)	2.7 (0.1-5.1)	3.3 (0.7-9.1)	17.2 (15.9-18.5)	2.6 (1.9-3.3)	11.5 (8.8-14.3)	1.2 (0.5-2.4)	10.5 (6.9-15.4)
Central Europe	4.6 (1.2-6.7)	3.2 (0.4-5.9)	3.7 (0.8-9.8)	17.3 (16.1-18.6)	2.7 (2.0-3.5)	14.2 (12.2-16.3)	2.3 (0.8-4.8)	10.1 (6.6-13.9)
Eastern Europe	5.3 (1.4-7.8)	2.7 (0.1-5.2)	3.2 (0.7-8.9)	17.3 (15.8-18.8)	2.4 (1.8-3.1)	9.0 (5.7-12.6)	0.4 (0.1-0.9)	10.7 (6.5-17.4)
Western Europe	5.1 (1.9-7.1)	3.4 (0.2-6.6)	6.0 (0.9-15.7)	12.1 (11.1-13.3)	1.2 (0.9-1.5)	6.7 (4.9-8.7)	0.0 (0.0-0.1)	9.4 (6.6-12.5)

Central Latin America	4.0 (0.9-6.1)	1.4 (-0.1-2.8)	2.9 (0.3-9.0)	8.0 (7.1-8.9)	1.7 (1.2-2.3)	13.9 (11.1-16.8)	5.2 (3.4-7.5)	2.9 (1.9-4.1)
Tropical Latin America	4.2 (0.9-6.4)	1.8 (0.0-3.6)	9.6 (2.1-19.4)	14.1 (12.7-15.6)	2.3 (1.6-2.9)	8.2 (5.8-10.8)	2.9 (1.4-5.2)	1.7 (0.6-3.1)
Southern Latin America	4.9 (1.2-7.2)	2.8 (-0.6-6.0)	2.2 (0.3-7.2)	14.9 (13.5-16.3)	2.6 (1.9-3.4)	10.9 (7.9-14.4)	0.6 (0.2-1.4)	9.0 (6.5-11.9)
Andean Latin America	4.8 (1.1-7.2)	-0.1 (-1.9-1.6)	3.2 (0.4-9.7)	5.2 (4.5-5.9)	1.1 (0.8-1.5)	16.9 (12.5-21.3)	5.0 (2.8-7.8)	5.3 (3.9-6.9)
Latin America and Caribbean	4.3 (1.0-6.5)	1.5 (-0.2-3.1)	6.7 (1.4-14.9)	11.5 (10.5-12.6)	2.0 (1.4-2.5)	10.8 (8.5-13.2)	4.5 (3.0-6.5)	2.2 (1.2-3.3)
High-income North America	4.3 (1.1-6.5)	1.9 (-0.6-4.4)	3.6 (0.4-11.4)	15.7 (14.1-17.2)	1.1 (0.8-1.4)	3.8 (2.0-5.9)	0.0 (0.0-0.0)	5.8 (4.3-7.6)
North Africa and Middle East	8.8 (4.0-11.7)	0.1 (-0.2-0.3)	7.4 (1.5-17.2)	13.9 (12.9-14.9)	4.0 (3.0-5.0)	26.9 (23.7-29.9)	3.4 (2.4-4.7)	5.1 (3.1-7.3)
Sub-Saharan Africa	4.9 (1.2-7.3)	0.9 (-0.6-2.5)	2.6 (0.4-8.2)	6.5 (5.8-7.3)	1.6 (1.2-2.1)	10.9 (7.7-14.7)	25.8 (20.8-31.0)	1.8 (1.0-2.9)
Central Sub-Saharan Africa	5.0 (1.1-7.4)	-0.6 (-2.5-1.5)	3.4 (0.5-10.1)	5.7 (4.7-6.9)	1.2 (0.8-1.6)	9.0 (4.6-14.7)	28.3 (22.4-33.9)	1.4 (0.1-3.0)
Southern Sub-Saharan Africa	3.8 (0.7-5.9)	2.5 (0.8-4.4)	4.2 (0.7-11.3)	10.6 (9.6-11.9)	2.5 (1.8-3.2)	16.8 (13.4-20.7)	8.3 (5.7-11.3)	5.0 (3.7-6.7)
Eastern Sub-Saharan Africa	4.7 (1.1-7.1)	0.8 (-0.8-2.3)	1.5 (0.3-5.7)	7.0 (6.1-8.2)	1.4 (1.0-1.9)	5.5 (3.0-9.1)	33.6 (28.3-39.0)	2.2 (0.9-3.8)
Western Sub-Saharan Africa	5.4 (1.4-8.0)	1.0 (-0.5-2.6)	2.8 (0.4-8.9)	4.8 (4.2-5.5)	1.7 (1.2-2.1)	14.1 (9.5-19.1)	24.3 (18.4-30.6)	0.6 (-0.3-1.5)
Countries in alphabetical order								
Afghanistan	11.3 (5.8-14.9)	-0.1 (-0.2-0.0)	6.1 (1.1-16.2)	8.1 (6.7-9.7)	4.5 (3.3-5.8)	10.1 (4.5-18.0)	32.5 (24.3-39.7)	8.3 (5.8-11.3)
Albania	9.2 (5.3-11.5)	0.9 (-1.4-3.2)	3.0 (0.6-8.7)	17.9 (16.3-19.8)	3.6 (2.7-4.6)	12.9 (10.3-15.5)	6.3 (2.8-12.1)	8.8 (6.3-11.5)
Algeria	8.6 (4.0-11.5)	0.0 (-0.3-0.4)	8.0 (1.7-17.9)	12.0 (10.7-13.7)	4.8 (3.6-6.0)	24.3 (17.6-30.7)	0.1 (0.0-0.2)	3.8 (0.7-6.8)
American Samoa	5.7 (1.4-8.4)	-0.8 (-1.8-0.3)	5.9 (1.0-15.3)	17.5 (15.3-19.8)	3.8 (2.8-4.9)	4.5 (1.8-8.7)	3.6 (0.9-8.7)	0.1 (-0.1-0.4)

Andorra	4.6 (1.2-6.8)	3.4 (-0.3-7.1)	6.2 (0.9-16.1)	13.8 (11.8-15.9)	1.4 (1.1-1.8)	5.0 (2.8-7.4)	0.0 (0.0-0.0)	9.6 (4.3-16.7)
Angola	5.3 (1.3-7.9)	2.3 (-0.5-5.1)	3.2 (0.5-9.8)	8.7 (7.5-10.1)	1.8 (1.3-2.4)	12.0 (6.0-18.9)	18.0 (13.0-23.4)	1.8 (0.1-3.9)
Antigua and Barbuda	8.0 (4.1-10.5)	1.2 (-0.4-2.8)	6.1 (0.9-15.6)	7.7 (6.6-9.0)	1.6 (1.1-2.0)	13.1 (4.9-22.6)	0.2 (0.1-0.5)	0.1 (-0.3-0.6)
Argentina	4.9 (1.2-7.2)	2.7 (-0.8-6.1)	1.5 (0.3-6.0)	16.4 (14.7-17.9)	2.5 (1.8-3.2)	9.4 (5.9-13.4)	0.5 (0.2-1.2)	8.6 (6.3-11.3)
Armenia	9.5 (5.4-12.0)	0.1 (-1.5-1.7)	3.2 (0.6-9.0)	15.5 (14.3-16.9)	3.7 (2.8-4.7)	23.1 (16.3-29.8)	0.6 (0.2-1.4)	10.4 (6.5-15.9)
Australia	3.9 (0.9-5.9)	2.2 (-1.4-6.0)	6.6 (1.0-16.8)	9.1 (8.0-10.3)	1.0 (0.8-1.3)	2.7 (0.7-5.0)	0.0 (0.0-0.1)	3.9 (2.4-5.6)
Austria	3.8 (0.9-5.7)	3.2 (-0.3-7.0)	5.8 (1.0-14.7)	15.0 (13.5-16.5)	1.6 (1.2-2.0)	7.5 (5.6-9.5)	0.0 (0.0-0.1)	9.1 (5.8-13.5)
Azerbaijan	10.1 (5.5-13.0)	2.5 (0.0-5.0)	2.9 (0.5-8.8)	16.8 (14.1-19.5)	4.3 (3.1-5.5)	19.9 (12.4-28.4)	1.7 (0.6-3.7)	8.3 (6.7-9.8)
Bahamas	6.9 (2.5-9.7)	0.9 (-1.5-3.2)	6.1 (0.9-15.6)	7.7 (6.4-9.2)	1.9 (1.4-2.4)	12.2 (3.6-23.3)	0.3 (0.1-0.7)	0.3 (-0.3-1.3)
Bahrain	8.5 (2.8-11.9)	0.4 (-0.2-1.1)	7.8 (1.3-19.0)	14.9 (12.9-17.6)	3.3 (2.5-4.2)	38.2 (33.2-42.6)	0.1 (0.0-0.2)	1.8 (-1.2-4.5)
Bangladesh	3.1 (0.5-5.0)	0.1 (-0.1-0.5)	3.7 (0.6-10.0)	12.8 (10.6-15.4)	3.2 (2.2-4.2)	16.9 (11.2-22.4)	21.2 (15.3-27.9)	1.5 (-0.4-3.4)
Barbados	4.7 (1.2-6.9)	1.8 (0.0-3.7)	7.4 (1.2-17.1)	5.3 (4.6-6.1)	1.1 (0.8-1.5)	15.0 (6.5-24.7)	0.0 (0.0-0.0)	0.2 (-0.3-0.8)
Belarus	7.1 (3.1-9.7)	4.3 (0.9-7.6)	3.4 (0.7-9.1)	17.4 (15.5-19.4)	2.3 (1.7-3.0)	12.7 (9.6-15.9)	0.1 (0.0-0.4)	10.2 (6.2-15.6)
Belgium	4.2 (1.1-6.2)	3.6 (-0.4-7.6)	6.7 (1.0-16.7)	14.2 (12.8-15.6)	1.2 (0.9-1.5)	7.7 (5.9-9.9)	0.0 (0.0-0.0)	10.1 (5.2-15.4)
Belize	5.4 (1.4-7.9)	2.3 (0.6-4.0)	6.0 (0.8-15.5)	10.1 (8.7-11.5)	2.0 (1.4-2.5)	14.9 (5.8-26.0)	4.8 (2.4-8.2)	0.4 (-0.1-1.1)
Benin	4.7 (1.0-7.0)	0.0 (-1.4-1.7)	2.8 (0.4-8.9)	5.3 (4.3-6.4)	1.6 (1.1-2.0)	7.7 (3.5-13.6)	31.3 (24.6-37.5)	0.3 (-0.2-0.8)
Bermuda	4.9 (1.4-7.2)	3.2 (0.8-5.8)	7.2 (1.1-17.1)	9.6 (8.4-10.9)	1.6 (1.2-2.1)	3.5 (0.7-6.8)	0.3 (0.0-1.0)	2.4 (-3.6-10.2)
Bhutan	4.2 (0.8-6.3)	0.0 (-0.8-1.0)	5.1 (1.0-12.8)	7.0 (5.6-8.7)	1.8 (1.2-2.3)	14.2 (8.7-19.5)	18.0 (12.3-24.5)	10.5 (7.5-14.4)
Bolivia (Plurinational State of)	4.8 (1.2-7.3)	-1.1 (-3.2-1.2)	2.8 (0.3-8.9)	5.9 (4.9-7.1)	1.1 (0.7-1.5)	15.5 (9.7-21.5)	9.0 (5.2-13.7)	5.5 (4.0-7.3)

Bosnia and Herzegovina	4.1 (0.9-6.2)	1.7 (-0.5-3.8)	2.8 (0.5-8.4)	23.9 (22.2-25.7)	3.5 (2.6-4.5)	18.9 (15.8-21.8)	6.1 (2.5-12.0)	10.7 (6.7-15.0)
Botswana	4.8 (1.0-7.3)	2.3 (0.4-4.5)	3.4 (0.5-10.2)	13.6 (11.8-15.5)	3.4 (2.4-4.5)	16.3 (11.1-21.7)	10.9 (5.9-17.2)	2.4 (-1.0-6.5)
Brazil	4.2 (0.9-6.4)	1.8 (0.0-3.6)	9.7 (2.1-19.7)	14.1 (12.7-15.6)	2.3 (1.6-2.9)	8.2 (5.8-10.8)	2.8 (1.3-5.0)	1.7 (0.6-3.1)
Brunei Darussalam	5.2 (1.1-7.8)	-1.0 (-2.3-0.0)	3.9 (0.4-12.9)	15.1 (13.1-17.6)	1.9 (1.4-2.5)	4.9 (1.4-8.7)	0.1 (0.0-0.3)	0.1 (-0.1-0.4)
Bulgaria	4.8 (1.1-7.0)	4.1 (0.8-7.2)	3.1 (0.7-8.7)	18.0 (16.4-19.5)	2.7 (2.0-3.5)	13.8 (11.7-16.0)	2.8 (0.9-6.5)	10.6 (6.6-14.7)
Burkina Faso	5.4 (1.3-8.1)	0.7 (-2.9-4.5)	1.8 (0.3-6.7)	4.6 (3.5-5.9)	1.8 (1.3-2.4)	5.2 (1.8-11.0)	35.4 (27.4-43.2)	0.7 (-0.8-2.0)
Burundi	5.8 (1.4-8.5)	2.9 (0.5-5.3)	1.4 (0.3-5.6)	5.7 (4.5-7.1)	1.1 (0.7-1.4)	3.8 (1.3-8.5)	37.8 (30.6-44.9)	3.1 (0.1-6.8)
CÃ´te d'Ivoire	6.0 (1.4-8.9)	1.0 (-1.8-4.1)	2.8 (0.4-9.3)	9.1 (7.9-10.6)	2.8 (2.0-3.6)	12.3 (5.9-20.0)	27.5 (19.7-34.6)	0.2 (-0.1-0.5)
Cabo Verde	4.5 (0.9-6.7)	2.1 (0.1-4.1)	3.2 (0.5-9.7)	5.2 (4.4-6.1)	1.6 (1.2-2.1)	24.2 (17.1-30.8)	7.4 (4.2-12.0)	1.9 (-1.9-6.6)
Cambodia	2.9 (0.4-4.8)	2.0 (-0.5-4.4)	2.5 (0.3-8.3)	17.5 (15.1-20.0)	3.3 (2.5-4.3)	7.2 (3.6-11.9)	28.7 (23.3-33.9)	0.1 (0.0-0.3)
Cameroon	5.0 (1.0-7.6)	2.5 (0.4-5.0)	3.0 (0.4-9.6)	6.4 (5.1-7.9)	1.5 (1.1-2.0)	18.1 (11.2-24.7)	20.4 (13.7-27.3)	0.9 (-0.2-2.4)
Canada	4.1 (1.0-6.1)	2.0 (-0.9-5.0)	4.9 (0.7-13.9)	13.8 (12.3-15.3)	1.0 (0.8-1.3)	3.2 (1.5-5.1)	0.0 (0.0-0.0)	8.7 (2.7-15.4)
Caribbean	5.0 (1.3-7.3)	1.5 (-0.1-3.1)	6.3 (1.0-15.4)	12.4 (11.0-13.8)	1.8 (1.4-2.3)	10.7 (5.9-16.6)	8.9 (6.3-12.8)	0.5 (-0.1-1.3)
Central African Republic	5.6 (1.3-8.4)	-1.3 (-3.5-1.2)	3.1 (0.5-9.7)	6.2 (4.8-8.0)	1.5 (1.1-2.0)	5.4 (1.7-11.8)	38.6 (30.8-46.2)	0.4 (-0.1-1.2)
Chad	9.4 (4.9-12.2)	0.7 (-1.3-2.8)	2.1 (0.3-7.3)	6.0 (4.6-7.5)	1.8 (1.2-2.3)	5.2 (1.9-11.0)	35.5 (27.3-43.8)	2.0 (-1.2-5.0)
Chile	4.8 (1.1-7.1)	3.0 (-0.3-6.2)	3.4 (0.4-10.7)	12.3 (10.9-13.9)	2.8 (2.0-3.7)	15.9 (12.8-19.1)	0.9 (0.3-2.1)	10.6 (7.1-15.0)
China	5.0 (1.2-7.3)	3.3 (1.0-5.6)	3.1 (0.5-9.1)	19.5 (17.7-21.2)	3.8 (2.8-4.8)	26.8 (22.9-29.9)	6.3 (3.2-10.7)	7.7 (5.9-9.9)

Colombia	4.4 (1.1-6.5)	0.5 (-0.8-1.8)	3.1 (0.3-9.9)	7.3 (6.3-8.5)	1.8 (1.3-2.4)	14.9 (11.4-19.0)	2.9 (1.3-5.3)	2.6 (1.7-3.6)
Comoros	4.8 (1.2-7.2)	-0.3 (-0.7-0.4)	1.2 (0.3-5.2)	6.0 (4.6-7.5)	2.4 (1.7-3.1)	5.1 (2.6-8.7)	30.1 (25.1-34.6)	0.3 (-0.7-1.8)
Congo	5.1 (1.1-7.6)	-0.6 (-3.8-2.6)	3.8 (0.6-11.1)	6.0 (4.9-7.5)	1.6 (1.2-2.2)	18.6 (10.6-27.8)	16.8 (10.3-24.2)	0.5 (-0.5-2.0)
Cook Islands	5.2 (1.3-7.7)	2.1 (-0.6-4.7)	5.5 (1.0-14.1)	14.9 (12.8-17.2)	3.6 (2.7-4.8)	3.6 (0.7-8.1)	0.9 (0.1-2.5)	0.6 (-1.2-2.9)
Costa Rica	5.0 (1.5-7.4)	0.4 (-1.2-2.0)	2.3 (0.3-8.2)	8.7 (7.7-9.7)	1.9 (1.4-2.4)	12.4 (9.5-15.6)	1.4 (0.5-3.0)	1.8 (0.7-3.2)
Croatia	4.2 (1.1-6.2)	2.4 (-1.1-5.6)	3.5 (0.7-9.4)	15.9 (14.5-17.4)	3.1 (2.3-4.0)	12.5 (10.3-14.9)	0.7 (0.2-1.8)	9.7 (5.3-14.2)
Cuba	4.4 (1.1-6.6)	1.4 (-0.3-3.1)	6.6 (1.0-16.1)	18.5 (16.7-20.4)	2.2 (1.6-2.8)	12.8 (6.5-21.1)	0.4 (0.1-1.0)	0.4 (-0.2-1.2)
Cyprus	5.1 (1.6-7.3)	2.5 (-0.5-5.5)	5.1 (0.6-14.5)	14.6 (13.0-16.1)	1.8 (1.3-2.4)	10.3 (7.8-13.1)	0.0 (0.0-0.1)	4.7 (0.8-9.0)
Czechia	3.7 (0.8-5.6)	4.7 (1.0-8.3)	4.2 (0.8-11.0)	16.8 (15.3-18.4)	2.0 (1.5-2.6)	11.7 (9.7-14.1)	0.2 (0.0-0.4)	9.5 (5.8-14.1)
Democratic People's Republic of Korea	4.7 (1.0-7.1)	1.8 (0.0-3.5)	2.8 (0.4-8.6)	17.8 (15.3-20.4)	4.1 (2.9-5.2)	16.3 (10.2-22.6)	23.6 (16.6-30.4)	9.6 (6.1-14.2)
Democratic Republic of the Congo	4.8 (1.1-7.2)	-1.7 (-3.6-0.4)	3.5 (0.5-10.3)	4.6 (3.4-5.9)	0.9 (0.6-1.2)	6.6 (2.7-12.0)	33.5 (26.9-39.5)	1.5 (0.2-3.1)
Denmark	4.5 (1.2-6.6)	2.4 (-2.0-6.7)	5.2 (0.7-14.3)	19.0 (17.3-20.8)	1.2 (0.9-1.6)	5.6 (3.5-8.0)	0.0 (0.0-0.0)	10.3 (4.5-17.0)
Djibouti	6.3 (1.5-9.2)	-1.1 (-2.2-0.1)	1.5 (0.3-6.0)	12.2 (9.6-15.2)	3.2 (2.3-4.3)	22.3 (11.7-33.2)	9.6 (4.9-15.8)	1.2 (-1.2-3.2)
Dominica	8.9 (5.2-11.3)	1.7 (0.0-3.7)	6.2 (1.0-15.7)	6.1 (5.2-7.2)	1.4 (1.0-1.8)	12.9 (5.4-22.6)	1.7 (0.6-3.9)	0.4 (-0.8-2.1)
Dominican Republic	5.0 (1.2-7.5)	1.6 (-0.3-3.5)	6.9 (1.1-16.5)	14.7 (12.8-16.7)	1.9 (1.4-2.4)	13.2 (6.2-22.5)	3.3 (1.4-6.2)	1.1 (0.0-2.4)
Ecuador	5.1 (1.2-7.5)	0.6 (-0.7-1.9)	3.3 (0.4-9.9)	6.8 (5.8-7.8)	1.2 (0.8-1.6)	15.0 (10.4-19.6)	1.7 (0.7-3.5)	3.9 (2.8-5.2)
Egypt	5.8 (1.4-8.6)	-0.1 (-0.4-0.2)	6.4 (1.1-16.2)	14.5 (12.4-17.1)	4.2 (3.0-5.3)	36.7 (31.3-41.3)	0.0 (0.0-0.1)	3.3 (-0.1-7.2)
El Salvador	4.2 (1.0-6.4)	0.2 (-0.8-1.4)	2.2 (0.3-7.9)	5.6 (4.7-6.5)	1.2 (0.8-1.5)	14.2 (9.2-19.8)	4.6 (2.6-7.4)	0.2 (-0.1-0.6)

Equatorial Guinea	4.4 (0.8-6.7)	1.4 (-1.6-4.6)	4.1 (0.7-12.2)	5.4 (4.2-7.1)	1.6 (1.1-2.1)	24.9 (15.6-33.6)	5.2 (2.2-9.7)	0.7 (-1.0-3.2)
Eritrea	5.2 (1.1-7.8)	-1.1 (-2.3-0.3)	1.5 (0.3-6.0)	4.4 (3.1-5.8)	1.5 (1.1-2.0)	10.0 (4.6-17.9)	28.4 (21.5-34.9)	1.1 (0.0-2.2)
Estonia	4.5 (1.1-6.6)	3.0 (-0.5-6.8)	3.6 (0.7-9.4)	17.5 (16.0-19.2)	1.8 (1.3-2.4)	2.8 (1.1-4.8)	0.7 (0.1-2.3)	9.7 (4.9-16.7)
Eswatini	3.8 (0.6-5.8)	2.3 (0.4-4.3)	3.5 (0.5-10.5)	5.7 (4.6-6.9)	1.4 (0.9-1.9)	12.7 (8.0-18.0)	18.0 (11.7-24.7)	4.5 (1.8-7.9)
Ethiopia	5.3 (1.3-7.8)	-0.9 (-2.8-1.2)	1.4 (0.3-5.6)	2.4 (1.9-3.0)	0.7 (0.5-0.9)	5.4 (2.9-9.3)	32.8 (26.9-39.3)	3.0 (1.9-4.2)
Fiji	6.2 (1.4-9.1)	1.1 (0.0-2.5)	4.4 (0.7-12.7)	15.3 (13.5-17.2)	3.4 (2.4-4.5)	8.7 (2.8-19.1)	8.4 (3.5-15.2)	0.6 (-0.4-1.9)
Finland	4.1 (1.0-6.1)	0.5 (-2.4-3.8)	5.4 (0.7-14.9)	11.2 (10.0-12.4)	0.9 (0.6-1.1)	1.6 (0.3-3.3)	0.0 (0.0-0.0)	10.5 (4.8-18.6)
France	5.0 (1.5-7.2)	4.2 (0.3-8.4)	7.6 (1.1-17.9)	11.2 (10.0-12.7)	0.9 (0.7-1.1)	6.6 (4.8-8.6)	0.0 (0.0-0.1)	9.3 (5.7-13.0)
Gabon	4.2 (0.8-6.5)	2.8 (-0.5-6.5)	3.7 (0.6-10.6)	6.2 (5.0-7.6)	1.6 (1.1-2.2)	24.4 (15.4-33.5)	2.1 (0.9-4.0)	0.4 (-0.4-1.7)
Gambia	5.0 (1.1-7.5)	0.3 (-1.3-1.9)	2.8 (0.4-8.8)	7.3 (6.0-8.6)	2.9 (2.1-3.7)	10.1 (5.0-17.0)	30.1 (22.7-36.8)	0.4 (-0.2-1.1)
Georgia	9.0 (5.2-11.3)	1.5 (-0.3-3.2)	3.3 (0.6-9.3)	14.1 (12.9-15.5)	3.5 (2.6-4.6)	12.1 (8.8-15.8)	6.4 (2.6-11.9)	10.8 (7.2-15.6)
Germany	3.9 (0.9-5.9)	6.2 (2.0-10.4)	4.6 (0.6-13.9)	13.5 (12.1-15.0)	1.0 (0.7-1.3)	7.3 (5.3-9.5)	0.0 (0.0-0.0)	9.7 (5.2-14.5)
Ghana	5.8 (1.4-8.6)	0.4 (-1.8-2.8)	3.0 (0.4-9.3)	3.9 (3.3-4.6)	1.3 (0.9-1.7)	20.5 (13.2-27.6)	18.6 (12.3-25.9)	0.1 (-0.1-0.5)
Greece	7.7 (4.6-9.6)	1.4 (-1.2-4.0)	4.7 (0.5-14.3)	12.7 (11.5-14.1)	1.7 (1.2-2.2)	8.2 (6.2-10.4)	0.1 (0.0-0.2)	8.8 (6.7-10.9)
Greenland	4.7 (1.1-7.1)	1.9 (-1.7-5.8)	3.7 (0.4-11.6)	25.5 (22.9-28.4)	2.7 (1.9-3.6)	3.2 (0.3-9.2)	0.2 (0.0-0.6)	11.5 (1.3-21.0)
Grenada	7.0 (2.9-9.5)	2.7 (0.7-4.7)	5.4 (0.8-14.3)	7.9 (6.8-9.1)	1.6 (1.1-2.2)	16.2 (6.3-27.8)	0.8 (0.3-1.6)	0.2 (-0.2-0.7)
Guam	5.7 (1.4-8.4)	-0.6 (-2.5-1.7)	4.2 (0.6-11.9)	15.8 (13.4-18.3)	3.7 (2.7-4.8)	5.8 (2.4-9.8)	0.9 (0.2-2.6)	0.1 (-0.1-0.3)

Guatemala	4.5 (1.1-6.7)	0.0 (-0.7-0.8)	1.1 (0.2-5.1)	5.8 (4.7-7.1)	1.3 (0.9-1.7)	11.9 (7.2-16.9)	16.8 (11.2-22.9)	2.1 (1.2-3.2)
Guinea	4.6 (1.0-7.0)	-0.1 (-0.9-0.8)	2.4 (0.4-7.8)	8.6 (7.0-10.4)	2.0 (1.4-2.6)	6.3 (2.7-12.3)	34.1 (26.5-41.3)	0.4 (-0.1-1.0)
Guinea-Bissau	5.1 (0.9-7.8)	0.8 (-1.0-2.6)	2.6 (0.4-8.7)	3.9 (3.2-4.7)	2.3 (1.6-2.9)	7.8 (3.4-14.3)	35.0 (26.9-41.7)	0.3 (-0.2-1.0)
Guyana	5.0 (1.1-7.4)	2.4 (0.0-4.7)	5.4 (0.8-14.3)	9.3 (8.0-10.8)	2.2 (1.6-2.9)	15.6 (6.4-27.2)	2.1 (0.9-3.8)	0.5 (-0.2-1.5)
Haiti	5.5 (1.4-8.2)	1.7 (0.2-3.2)	4.6 (0.6-13.1)	4.8 (3.9-5.8)	1.3 (1.0-1.8)	5.1 (2.0-10.1)	33.8 (28.2-38.8)	0.5 (-0.3-1.7)
Honduras	4.4 (1.0-6.6)	1.1 (0.1-2.1)	1.8 (0.3-6.3)	9.6 (8.2-11.2)	2.4 (1.7-3.1)	9.5 (5.3-14.2)	20.4 (15.1-25.7)	1.5 (-0.4-4.0)
Hungary	4.9 (1.2-7.2)	3.8 (0.5-6.9)	3.0 (0.6-8.5)	18.8 (17.3-20.6)	3.1 (2.2-4.0)	12.2 (10.1-14.3)	2.4 (0.7-5.7)	9.3 (4.0-14.7)
Iceland	4.5 (1.1-6.6)	1.5 (-1.8-4.7)	6.4 (0.9-16.5)	13.2 (11.6-14.9)	1.2 (1.0-1.6)	1.7 (0.3-3.6)	0.0 (0.0-0.0)	11.2 (5.2-18.8)
India	4.6 (1.1-6.9)	1.5 (0.3-2.9)	3.2 (0.7-8.7)	11.0 (9.5-12.8)	3.1 (2.2-4.0)	23.7 (18.9-28.1)	14.5 (10.0-19.7)	1.8 (-0.3-3.7)
Indonesia	3.8 (0.6-5.9)	-0.5 (-1.3-0.3)	3.4 (0.4-10.7)	16.4 (13.5-19.7)	3.6 (2.6-4.7)	13.8 (10.3-17.5)	9.9 (5.2-16.0)	0.6 (0.0-1.6)
Iran (Islamic Republic of)	10.2 (5.6-13.1)	0.3 (0.1-0.6)	6.3 (1.2-15.9)	12.2 (11.1-13.3)	3.5 (2.6-4.4)	26.7 (23.6-30.0)	0.1 (0.0-0.1)	7.3 (5.5-9.1)
Iraq	7.6 (2.9-10.5)	-0.1 (-0.4-0.3)	7.7 (1.5-17.7)	18.2 (16.4-20.1)	4.6 (3.5-5.9)	31.9 (26.5-37.0)	0.1 (0.0-0.2)	4.3 (1.1-7.5)
Ireland	3.5 (0.7-5.2)	3.2 (0.1-6.4)	8.1 (1.3-18.2)	13.7 (12.3-15.1)	1.2 (0.9-1.6)	3.7 (1.9-5.8)	0.0 (0.0-0.0)	10.8 (4.5-17.8)
Israel	6.7 (3.0-9.0)	-0.9 (-2.4-0.7)	6.2 (0.9-16.1)	12.5 (11.2-13.8)	1.3 (1.0-1.7)	13.3 (11.0-15.5)	0.0 (0.0-0.1)	3.7 (0.1-7.8)
Italy	8.0 (4.7-10.0)	3.3 (0.8-6.0)	6.9 (1.0-17.0)	9.7 (8.7-10.8)	1.3 (1.0-1.7)	9.0 (7.2-11.0)	0.0 (0.0-0.1)	9.0 (6.8-11.2)
Jamaica	4.9 (1.4-7.2)	0.4 (-0.9-1.8)	7.6 (1.2-17.1)	8.8 (7.8-10.0)	1.7 (1.3-2.2)	10.3 (6.7-14.7)	2.2 (0.8-4.6)	0.1 (-0.3-0.9)
Japan	3.7 (0.9-5.6)	0.3 (-2.7-3.4)	4.7 (0.5-14.4)	11.5 (10.4-12.8)	1.3 (1.0-1.6)	8.0 (5.4-11.0)	0.0 (0.0-0.1)	6.5 (5.0-8.1)
Jordan	10.2 (5.5-13.0)	0.0 (-0.4-0.4)	6.0 (1.1-15.1)	19.5 (17.6-21.5)	4.0 (3.0-5.1)	24.0 (20.3-27.6)	0.0 (0.0-0.0)	4.5 (1.1-8.4)

Kazakhstan	9.9 (5.5-12.7)	0.7 (-1.5-2.9)	3.7 (0.7-10.2)	16.3 (14.8-17.8)	3.1 (2.2-4.0)	15.9 (11.0-21.9)	2.3 (0.8-4.9)	9.8 (6.6-14.4)
Kenya	4.8 (1.1-7.2)	1.4 (-0.6-3.3)	1.3 (0.3-5.6)	6.5 (5.5-7.6)	1.4 (1.0-1.8)	7.7 (4.6-11.8)	27.1 (21.6-31.9)	2.8 (1.5-4.2)
Kiribati	7.3 (1.7-10.7)	-0.1 (-1.5-1.6)	3.8 (0.5-11.6)	33.0 (30.5-35.4)	5.4 (3.9-7.1)	4.1 (1.2-10.0)	29.7 (23.3-35.4)	0.0 (0.0-0.0)
Kuwait	7.1 (1.6-10.4)	-0.1 (-0.2-0.1)	9.6 (1.9-21.6)	17.5 (15.8-19.3)	4.1 (3.1-5.1)	37.5 (33.7-41.0)	0.0 (0.0-0.0)	3.0 (-2.5-7.8)
Kyrgyzstan	10.4 (5.6-13.3)	2.3 (0.4-4.3)	2.6 (0.5-8.2)	21.3 (19.6-23.2)	4.2 (3.0-5.3)	16.8 (10.6-24.0)	9.2 (4.9-14.3)	12.7 (6.8-20.8)
Lao People's Democratic Republic	3.4 (0.5-5.4)	0.9 (-2.0-4.0)	2.4 (0.3-8.1)	17.1 (14.6-19.5)	3.6 (2.6-4.7)	7.2 (3.6-11.4)	32.1 (26.2-37.3)	1.6 (-0.2-3.7)
Latvia	4.8 (1.3-7.0)	1.2 (-1.9-4.4)	5.4 (1.2-13.0)	13.6 (12.0-15.5)	2.2 (1.6-2.9)	8.1 (5.7-10.6)	0.8 (0.2-2.0)	10.5 (6.2-17.0)
Lebanon	9.5 (5.2-12.2)	0.3 (-0.1-0.7)	8.2 (1.7-17.9)	25.2 (22.7-28.0)	4.0 (3.0-5.0)	21.6 (17.1-26.2)	0.1 (0.0-0.2)	6.3 (4.2-8.4)
Lesotho	3.3 (0.5-5.3)	0.7 (-1.4-3.1)	2.7 (0.4-8.5)	11.6 (9.1-13.8)	3.0 (2.1-4.0)	11.2 (6.4-17.1)	21.6 (15.6-27.5)	10.3 (6.5-14.3)
Liberia	5.5 (1.2-8.1)	1.2 (-0.6-3.4)	3.2 (0.5-9.9)	5.4 (4.5-6.6)	1.5 (1.1-2.0)	8.2 (4.0-14.4)	32.6 (25.5-39.0)	0.2 (-0.4-1.3)
Libya	9.4 (4.2-12.6)	0.2 (0.0-0.3)	7.7 (1.5-18.0)	12.1 (10.1-14.2)	5.3 (4.1-6.6)	28.6 (21.3-35.5)	0.1 (0.0-0.2)	3.3 (-0.6-7.6)
Lithuania	4.6 (1.2-6.8)	2.7 (-0.7-6.4)	5.4 (1.2-12.9)	12.6 (11.4-13.9)	1.8 (1.3-2.4)	6.8 (4.4-9.3)	0.3 (0.1-0.9)	10.2 (5.3-16.1)
Luxembourg	4.1 (1.0-6.1)	3.7 (-0.3-7.5)	5.5 (0.7-15.1)	14.3 (12.3-16.5)	1.4 (1.0-1.7)	5.8 (3.8-7.8)	0.0 (0.0-0.0)	9.8 (4.3-16.0)
Madagascar	4.6 (0.8-7.1)	-0.9 (-2.5-0.9)	1.4 (0.3-5.9)	5.4 (4.3-6.7)	1.9 (1.3-2.5)	4.0 (1.8-7.3)	37.6 (32.5-42.2)	2.2 (0.8-3.9)
Malawi	3.8 (0.6-5.9)	0.3 (-1.1-2.0)	1.6 (0.3-6.2)	8.2 (6.5-10.2)	1.4 (0.9-1.8)	3.9 (1.7-7.5)	35.3 (29.8-40.6)	2.4 (0.3-5.0)
Malaysia	5.1 (1.2-7.7)	0.3 (-0.6-1.2)	4.7 (0.6-13.3)	13.5 (12.0-14.8)	3.6 (2.6-4.7)	13.9 (10.0-17.8)	0.2 (0.0-0.4)	0.4 (-0.1-1.1)
Maldives	6.2 (1.4-9.1)	-0.9 (-2.7-1.0)	4.4 (0.6-12.9)	16.3 (14.8-17.8)	3.7 (2.8-4.6)	8.2 (5.4-11.3)	3.9 (1.7-7.3)	0.0 (0.0-0.0)

Mali	4.8 (1.1-7.2)	0.1 (-0.6-0.8)	2.1 (0.3-7.1)	4.8 (3.8-6.0)	1.7 (1.2-2.3)	5.2 (1.8-11.0)	34.4 (26.1-42.8)	2.4 (-3.6-7.7)
Malta	7.7 (4.3-9.7)	2.0 (-0.7-4.8)	9.5 (1.8-19.5)	12.1 (10.8-13.8)	1.3 (1.0-1.7)	8.2 (6.1-10.6)	0.0 (0.0-0.1)	4.9 (-2.1-12.6)
Marshall Islands	7.1 (1.6-10.5)	-0.4 (-2.2-1.6)	4.8 (0.6-14.1)	14.6 (12.0-17.4)	4.2 (3.0-5.5)	6.6 (2.4-13.4)	13.7 (8.3-20.0)	0.0 (0.0-0.0)
Mauritania	6.1 (1.6-8.9)	0.0 (0.0-0.0)	6.8 (1.2-16.4)	6.5 (5.4-8.0)	1.9 (1.3-2.5)	20.1 (12.2-28.0)	16.8 (10.3-23.9)	2.5 (-2.4-7.1)
Mauritius	4.9 (1.2-7.3)	0.3 (-2.0-2.7)	4.4 (0.6-12.5)	11.7 (10.6-12.9)	3.2 (2.3-4.1)	11.5 (6.0-17.8)	0.3 (0.1-0.6)	1.0 (-1.8-5.5)
Mexico	3.8 (0.8-5.8)	1.8 (0.1-3.5)	3.5 (0.4-11.1)	8.0 (6.9-9.2)	1.5 (1.0-2.1)	14.1 (11.0-17.4)	4.3 (2.5-6.8)	4.2 (2.8-5.6)
Micronesia (Federated States of)	6.7 (1.6-9.9)	0.6 (-0.5-1.7)	5.1 (0.8-14.3)	23.9 (19.9-27.8)	4.0 (2.8-5.3)	7.7 (2.5-17.1)	12.3 (6.5-19.4)	0.0 (0.0-0.0)
Monaco	4.2 (1.1-6.2)	0.0 (-4.9-5.8)	7.1 (1.0-17.3)	12.4 (10.4-14.7)	1.3 (1.0-1.6)	6.9 (4.1-9.8)	0.0 (0.0-0.0)	8.1 (6.2-9.9)
Mongolia	11.4 (5.7-15.0)	2.4 (-0.4-5.1)	2.3 (0.4-8.3)	21.1 (18.9-23.3)	3.7 (2.7-4.8)	24.4 (17.8-30.4)	11.1 (5.5-18.4)	9.9 (4.4-17.3)
Montenegro	5.0 (1.3-7.3)	2.8 (-0.5-6.1)	3.4 (0.7-9.4)	25.8 (23.3-28.4)	3.6 (2.6-4.7)	14.9 (12.0-17.9)	4.6 (1.5-9.5)	9.7 (6.9-13.1)
Morocco	9.9 (5.3-12.8)	0.0 (-0.2-0.3)	7.4 (1.5-17.1)	8.9 (7.6-10.3)	3.4 (2.5-4.4)	25.4 (20.9-29.8)	1.6 (0.7-3.1)	6.2 (4.1-8.7)
Mozambique	5.3 (1.3-7.9)	-1.5 (-3.6-0.6)	1.4 (0.3-5.8)	8.5 (6.9-10.4)	1.5 (1.0-2.0)	3.1 (1.3-6.3)	38.8 (33.4-45.0)	1.5 (-0.1-3.5)
Myanmar	4.0 (0.7-6.1)	1.1 (-0.4-2.6)	2.8 (0.4-8.8)	14.5 (12.5-16.5)	2.9 (2.0-3.8)	11.9 (7.5-16.4)	24.1 (17.8-29.9)	2.6 (1.1-4.2)
Namibia	4.4 (1.0-6.5)	1.0 (-1.7-4.0)	4.6 (0.7-12.6)	9.9 (8.5-11.5)	1.9 (1.3-2.6)	13.9 (8.8-19.6)	13.6 (8.0-20.3)	2.9 (0.3-6.0)
Nauru	8.0 (1.9-11.8)	1.0 (-1.1-3.7)	4.0 (0.4-12.4)	25.2 (21.3-28.9)	4.9 (3.5-6.6)	4.1 (1.2-9.1)	1.4 (0.3-3.7)	0.0 (0.0-0.2)
Nepal	4.1 (0.8-6.3)	0.7 (-1.5-3.1)	3.3 (0.6-9.5)	17.3 (15.5-19.4)	2.6 (1.7-3.5)	18.7 (11.6-25.5)	21.1 (14.3-29.2)	8.4 (5.8-11.6)
Netherlands	4.3 (1.1-6.3)	2.3 (-1.2-6.3)	3.3 (0.3-10.6)	15.0 (13.4-16.6)	1.2 (0.9-1.6)	7.5 (5.5-9.8)	0.0 (0.0-0.0)	10.0 (4.3-16.1)
New Zealand	4.1 (1.1-6.1)	1.9 (-1.7-5.5)	6.1 (0.9-16.0)	10.2 (9.0-11.5)	1.0 (0.8-1.3)	2.0 (0.4-4.1)	0.0 (0.0-0.1)	9.9 (7.0-13.1)

Nicaragua	4.2 (0.9-6.3)	1.3 (0.1-2.5)	1.9 (0.3-7.3)	7.2 (6.1-8.4)	2.1 (1.5-2.7)	9.9 (5.7-15.1)	17.1 (11.1-22.7)	0.4 (-0.3-1.4)
Niger	5.2 (1.3-7.7)	-0.7 (-1.3--0.1)	2.4 (0.3-7.7)	3.2 (2.5-4.1)	1.7 (1.2-2.2)	4.5 (1.2-11.6)	37.9 (27.3-49.3)	2.3 (-2.5-6.6)
Nigeria	5.1 (1.2-7.7)	1.6 (-0.3-3.6)	2.8 (0.4-8.9)	3.5 (2.9-4.3)	1.4 (1.0-1.8)	16.7 (11.0-22.8)	19.8 (13.8-26.3)	0.3 (-0.3-0.9)
Niue	5.3 (1.3-7.8)	0.4 (-1.8-2.8)	5.2 (1.0-13.6)	13.4 (11.3-15.6)	4.1 (3.0-5.3)	4.0 (0.9-9.2)	0.9 (0.2-2.2)	0.3 (-0.7-1.7)
North Macedonia	5.4 (1.8-7.7)	2.0 (-0.6-4.6)	3.0 (0.6-8.5)	20.7 (18.9-22.5)	3.8 (2.7-4.9)	19.7 (16.7-22.6)	3.9 (1.5-7.9)	10.9 (6.9-15.1)
Northern Ireland	4.3 (1.1-6.3)	0.4 (-3.6-5.5)	9.8 (2.1-19.8)	13.1 (11.6-14.9)	1.4 (1.0-1.9)	4.1 (1.9-6.4)	0.0 (0.0-0.0)	
Northern Mariana Islands	6.0 (1.4-8.9)	-0.4 (-2.1-1.8)	4.8 (0.7-13.6)	18.4 (15.8-21.1)	3.7 (2.6-4.9)	6.6 (3.5-10.3)	3.5 (0.9-8.3)	0.1 (-0.1-0.4)
Norway	5.0 (1.6-7.2)	0.1 (-3.2-3.5)	5.7 (0.8-15.3)	10.3 (9.2-11.5)	1.1 (0.8-1.4)	2.5 (0.9-4.3)	0.0 (0.0-0.0)	9.2 (4.8-15.0)
Oman	6.7 (1.6-9.8)	-0.2 (-0.6-0.3)	7.4 (1.3-17.8)	9.7 (8.5-11.2)	2.9 (2.1-3.6)	31.6 (24.9-37.9)	0.1 (0.0-0.3)	2.4 (-3.5-7.7)
Pakistan	9.4 (4.8-12.3)	0.3 (-0.1-0.6)	4.0 (0.7-10.8)	12.8 (10.9-14.9)	3.3 (2.4-4.3)	19.6 (13.8-25.4)	19.1 (12.9-25.5)	4.1 (-0.1-8.1)
Palau	6.5 (1.6-9.6)	0.0 (-2.4-2.8)	4.3 (0.6-12.6)	17.1 (14.4-19.8)	4.8 (3.3-6.2)	4.2 (0.0-10.2)	0.0 (0.0-0.0)	0.0 (-0.1-0.0)
Palestine	9.1 (4.7-11.9)	0.7 (0.3-1.1)	6.9 (1.4-16.4)	14.9 (13.2-16.9)	4.4 (3.3-5.5)	23.6 (18.1-28.6)	0.4 (0.2-0.8)	4.5 (0.4-9.0)
Panama	3.5 (0.7-5.3)	1.8 (0.3-3.5)	2.1 (0.3-7.4)	5.7 (4.8-6.7)	1.1 (0.8-1.5)	8.8 (5.6-12.1)	2.1 (0.8-4.1)	0.7 (0.0-1.5)
Papua New Guinea	6.7 (1.6-9.9)	-1.0 (-2.7-0.7)	3.3 (0.4-10.2)	16.1 (13.3-19.2)	4.0 (2.8-5.2)	3.7 (0.9-9.8)	34.7 (28.5-39.6)	1.9 (1.0-3.1)
Paraguay	4.3 (1.0-6.5)	3.0 (0.2-6.0)	3.3 (0.3-10.7)	15.8 (13.6-18.1)	2.4 (1.7-3.1)	8.4 (5.6-12.0)	9.6 (5.0-15.3)	2.0 (-0.2-4.7)
Peru	4.5 (1.0-6.8)	-0.2 (-2.4-2.0)	3.4 (0.4-10.1)	3.7 (3.1-4.4)	1.2 (0.8-1.5)	18.9 (14.1-24.0)	5.2 (2.6-8.7)	6.1 (4.3-8.2)
Philippines	4.3 (0.7-6.6)	2.2 (-0.1-4.5)	1.6 (0.3-6.4)	18.3 (16.3-20.3)	3.8 (2.8-4.9)	12.2 (9.0-15.2)	16.5 (10.8-22.4)	0.2 (0.0-0.4)
Poland	4.7 (1.2-7.0)	2.9 (0.0-5.8)	4.2 (0.9-11.2)	18.2 (16.4-20.0)	2.2 (1.6-2.8)	16.1 (14.1-18.4)	1.7 (0.5-4.0)	9.6 (5.2-14.7)

Portugal	4.1 (1.1-6.1)	4.3 (1.3-7.2)	7.2 (1.0-17.6)	7.2 (6.4-8.1)	1.4 (1.0-1.9)	3.9 (2.3-5.7)	0.1 (0.0-0.2)	8.7 (6.6-10.8)
Puerto Rico	4.6 (1.2-6.7)	1.4 (0.2-2.6)	7.2 (1.1-16.8)	8.2 (6.8-9.8)	1.1 (0.8-1.4)	2.9 (0.7-5.3)	0.0 (0.0-0.0)	0.3 (-0.4-1.5)
Qatar	9.9 (2.7-14.2)	0.0 (-0.8-0.8)	8.1 (1.0-21.8)	15.4 (13.6-17.5)	3.8 (2.9-4.8)	45.2 (40.7-49.1)	0.0 (0.0-0.0)	1.7 (-2.3-5.2)
Republic of Korea	3.5 (0.6-5.4)	4.4 (1.0-7.8)	4.4 (0.5-13.4)	13.7 (12.4-15.1)	1.6 (1.2-2.0)	19.3 (15.9-22.9)	0.0 (0.0-0.0)	7.8 (5.0-10.7)
Republic of Moldova	4.8 (1.2-7.2)	2.4 (-1.1-6.0)	3.1 (0.6-8.7)	16.1 (14.4-18.0)	2.1 (1.5-2.7)	10.6 (6.2-15.4)	1.4 (0.5-3.1)	10.4 (4.6-16.9)
Romania	4.2 (1.0-6.2)	3.4 (0.3-6.4)	4.3 (0.9-10.9)	13.3 (12.0-14.6)	2.6 (1.9-3.4)	10.9 (8.9-13.2)	1.9 (0.6-4.4)	10.3 (6.6-14.2)
Russian Federation	5.1 (1.2-7.5)	2.9 (0.3-5.5)	3.4 (0.7-9.2)	17.5 (15.8-19.4)	2.4 (1.8-3.1)	8.2 (4.7-11.8)	0.3 (0.1-0.7)	11.0 (5.8-19.0)
Rwanda	5.2 (1.3-7.8)	2.2 (-0.8-5.4)	1.4 (0.3-5.7)	13.0 (10.5-15.6)	1.4 (1.0-1.8)	7.7 (3.3-13.7)	31.8 (24.2-37.8)	4.0 (1.4-7.4)
Saint Kitts and Nevis	5.9 (1.7-8.4)	0.2 (-2.0-3.9)	5.9 (0.9-15.3)	6.7 (5.7-7.8)	2.0 (1.4-2.6)	6.0 (2.8-9.8)	0.5 (0.1-1.3)	0.3 (-0.2-1.0)
Saint Lucia	6.4 (2.5-8.9)	2.1 (0.1-4.4)	6.4 (0.9-16.1)	8.2 (7.2-9.4)	1.4 (1.0-1.8)	15.2 (6.6-25.3)	0.9 (0.3-1.8)	0.2 (-0.3-0.7)
Saint Vincent and the Grenadines	5.4 (1.5-7.7)	2.1 (0.3-4.0)	6.2 (0.9-15.6)	7.1 (5.9-8.3)	1.7 (1.2-2.1)	15.2 (5.8-26.3)	1.1 (0.5-2.2)	0.2 (-0.3-0.8)
Samoa	6.4 (1.6-9.3)	-0.3 (-2.0-1.5)	5.2 (0.9-13.8)	21.0 (18.6-23.5)	4.6 (3.4-5.8)	6.3 (1.8-14.2)	22.2 (14.9-28.5)	0.1 (-0.3-0.9)
San Marino	4.4 (1.1-6.5)	3.0 (-3.8-8.2)	6.5 (0.9-16.7)	11.2 (9.4-13.3)	1.3 (1.0-1.7)	5.4 (2.3-8.8)	0.0 (0.0-0.0)	8.3 (6.4-10.3)
Sao Tome and Principe	6.3 (1.5-9.2)	2.0 (-0.4-4.4)	2.9 (0.4-9.3)	4.2 (3.5-5.2)	1.0 (0.7-1.3)	12.5 (6.8-19.8)	21.5 (15.3-27.2)	0.2 (-0.5-1.3)
Saudi Arabia	8.0 (1.9-11.6)	-0.1 (-0.4-0.3)	12.5 (2.5-25.8)	14.2 (12.1-16.6)	4.8 (3.6-6.0)	40.1 (35.6-44.2)	0.1 (0.0-0.2)	2.9 (-0.2-6.0)
Scotland	4.3 (1.1-6.4)	1.6 (-3.2-7.2)	6.5 (0.9-16.2)	16.3 (14.7-18.0)	1.3 (0.9-1.7)	2.7 (0.8-5.0)	0.0 (0.0-0.0)	10.3 (6.1-14.9)
Senegal	5.2 (1.2-7.7)	-0.1 (-0.3-0.2)	3.4 (0.5-10.3)	6.1 (5.0-7.2)	3.0 (2.3-3.9)	10.8 (5.4-18.2)	28.4 (20.8-35.2)	0.6 (-0.5-1.5)
Serbia	4.6 (1.1-6.7)	2.9 (0.6-5.4)	3.3 (0.6-9.1)	19.4 (17.7-21.0)	3.1 (2.2-4.0)	17.0 (14.3-19.9)	3.2 (1.1-7.2)	10.7 (6.2-15.1)
Seychelles	4.5 (0.8-6.8)	2.8 (-0.1-6.0)	3.3 (0.4-10.0)	15.3 (13.3-17.3)	2.9 (2.1-3.7)	12.7 (6.8-19.6)	0.1 (0.0-0.3)	0.1 (-0.2-0.4)

Sierra Leone	4.9 (1.0-7.3)	1.1 (-0.6-3.2)	2.6 (0.4-8.3)	8.4 (6.8-10.2)	2.6 (1.8-3.4)	6.8 (3.0-12.7)	33.0 (25.9-39.6)	0.2 (-0.2-0.8)
Singapore	4.2 (0.8-6.4)	-0.8 (-2.1-0.4)	4.4 (0.5-13.3)	9.5 (8.6-10.6)	1.2 (0.9-1.6)	14.4 (9.2-19.8)	0.0 (0.0-0.1)	0.1 (-0.1-0.3)
Slovakia	5.0 (1.2-7.3)	2.8 (-0.9-6.2)	3.3 (0.7-9.3)	15.4 (14.0-16.8)	2.6 (1.9-3.4)	14.1 (11.8-16.8)	0.1 (0.0-0.4)	9.5 (6.3-13.5)
Slovenia	4.0 (1.0-5.9)	0.0 (-4.1-4.5)	3.9 (0.8-10.3)	14.3 (12.7-16.0)	2.0 (1.5-2.6)	11.4 (9.3-13.6)	0.9 (0.2-2.4)	9.7 (5.7-13.9)
Solomon Islands	6.6 (1.6-9.8)	-1.0 (-2.5-0.5)	3.7 (0.5-11.3)	21.1 (18.0-24.0)	4.7 (3.5-6.3)	3.5 (0.9-8.8)	39.5 (33.6-44.2)	0.1 (-0.1-0.4)
Somalia	5.9 (1.4-8.6)		1.3 (0.3-5.5)	6.6 (4.9-8.5)	1.8 (1.2-2.4)	1.3 (0.3-4.0)	46.3 (36.9-59.5)	0.6 (0.0-1.3)
South Africa	3.6 (0.6-5.7)	3.1 (1.2-5.1)	4.8 (0.8-12.6)	10.4 (9.2-11.7)	2.5 (1.7-3.2)	19.0 (15.4-23.0)	3.7 (1.9-6.0)	5.5 (4.2-7.0)
South Sudan	4.6 (1.0-6.9)	-0.8 (-1.6-0.0)	1.4 (0.3-5.9)	6.5 (5.0-8.4)	1.6 (1.1-2.1)	6.4 (2.8-11.7)	32.8 (26.3-38.8)	0.2 (-0.1-0.7)
Spain	4.2 (1.1-6.1)	2.7 (0.4-5.3)	6.3 (0.9-16.5)	10.7 (9.5-12.0)	1.3 (1.0-1.7)	5.2 (3.5-7.0)	0.1 (0.0-0.3)	8.3 (6.0-10.3)
Sri Lanka	4.2 (0.8-6.4)	2.6 (0.9-4.5)	2.5 (0.3-8.4)	8.2 (7.0-9.6)	2.2 (1.6-3.0)	13.2 (9.1-17.5)	12.3 (6.1-20.9)	0.4 (0.0-0.9)
Suriname	4.6 (1.0-7.0)	1.6 (-0.2-3.4)	6.0 (0.9-15.2)	14.4 (12.6-16.4)	2.9 (2.1-3.7)	15.5 (7.5-25.5)	2.8 (1.1-5.5)	0.2 (-0.3-0.8)
Sweden	4.9 (1.6-7.0)	2.8 (-1.0-6.8)	4.5 (0.5-13.5)	13.6 (12.0-15.3)	0.9 (0.7-1.1)	1.7 (0.5-3.5)	0.0 (0.0-0.0)	9.6 (5.6-15.4)
Switzerland	4.7 (1.3-6.8)	3.0 (-1.4-7.3)	6.4 (0.9-16.4)	12.2 (10.9-13.6)	1.1 (0.9-1.5)	5.3 (3.6-7.3)	0.0 (0.0-0.0)	10.2 (6.5-14.9)
Syrian Arab Republic	9.4 (4.8-12.3)	0.3 (0.0-0.6)	7.2 (1.4-17.0)	17.1 (15.3-18.9)	3.8 (2.8-4.8)	24.2 (20.0-28.6)	0.0 (0.0-0.1)	5.6 (2.5-9.0)
Taiwan (Province of China)	4.8 (1.2-7.2)	2.0 (0.2-3.8)	4.2 (0.7-11.6)	11.5 (10.4-12.8)	2.7 (2.0-3.4)	17.3 (15.0-19.6)	0.8 (0.2-2.1)	2.6 (1.4-4.4)
Tajikistan	10.0 (5.3-12.9)	0.2 (-1.0-1.4)	2.3 (0.4-7.3)	11.2 (9.4-13.8)	2.7 (2.0-3.5)	20.5 (11.1-30.3)	11.4 (6.2-17.2)	11.2 (6.2-17.7)
Thailand	4.0 (0.6-6.2)	3.2 (0.8-5.6)	3.3 (0.4-9.9)	13.5 (12.1-15.1)	2.5 (1.9-3.2)	19.9 (16.4-23.3)	4.7 (2.0-9.2)	0.4 (-0.1-1.0)
Timor-Leste	4.0 (0.6-6.1)	1.1 (-0.7-3.2)	2.5 (0.3-8.3)	14.9 (11.7-18.4)	3.2 (2.2-4.2)	6.2 (2.9-11.3)	29.4 (22.8-34.8)	0.6 (-0.4-1.9)

Togo	5.6 (1.2-8.3)	0.3 (-1.3-2.0)	2.7 (0.4-8.9)	9.3 (7.8-10.8)	2.0 (1.5-2.6)	10.3 (5.2-16.6)	31.0 (24.0-37.3)	0.1 (-0.1-0.4)
Tokelau	5.7 (1.4-8.4)	-0.2 (-1.7-1.4)	5.0 (0.8-13.5)	14.5 (12.1-16.8)	4.2 (3.1-5.5)	3.5 (0.0-9.7)	0.0 (0.0-0.0)	0.1 (-0.1-0.3)
Tonga	5.3 (1.3-7.9)	-0.7 (-2.0-0.6)	5.2 (1.0-13.5)	15.3 (13.3-17.5)	3.6 (2.6-4.6)	7.0 (2.4-15.4)	11.3 (5.7-17.6)	0.5 (-0.4-1.8)
Trinidad and Tobago	5.9 (1.9-8.4)	1.7 (-0.5-3.8)	7.1 (1.1-16.7)	11.2 (9.8-12.7)	2.3 (1.6-3.0)	16.3 (5.6-29.4)	0.0 (0.0-0.1)	0.0 (-0.2-0.4)
Tunisia	8.1 (3.7-10.7)	0.3 (-0.3-0.8)	3.6 (0.6-10.2)	15.2 (13.5-17.0)	4.1 (3.0-5.1)	22.8 (17.3-28.5)	0.1 (0.0-0.2)	5.0 (1.2-9.1)
Turkey	9.3 (5.2-11.8)	0.1 (-0.5-0.6)	6.9 (1.3-16.0)	16.2 (14.6-17.8)	3.2 (2.4-4.0)	19.3 (16.2-22.6)	0.1 (0.0-0.3)	8.4 (6.0-10.8)
Turkmenistan	10.7 (5.7-13.8)	1.6 (-0.8-4.0)	2.7 (0.5-8.7)	16.6 (14.6-18.6)	5.1 (3.7-6.5)	22.1 (13.7-31.7)	0.0 (0.0-0.1)	7.7 (5.8-9.8)
Tuvalu	6.0 (1.4-8.9)	-0.6 (-2.0-0.9)	4.3 (0.6-12.4)	17.6 (14.9-20.5)	4.3 (3.1-5.5)	4.0 (1.7-8.2)	3.4 (1.2-6.7)	0.1 (-0.1-0.3)
Uganda	4.4 (0.8-6.7)	3.5 (0.8-6.4)	1.4 (0.3-5.5)	5.3 (4.2-6.6)	1.2 (0.8-1.6)	6.6 (3.3-11.4)	31.7 (25.9-37.4)	1.3 (-0.1-3.3)
Ukraine	5.9 (1.8-8.4)	1.9 (-0.8-4.8)	2.3 (0.4-7.1)	17.1 (15.1-19.2)	2.6 (1.9-3.3)	11.2 (6.9-16.1)	0.7 (0.2-1.7)	9.9 (6.4-13.9)
United Arab Emirates	10.3 (2.6-14.9)	0.0 (-1.4-1.4)	11.4 (1.5-26.5)	20.6 (17.8-23.7)	5.5 (4.0-7.2)	38.9 (32.1-45.7)	0.0 (0.0-0.0)	3.0 (-4.8-9.8)
United Kingdom	4.4 (1.1-6.4)	0.3 (-3.2-4.0)	6.8 (1.1-16.7)	13.4 (12.1-14.9)	1.2 (0.9-1.6)	5.2 (3.4-7.2)	0.0 (0.0-0.0)	10.8 (5.9-16.2)
United Republic of Tanzania	3.8 (0.6-5.8)	3.3 (0.9-6.0)	1.5 (0.3-5.8)	11.0 (9.1-13.3)	1.9 (1.3-2.5)	5.6 (2.8-9.6)	33.1 (27.5-38.0)	2.1 (0.3-4.3)
United States of America	4.4 (1.1-6.5)	1.8 (-0.7-4.5)	3.5 (0.4-11.1)	15.8 (14.3-17.4)	1.1 (0.8-1.4)	3.9 (2.1-5.9)	0.0 (0.0-0.0)	5.5 (4.2-7.1)
United States Virgin Islands	4.8 (1.2-7.1)	0.6 (-3.5-5.5)	4.0 (0.6-11.1)	9.0 (7.4-10.9)	1.8 (1.3-2.3)	5.7 (2.8-8.8)	0.2 (0.0-0.5)	0.2 (-0.3-0.8)
Uruguay	5.2 (1.5-7.5)	2.5 (-0.2-5.5)	3.3 (0.3-11.4)	13.9 (12.5-15.3)	2.6 (1.9-3.4)	5.6 (3.0-9.1)	0.4 (0.1-1.0)	6.5 (3.6-9.4)
Uzbekistan	10.9 (5.6-14.1)	0.7 (-1.1-2.6)	2.2 (0.4-7.6)	14.8 (13.4-16.2)	3.1 (2.1-4.2)	26.5 (17.1-35.8)	3.1 (1.2-6.2)	9.2 (6.6-11.5)

Vanuatu	6.4 (1.5-9.4)	0.4 (-0.5-1.4)	2.8 (0.3-9.2)	11.8 (10.0-13.8)	3.2 (2.2-4.1)	5.1 (1.4-11.0)	34.4 (26.6-39.9)	0.4 (-0.4-1.6)
Venezuela (Bolivarian Republic of)	3.8 (0.8-5.8)	1.6 (-0.1-3.3)	2.0 (0.3-7.4)	9.5 (8.1-11.0)	2.2 (1.6-3.0)	15.9 (10.6-21.5)	0.1 (0.0-0.2)	1.1 (0.2-2.2)
Viet Nam	3.5 (0.5-5.5)	4.3 (0.7-8.0)	2.3 (0.3-8.0)	17.9 (15.6-20.1)	3.4 (2.5-4.4)	13.3 (9.6-16.7)	11.7 (6.3-18.2)	1.6 (0.0-3.6)
Yemen	10.6 (5.6-13.7)	0.2 (0.0-0.4)	6.0 (1.1-15.1)	18.6 (16.6-20.8)	4.5 (3.4-5.7)	18.6 (9.7-28.2)	15.9 (9.9-22.8)	2.1 (-0.2-4.2)
Zambia	4.0 (0.7-6.1)	1.0 (-1.1-3.2)	2.3 (0.3-7.8)	8.6 (7.0-10.4)	1.9 (1.3-2.5)	9.2 (4.9-14.8)	26.9 (20.7-32.4)	2.2 (-0.7-5.9)
Zimbabwe	4.4 (0.8-6.8)	0.2 (-1.4-2.0)	1.8 (0.3-6.9)	11.8 (9.8-14.0)	2.5 (1.8-3.2)	7.3 (3.8-11.9)	27.6 (22.1-32.6)	2.8 (0.5-5.6)

Supplement Table 11a. Percent of DALYs (with 95% uncertainty intervals) due to intracerebral haemorrhage for high systolic blood pressure, high body-mass index, high fasting plasma glucose, high LDL cholesterol, low glomerular filtration rate, diet low in vegetable, diet low in fruits and diet high in sodium by location for both sexes combined in 2019, all ages

Country, region	High systolic blood pressure	High body-mass index	High fasting plasma glucose	Low glomerular filtration rate	Diet low in vegetables	Diet high in red meat	Diet low in fruits	Diet high in sodium
GBD regions								
High-income Asia Pacific	59.9 (50.1-69.0)	24.4 (13.6-35.8)	16.5 (10.5-24.0)	9.2 (7.6-11.0)	2.2 (0.5-4.2)	9.5 (4.9-13.2)	7.9 (3.9-13.8)	20.8 (8.9-35.7)
Central Asia	57.8 (47.4-67.3)	21.6 (10.7-33.5)	15.8 (10.1-23.0)	8.3 (6.8-9.9)	0.4 (0.2-0.8)	11.7 (6.4-16.0)	7.2 (3.4-12.7)	24.0 (11.6-39.1)
East Asia	58.2 (47.5-67.6)	22.0 (11.0-33.8)	15.8 (10.2-23.1)	8.2 (6.8-9.8)	0.3 (0.2-0.5)	12.0 (6.5-16.3)	7.1 (3.4-12.7)	24.4 (12.0-39.4)
Southeast Asia	49.1 (37.9-59.4)	10.3 (2.2-22.8)	14.3 (9.5-20.9)	9.0 (7.3-10.8)	3.0 (0.2-7.6)	3.5 (0.4-5.8)	8.7 (4.0-16.0)	17.5 (5.1-33.1)
South Asia	54.5 (44.1-64.5)	30.2 (17.5-42.6)	19.6 (12.5-28.4)	10.6 (8.7-12.7)	1.2 (0.2-4.0)	15.2 (9.2-20.1)	5.2 (2.5-9.1)	8.2 (0.5-22.0)
Southeast Asia, East Asia, and Oceania	64.4 (54.9-72.5)	30.2 (19.1-40.8)	17.8 (10.9-25.7)	11.3 (9.3-13.7)	6.1 (1.1-11.5)	4.8 (1.3-7.3)	9.4 (4.8-16.1)	13.9 (2.5-28.9)
Oceania	42.6 (31.9-53.1)	19.8 (9.9-31.0)	25.5 (14.2-38.7)	10.1 (8.3-12.3)	9.2 (2.7-15.3)	4.5 (0.6-7.3)	13.2 (7.0-20.8)	12.6 (1.6-27.4)
Australasia	71.6 (61.2-79.7)	33.6 (22.2-44.4)	16.2 (9.7-23.7)	11.3 (9.2-13.8)	7.0 (1.4-12.9)	2.2 (0.3-3.8)	9.8 (4.8-16.9)	14.4 (2.6-29.7)
Central Europe, Eastern Europe, and Central Asia	55.4 (44.2-64.9)	31.1 (17.7-43.8)	22.3 (13.8-32.2)	11.2 (9.1-13.5)	6.0 (0.8-11.6)	6.1 (1.1-9.6)	8.4 (3.8-15.6)	12.8 (1.8-27.2)
Central Europe	67.8 (57.0-76.4)	40.7 (28.5-51.6)	22.8 (14.7-32.6)	11.1 (9.3-13.1)	5.9 (0.7-11.3)	4.0 (0.5-6.6)	7.6 (3.5-14.0)	13.4 (1.8-27.9)
Eastern Europe	59.6 (49.0-68.4)	32.0 (20.0-44.0)	17.4 (10.9-25.8)	11.1 (9.2-13.2)	4.7 (0.3-9.8)	2.0 (0.3-3.5)	10.3 (5.1-18.0)	12.4 (1.8-26.1)
Western Europe	59.4 (49.3-68.8)	25.3 (14.4-36.4)	21.5 (13.1-31.3)	10.8 (8.9-13.0)	5.4 (0.5-10.6)	4.8 (0.7-7.6)	11.7 (6.1-19.3)	13.0 (1.6-27.8)

Central Latin America	52.1 (41.5-62.0)	33.5 (20.4-45.6)	15.0 (9.4-21.3)	12.0 (9.8-14.5)	6.4 (1.0-12.2)	7.7 (2.3-11.4)	7.7 (3.8-13.5)	13.2 (2.1-28.5)
Tropical Latin America	61.0 (50.1-70.7)	30.5 (18.0-42.3)	30.7 (18.4-43.6)	12.0 (10.0-14.2)	6.8 (1.2-12.4)	0.9 (0.2-1.6)	11.4 (5.9-18.7)	13.5 (1.8-28.8)
Southern Latin America	50.4 (40.1-60.5)	37.2 (24.9-49.4)	16.6 (10.6-24.3)	12.2 (10.2-14.5)	7.3 (1.4-13.4)	5.4 (0.8-8.5)	6.0 (2.8-10.6)	12.4 (1.7-26.9)
Andean Latin America	63.2 (52.3-71.8)	13.4 (5.1-23.7)	22.7 (12.6-35.4)	10.5 (8.8-12.5)	8.9 (2.7-15.1)	3.7 (0.5-6.2)	13.8 (7.4-21.4)	13.5 (1.9-28.5)
Latin America and Caribbean	64.9 (53.9-74.0)	18.9 (9.1-30.0)	19.5 (11.4-30.2)	10.8 (8.7-13.1)	2.6 (0.2-6.9)	9.9 (3.7-14.3)	9.0 (4.2-16.4)	14.7 (2.0-30.5)
High-income North America	46.7 (37.1-56.7)	35.8 (21.9-50.1)	32.8 (19.7-47.0)	10.1 (8.1-12.3)	8.6 (2.2-15.0)	6.3 (1.5-9.9)	11.1 (5.6-18.7)	7.0 (0.6-18.5)
North Africa and Middle East	67.1 (57.0-75.5)	55.1 (41.5-66.1)	34.1 (21.5-48.0)	12.2 (10.0-14.9)	7.5 (1.6-13.5)	9.7 (3.4-13.9)	13.2 (7.1-20.9)	8.7 (0.6-21.6)
Sub-Saharan Africa	49.9 (38.7-60.0)	53.0 (36.1-68.3)	33.1 (20.4-48.2)	11.6 (9.2-14.5)	9.4 (2.4-16.1)	5.2 (0.7-8.4)	10.5 (5.0-18.9)	6.7 (0.5-18.3)
Central Sub-Saharan Africa	48.8 (38.2-59.5)	46.9 (30.4-63.4)	40.1 (23.0-59.8)	11.6 (9.3-14.2)	10.1 (2.8-16.9)	7.6 (1.9-11.8)	11.7 (6.0-19.8)	7.2 (0.6-18.6)
Southern Sub-Saharan Africa	48.1 (37.6-58.1)	55.7 (40.5-68.8)	34.8 (21.6-50.1)	12.4 (9.9-15.5)	9.8 (2.9-16.5)	7.1 (1.6-11.2)	11.3 (5.7-19.3)	7.5 (0.6-19.6)
Eastern Sub-Saharan Africa	43.8 (33.2-54.6)	30.1 (16.1-44.9)	33.1 (19.3-48.1)	9.4 (7.5-11.6)	8.2 (1.9-14.4)	6.2 (1.2-9.9)	11.0 (5.6-18.6)	7.0 (0.5-18.6)
Western Sub-Saharan Africa	56.2 (45.6-66.6)	53.6 (39.5-65.6)	35.0 (21.3-50.4)	11.9 (9.7-14.5)	10.2 (3.3-16.9)	8.3 (2.6-12.6)	10.0 (4.9-17.5)	2.3 (0.3-8.9)
Countries in alphabetical order								
Afghanistan	43.3 (32.1-54.7)	46.2 (29.6-61.9)	30.8 (18.0-45.2)	11.6 (9.2-14.5)	11.6 (3.8-18.9)	3.8 (0.4-6.5)	11.9 (6.0-20.2)	6.8 (0.5-18.8)
Albania	61.1 (51.3-70.7)	47.8 (35.6-58.9)	31.3 (20.0-44.2)	11.4 (9.4-13.7)	8.5 (2.4-14.3)	6.5 (1.7-10.0)	9.3 (4.6-16.2)	8.9 (0.7-21.8)
Algeria	67.6 (57.3-75.6)	45.5 (30.9-59.4)	29.4 (18.5-42.0)	10.9 (8.8-13.3)	9.3 (2.5-15.9)	10.6 (4.2-15.2)	9.2 (4.3-17.2)	7.8 (0.7-20.1)
American Samoa	66.2 (56.4-74.5)	46.8 (34.1-59.2)	20.6 (13.3-29.1)	9.5 (7.8-11.4)	1.7 (0.3-4.0)	11.1 (5.7-15.1)	8.9 (4.4-15.6)	10.4 (2.1-23.4)

Andorra	65.3 (55.3-73.6)	46.1 (32.9-58.4)	24.6 (14.5-36.2)	9.6 (7.8-11.6)	1.1 (0.3-2.1)	11.7 (6.4-15.8)	9.1 (4.6-15.7)	7.6 (0.7-20.1)
Angola	64.9 (54.7-74.5)	43.7 (31.6-55.5)	27.7 (15.4-41.6)	9.0 (7.3-10.8)	0.2 (0.2-0.2)	8.7 (3.1-12.4)	4.6 (2.0-8.0)	7.4 (0.5-19.9)
Antigua and Barbuda	59.7 (48.8-69.5)	44.6 (30.2-57.4)	26.8 (15.3-40.6)	9.4 (7.7-11.4)	0.3 (0.2-0.5)	6.9 (1.7-10.4)	6.0 (2.7-10.9)	7.0 (0.5-19.9)
Argentina	65.5 (54.4-75.3)	36.5 (25.0-48.2)	31.1 (18.1-46.4)	8.7 (7.1-10.4)	2.9 (0.2-7.0)	5.4 (1.1-8.4)	8.6 (4.0-15.5)	7.8 (0.6-20.5)
Armenia	72.2 (61.8-80.2)	51.9 (38.3-64.2)	27.3 (16.1-40.3)	9.5 (7.7-11.5)	0.3 (0.2-0.8)	15.9 (10.0-20.8)	9.0 (4.2-16.5)	7.9 (0.6-20.9)
Australia	49.3 (37.5-60.7)	47.0 (32.5-60.7)	10.9 (6.9-15.9)	9.2 (7.3-11.4)	0.6 (0.2-2.1)	11.9 (5.6-16.5)	11.1 (5.5-19.3)	7.4 (0.5-20.2)
Austria	70.3 (59.6-78.9)	46.3 (32.2-59.7)	7.9 (4.7-11.7)	9.7 (7.7-12.1)	7.4 (1.3-13.8)	20.4 (13.7-26.4)	16.7 (9.3-25.0)	8.4 (0.6-21.7)
Azerbaijan	61.7 (49.9-72.3)	28.0 (16.1-40.4)	29.1 (16.3-44.7)	8.5 (6.9-10.4)	0.6 (0.2-2.2)	4.2 (0.5-6.8)	10.6 (5.4-18.1)	7.0 (0.5-19.6)
Bahamas	68.3 (57.0-77.2)	56.8 (42.0-69.6)	17.7 (9.7-28.4)	9.9 (7.9-12.3)	0.2 (0.2-0.3)	18.1 (11.8-23.4)	7.8 (3.5-14.8)	8.1 (0.6-21.2)
Bahrain	65.0 (53.6-74.5)	49.2 (34.6-62.6)	26.7 (15.1-39.2)	10.2 (8.2-12.5)	0.2 (0.2-0.2)	11.9 (5.5-16.5)	8.2 (3.6-15.5)	7.5 (0.5-20.2)
Bangladesh	64.1 (53.8-73.2)	42.0 (30.4-54.0)	23.6 (14.4-34.2)	8.0 (6.4-9.5)	0.9 (0.2-2.3)	11.4 (6.3-15.4)	6.9 (3.3-12.4)	18.2 (6.0-33.1)
Barbados	60.5 (46.9-72.4)	26.8 (16.6-38.6)	13.4 (8.3-20.8)	7.1 (5.2-9.0)	0.3 (0.2-0.6)	8.6 (3.7-12.1)	2.4 (1.1-4.0)	18.8 (5.8-34.3)
Belarus	63.6 (52.6-73.0)	36.4 (24.5-49.2)	32.6 (19.2-48.8)	8.1 (6.4-9.7)	0.9 (0.2-3.0)	4.5 (0.7-7.0)	6.3 (2.8-12.0)	18.7 (5.8-34.1)
Belgium	60.1 (48.1-70.6)	40.4 (28.2-52.4)	23.3 (13.1-38.0)	8.3 (6.7-9.9)	0.4 (0.2-1.1)	12.9 (7.7-17.2)	8.2 (3.8-14.9)	18.8 (6.1-33.5)
Belize	64.9 (53.6-75.1)	40.9 (28.2-53.4)	29.1 (16.7-44.0)	8.6 (6.8-10.5)	3.1 (0.2-7.4)	8.1 (2.8-11.7)	6.0 (2.7-11.2)	19.6 (6.6-35.1)
Benin	55.9 (44.3-66.7)	42.0 (29.8-54.5)	33.1 (19.6-47.3)	7.9 (6.4-9.5)	4.0 (0.3-8.5)	10.8 (5.5-14.8)	8.0 (3.6-14.5)	18.8 (6.2-33.9)
Bermuda	59.5 (48.6-69.9)	45.7 (33.6-57.6)	28.0 (16.9-41.8)	8.1 (6.6-9.7)	1.2 (0.2-4.1)	10.0 (4.5-14.0)	8.2 (3.7-15.0)	23.6 (10.0-38.9)

Bhutan	63.9 (52.0-73.5)	43.5 (30.7-56.1)	34.1 (19.7-49.8)	9.0 (7.3-10.8)	0.3 (0.2-0.6)	5.7 (1.1-8.8)	5.3 (2.4-9.4)	18.7 (5.8-34.3)
Bolivia (Plurinational State of)	63.2 (49.7-74.9)	36.4 (25.0-48.8)	31.3 (17.9-48.3)	8.8 (6.6-11.1)	0.3 (0.2-0.6)	10.0 (5.0-13.8)	2.5 (1.1-4.1)	18.3 (5.7-34.0)
Bosnia and Herzegovina	62.1 (52.2-71.1)	46.3 (33.2-58.8)	26.6 (16.4-38.5)	7.5 (6.1-9.0)	0.8 (0.2-2.2)	13.8 (8.3-18.2)	8.7 (4.1-15.4)	13.3 (2.5-27.9)
Botswana	69.3 (58.4-78.6)	43.4 (32.5-54.9)	15.8 (10.4-22.6)	7.7 (6.3-9.2)	0.2 (0.2-0.3)	13.3 (7.8-17.6)	6.9 (3.1-13.1)	19.5 (6.1-35.1)
Brazil	67.8 (56.2-77.8)	40.9 (27.9-53.1)	32.0 (18.4-47.4)	8.6 (6.8-10.5)	2.2 (0.2-6.1)	6.6 (1.8-9.8)	4.5 (2.0-7.6)	19.1 (6.2-34.6)
Brunei Darussalam	65.9 (54.9-75.7)	45.6 (32.3-58.6)	18.9 (11.5-28.8)	8.1 (6.6-9.7)	2.4 (0.2-6.6)	11.0 (5.3-15.3)	8.8 (4.1-15.7)	19.3 (6.4-34.3)
Bulgaria	64.8 (52.5-75.6)	37.9 (26.0-50.4)	21.1 (12.1-33.5)	7.7 (5.9-9.4)	3.4 (0.2-7.4)	11.0 (5.8-15.0)	5.1 (2.4-9.2)	19.9 (7.0-35.6)
Burkina Faso	67.9 (57.8-76.2)	49.6 (36.5-62.1)	16.8 (11.1-23.7)	10.2 (8.4-12.4)	2.5 (0.2-5.9)	10.6 (4.9-14.7)	9.7 (4.8-16.8)	8.3 (0.9-20.8)
Burundi	72.0 (61.2-80.1)	47.3 (33.9-60.6)	12.8 (8.4-18.0)	9.6 (7.7-11.7)	0.6 (0.2-1.9)	15.4 (9.5-20.2)	8.7 (3.9-15.9)	4.6 (0.2-15.2)
CÃ´te d'Ivoire	63.7 (53.3-73.7)	43.5 (32.0-55.4)	14.9 (9.8-21.2)	9.8 (8.0-11.7)	2.3 (0.2-6.3)	9.1 (3.6-13.0)	8.3 (3.9-15.0)	2.8 (0.3-10.5)
Cabo Verde	68.9 (57.1-78.4)	42.7 (30.4-55.3)	15.1 (10.1-21.2)	9.3 (7.6-11.1)	2.1 (0.2-6.0)	9.0 (3.7-12.8)	9.7 (4.9-16.7)	5.6 (0.3-16.7)
Cambodia	70.3 (59.3-79.2)	43.1 (29.5-56.3)	11.9 (7.8-16.8)	9.1 (7.5-10.9)	2.0 (0.2-5.9)	12.2 (6.5-16.6)	8.7 (4.1-15.7)	5.3 (0.2-17.4)
Cameroon	73.3 (62.8-81.8)	48.1 (34.2-61.0)	15.8 (10.1-22.7)	9.3 (7.6-11.2)	3.4 (0.2-7.9)	5.5 (0.9-8.7)	9.0 (4.2-16.4)	4.4 (0.2-15.2)
Canada	67.7 (57.9-75.8)	49.1 (35.7-61.5)	18.3 (12.2-26.2)	10.4 (8.6-12.5)	3.2 (0.3-7.5)	10.8 (5.2-15.0)	9.3 (4.6-16.4)	10.0 (1.2-23.3)
Caribbean	67.2 (56.2-76.4)	52.0 (38.4-64.6)	13.4 (8.9-19.0)	9.9 (7.9-12.1)	0.7 (0.2-2.5)	9.3 (3.0-13.5)	11.3 (5.8-19.1)	4.7 (0.2-15.5)
Central African Republic	53.0 (42.2-63.1)	15.7 (6.7-26.0)	16.8 (10.0-26.6)	9.2 (7.1-11.3)	0.9 (0.2-2.7)	6.8 (2.5-9.8)	8.0 (4.0-13.7)	11.7 (1.8-25.7)
Chad	59.9 (48.7-69.6)	29.8 (16.4-42.9)	34.8 (21.5-48.8)	9.9 (8.1-11.8)	6.6 (1.0-12.3)	6.2 (1.1-9.5)	11.2 (5.8-18.8)	14.0 (2.4-29.1)

Chile	55.3 (43.9-66.0)	13.4 (5.3-23.4)	15.2 (8.9-24.6)	9.7 (7.2-12.0)	0.9 (0.2-2.6)	5.3 (1.4-8.1)	8.4 (4.2-14.3)	10.5 (1.3-23.8)
China	46.3 (36.1-56.4)	21.6 (10.3-33.3)	21.2 (12.5-32.5)	7.7 (6.2-9.2)	1.0 (0.2-3.1)	10.8 (5.7-15.0)	6.8 (3.2-12.5)	15.1 (3.2-30.4)
Colombia	45.7 (35.5-55.9)	31.7 (20.3-43.0)	20.6 (13.0-30.3)	10.3 (8.5-12.3)	1.3 (0.2-4.5)	11.9 (6.1-16.3)	5.3 (2.4-9.1)	13.4 (2.3-28.8)
Comoros	53.6 (42.7-65.1)	34.5 (23.9-45.9)	19.5 (10.7-32.5)	8.1 (5.8-10.3)	3.1 (0.2-7.2)	14.9 (9.9-19.3)	7.0 (3.2-12.9)	3.2 (0.2-11.7)
Congo	53.6 (42.7-65.2)	34.8 (24.2-46.2)	20.2 (11.0-33.8)	8.1 (5.8-10.4)	3.1 (0.2-7.3)	14.9 (10.0-19.4)	7.2 (3.2-13.3)	2.9 (0.2-11.0)
Cook Islands	53.5 (41.6-64.9)	33.4 (22.3-45.3)	16.3 (9.5-26.5)	8.1 (6.0-10.2)	2.9 (0.2-6.9)	14.7 (9.7-19.1)	6.0 (2.8-11.1)	4.6 (0.2-15.0)
Costa Rica	55.9 (44.4-66.8)	27.6 (17.4-39.2)	25.2 (14.9-40.3)	7.0 (4.9-9.1)	2.6 (0.3-5.9)	11.5 (6.7-15.4)	5.5 (2.6-9.6)	5.0 (0.4-15.2)
Croatia	61.3 (50.3-72.2)	34.1 (22.0-46.7)	20.3 (12.0-32.5)	6.7 (5.0-8.3)	2.0 (0.2-5.3)	13.3 (8.1-17.7)	5.2 (2.5-9.4)	4.2 (0.2-14.3)
Cuba	59.2 (46.6-70.9)	27.6 (17.0-39.4)	22.3 (12.3-37.0)	7.7 (5.4-10.0)	2.7 (0.2-6.5)	12.2 (7.2-16.2)	4.4 (2.0-7.6)	7.3 (0.4-19.8)
Cyprus	60.1 (47.1-71.7)	25.7 (15.8-36.9)	21.5 (12.0-36.2)	7.3 (5.0-9.7)	1.5 (0.2-4.5)	12.3 (7.4-16.3)	6.2 (2.8-11.5)	6.4 (0.3-18.4)
Czechia	59.0 (47.6-70.3)	26.1 (16.0-36.8)	30.3 (17.0-46.6)	7.8 (5.7-9.9)	3.7 (0.3-8.0)	9.2 (3.8-13.0)	5.9 (2.7-11.0)	5.2 (0.3-16.2)
Democratic People's Republic of Korea	62.1 (49.3-73.9)	27.2 (17.1-38.7)	18.9 (10.6-30.8)	7.2 (5.2-9.0)	2.2 (0.2-6.0)	12.1 (6.8-16.2)	4.8 (2.3-8.6)	5.3 (0.2-16.7)
Democratic Republic of the Congo	60.9 (47.5-73.2)	27.5 (17.1-39.5)	28.5 (15.8-44.8)	6.2 (4.5-8.0)	3.8 (0.3-7.9)	10.7 (5.5-14.5)	7.4 (3.4-13.5)	5.2 (0.3-15.9)
Denmark	57.1 (44.8-69.0)	26.3 (16.7-37.4)	12.0 (7.4-19.2)	6.8 (4.7-9.0)	3.9 (0.3-8.2)	13.5 (8.4-17.7)	7.4 (3.5-13.5)	4.2 (0.2-14.2)
Djibouti	60.3 (47.4-71.7)	29.6 (18.8-41.8)	30.2 (17.4-47.7)	7.4 (5.1-9.7)	3.4 (0.3-7.5)	12.0 (6.9-16.0)	6.7 (3.0-12.5)	5.5 (0.3-16.4)
Dominica	52.9 (40.5-65.2)	26.3 (15.8-38.1)	21.1 (12.0-35.2)	7.7 (5.1-10.3)	0.4 (0.2-1.0)	12.4 (7.2-16.4)	2.3 (1.0-3.8)	4.8 (0.3-15.2)
Dominican Republic	55.9 (44.1-67.3)	31.5 (20.4-43.8)	21.5 (12.3-35.5)	6.1 (4.4-7.7)	4.4 (0.4-8.9)	13.8 (8.9-18.1)	7.1 (3.3-13.0)	6.0 (0.3-17.3)

Ecuador	59.4 (47.4-69.5)	31.8 (20.7-44.1)	22.6 (12.8-37.1)	7.6 (5.8-9.5)	2.2 (0.2-5.9)	15.2 (10.2-19.6)	6.6 (3.0-12.4)	3.8 (0.2-13.5)
Egypt	60.9 (48.8-71.8)	31.3 (19.4-43.9)	26.0 (14.5-41.4)	8.0 (6.0-10.0)	0.3 (0.2-0.5)	8.4 (3.2-11.9)	2.5 (1.1-4.2)	5.5 (0.3-16.7)
El Salvador	51.2 (39.4-62.7)	23.2 (13.7-34.7)	27.2 (15.3-45.1)	7.0 (4.9-9.2)	1.9 (0.2-5.1)	10.8 (6.0-14.6)	3.4 (1.6-5.8)	6.5 (0.5-17.5)
Equatorial Guinea	61.2 (50.1-72.1)	29.0 (18.7-40.3)	29.8 (17.3-46.8)	7.5 (5.4-9.7)	2.6 (0.2-6.4)	13.9 (9.0-18.3)	6.8 (3.2-12.1)	5.3 (0.3-16.2)
Eritrea	60.3 (49.2-71.1)	27.7 (17.1-39.2)	30.7 (16.9-46.3)	7.4 (5.5-9.3)	0.8 (0.2-2.4)	9.9 (4.8-13.7)	6.6 (3.0-12.4)	7.0 (0.4-19.1)
Estonia	58.5 (45.9-70.2)	26.3 (15.9-38.2)	19.1 (10.8-32.8)	7.5 (5.5-9.7)	3.9 (0.3-8.1)	12.3 (7.2-16.5)	6.0 (2.7-10.9)	4.4 (0.2-14.9)
Eswatini	59.6 (47.2-71.0)	22.9 (13.5-33.6)	25.7 (14.4-42.1)	6.5 (4.7-8.4)	4.4 (0.6-8.6)	10.7 (5.9-14.4)	5.2 (2.5-9.2)	4.4 (0.3-14.2)
Ethiopia	56.0 (44.6-67.0)	27.1 (16.2-39.0)	29.3 (17.8-44.6)	6.9 (5.1-8.8)	0.8 (0.2-2.6)	14.1 (8.9-18.3)	5.4 (2.4-9.8)	5.6 (0.4-16.1)
Fiji	57.1 (45.1-68.7)	29.1 (18.6-40.8)	27.6 (16.1-45.6)	6.7 (4.5-8.8)	1.7 (0.2-5.0)	11.5 (6.3-15.5)	3.5 (1.7-5.9)	3.5 (0.3-11.6)
Finland	58.4 (45.7-70.0)	26.3 (16.1-37.9)	23.2 (13.0-38.5)	7.4 (5.6-9.2)	3.8 (0.4-7.8)	11.0 (5.9-14.7)	6.1 (2.9-11.2)	5.1 (0.3-15.7)
France	48.8 (36.5-61.1)	23.2 (13.7-34.1)	20.4 (11.7-36.5)	7.7 (5.2-10.1)	3.5 (0.3-7.6)	12.5 (7.5-16.4)	6.0 (2.7-11.3)	4.5 (0.2-15.1)
Gabon	52.3 (41.7-62.6)	32.4 (21.2-44.5)	30.1 (18.2-46.3)	6.4 (4.5-8.2)	3.5 (0.4-7.3)	8.6 (4.0-12.1)	7.9 (4.0-13.4)	3.7 (0.3-12.9)
Gambia	55.7 (45.7-64.9)	40.5 (27.2-53.9)	22.6 (13.8-32.8)	7.6 (6.1-9.1)	3.7 (0.3-8.2)	16.4 (10.8-21.0)	5.3 (2.6-9.1)	7.5 (0.4-20.6)
Georgia	53.8 (43.6-63.5)	39.8 (26.3-53.3)	21.2 (13.0-30.9)	7.4 (6.0-8.8)	4.0 (0.3-8.7)	17.3 (11.7-22.1)	4.5 (2.1-7.7)	7.1 (0.3-20.3)
Germany	62.0 (51.2-71.6)	43.8 (29.8-57.0)	28.3 (16.6-41.5)	8.3 (6.6-9.9)	2.1 (0.2-6.2)	13.3 (7.3-17.7)	7.7 (3.5-14.2)	8.8 (0.4-22.9)
Ghana	56.4 (45.8-66.5)	35.8 (23.3-48.6)	18.6 (11.1-28.4)	7.2 (5.7-8.6)	6.0 (1.0-11.2)	15.0 (9.6-19.7)	6.8 (3.0-12.7)	8.0 (0.4-21.2)
Greece	49.3 (39.2-58.8)	44.9 (32.6-56.0)	29.3 (18.9-40.9)	8.8 (6.9-10.6)	3.3 (0.5-6.9)	12.9 (7.6-17.1)	7.0 (3.4-12.5)	6.3 (0.4-18.2)

Greenland	49.4 (37.3-60.6)	35.9 (24.4-47.8)	20.6 (11.1-33.9)	7.3 (5.5-9.1)	2.7 (0.2-6.6)	11.0 (5.9-15.1)	6.3 (2.9-11.6)	6.2 (0.3-18.2)
Grenada	49.3 (39.2-58.8)	45.5 (33.1-56.6)	29.9 (19.4-41.4)	8.8 (7.0-10.7)	3.3 (0.5-6.9)	13.0 (7.8-17.2)	7.0 (3.4-12.6)	6.3 (0.4-18.2)
Guam	58.5 (48.8-67.0)	45.3 (32.4-57.8)	24.5 (15.3-34.5)	9.3 (7.6-11.4)	6.4 (1.2-11.9)	11.3 (6.4-15.4)	6.2 (3.0-10.8)	7.5 (0.6-20.3)
Guatemala	59.7 (49.9-68.2)	37.0 (24.5-48.9)	26.2 (16.2-36.8)	8.7 (7.1-10.8)	6.8 (1.9-12.2)	5.0 (1.0-7.9)	6.3 (3.1-11.2)	4.4 (0.2-15.5)
Guinea	58.6 (47.4-68.7)	41.5 (28.3-54.2)	33.2 (19.9-47.7)	10.4 (8.4-12.9)	6.5 (1.1-12.0)	5.5 (0.9-8.5)	6.1 (2.8-10.9)	4.6 (0.2-15.9)
Guinea-Bissau	61.1 (50.1-70.5)	52.9 (37.7-65.8)	28.6 (17.6-41.8)	9.8 (7.8-12.3)	3.8 (0.2-9.0)	10.8 (4.5-15.3)	8.0 (3.9-14.2)	4.7 (0.2-16.2)
Guyana	61.2 (49.7-71.2)	42.7 (29.1-55.5)	28.5 (17.5-40.7)	9.8 (7.9-11.9)	5.5 (0.7-10.7)	6.6 (1.7-10.0)	9.7 (4.8-16.8)	3.5 (0.2-13.4)
Haiti	55.8 (44.9-65.6)	53.3 (38.8-65.6)	21.6 (13.5-31.6)	9.6 (7.7-12.1)	7.9 (1.8-14.0)	4.3 (0.5-7.1)	1.6 (0.7-2.7)	4.6 (0.2-15.8)
Honduras	51.8 (40.9-62.1)	40.8 (27.9-53.9)	26.6 (16.4-38.5)	8.9 (7.2-10.9)	2.2 (0.2-6.3)	7.5 (2.1-11.1)	5.3 (2.5-9.4)	4.6 (0.2-15.8)
Hungary	57.3 (45.8-67.6)	43.7 (30.4-56.4)	35.4 (22.3-49.6)	10.2 (8.2-12.5)	4.4 (0.3-9.2)	6.2 (1.3-9.6)	0.4 (0.2-0.6)	4.7 (0.2-16.2)
Iceland	57.9 (47.0-67.1)	45.1 (30.4-58.8)	16.2 (9.7-24.3)	8.5 (6.8-10.4)	5.9 (0.6-11.5)	6.1 (1.1-9.6)	2.1 (1.0-3.6)	4.7 (0.2-15.9)
India	57.9 (46.8-67.4)	43.7 (30.2-56.4)	34.8 (20.8-49.9)	10.9 (8.7-13.7)	8.4 (2.2-14.4)	3.8 (0.5-6.4)	5.8 (2.6-10.4)	4.7 (0.2-16.5)
Indonesia	56.9 (44.8-66.7)	45.9 (31.0-60.1)	36.4 (21.5-51.6)	10.0 (7.9-12.8)	6.4 (0.9-12.2)	2.1 (0.3-3.7)	10.2 (4.9-17.9)	4.5 (0.2-15.7)
Iran (Islamic Republic of)	65.0 (54.3-73.9)	24.6 (12.9-36.9)	29.0 (17.2-42.5)	8.2 (6.5-10.1)	9.8 (3.1-16.1)	3.3 (0.4-5.7)	8.9 (4.1-16.1)	4.1 (0.2-14.4)
Iraq	57.1 (46.6-67.1)	46.8 (34.3-59.0)	33.6 (19.7-48.6)	9.7 (7.8-11.8)	5.0 (0.5-10.1)	3.9 (0.5-6.4)	7.0 (3.1-12.9)	4.5 (0.2-15.6)
Ireland	61.0 (50.4-69.9)	43.8 (30.2-57.3)	34.8 (22.1-48.5)	10.1 (8.1-12.5)	8.2 (2.1-14.2)	8.2 (2.3-12.0)	7.4 (3.3-13.7)	4.9 (0.2-16.2)
Israel	58.4 (48.4-67.7)	43.6 (29.7-56.8)	34.7 (21.4-49.9)	9.7 (7.8-12.0)	7.5 (1.7-13.4)	5.0 (0.8-7.9)	5.5 (2.5-9.5)	4.7 (0.2-16.4)

Italy	52.6 (41.9-63.0)	45.6 (31.0-59.1)	34.0 (21.3-47.5)	9.9 (7.9-12.5)	7.2 (1.4-13.0)	3.1 (0.4-5.3)	8.1 (3.7-14.9)	4.6 (0.2-16.2)
Jamaica	65.1 (54.2-74.7)	49.2 (35.5-61.8)	36.0 (22.6-50.7)	9.9 (8.0-12.3)	7.5 (1.8-13.2)	4.5 (0.6-7.3)	9.6 (4.7-16.7)	5.2 (0.2-17.3)
Japan	45.0 (36.4-53.9)	48.9 (35.5-61.2)	17.1 (10.7-24.8)	7.7 (6.3-9.3)	7.2 (1.6-12.8)	7.5 (2.5-11.0)	6.7 (3.1-12.1)	6.7 (0.3-18.9)
Jordan	40.9 (30.9-51.4)	47.0 (33.9-59.7)	20.0 (12.1-29.4)	7.6 (6.2-9.0)	7.9 (2.0-13.6)	9.9 (4.0-14.1)	7.8 (3.6-14.5)	6.7 (0.3-19.2)
Kazakhstan	43.0 (34.0-52.2)	54.2 (41.6-65.1)	21.3 (13.1-31.0)	8.6 (7.0-10.4)	7.3 (1.8-13.1)	9.8 (4.0-13.9)	3.7 (1.7-6.1)	6.8 (0.3-19.0)
Kenya	48.9 (40.2-57.7)	46.8 (32.4-59.8)	12.6 (7.7-18.9)	7.3 (6.0-8.8)	6.5 (1.0-12.2)	4.5 (0.6-7.2)	7.8 (3.6-14.5)	6.7 (0.3-18.7)
Kiribati	57.6 (48.5-66.2)	43.6 (30.2-56.4)	29.8 (18.8-41.0)	12.3 (10.0-14.9)	6.0 (0.9-11.3)	8.3 (3.2-11.8)	6.9 (3.3-12.3)	8.8 (1.0-22.0)
Kuwait	52.3 (42.1-61.9)	39.2 (26.7-52.0)	28.1 (16.8-41.4)	10.4 (8.5-12.4)	6.0 (0.9-11.3)	8.2 (2.6-11.9)	5.9 (2.7-10.7)	13.7 (3.2-28.0)
Kyrgyzstan	67.3 (57.6-76.1)	42.7 (29.2-55.7)	28.4 (17.7-41.0)	13.1 (10.8-15.6)	7.2 (1.6-12.9)	7.1 (1.8-10.6)	5.7 (2.6-10.2)	10.1 (0.8-24.4)
Lao People's Democratic Republic	55.8 (45.4-65.0)	41.5 (28.1-53.6)	28.0 (17.1-39.8)	11.6 (9.5-14.2)	5.9 (0.9-11.3)	3.1 (0.4-5.2)	8.2 (3.8-15.1)	8.6 (0.6-21.7)
Latvia	57.9 (48.9-66.6)	37.0 (23.5-50.1)	27.4 (17.2-39.3)	10.8 (8.8-13.0)	6.4 (1.0-12.0)	3.1 (0.3-5.3)	8.8 (4.0-16.0)	8.2 (0.5-20.6)
Lebanon	56.7 (45.3-66.6)	39.0 (24.5-52.2)	30.1 (18.0-43.5)	11.0 (9.0-13.3)	6.7 (1.1-12.3)	4.0 (0.5-6.6)	8.1 (3.6-15.1)	8.8 (0.6-22.4)
Lesotho	56.4 (47.3-65.5)	48.1 (33.9-60.6)	30.6 (19.7-42.2)	13.4 (10.9-16.5)	5.3 (0.6-10.4)	10.7 (5.2-14.8)	6.2 (2.9-10.8)	6.2 (0.3-18.4)
Liberia	60.9 (49.3-70.4)	39.3 (26.3-52.5)	31.7 (18.9-45.6)	13.2 (10.7-16.2)	9.2 (3.0-15.2)	2.6 (0.3-4.5)	12.6 (6.7-19.9)	9.4 (0.6-23.1)
Libya	59.2 (49.1-68.6)	39.6 (27.0-52.2)	30.7 (18.8-44.6)	11.6 (9.4-14.0)	7.6 (1.8-13.3)	9.0 (3.6-12.9)	8.1 (3.8-14.7)	9.9 (0.8-23.8)
Lithuania	62.8 (52.7-72.6)	42.9 (28.6-56.6)	30.1 (17.8-43.3)	12.3 (10.0-14.9)	6.3 (1.0-11.9)	8.2 (2.4-12.1)	7.2 (3.3-13.3)	10.4 (0.8-25.0)
Luxembourg	60.8 (50.8-69.6)	48.8 (35.9-61.5)	21.5 (13.3-31.2)	7.8 (6.3-9.4)	6.4 (1.0-12.0)	16.2 (10.4-21.2)	5.5 (2.6-9.6)	7.8 (0.5-20.8)

Madagascar	60.9 (50.7-69.7)	48.9 (36.0-61.5)	21.4 (13.3-31.2)	7.7 (6.3-9.4)	6.4 (1.0-12.0)	16.2 (10.4-21.3)	5.5 (2.6-9.6)	7.8 (0.5-20.8)
Malawi	60.2 (49.0-69.4)	45.7 (32.0-59.1)	22.3 (12.9-32.7)	8.6 (6.9-10.7)	7.5 (1.6-13.4)	15.6 (9.5-20.6)	6.5 (3.0-12.0)	7.6 (0.3-21.2)
Malaysia	56.0 (46.6-64.0)	50.5 (38.4-61.2)	21.8 (13.9-30.9)	8.7 (7.1-10.5)	2.7 (0.8-5.1)	4.6 (0.8-7.3)	6.3 (3.3-10.6)	2.2 (0.3-8.6)
Maldives	55.1 (44.2-64.6)	52.5 (39.6-63.4)	25.5 (16.3-36.1)	9.6 (7.7-11.5)	2.7 (0.2-7.3)	4.8 (0.6-7.7)	6.4 (2.9-11.3)	2.2 (0.2-8.7)
Mali	63.8 (52.2-73.3)	64.6 (50.7-75.1)	29.4 (17.6-42.8)	9.7 (7.9-11.8)	0.8 (0.2-2.8)	7.4 (1.8-11.6)	4.5 (2.0-8.2)	2.7 (0.2-10.5)
Malta	46.4 (36.6-55.6)	50.0 (37.5-61.4)	15.9 (9.1-25.2)	6.9 (5.2-8.8)	0.1 (0.1-0.2)	4.7 (0.7-7.6)	3.6 (1.8-6.4)	1.9 (0.2-7.4)
Marshall Islands	54.3 (45.8-62.9)	47.1 (35.4-57.9)	22.1 (14.2-31.6)	9.0 (7.4-10.8)	0.6 (0.2-1.8)	4.5 (0.7-7.2)	3.1 (1.6-5.2)	2.5 (0.3-9.3)
Mauritania	64.9 (55.1-72.7)	55.6 (41.1-68.0)	24.1 (15.1-33.7)	9.2 (7.5-11.2)	0.9 (0.2-3.3)	2.2 (0.2-3.9)	9.1 (4.2-16.4)	2.5 (0.2-9.6)
Mauritius	58.3 (47.5-67.4)	58.4 (45.4-69.0)	25.6 (16.7-36.0)	10.6 (8.7-12.8)	2.5 (0.2-7.0)	4.8 (0.7-7.8)	8.2 (3.8-15.4)	2.4 (0.3-9.3)
Mexico	57.1 (45.8-67.2)	66.6 (53.7-75.8)	23.9 (15.1-34.7)	8.2 (6.7-10.0)	0.4 (0.2-1.5)	12.2 (5.2-17.1)	8.8 (3.9-16.4)	5.4 (0.2-16.7)
Micronesia (Federated States of)	59.4 (50.2-68.1)	49.6 (36.7-61.0)	26.5 (16.7-38.1)	10.4 (8.4-12.6)	0.2 (0.2-0.3)	7.6 (2.2-11.4)	3.4 (1.5-5.7)	2.2 (0.3-8.7)
Monaco	63.0 (53.5-71.1)	59.1 (46.4-69.8)	25.0 (15.2-37.7)	8.4 (6.8-10.2)	2.4 (0.2-6.9)	5.3 (0.8-8.6)	7.3 (3.2-13.7)	2.4 (0.2-9.1)
Mongolia	66.5 (56.1-75.3)	48.4 (34.8-61.7)	27.9 (16.8-40.6)	9.8 (8.0-12.0)	1.2 (0.2-4.1)	5.4 (0.8-8.5)	3.8 (1.7-6.4)	2.4 (0.2-9.4)
Montenegro	51.4 (41.5-60.0)	45.4 (32.8-57.5)	30.0 (18.5-42.8)	10.2 (8.2-12.4)	2.1 (0.2-6.0)	3.0 (0.3-5.0)	7.8 (3.6-14.0)	2.2 (0.2-8.7)
Morocco	57.3 (47.4-66.0)	58.3 (46.2-68.3)	22.2 (14.0-31.7)	8.5 (7.0-10.2)	2.0 (0.2-6.0)	9.4 (3.5-13.6)	4.0 (1.9-6.7)	2.2 (0.2-8.4)
Mozambique	57.4 (45.7-68.5)	75.7 (62.7-84.1)	25.8 (14.5-40.5)	7.7 (6.1-9.7)	0.2 (0.2-0.5)	10.2 (3.2-15.2)	3.1 (1.2-5.8)	3.0 (0.2-11.2)
Myanmar	59.0 (46.2-70.2)	71.3 (57.4-80.9)	24.3 (14.8-35.7)	9.6 (7.6-12.0)	3.8 (0.2-9.3)	5.2 (0.7-8.6)	9.3 (4.2-17.2)	2.7 (0.2-10.5)

Namibia	55.4 (45.9-64.1)	48.3 (34.6-60.9)	25.7 (15.5-36.7)	9.2 (7.5-11.1)	3.8 (0.2-8.4)	4.8 (0.7-7.7)	6.5 (2.9-12.2)	2.3 (0.2-8.9)
Nauru	56.7 (46.8-66.6)	47.5 (34.2-59.8)	29.1 (18.3-42.2)	10.3 (8.3-12.4)	0.6 (0.2-2.0)	4.4 (0.6-7.2)	5.6 (2.6-9.5)	2.3 (0.3-9.0)
Nepal	58.5 (48.1-67.7)	48.2 (34.1-60.2)	20.3 (12.8-30.1)	10.4 (8.3-12.6)	0.2 (0.2-0.2)	4.6 (0.7-7.4)	1.6 (0.8-2.6)	1.6 (0.3-6.8)
Netherlands	64.0 (49.9-76.2)	79.6 (67.0-87.6)	22.8 (12.7-34.5)	8.0 (6.0-10.3)	3.1 (0.2-8.9)	10.2 (2.7-15.3)	8.0 (3.5-14.4)	3.4 (0.2-12.6)
New Zealand	50.2 (40.0-59.6)	32.8 (20.1-45.2)	15.9 (9.0-24.4)	7.4 (6.0-8.9)	8.3 (2.2-14.4)	3.4 (0.4-5.8)	11.7 (6.1-19.2)	2.2 (0.2-8.7)
Nicaragua	58.7 (48.2-67.6)	25.5 (15.3-36.1)	24.3 (14.6-34.8)	9.2 (7.6-11.1)	6.7 (1.5-12.2)	1.6 (0.4-2.7)	15.1 (8.7-22.8)	7.7 (0.6-20.6)
Niger	52.3 (40.8-63.2)	43.4 (29.3-57.1)	25.1 (14.4-39.6)	7.9 (6.3-9.8)	10.9 (3.5-18.0)	6.1 (0.9-9.7)	12.6 (6.5-21.0)	2.1 (0.2-8.7)
Nigeria	58.4 (47.7-68.0)	19.7 (10.7-29.7)	16.6 (10.2-24.9)	7.2 (6.0-8.6)	8.7 (2.4-14.5)	1.2 (0.2-2.1)	13.3 (7.0-20.8)	6.7 (0.3-19.3)
Niue	57.0 (46.2-66.6)	30.7 (18.3-43.1)	20.3 (12.3-30.5)	8.4 (7.0-10.1)	6.5 (1.3-12.1)	2.3 (0.3-4.0)	10.4 (5.3-17.3)	6.8 (0.3-19.4)
North Macedonia	58.2 (47.6-67.4)	25.7 (15.4-36.2)	26.6 (15.8-38.5)	9.8 (8.0-11.8)	5.9 (1.0-11.4)	1.1 (0.3-1.8)	16.2 (9.2-23.8)	8.1 (0.7-21.2)
Northern Ireland	50.9 (40.5-60.5)	23.5 (12.5-35.2)	19.4 (11.9-29.1)	8.0 (6.5-9.7)	6.2 (1.0-11.6)	4.4 (0.6-7.2)	10.0 (4.9-17.2)	6.8 (0.3-19.7)
Northern Mariana Islands	62.7 (52.4-70.7)	31.1 (18.2-44.0)	20.2 (12.7-28.9)	8.5 (6.8-10.3)	8.5 (2.2-14.5)	5.0 (0.8-7.9)	11.8 (6.1-19.3)	7.0 (0.4-19.4)
Norway	59.2 (49.5-67.1)	31.5 (20.9-42.3)	14.0 (8.8-20.6)	6.3 (5.2-7.6)	7.8 (2.1-13.6)	4.0 (0.8-6.2)	10.5 (5.7-16.6)	6.5 (0.3-21.1)
Oman	60.7 (50.2-69.0)	26.8 (15.4-38.9)	18.6 (11.5-26.9)	6.2 (5.0-7.7)	9.7 (3.0-16.0)	3.1 (0.9-4.9)	10.7 (5.5-18.2)	3.4 (0.2-14.7)
Pakistan	63.1 (52.0-71.2)	29.2 (16.7-41.4)	18.4 (10.5-28.0)	6.2 (4.9-7.6)	6.9 (1.2-12.7)	5.2 (0.8-8.3)	9.1 (4.2-16.3)	4.6 (0.2-18.2)
Palau	61.6 (50.3-70.9)	18.5 (8.0-30.5)	18.9 (11.0-28.0)	6.2 (4.8-7.7)	10.7 (3.5-17.4)	10.8 (4.3-15.3)	11.6 (5.9-19.3)	4.6 (0.2-18.7)
Palestine	67.1 (55.8-75.8)	46.5 (33.7-57.6)	19.7 (12.1-28.8)	6.8 (5.5-8.3)	10.3 (3.3-16.8)	5.2 (0.7-8.4)	10.3 (5.1-18.1)	4.8 (0.2-19.6)

Panama	59.3 (48.5-68.0)	25.0 (13.4-37.4)	18.4 (11.6-26.2)	6.2 (5.0-7.6)	10.4 (3.3-16.8)	1.3 (0.3-2.3)	11.4 (5.8-19.0)	2.7 (0.2-12.9)
Papua New Guinea	65.5 (56.0-73.8)	48.7 (36.7-59.9)	20.3 (12.3-30.5)	6.5 (5.3-7.9)	8.6 (2.2-14.6)	6.9 (1.8-10.6)	6.0 (2.9-10.8)	4.7 (0.2-18.3)
Paraguay	63.0 (52.7-72.4)	47.4 (33.8-60.4)	25.6 (15.3-37.4)	7.1 (5.7-8.7)	8.7 (2.3-14.8)	12.2 (5.9-16.7)	4.1 (1.8-6.8)	4.7 (0.2-18.3)
Peru	57.9 (48.3-66.1)	28.1 (17.4-39.4)	12.2 (7.5-18.2)	6.2 (5.1-7.5)	8.9 (2.5-15.1)	3.7 (0.6-6.0)	11.0 (6.2-17.2)	8.9 (0.4-25.7)
Philippines	57.8 (47.5-67.3)	18.2 (8.3-29.7)	11.1 (6.7-17.1)	6.0 (4.9-7.3)	9.3 (2.9-15.6)	1.1 (0.3-1.9)	5.3 (2.4-9.1)	8.7 (0.3-26.3)
Poland	60.4 (50.3-69.5)	31.4 (19.5-42.7)	12.2 (6.5-20.3)	6.4 (5.3-7.7)	10.0 (3.1-16.0)	2.4 (0.3-4.2)	7.5 (3.3-14.4)	9.7 (0.3-28.5)
Portugal	62.1 (50.8-71.3)	31.1 (18.3-44.0)	12.4 (7.3-20.1)	6.7 (5.4-8.2)	7.4 (1.4-13.7)	6.8 (1.3-10.4)	15.4 (8.3-23.3)	8.7 (0.3-26.2)
Puerto Rico	52.4 (41.5-62.6)	22.1 (11.4-33.4)	11.7 (6.9-18.2)	6.4 (5.1-7.8)	10.0 (3.0-16.4)	3.6 (0.4-6.2)	11.6 (5.9-19.4)	8.8 (0.3-26.2)
Qatar	46.7 (38.1-55.5)	21.2 (11.3-32.2)	10.0 (6.4-14.6)	5.9 (4.9-7.1)	8.9 (2.6-14.9)	3.0 (0.4-5.1)	15.1 (8.5-22.5)	8.8 (0.4-25.6)
Republic of Korea	60.6 (50.8-69.0)	38.7 (27.0-49.6)	12.5 (7.8-18.3)	6.3 (5.2-7.7)	6.7 (1.3-12.4)	5.3 (1.1-8.3)	10.4 (5.5-17.5)	5.8 (0.6-17.3)
Republic of Moldova	59.9 (48.3-69.3)	29.0 (16.4-41.5)	9.3 (5.8-13.8)	6.1 (4.9-7.6)	10.4 (3.4-17.1)	4.6 (0.6-7.5)	12.0 (6.2-19.8)	8.7 (0.3-26.6)
Romania	66.2 (56.9-74.1)	28.7 (16.5-41.0)	17.2 (9.4-27.2)	6.2 (5.1-7.5)	9.0 (2.6-15.1)	2.4 (0.3-4.2)	9.5 (4.6-16.7)	8.8 (0.3-26.5)
Russian Federation	58.1 (46.7-69.1)	41.5 (28.3-53.6)	30.1 (19.5-42.2)	14.6 (11.8-17.8)	5.1 (0.4-10.2)	4.9 (0.7-7.9)	12.5 (6.5-20.4)	13.9 (1.9-29.2)
Rwanda	69.1 (58.5-77.1)	27.8 (15.7-39.9)	14.9 (8.6-22.9)	6.5 (5.2-8.0)	9.7 (3.0-16.1)	2.4 (0.3-4.2)	13.7 (7.3-21.5)	9.0 (0.3-27.2)
Saint Kitts and Nevis	54.7 (44.4-64.1)	25.4 (13.8-38.2)	15.3 (7.9-25.7)	6.2 (5.1-7.5)	8.4 (2.2-14.3)	2.5 (0.3-4.4)	0.3 (0.2-0.4)	9.4 (0.3-27.6)
Saint Lucia	65.2 (53.8-74.4)	45.1 (31.6-57.6)	27.2 (17.1-39.1)	12.6 (10.2-15.2)	8.6 (2.2-14.9)	3.6 (0.5-6.2)	9.1 (4.3-16.3)	10.1 (0.9-23.2)
Saint Vincent and the Grenadines	55.0 (44.5-64.8)	11.5 (3.3-23.3)	11.7 (6.6-18.3)	6.0 (4.8-7.4)	10.5 (3.3-17.1)	6.6 (1.3-10.1)	14.4 (7.7-22.1)	8.5 (0.3-25.5)

Samoa	65.6 (55.8-73.4)	35.3 (24.4-46.1)	11.2 (6.5-18.1)	6.3 (5.2-7.6)	7.8 (2.0-13.7)	3.1 (0.3-5.3)	8.1 (3.7-14.8)	12.5 (0.5-32.0)
San Marino	58.3 (48.2-67.1)	29.5 (17.5-41.4)	15.6 (9.1-23.9)	5.9 (4.8-7.1)	9.0 (2.7-14.9)	3.9 (0.5-6.5)	3.5 (1.6-5.9)	8.5 (0.2-25.6)
Sao Tome and Principe	48.8 (38.3-58.6)	35.4 (23.4-47.4)	14.1 (8.3-21.8)	6.5 (5.3-8.0)	9.4 (2.9-15.7)	4.1 (0.5-6.7)	16.0 (8.9-23.7)	8.4 (0.3-25.8)
Saudi Arabia	66.9 (57.0-74.7)	50.2 (38.7-60.9)	24.4 (14.9-34.7)	8.2 (6.7-9.8)	7.9 (2.0-13.9)	8.9 (3.5-12.8)	13.6 (7.4-21.2)	4.9 (0.2-19.1)
Scotland	67.7 (57.2-75.8)	50.7 (38.4-62.2)	23.7 (14.1-34.9)	8.2 (6.7-10.1)	8.6 (2.2-14.8)	8.3 (2.4-12.3)	14.9 (8.1-22.7)	5.5 (0.2-20.8)
Senegal	58.7 (47.0-68.6)	42.8 (30.0-55.2)	24.3 (13.0-36.1)	7.7 (6.3-9.4)	9.4 (2.8-15.6)	6.3 (1.2-9.8)	14.8 (7.9-22.5)	5.1 (0.2-19.9)
Serbia	61.3 (50.2-70.5)	40.5 (29.3-51.0)	21.5 (12.7-32.3)	7.6 (6.2-9.2)	8.0 (2.0-13.9)	8.0 (2.4-11.9)	13.1 (7.0-20.5)	4.9 (0.2-18.9)
Seychelles	68.7 (58.3-76.7)	54.0 (42.1-64.9)	24.9 (15.3-35.6)	8.4 (6.9-10.1)	7.2 (1.5-13.1)	10.2 (4.4-14.4)	13.1 (7.1-20.8)	4.4 (0.2-18.1)
Sierra Leone	64.5 (53.8-72.7)	53.9 (39.8-66.4)	23.3 (14.5-33.9)	8.2 (6.7-10.2)	9.5 (3.0-15.6)	8.1 (2.3-12.0)	10.7 (5.4-18.1)	5.3 (0.2-20.1)
Singapore	63.5 (52.7-72.3)	35.4 (23.7-47.7)	23.0 (13.5-33.8)	7.2 (5.8-8.9)	10.3 (3.2-16.6)	4.1 (0.5-6.9)	15.3 (8.2-23.0)	7.1 (0.2-24.4)
Slovakia	58.6 (49.0-66.1)	33.2 (22.5-43.8)	12.7 (7.9-18.9)	6.1 (5.0-7.3)	6.2 (1.4-11.3)	3.7 (0.6-6.0)	9.4 (5.0-15.1)	5.4 (0.2-19.5)
Slovenia	57.4 (47.4-65.9)	38.6 (27.0-49.8)	16.9 (9.8-25.4)	6.1 (5.0-7.3)	5.8 (0.8-10.9)	2.1 (0.2-3.7)	11.0 (5.7-17.7)	6.3 (0.2-22.2)
Solomon Islands	49.5 (39.0-58.6)	27.1 (15.6-39.3)	15.1 (8.5-23.6)	5.7 (4.5-7.0)	8.6 (2.7-14.4)	4.9 (0.8-8.0)	14.9 (8.2-21.9)	5.2 (0.2-19.1)
Somalia	60.1 (49.9-68.5)	47.1 (34.6-59.4)	12.2 (7.8-17.3)	7.0 (5.7-8.5)	4.2 (0.3-9.0)	4.1 (0.5-6.8)	6.9 (3.2-12.6)	5.6 (0.2-20.9)
South Africa	67.9 (58.5-76.1)	39.9 (28.6-51.7)	21.0 (13.5-30.4)	6.8 (5.6-8.3)	2.8 (0.3-7.2)	4.5 (0.7-7.2)	10.4 (5.2-17.8)	6.1 (0.2-22.6)
South Sudan	48.6 (38.6-57.9)	20.3 (10.8-30.5)	12.5 (7.6-18.3)	5.3 (4.3-6.5)	9.0 (2.8-14.6)	4.7 (0.7-7.5)	13.3 (7.1-20.0)	5.0 (0.2-18.7)
Spain	63.4 (53.1-71.6)	39.1 (26.9-50.4)	14.3 (8.6-21.8)	6.3 (5.1-7.7)	7.7 (1.9-13.5)	4.9 (0.6-7.9)	8.4 (3.9-15.5)	6.2 (0.2-21.8)

Sri Lanka	63.8 (53.6-71.9)	37.7 (25.6-49.7)	14.4 (8.6-21.9)	6.3 (5.2-7.6)	9.0 (2.7-15.2)	2.2 (0.3-3.8)	16.0 (9.1-23.7)	5.7 (0.2-20.9)
Suriname	63.1 (52.3-72.2)	49.1 (36.1-60.8)	16.7 (10.4-24.8)	6.7 (5.4-8.1)	7.4 (1.5-13.4)	3.0 (0.3-5.2)	4.4 (2.0-7.4)	8.8 (0.2-27.6)
Sweden	53.7 (43.5-62.5)	27.3 (16.6-38.2)	13.2 (8.0-20.2)	5.7 (4.7-7.0)	7.3 (1.8-12.9)	2.7 (0.3-4.7)	7.0 (3.2-12.8)	5.2 (0.2-19.1)
Switzerland	60.6 (50.5-68.9)	29.8 (17.1-42.5)	13.8 (7.9-20.6)	6.4 (5.1-7.7)	10.2 (3.3-16.6)	5.5 (0.8-8.7)	10.9 (5.5-18.6)	5.6 (0.2-21.2)
Syrian Arab Republic	61.5 (51.1-70.2)	46.8 (35.0-57.7)	17.3 (10.4-26.2)	6.3 (5.1-7.7)	9.3 (2.9-15.5)	2.1 (0.2-3.7)	11.2 (5.7-18.5)	5.5 (0.2-20.3)
Taiwan (Province of China)	44.1 (33.8-53.7)	26.4 (15.7-37.4)	12.6 (7.1-19.4)	5.3 (4.1-6.5)	7.5 (2.3-12.7)	6.7 (2.0-10.1)	9.7 (5.0-16.0)	4.7 (0.2-18.0)
Tajikistan	59.7 (49.5-68.4)	44.0 (32.8-54.9)	10.7 (6.6-15.9)	6.8 (5.7-8.2)	8.8 (2.5-14.6)	7.4 (2.1-11.0)	14.6 (8.0-21.8)	5.6 (0.2-21.4)
Thailand	51.1 (41.5-60.2)	21.7 (11.9-32.7)	11.1 (5.6-18.7)	5.3 (4.2-6.5)	4.9 (0.5-9.6)	6.2 (1.5-9.5)	12.0 (6.3-18.4)	4.9 (0.2-18.6)
Timor-Leste	61.9 (52.3-69.5)	30.4 (19.5-41.2)	11.0 (6.7-16.9)	6.1 (5.0-7.4)	4.8 (0.6-9.6)	2.9 (0.4-5.0)	8.7 (4.3-14.7)	4.7 (0.2-18.0)
Togo	68.2 (58.7-76.0)	46.4 (33.8-58.4)	16.1 (10.3-24.1)	7.2 (5.9-8.7)	8.8 (2.4-14.9)	1.4 (0.3-2.5)	4.9 (2.3-8.3)	5.8 (0.2-21.7)
Tokelau	61.7 (51.3-70.0)	33.9 (22.3-44.7)	19.7 (11.7-29.2)	6.2 (5.1-7.5)	6.6 (1.2-12.0)	3.8 (0.5-6.3)	12.5 (6.7-19.7)	5.7 (0.2-20.9)
Tonga	60.2 (49.9-69.1)	25.6 (14.5-36.8)	7.7 (4.7-11.4)	5.5 (4.5-6.9)	7.2 (1.8-12.6)	1.6 (0.2-2.9)	10.6 (5.5-17.0)	5.1 (0.2-19.4)
Trinidad and Tobago	60.5 (50.3-69.5)	34.7 (22.7-46.5)	8.7 (5.6-12.7)	6.5 (5.2-7.9)	9.7 (3.0-15.9)	2.3 (0.3-4.0)	16.0 (9.0-23.5)	5.7 (0.2-21.7)
Tunisia	63.2 (52.7-72.1)	59.7 (47.7-69.7)	35.6 (22.3-50.8)	12.6 (10.3-15.6)	8.1 (2.0-14.0)	8.4 (2.7-12.6)	8.4 (3.9-15.1)	3.5 (0.2-13.1)
Turkey	57.0 (46.1-67.0)	44.3 (31.1-56.8)	22.3 (13.6-33.6)	9.8 (7.9-12.0)	3.2 (0.2-7.7)	11.9 (6.1-16.2)	8.0 (3.9-14.2)	4.9 (0.2-16.4)
Turkmenistan	59.1 (47.6-69.1)	52.3 (39.5-63.9)	34.4 (22.4-47.4)	12.2 (10.0-14.8)	7.1 (1.4-12.8)	8.8 (3.2-13.0)	6.9 (3.2-12.7)	9.5 (0.7-23.2)
Tuvalu	51.1 (39.9-61.8)	45.9 (30.9-59.3)	23.0 (12.9-35.5)	6.9 (5.5-8.4)	1.7 (0.2-5.2)	14.9 (9.2-19.6)	6.4 (3.0-11.6)	6.8 (0.3-19.7)

Uganda	62.6 (51.6-71.8)	54.7 (40.1-67.0)	23.8 (15.4-33.9)	11.6 (9.4-14.3)	6.5 (1.1-12.4)	10.7 (4.7-15.4)	6.4 (3.0-11.5)	8.3 (0.6-21.1)
Ukraine	60.9 (48.6-72.3)	33.5 (21.2-46.5)	19.4 (11.4-32.6)	6.9 (5.1-8.7)	0.3 (0.2-0.8)	14.5 (9.2-18.9)	2.0 (0.9-3.4)	5.0 (0.2-16.1)
United Arab Emirates	66.5 (55.2-75.5)	62.5 (46.5-75.1)	28.7 (16.1-43.3)	12.5 (9.8-15.8)	9.7 (2.4-16.5)	9.4 (2.8-14.3)	10.7 (5.0-19.1)	5.7 (0.4-16.5)
United Kingdom	63.7 (54.0-72.6)	48.9 (35.4-61.6)	36.0 (23.9-50.0)	12.4 (10.1-15.1)	8.0 (2.0-13.8)	7.5 (2.1-11.4)	8.2 (3.9-15.1)	9.4 (0.7-23.3)
United Republic of Tanzania	63.1 (51.1-72.5)	57.4 (42.6-69.9)	33.9 (20.9-49.6)	13.3 (10.5-16.9)	7.4 (1.5-13.2)	9.4 (3.5-13.9)	7.2 (3.3-13.4)	9.6 (0.8-23.8)
United States of America	61.9 (49.9-71.6)	59.6 (45.0-72.1)	37.0 (22.2-52.8)	13.2 (10.4-16.7)	8.7 (2.1-15.2)	9.1 (2.8-13.6)	9.0 (4.3-16.2)	8.5 (0.7-22.1)
United States Virgin Islands	58.6 (47.6-68.5)	46.4 (34.0-57.9)	30.3 (19.7-42.1)	10.7 (8.6-12.9)	7.8 (2.1-13.2)	5.7 (1.3-8.8)	5.5 (2.5-9.9)	4.8 (0.2-16.1)
Uruguay	65.1 (53.8-74.2)	47.8 (32.9-62.1)	34.0 (19.9-48.6)	11.3 (8.9-14.5)	6.7 (1.1-12.5)	6.3 (1.3-9.9)	12.0 (6.3-19.3)	5.1 (0.2-17.1)
Uzbekistan	58.8 (46.3-70.7)	29.5 (18.7-42.2)	22.8 (12.6-37.6)	6.5 (4.5-8.5)	2.2 (0.2-5.6)	11.9 (6.9-16.0)	5.2 (2.4-9.6)	4.6 (0.2-14.9)
Vanuatu	56.4 (45.5-66.0)	47.9 (33.9-60.9)	33.9 (21.0-48.4)	11.9 (9.7-14.4)	8.7 (2.3-14.8)	7.3 (1.8-11.2)	9.3 (4.4-16.5)	8.6 (0.6-21.4)
Venezuela (Bolivarian Republic of)	55.9 (45.3-65.7)	45.9 (30.7-59.4)	34.3 (21.3-48.7)	11.5 (9.5-14.1)	9.4 (2.7-15.9)	6.9 (1.7-10.7)	10.8 (5.5-18.5)	8.0 (0.6-20.8)
Viet Nam	60.7 (48.1-70.4)	49.0 (34.9-61.7)	34.6 (20.6-50.5)	10.3 (8.2-12.6)	4.9 (0.5-10.0)	6.1 (1.5-9.7)	3.7 (1.7-6.7)	5.0 (0.2-17.1)
Yemen	58.2 (46.5-69.4)	32.8 (21.3-45.1)	24.2 (13.5-40.2)	6.5 (4.8-8.2)	3.1 (0.3-6.8)	9.3 (4.6-13.1)	8.1 (4.0-13.8)	3.8 (0.2-13.1)
Zambia	55.7 (44.0-66.8)	35.6 (23.5-48.3)	30.8 (17.7-47.5)	6.8 (5.0-8.6)	3.4 (0.3-7.4)	8.8 (4.3-12.5)	8.1 (4.1-14.0)	2.5 (0.2-10.1)
Zimbabwe	57.8 (47.3-66.9)	34.1 (22.1-45.7)	11.8 (6.9-19.0)	5.9 (4.8-7.3)	8.2 (2.2-14.0)	4.1 (0.6-6.8)	7.9 (3.7-14.6)	8.8 (0.2-25.9)

Supplement Table 11b. Percent of DALYs (with 95% uncertainty intervals) due to intracerebral haemorrhage for diet low in whole grains, alcohol use, low physical activity, smoking, second-hand smoking, ambient particulate matter$_{2.5}$ pollution, household air pollution from solid fuels and low ambient temperature by location for both sexes combined in 2019, all ages

Country, region	Alcohol use	Smoking	Second-hand smoking	Ambient particulate matter$_{2.5}$ pollution	Household air pollution from solid fuels	Low ambient temperature
GBD regions						
High-income Asia Pacific	11.7 (8.3-15.5)	23.2 (21.2-25.2)	4.6 (3.4-5.8)	24.3 (20.2-27.5)	10.5 (6.3-15.9)	6.9 (5.3-8.9)
Central Asia	13.5 (9.6-18.0)	24.1 (21.7-26.6)	4.6 (3.4-5.8)	28.4 (24.1-31.8)	8.2 (4.6-13.3)	9.7 (7.5-12.4)
East Asia	13.7 (9.8-18.2)	24.2 (21.7-26.7)	4.6 (3.4-5.8)	28.8 (24.5-32.1)	7.7 (4.1-12.7)	9.6 (7.5-12.3)
Southeast Asia	9.6 (6.0-13.2)	22.3 (19.7-24.8)	4.9 (3.6-6.3)	18.2 (11.5-25.2)	25.5 (17.8-32.8)	11.5 (7.4-17.0)
South Asia	13.3 (8.6-18.4)	23.7 (21.7-25.8)	3.8 (2.8-4.8)	21.6 (18.7-24.5)	0.9 (0.2-2.5)	4.3 (2.3-6.8)
Southeast Asia, East Asia, and Oceania	7.9 (5.3-10.5)	21.3 (18.9-23.6)	4.5 (3.3-5.9)	15.8 (12.1-19.4)	15.0 (9.5-21.1)	1.1 (0.2-2.2)
Oceania	12.4 (7.9-17.1)	20.8 (18.3-23.5)	4.8 (3.5-6.1)	8.4 (4.2-14.0)	33.2 (26.9-38.6)	0.1 (-0.1-0.3)
Australasia	0.7 (-0.2-2.1)	20.4 (16.8-23.8)	4.9 (3.5-6.5)	15.9 (12.0-20.1)	11.9 (6.4-19.3)	0.7 (0.0-1.8)
Central Europe, Eastern Europe, and Central Asia	12.4 (6.8-18.3)	20.2 (17.6-22.8)	4.8 (3.5-6.3)	8.2 (4.0-13.1)	36.6 (29.9-42.0)	1.8 (-0.2-4.3)
Central Europe	3.8 (1.7-6.2)	18.1 (16.4-19.9)	4.4 (3.2-5.7)	15.9 (11.6-20.1)	0.2 (0.1-0.4)	0.5 (-0.2-1.3)
Eastern Europe	2.9 (-0.2-7.5)	22.4 (20.2-24.4)	4.8 (3.6-6.2)	10.0 (6.7-13.8)	4.5 (1.9-8.6)	0.0 (0.0-0.0)
Western Europe	7.7 (4.4-11.3)	17.2 (14.7-19.6)	3.8 (2.6-5.0)	13.9 (8.7-19.2)	27.5 (20.1-34.0)	2.8 (1.3-4.6)
Central Latin America	15.5 (10.6-20.6)	24.5 (21.7-27.3)	4.8 (3.5-6.3)	14.5 (10.8-18.2)	18.9 (12.0-25.6)	0.2 (0.0-0.5)
Tropical Latin America	12.7 (8.5-17.4)	12.1 (10.2-14.2)	3.0 (2.1-4.0)	15.8 (10.9-20.9)	14.1 (6.9-23.8)	0.4 (-0.1-1.0)
Southern Latin America	17.4 (12.4-22.4)	20.2 (18.0-22.5)	3.2 (2.4-4.2)	23.7 (19.7-27.5)	5.2 (2.2-10.3)	0.5 (-0.1-1.3)
Andean Latin America	7.3 (3.7-11.6)	17.5 (14.2-21.6)	4.2 (2.9-5.5)	6.9 (3.1-12.6)	33.1 (25.9-38.8)	0.7 (-0.4-2.3)
Latin America and Caribbean	19.0 (12.7-24.9)	26.0 (23.6-28.3)	4.2 (3.0-5.5)	16.2 (11.8-20.5)	13.7 (7.4-21.8)	1.8 (0.0-4.1)
High-income North America	3.7 (0.9-6.9)	21.5 (18.6-24.5)	5.4 (3.8-7.1)	5.0 (1.5-11.7)	36.8 (30.1-41.7)	2.0 (1.0-3.1)
North Africa and Middle East	6.2 (3.8-9.1)	18.0 (15.6-20.2)	4.3 (3.0-5.7)	9.7 (3.2-21.0)	9.3 (4.0-16.8)	0.7 (-0.5-2.4)

Sub-Saharan Africa	3.5 (0.1-7.9)	35.5 (32.7-38.1)	6.6 (4.7-8.6)	4.7 (1.4-11.3)	33.0 (25.9-39.0)	0.0 (0.0-0.0)
Central Sub-Saharan Africa	5.9 (1.7-11.0)	19.2 (16.2-22.3)	5.2 (3.7-6.8)	7.6 (2.9-15.3)	15.1 (9.2-22.4)	0.0 (0.0-0.0)
Southern Sub-Saharan Africa	5.8 (3.1-9.0)	28.5 (24.6-32.7)	4.9 (3.4-6.7)	8.9 (3.0-19.1)	13.5 (6.9-21.6)	0.0 (0.0-0.0)
Eastern Sub-Saharan Africa	3.4 (0.4-7.2)	20.8 (17.4-24.4)	5.4 (3.8-7.1)	4.5 (1.1-11.8)	40.0 (32.9-45.4)	2.6 (1.4-4.0)
Western Sub-Saharan Africa	6.1 (2.6-10.1)	26.4 (23.6-29.2)	6.2 (4.6-8.0)	7.5 (2.2-16.3)	25.5 (16.9-32.8)	0.2 (-0.4-1.1)
Countries in alphabetical order						
Afghanistan	2.9 (0.3-5.8)	25.1 (22.7-27.6)	5.8 (4.1-7.7)	4.0 (1.1-10.3)	43.8 (37.3-48.8)	0.1 (-0.1-0.5)
Albania	2.8 (0.4-6.1)	19.6 (17.2-22.2)	5.0 (3.6-6.4)	8.3 (2.8-18.0)	13.0 (6.6-20.7)	0.6 (-0.5-2.3)
Algeria	4.4 (2.0-7.1)	15.5 (13.0-18.1)	4.2 (3.0-5.6)	5.9 (1.6-12.8)	38.4 (29.9-44.2)	0.5 (-0.4-1.9)
American Samoa	16.9 (12.5-21.2)	25.6 (24.0-27.3)	3.8 (2.7-4.8)	16.0 (12.5-19.6)	2.3 (1.1-4.2)	11.2 (7.7-16.0)
Andorra	10.5 (7.2-13.8)	20.0 (18.6-21.3)	4.2 (3.0-5.3)	23.3 (17.0-29.9)	5.0 (2.6-8.5)	10.8 (7.7-14.8)
Angola	7.9 (4.5-11.4)	22.3 (20.5-24.0)	4.4 (3.2-5.6)	26.3 (18.8-33.5)	0.7 (0.2-1.6)	11.3 (7.1-17.4)
Antigua and Barbuda	12.3 (7.5-17.2)	19.7 (16.9-22.7)	5.0 (3.6-6.5)	21.5 (13.4-30.4)	1.8 (0.7-4.0)	9.6 (7.7-11.3)
Argentina	11.9 (7.8-16.1)	22.1 (20.3-23.8)	4.3 (3.2-5.4)	14.4 (10.5-18.7)	7.2 (2.8-13.5)	11.6 (7.7-16.9)
Armenia	12.5 (7.7-17.3)	22.7 (20.7-24.9)	3.9 (2.8-5.2)	18.7 (13.0-25.6)	2.6 (0.9-5.6)	10.6 (7.1-15.4)
Australia	12.5 (8.6-16.6)	27.4 (25.3-29.6)	5.1 (3.6-6.5)	19.5 (12.5-27.8)	10.5 (5.6-16.2)	13.8 (7.3-22.5)
Austria	14.1 (9.2-19.2)	23.9 (21.6-26.1)	4.6 (3.2-6.1)	26.3 (19.1-32.7)	11.9 (5.9-19.7)	12.2 (5.4-21.3)
Azerbaijan	5.8 (3.2-8.7)	15.2 (13.1-17.6)	3.5 (2.5-4.5)	22.8 (12.4-33.7)	12.4 (6.7-18.9)	13.7 (7.5-21.2)
Bahamas	12.7 (8.5-17.1)	22.8 (20.8-24.8)	5.9 (4.2-7.6)	25.0 (15.8-35.5)	0.0 (0.0-0.1)	8.4 (6.3-10.6)
Bahrain	8.1 (4.7-11.5)	16.8 (15.3-18.3)	3.5 (2.5-4.6)	28.2 (18.5-37.8)	3.3 (1.3-6.6)	10.1 (7.2-12.7)
Bangladesh	18.7 (13.8-23.3)	25.3 (23.8-26.9)	3.6 (2.7-4.6)	16.5 (14.2-19.0)	2.8 (1.0-5.8)	11.3 (7.4-15.4)
Barbados	9.0 (5.3-13.3)	18.1 (16.5-19.9)	3.9 (2.9-4.9)	12.7 (10.1-15.2)	6.1 (2.7-11.8)	11.0 (7.9-14.0)
Belarus	12.3 (8.4-16.1)	29.7 (27.6-31.9)	4.2 (3.1-5.3)	20.9 (17.5-24.1)	6.6 (2.7-13.1)	11.7 (7.4-16.3)
Belgium	20.8 (15.3-26.1)	27.2 (25.2-29.2)	3.6 (2.6-4.6)	16.2 (13.8-18.8)	3.2 (1.0-7.3)	11.5 (7.2-16.0)
Belize	19.1 (13.2-24.7)	25.9 (23.9-28.0)	4.5 (3.3-5.8)	15.6 (12.8-18.4)	0.8 (0.2-2.1)	11.0 (6.1-15.9)
Benin	24.7 (18.8-30.6)	24.1 (22.3-25.9)	3.0 (2.2-3.8)	14.2 (11.8-16.9)	0.2 (0.0-0.4)	11.1 (6.7-16.3)
Bermuda	20.4 (15.0-25.8)	27.2 (25.3-29.3)	4.1 (3.0-5.2)	14.5 (12.0-16.9)	2.7 (0.8-6.5)	10.9 (4.7-17.3)
Bhutan	14.4 (9.9-18.6)	30.2 (27.9-32.5)	4.8 (3.5-6.2)	23.3 (19.8-26.5)	4.5 (1.7-9.1)	11.6 (7.4-16.1)

Bolivia (Plurinational State of)	15.5 (10.5-20.9)	29.0 (26.4-31.6)	4.3 (3.2-5.4)	15.9 (13.0-19.0)	4.9 (1.6-10.1)	11.8 (8.5-15.9)
Bosnia and Herzegovina	19.7 (14.2-25.4)	27.0 (24.9-29.2)	3.1 (2.3-4.0)	19.8 (17.3-22.4)	1.9 (0.6-4.6)	11.2 (5.9-17.0)
Botswana	20.1 (14.7-25.5)	22.5 (20.8-24.4)	3.6 (2.6-4.6)	13.6 (11.1-16.1)	2.2 (0.7-5.3)	11.4 (7.3-15.7)
Brazil	15.3 (10.8-20.1)	27.7 (25.6-29.7)	3.9 (2.8-5.0)	19.8 (16.8-23.0)	3.6 (1.2-8.2)	11.5 (6.8-16.3)
Brunei Darussalam	20.6 (15.0-26.3)	22.2 (20.1-24.2)	3.5 (2.6-4.6)	16.7 (14.0-19.6)	0.2 (0.0-0.4)	11.2 (7.5-15.9)
Bulgaria	13.9 (4.3-22.9)	22.6 (20.5-24.8)	3.1 (2.2-4.0)	13.8 (11.4-16.5)	1.0 (0.2-2.7)	11.4 (6.7-16.4)
Burkina Faso	19.7 (14.7-24.7)	29.0 (26.9-31.1)	3.6 (2.6-4.7)	11.6 (7.3-16.0)	0.5 (0.2-1.1)	11.3 (7.0-17.7)
Burundi	22.6 (16.5-28.9)	27.6 (24.8-30.6)	3.3 (2.3-4.4)	15.7 (11.8-19.7)	0.2 (0.1-0.4)	11.0 (6.7-17.2)
CÃ´te d'Ivoire	21.9 (16.0-27.4)	25.4 (23.3-27.5)	2.6 (1.9-3.4)	3.4 (1.3-5.8)	0.8 (0.1-2.7)	10.5 (5.4-18.4)
Cabo Verde	18.7 (13.0-24.1)	22.7 (20.2-25.1)	3.0 (2.1-3.9)	10.0 (7.2-13.2)	0.9 (0.2-2.5)	11.1 (6.5-17.8)
Cambodia	21.9 (15.9-28.0)	21.4 (19.5-23.3)	2.8 (2.0-3.6)	8.6 (5.7-11.7)	0.4 (0.1-1.1)	10.9 (5.8-17.4)
Cameroon	20.1 (14.1-26.1)	23.7 (21.4-26.4)	3.1 (2.2-3.9)	12.7 (7.4-18.2)	1.7 (0.6-3.5)	11.3 (5.0-18.3)
Canada	19.8 (14.7-25.2)	29.4 (27.0-31.8)	3.6 (2.6-4.6)	10.4 (6.0-14.9)	0.3 (0.1-0.9)	11.7 (6.2-19.8)
Caribbean	19.0 (13.3-25.0)	29.0 (26.1-32.0)	3.8 (2.8-5.0)	14.5 (9.0-20.4)	0.9 (0.3-2.1)	10.4 (6.7-14.7)
Central African Republic	15.8 (10.8-20.8)	19.5 (18.1-21.1)	2.3 (1.7-2.9)	13.5 (10.1-17.2)	0.0 (0.0-0.1)	8.8 (6.6-10.8)
Chad	0.6 (-0.4-2.0)	19.3 (16.8-22.1)	3.0 (2.1-3.9)	5.8 (1.6-10.2)	0.1 (0.0-0.3)	0.1 (-0.2-0.5)
Chile	14.1 (8.8-19.5)	19.0 (17.6-20.8)	2.2 (1.6-2.9)	10.2 (6.9-13.8)	0.0 (0.0-0.1)	8.5 (6.8-10.3)
China	21.7 (15.9-27.7)	21.2 (19.5-23.3)	2.5 (1.9-3.2)	23.1 (19.1-27.3)	0.0 (0.0-0.0)	10.1 (6.4-13.8)
Colombia	5.0 (2.1-8.1)	14.9 (13.5-16.4)	2.1 (1.5-2.8)	17.4 (11.4-23.6)	0.0 (0.0-0.1)	0.2 (-0.2-0.5)
Comoros	20.7 (15.0-26.4)	14.6 (13.3-16.1)	1.8 (1.3-2.3)	3.2 (0.8-6.0)	0.0 (0.0-0.1)	6.2 (4.4-8.0)
Congo	20.8 (15.2-26.8)	14.4 (13.0-15.9)	1.8 (1.3-2.3)	3.4 (0.8-6.2)	0.0 (0.0-0.1)	5.0 (3.1-7.0)
Cook Islands	20.0 (14.2-25.7)	15.5 (13.9-17.3)	1.9 (1.4-2.5)	2.6 (0.5-5.3)	0.0 (0.0-0.1)	11.9 (8.3-15.6)
Costa Rica	20.0 (14.9-24.9)	19.0 (17.7-20.5)	2.1 (1.6-2.7)	8.4 (6.2-10.8)	0.0 (0.0-0.1)	11.1 (7.8-14.5)
Croatia	21.3 (15.5-26.9)	21.6 (18.8-24.7)	2.5 (1.9-3.3)	6.4 (3.6-9.3)	0.0 (0.0-0.1)	12.0 (5.3-21.2)
Cuba	21.2 (15.5-26.3)	22.3 (20.5-24.1)	2.6 (2.0-3.3)	9.1 (6.8-11.5)	0.0 (0.0-0.1)	11.4 (7.3-17.0)
Cyprus	22.6 (16.6-28.6)	20.8 (19.2-22.7)	2.0 (1.5-2.5)	9.4 (7.2-11.8)	0.0 (0.0-0.0)	12.1 (6.4-18.4)
Czechia	17.9 (12.5-23.3)	24.3 (22.3-26.2)	3.0 (2.2-3.8)	12.8 (9.9-16.1)	0.0 (0.0-0.1)	5.3 (0.9-10.4)

Democratic People's Republic of Korea	21.9 (15.3-28.2)	24.5 (22.6-26.6)	2.0 (1.4-2.6)	6.8 (4.3-9.7)	0.0 (0.0-0.0)	12.2 (5.3-19.7)
Democratic Republic of the Congo	16.2 (10.9-21.4)	17.0 (15.6-18.6)	1.5 (1.1-1.9)	2.0 (0.4-4.1)	0.0 (0.0-0.0)	11.9 (5.4-21.0)
Denmark	23.6 (17.5-29.7)	17.5 (15.9-19.4)	1.6 (1.2-2.1)	8.2 (6.0-10.5)	0.0 (0.0-0.1)	11.4 (7.1-16.1)
Djibouti	26.6 (20.0-32.7)	19.5 (17.8-21.5)	1.8 (1.3-2.3)	8.8 (6.6-11.3)	0.0 (0.0-0.0)	11.5 (6.3-17.1)
Dominica	15.2 (10.5-19.7)	23.6 (21.8-25.7)	2.9 (2.2-3.7)	10.7 (8.2-13.3)	0.1 (0.0-0.2)	9.7 (7.4-11.9)
Dominican Republic	17.5 (11.9-23.0)	19.0 (17.0-21.2)	2.2 (1.7-2.8)	2.2 (0.4-4.5)	0.0 (0.0-0.0)	14.3 (6.7-23.9)
Ecuador	19.5 (14.3-25.1)	19.9 (18.2-21.7)	2.1 (1.5-2.7)	4.8 (2.5-7.3)	0.0 (0.0-0.1)	12.5 (5.3-20.5)
Egypt	6.0 (2.7-9.7)	19.0 (17.4-20.7)	2.3 (1.7-3.0)	16.0 (13.4-18.8)	0.0 (0.0-0.1)	4.8 (0.2-10.1)
El Salvador	18.0 (13.1-23.4)	16.8 (15.4-18.4)	2.5 (1.9-3.2)	11.5 (9.3-13.8)	0.1 (0.0-0.2)	10.4 (7.9-12.9)
Equatorial Guinea	23.1 (17.0-29.3)	20.4 (17.8-23.2)	2.3 (1.7-3.0)	7.2 (4.8-9.7)	0.0 (0.0-0.0)	11.8 (5.2-19.1)
Eritrea	16.0 (11.2-21.0)	19.9 (18.0-22.1)	2.3 (1.7-2.8)	10.3 (7.7-13.0)	0.0 (0.0-0.1)	5.6 (-2.3-14.5)
Estonia	20.7 (14.9-26.6)	20.0 (18.2-22.0)	2.0 (1.4-2.5)	9.0 (6.7-11.6)	0.0 (0.0-0.0)	11.9 (5.1-19.3)
Eswatini	14.8 (9.2-19.9)	15.4 (14.0-16.8)	1.7 (1.3-2.2)	3.0 (1.1-5.2)	0.0 (0.0-0.0)	12.3 (6.4-20.0)
Ethiopia	20.9 (15.4-26.0)	16.1 (14.6-17.6)	2.5 (1.8-3.1)	5.2 (3.1-7.6)	0.1 (0.0-0.2)	9.7 (7.4-12.0)
Fiji	16.9 (11.8-22.0)	19.7 (17.9-21.7)	2.4 (1.8-3.0)	6.6 (4.4-8.9)	0.1 (0.0-0.3)	10.1 (7.5-12.5)
Finland	21.3 (15.0-27.0)	17.7 (15.9-19.7)	1.5 (1.1-2.0)	2.1 (0.6-4.1)	0.0 (0.0-0.0)	11.9 (6.8-19.0)
France	22.6 (16.3-29.1)	17.4 (15.8-19.4)	1.8 (1.3-2.4)	6.5 (4.4-8.8)	0.0 (0.0-0.0)	12.2 (7.6-18.1)
Gabon	16.8 (11.1-22.1)	19.8 (18.2-21.6)	2.2 (1.6-2.8)	6.7 (4.5-9.2)	0.0 (0.0-0.0)	12.7 (6.9-18.9)
Gambia	20.2 (14.6-25.8)	24.3 (22.4-26.2)	3.7 (2.7-4.8)	13.6 (9.6-18.0)	0.8 (0.3-1.7)	10.5 (7.7-13.9)
Georgia	20.5 (14.8-26.5)	24.9 (22.9-26.9)	3.6 (2.6-4.7)	12.2 (7.9-17.4)	0.7 (0.2-1.6)	10.2 (7.5-13.6)
Germany	19.7 (14.4-25.1)	22.6 (20.6-24.8)	4.0 (2.8-5.2)	20.2 (16.4-24.2)	1.1 (0.3-2.7)	12.1 (8.2-17.3)
Ghana	17.5 (12.3-23.0)	23.2 (21.3-25.1)	3.6 (2.6-4.6)	7.2 (3.7-11.5)	0.5 (0.2-1.2)	7.2 (4.1-10.4)
Greece	16.1 (11.6-20.8)	22.7 (21.0-24.5)	2.1 (1.6-2.7)	4.9 (2.6-7.5)	0.0 (0.0-0.0)	9.0 (6.9-11.2)
Greenland	17.0 (11.8-21.9)	19.5 (17.7-21.3)	1.8 (1.4-2.4)	3.9 (1.8-6.3)	0.0 (0.0-0.0)	13.2 (4.1-23.5)
Grenada	16.1 (11.6-20.8)	22.9 (21.2-24.7)	2.1 (1.6-2.8)	5.0 (2.7-7.6)	0.0 (0.0-0.0)	8.7 (6.8-10.8)
Guam	11.5 (8.2-14.8)	15.3 (14.0-16.7)	2.9 (2.1-3.8)	13.6 (10.7-16.5)	6.7 (4.5-9.3)	2.4 (1.3-3.6)

Guatemala	11.3 (8.0-14.6)	14.5 (12.6-16.3)	2.5 (1.8-3.3)	11.8 (6.6-18.7)	15.7 (10.8-21.0)	0.6 (-0.1-1.6)
Guinea	12.0 (8.1-16.1)	12.1 (10.5-13.8)	2.4 (1.6-3.1)	16.1 (6.2-27.4)	0.3 (0.1-0.6)	0.1 (-0.3-0.7)
Guinea-Bissau	11.3 (4.0-18.2)	11.8 (10.1-14.0)	2.9 (2.1-3.8)	15.3 (4.6-28.3)	0.3 (0.1-0.8)	0.4 (-0.4-1.5)
Guyana	14.7 (10.5-18.9)	9.7 (8.5-11.1)	1.9 (1.3-2.4)	18.7 (8.3-30.4)	0.0 (0.0-0.1)	0.2 (-0.3-0.8)
Haiti	13.0 (9.1-17.2)	14.5 (12.6-16.3)	2.9 (2.1-3.8)	18.3 (7.4-31.4)	5.6 (2.7-9.9)	0.5 (-0.2-1.3)
Honduras	11.6 (7.9-15.2)	26.1 (24.2-28.4)	3.2 (2.3-4.2)	15.8 (8.2-25.7)	0.5 (0.2-1.2)	0.4 (-0.2-1.3)
Hungary	13.9 (9.5-18.6)	10.8 (9.3-12.3)	2.1 (1.4-2.8)	16.2 (7.0-27.7)	2.0 (0.7-4.6)	0.4 (-0.8-2.3)
Iceland	12.4 (8.5-16.9)	17.5 (15.5-19.7)	2.7 (1.9-3.5)	16.1 (7.6-27.1)	3.9 (1.7-7.3)	1.2 (0.0-2.7)
India	16.2 (11.8-20.6)	12.4 (10.7-14.2)	2.4 (1.6-3.2)	19.5 (7.7-32.9)	0.9 (0.3-1.8)	0.2 (-0.3-0.8)
Indonesia	13.1 (8.8-17.4)	13.4 (11.6-15.2)	3.0 (2.1-4.0)	18.4 (7.7-31.6)	2.4 (1.1-4.5)	0.6 (-0.2-1.6)
Iran (Islamic Republic of)	10.3 (7.1-13.8)	6.8 (5.6-8.2)	1.8 (1.3-2.5)	5.7 (2.2-11.2)	37.6 (31.3-42.8)	0.5 (-0.4-1.8)
Iraq	9.0 (5.7-12.4)	15.8 (14.3-17.5)	2.9 (2.1-3.8)	13.9 (9.1-19.7)	2.9 (1.1-6.2)	0.1 (-0.3-1.0)
Ireland	17.3 (12.6-22.1)	14.9 (13.2-16.6)	2.3 (1.6-3.1)	19.2 (8.5-31.9)	1.0 (0.4-2.2)	0.2 (-0.3-0.8)
Israel	15.5 (11.2-20.3)	12.5 (10.4-14.4)	2.5 (1.8-3.3)	19.0 (7.5-32.0)	1.3 (0.5-2.5)	0.2 (-0.3-0.9)
Italy	12.8 (8.7-16.9)	21.8 (19.3-24.4)	4.0 (2.8-5.3)	18.9 (9.1-30.6)	3.3 (1.3-6.5)	0.2 (-0.3-0.9)
Jamaica	13.6 (9.2-17.7)	16.2 (14.3-18.2)	3.2 (2.2-4.2)	19.6 (6.9-34.4)	0.0 (0.0-0.1)	0.1 (-0.2-0.5)
Japan	10.2 (6.6-14.1)	7.2 (6.2-8.3)	1.8 (1.3-2.5)	20.5 (15.6-25.8)	6.3 (3.5-9.9)	6.3 (4.6-8.1)
Jordan	8.6 (4.6-12.9)	7.7 (6.4-9.1)	1.7 (1.1-2.4)	18.3 (11.6-25.2)	10.4 (6.0-15.9)	6.2 (4.5-8.2)
Kazakhstan	8.8 (5.7-12.0)	9.6 (8.2-11.1)	1.8 (1.2-2.5)	18.3 (12.8-23.9)	2.0 (0.8-4.1)	4.6 (3.3-6.0)
Kenya	12.2 (7.3-17.7)	5.4 (4.5-6.5)	2.0 (1.4-2.7)	23.4 (17.6-29.3)	6.2 (3.1-10.5)	7.4 (5.2-9.8)
Kiribati	10.7 (7.5-13.7)	11.4 (10.0-12.8)	2.6 (1.8-3.5)	17.2 (13.7-20.9)	6.3 (4.2-8.9)	3.3 (2.1-4.7)
Kuwait	8.4 (5.6-11.5)	10.9 (9.6-12.4)	2.6 (1.8-3.4)	18.3 (14.0-23.1)	3.4 (1.5-6.5)	3.1 (2.1-4.3)
Kyrgyzstan	10.0 (6.3-14.0)	13.9 (12.5-15.3)	3.0 (2.2-3.9)	15.8 (12.2-19.8)	1.7 (0.7-3.7)	2.2 (0.8-3.8)
Lao People's Democratic Republic	7.1 (4.4-9.8)	8.7 (7.5-10.1)	1.8 (1.3-2.4)	17.5 (11.6-24.3)	5.6 (3.1-9.1)	0.2 (-0.2-0.7)
Latvia	5.2 (3.2-7.3)	8.1 (6.4-9.8)	2.2 (1.5-2.9)	14.7 (8.9-20.6)	20.3 (13.7-27.8)	2.5 (1.4-3.8)
Lebanon	6.3 (4.1-8.5)	11.7 (9.9-13.6)	3.3 (2.4-4.3)	10.9 (6.1-16.6)	23.9 (17.7-29.7)	1.6 (-0.4-4.3)
Lesotho	12.7 (9.0-16.5)	10.8 (9.2-12.6)	2.2 (1.5-3.1)	17.3 (13.6-21.2)	4.7 (2.7-7.5)	5.4 (3.7-7.2)

Liberia	7.5 (5.1-10.3)	10.2 (8.8-11.9)	2.9 (2.1-3.8)	11.7 (6.6-17.9)	19.9 (13.0-26.2)	0.5 (-0.3-1.5)
Libya	13.4 (9.6-17.5)	9.3 (8.1-10.7)	1.9 (1.4-2.5)	11.7 (7.4-15.9)	2.7 (1.0-5.3)	0.8 (0.0-1.7)
Lithuania	12.3 (8.2-16.5)	14.3 (12.3-16.4)	3.3 (2.2-4.4)	20.4 (14.0-27.2)	0.1 (0.1-0.3)	1.2 (0.3-2.5)
Luxembourg	12.4 (8.7-16.2)	19.7 (17.9-21.6)	3.5 (2.5-4.5)	10.6 (7.5-13.9)	3.8 (1.8-6.7)	1.8 (0.5-3.3)
Madagascar	12.3 (8.6-16.0)	19.7 (17.9-21.6)	3.5 (2.5-4.5)	10.6 (7.5-13.9)	3.6 (1.6-6.4)	1.8 (0.5-3.3)
Malawi	16.6 (11.4-21.8)	19.4 (16.7-22.2)	3.7 (2.6-4.8)	10.7 (7.2-15.2)	12.2 (6.3-19.5)	2.2 (-0.2-5.1)
Malaysia	1.4 (0.8-2.1)	16.3 (15.0-17.8)	4.8 (3.6-6.1)	27.9 (24.7-31.2)	5.2 (3.7-7.0)	5.7 (3.3-8.2)
Maldives	1.9 (1.0-3.0)	13.7 (11.6-16.1)	6.2 (4.7-7.8)	28.0 (20.7-34.6)	0.1 (0.0-0.2)	4.3 (0.8-7.7)
Mali	3.5 (1.9-5.3)	16.9 (14.5-20.0)	4.4 (3.3-5.7)	42.3 (36.8-47.2)	0.1 (0.0-0.3)	2.5 (-1.7-6.2)
Malta	0.6 (0.1-1.3)	12.7 (9.8-16.0)	4.2 (3.0-5.6)	32.1 (26.5-38.2)	0.0 (0.0-0.1)	3.9 (-0.1-8.4)
Marshall Islands	1.8 (1.2-2.6)	15.2 (13.9-16.6)	4.4 (3.3-5.5)	29.8 (26.2-33.3)	0.1 (0.0-0.2)	8.1 (6.2-10.1)
Mauritania	1.6 (0.5-2.8)	20.6 (18.5-22.8)	5.4 (4.0-6.9)	34.7 (29.4-40.0)	0.1 (0.0-0.2)	4.8 (1.3-8.4)
Mauritius	1.5 (0.5-2.6)	23.9 (21.1-26.5)	5.3 (3.9-6.8)	26.9 (22.8-30.8)	0.0 (0.0-0.0)	5.5 (1.4-10.3)
Mexico	0.2 (0.0-0.7)	24.0 (21.4-26.5)	5.2 (3.9-6.6)	42.5 (38.3-46.5)	0.0 (0.0-0.0)	3.8 (-3.1-9.8)
Micronesia (Federated States of)	2.6 (1.4-4.0)	28.5 (25.7-31.8)	5.4 (4.1-6.8)	25.0 (20.1-30.1)	0.1 (0.0-0.2)	7.5 (5.2-9.9)
Monaco	0.9 (0.2-1.5)	13.8 (10.5-17.4)	6.5 (4.9-8.4)	30.9 (23.5-37.9)	0.1 (0.0-0.2)	3.8 (-0.7-8.7)
Mongolia	1.1 (0.4-2.0)	11.6 (9.8-13.7)	4.3 (3.1-5.6)	28.3 (23.4-33.0)	1.8 (0.8-3.5)	6.8 (4.5-9.5)
Montenegro	3.4 (2.3-4.7)	18.6 (16.5-21.3)	5.5 (4.0-7.1)	26.1 (20.4-31.5)	0.4 (0.2-0.8)	5.0 (0.4-10.1)
Morocco	0.8 (0.1-1.6)	8.0 (6.6-9.7)	3.5 (2.6-4.5)	32.5 (26.1-38.8)	0.1 (0.0-0.3)	2.9 (-4.2-9.1)
Mozambique	2.5 (1.0-4.2)	17.2 (14.9-19.7)	4.9 (3.6-6.2)	47.4 (42.6-51.5)	0.0 (0.0-0.0)	3.0 (-4.0-9.3)
Myanmar	0.8 (0.0-1.9)	17.1 (14.6-19.9)	5.8 (4.2-7.6)	44.0 (39.0-48.4)	0.1 (0.0-0.3)	3.2 (-0.2-6.7)
Namibia	1.6 (0.8-2.4)	20.3 (18.3-22.4)	4.7 (3.4-6.0)	26.0 (21.5-30.5)	0.0 (0.0-0.1)	6.5 (2.9-10.6)
Nauru	3.9 (2.5-5.6)	18.8 (16.5-21.2)	5.3 (4.0-6.8)	26.3 (20.1-32.5)	0.1 (0.0-0.2)	5.6 (1.4-10.1)
Nepal	3.6 (2.1-5.2)	23.8 (21.7-25.9)	4.1 (3.0-5.2)	22.7 (19.2-26.3)	0.1 (0.0-0.3)	10.0 (7.2-12.7)
Netherlands	4.3 (1.7-7.2)	22.8 (19.7-26.2)	6.1 (4.3-8.2)	41.1 (34.1-48.3)	0.0 (0.0-0.0)	3.7 (-5.9-12.0)
New Zealand	1.3 (0.7-2.1)	20.6 (18.3-23.2)	5.3 (4.0-6.8)	19.9 (10.3-29.9)	16.7 (10.3-24.1)	2.3 (-0.2-4.7)
Nicaragua	5.4 (3.3-7.7)	13.9 (12.3-15.5)	4.0 (2.8-5.1)	24.5 (18.7-29.8)	19.2 (13.6-25.6)	2.5 (-0.3-4.9)

Niger	0.2 (0.0-0.4)	9.5 (7.8-11.5)	5.4 (3.8-7.1)	10.9 (4.8-19.4)	34.9 (26.4-42.1)	9.1 (6.4-12.5)
Nigeria	0.9 (0.1-1.7)	15.5 (13.3-17.5)	4.0 (2.8-5.2)	19.3 (12.8-25.7)	24.4 (17.6-31.8)	1.6 (-0.4-3.7)
Niue	2.6 (0.7-4.7)	8.9 (7.2-11.0)	2.4 (1.7-3.1)	16.3 (9.9-22.5)	20.6 (14.2-27.8)	11.8 (8.3-16.4)
North Macedonia	6.9 (4.2-10.0)	13.5 (11.7-15.2)	3.9 (2.8-5.1)	26.2 (20.6-31.2)	17.7 (12.5-23.8)	2.1 (-0.5-4.4)
Northern Ireland	6.3 (1.8-11.2)	19.4 (17.2-21.6)	3.2 (2.2-4.2)	20.6 (12.8-28.2)	24.0 (16.3-32.4)	9.3 (6.3-12.7)
Northern Mariana Islands	1.7 (0.9-2.6)	14.2 (11.8-16.5)	4.2 (3.0-5.5)	21.4 (14.9-27.8)	21.6 (14.8-28.5)	4.8 (0.0-9.2)
Norway	8.8 (6.1-11.6)	8.4 (7.4-9.4)	2.2 (1.6-3.0)	10.9 (7.2-15.3)	31.4 (25.8-37.1)	2.1 (0.9-3.4)
Oman	7.9 (4.3-11.5)	8.4 (6.9-9.8)	1.8 (1.2-2.4)	10.0 (4.9-16.4)	32.9 (26.2-38.9)	1.7 (0.2-3.5)
Pakistan	16.3 (11.7-21.1)	12.1 (10.6-13.7)	2.7 (1.9-3.6)	14.1 (7.1-22.2)	20.3 (14.4-26.6)	2.2 (0.2-4.8)
Palau	6.4 (2.0-11.4)	8.9 (7.0-11.0)	2.1 (1.5-3.0)	6.1 (1.9-13.6)	41.9 (33.4-49.1)	0.5 (-0.2-1.4)
Palestine	11.4 (5.2-17.6)	8.7 (7.2-10.3)	2.5 (1.7-3.3)	21.8 (12.4-32.6)	19.2 (11.6-27.7)	0.6 (-0.6-2.4)
Panama	4.9 (1.1-9.5)	7.1 (5.6-8.7)	1.4 (0.9-2.0)	7.7 (3.2-14.2)	37.7 (30.2-43.7)	1.8 (0.2-3.8)
Papua New Guinea	14.6 (9.2-20.3)	8.5 (6.3-10.9)	2.4 (1.7-3.2)	29.1 (18.8-38.9)	5.9 (2.4-11.3)	0.9 (-1.3-4.1)
Paraguay	18.4 (12.3-25.1)	10.7 (8.6-12.8)	2.5 (1.6-3.4)	28.8 (18.5-39.1)	2.3 (0.9-4.5)	0.5 (-0.5-2.1)
Peru	8.8 (5.8-11.9)	8.9 (7.7-10.3)	2.1 (1.5-2.9)	6.5 (3.5-10.6)	38.3 (32.6-43.6)	2.8 (1.2-4.8)
Philippines	14.0 (9.2-19.3)	8.2 (6.5-10.0)	1.7 (1.2-2.4)	4.5 (1.5-10.0)	42.6 (35.1-49.0)	4.0 (0.1-9.0)
Poland	1.3 (0.1-3.0)	8.4 (6.7-10.2)	3.5 (2.5-4.6)	6.1 (3.0-10.4)	34.6 (29.2-39.7)	0.4 (-0.8-2.2)
Portugal	1.0 (-0.4-2.7)	16.5 (13.2-19.7)	4.0 (2.8-5.4)	25.8 (13.6-37.6)	10.6 (5.4-17.9)	1.5 (-1.5-4.2)
Puerto Rico	3.7 (0.8-7.1)	7.9 (6.0-10.1)	2.3 (1.6-3.1)	12.1 (5.5-21.6)	32.2 (23.9-39.6)	1.4 (0.1-2.8)
Qatar	6.9 (3.0-11.4)	3.7 (3.0-4.5)	1.2 (0.8-1.6)	6.2 (3.2-10.7)	38.1 (31.8-44.1)	3.9 (2.5-5.4)
Republic of Korea	10.5 (6.4-15.2)	9.5 (7.9-11.1)	2.0 (1.4-2.8)	9.4 (5.6-14.1)	30.7 (24.6-36.4)	3.8 (2.1-5.8)
Republic of Moldova	4.3 (1.4-8.0)	7.8 (6.2-9.2)	2.7 (1.8-3.8)	4.7 (2.1-8.6)	42.2 (37.0-46.9)	2.8 (1.1-4.9)
Romania	7.5 (3.9-11.4)	10.8 (8.7-13.1)	2.1 (1.5-2.9)	4.7 (2.0-9.1)	39.9 (34.2-45.1)	3.0 (0.4-6.3)
Russian Federation	11.0 (5.8-16.5)	18.5 (16.6-20.4)	4.3 (3.0-5.6)	14.7 (7.8-22.0)	0.3 (0.1-0.8)	1.3 (-2.3-6.9)
Rwanda	4.8 (0.9-9.2)	11.4 (9.3-13.6)	2.1 (1.4-3.0)	3.6 (1.5-7.6)	43.5 (37.9-49.1)	1.8 (-0.2-4.3)
Saint Kitts and Nevis	16.0 (10.1-22.3)	14.1 (11.5-16.8)	2.3 (1.6-3.0)	9.1 (3.8-16.5)	36.3 (28.1-42.8)	5.2 (1.8-9.5)
Saint Lucia	16.9 (10.8-23.4)	23.1 (20.3-25.9)	3.8 (2.7-5.0)	15.9 (8.8-23.9)	0.2 (0.1-0.4)	0.1 (-0.2-0.5)

Saint Vincent and the Grenadines	0.0 (0.0-0.0)	9.1 (6.9-11.5)	2.5 (1.7-3.4)	1.5 (0.3-4.7)	50.0 (40.9-61.5)	0.8 (0.0-1.6)
Samoa	13.9 (9.6-18.5)	13.4 (11.2-15.8)	2.8 (2.0-3.7)	6.3 (3.2-10.8)	37.3 (31.5-42.5)	2.6 (0.4-5.4)
San Marino	18.4 (13.1-23.8)	7.7 (6.1-9.6)	1.9 (1.3-2.6)	8.0 (3.9-13.9)	36.1 (29.5-42.1)	1.8 (-0.1-4.4)
Sao Tome and Principe	11.6 (6.7-16.7)	10.9 (9.1-12.8)	2.6 (1.8-3.6)	11.3 (6.0-18.2)	30.8 (23.4-37.3)	2.7 (-0.8-7.2)
Saudi Arabia	13.7 (9.9-17.5)	15.4 (13.8-17.0)	3.5 (2.4-4.6)	20.8 (16.6-25.2)	10.1 (6.8-13.8)	6.3 (4.7-8.2)
Scotland	12.1 (8.1-16.9)	17.4 (15.2-19.9)	4.5 (3.1-6.0)	19.3 (13.5-25.8)	12.6 (6.6-20.1)	2.8 (-1.1-7.5)
Senegal	10.8 (5.7-16.0)	16.7 (13.9-19.3)	4.1 (2.9-5.3)	13.6 (7.6-20.4)	24.8 (17.8-31.6)	11.8 (7.5-16.4)
Serbia	14.8 (8.7-21.2)	11.7 (10.2-13.4)	2.7 (1.8-3.7)	16.8 (10.5-23.5)	15.8 (9.0-24.2)	3.4 (0.3-6.9)
Seychelles	15.0 (11.0-19.1)	15.6 (13.8-17.5)	3.5 (2.4-4.6)	24.2 (19.9-28.7)	4.1 (2.0-6.7)	6.8 (5.2-8.7)
Sierra Leone	13.3 (9.3-17.7)	7.2 (5.8-8.8)	2.0 (1.3-2.7)	15.2 (9.5-21.4)	20.3 (12.8-28.4)	5.3 (2.1-9.1)
Singapore	8.3 (4.3-12.6)	15.2 (12.6-18.2)	3.2 (2.3-4.3)	8.6 (4.5-14.2)	30.7 (24.4-36.5)	3.3 (0.6-6.6)
Slovakia	8.5 (5.8-11.1)	6.8 (5.9-7.7)	2.3 (1.6-3.0)	13.9 (9.1-19.5)	27.8 (21.4-34.3)	0.8 (-0.5-2.2)
Slovenia	6.4 (3.3-9.8)	7.2 (5.9-8.4)	2.1 (1.5-2.9)	8.6 (3.9-15.1)	33.4 (26.2-39.5)	0.3 (-0.2-1.0)
Solomon Islands	14.2 (8.3-20.4)	6.9 (5.5-8.5)	2.4 (1.6-3.3)	5.7 (1.9-12.0)	37.4 (29.1-45.1)	0.9 (-1.0-2.6)
Somalia	13.4 (8.9-18.1)	9.1 (7.7-10.7)	2.1 (1.5-2.8)	20.3 (12.8-27.7)	22.1 (14.7-29.7)	1.1 (-0.3-3.0)
South Africa	12.9 (8.5-17.8)	8.5 (7.3-9.8)	2.3 (1.6-3.1)	28.8 (20.2-36.2)	8.3 (4.6-13.6)	2.3 (-2.3-8.1)
South Sudan	6.7 (1.9-11.2)	7.6 (5.9-9.4)	2.3 (1.6-3.0)	5.5 (2.0-11.7)	36.4 (27.9-44.1)	2.5 (-1.5-6.2)
Spain	12.3 (7.1-18.1)	12.4 (10.6-14.4)	3.6 (2.6-4.7)	13.6 (6.6-22.0)	29.4 (21.2-36.8)	0.2 (-0.1-0.7)
Sri Lanka	7.4 (4.1-11.2)	10.8 (9.1-12.5)	3.8 (2.8-4.8)	11.4 (5.6-19.1)	33.0 (25.0-40.2)	0.5 (-0.3-1.3)
Suriname	10.3 (6.0-14.7)	5.4 (4.5-6.4)	1.8 (1.3-2.5)	23.3 (15.1-31.4)	20.4 (13.2-28.1)	0.2 (-0.1-0.6)
Sweden	2.9 (1.2-5.1)	11.2 (9.3-13.2)	2.6 (1.9-3.5)	6.8 (2.8-13.0)	35.6 (28.0-42.4)	0.4 (-0.1-1.2)
Switzerland	8.6 (5.0-12.7)	5.4 (4.4-6.3)	3.0 (2.1-4.0)	8.7 (3.7-16.1)	37.7 (29.3-44.7)	0.4 (-0.3-1.2)
Syrian Arab Republic	10.1 (6.0-14.4)	7.3 (6.0-8.7)	2.1 (1.5-2.9)	9.2 (4.3-16.3)	35.7 (28.2-42.3)	0.3 (-0.5-1.7)
Taiwan (Province of China)	2.7 (1.4-4.2)	6.0 (4.6-7.4)	2.3 (1.6-3.0)	5.4 (1.9-11.6)	35.2 (26.8-43.4)	3.0 (-4.5-9.6)
Tajikistan	0.0 (0.0-0.0)	9.9 (8.2-11.9)	2.6 (1.8-3.5)	22.4 (13.7-31.1)	18.5 (11.3-26.1)	3.3 (-3.1-9.1)
Thailand	0.8 (-0.1-1.9)	4.5 (3.6-5.5)	2.2 (1.5-3.0)	4.6 (1.2-11.9)	38.4 (28.0-48.2)	2.9 (-3.0-8.2)
Timor-Leste	9.2 (5.9-13.1)	4.8 (3.8-5.9)	1.9 (1.4-2.6)	16.9 (11.1-23.6)	23.0 (16.3-29.9)	0.4 (-0.4-1.4)

Togo	12.9 (8.7-18.2)	5.6 (4.6-6.8)	1.5 (1.0-2.0)	14.4 (7.7-22.7)	24.0 (17.1-30.6)	0.2 (-0.6-1.6)
Tokelau	1.4 (0.6-2.2)	9.3 (7.8-10.8)	3.9 (2.8-5.0)	12.2 (6.0-20.4)	30.7 (22.4-38.0)	0.7 (-0.6-1.9)
Tonga	8.8 (5.3-12.7)	10.6 (8.5-12.8)	3.1 (2.1-4.1)	7.0 (3.0-13.2)	33.6 (26.5-40.3)	0.2 (-0.3-1.0)
Trinidad and Tobago	7.3 (4.1-11.1)	11.4 (9.6-13.1)	2.8 (2.0-3.7)	11.5 (5.7-18.4)	33.4 (25.9-40.3)	0.2 (-0.1-0.6)
Tunisia	1.7 (-0.2-4.0)	23.9 (21.1-26.9)	5.2 (3.7-6.8)	5.3 (2.1-10.1)	4.1 (1.0-9.9)	0.2 (-0.2-0.6)
Turkey	16.9 (11.3-22.6)	15.2 (13.6-17.1)	2.4 (1.7-3.2)	4.5 (0.8-8.5)	0.4 (0.0-1.2)	2.8 (-4.1-11.7)
Turkmenistan	14.9 (9.5-20.8)	20.3 (17.6-23.2)	5.0 (3.5-6.5)	4.3 (0.8-9.7)	1.0 (0.1-2.9)	0.8 (-1.8-4.1)
Tuvalu	15.4 (8.3-23.4)	34.0 (30.8-37.3)	4.7 (3.2-6.2)	4.0 (0.4-11.2)	0.2 (0.0-0.7)	16.6 (1.9-30.5)
Uganda	5.2 (0.0-12.7)	21.2 (18.2-24.4)	5.3 (3.8-7.0)	7.0 (3.0-11.7)	1.0 (0.2-3.0)	0.1 (-0.2-0.4)
Ukraine	12.1 (0.0-25.6)	20.5 (17.7-23.7)	2.3 (1.7-3.0)	8.8 (5.3-12.5)	0.0 (0.0-0.0)	9.3 (7.3-11.5)
United Arab Emirates	11.2 (6.5-16.3)	27.4 (23.2-31.5)	5.8 (4.1-7.8)	4.7 (1.4-10.1)	1.6 (0.3-3.9)	0.1 (0.0-0.2)
United Kingdom	8.6 (1.6-14.7)	18.0 (15.3-20.8)	5.4 (3.8-7.0)	4.8 (1.1-10.7)	1.0 (0.2-2.6)	0.4 (-0.8-2.0)
United Republic of Tanzania	4.5 (-0.1-11.8)	23.3 (20.2-26.5)	4.7 (3.2-6.4)	7.4 (3.8-11.7)	3.7 (0.9-8.9)	0.2 (-0.1-0.6)
United States of America	7.5 (1.2-14.9)	22.0 (18.7-25.5)	5.7 (4.0-7.5)	4.8 (0.0-11.8)	0.0 (0.0-0.0)	0.0 (-0.1-0.0)
United States Virgin Islands	11.1 (7.8-14.5)	13.7 (11.7-16.0)	2.1 (1.5-2.8)	3.8 (0.9-7.0)	0.0 (0.0-0.0)	0.4 (-0.5-1.9)
Uruguay	7.2 (0.0-18.0)	10.5 (9.0-12.0)	2.8 (1.9-3.8)	7.3 (3.3-11.8)	0.6 (0.1-1.5)	0.3 (-0.3-1.1)
Uzbekistan	17.7 (0.3-27.1)	17.2 (14.5-20.6)	2.2 (1.7-2.8)	6.7 (2.8-11.0)	0.0 (0.0-0.1)	9.4 (7.4-11.6)
Vanuatu	5.5 (2.1-9.2)	19.0 (16.1-22.1)	5.8 (4.1-7.5)	4.2 (0.0-11.4)	0.0 (0.0-0.0)	0.1 (-0.2-0.4)
Venezuela (Bolivarian Republic of)	4.3 (1.2-7.7)	22.6 (19.5-25.9)	5.5 (3.9-7.1)	4.8 (2.1-9.8)	3.9 (1.4-7.8)	0.1 (-0.1-0.3)
Viet Nam	11.7 (0.2-23.7)	12.3 (10.3-14.6)	2.5 (1.8-3.4)	6.9 (3.4-10.6)	0.2 (0.0-0.5)	0.2 (-0.3-0.9)
Yemen	15.7 (6.8-24.7)	19.9 (18.0-22.0)	2.5 (1.8-3.3)	5.1 (2.5-8.0)	0.0 (0.0-0.0)	
Zambia	18.2 (8.4-27.0)	23.0 (21.2-25.1)	2.3 (1.6-3.1)	3.4 (1.1-6.0)	0.0 (0.0-0.0)	13.1 (7.9-19.0)
Zimbabwe	0.8 (-0.3-2.2)	8.9 (7.0-11.1)	2.3 (1.6-3.1)	7.4 (3.2-13.5)	36.9 (29.5-43.1)	0.3 (-0.1-0.9)

Supplement Table 12a. Percent of DALYs (with 95% uncertainty intervals) due to subarachnoid haemorrhage for high systolic blood pressure, high body-mass index, high fasting plasma glucose, high LDL cholesterol, low glomerular filtration rate, diet low in vegetable, diet low in fruits and diet high in sodium by location for both sexes combined in 2019, all ages

Country, region	High systolic blood pressure	High body-mass index	High fasting plasma glucose	Diet low in vegetables	Diet high in red meat	Diet low in fruits	Diet high in sodium
GBD regions							
High-income Asia Pacific	58.0 (48.6-66.5)	28.4 (16.4-40.6)	15.1 (9.7-21.2)	2.4 (0.5-4.5)	10.2 (5.3-14.1)	8.3 (4.1-14.6)	20.4 (9.0-34.5)
Central Asia	57.6 (47.5-66.9)	24.8 (12.6-37.9)	15.3 (9.9-22.0)	0.4 (0.2-0.8)	12.7 (7.2-17.1)	7.6 (3.7-13.5)	24.6 (12.4-39.0)
East Asia	58.0 (47.8-67.2)	25.0 (12.7-38.0)	15.3 (9.9-22.0)	0.3 (0.2-0.5)	12.9 (7.3-17.4)	7.6 (3.6-13.5)	25.1 (12.8-39.4)
Southeast Asia	46.7 (36.1-56.6)	11.0 (2.4-23.9)	13.5 (8.9-19.6)	3.1 (0.2-7.7)	3.6 (0.4-6.1)	9.0 (4.1-16.6)	16.2 (4.2-31.5)
South Asia	52.7 (41.2-62.9)	35.6 (22.0-48.7)	17.7 (11.6-25.5)	1.2 (0.2-4.1)	16.7 (10.4-21.9)	6.1 (2.8-10.6)	7.7 (0.4-20.9)
Southeast Asia, East Asia, and Oceania	59.3 (49.3-67.9)	35.7 (23.8-47.1)	14.6 (9.2-20.7)	6.5 (1.2-12.3)	5.1 (1.4-8.0)	9.8 (5.0-16.7)	12.0 (1.9-25.8)
Oceania	39.3 (29.0-49.9)	22.8 (11.6-35.2)	19.4 (11.2-29.6)	9.5 (2.8-16.0)	4.9 (0.6-7.9)	13.9 (7.4-21.7)	10.8 (1.2-24.4)
Australasia	68.1 (57.5-76.2)	40.2 (27.7-52.1)	12.9 (7.8-18.7)	7.5 (1.4-13.7)	2.4 (0.3-4.2)	10.7 (5.4-18.2)	12.7 (2.1-27.1)
Central Europe, Eastern Europe, and Central Asia	51.1 (39.7-61.3)	35.2 (20.5-49.0)	16.4 (10.2-24.1)	6.2 (0.8-12.2)	6.6 (1.2-10.2)	9.0 (4.2-16.5)	10.6 (1.3-23.8)
Central Europe	67.0 (56.0-75.5)	48.3 (35.2-59.4)	19.2 (12.6-27.0)	6.3 (0.7-12.2)	4.5 (0.6-7.5)	8.5 (3.8-15.6)	11.9 (1.4-25.7)
Eastern Europe	59.1 (47.8-68.8)	36.3 (22.8-49.4)	15.2 (9.6-22.8)	4.9 (0.3-10.6)	2.1 (0.3-3.8)	11.1 (5.4-19.1)	11.4 (1.4-24.9)
Western Europe	54.1 (44.0-64.0)	29.8 (17.2-42.2)	16.0 (9.8-22.9)	5.5 (0.5-11.0)	5.1 (0.7-8.2)	12.4 (6.5-20.0)	10.6 (1.1-23.8)
Central Latin America	45.8 (35.5-55.5)	38.4 (24.1-51.2)	10.8 (6.7-15.8)	6.5 (1.0-12.3)	8.1 (2.5-12.0)	8.2 (4.0-14.4)	10.2 (1.4-23.0)
Tropical Latin America	59.4 (48.6-69.2)	31.2 (18.8-44.0)	29.0 (17.6-41.1)	6.7 (1.2-12.4)	0.9 (0.2-1.6)	11.4 (5.8-18.8)	12.5 (1.6-27.1)
Southern Latin America	50.1 (39.5-60.0)	37.2 (24.8-49.4)	16.7 (10.6-24.5)	7.3 (1.4-13.3)	5.3 (0.9-8.5)	6.0 (2.8-10.7)	12.3 (1.6-26.9)
Andean Latin America	57.9 (47.6-66.7)	15.7 (6.1-27.5)	15.6 (9.2-22.9)	9.0 (2.6-15.3)	4.0 (0.5-6.7)	14.1 (7.5-21.5)	11.1 (1.3-24.8)
Latin America and Caribbean	62.3 (50.8-71.5)	22.5 (11.0-35.5)	14.4 (8.8-21.7)	2.8 (0.2-7.6)	11.2 (4.5-16.0)	10.1 (4.8-18.2)	12.7 (1.5-27.4)
High-income North America	36.8 (27.7-46.3)	33.5 (20.7-46.6)	22.3 (12.2-34.7)	7.1 (1.8-12.6)	5.7 (1.5-9.2)	9.6 (4.9-16.2)	3.8 (0.3-11.7)
North Africa and Middle East	62.1 (51.6-70.4)	58.5 (45.7-67.9)	29.6 (17.9-43.5)	7.5 (1.6-13.5)	10.0 (3.8-14.4)	13.5 (7.3-21.2)	5.7 (0.3-16.3)
Sub-Saharan Africa	43.6 (32.1-54.6)	55.5 (38.5-70.7)	26.1 (14.5-40.9)	9.3 (2.3-15.9)	5.3 (0.7-8.8)	10.8 (5.2-19.0)	3.7 (0.3-11.9)

Central Sub-Saharan Africa	42.3 (31.7-53.8)	50.7 (34.2-66.4)	37.1 (19.1-57.6)	10.0 (2.9-17.0)	7.8 (2.0-12.0)	11.9 (6.0-20.2)	4.5 (0.3-13.8)
Southern Sub-Saharan Africa	42.1 (31.8-52.4)	58.8 (43.3-71.8)	28.4 (16.8-42.3)	9.7 (3.0-16.4)	7.2 (1.6-11.5)	11.4 (5.6-19.1)	5.0 (0.3-14.5)
Eastern Sub-Saharan Africa	31.8 (22.3-42.2)	26.8 (14.2-40.5)	20.8 (10.9-33.9)	6.5 (1.4-11.6)	5.0 (1.0-8.6)	9.0 (4.4-15.5)	3.5 (0.2-11.0)
Western Sub-Saharan Africa	52.1 (41.2-62.5)	59.2 (44.7-70.8)	28.4 (16.7-44.2)	10.4 (3.3-16.9)	8.9 (2.9-13.2)	10.6 (5.2-18.3)	1.7 (0.2-6.8)
Countries in alphabetical order							
Afghanistan	38.4 (27.2-49.6)	45.8 (29.3-61.8)	25.2 (14.4-39.3)	10.9 (3.6-17.6)	3.6 (0.4-6.4)	11.3 (5.6-19.2)	4.7 (0.3-14.6)
Albania	54.9 (46.0-63.5)	53.2 (40.9-63.1)	24.3 (15.6-36.0)	8.4 (2.4-14.2)	6.8 (1.9-10.4)	9.5 (4.7-16.5)	5.5 (0.4-15.2)
Algeria	57.4 (47.7-67.0)	46.8 (32.4-60.5)	21.9 (13.4-32.3)	8.8 (2.4-15.0)	10.4 (4.3-15.1)	9.0 (4.1-16.6)	4.5 (0.3-13.6)
American Samoa	66.9 (57.1-74.7)	52.2 (39.5-63.9)	18.3 (12.3-25.7)	2.1 (0.2-5.0)	12.3 (6.5-16.6)	9.8 (4.9-17.1)	10.4 (2.0-23.1)
Andorra	66.3 (55.6-74.8)	53.5 (40.1-65.6)	20.6 (12.2-30.0)	1.1 (0.3-2.4)	13.9 (8.0-18.5)	10.0 (5.0-17.5)	8.0 (0.8-20.5)
Angola	67.1 (57.1-75.7)	51.9 (39.0-64.1)	25.0 (14.5-37.6)	0.2 (0.2-0.2)	9.9 (3.8-14.2)	5.3 (2.3-9.3)	7.6 (0.5-20.1)
Antigua and Barbuda	62.4 (49.7-73.0)	57.2 (40.8-71.1)	20.3 (11.6-31.9)	0.2 (0.2-0.4)	8.7 (2.4-13.0)	7.5 (3.3-13.6)	7.5 (0.5-20.2)
Argentina	66.2 (56.1-74.8)	41.8 (29.6-54.0)	28.3 (17.4-42.2)	3.0 (0.2-7.5)	6.0 (1.2-9.4)	9.4 (4.4-16.7)	8.0 (0.7-20.5)
Armenia	72.8 (62.0-81.2)	58.2 (43.8-70.4)	23.8 (14.1-35.6)	0.3 (0.2-0.8)	17.5 (11.3-22.8)	9.9 (4.7-17.8)	8.1 (0.6-21.0)
Australia	48.9 (36.3-60.5)	53.1 (38.2-66.6)	9.7 (5.7-14.9)	0.6 (0.2-2.2)	13.5 (6.6-18.6)	12.5 (6.4-21.3)	7.6 (0.6-20.1)
Austria	67.9 (57.0-77.2)	46.4 (32.2-60.3)	7.6 (4.6-11.2)	7.4 (1.3-13.6)	20.3 (13.6-26.0)	16.7 (9.2-24.7)	8.1 (0.6-20.8)
Azerbaijan	61.7 (49.7-71.6)	33.6 (19.3-47.5)	22.8 (12.9-34.9)	0.7 (0.2-2.5)	5.0 (0.6-8.1)	12.2 (6.3-20.5)	7.5 (0.6-20.1)
Bahamas	67.3 (55.2-76.6)	61.3 (47.1-73.4)	14.9 (8.2-23.6)	0.2 (0.2-0.4)	19.3 (12.9-25.0)	8.6 (3.8-16.1)	8.3 (0.6-21.2)
Bahrain	65.4 (54.2-75.2)	56.3 (41.2-68.8)	21.3 (12.2-32.2)	0.2 (0.2-0.2)	13.4 (6.4-18.5)	9.2 (4.1-17.2)	7.9 (0.6-20.4)
Bangladesh	65.0 (55.3-73.3)	50.1 (37.6-61.7)	21.2 (14.0-29.4)	1.2 (0.2-3.0)	13.0 (7.5-17.3)	8.2 (3.9-14.5)	16.5 (4.8-31.0)
Barbados	62.5 (51.5-71.9)	37.6 (24.8-51.8)	12.1 (8.1-17.4)	0.2 (0.2-0.4)	10.8 (5.3-15.0)	3.0 (1.4-4.9)	17.4 (5.6-31.7)
Belarus	65.5 (54.8-74.5)	42.2 (29.4-55.3)	29.3 (17.8-42.6)	0.9 (0.2-3.0)	5.0 (0.8-7.9)	7.0 (3.1-13.0)	17.1 (4.8-32.0)
Belgium	61.1 (50.6-70.4)	48.2 (34.7-60.6)	19.5 (12.1-30.4)	0.4 (0.2-1.0)	14.9 (9.1-19.7)	9.3 (4.4-16.7)	17.3 (5.2-31.8)
Belize	66.6 (56.4-75.3)	49.4 (36.1-61.9)	23.1 (14.5-33.8)	3.4 (0.2-8.1)	9.6 (3.6-13.8)	7.0 (3.1-13.1)	17.1 (4.7-32.0)
Benin	58.5 (47.6-67.9)	50.5 (36.8-62.7)	29.7 (18.5-41.7)	4.4 (0.3-9.4)	12.8 (7.0-17.4)	9.2 (4.2-16.5)	17.0 (5.0-31.8)
Bermuda	58.1 (46.7-68.2)	54.4 (41.9-65.5)	22.3 (14.1-32.6)	1.3 (0.2-4.3)	11.8 (5.6-16.2)	9.4 (4.4-17.0)	20.3 (7.5-35.2)
Bhutan	64.7 (53.6-73.7)	51.9 (38.1-64.3)	28.0 (16.9-39.8)	0.3 (0.2-0.6)	6.8 (1.5-10.3)	6.4 (2.8-11.5)	16.5 (4.5-31.7)
Bolivia (Plurinational State of)	66.9 (56.2-75.8)	51.9 (38.4-63.8)	26.3 (16.4-38.1)	0.2 (0.2-0.4)	13.1 (7.2-17.7)	3.3 (1.6-5.5)	17.0 (4.8-32.0)

Bosnia and Herzegovina	63.9 (53.9-72.2)	56.4 (42.4-68.7)	22.5 (14.8-31.2)	0.8 (0.2-2.4)	16.3 (10.0-21.5)	10.2 (4.9-17.8)	12.4 (2.1-26.7)
Botswana	68.3 (57.9-77.3)	45.2 (34.1-57.3)	14.7 (9.9-21.2)	0.2 (0.2-0.3)	13.9 (8.4-18.5)	7.3 (3.2-13.8)	18.4 (5.7-33.3)
Brazil	69.4 (59.4-77.8)	48.4 (34.2-60.7)	27.4 (17.0-40.9)	2.3 (0.2-6.6)	7.7 (2.2-11.3)	5.3 (2.4-9.1)	17.4 (5.1-32.2)
Brunei Darussalam	66.5 (56.0-75.6)	52.1 (38.2-64.5)	16.3 (10.4-23.4)	2.5 (0.2-7.2)	12.7 (6.6-17.3)	9.9 (4.8-17.5)	17.3 (5.2-32.1)
Bulgaria	67.8 (57.1-76.7)	50.4 (37.8-62.5)	16.9 (10.9-24.7)	3.8 (0.2-8.5)	13.6 (7.7-18.4)	6.3 (2.9-11.4)	17.2 (4.7-31.9)
Burkina Faso	67.9 (58.3-75.8)	52.8 (39.9-64.9)	16.5 (10.8-23.9)	2.7 (0.3-6.5)	11.6 (5.6-16.0)	10.5 (5.3-18.0)	8.3 (0.9-20.6)
Burundi	73.7 (63.0-81.7)	54.2 (40.1-66.6)	11.9 (7.7-17.3)	0.6 (0.2-2.1)	17.4 (11.2-22.8)	9.8 (4.6-18.0)	4.6 (0.2-15.2)
CÃ´te d'Ivoire	66.5 (56.2-75.8)	55.2 (42.9-66.9)	14.0 (9.2-19.6)	2.4 (0.2-6.9)	11.1 (4.7-15.6)	9.7 (4.5-17.4)	2.7 (0.3-10.4)
Cabo Verde	72.4 (62.0-80.4)	55.2 (42.1-66.9)	14.1 (9.3-20.0)	2.3 (0.2-6.8)	11.2 (5.1-15.7)	11.6 (5.9-19.7)	5.7 (0.3-16.9)
Cambodia	73.8 (63.6-81.7)	52.3 (37.8-65.8)	11.4 (7.4-16.4)	2.2 (0.2-6.7)	14.4 (8.0-19.4)	10.1 (4.9-18.1)	5.3 (0.2-17.4)
Cameroon	73.7 (64.0-81.6)	54.6 (40.4-67.4)	13.9 (8.9-19.9)	3.8 (0.2-8.8)	6.5 (1.1-10.1)	10.4 (5.0-18.6)	4.6 (0.2-15.1)
Canada	67.9 (58.2-75.9)	53.0 (39.9-65.2)	18.0 (11.7-26.1)	3.4 (0.3-7.9)	11.9 (5.9-16.3)	10.2 (5.1-17.6)	9.7 (1.2-22.8)
Caribbean	66.1 (56.6-75.1)	51.4 (38.9-63.2)	12.7 (8.4-18.1)	0.8 (0.2-2.8)	9.6 (3.2-13.8)	11.7 (6.0-19.9)	4.4 (0.2-14.5)
Central African Republic	54.2 (44.4-63.1)	19.8 (8.8-31.9)	13.6 (8.8-20.2)	1.0 (0.2-3.0)	7.6 (2.9-11.0)	9.3 (4.8-15.6)	11.0 (1.5-25.0)
Chad	60.6 (48.9-71.3)	35.3 (19.9-50.1)	31.1 (18.4-45.7)	7.2 (1.1-13.8)	7.1 (1.3-10.9)	12.6 (6.5-21.0)	13.3 (2.0-28.1)
Chile	56.1 (46.0-65.1)	17.8 (7.5-29.6)	12.7 (8.2-19.1)	1.0 (0.2-3.0)	6.3 (1.8-9.5)	9.8 (5.0-16.4)	10.2 (1.1-23.9)
China	46.8 (36.6-57.3)	27.2 (13.5-40.8)	17.0 (10.7-25.2)	0.9 (0.2-2.9)	12.8 (7.0-17.6)	7.3 (3.4-13.4)	14.8 (2.8-30.4)
Colombia	44.9 (34.5-55.3)	38.7 (25.7-51.1)	17.6 (11.5-24.8)	1.3 (0.2-4.8)	13.6 (7.3-18.4)	6.2 (2.8-10.9)	12.9 (2.0-28.2)
Comoros	56.2 (46.5-65.7)	50.1 (37.6-61.0)	14.6 (9.1-22.1)	3.4 (0.2-8.2)	18.0 (12.2-23.0)	8.5 (3.9-15.6)	4.0 (0.2-13.7)
Congo	56.5 (46.6-66.5)	50.8 (38.6-61.6)	14.9 (9.1-22.9)	3.5 (0.2-8.4)	18.1 (12.2-23.0)	8.8 (4.1-16.0)	3.7 (0.2-13.0)
Cook Islands	54.9 (44.6-64.7)	47.5 (34.4-59.7)	13.5 (8.4-19.6)	3.1 (0.2-7.7)	17.8 (12.1-22.9)	7.5 (3.5-13.8)	5.0 (0.2-16.4)
Costa Rica	57.9 (48.2-66.5)	39.1 (27.2-51.1)	21.7 (14.1-32.2)	3.2 (0.3-7.2)	13.9 (8.4-18.4)	7.1 (3.4-12.4)	5.2 (0.4-15.9)
Croatia	64.4 (53.9-74.4)	46.7 (32.6-59.6)	16.1 (10.1-24.0)	2.1 (0.2-5.9)	16.2 (10.1-21.2)	6.5 (3.1-11.8)	4.6 (0.2-15.2)
Cuba	62.6 (51.8-72.0)	38.4 (26.1-51.1)	17.8 (11.1-28.1)	2.9 (0.2-7.3)	14.8 (9.1-19.5)	5.4 (2.5-9.3)	6.7 (0.3-19.3)
Cyprus	62.6 (52.7-71.8)	37.4 (25.7-49.5)	17.8 (10.9-27.1)	1.5 (0.2-4.7)	15.1 (9.7-19.9)	7.4 (3.4-13.6)	6.0 (0.3-17.7)
Czechia	61.1 (51.0-70.7)	35.1 (22.9-47.2)	24.4 (15.2-36.4)	4.0 (0.3-8.8)	11.1 (5.1-15.5)	7.0 (3.2-13.0)	5.5 (0.3-16.8)
Democratic People's Republic of Korea	64.3 (54.0-73.6)	38.5 (26.8-50.6)	15.8 (9.8-24.3)	2.4 (0.2-6.5)	14.8 (9.1-19.7)	5.8 (2.7-10.5)	5.4 (0.2-17.1)

Democratic Republic of the Congo	66.1 (55.5-75.0)	41.8 (28.4-54.8)	24.0 (15.2-35.0)	4.2 (0.3-9.1)	13.8 (7.8-18.3)	9.1 (4.2-16.4)	5.8 (0.3-17.5)
Denmark	58.4 (47.9-68.4)	39.0 (27.1-50.1)	10.3 (6.8-14.9)	4.5 (0.3-9.4)	16.4 (10.8-21.4)	9.0 (4.2-16.2)	4.2 (0.2-14.3)
Djibouti	61.9 (51.5-71.5)	41.6 (29.7-53.7)	26.4 (16.1-39.7)	3.7 (0.2-8.5)	14.8 (8.8-19.4)	8.0 (3.6-14.9)	5.9 (0.3-17.4)
Dominica	54.6 (44.3-64.3)	37.6 (24.9-50.2)	17.6 (10.6-27.8)	0.3 (0.2-0.8)	15.1 (9.3-19.7)	3.0 (1.4-4.8)	5.3 (0.3-16.4)
Dominican Republic	55.8 (45.3-65.5)	42.8 (30.5-55.3)	17.2 (10.6-26.9)	4.9 (0.4-10.0)	16.3 (10.9-21.1)	8.4 (3.9-15.2)	5.7 (0.2-17.0)
Ecuador	61.2 (50.6-70.0)	44.5 (31.8-57.5)	17.5 (11.0-26.0)	2.3 (0.2-6.8)	18.2 (12.3-23.3)	8.0 (3.6-14.7)	4.0 (0.2-13.9)
Egypt	60.5 (50.2-69.3)	39.1 (26.7-52.2)	21.0 (13.0-32.7)	0.2 (0.2-0.4)	9.8 (4.2-13.8)	3.4 (1.6-5.6)	5.4 (0.3-16.5)
El Salvador	53.2 (42.5-63.2)	31.5 (20.0-43.6)	23.5 (14.7-36.3)	2.0 (0.2-5.4)	12.7 (7.4-17.0)	4.0 (1.9-6.7)	7.3 (0.6-19.2)
Equatorial Guinea	62.7 (52.5-71.2)	41.0 (28.7-53.3)	24.2 (15.0-35.7)	2.8 (0.2-7.3)	17.0 (11.3-22.1)	8.3 (4.0-14.9)	5.8 (0.3-17.0)
Eritrea	62.9 (52.6-71.7)	37.5 (24.9-50.4)	26.0 (15.8-38.3)	0.7 (0.2-2.2)	12.0 (6.2-16.3)	7.8 (3.7-14.4)	7.8 (0.5-20.2)
Estonia	59.9 (49.3-69.2)	38.6 (25.7-51.7)	15.2 (9.3-23.4)	4.4 (0.3-9.3)	15.3 (9.4-20.0)	7.2 (3.3-13.1)	5.0 (0.2-16.2)
Eswatini	62.8 (52.7-71.6)	34.2 (22.5-45.7)	21.4 (13.4-32.1)	4.9 (0.5-9.9)	13.5 (7.9-17.9)	6.4 (3.1-11.2)	4.7 (0.3-15.2)
Ethiopia	57.6 (46.9-66.8)	33.9 (21.5-46.5)	25.3 (16.1-38.6)	0.8 (0.2-2.6)	16.0 (10.5-20.7)	6.3 (2.9-11.5)	6.1 (0.4-17.2)
Fiji	59.0 (48.3-68.5)	38.8 (27.2-51.3)	23.2 (14.4-36.7)	1.8 (0.2-5.4)	13.7 (7.9-18.2)	4.3 (2.0-7.3)	4.7 (0.4-14.1)
Finland	60.5 (49.7-70.4)	36.3 (24.5-48.5)	19.6 (11.7-30.9)	4.1 (0.4-8.6)	13.1 (7.7-17.3)	7.1 (3.4-12.9)	5.1 (0.3-16.2)
France	48.4 (37.9-58.5)	34.7 (22.9-46.4)	17.1 (11.0-27.5)	3.8 (0.3-8.5)	15.5 (10.0-20.1)	7.3 (3.3-13.7)	5.1 (0.3-16.1)
Gabon	53.8 (45.1-62.0)	44.1 (31.1-56.5)	27.2 (17.6-39.3)	3.9 (0.4-8.3)	10.7 (5.3-14.7)	9.4 (4.8-16.1)	3.5 (0.3-12.6)
Gambia	54.9 (44.8-64.6)	50.1 (35.4-63.4)	17.5 (11.4-24.6)	4.0 (0.3-8.9)	18.2 (12.1-23.3)	6.6 (3.2-11.7)	7.0 (0.4-19.7)
Georgia	53.5 (42.6-63.9)	49.3 (34.6-63.1)	16.2 (10.3-23.1)	4.3 (0.3-9.6)	19.5 (13.0-24.7)	5.7 (2.7-10.0)	6.7 (0.3-19.2)
Germany	58.2 (47.8-68.0)	53.4 (38.6-66.2)	22.1 (13.9-31.7)	2.2 (0.2-6.6)	15.2 (8.8-20.1)	8.8 (4.0-16.1)	7.8 (0.4-21.0)
Ghana	57.0 (46.1-66.4)	46.2 (31.9-59.0)	14.2 (9.2-20.6)	6.7 (1.1-12.6)	17.4 (11.6-22.5)	8.0 (3.7-15.1)	7.5 (0.4-20.4)
Greece	49.4 (40.0-58.5)	52.7 (39.8-63.5)	26.5 (17.9-36.0)	3.4 (0.5-7.3)	14.3 (8.7-19.0)	7.8 (3.7-13.8)	6.2 (0.4-18.1)
Greenland	49.4 (38.5-59.6)	48.4 (35.6-60.3)	16.2 (9.3-25.2)	3.0 (0.2-7.4)	13.4 (7.6-18.0)	7.5 (3.4-13.8)	6.3 (0.4-18.7)
Grenada	49.4 (39.9-58.6)	53.0 (40.3-63.8)	27.4 (18.5-37.0)	3.5 (0.5-7.3)	14.4 (8.8-19.1)	7.8 (3.7-13.8)	6.2 (0.4-18.2)
Guam	54.6 (45.1-63.2)	53.1 (40.0-64.9)	18.9 (11.9-27.0)	6.8 (1.2-12.5)	12.8 (7.4-17.3)	7.0 (3.4-12.3)	6.8 (0.6-18.5)
Guatemala	55.4 (44.8-63.6)	41.2 (28.2-54.3)	20.8 (12.8-30.9)	7.1 (2.0-12.7)	5.3 (1.1-8.6)	7.2 (3.5-12.7)	3.6 (0.2-13.1)
Guinea	57.8 (46.6-67.6)	51.0 (37.0-63.6)	27.1 (16.5-39.3)	7.0 (1.1-13.0)	6.3 (1.1-9.8)	7.1 (3.2-12.9)	4.2 (0.2-14.5)

Guinea-Bissau	59.2 (47.9-70.0)	62.1 (47.4-73.7)	22.1 (13.6-33.1)	4.2 (0.2-9.8)	12.3 (5.2-17.2)	9.2 (4.5-16.3)	4.1 (0.2-14.7)
Guyana	60.6 (49.0-70.1)	52.3 (38.2-64.5)	23.7 (15.4-33.5)	6.1 (0.7-11.7)	7.7 (2.1-11.6)	11.0 (5.6-18.8)	3.3 (0.2-12.9)
Haiti	50.4 (39.3-60.6)	61.3 (47.3-72.0)	17.0 (10.2-25.1)	8.3 (1.8-14.5)	4.7 (0.6-7.8)	2.4 (1.0-4.1)	3.6 (0.2-13.1)
Honduras	50.7 (40.0-60.8)	51.0 (37.3-63.4)	22.2 (14.3-31.7)	2.3 (0.2-6.8)	8.6 (2.6-12.7)	6.3 (2.9-11.1)	4.0 (0.2-14.5)
Hungary	57.3 (46.5-67.5)	52.6 (39.0-64.6)	30.5 (19.3-43.2)	4.8 (0.3-10.0)	7.2 (1.6-11.1)	0.6 (0.3-1.1)	4.4 (0.2-15.2)
Iceland	53.8 (42.0-63.9)	53.9 (39.5-66.4)	12.0 (7.4-18.0)	6.2 (0.6-12.0)	6.8 (1.3-10.6)	3.0 (1.3-5.2)	3.7 (0.2-13.4)
India	56.4 (45.5-66.5)	53.6 (40.2-65.8)	28.5 (17.1-41.6)	9.1 (2.4-15.5)	4.4 (0.5-7.3)	6.8 (3.1-12.3)	4.1 (0.2-14.8)
Indonesia	52.1 (40.8-62.9)	53.5 (38.7-66.5)	27.7 (16.2-41.8)	6.8 (0.9-12.9)	2.3 (0.2-4.1)	11.2 (5.6-19.2)	3.8 (0.2-13.6)
Iran (Islamic Republic of)	57.1 (43.1-67.5)	25.7 (13.5-38.9)	21.2 (11.7-33.5)	9.3 (2.7-15.3)	3.3 (0.3-5.8)	8.8 (4.0-15.8)	3.3 (0.2-12.2)
Iraq	54.6 (43.4-65.0)	56.7 (44.0-67.5)	26.0 (15.9-39.1)	5.4 (0.5-10.8)	4.4 (0.5-7.3)	8.0 (3.6-14.8)	3.8 (0.2-13.8)
Ireland	60.0 (49.2-69.5)	55.3 (41.3-67.3)	29.7 (18.4-43.1)	9.0 (2.2-15.5)	9.4 (2.9-13.8)	8.5 (4.0-15.7)	4.1 (0.2-14.6)
Israel	56.3 (45.5-66.6)	53.8 (39.4-66.2)	28.3 (17.0-40.9)	8.2 (1.8-14.3)	5.7 (0.9-9.1)	6.5 (3.0-11.6)	3.9 (0.2-14.2)
Italy	48.6 (37.6-58.8)	52.9 (38.4-65.4)	27.5 (17.5-39.7)	7.6 (1.5-13.8)	3.5 (0.4-5.9)	8.9 (4.1-16.4)	3.9 (0.2-14.1)
Jamaica	63.6 (53.5-73.0)	57.7 (44.7-69.3)	31.4 (18.9-46.1)	8.2 (2.0-14.2)	5.2 (0.7-8.4)	10.8 (5.4-18.7)	4.6 (0.2-15.8)
Japan	41.0 (32.5-50.1)	55.6 (41.8-67.3)	13.2 (8.5-19.0)	7.3 (1.6-13.2)	8.6 (3.2-12.4)	7.1 (3.4-12.8)	5.8 (0.3-16.7)
Jordan	37.0 (27.2-47.4)	52.6 (38.9-64.5)	14.8 (9.2-21.7)	8.3 (2.0-14.2)	10.9 (4.6-15.5)	8.5 (3.9-15.7)	5.8 (0.3-17.0)
Kazakhstan	40.6 (31.2-50.4)	60.3 (47.3-69.9)	16.1 (10.2-23.6)	7.1 (1.5-12.9)	10.8 (4.7-15.1)	4.4 (2.0-7.4)	5.9 (0.3-16.9)
Kenya	44.2 (35.0-53.8)	53.6 (38.1-67.0)	9.5 (5.8-14.1)	6.8 (1.0-12.7)	4.9 (0.6-8.0)	8.5 (3.9-15.7)	5.7 (0.3-16.4)
Kiribati	53.0 (44.0-61.7)	50.5 (36.8-63.0)	23.6 (15.1-33.2)	6.2 (0.9-11.7)	9.4 (3.9-13.3)	7.4 (3.6-13.1)	8.1 (1.0-20.2)
Kuwait	50.3 (41.0-59.8)	47.8 (34.3-60.7)	22.2 (14.1-32.3)	6.5 (0.9-12.2)	9.4 (3.3-13.6)	6.8 (3.1-12.3)	11.9 (2.5-25.2)
Kyrgyzstan	68.7 (58.5-77.2)	52.9 (38.7-65.9)	23.1 (14.6-33.1)	7.9 (1.7-14.1)	8.3 (2.2-12.4)	6.9 (3.2-12.4)	9.3 (0.7-22.8)
Lao People's Democratic Republic	52.2 (42.1-61.8)	50.2 (35.9-62.1)	21.2 (13.4-30.5)	6.3 (0.9-11.9)	3.5 (0.4-5.9)	9.1 (4.2-16.3)	7.6 (0.5-19.7)
Latvia	49.7 (40.3-58.6)	40.1 (25.9-52.6)	17.4 (10.3-26.5)	6.2 (0.9-11.6)	3.2 (0.3-5.5)	9.0 (4.2-15.8)	6.6 (0.4-17.0)
Lebanon	51.0 (40.4-61.2)	42.7 (28.4-56.8)	23.0 (14.0-34.3)	6.8 (1.2-12.4)	4.2 (0.5-7.1)	8.5 (3.9-15.8)	7.6 (0.5-19.9)
Lesotho	52.6 (43.4-61.9)	54.1 (40.1-65.9)	25.9 (16.6-37.0)	5.5 (0.6-10.8)	11.5 (5.7-15.8)	6.8 (3.3-11.9)	5.6 (0.3-16.8)
Liberia	59.4 (48.8-68.4)	48.1 (34.1-61.3)	23.3 (14.4-34.0)	9.9 (3.2-16.0)	3.0 (0.3-5.2)	13.8 (7.4-21.5)	8.1 (0.5-20.6)
Libya	56.6 (46.4-65.7)	47.0 (33.7-59.3)	23.8 (14.9-34.5)	8.0 (1.9-14.0)	10.1 (4.1-14.4)	8.9 (4.2-16.1)	8.4 (0.6-21.2)

Lithuania	58.2 (46.8-68.4)	52.1 (37.0-65.5)	22.0 (13.4-32.4)	6.8 (1.0-12.7)	9.4 (3.1-13.7)	8.3 (3.8-15.3)	8.8 (0.6-21.9)
Luxembourg	58.8 (48.2-68.2)	57.0 (44.2-68.9)	16.1 (10.2-23.0)	6.9 (1.1-13.0)	18.1 (11.8-23.4)	6.5 (3.0-11.6)	6.8 (0.4-18.8)
Madagascar	58.8 (48.2-68.2)	57.1 (44.3-69.0)	16.1 (10.2-23.0)	6.9 (1.1-12.9)	18.1 (11.7-23.4)	6.5 (3.0-11.6)	6.8 (0.4-18.8)
Malawi	59.7 (48.6-69.4)	54.4 (40.1-66.9)	16.0 (9.6-23.3)	8.2 (1.7-14.5)	17.5 (10.9-22.9)	7.6 (3.4-14.0)	6.7 (0.3-18.8)
Malaysia	50.2 (41.4-58.4)	49.0 (37.5-59.6)	17.3 (11.0-24.9)	2.3 (0.6-4.6)	4.7 (0.8-7.6)	5.7 (3.0-9.4)	1.9 (0.3-7.3)
Maldives	53.3 (42.1-63.5)	55.3 (42.5-66.6)	22.5 (14.2-32.4)	2.8 (0.2-7.4)	5.0 (0.7-8.2)	6.7 (3.0-12.1)	2.2 (0.2-8.5)
Mali	61.1 (48.8-71.2)	67.5 (53.9-77.2)	23.3 (13.2-36.5)	0.8 (0.2-2.9)	8.0 (1.9-12.5)	5.2 (2.3-9.3)	2.7 (0.2-10.3)
Malta	33.9 (23.5-47.3)	40.8 (28.4-53.7)	9.3 (4.9-15.7)	0.1 (0.1-0.1)	3.8 (0.6-6.8)	3.2 (1.4-5.8)	1.4 (0.1-5.4)
Marshall Islands	53.7 (44.8-62.3)	49.3 (37.7-60.1)	21.6 (14.0-30.9)	0.6 (0.2-1.8)	4.7 (0.7-7.5)	3.3 (1.6-5.5)	2.3 (0.3-8.8)
Mauritania	61.5 (51.7-70.1)	57.7 (43.7-68.8)	19.8 (12.1-28.2)	0.9 (0.2-3.3)	2.3 (0.2-4.1)	9.5 (4.4-17.1)	2.2 (0.2-8.4)
Mauritius	56.8 (45.6-66.4)	65.6 (52.7-75.0)	20.2 (12.6-29.3)	2.8 (0.2-7.7)	5.6 (0.8-9.0)	9.3 (4.3-17.1)	2.4 (0.2-9.2)
Mexico	54.5 (43.6-65.3)	69.6 (57.1-78.0)	21.6 (13.3-31.9)	0.4 (0.2-1.5)	12.6 (5.5-17.6)	9.1 (4.1-16.8)	4.8 (0.2-15.3)
Micronesia (Federated States of)	61.0 (50.3-70.3)	56.9 (43.6-67.9)	22.0 (13.8-31.6)	0.2 (0.2-0.3)	8.7 (2.5-13.0)	4.2 (1.9-7.3)	2.2 (0.3-8.4)
Monaco	57.3 (46.4-68.1)	56.2 (43.3-67.7)	20.5 (12.0-32.0)	2.3 (0.2-6.6)	5.1 (0.8-8.5)	7.0 (3.1-13.2)	2.1 (0.2-7.8)
Mongolia	65.0 (54.7-74.5)	49.8 (35.5-62.5)	24.6 (14.9-36.3)	1.2 (0.2-4.1)	5.5 (0.9-8.9)	4.1 (1.8-7.1)	2.2 (0.2-8.9)
Montenegro	47.9 (37.3-57.2)	51.7 (38.7-62.9)	22.4 (13.5-32.5)	2.1 (0.2-6.4)	3.2 (0.3-5.6)	8.5 (4.0-15.2)	2.0 (0.2-7.8)
Morocco	48.5 (39.0-59.3)	54.8 (42.8-66.1)	14.5 (8.3-21.5)	1.9 (0.2-5.7)	9.2 (3.5-13.4)	4.3 (2.0-7.6)	2.1 (0.2-7.6)
Mozambique	56.8 (44.1-69.3)	78.3 (65.6-86.8)	24.2 (12.4-39.6)	0.2 (0.2-0.5)	10.9 (3.3-16.3)	3.5 (1.3-6.4)	3.1 (0.2-11.4)
Myanmar	52.4 (39.5-65.4)	76.8 (63.5-85.6)	18.7 (10.4-29.0)	4.2 (0.2-10.2)	5.9 (0.7-9.7)	10.5 (4.8-19.6)	2.5 (0.2-9.3)
Namibia	31.4 (24.2-39.6)	31.3 (22.2-40.5)	12.4 (7.3-19.0)	2.2 (0.1-5.2)	3.0 (0.4-5.0)	4.0 (1.8-7.7)	1.3 (0.1-5.2)
Nauru	56.9 (46.7-66.4)	52.5 (39.0-64.5)	25.1 (16.0-36.6)	0.6 (0.2-2.0)	4.8 (0.6-7.8)	6.3 (2.8-10.8)	2.2 (0.2-8.7)
Nepal	58.5 (48.4-67.7)	57.0 (42.5-68.5)	16.8 (11.1-24.2)	0.2 (0.2-0.2)	5.4 (0.8-8.6)	2.3 (1.1-3.7)	1.6 (0.3-7.0)
Netherlands	61.7 (46.9-74.8)	81.0 (69.1-88.6)	19.8 (10.3-31.4)	3.2 (0.2-9.2)	10.7 (2.8-16.0)	8.6 (3.7-15.7)	3.1 (0.2-11.6)
New Zealand	46.4 (36.2-56.5)	32.7 (20.3-45.3)	13.7 (7.6-21.6)	7.9 (2.1-13.8)	3.3 (0.3-5.8)	11.3 (5.6-18.2)	1.9 (0.2-7.7)
Nicaragua	56.8 (46.3-65.9)	31.0 (19.2-42.9)	19.2 (11.8-28.0)	7.2 (1.6-13.2)	1.8 (0.4-3.0)	16.2 (9.2-24.2)	7.6 (0.6-20.2)
Niger	49.8 (37.7-61.7)	42.1 (27.3-56.5)	23.7 (13.2-38.1)	10.4 (3.5-17.0)	5.8 (0.9-9.5)	12.2 (6.0-20.2)	2.0 (0.2-7.9)
Nigeria	56.8 (46.4-66.6)	27.6 (15.4-40.2)	13.3 (8.2-19.6)	9.7 (2.6-16.3)	1.4 (0.2-2.5)	15.1 (8.1-23.4)	6.4 (0.3-18.3)

Niue	55.2 (43.9-64.6)	37.7 (23.7-51.2)	16.2 (9.8-23.7)	7.1 (1.3-13.2)	2.6 (0.3-4.6)	11.6 (6.0-19.5)	6.5 (0.3-18.3)
North Macedonia	56.8 (45.8-66.2)	31.7 (19.7-43.4)	21.5 (13.0-31.5)	6.3 (1.0-12.0)	1.1 (0.3-1.8)	17.4 (9.8-25.5)	8.3 (0.8-21.2)
Northern Ireland	50.2 (38.8-60.0)	29.4 (16.8-42.5)	16.4 (10.1-24.3)	6.6 (1.1-12.6)	5.0 (0.7-8.1)	11.1 (5.5-18.6)	6.6 (0.3-18.9)
Northern Mariana Islands	57.7 (47.6-66.5)	32.5 (19.1-46.2)	15.9 (9.6-23.3)	8.6 (2.1-14.6)	5.2 (0.9-8.3)	12.2 (6.3-19.8)	6.2 (0.3-17.6)
Norway	50.4 (38.9-58.8)	31.6 (20.4-43.9)	9.7 (6.0-14.6)	7.7 (2.1-13.3)	3.9 (0.8-6.4)	10.4 (5.5-16.5)	5.3 (0.2-18.4)
Oman	56.4 (45.2-65.0)	28.6 (16.0-41.8)	14.7 (8.7-21.6)	9.7 (3.1-15.9)	3.1 (0.8-5.1)	11.1 (5.8-18.5)	3.3 (0.2-14.3)
Pakistan	58.2 (46.3-67.6)	31.3 (18.0-45.1)	13.4 (7.6-20.6)	6.9 (1.2-12.5)	5.4 (0.8-8.7)	9.3 (4.3-16.5)	4.5 (0.2-18.1)
Palau	56.6 (44.6-66.7)	19.1 (8.3-31.9)	15.4 (8.5-23.8)	10.3 (3.4-16.7)	10.5 (4.2-15.1)	11.3 (5.8-18.6)	4.3 (0.2-17.6)
Palestine	64.2 (52.5-73.1)	50.2 (36.9-62.2)	15.6 (9.2-23.3)	10.6 (3.5-17.3)	5.6 (0.8-9.0)	10.9 (5.4-18.9)	5.0 (0.2-19.8)
Panama	55.2 (43.4-64.3)	26.5 (14.1-40.2)	14.9 (8.9-22.0)	10.5 (3.4-17.0)	1.3 (0.2-2.4)	11.8 (6.0-19.4)	2.6 (0.2-12.1)
Papua New Guinea	60.5 (49.4-69.8)	54.8 (42.8-65.8)	15.1 (9.4-23.1)	8.8 (2.3-14.7)	7.4 (2.1-11.4)	6.6 (3.1-11.8)	4.7 (0.2-18.2)
Paraguay	60.6 (49.3-70.2)	53.7 (39.9-65.8)	19.6 (11.8-29.3)	9.1 (2.4-15.3)	13.2 (6.9-18.1)	4.9 (2.2-8.3)	4.8 (0.2-18.3)
Peru	53.7 (43.9-62.1)	31.2 (18.6-44.4)	9.0 (5.5-13.5)	9.1 (2.5-15.1)	4.0 (0.6-6.4)	11.5 (6.5-17.9)	7.1 (0.3-22.4)
Philippines	53.2 (42.9-62.8)	19.7 (8.5-33.1)	8.3 (4.9-12.6)	9.3 (2.9-15.3)	1.1 (0.2-2.0)	5.8 (2.6-10.1)	6.9 (0.2-22.8)
Poland	59.5 (49.6-68.5)	37.6 (23.7-50.4)	9.0 (4.9-14.7)	10.7 (3.3-17.1)	2.7 (0.3-4.7)	8.3 (3.7-15.7)	8.0 (0.3-25.5)
Portugal	60.1 (48.6-69.5)	35.1 (20.9-49.6)	9.2 (5.3-14.7)	7.8 (1.5-14.1)	7.4 (1.6-11.4)	16.3 (8.8-24.5)	7.1 (0.2-23.6)
Puerto Rico	48.3 (37.0-59.1)	22.6 (11.1-35.9)	8.9 (5.1-14.0)	10.0 (3.0-16.6)	3.7 (0.4-6.5)	12.0 (6.3-19.9)	7.2 (0.2-23.3)
Qatar	43.9 (33.9-53.5)	25.4 (13.5-38.4)	7.9 (4.9-11.9)	9.2 (2.6-15.2)	3.3 (0.4-5.5)	15.7 (8.5-23.2)	6.9 (0.3-21.6)
Republic of Korea	56.5 (46.6-64.8)	43.5 (30.9-55.2)	9.3 (5.7-13.8)	6.8 (1.3-12.7)	5.7 (1.2-8.8)	11.0 (5.8-18.2)	4.9 (0.5-16.2)
Republic of Moldova	55.6 (43.9-65.6)	30.4 (16.6-44.8)	7.2 (4.3-11.1)	10.4 (3.4-17.1)	4.8 (0.6-8.0)	12.3 (6.5-20.2)	6.7 (0.2-22.4)
Romania	61.1 (51.6-70.3)	32.1 (17.7-45.6)	11.7 (6.7-18.7)	9.0 (2.5-15.0)	2.5 (0.3-4.5)	9.7 (4.7-17.0)	6.9 (0.2-22.3)
Russian Federation	57.3 (44.6-69.2)	49.4 (35.7-61.7)	26.7 (16.3-37.9)	5.5 (0.4-11.1)	5.6 (0.8-9.0)	13.9 (7.5-22.3)	12.2 (1.4-26.8)
Rwanda	65.9 (56.1-74.3)	32.0 (18.6-45.5)	11.3 (6.6-17.8)	9.8 (3.0-16.3)	2.5 (0.3-4.4)	14.0 (7.5-21.8)	7.6 (0.2-24.2)
Saint Kitts and Nevis	52.2 (42.0-62.3)	28.0 (14.0-43.2)	10.5 (5.7-17.9)	8.8 (2.3-15.2)	2.8 (0.3-4.9)	0.3 (0.2-0.6)	7.6 (0.2-24.0)
Saint Lucia	61.2 (49.9-71.2)	51.5 (38.0-63.8)	22.6 (14.1-32.8)	8.9 (2.2-15.4)	3.8 (0.5-6.6)	9.7 (4.7-17.1)	7.3 (0.6-18.2)
Saint Vincent and the Grenadines	50.9 (39.4-61.4)	12.7 (3.4-26.3)	8.9 (4.9-14.4)	10.4 (3.3-16.9)	6.7 (1.3-10.5)	14.4 (7.7-22.1)	7.0 (0.2-23.3)
Samoa	61.8 (50.4-70.7)	39.3 (25.9-52.3)	8.2 (4.8-13.0)	8.0 (2.0-13.8)	3.4 (0.3-5.8)	8.5 (4.0-15.7)	10.0 (0.4-27.7)

San Marino	52.9 (41.9-63.5)	32.6 (19.3-46.7)	11.0 (6.6-17.0)	9.1 (2.6-14.9)	4.1 (0.5-7.0)	4.1 (1.8-7.0)	6.6 (0.2-22.1)
Sao Tome and Principe	45.3 (34.6-55.8)	39.7 (25.3-52.9)	10.2 (6.1-15.8)	9.7 (2.9-16.0)	4.4 (0.5-7.2)	16.5 (9.1-24.4)	6.6 (0.2-22.2)
Saudi Arabia	58.5 (49.2-67.3)	47.3 (35.6-57.8)	17.5 (11.1-24.9)	8.3 (2.2-14.3)	8.0 (2.9-11.8)	14.0 (7.5-21.5)	5.3 (0.2-19.7)
Scotland	65.4 (55.4-74.0)	53.3 (39.9-65.0)	17.8 (10.9-26.1)	9.1 (2.4-15.6)	9.2 (2.8-13.6)	15.9 (8.7-24.1)	6.1 (0.2-21.7)
Senegal	55.8 (45.1-65.3)	43.6 (30.8-55.9)	20.0 (11.4-30.1)	9.4 (2.9-15.6)	6.5 (1.3-10.1)	14.9 (8.1-22.4)	5.3 (0.2-20.1)
Serbia	59.3 (49.0-68.0)	43.9 (32.0-54.9)	17.0 (10.5-24.7)	8.4 (2.1-14.4)	8.7 (2.8-12.9)	13.9 (7.4-21.6)	5.4 (0.2-19.4)
Seychelles	66.9 (57.5-75.6)	58.1 (46.4-68.2)	20.5 (13.2-29.2)	7.6 (1.5-13.9)	11.2 (4.9-15.7)	14.1 (7.7-22.2)	4.7 (0.2-18.2)
Sierra Leone	58.5 (49.0-67.1)	53.9 (39.7-66.1)	18.6 (11.7-26.5)	9.5 (3.0-15.6)	8.3 (2.4-12.3)	10.9 (5.5-18.5)	5.4 (0.2-20.4)
Singapore	46.7 (35.9-58.9)	32.6 (20.6-45.0)	13.1 (7.6-19.7)	9.1 (2.8-14.8)	3.8 (0.4-6.6)	13.6 (7.3-20.6)	6.1 (0.2-20.9)
Slovakia	42.8 (26.9-53.2)	29.9 (17.7-41.9)	6.9 (3.7-11.2)	5.2 (1.1-9.6)	3.2 (0.5-5.6)	8.0 (3.6-13.7)	3.9 (0.1-14.9)
Slovenia	41.6 (24.5-53.9)	34.1 (20.7-47.7)	8.6 (4.2-14.3)	4.7 (0.6-9.0)	1.8 (0.2-3.5)	9.1 (4.1-15.4)	4.3 (0.1-15.7)
Solomon Islands	33.1 (16.4-46.6)	22.2 (10.4-36.7)	7.1 (3.1-12.8)	6.6 (1.6-12.0)	4.0 (0.6-7.1)	11.3 (4.8-18.5)	3.3 (0.1-14.0)
Somalia	45.5 (29.8-57.8)	42.7 (28.0-57.4)	7.1 (3.9-11.6)	3.6 (0.2-8.1)	3.7 (0.4-6.7)	6.3 (2.7-12.5)	4.0 (0.1-16.1)
South Africa	64.2 (54.5-72.5)	47.6 (34.5-60.1)	15.3 (9.6-22.2)	2.8 (0.2-7.3)	5.0 (0.7-8.1)	11.2 (5.7-18.8)	5.5 (0.2-20.4)
South Sudan	32.4 (15.8-44.8)	16.5 (6.6-28.4)	6.4 (2.7-10.9)	6.8 (1.6-12.0)	3.7 (0.5-6.8)	10.1 (4.3-16.6)	3.2 (0.1-13.3)
Spain	45.7 (30.9-57.8)	34.7 (21.7-48.6)	7.5 (3.9-12.0)	6.3 (1.5-11.2)	4.2 (0.5-7.1)	7.2 (3.1-13.4)	4.4 (0.1-16.4)
Sri Lanka	50.6 (38.6-60.4)	37.7 (25.7-50.1)	8.1 (4.7-13.1)	8.1 (2.2-13.6)	2.1 (0.2-3.8)	14.4 (7.9-21.5)	4.2 (0.1-16.4)
Suriname	51.2 (39.2-62.0)	48.5 (35.9-61.1)	10.5 (6.1-16.1)	7.1 (1.4-13.0)	3.0 (0.3-5.4)	4.9 (2.3-8.7)	7.2 (0.2-23.6)
Sweden	37.8 (20.8-51.1)	22.8 (11.4-35.6)	6.8 (3.2-11.2)	5.8 (1.2-10.2)	2.2 (0.2-4.3)	5.7 (2.1-11.2)	3.5 (0.1-13.7)
Switzerland	46.5 (33.2-57.6)	26.9 (14.2-41.1)	8.1 (4.5-13.0)	8.7 (2.4-14.4)	4.9 (0.7-8.2)	9.6 (4.5-16.4)	4.1 (0.1-16.1)
Syrian Arab Republic	52.0 (38.8-62.4)	46.8 (34.2-58.6)	10.3 (6.0-16.2)	8.7 (2.3-14.7)	2.1 (0.2-3.8)	10.7 (5.2-17.5)	4.4 (0.1-17.1)
Taiwan (Province of China)	28.7 (14.3-40.8)	21.3 (10.1-34.8)	6.1 (2.6-10.7)	5.8 (1.3-10.5)	5.4 (1.5-9.3)	7.7 (3.0-13.7)	3.0 (0.1-12.8)
Tajikistan	52.2 (41.2-61.5)	47.9 (35.9-58.8)	7.0 (4.2-10.7)	8.8 (2.3-14.9)	8.0 (2.4-12.0)	14.7 (8.0-22.1)	4.8 (0.2-18.2)
Thailand	32.6 (13.0-47.1)	16.4 (5.7-29.1)	4.7 (1.6-9.1)	3.5 (0.3-7.3)	4.7 (1.0-8.5)	8.7 (3.1-15.4)	3.0 (0.1-12.8)
Timor-Leste	46.7 (27.4-58.9)	29.1 (16.4-42.0)	6.1 (3.0-10.0)	4.1 (0.4-8.4)	2.6 (0.3-4.9)	7.6 (3.0-13.9)	3.4 (0.1-14.1)
Togo	61.4 (50.5-70.8)	49.8 (36.6-62.9)	10.4 (6.3-15.9)	8.9 (2.5-15.1)	1.4 (0.2-2.6)	5.6 (2.4-10.1)	4.9 (0.2-18.8)
Tokelau	49.2 (37.2-58.9)	34.5 (22.5-47.1)	11.8 (7.0-18.5)	6.2 (1.1-11.1)	3.7 (0.4-6.5)	11.7 (5.7-18.5)	4.4 (0.1-17.1)
Tonga	42.7 (24.2-56.3)	21.0 (9.6-34.3)	4.3 (2.0-7.4)	5.8 (1.3-10.6)	1.3 (0.1-2.7)	8.7 (3.6-14.9)	3.5 (0.1-13.8)

Trinidad and Tobago	47.3 (33.0-58.3)	33.5 (19.9-47.0)	5.5 (3.1-8.9)	8.8 (2.5-14.6)	2.1 (0.2-4.0)	14.4 (7.3-22.0)	4.3 (0.1-16.8)
Tunisia	60.0 (49.7-68.6)	66.2 (54.9-74.7)	33.6 (19.5-50.9)	8.3 (2.0-14.4)	9.0 (3.0-13.4)	9.0 (4.2-16.0)	2.7 (0.2-10.8)
Turkey	56.3 (45.6-65.7)	52.7 (38.9-65.0)	19.0 (12.0-28.2)	3.4 (0.2-8.2)	13.7 (7.6-18.4)	9.1 (4.5-15.9)	4.7 (0.2-15.8)
Turkmenistan	57.8 (47.9-67.7)	60.3 (47.9-71.0)	30.2 (19.5-43.3)	7.5 (1.5-13.6)	9.8 (3.8-14.1)	7.7 (3.6-14.1)	6.6 (0.4-18.0)
Tuvalu	51.7 (40.0-62.7)	55.1 (39.6-68.6)	16.4 (9.7-25.1)	1.8 (0.2-5.8)	16.9 (10.5-22.2)	7.5 (3.5-13.6)	6.6 (0.3-19.2)
Uganda	57.2 (46.7-66.2)	58.0 (43.7-68.8)	19.6 (12.4-28.3)	6.5 (1.0-12.3)	11.0 (4.8-15.5)	6.8 (3.2-12.3)	5.6 (0.3-15.8)
Ukraine	62.5 (51.2-72.2)	43.3 (29.9-55.8)	16.9 (10.7-26.0)	0.3 (0.2-0.8)	16.7 (10.9-21.5)	2.6 (1.2-4.5)	5.1 (0.2-16.6)
United Arab Emirates	51.8 (40.9-62.4)	56.0 (41.8-68.0)	19.9 (10.3-32.4)	8.2 (2.1-14.1)	8.1 (2.5-12.6)	9.3 (4.4-16.2)	3.2 (0.2-10.6)
United Kingdom	58.8 (49.8-67.7)	54.4 (41.8-65.7)	32.3 (20.0-47.7)	8.1 (2.0-14.1)	8.0 (2.4-12.1)	8.7 (4.2-15.8)	6.3 (0.4-17.0)
United Republic of Tanzania	61.2 (49.8-70.8)	63.4 (48.8-74.3)	28.8 (17.3-43.5)	7.7 (1.6-13.8)	10.1 (3.8-14.8)	7.9 (3.8-14.4)	6.8 (0.4-18.6)
United States of America	58.9 (46.9-69.0)	65.0 (51.2-76.0)	32.0 (18.4-48.8)	9.0 (2.2-15.8)	9.6 (3.1-14.6)	9.7 (4.6-17.3)	5.5 (0.4-16.2)
United States Virgin Islands	58.2 (47.2-67.8)	54.1 (41.6-64.8)	28.6 (19.1-39.4)	8.4 (2.2-14.3)	6.4 (1.5-9.9)	6.1 (2.8-11.1)	4.2 (0.2-14.7)
Uruguay	63.9 (53.0-73.3)	55.3 (39.8-68.7)	27.8 (16.7-40.3)	7.1 (1.2-13.2)	7.0 (1.5-11.0)	13.0 (6.8-20.9)	4.5 (0.2-15.5)
Uzbekistan	62.7 (51.7-71.9)	44.2 (31.0-57.5)	19.1 (11.9-29.5)	2.4 (0.2-6.6)	15.1 (9.1-19.9)	6.5 (3.0-12.1)	5.5 (0.2-17.0)
Vanuatu	53.2 (41.5-63.6)	54.8 (41.1-66.7)	28.1 (17.6-41.5)	9.0 (2.4-15.1)	7.9 (2.1-12.2)	10.0 (4.8-17.5)	5.8 (0.4-16.2)
Venezuela (Bolivarian Republic of)	51.6 (41.3-61.5)	52.8 (36.8-66.4)	28.3 (16.8-42.1)	9.6 (3.0-16.1)	7.3 (1.8-11.5)	11.3 (5.8-18.8)	5.3 (0.3-15.4)
Viet Nam	61.1 (50.0-71.3)	57.0 (43.1-68.5)	29.8 (18.6-43.1)	5.2 (0.5-10.6)	6.9 (1.7-10.8)	4.4 (2.1-7.9)	4.5 (0.2-15.7)
Yemen	60.9 (51.2-70.2)	46.7 (33.0-59.3)	19.4 (12.2-29.9)	3.2 (0.3-7.7)	11.5 (6.0-15.9)	9.8 (5.0-16.7)	3.3 (0.2-12.0)
Zambia	56.1 (45.7-66.1)	47.7 (33.9-60.4)	26.3 (16.3-38.2)	4.0 (0.4-8.6)	10.5 (5.2-14.7)	9.9 (5.2-16.7)	2.2 (0.2-8.9)
Zimbabwe	54.8 (42.7-65.6)	38.3 (24.3-51.4)	8.8 (5.1-13.7)	8.4 (2.1-14.5)	4.3 (0.6-7.6)	8.3 (3.8-15.5)	7.2 (0.2-23.2)

Supplement Table 12b. Percent of DALYs (with 95% uncertainty intervals) due to subarachnoid haemorrhage for diet low in whole grains, alcohol use, low physical activity, smoking, second-hand smoking, ambient particulate matter$_{2.5}$ pollution, household air pollution from solid fuels and low ambient temperature by location for both sexes combined in 2019, all ages

Country, region	Smoking	Second-hand smoking	Ambient particulate matter$_{2.5}$ pollution	Household air pollution from solid fuels	Low ambient temperature
GBD regions					
High-income Asia Pacific	21.8 (19.2-24.5)	4.8 (3.5-6.1)	25.4 (21.0-28.7)	10.4 (6.3-15.9)	6.2 (4.6-8.2)
Central Asia	22.9 (19.5-25.8)	4.9 (3.7-6.2)	29.7 (25.0-33.3)	8.4 (4.7-13.5)	8.9 (6.8-11.8)
East Asia	23.1 (19.4-26.1)	4.9 (3.7-6.3)	30.1 (25.4-33.6)	8.0 (4.2-13.1)	8.9 (6.8-11.8)
Southeast Asia	19.9 (15.6-24.3)	5.3 (3.8-7.0)	18.0 (11.2-25.3)	25.9 (18.1-33.0)	10.8 (7.0-16.1)
South Asia	19.7 (17.4-22.1)	4.7 (3.5-6.1)	23.0 (20.1-26.1)	1.0 (0.2-2.7)	3.9 (2.1-6.2)
Southeast Asia, East Asia, and Oceania	19.4 (16.5-23.2)	4.6 (3.4-6.0)	17.1 (13.3-20.8)	14.0 (8.9-20.4)	0.9 (0.2-1.9)
Oceania	17.9 (14.8-22.7)	5.2 (3.8-6.8)	8.4 (4.1-13.8)	33.5 (27.1-38.9)	0.1 (0.0-0.3)
Australasia	20.6 (15.2-26.1)	5.2 (3.5-7.2)	16.9 (12.8-21.2)	12.3 (6.4-19.7)	0.7 (0.0-1.7)
Central Europe, Eastern Europe, and Central Asia	17.6 (13.6-23.2)	5.0 (3.5-6.9)	8.1 (4.0-13.0)	36.3 (29.6-42.0)	1.7 (-0.2-4.1)
Central Europe	16.1 (13.7-18.9)	5.0 (3.6-6.6)	17.0 (12.4-21.3)	0.2 (0.1-0.5)	0.4 (-0.2-1.2)
Eastern Europe	21.5 (18.8-25.7)	5.0 (3.7-6.4)	10.6 (7.1-14.6)	4.9 (2.1-9.2)	0.0 (0.0-0.0)
Western Europe	14.1 (11.3-18.1)	4.0 (2.8-5.4)	13.6 (8.4-18.9)	27.5 (20.5-34.1)	2.7 (1.2-4.3)
Central Latin America	19.7 (16.9-23.0)	5.0 (3.7-6.6)	14.9 (11.2-18.5)	17.7 (11.6-24.3)	0.2 (0.0-0.5)
Tropical Latin America	10.0 (8.0-13.9)	3.1 (2.2-4.2)	15.4 (10.6-20.7)	14.3 (7.0-24.0)	0.4 (-0.1-1.0)
Southern Latin America	18.6 (16.3-21.0)	3.4 (2.5-4.3)	23.6 (19.5-27.4)	5.4 (2.2-10.6)	0.5 (-0.1-1.3)
Andean Latin America	16.2 (11.3-22.7)	4.1 (2.8-5.5)	6.7 (3.0-12.3)	32.1 (24.9-38.2)	0.7 (-0.4-2.1)
Latin America and Caribbean	23.2 (17.2-28.6)	4.7 (3.3-6.4)	17.0 (12.1-21.5)	14.9 (8.1-23.0)	1.6 (0.0-3.8)
High-income North America	15.2 (11.9-18.6)	4.6 (3.2-6.1)	4.2 (1.3-9.9)	28.8 (22.9-34.1)	2.0 (1.0-3.2)
North Africa and Middle East	12.9 (10.5-15.4)	4.7 (3.3-6.2)	9.5 (3.2-20.2)	10.1 (4.3-17.7)	0.7 (-0.5-2.3)
Sub-Saharan Africa	27.9 (24.3-31.3)	6.5 (4.5-8.7)	4.5 (1.3-10.3)	31.6 (24.7-37.3)	0.0 (0.0-0.0)

Central Sub-Saharan Africa	15.5 (11.7-19.2)	5.4 (3.8-7.3)	7.4 (2.7-14.5)	15.3 (9.4-22.3)	0.0 (0.0-0.0)
Southern Sub-Saharan Africa	23.4 (19.0-27.9)	5.0 (3.4-6.8)	8.6 (2.9-18.7)	13.6 (7.1-21.6)	0.0 (0.0-0.0)
Eastern Sub-Saharan Africa	14.4 (10.5-18.6)	4.3 (2.9-5.9)	3.3 (0.8-8.9)	30.7 (23.9-36.7)	2.6 (1.4-4.0)
Western Sub-Saharan Africa	22.7 (19.5-25.7)	6.4 (4.7-8.3)	7.4 (2.2-16.5)	25.7 (17.2-33.0)	0.1 (-0.4-1.1)
Countries in alphabetical order					
Afghanistan	20.1 (16.3-24.2)	5.6 (3.9-7.6)	3.5 (1.0-9.4)	40.2 (33.6-45.7)	0.1 (-0.1-0.4)
Albania	15.4 (12.8-18.3)	4.9 (3.6-6.3)	8.1 (2.8-17.1)	13.0 (6.6-20.2)	0.6 (-0.5-2.1)
Algeria	11.4 (7.5-14.9)	4.1 (2.9-5.7)	5.2 (1.5-11.4)	35.1 (27.5-41.0)	0.5 (-0.4-1.9)
American Samoa	27.8 (25.8-30.5)	4.0 (3.0-5.1)	15.3 (11.8-18.9)	1.7 (0.7-3.3)	10.2 (6.7-15.2)
Andorra	22.3 (20.6-25.1)	4.5 (3.3-5.7)	23.3 (17.4-29.6)	4.9 (2.4-8.3)	10.0 (7.0-13.9)
Angola	24.3 (22.3-26.8)	4.8 (3.5-6.1)	28.5 (20.6-36.1)	0.7 (0.3-1.7)	10.2 (6.4-15.5)
Antigua and Barbuda	22.7 (18.4-29.4)	5.2 (3.8-7.0)	24.6 (15.5-34.5)	2.1 (0.8-4.5)	7.0 (5.4-8.8)
Argentina	23.0 (20.9-26.5)	4.6 (3.5-5.9)	15.3 (11.2-20.0)	7.8 (3.1-14.8)	11.2 (7.4-16.2)
Armenia	24.2 (21.5-28.5)	4.2 (3.0-5.5)	20.1 (14.1-27.5)	2.8 (1.0-6.0)	10.0 (6.7-14.7)
Australia	26.8 (24.1-29.6)	5.5 (4.1-7.0)	20.8 (13.4-29.3)	11.3 (6.1-17.3)	12.9 (6.8-21.1)
Austria	22.5 (19.3-27.8)	4.5 (3.2-5.9)	26.0 (19.2-32.6)	11.8 (5.8-19.5)	11.6 (5.1-20.4)
Azerbaijan	16.7 (12.8-21.2)	3.6 (2.6-4.7)	24.7 (13.7-36.5)	13.3 (7.1-20.7)	10.5 (5.7-16.9)
Bahamas	23.6 (20.8-26.5)	5.9 (4.4-7.7)	26.1 (16.7-36.4)	0.0 (0.0-0.1)	8.1 (6.1-10.2)
Bahrain	17.6 (15.8-19.7)	3.6 (2.6-4.7)	29.9 (19.8-40.0)	3.5 (1.3-7.0)	8.9 (6.4-11.3)
Bangladesh	28.5 (26.6-30.8)	4.0 (3.0-5.1)	18.5 (16.0-21.1)	2.8 (1.0-6.0)	10.2 (6.6-14.1)
Barbados	21.1 (18.4-24.3)	4.1 (3.1-5.2)	14.6 (11.7-17.6)	7.0 (3.1-13.5)	8.8 (6.2-11.6)
Belarus	32.7 (29.9-35.7)	4.5 (3.4-5.8)	22.1 (18.4-25.7)	7.3 (3.0-14.2)	10.5 (6.6-14.9)
Belgium	31.9 (29.3-34.9)	4.2 (3.1-5.4)	17.9 (15.3-20.6)	3.6 (1.1-8.2)	10.4 (6.6-14.5)
Belize	30.8 (28.4-33.4)	5.2 (3.8-6.6)	17.4 (14.5-20.4)	1.0 (0.3-2.5)	9.7 (5.3-14.0)
Benin	27.6 (25.3-30.2)	3.6 (2.6-4.6)	16.0 (13.2-19.0)	0.2 (0.1-0.5)	9.4 (5.7-14.0)
Bermuda	31.6 (29.1-34.2)	4.7 (3.5-6.0)	16.1 (13.4-18.8)	3.2 (0.9-7.8)	9.9 (4.1-15.7)
Bhutan	33.6 (31.1-36.3)	5.5 (4.1-7.0)	25.4 (21.6-28.9)	5.2 (1.9-10.3)	10.8 (6.8-15.0)
Bolivia (Plurinational State of)	37.9 (34.7-41.2)	4.7 (3.5-5.9)	18.9 (15.3-22.7)	5.9 (2.0-12.2)	9.1 (6.3-12.5)

Bosnia and Herzegovina	31.2 (28.6-34.1)	3.8 (2.8-4.9)	22.3 (19.5-25.1)	2.3 (0.7-5.5)	9.9 (5.3-15.3)
Botswana	22.3 (20.1-26.6)	3.7 (2.8-4.8)	14.0 (11.4-16.6)	2.4 (0.7-5.7)	10.8 (6.9-14.9)
Brazil	32.0 (29.5-34.6)	4.3 (3.2-5.5)	21.7 (18.3-24.9)	4.2 (1.4-9.2)	10.6 (6.2-15.1)
Brunei Darussalam	24.3 (22.0-26.9)	3.8 (2.8-4.9)	18.2 (15.3-21.3)	0.2 (0.0-0.5)	9.1 (5.9-13.3)
Bulgaria	30.1 (27.6-32.6)	3.7 (2.7-4.8)	16.2 (13.4-19.1)	1.3 (0.3-3.5)	8.9 (5.3-12.8)
Burkina Faso	28.7 (26.3-31.9)	3.9 (2.9-5.0)	12.0 (7.6-16.5)	0.5 (0.2-1.2)	10.3 (6.1-16.6)
Burundi	28.8 (25.5-32.2)	3.9 (2.9-5.1)	17.2 (13.1-21.6)	0.2 (0.1-0.5)	9.7 (5.8-15.4)
CÃ´te d'Ivoire	29.6 (27.2-32.2)	3.3 (2.4-4.3)	4.0 (1.5-6.7)	1.0 (0.1-3.2)	8.8 (4.5-15.0)
Cabo Verde	27.2 (24.3-30.5)	3.6 (2.6-4.8)	11.8 (8.4-15.5)	1.1 (0.3-2.9)	9.3 (5.3-15.1)
Cambodia	24.6 (22.2-27.2)	3.5 (2.5-4.6)	9.9 (6.6-13.5)	0.5 (0.1-1.3)	9.3 (4.9-15.0)
Cameroon	25.6 (22.9-28.8)	3.5 (2.6-4.5)	13.9 (8.3-19.9)	1.9 (0.7-3.9)	8.9 (3.9-14.5)
Canada	29.4 (26.8-32.7)	4.0 (3.0-5.1)	11.0 (6.4-15.7)	0.4 (0.1-1.0)	10.6 (5.5-18.3)
Caribbean	26.6 (23.6-29.7)	3.9 (2.9-4.9)	14.6 (9.1-20.5)	0.9 (0.3-2.2)	9.4 (6.0-13.3)
Central African Republic	20.3 (18.7-22.1)	2.9 (2.2-3.9)	14.4 (10.7-18.6)	0.0 (0.0-0.1)	6.8 (5.1-8.6)
Chad	18.9 (15.9-22.5)	3.7 (2.6-4.9)	6.4 (1.9-11.3)	0.1 (0.0-0.4)	0.1 (-0.2-0.5)
Chile	20.4 (18.6-22.3)	2.9 (2.1-3.8)	11.8 (8.1-15.7)	0.0 (0.0-0.1)	6.5 (5.0-8.1)
China	20.4 (18.3-22.9)	3.3 (2.4-4.3)	25.7 (21.4-30.2)	0.0 (0.0-0.1)	8.6 (5.4-11.8)
Colombia	14.9 (13.1-16.9)	2.4 (1.7-3.2)	19.1 (12.7-25.6)	0.0 (0.0-0.1)	0.1 (-0.1-0.4)
Comoros	21.6 (19.6-23.9)	2.8 (2.0-3.7)	4.0 (1.0-7.3)	0.0 (0.0-0.1)	5.9 (4.3-7.7)
Congo	21.7 (19.6-23.9)	2.8 (2.0-3.6)	4.2 (1.0-7.7)	0.0 (0.0-0.1)	4.6 (2.8-6.5)
Cook Islands	21.5 (19.1-24.0)	2.9 (2.1-3.9)	3.2 (0.6-6.4)	0.0 (0.0-0.1)	10.8 (7.6-14.4)
Costa Rica	24.4 (22.7-26.4)	2.9 (2.2-3.8)	9.6 (7.1-12.3)	0.0 (0.0-0.1)	10.1 (6.8-13.7)
Croatia	28.0 (24.2-32.0)	3.6 (2.6-4.7)	7.7 (4.2-11.1)	0.0 (0.0-0.1)	10.6 (4.8-18.8)
Cuba	29.4 (27.2-31.8)	3.5 (2.6-4.5)	10.7 (8.1-13.6)	0.0 (0.0-0.1)	9.6 (6.1-14.2)
Cyprus	27.4 (25.2-29.8)	2.8 (2.1-3.7)	11.2 (8.5-14.0)	0.0 (0.0-0.1)	10.7 (5.6-16.2)
Czechia	27.9 (25.5-30.5)	3.9 (3.0-5.0)	14.7 (11.3-18.3)	0.0 (0.0-0.1)	4.6 (0.8-9.1)
Democratic People's Republic of Korea	29.5 (27.0-32.1)	2.7 (2.0-3.6)	8.1 (5.0-11.3)	0.0 (0.0-0.0)	11.2 (4.8-18.1)
Democratic Republic of the Congo	23.4 (21.3-25.6)	2.3 (1.7-3.1)	2.5 (0.5-5.2)	0.0 (0.0-0.1)	11.1 (5.0-19.5)

Denmark	23.8 (21.5-26.2)	2.6 (1.9-3.4)	9.9 (7.3-12.7)	0.0 (0.0-0.1)	10.5 (6.4-14.8)
Djibouti	25.3 (23.0-27.9)	2.7 (2.0-3.6)	10.4 (7.8-13.2)	0.0 (0.0-0.0)	10.1 (5.4-15.0)
Dominica	31.3 (28.8-34.4)	3.8 (2.9-4.9)	12.7 (9.8-15.6)	0.1 (0.0-0.2)	8.6 (6.5-10.6)
Dominican Republic	23.5 (20.9-26.1)	3.0 (2.2-3.9)	2.6 (0.5-5.4)	0.0 (0.0-0.0)	12.4 (5.8-20.6)
Ecuador	25.4 (23.1-27.8)	2.9 (2.1-3.8)	5.8 (3.0-8.7)	0.0 (0.0-0.1)	11.8 (4.9-19.3)
Egypt	20.7 (18.7-23.1)	2.8 (2.0-3.7)	17.7 (14.8-20.7)	0.0 (0.0-0.1)	3.9 (0.1-8.2)
El Salvador	20.9 (19.1-23.0)	3.2 (2.4-4.2)	13.4 (11.0-16.0)	0.1 (0.0-0.2)	8.9 (6.7-11.1)
Equatorial Guinea	25.8 (22.2-29.5)	3.2 (2.4-4.2)	8.7 (5.8-11.6)	0.0 (0.0-0.1)	9.9 (4.4-16.3)
Eritrea	24.5 (22.1-27.3)	2.9 (2.1-3.6)	11.9 (8.9-14.9)	0.0 (0.0-0.1)	4.4 (-1.8-11.2)
Estonia	25.7 (23.2-28.2)	2.8 (2.1-3.8)	10.8 (8.1-13.7)	0.0 (0.0-0.0)	10.8 (4.7-17.6)
Eswatini	21.4 (19.6-23.4)	2.4 (1.8-3.2)	3.7 (1.4-6.4)	0.0 (0.0-0.0)	9.6 (5.0-15.9)
Ethiopia	18.9 (16.7-21.3)	3.1 (2.3-4.0)	5.9 (3.5-8.5)	0.1 (0.0-0.2)	8.9 (6.8-11.0)
Fiji	25.4 (22.9-28.5)	3.1 (2.3-4.1)	7.6 (5.2-10.1)	0.2 (0.0-0.4)	8.9 (6.6-11.2)
Finland	22.0 (19.5-24.5)	2.0 (1.5-2.7)	2.4 (0.6-4.8)	0.0 (0.0-0.0)	11.0 (6.2-17.6)
France	23.9 (21.5-26.4)	2.7 (2.0-3.5)	8.0 (5.4-10.7)	0.0 (0.0-0.0)	10.3 (6.5-15.3)
Gabon	25.1 (23.0-27.4)	3.1 (2.3-4.0)	8.2 (5.5-11.0)	0.0 (0.0-0.0)	11.9 (6.5-17.8)
Gambia	26.6 (24.0-28.9)	4.2 (3.1-5.4)	15.3 (10.9-20.0)	0.9 (0.3-2.0)	10.2 (7.5-13.6)
Georgia	26.6 (24.0-29.2)	4.1 (3.0-5.3)	13.6 (8.8-19.0)	0.8 (0.3-1.8)	9.9 (7.2-13.1)
Germany	26.4 (23.5-29.2)	4.3 (3.1-5.7)	22.1 (18.1-26.4)	1.3 (0.4-3.1)	12.0 (8.2-17.1)
Ghana	27.3 (24.7-30.0)	4.3 (3.1-5.5)	8.3 (4.4-13.0)	0.6 (0.2-1.5)	7.2 (4.1-10.4)
Greece	25.7 (23.6-27.8)	2.5 (1.8-3.2)	5.4 (2.9-8.1)	0.0 (0.0-0.1)	7.8 (6.0-10.0)
Greenland	24.7 (22.4-27.2)	2.5 (1.8-3.3)	4.6 (2.2-7.3)	0.0 (0.0-0.0)	11.8 (3.7-21.3)
Grenada	25.8 (23.7-27.9)	2.5 (1.8-3.2)	5.5 (3.0-8.2)	0.0 (0.0-0.1)	7.5 (5.7-9.4)
Guam	14.4 (12.7-16.1)	3.2 (2.3-4.2)	14.8 (11.9-17.8)	6.3 (4.0-9.0)	2.7 (1.6-4.0)
Guatemala	12.5 (10.2-14.9)	2.7 (1.9-3.5)	11.2 (6.2-17.9)	16.7 (10.5-23.5)	0.6 (-0.1-1.6)
Guinea	12.0 (10.2-13.8)	2.8 (2.0-3.6)	17.4 (7.0-29.5)	0.3 (0.1-0.7)	0.1 (-0.3-0.7)
Guinea-Bissau	10.8 (8.9-13.0)	3.4 (2.4-4.5)	16.5 (5.0-30.0)	0.4 (0.1-0.9)	0.4 (-0.4-1.5)
Guyana	10.7 (9.3-12.3)	2.3 (1.7-3.0)	20.5 (9.2-33.1)	0.0 (0.0-0.1)	0.2 (-0.3-0.8)

Haiti	10.8 (9.1-12.5)	3.4 (2.5-4.5)	18.6 (7.7-31.3)	6.2 (3.1-10.7)	0.5 (-0.1-1.3)
Honduras	26.0 (23.4-28.7)	3.8 (2.7-4.9)	17.3 (8.9-27.9)	0.6 (0.2-1.4)	0.4 (-0.2-1.3)
Hungary	11.3 (9.7-13.0)	2.5 (1.8-3.3)	17.5 (7.7-29.5)	2.2 (0.8-5.2)	0.4 (-0.8-2.2)
Iceland	15.2 (12.9-17.8)	2.9 (2.1-3.8)	16.6 (7.8-28.0)	4.4 (2.0-8.1)	1.2 (0.0-2.6)
India	11.3 (9.6-13.1)	2.8 (2.0-3.8)	20.9 (8.4-35.0)	1.0 (0.4-2.0)	0.2 (-0.3-0.8)
Indonesia	12.0 (10.2-13.9)	3.4 (2.4-4.5)	19.0 (8.4-32.0)	2.6 (1.2-4.7)	0.6 (-0.2-1.6)
Iran (Islamic Republic of)	5.4 (3.8-7.2)	1.9 (1.3-2.6)	5.2 (1.9-10.4)	34.6 (27.3-40.5)	0.5 (-0.4-1.8)
Iraq	14.3 (12.6-16.2)	3.5 (2.5-4.6)	14.9 (9.9-21.0)	3.3 (1.3-7.0)	0.1 (-0.3-0.9)
Ireland	12.7 (11.0-14.6)	2.8 (1.9-3.8)	20.9 (9.3-33.8)	1.2 (0.5-2.6)	0.2 (-0.3-0.8)
Israel	10.4 (8.4-12.4)	3.0 (2.1-4.0)	20.2 (8.2-33.5)	1.5 (0.6-3.0)	0.2 (-0.3-0.8)
Italy	19.5 (16.7-22.5)	4.6 (3.3-6.0)	19.5 (9.8-31.2)	3.6 (1.5-6.9)	0.2 (-0.3-0.9)
Jamaica	15.5 (13.5-17.7)	3.8 (2.6-5.0)	21.0 (7.8-36.6)	0.0 (0.0-0.1)	0.0 (-0.2-0.5)
Japan	6.3 (5.3-7.4)	2.1 (1.4-2.8)	20.7 (15.7-25.7)	6.4 (3.5-9.9)	6.0 (4.4-7.9)
Jordan	5.8 (4.6-7.1)	2.0 (1.3-2.7)	18.5 (11.7-25.5)	11.1 (6.3-16.7)	6.2 (4.4-8.2)
Kazakhstan	8.2 (6.7-9.7)	2.0 (1.4-2.7)	18.7 (13.2-24.4)	2.2 (0.9-4.5)	4.6 (3.3-6.0)
Kenya	4.9 (3.9-6.0)	2.2 (1.5-3.0)	24.0 (18.1-29.9)	6.7 (3.3-11.2)	7.2 (5.1-9.7)
Kiribati	10.3 (8.9-11.7)	2.8 (2.0-3.8)	18.1 (14.5-21.6)	6.2 (4.1-8.9)	3.5 (2.3-4.9)
Kuwait	10.9 (9.4-12.5)	2.9 (2.1-3.8)	19.7 (15.2-24.8)	3.9 (1.7-7.2)	3.2 (2.1-4.3)
Kyrgyzstan	13.8 (12.1-15.5)	3.6 (2.6-4.8)	17.6 (13.5-21.9)	2.0 (0.8-4.3)	2.1 (0.8-3.8)
Lao People's Democratic Republic	8.4 (7.0-10.2)	2.1 (1.5-2.9)	18.4 (12.2-25.3)	6.2 (3.4-9.8)	0.2 (-0.2-0.7)
Latvia	6.6 (5.2-8.3)	2.2 (1.5-3.1)	13.7 (8.2-19.3)	19.2 (13.1-26.2)	2.5 (1.4-3.8)
Lebanon	9.9 (8.2-11.9)	3.5 (2.6-4.7)	10.6 (5.8-16.0)	24.0 (17.8-29.4)	1.6 (-0.4-4.3)
Lesotho	9.5 (7.8-11.2)	2.5 (1.6-3.4)	17.8 (14.0-21.8)	5.2 (3.0-8.2)	5.3 (3.6-7.1)
Liberia	9.3 (7.6-11.1)	3.3 (2.3-4.5)	11.8 (6.7-18.2)	21.4 (14.4-28.0)	0.5 (-0.3-1.5)
Libya	8.0 (6.8-9.4)	2.2 (1.6-2.8)	12.4 (8.0-16.8)	3.1 (1.2-6.1)	0.8 (0.0-1.7)
Lithuania	13.5 (11.3-15.7)	3.6 (2.6-4.9)	21.8 (15.2-29.1)	0.2 (0.1-0.4)	1.2 (0.3-2.4)
Luxembourg	19.8 (17.6-22.2)	4.0 (2.9-5.2)	11.6 (8.2-15.0)	4.3 (2.1-7.3)	1.8 (0.5-3.4)
Madagascar	19.9 (17.6-22.3)	3.9 (2.9-5.2)	11.6 (8.2-15.0)	4.0 (1.9-7.0)	1.8 (0.5-3.4)

Malawi	16.5 (13.6-19.7)	4.3 (3.1-5.7)	11.4 (7.6-16.1)	13.9 (7.2-21.2)	2.2 (-0.2-5.1)
Malaysia	14.6 (12.9-16.5)	4.7 (3.5-6.0)	25.3 (21.5-29.0)	4.8 (2.5-7.3)	5.8 (3.7-8.1)
Maldives	13.0 (10.1-16.0)	6.3 (4.6-8.1)	28.4 (21.1-35.5)	0.1 (0.0-0.2)	4.1 (0.7-7.2)
Mali	16.6 (13.8-19.9)	4.5 (3.3-5.8)	42.6 (37.3-47.5)	0.1 (0.0-0.3)	2.4 (-1.6-6.0)
Malta	7.9 (5.5-12.8)	3.5 (2.2-5.2)	24.7 (17.7-33.5)	0.0 (0.0-0.0)	3.8 (-0.1-8.2)
Marshall Islands	14.1 (12.5-16.2)	4.7 (3.5-5.9)	30.9 (27.3-34.4)	0.1 (0.0-0.1)	7.6 (5.9-9.5)
Mauritania	16.0 (13.6-19.4)	5.8 (4.3-7.6)	34.7 (29.4-40.2)	0.1 (0.0-0.2)	4.4 (1.1-7.8)
Mauritius	23.7 (20.3-26.9)	5.4 (4.0-7.1)	28.9 (24.5-33.0)	0.0 (0.0-0.0)	4.2 (1.1-7.8)
Mexico	19.1 (16.5-21.5)	5.6 (4.2-7.2)	42.8 (38.7-47.0)	0.0 (0.0-0.1)	3.0 (-2.4-7.9)
Micronesia (Federated States of)	29.4 (25.9-33.3)	5.6 (4.2-7.2)	27.1 (21.8-32.4)	0.1 (0.0-0.2)	5.8 (3.8-8.1)
Monaco	11.5 (8.6-15.5)	6.1 (4.3-8.2)	28.9 (21.7-35.9)	0.1 (0.0-0.2)	3.4 (-0.7-8.1)
Mongolia	10.0 (7.8-12.6)	4.4 (3.1-5.8)	28.2 (23.3-33.0)	1.8 (0.8-3.6)	6.5 (4.3-9.1)
Montenegro	15.3 (12.8-17.8)	5.9 (4.5-7.6)	26.9 (21.2-32.5)	0.4 (0.2-0.9)	4.1 (0.4-8.3)
Morocco	7.6 (6.0-9.7)	3.1 (2.1-4.2)	29.6 (22.7-36.5)	0.1 (0.0-0.3)	2.8 (-3.9-8.7)
Mozambique	17.1 (14.5-19.9)	5.3 (3.8-7.0)	49.3 (44.2-53.8)	0.0 (0.0-0.0)	3.0 (-3.9-9.1)
Myanmar	13.2 (9.8-16.8)	6.7 (4.7-9.0)	45.8 (40.6-50.7)	0.1 (0.0-0.3)	2.9 (-0.2-6.1)
Namibia	11.0 (8.6-14.4)	2.8 (2.0-3.8)	15.5 (12.0-19.6)	0.0 (0.0-0.1)	6.3 (2.8-10.2)
Nauru	16.9 (12.5-20.8)	5.7 (4.2-7.4)	27.5 (21.2-33.8)	0.1 (0.0-0.2)	5.0 (1.2-9.3)
Nepal	27.3 (24.7-30.0)	4.7 (3.5-6.0)	25.0 (21.2-28.7)	0.1 (0.0-0.4)	9.7 (7.0-12.5)
Netherlands	20.4 (16.2-25.2)	6.4 (4.3-8.9)	41.8 (34.7-49.4)	0.0 (0.0-0.0)	3.3 (-5.3-10.9)
New Zealand	17.6 (14.2-21.0)	5.2 (3.8-6.9)	18.4 (9.1-27.5)	16.1 (9.9-23.1)	2.2 (-0.2-4.5)
Nicaragua	13.3 (10.4-16.1)	4.3 (3.1-5.8)	25.4 (19.4-31.0)	19.6 (13.9-25.6)	2.5 (-0.2-4.9)
Niger	8.2 (5.2-11.0)	5.2 (3.6-7.0)	10.3 (4.5-18.7)	33.6 (25.2-41.4)	8.9 (6.2-12.2)
Nigeria	16.3 (12.1-20.8)	4.2 (3.0-5.8)	20.7 (13.4-28.0)	25.6 (18.5-33.2)	1.6 (-0.4-3.6)
Niue	8.8 (6.6-11.0)	2.8 (2.0-3.7)	17.0 (10.3-23.6)	21.8 (15.1-29.2)	11.5 (8.1-15.9)
North Macedonia	12.5 (9.6-15.1)	4.3 (3.1-5.8)	27.8 (22.1-33.1)	17.6 (12.3-23.3)	2.0 (-0.5-4.4)
Northern Ireland	18.0 (15.4-20.6)	3.5 (2.4-4.7)	21.0 (13.1-28.9)	25.0 (17.0-33.5)	9.0 (6.1-12.4)
Northern Mariana Islands	12.2 (8.7-15.8)	4.4 (3.2-6.0)	20.5 (14.0-27.3)	21.3 (14.6-28.3)	4.8 (0.3-9.2)

Norway	7.6 (5.8-10.1)	2.2 (1.5-3.0)	9.4 (6.0-13.7)	28.9 (22.1-35.5)	2.1 (0.9-3.5)
Oman	8.0 (5.6-10.9)	1.9 (1.2-2.6)	9.7 (4.8-16.3)	32.0 (25.2-38.5)	1.6 (0.2-3.4)
Pakistan	10.6 (8.3-13.9)	2.9 (2.0-4.1)	13.6 (6.9-21.7)	19.8 (13.9-26.0)	2.1 (0.2-4.6)
Palau	7.4 (4.3-10.5)	2.2 (1.4-3.1)	5.6 (1.8-12.5)	39.4 (30.5-46.9)	0.4 (-0.1-1.3)
Palestine	8.9 (6.3-12.3)	2.6 (1.8-3.6)	21.9 (12.6-32.7)	19.1 (11.3-27.6)	0.6 (-0.5-2.2)
Panama	7.0 (4.4-10.4)	1.4 (0.9-2.1)	7.6 (3.1-14.2)	36.7 (29.2-43.1)	1.7 (0.2-3.5)
Papua New Guinea	8.3 (5.9-12.1)	2.5 (1.7-3.5)	28.8 (18.7-39.0)	5.7 (2.4-11.0)	0.8 (-1.2-3.7)
Paraguay	9.9 (7.7-12.7)	2.8 (1.9-3.8)	29.4 (19.1-39.6)	2.4 (1.0-4.7)	0.5 (-0.4-1.9)
Peru	8.3 (6.3-10.9)	2.2 (1.5-3.2)	6.3 (3.5-10.5)	37.2 (31.2-42.8)	2.7 (1.2-4.6)
Philippines	7.4 (5.2-10.1)	1.8 (1.2-2.6)	4.3 (1.4-9.6)	40.7 (33.0-47.2)	3.8 (0.1-8.6)
Poland	9.4 (6.2-13.9)	3.8 (2.7-5.2)	6.5 (3.2-11.0)	35.4 (29.3-40.8)	0.3 (-0.7-2.1)
Portugal	16.0 (11.2-21.5)	4.1 (2.7-5.7)	26.3 (14.1-38.2)	10.8 (5.3-18.1)	1.4 (-1.4-4.0)
Puerto Rico	7.2 (4.2-10.8)	2.4 (1.6-3.2)	11.7 (5.1-20.5)	31.3 (23.1-38.3)	1.4 (0.1-2.7)
Qatar	3.7 (2.5-5.4)	1.3 (0.9-1.9)	6.0 (3.0-10.6)	37.4 (30.7-43.5)	3.5 (2.3-5.1)
Republic of Korea	9.0 (6.8-12.1)	2.1 (1.4-3.1)	9.4 (5.7-14.2)	29.9 (23.6-35.3)	3.5 (1.9-5.4)
Republic of Moldova	8.0 (5.4-11.4)	2.7 (1.7-4.0)	4.6 (2.1-8.6)	40.7 (34.6-46.3)	2.7 (1.0-4.7)
Romania	9.3 (6.1-13.1)	2.3 (1.5-3.2)	4.5 (1.9-8.7)	38.1 (32.0-43.8)	2.9 (0.4-5.8)
Russian Federation	16.1 (14.1-18.1)	5.2 (3.8-6.8)	15.9 (8.6-23.9)	0.4 (0.1-0.9)	1.2 (-2.2-6.5)
Rwanda	10.4 (7.6-14.0)	2.3 (1.5-3.3)	3.5 (1.4-7.2)	42.4 (36.3-48.5)	1.7 (-0.1-4.0)
Saint Kitts and Nevis	12.6 (9.6-16.1)	2.5 (1.7-3.5)	9.3 (3.8-16.9)	36.2 (27.7-43.0)	4.9 (1.8-9.2)
Saint Lucia	19.5 (15.7-23.4)	4.1 (2.9-5.4)	16.4 (9.0-24.6)	0.2 (0.1-0.4)	0.1 (-0.2-0.4)
Saint Vincent and the Grenadines	8.2 (4.5-12.9)	2.5 (1.6-3.6)	1.4 (0.3-4.7)	47.2 (38.1-57.7)	0.7 (0.0-1.6)
Samoa	12.9 (9.8-16.4)	2.9 (1.9-4.2)	6.3 (3.1-11.2)	36.2 (29.3-42.2)	2.4 (0.4-5.2)
San Marino	7.0 (4.9-9.7)	2.1 (1.3-3.0)	7.8 (3.7-13.6)	34.7 (27.5-41.1)	1.7 (-0.1-4.1)
Sao Tome and Principe	10.1 (7.7-12.9)	2.8 (1.8-4.0)	11.3 (5.7-18.5)	30.3 (22.7-36.8)	2.6 (-0.8-7.0)
Saudi Arabia	13.2 (11.3-15.9)	3.4 (2.5-4.5)	17.7 (13.7-22.2)	13.9 (9.7-19.2)	5.0 (3.3-7.1)
Scotland	16.9 (13.9-20.9)	4.6 (3.3-6.3)	20.0 (13.9-26.9)	12.9 (6.8-20.6)	2.6 (-1.0-7.0)
Senegal	15.3 (11.3-19.5)	4.2 (2.9-5.7)	13.3 (7.6-20.1)	24.4 (17.6-30.8)	11.3 (7.1-15.7)

Serbia	10.6 (9.0-12.3)	2.9 (2.0-3.9)	17.1 (10.9-23.8)	16.1 (9.1-24.3)	3.1 (0.3-6.5)
Seychelles	14.8 (12.9-17.0)	3.7 (2.6-4.9)	25.5 (21.2-29.9)	4.1 (2.1-6.7)	6.2 (4.8-7.9)
Sierra Leone	6.1 (4.7-7.8)	2.0 (1.4-2.9)	14.7 (9.2-20.7)	19.9 (12.5-27.5)	5.0 (1.9-8.6)
Singapore	10.8 (7.8-16.2)	3.0 (2.0-4.2)	7.4 (3.7-12.6)	25.8 (19.8-31.7)	3.3 (0.6-6.7)
Slovakia	5.5 (3.2-7.8)	1.9 (1.2-2.7)	11.1 (6.2-17.0)	21.5 (13.7-28.4)	0.9 (-0.6-2.4)
Slovenia	5.3 (3.0-7.9)	1.8 (1.0-2.6)	6.7 (2.7-13.0)	25.5 (15.6-34.0)	0.3 (-0.2-0.9)
Solomon Islands	5.3 (2.6-8.8)	1.8 (0.9-2.9)	4.1 (1.3-9.3)	26.9 (13.7-37.6)	0.9 (-0.9-2.5)
Somalia	6.8 (4.2-9.8)	1.9 (1.2-2.9)	16.6 (9.4-24.4)	18.0 (11.2-25.8)	1.0 (-0.3-2.8)
South Africa	7.6 (5.8-9.7)	2.4 (1.7-3.4)	29.0 (20.4-36.5)	8.6 (4.6-13.9)	1.9 (-1.8-6.6)
South Sudan	5.6 (2.7-8.6)	1.8 (0.9-2.8)	3.9 (1.2-9.2)	25.6 (13.2-35.9)	2.4 (-1.4-5.9)
Spain	9.7 (6.3-13.0)	2.9 (1.9-4.1)	10.5 (4.7-18.3)	22.7 (14.9-30.2)	0.2 (-0.1-0.6)
Sri Lanka	9.3 (6.2-13.6)	3.3 (2.2-4.6)	9.8 (4.8-16.8)	27.9 (20.1-35.2)	0.5 (-0.2-1.2)
Suriname	4.4 (3.3-5.9)	1.9 (1.3-2.7)	21.2 (13.5-29.0)	18.4 (11.5-25.7)	0.2 (-0.1-0.5)
Sweden	8.6 (4.1-13.5)	2.1 (1.1-3.0)	5.1 (1.8-10.8)	26.0 (15.0-35.4)	0.4 (-0.1-1.1)
Switzerland	4.0 (2.6-5.9)	2.6 (1.7-3.7)	7.1 (3.0-13.4)	30.7 (21.9-38.7)	0.4 (-0.3-1.1)
Syrian Arab Republic	6.5 (4.1-9.4)	2.0 (1.3-2.9)	8.3 (3.8-15.8)	31.4 (23.3-38.9)	0.3 (-0.5-1.5)
Taiwan (Province of China)	4.8 (2.1-8.1)	1.7 (0.9-2.8)	4.0 (1.2-9.4)	25.0 (13.4-34.9)	2.9 (-4.2-9.1)
Tajikistan	11.9 (8.9-15.6)	2.5 (1.7-3.6)	21.8 (13.1-30.9)	17.2 (10.3-25.1)	2.9 (-2.8-8.0)
Thailand	3.7 (1.4-6.2)	1.6 (0.6-2.5)	3.2 (0.7-8.6)	25.4 (10.5-37.9)	2.7 (-2.9-7.8)
Timor-Leste	4.0 (2.2-5.9)	1.6 (0.9-2.3)	14.1 (7.6-21.1)	17.9 (10.0-25.7)	0.4 (-0.5-1.5)
Togo	5.0 (3.5-7.1)	1.6 (1.1-2.4)	14.0 (7.5-21.8)	22.9 (15.7-29.5)	0.2 (-0.5-1.4)
Tokelau	8.8 (5.8-12.5)	3.5 (2.4-4.8)	10.8 (5.1-18.5)	26.6 (18.6-34.4)	0.7 (-0.5-1.8)
Tonga	8.4 (4.2-12.4)	2.3 (1.2-3.5)	5.5 (2.0-11.2)	25.2 (14.3-34.3)	0.2 (-0.2-0.9)
Trinidad and Tobago	8.8 (6.0-12.1)	2.7 (1.8-3.8)	9.9 (4.8-16.6)	28.7 (20.5-36.1)	0.2 (-0.1-0.5)
Tunisia	22.2 (19.0-25.4)	5.3 (3.8-7.0)	5.5 (2.3-10.6)	4.4 (1.0-10.4)	0.2 (-0.2-0.5)
Turkey	16.6 (14.7-18.6)	2.8 (2.0-3.8)	5.0 (0.9-9.6)	0.5 (0.1-1.4)	2.6 (-3.9-11.0)
Turkmenistan	18.9 (15.6-22.2)	5.2 (3.8-6.9)	4.7 (0.9-10.5)	1.2 (0.2-3.3)	0.6 (-1.5-3.4)
Tuvalu	38.6 (34.7-42.7)	5.4 (3.7-7.2)	4.5 (0.4-12.3)	0.3 (0.0-0.9)	16.1 (1.9-29.7)

Uganda	19.0 (15.7-22.3)	5.1 (3.7-6.8)	7.0 (3.0-11.5)	1.1 (0.2-3.1)	0.1 (-0.1-0.4)
Ukraine	25.2 (21.5-29.4)	3.0 (2.2-4.0)	10.1 (6.1-14.2)	0.0 (0.0-0.0)	8.1 (6.2-10.0)
United Arab Emirates	20.4 (16.2-25.1)	5.1 (3.5-7.0)	4.0 (1.2-8.7)	1.4 (0.3-3.6)	0.1 (0.0-0.2)
United Kingdom	16.3 (13.3-19.4)	5.5 (4.0-7.3)	5.0 (1.1-10.9)	1.1 (0.2-2.9)	0.3 (-0.8-1.9)
United Republic of Tanzania	21.5 (18.0-25.0)	5.1 (3.5-6.9)	7.8 (4.1-11.9)	4.1 (1.1-9.8)	0.2 (-0.1-0.6)
United States of America	18.0 (14.7-21.4)	6.1 (4.4-8.1)	4.9 (0.0-12.2)	0.0 (0.0-0.0)	0.0 (-0.1-0.0)
United States Virgin Islands	13.9 (11.5-16.5)	2.4 (1.8-3.3)	4.2 (1.0-7.5)	0.0 (0.0-0.0)	0.3 (-0.5-1.7)
Uruguay	9.6 (8.1-11.1)	3.1 (2.1-4.3)	7.8 (3.6-12.6)	0.6 (0.1-1.7)	0.3 (-0.2-1.1)
Uzbekistan	24.6 (20.9-29.1)	3.3 (2.4-4.3)	8.4 (3.5-13.5)	0.0 (0.0-0.1)	7.8 (5.9-9.9)
Vanuatu	17.3 (14.1-20.7)	5.9 (4.3-7.8)	4.3 (0.0-11.8)	0.0 (0.0-0.0)	0.1 (-0.2-0.4)
Venezuela (Bolivarian Republic of)	19.2 (15.9-22.9)	5.7 (4.0-7.4)	4.9 (2.2-9.9)	4.1 (1.4-8.1)	0.1 (-0.1-0.3)
Viet Nam	12.7 (10.2-15.6)	2.8 (2.0-3.8)	7.5 (3.7-11.4)	0.2 (0.0-0.6)	0.2 (-0.3-0.9)
Yemen	26.5 (24.0-29.5)	3.5 (2.5-4.7)	6.3 (3.1-9.7)	0.0 (0.0-0.0)	
Zambia	29.3 (26.9-31.9)	3.2 (2.3-4.3)	4.1 (1.3-7.2)	0.0 (0.0-0.0)	12.0 (7.2-17.4)
Zimbabwe	8.6 (5.6-13.0)	2.4 (1.5-3.4)	7.3 (3.1-13.3)	36.3 (28.3-43.8)	0.3 (-0.1-0.8)

Supplement Table 13. Age-standardised rates (per 100,000 people) and percentages of DALYs (with 95% uncertainty intervals) due to stroke in 2019 attributable to metabolic, behavioural and environmental/occupational risk clusters and all risk factors combined in low-income and middle-income countries, high-income countries and globally by sex

Rates	Low-income countries		Low-middle income countries		Upper middle-income countries		High-income countries		Globally	
	Males	Females	Males	Females	Males	Females	Males	Females	Males	Females
Metabolic risks	1630.0 (1340.0-1940.0)	1689.6 (1465.0-1909.2)	1460.0 (1260.0-1640.0)	1726.5 (1443.5-2019.3)	1170.0 (984.0-1360.0)	552.1 (486.3-617.8)	436.0 (372.0-500.0)	1419.7 (1236.9-1587.6)	1080.0 (936.0-1210.0)	1630.0 (1340.0-1940.0)
Behavioural risks	824.0 (594.0-1130.0)	1178.2 (998.1-1372.5)	673.0 (521.0-857.0)	1468.8 (1216.8-1753.8)	650.0 (506.0-827.0)	394.8 (347.8-449.3)	268.0 (229.0-316.0)	1105.5 (951.7-1274.0)	567.0 (457.0-711.0)	824.0 (594.0-1130.0)
Environmental and occupational risks	1180.0 (980.0-1400.0)	960.6 (838.7-1093.5)	792.0 (684.0-913.0)	967.6 (808.4-1132.7)	591.0 (495.0-693.0)	162.5 (136.4-191.0)	118.0 (96.7-141.0)	776.8 (682.6-872.9)	551.0 (484.0-626.0)	1180.0 (980.0-1400.0)
All risks combined	2080.0 (1760.0-2420.0)	2078.3 (1878.4-2282.3)	1720.0 (1540.0-1910.0)	2205.7 (1901.3-2514.1)	1420.0 (1240.0-1610.0)	663.4 (612.9-717.5)	526.0 (469.0-582.0)	1777.4 (1616.7-1925.3)	1300.0 (1170.0-1420.0)	2080.0 (1760.0-2420.0)
Percentages										
Metabolic risks	67.3 (60.4-73.3)	66.6 (60.1-72.7)	71.7 (65.3-77.3)	72.8 (66.5-78.5)	69.2 (62.0-75.7)	69.6 (62.3-76.9)	71.4 (64.2-78.1)	68.1 (60.8-75.4)	70.1 (63.7-76.3)	70.3 (63.8-76.5)
Behavioural risks	44.4 (37.4-53.2)	33.6 (25.8-43.8)	50.0 (43.9-56.7)	33.6 (27.0-41.8)	58.8 (52.7-64.9)	38.5 (31.2-46.6)	51.1 (45.2-56.9)	41.9 (36.6-47.8)	54.6 (48.8-60.7)	37.0 (30.6-44.8)
Environmental and occupational risks	49.1 (45.6-52.6)	47.9 (44.4-51.9)	40.8 (37.4-44.1)	39.6 (36.5-42.8)	38.7 (35.6-42.2)	35.0 (31.8-38.2)	21.0 (17.9-24.6)	18.4 (15.4-21.7)	38.4 (35.3-41.5)	36.0 (33.1-39.0)
All risks combined	87.0 (84.2-89.4)	84.9 (81.8-87.9)	88.2 (85.6-90.7)	86.2 (83.0-89.2)	88.4 (85.6-91.1)	84.3 (80.5-88.2)	85.8 (82.3-89.5)	82.1 (77.8-86.5)	87.8 (85.1-90.2)	84.6 (81.3-88.0)

Supplement Table 14. Age-standardised rates (per 100,000 people) and percentages of DALYs (with 95% uncertainty intervals) due to ischaemic stroke in 2019 attributable to metabolic, behavioural and environmental/occupational risk clusters and all risk factors combined in low-income and middle-income countries, high-income countries and globally by sex

Rates	Low-income countries		Low-middle income countries		Upper middle-income countries		High-income countries		Globally	
	Males	Females	Males	Females	Males	Females	Males	Females	Males	Females
Metabolic risks	572.6 (447.1-749.8)	573.0 (459.0-695.0)	656.4 (532.3-784.7)	601.0 (503.0-694.0)	823.4 (667.0-979.5)	643.0 (532.0-762.0)	281.1 (235.6-334.7)	241.0 (196.0-294.0)	615.6 (514.3-717.8)	512.0 (430.0-592.0)
Behavioural risks	327.6 (245.7-433.4)	252.0 (174.0-352.0)	405.8 (320.9-508.8)	246.0 (180.0-321.0)	634.9 (518.3-753.6)	329.0 (252.0-424.0)	181.0 (150.1-214.9)	131.0 (103.0-162.0)	435.2 (366.0-508.2)	248.0 (190.0-317.0)
Environmental and occupational risks	392.3 (318.4-486.9)	385.0 (321.0-463.0)	341.5 (279.7-412.3)	290.0 (247.0-335.0)	427.7 (356.1-497.0)	297.0 (249.0-350.0)	76.3 (63.6-89.6)	58.5 (47.3-70.5)	303.5 (262.0-346.4)	230.0 (200.0-264.0)
All risks combined	729.4 (599.4-915.1)	719.0 (606.0-844.0)	799.3 (678.0-933.9)	701.0 (610.0-783.0)	1035.8 (901.3-1173.6)	768.0 (661.0-874.0)	333.6 (295.9-373.9)	283.0 (241.0-327.0)	759.9 (676.4-840.3)	606.0 (533.0-674.0)
Percentages										
Metabolic risks	66.9 (57.4-76.6)	66.0 (56.9-75.7)	71.1 (61.7-80.8)	72.5 (63.6-81.6)	69.2 (59.0-78.6)	70.0 (60.2-79.8)	70.5 (60.6-81.6)	68.6 (58.3-79.6)	70.1 (60.7-79.4)	70.5 (61.3-80.2)
Behavioural risks	38.3 (30.5-47.5)	29.1 (20.8-39.2)	43.9 (37.3-51.0)	29.6 (22.7-38.1)	53.4 (46.2-60.2)	35.8 (27.9-44.1)	45.4 (38.4-52.4)	37.3 (30.3-44.9)	49.5 (42.8-56.3)	34.1 (26.6-42.1)
Environmental and occupational risks	45.8 (42.3-49.7)	44.4 (40.8-48.5)	37.0 (33.6-40.5)	35.0 (32.0-38.1)	35.9 (32.8-39.4)	32.3 (29.4-35.6)	19.1 (16.2-22.5)	16.7 (13.9-19.8)	34.5 (31.7-37.8)	31.6 (28.9-34.7)
All risks combined	85.2 (81.0-89.9)	82.9 (78.0-88.0)	86.6 (82.4-91.3)	84.6 (79.7-89.9)	87.1 (82.6-91.4)	83.6 (78.2-89.3)	83.6 (77.8-90.3)	80.5 (74.0-87.7)	86.5 (82.2-91.1)	83.4 (78.1-88.8)

Supplement Table 15. Age-standardised rates (per 100,000 people) and percentages of DALYs (with 95% uncertainty intervals) due to intracerebral haemorrhage in 2019 attributable to metabolic, behavioural and environmental/occupational risk clusters and all risk factors combined in low-income and middle-income countries, high-income countries and globally by sex

Rates	Low-income countries		Low-middle income countries		Upper middle-income countries		High-income countries		Globally	
	Males	Females	Males	Females	Males	Females	Males	Females	Males	Females
Metabolic risks	1180.4 (930.9-1437.7)	989.0 (771.0-1220.0)	922.6 (771.8-1076.2)	755.0 (636.0-873.0)	802.6 (651.2-960.4)	450.0 (366.0-540.0)	201.0 (176.5-222.8)	120.0 (104.0-136.0)	707.5 (607.7-803.7)	480.0 (413.0-549.0)
Behavioural risks	830.4 (634.1-1062.7)	532.0 (364.0-744.0)	691.3 (565.3-821.7)	373.0 (275.0-490.0)	750.7 (607.3-905.5)	270.0 (201.0-355.0)	162.4 (142.4-184.9)	82.7 (70.8-96.3)	595.8 (509.2-696.0)	267.0 (205.0-339.0)
Environmental and occupational risks	880.5 (721.8-1047.9)	734.0 (588.0-895.0)	545.1 (460.9-639.2)	437.0 (369.0-507.0)	483.2 (399.2-573.7)	252.0 (207.0-301.0)	65.0 (54.4-76.6)	37.0 (30.8-43.9)	417.8 (363.0-471.9)	275.0 (240.0-313.0)
All risks combined	1533.6 (1278.6-1811.2)	1270.0 (1040.0-1530.0)	1138.6 (976.3-1292.6)	899.0 (776.0-1010.0)	1042.3 (882.8-1215.5)	554.0 (472.0-649.0)	245.4 (229.6-263.6)	148.0 (135.0-161.0)	895.7 (804.4-985.1)	583.0 (521.0-645.0)
Percentages										
Metabolic risks	67.9 (59.8-74.8)	67.1 (58.9-74.5)	72.8 (65.1-79.1)	73.7 (66.2-80.0)	69.4 (60.4-77.4)	69.5 (60.7-77.1)	73.0 (65.1-79.6)	68.7 (60.6-75.7)	70.7 (62.9-77.7)	70.8 (63.2-77.1)
Behavioural risks	47.8 (39.6-57.1)	36.1 (26.7-47.6)	54.5 (47.4-62.0)	36.4 (27.8-46.3)	64.9 (58.1-71.5)	41.7 (33.1-51.2)	59.0 (52.3-65.8)	47.3 (41.0-54.2)	59.5 (53.0-66.3)	39.5 (31.4-48.8)
Environmental and occupational risks	50.6 (47.2-54.2)	49.8 (46.2-53.8)	43.0 (39.3-46.7)	42.6 (39.2-46.1)	41.8 (38.4-45.2)	38.9 (35.8-42.2)	23.6 (20.1-27.4)	21.2 (17.9-24.7)	41.7 (38.5-45.0)	40.7 (37.6-43.9)
All risks combined	88.2 (85.3-90.6)	86.2 (82.7-89.2)	89.8 (87.0-92.0)	87.7 (84.4-90.5)	90.1 (87.1-92.7)	85.6 (81.4-89.2)	89.2 (85.9-91.8)	84.7 (80.5-88.4)	89.5 (86.8-91.6)	86.2 (82.7-89.0)

Supplement Table 16. Age-standardised rates (per 100,000 people) and percentages of DALYs (with 95% uncertainty intervals) due to subarachnoid haemorrhage in 2019 attributable to metabolic, behavioural and environmental/occupational risk clusters and all risk factors combined in low-income and middle-income countries, high-income countries and globally by sex

Rates	Low-income countries		Low-middle income countries		Upper middle-income countries		High-income countries		Globally	
	Males	Females	Males	Females	Males	Females	Males	Females	Males	Females
Metabolic risks	88.9 (37.7-233.5)	71.1 (41.0-143.0)	110.6 (74.6-163.9)	100.0 (73.5-137.0)	100.5 (76.5-126.3)	81.3 (67.7-96.9)	70.0 (60.5-79.2)	75.3 (63.6-86.8)	96.6 (78.7-119.7)	85.5 (71.4-101.0)
Behavioural risks	58.2 (25.1-153.2)	40.0 (21.7-80.4)	81.0 (53.0-123.4)	54.0 (35.5-77.2)	83.2 (62.2-108.7)	50.5 (39.3-63.7)	51.4 (43.8-59.6)	54.4 (46.4-64.2)	74.5 (61.1-95.3)	51.9 (41.0-65.5)
Environmental and occupational risks	70.2 (30.6-188.9)	56.8 (34.3-114.0)	74.0 (49.2-113.8)	65.7 (47.1-89.6)	56.7 (40.7-72.3)	41.4 (34.8-48.9)	21.2 (17.4-25.4)	22.1 (18.0-26.5)	55.5 (44.0-71.7)	45.7 (37.5-55.2)
All risks combined	116.9 (51.7-313.5)	94.1 (56.7-188.0)	140.4 (97.1-210.7)	124.0 (93.3-166.0)	127.6 (98.3-157.3)	101.0 (88.3-116.0)	84.3 (77.1-92.0)	94.9 (85.8-105.0)	121.8 (103.8-152.0)	106.0 (93.0-122.0)
Percentages										
Metabolic risks	62.3 (52.5-70.1)	63.8 (55.4-71.4)	66.8 (58.1-74.0)	68.2 (60.1-75.4)	67.3 (58.5-74.8)	67.1 (58.5-74.3)	70.9 (62.8-77.9)	65.8 (57.2-73.2)	66.8 (58.7-73.9)	66.7 (58.4-73.9)
Behavioural risks	41.0 (33.1-50.2)	36.0 (27.0-46.1)	48.9 (41.7-56.7)	36.7 (28.1-45.8)	55.6 (48.8-62.6)	41.7 (34.1-49.8)	52.1 (45.3-59.0)	47.5 (41.2-54.5)	51.5 (44.8-58.5)	40.5 (33.1-48.4)
Environmental and occupational risks	49.2 (45.2-52.9)	51.0 (47.6-54.3)	44.5 (40.0-48.6)	44.5 (40.4-48.4)	37.8 (33.9-41.8)	34.1 (30.6-37.6)	21.5 (17.8-25.4)	19.3 (15.8-23.0)	38.3 (34.5-42.0)	35.6 (32.1-39.5)
All risks combined	81.9 (75.7-85.8)	84.4 (80.8-87.6)	84.7 (80.8-87.7)	84.5 (80.8-87.7)	85.4 (82.2-88.3)	83.1 (79.1-86.5)	85.5 (81.8-88.5)	82.9 (78.7-86.4)	84.2 (80.9-87.2)	83.1 (79.3-86.4)

Section 6. Supplementary figures

6.1 Supplement Figure 1. 21 GBD regions

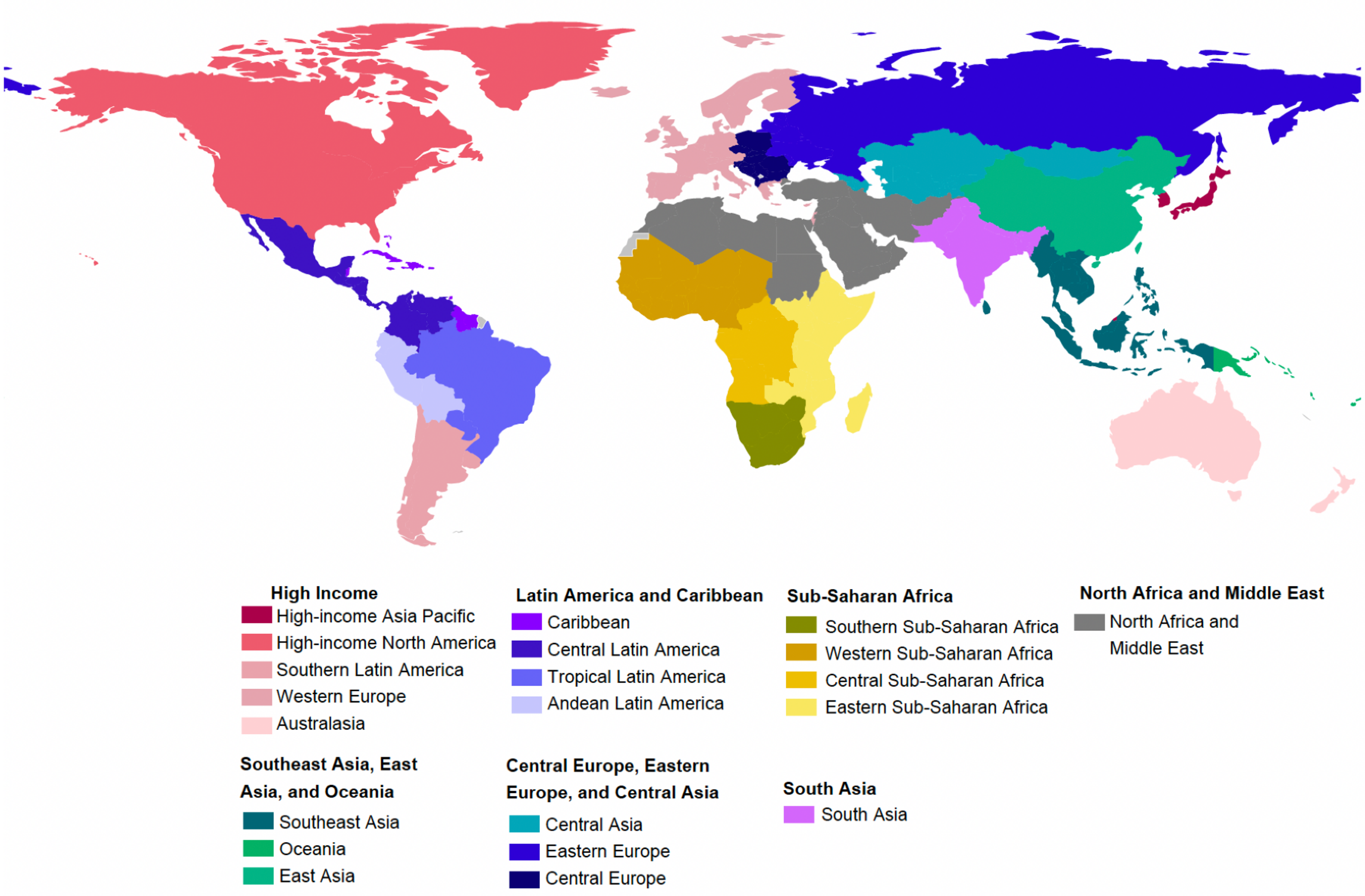

List of countries in 21 GBD regions

ASIA PACIFIC, HIGH INCOME: Brunei, Japan, Republic of Korea, Singapore; **ASIA CENTRAL:** Armenia, Azerbaijan, Georgia, Kazakhstan, Kyrgyzstan, Mongolia**,** Tajikistan, Turkmenistan, Uzbekistan; **ASIA, SOUTHEAST:** Cambodia, Indonesia**,** Lao People's Democratic Republic, Malaysia, Maldives**,** Mauritius, Mayotte**,** Myanmar**,** Philippines**,** Seychelles**,** Sri Lanka**,** Thailand, Timore Leste**,** Viet Nam; **ASIA EAST:** China, Democratic People's Republic of Korea, Hong Kong, Taiwan; **ASIA SOUTH:** Afghanistan, Bangladesh, Bhutan**,** India**,** Nepal**,** Pakistan; **AUSTRALASIA:** Australia, New Zealand; **CARIBBEAN:** Anguilla, Antigua and Barbuda, Aruba, Bahamas, Barbados, Belize, Bermuda, British Virgin Islands, Cayman Islands, Cuba, Dominica, Dominican Republic, French Guiana, Grenada, Guadeloupe, Guyana, Haiti, Jamaica, Martinique, Montserrat, Netherlands Antilles, Saint Kitts and Nevis, St. Lucia, St. Vincent, Suriname, Trinidad and Tobago, Turks and Caicos Islands; **EUROPE, CENTRAL:** Albania, Bosnia and Herzegovina, Bulgaria, Croatia, Czech Republic, Hungary, Poland, Romania, Serbia and Montenegro, Slovakia, Slovenia, The Former Yugoslav Republic of Macedonia; **EUROPE, EASTERN:** Belarus, Estonia, Latvia, Lithuania, Republic of Moldova, Russian Federation, Ukraine; **EUROPE, WESTERN:** Andorra, Austria, Belgium, Channel Islands, Cyprus, Denmark, Faeroe Islands, Finland, France, Germany, Gibraltar, Greece, Greenland, Holy See, Iceland, Ireland, Isle of Man, Israel, Italy, Liechtenstein, Luxembourg, Malta, Monaco, Netherlands, Norway, Portugal, Saint Pierre et Miquelon, San Marino, Spain, Sweden, Switzerland, United Kingdom; **LATIN AMERICA, ANDEAN:** Bolivia, Ecuador, Peru; **LATIN AMERICA, CENTRAL:** Colombia, Costa Rica, El Salvador, Guatemala, Honduras, Mexico, Nicaragua, Panama, Venezuela; **LATIN AMERICA, SOUTHERN:** Argentina, Chile, Falkland Islands (Malvinas), Uruguay; **LATIN AMERICA, TROPICAL:** Brazil, Paraguay; **NORTH AFRICA / MIDDLE EAST:** Algeria, Bahrain, Egypt, Iran (Islamic Republic of), Iraq, Jordan, Kuwait, Lebanon, Libyan Arab Jamahiriya, Morocco, Occupied Palestinian Territory, Oman, Qatar, Saudi Arabia, Syrian Arab Republic, Tunisia, Turkey, United Arab Emirates, Western Sahara, Yemen; **NORTH AMERICA, HIGH INCOME:** Canada, United States of America; **OCEANIA:** American Samoa, Cook Islands, Fiji, French Polynesia, Guam, Kiribati, Marshall Islands, Micronesia (Federated States of), Nauru, New Caledonia, Niue, Northern Mariana Islands, Palau, Papua New Guinea, Pitcairn, Samoa, Solomon Islands, Tokelau, Tonga, Tuvalu, Vanuatu, Wallis and Futuna Islands; **SUB-SAHARAN AFRICA, CENTRAL:** Angola, Central African Republic, Congo, Democratic Republic of the Congo, Equatorial Guinea, Gabon; **SUB-SAHARAN AFRICA, EAST:** Burundi, Comoros, Djibouti, Eritrea, Ethiopia, Kenya, Madagascar, Malawi, Mozambique, Rwanda, Somalia, Sudan, Uganda, United Republic of Tanzania, Zambia; **SUB-SAHARAN AFRICA, SOUTHERN:** Botswana, Lesotho, Namibia, South Africa, Swaziland, Zimbabwe; **SUB-SAHARAN AFRICA, WEST:** Benin, Burkina Faso, Cameroon, Cape Verde, Chad, Cote d'Ivoire, Gambia, Ghana, Guinea, Guinea-Bissau, Liberia, Mali, Mauritania, Niger, Nigeria, Saint Helena, Sao Tome and Principe, Senegal, Sierra Leone, Togo

6.2 Supplement Figure 2. Global number of deaths and DALYs by causes in 2019, both sexes, all ages

Global number of deaths by causes in 2019, both sexes, all ages

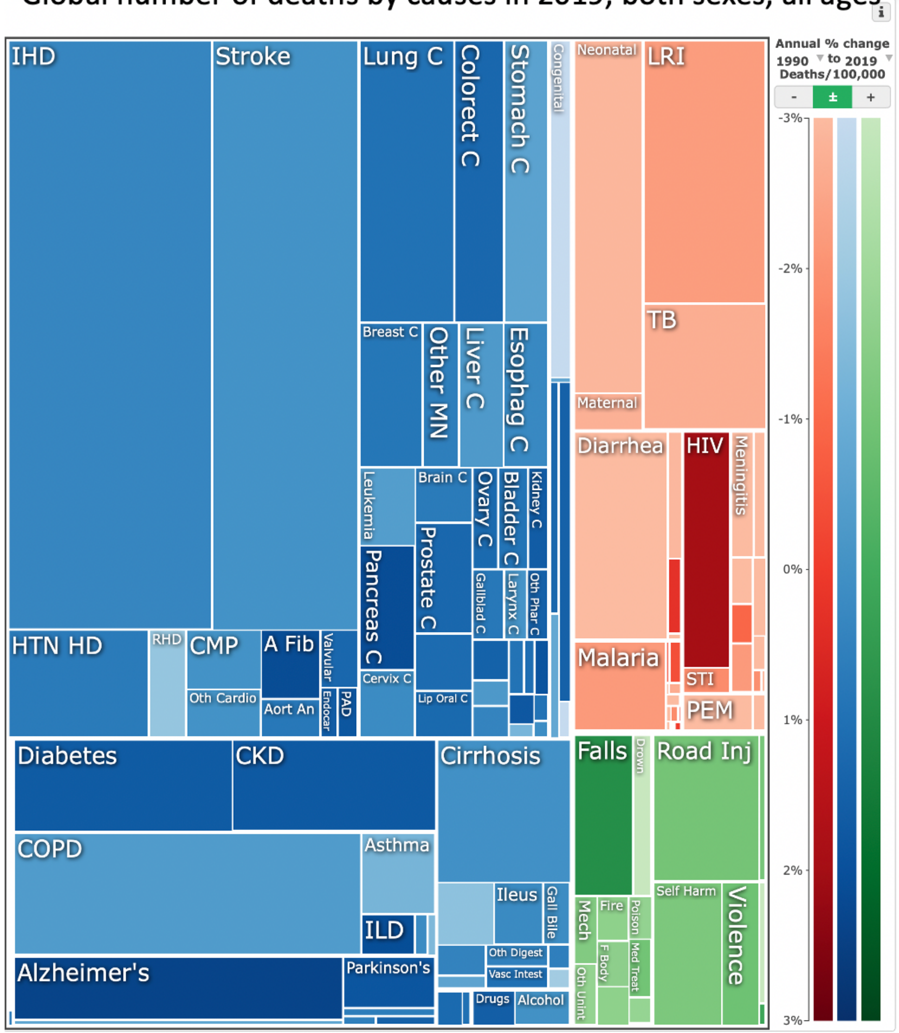

Global number of DALYs by causes in 2019, both sexes, all ages

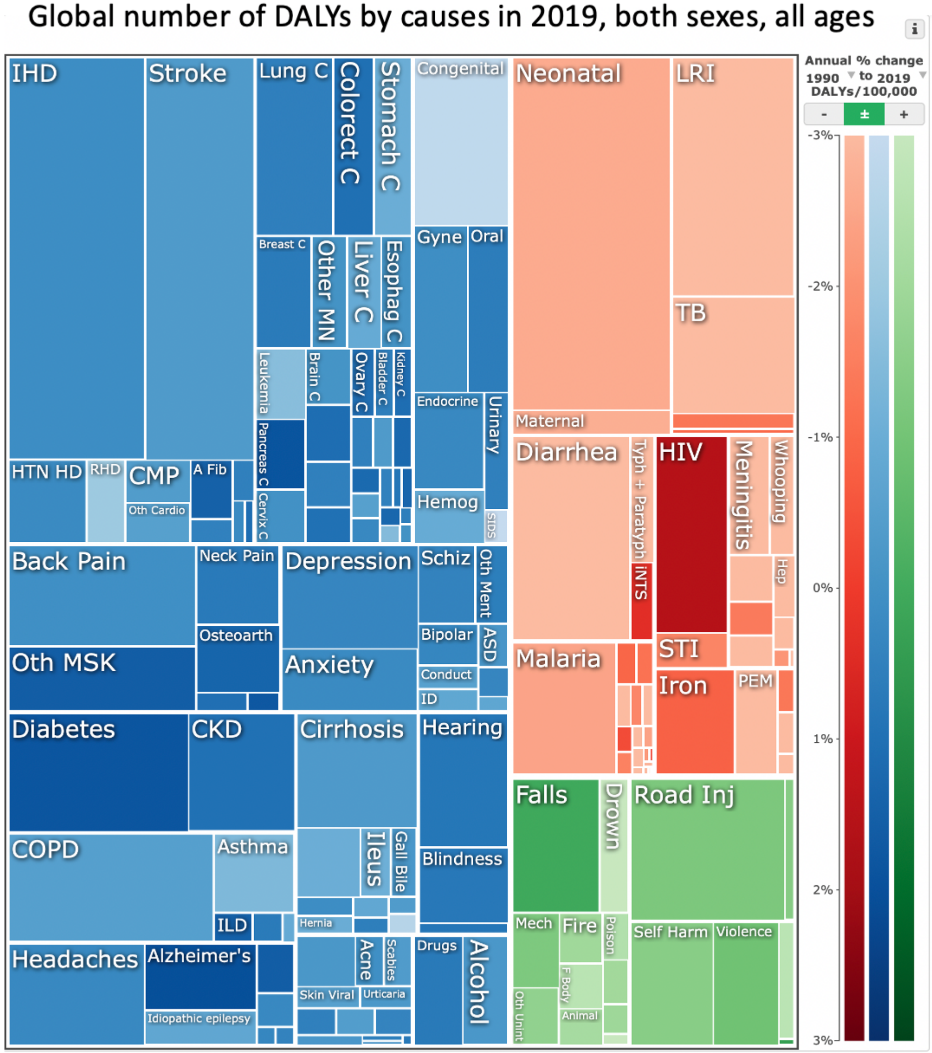

6.3 Supplement Figure 3. Absolute number (in millions) incident and prevalent strokes, deaths from stroke and DALYs due to stroke (shadow areas are 95% uncertainty intervals) by year from 1990 to 2019

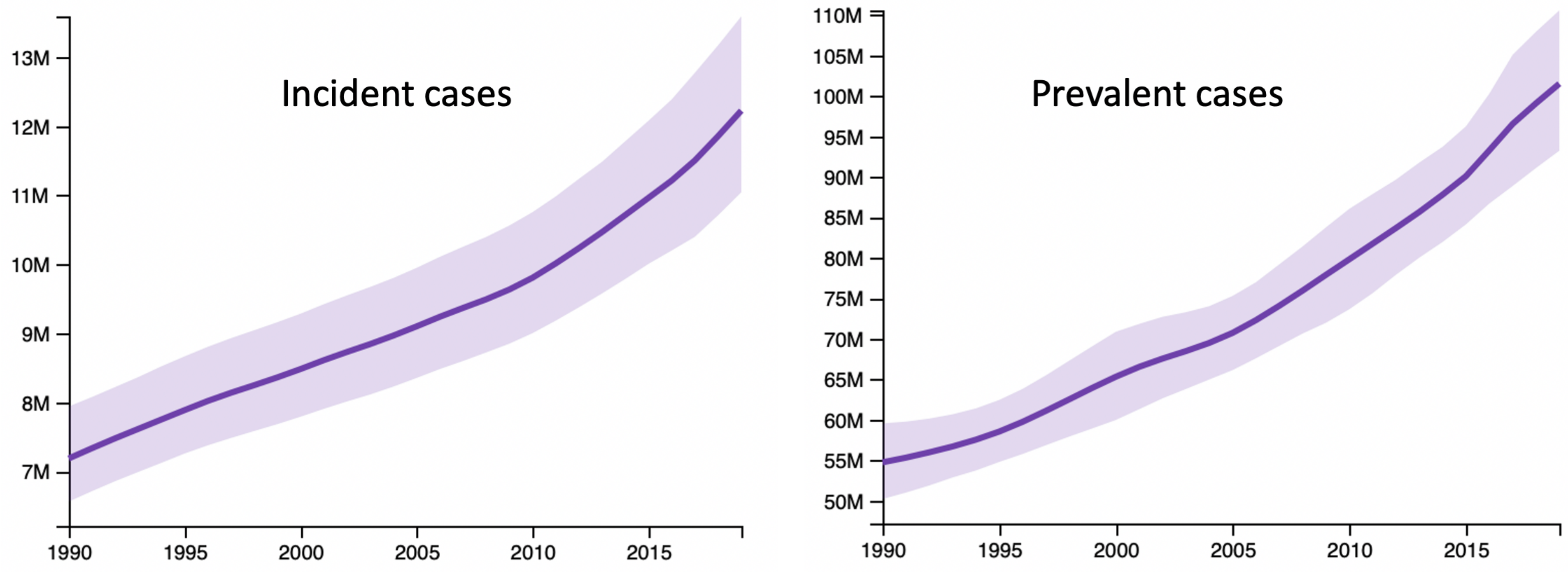

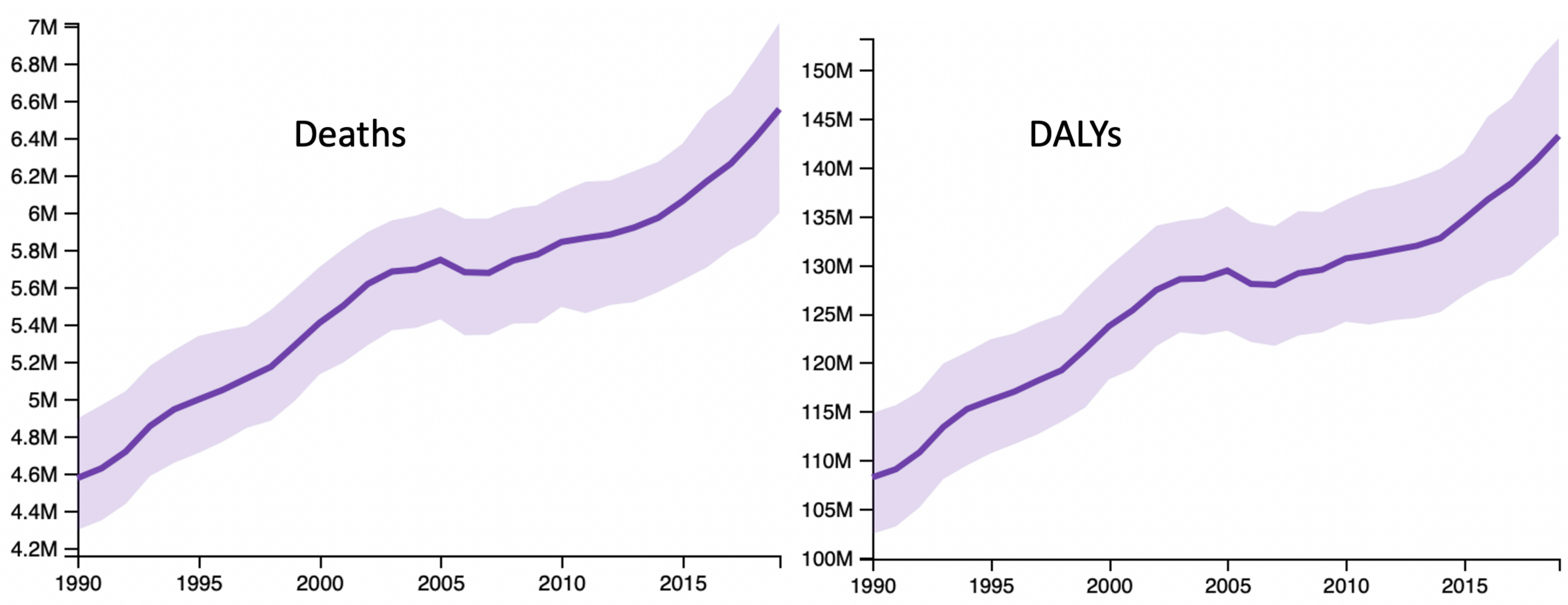
Deaths
7M
6.8M
6.6M
6.4M
6.2M
6M
5.8M
5.6M
5.4M
5.2M
5M
4.8M
4.6M
4.4M
4.2M
1990
1995
2000
2005
2010
2015
DALYs
150M
145M
140M
135M
130M
125M
120M
115M
110M
105M
100M
1990
1995
2000
2005
2010
2015

6.4 Supplement Figure 4. Age-standardized stroke death rates per 100,000 people in the world in 2019

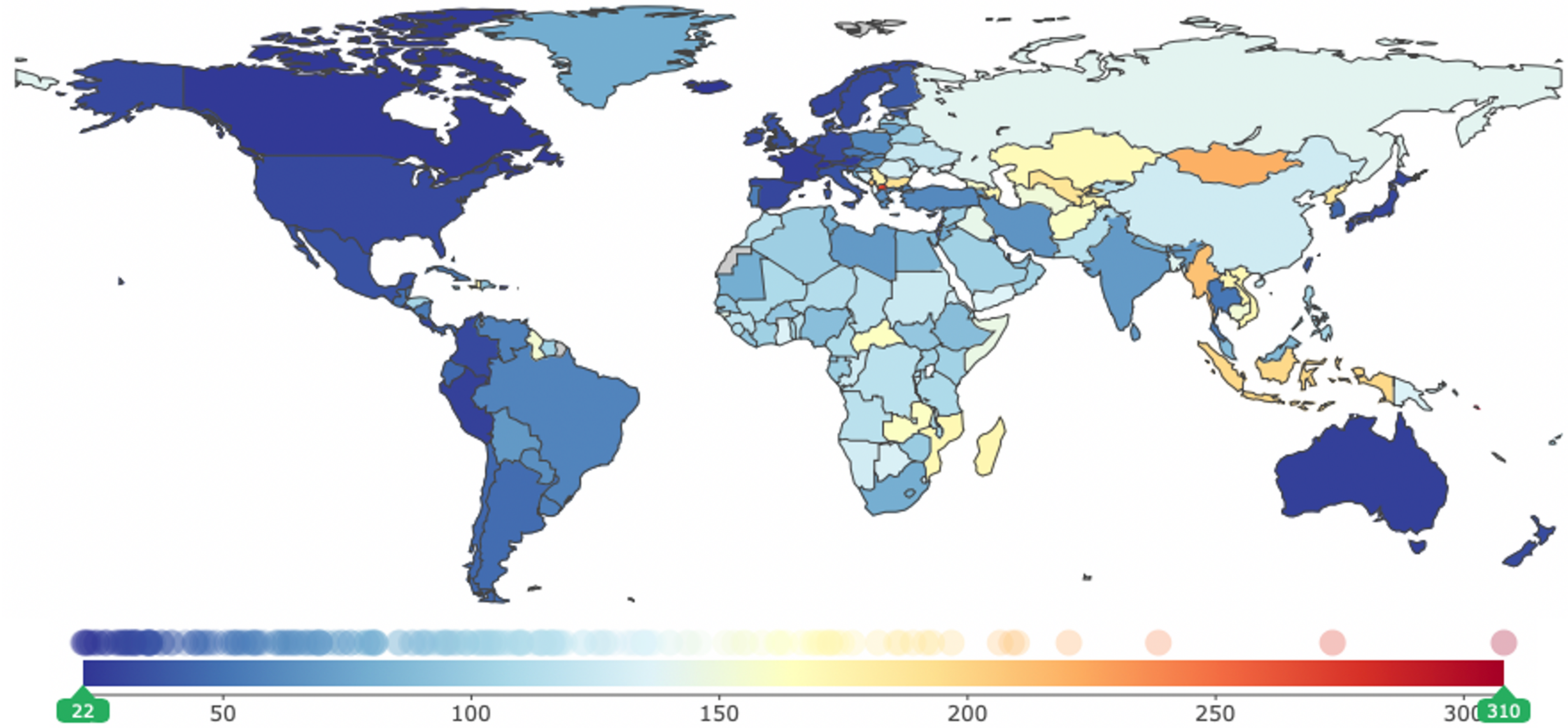

6.5 Supplement Figure 5. Age-standardized stroke prevalence per 100,000 people in the world in 2019

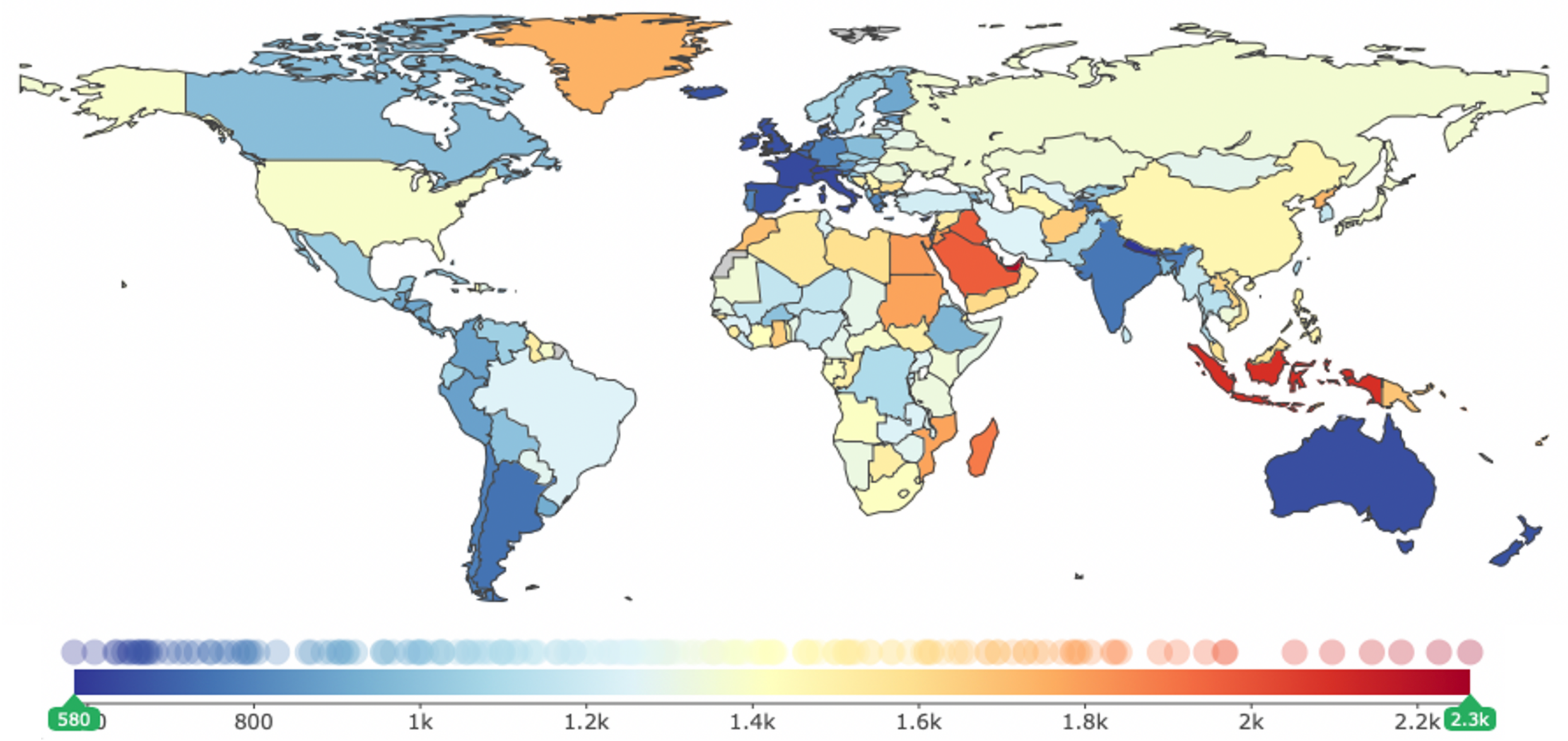

6.6 Supplement Figure 6. Age-standardized stroke DALYs rates per 100,000 people in the world in 2019

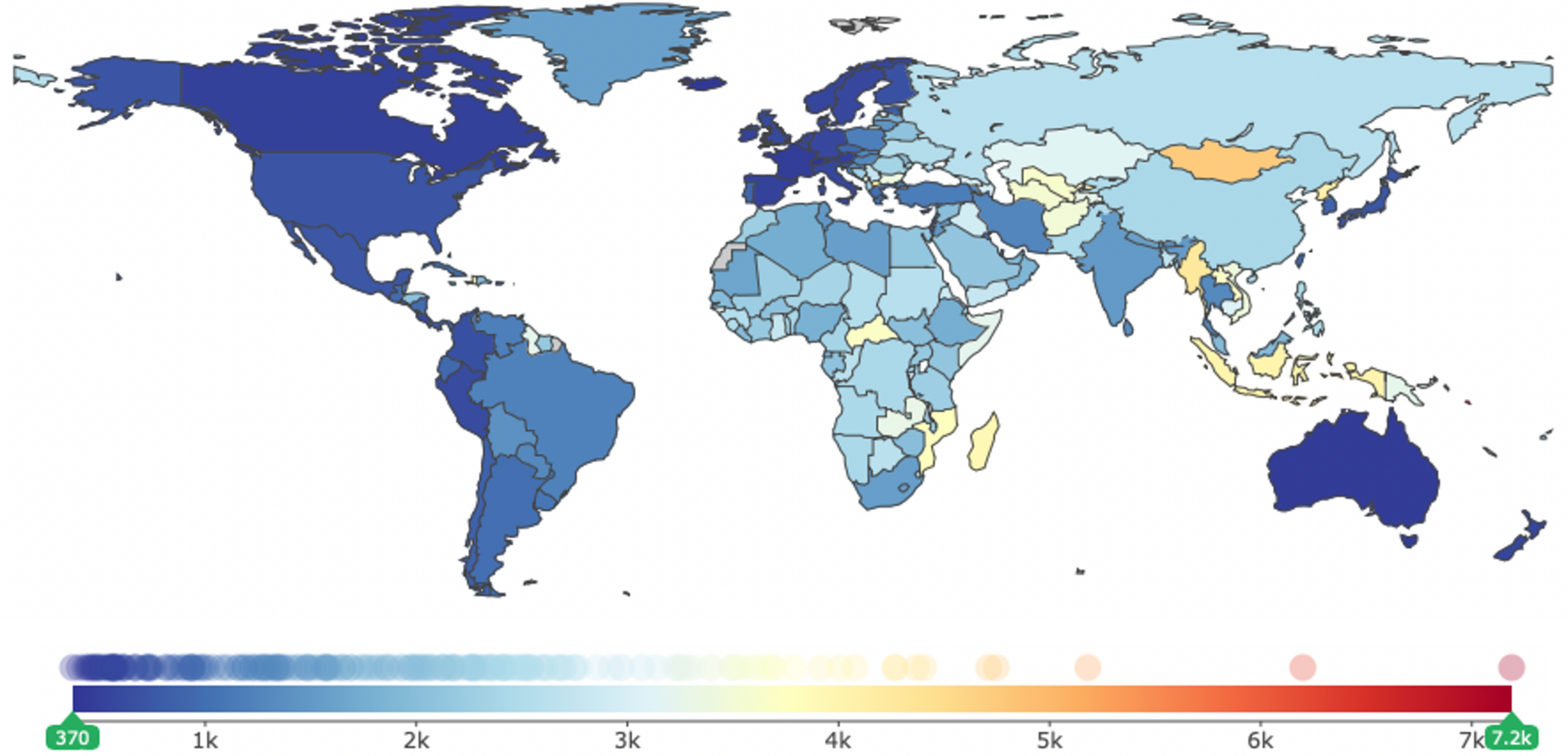

6.7 Supplement Figure 7. Annual stroke incidence rate and prevalence per 100,000 people aged <70 years and people aged 55-89 years (shadow areas are 95% uncertainty intervals)

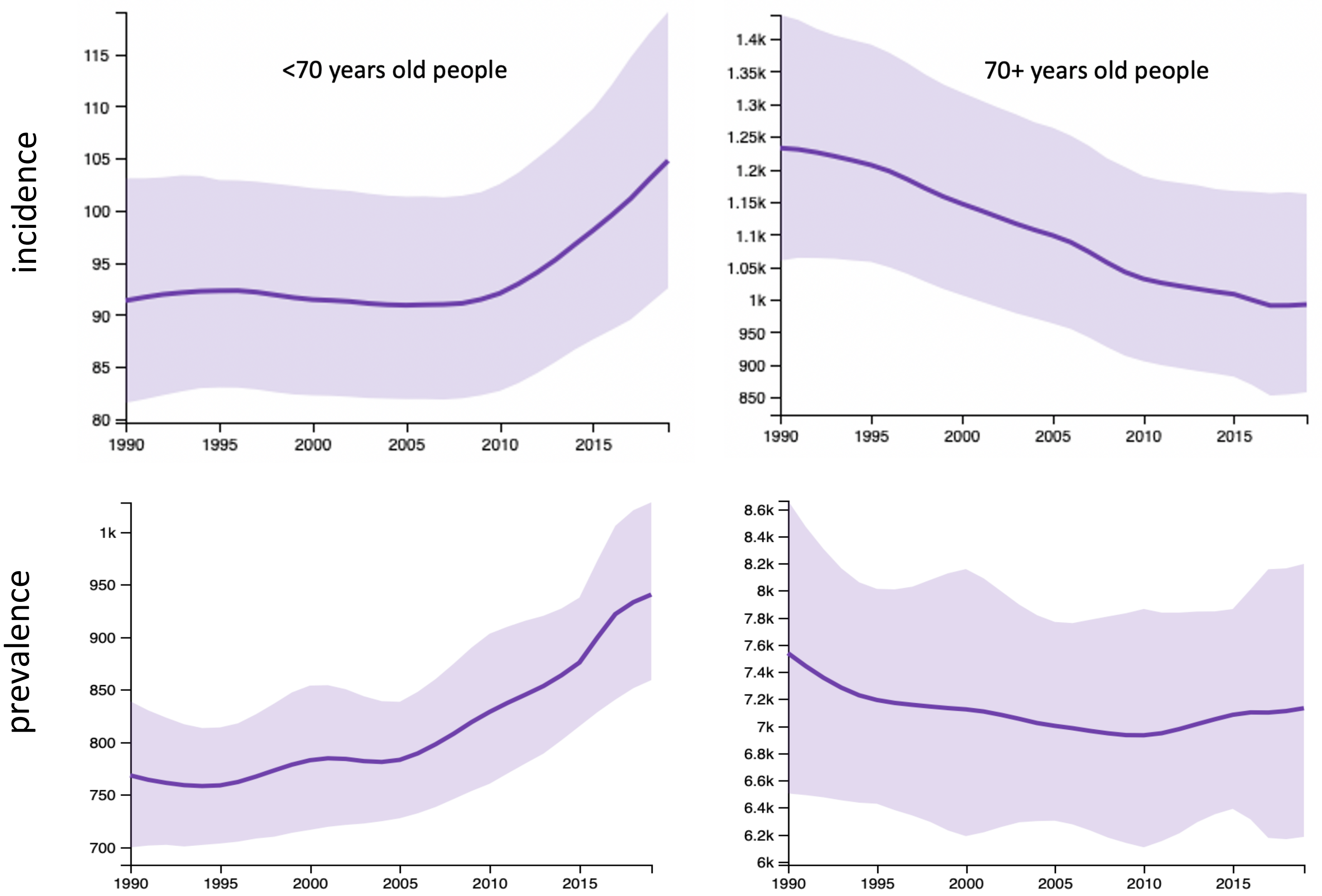

6.8 Supplement Figure 8. Annual age-standardised incidence rates of ischaemic stroke (IS), intracerebral haemorrhage (ICH) and subarachnoid haemorrhage (SAH) per 100,000 people from 1990 to 2019 (shadow areas are 95% uncertainty intervals)

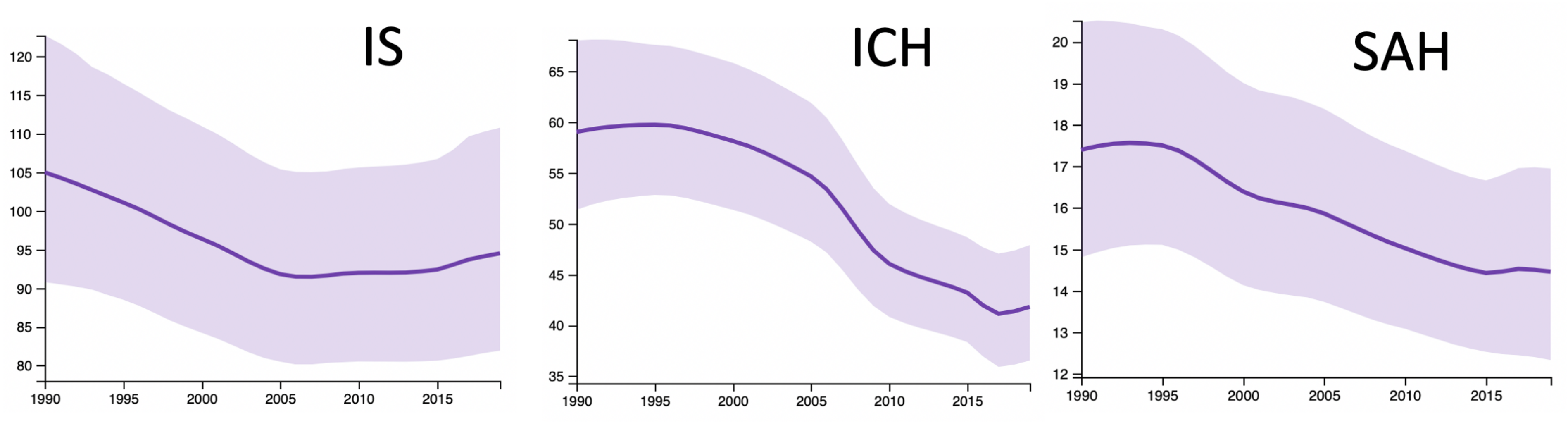

6.9 Supplement Figure 9. Annual age-standardised incidence rates of ischaemic stroke (IS), intracerebral haemorrhage (ICH) and subarachnoid haemorrhage (SAH) per 100,000 people in high-income countries (HIC) and low- to middle-income countries (LMIC) from 1990 to 2019 by sex (shadow areas are 95% uncertainty intervals)

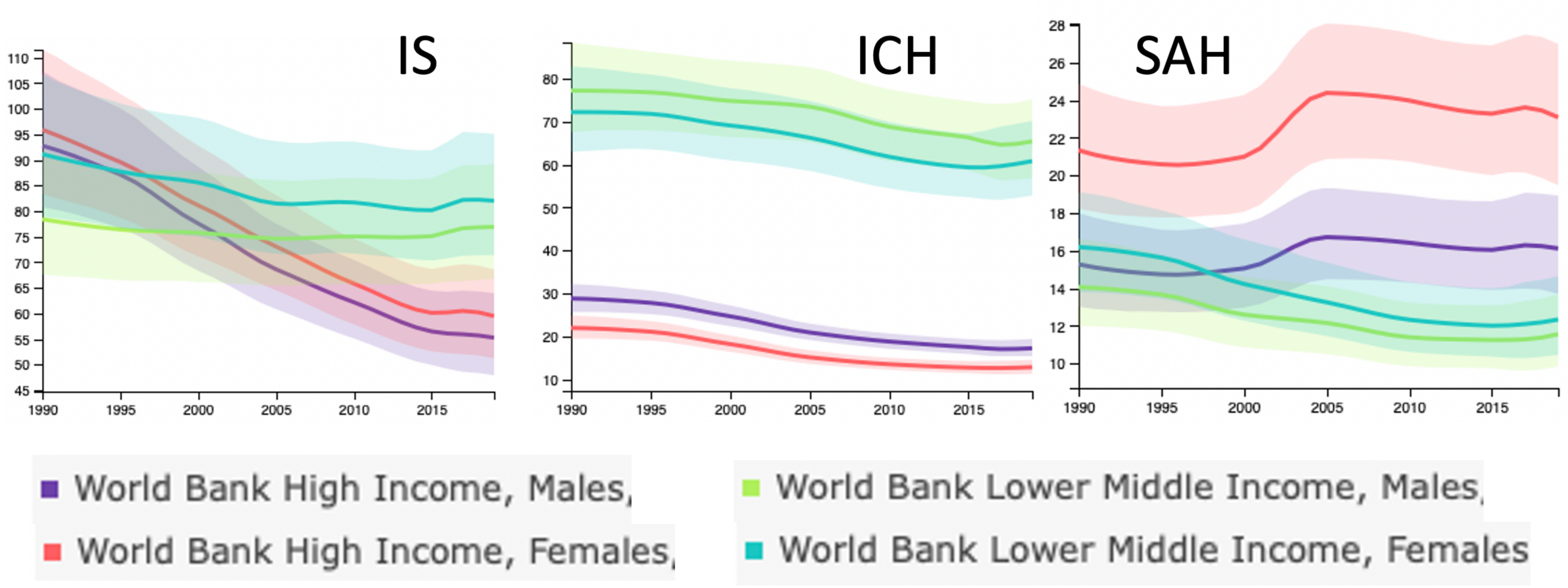

6.10 Supplement Figure 10. Annual age-standardised incidence rates of ischaemic stroke (IS), intracerebral haemorrhage (ICH) and subarachnoid haemorrhage (SAH) per 100,000 people in males and females from 1990 to 2019 (shadow areas are 95% uncertainty intervals)

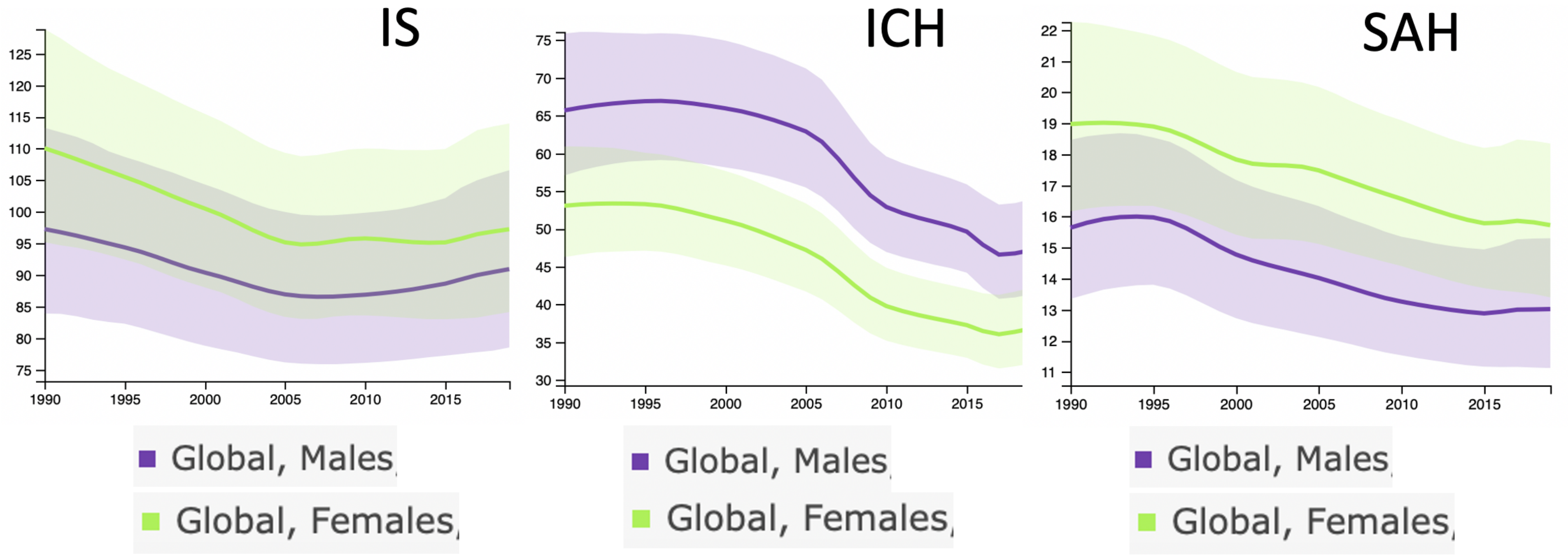

6.11 Supplement Figure 11. Age-standardised stroke prevalence, death and DALYs rates per 100,000 people by stroke type and country, for both sexes, 2019

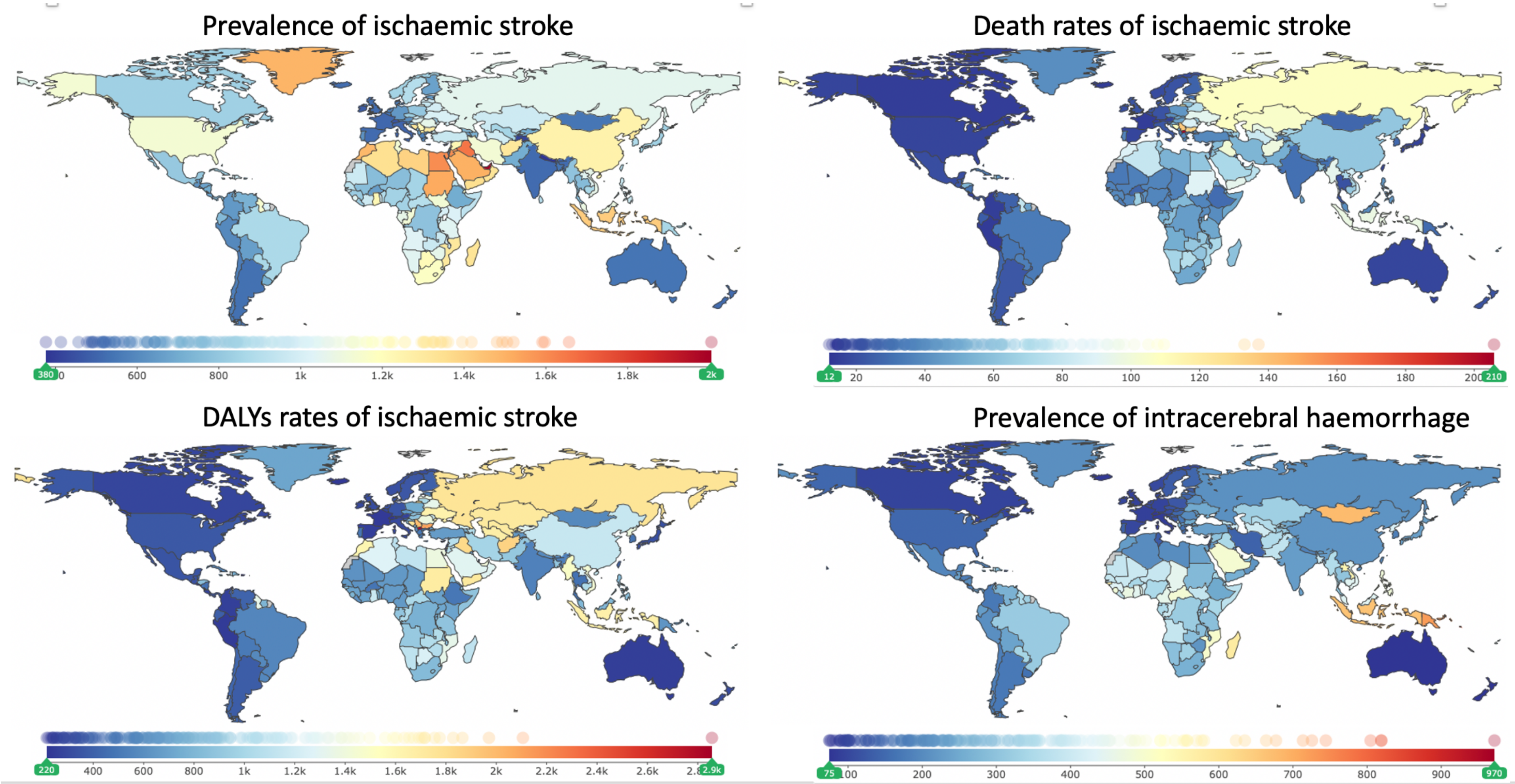

Death rates of intracerebral haemorrhage

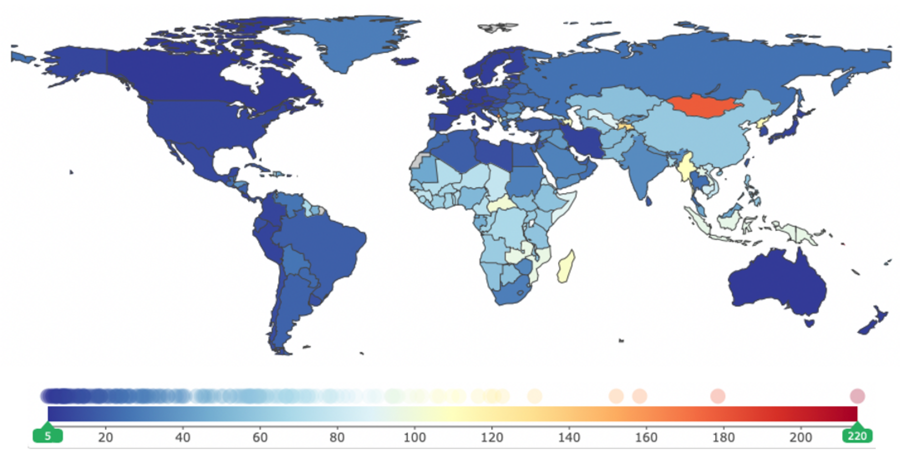

DALYs rates of intracerebral haemorrhage

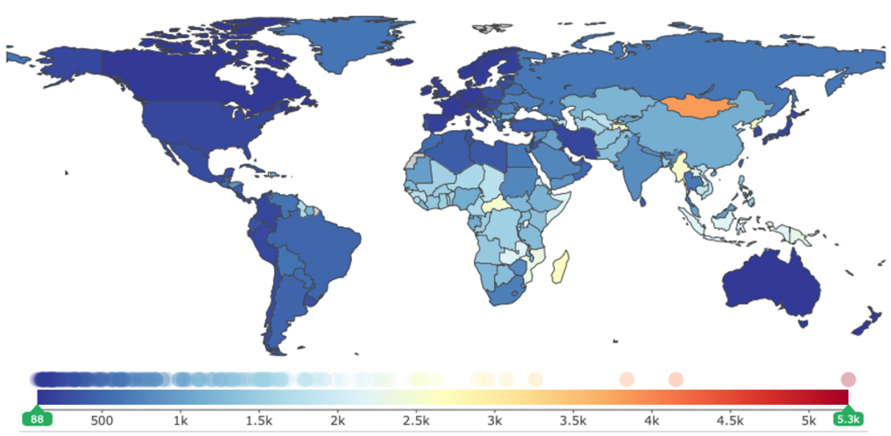

Prevalence of subarachnoid haemorrhage

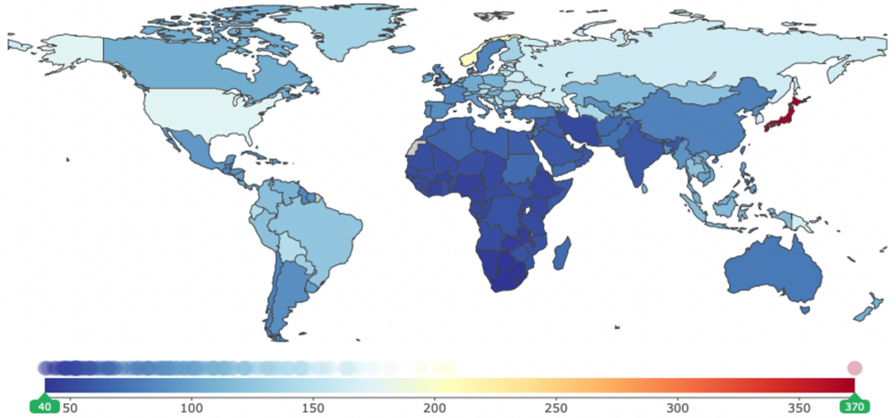

Death rates of subarachnoid haemorrhage

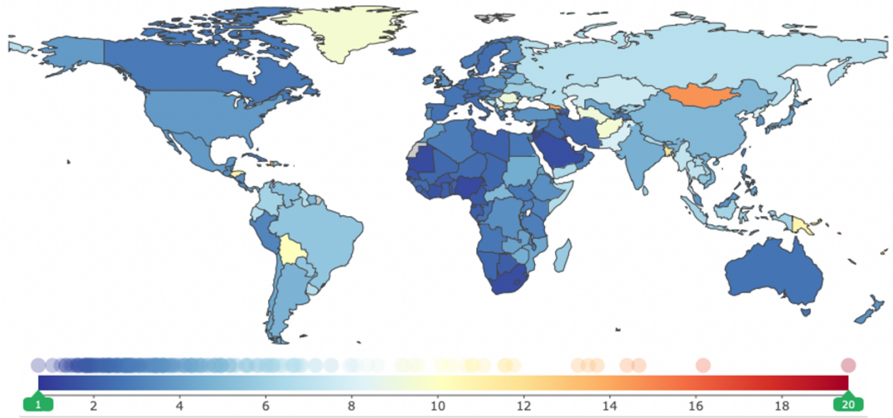

DALYs rates of subarachnoid haemorrhage

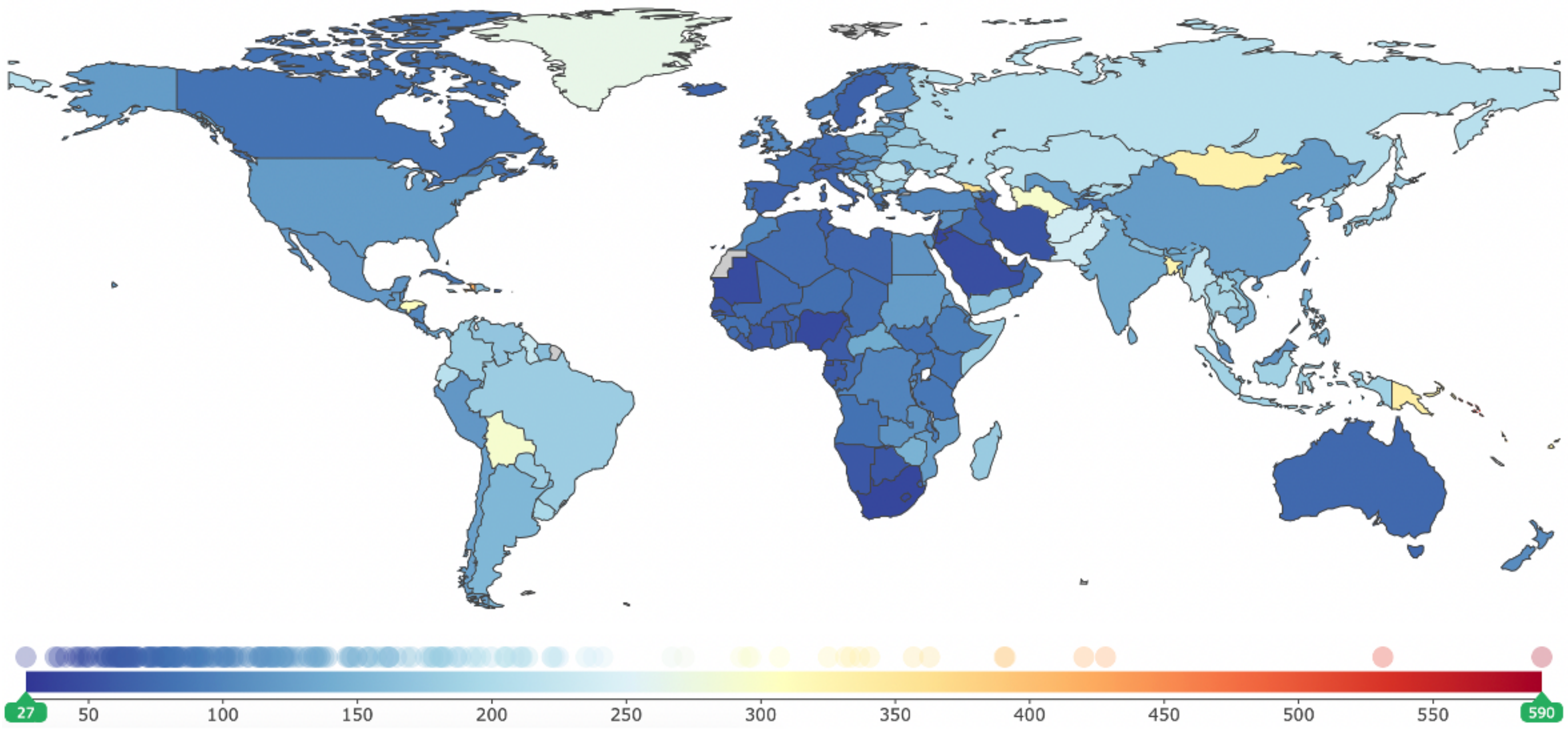

6.12 Supplement Figure 12. Age-standardised population-attributable fraction of DALYs of ischaemic stroke due to the three risk clusters and five main risk factors, for both sexes, 2019

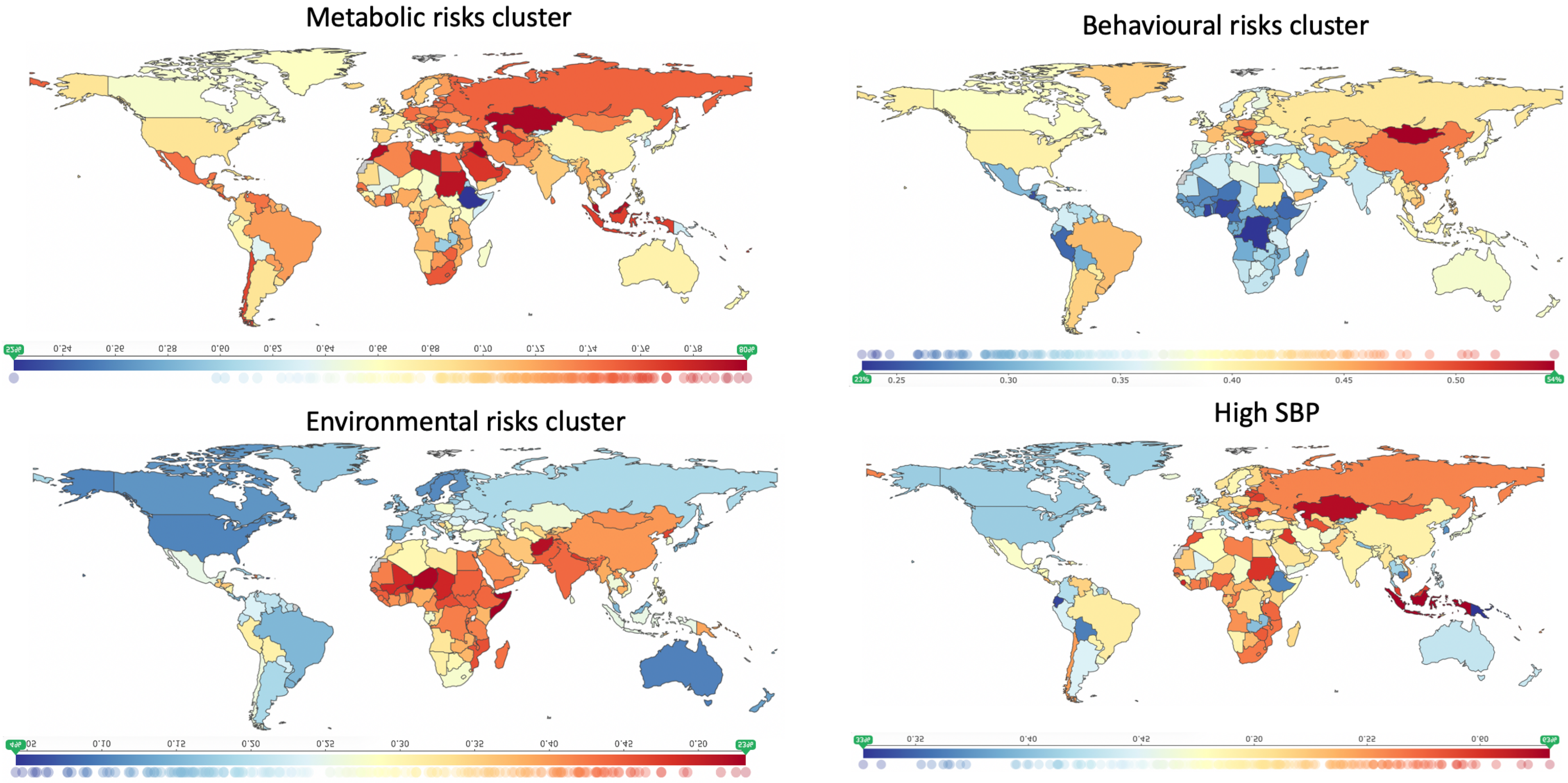

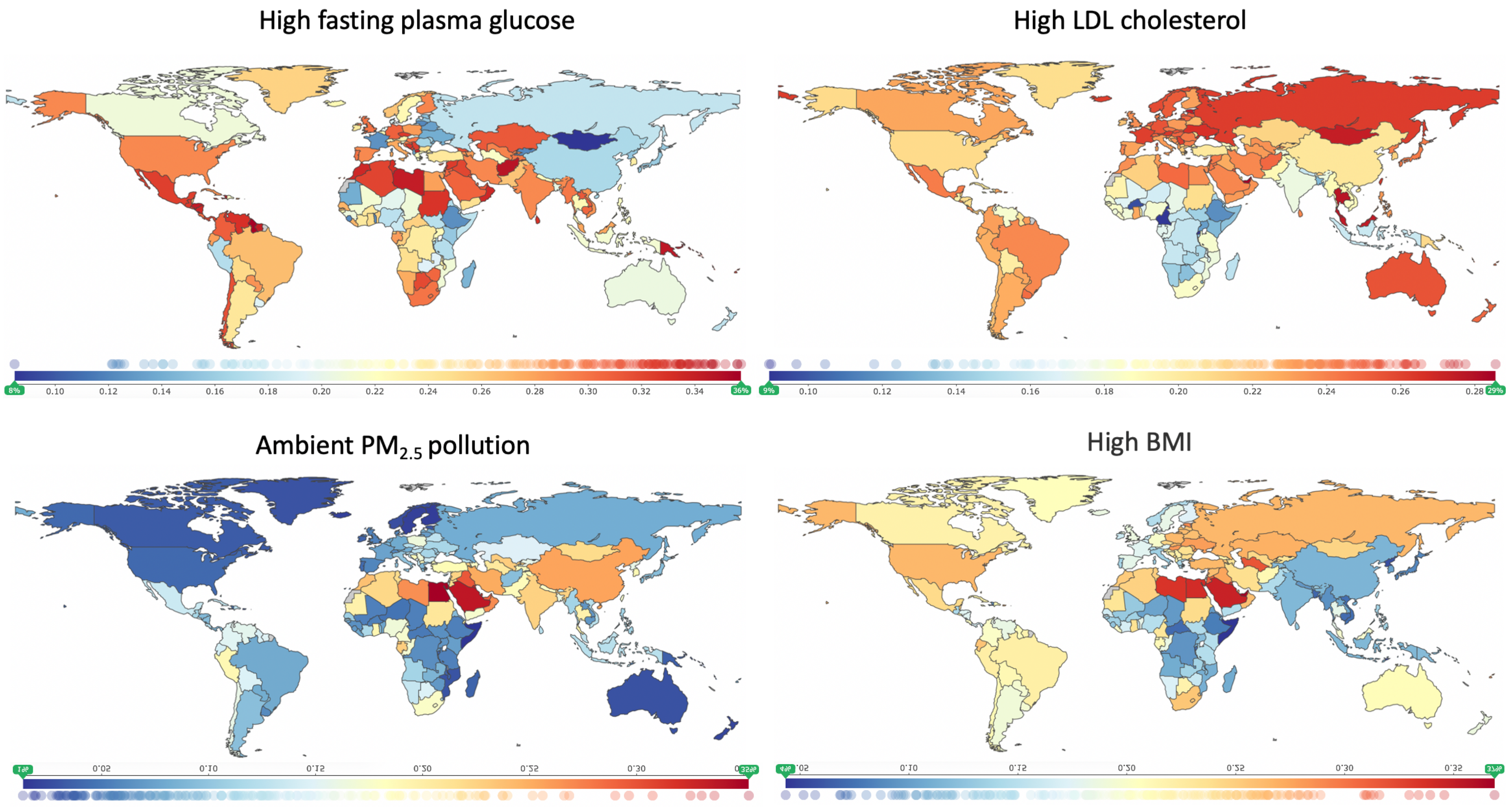
High fasting plasma glucose
8%
0.10
0.12
0.14
0.16
0.18
0.20
0.22
0.24
0.26
0.28
0.30
0.32
0.34
36%
High LDL cholesterol
9%
0.10
0.12
0.14
0.16
0.18
0.20
0.22
0.24
0.26
0.28
29%
Ambient PM2.5 pollution
High BMI

6.13. Supplement Figure 13. Age-standardised population-attributable fraction of DALYs of intracerebral haemorrhage due to the three risk clusters and five main risk factors, for both sexes, 2019

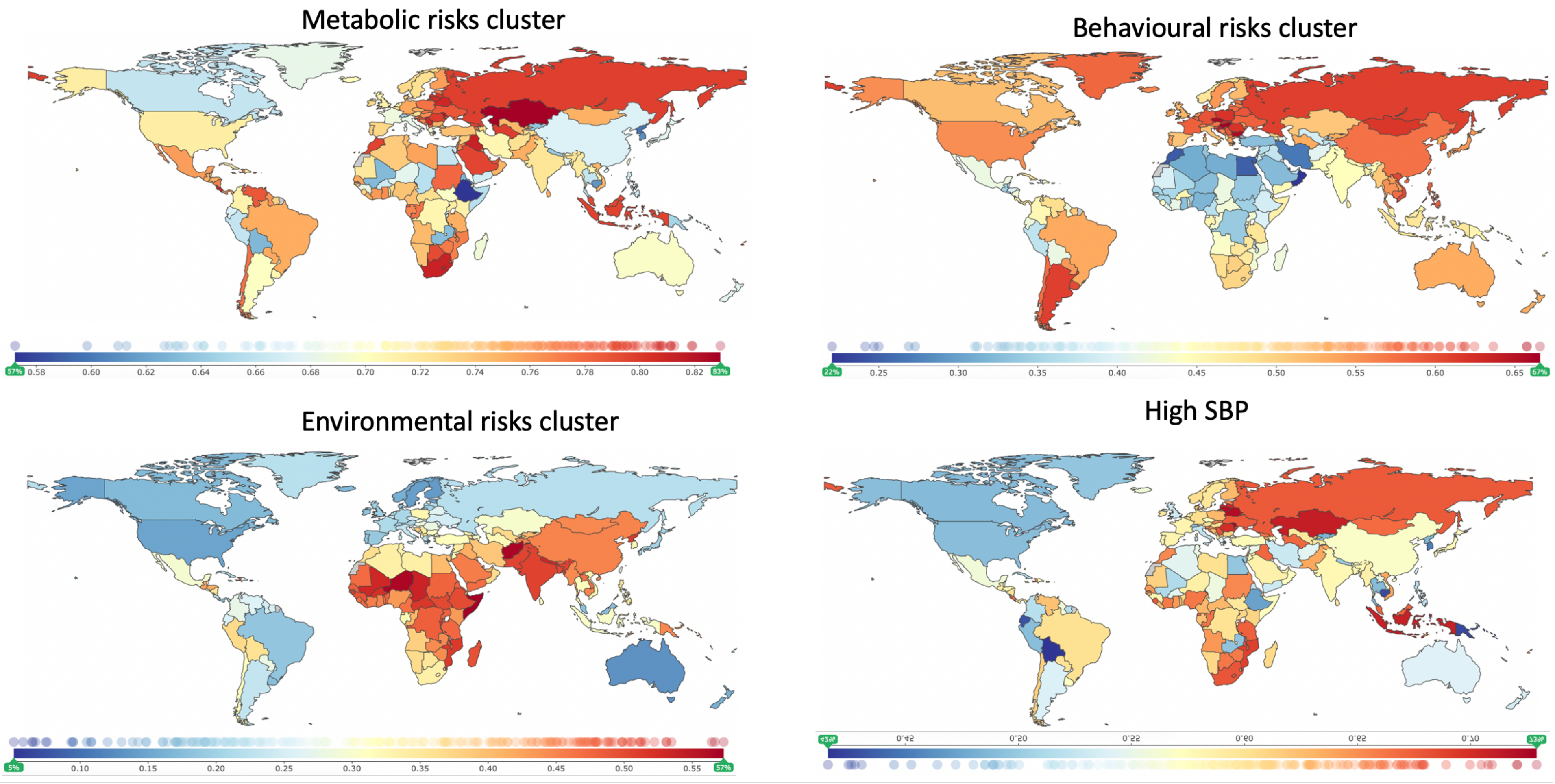

High BMI

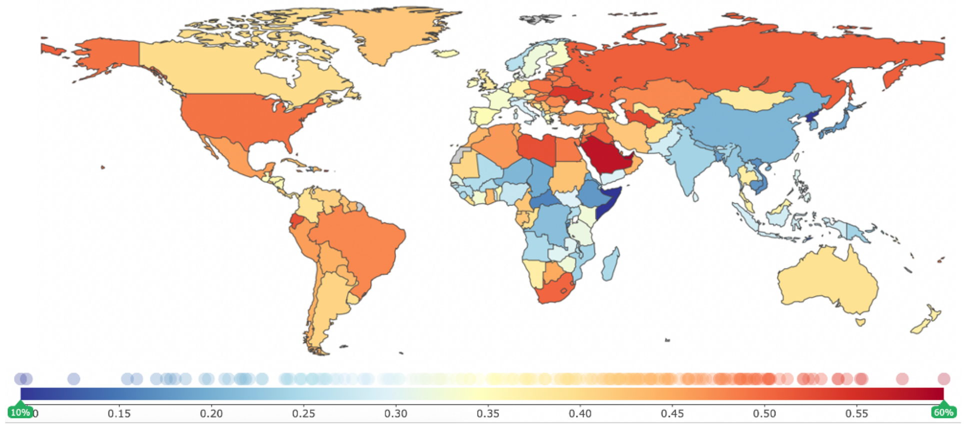

Ambient $PM_{2.5}$ pollution

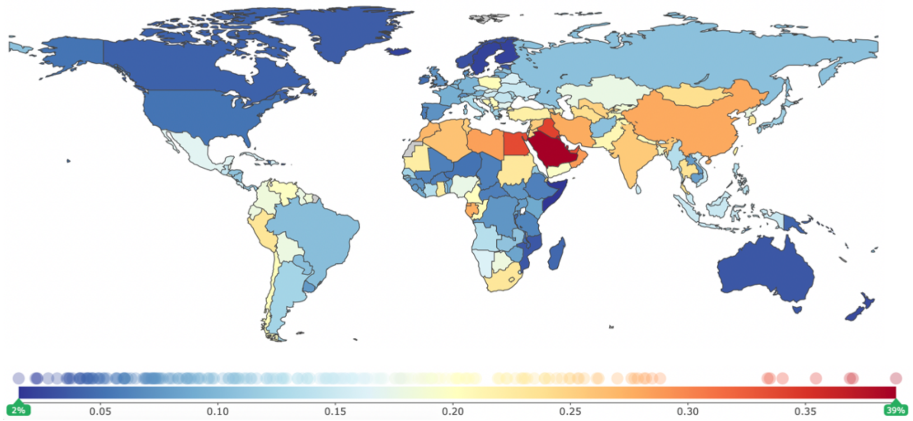

Smoking

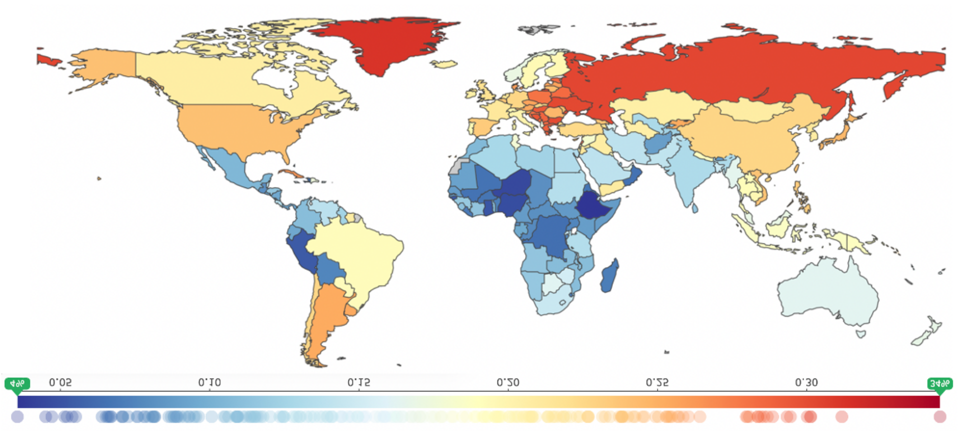

High fasting plasma glucose

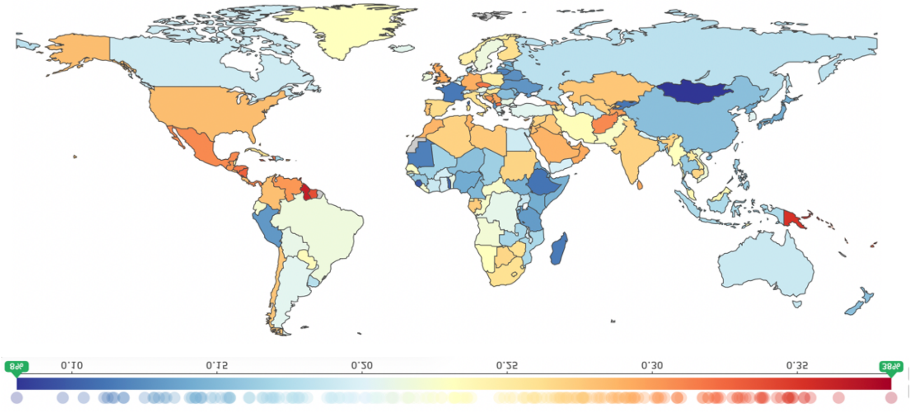

6.14. Supplement Figure 14. Age-standardised population-attributable fraction of DALYs of subarachnoid haemorrhage due to the three risk clusters and five main risk factors, for both sexes, 2019

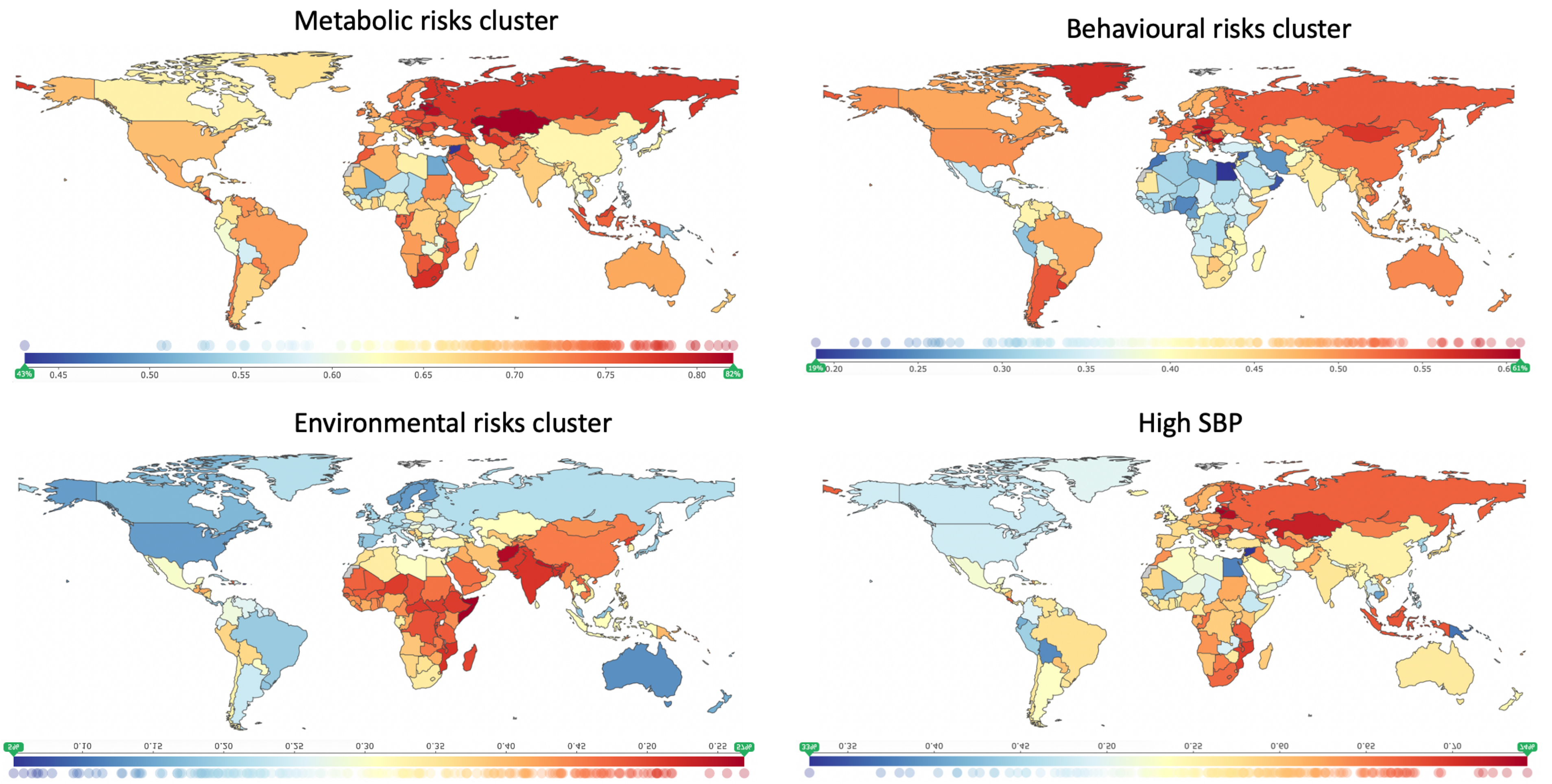

High BMI

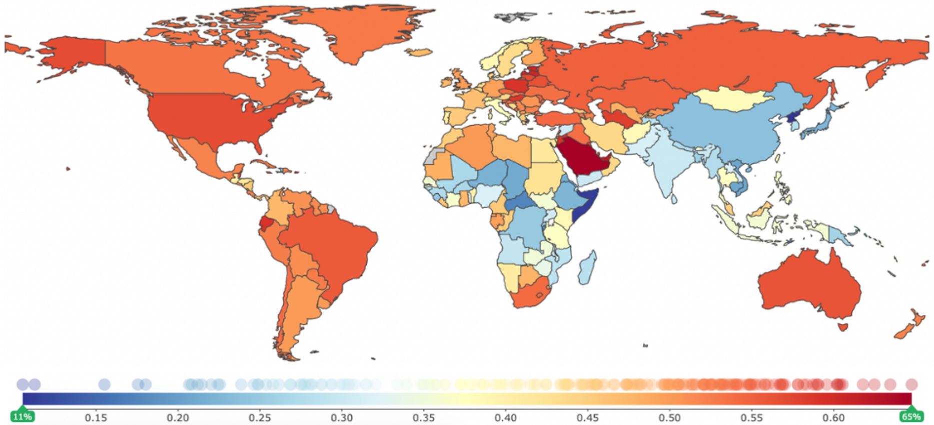

Ambient $PM_{2.5}$ pollution

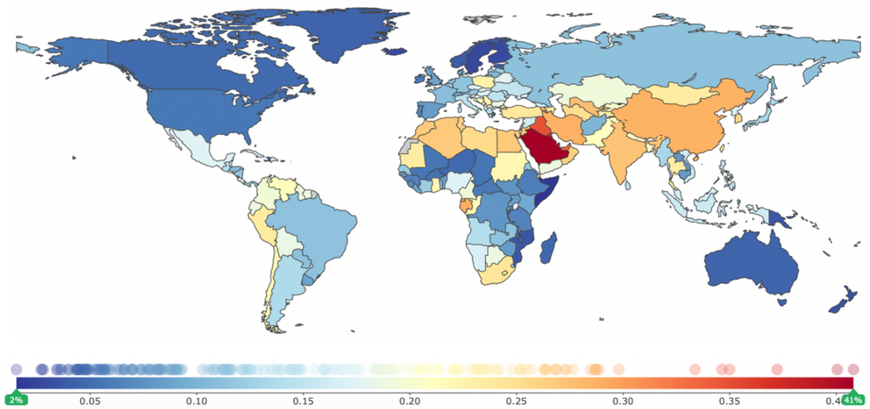

Smoking

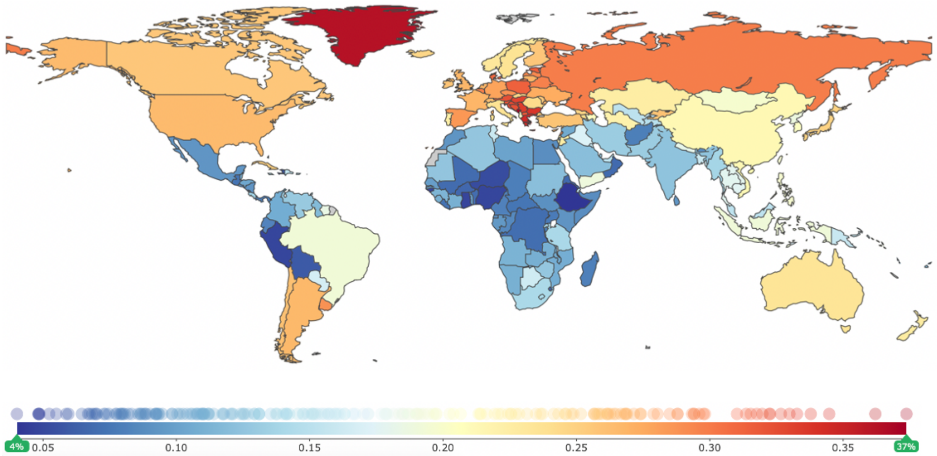

High fasting plasma glucose

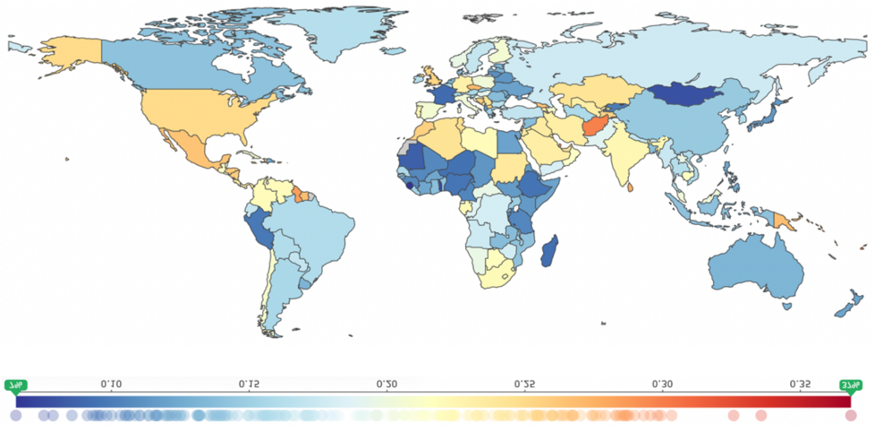

6.15 Supplement Figure 15. Global stroke related DALYs attributable to risk factors in 1990 and 2019 (rank based on total number of all ages DALYs)

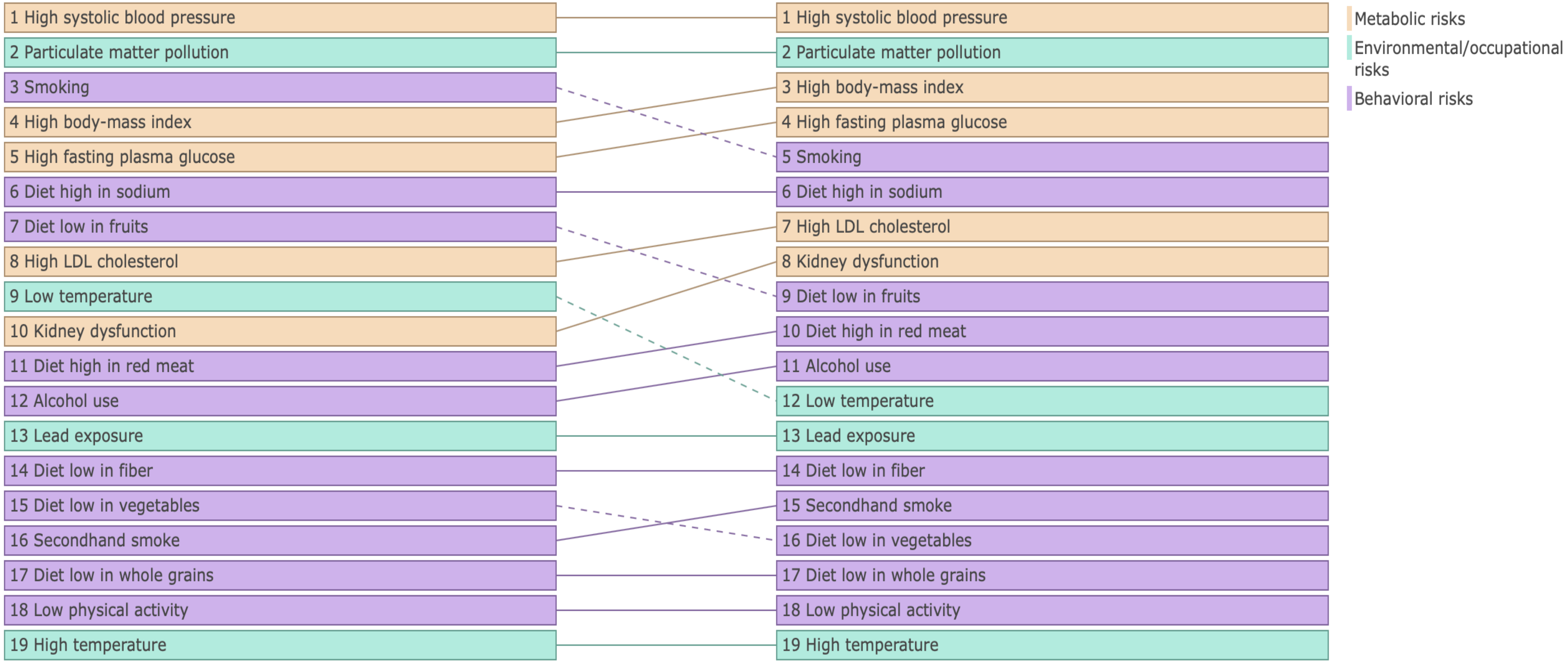

6.16 Supplement Figure 16. Global ischaemic stroke related DALYs attributable to risk factors in 1990 and 2019 (rank based on total number of all ages DALYs)

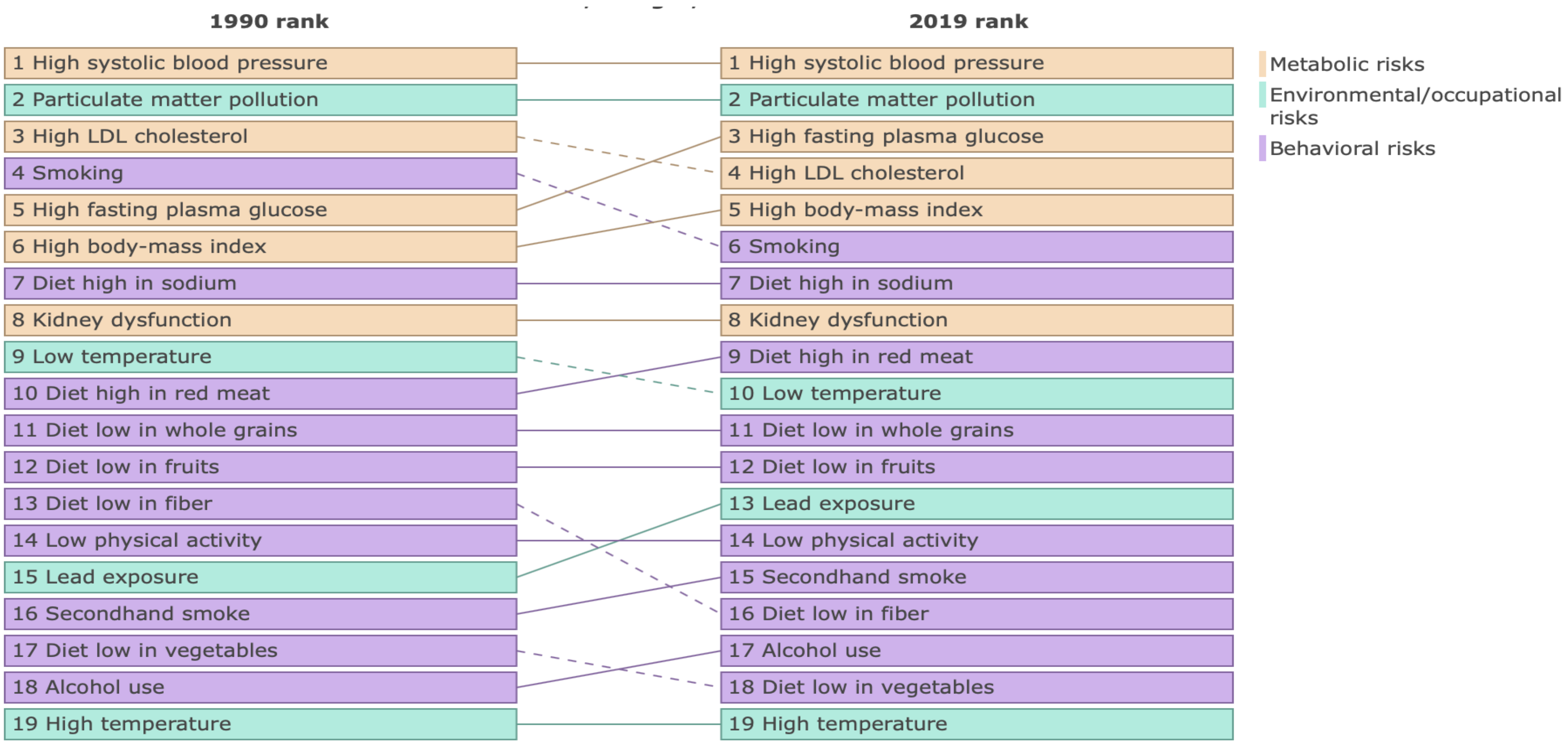

6.17 Supplement Figure 17. Global intracerebral haemorrhage related DALYs attributable to risk factors in 1990 and 2019 (rank based on total number of all ages DALYs)

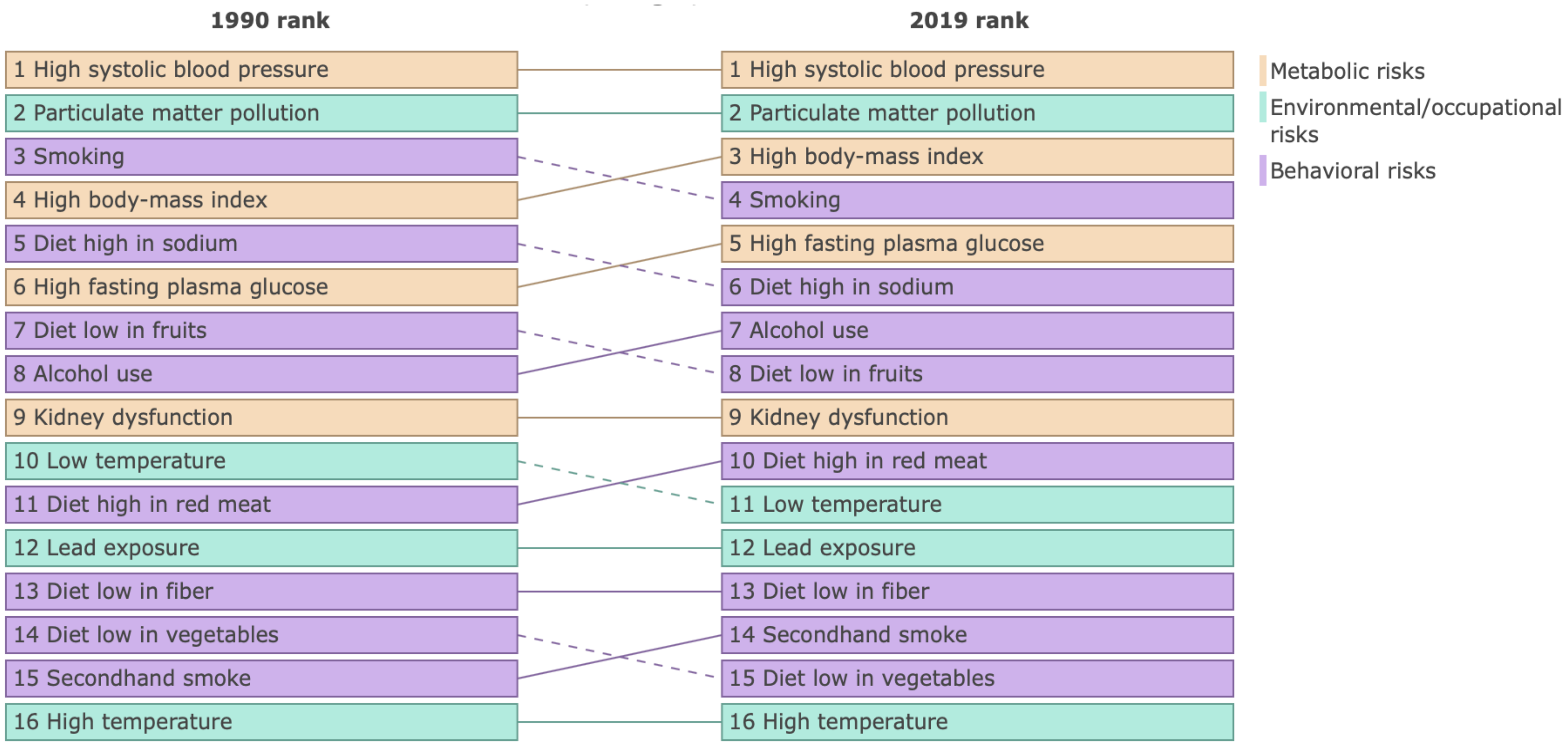

6.18 Supplement Figure 18. Global subarachnoid haemorrhage related DALYs attributable to risk factors in 1990 and 2019 (rank based on total number of all ages DALYs)

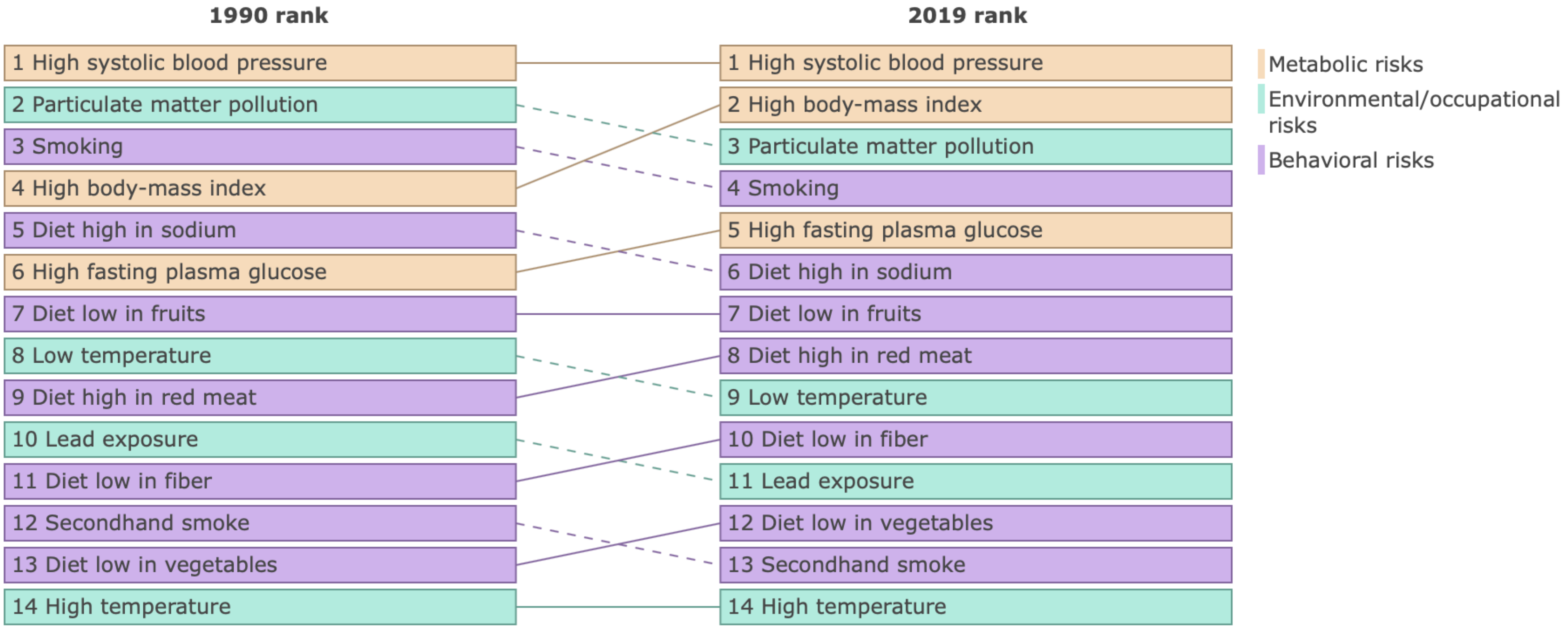

6.19 Supplement Figure 19. Age-standardised population-attributable fraction of DALYs of stroke due to low daily ambient temperature, for both sexes, 2019

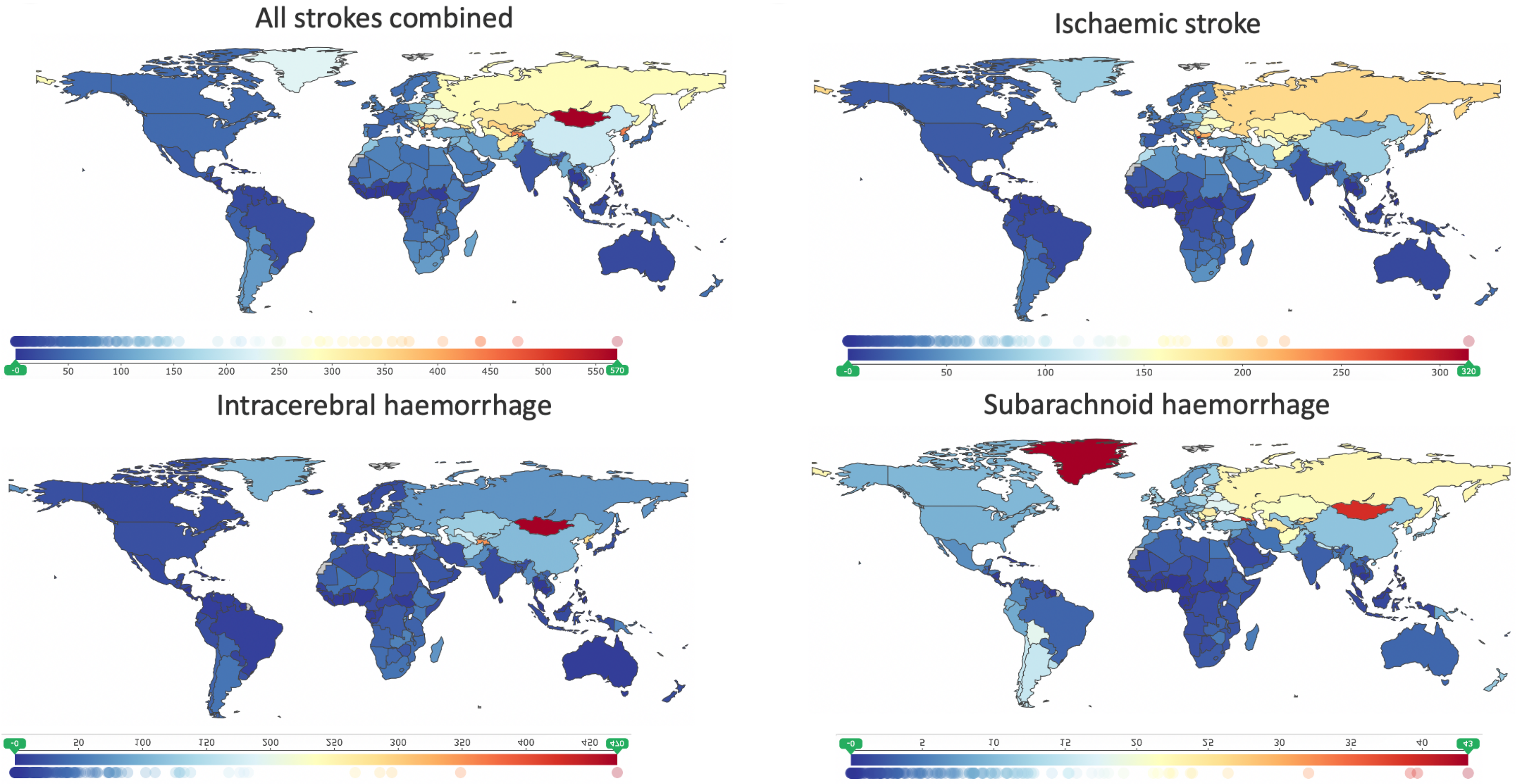

6.20 Supplement Figure 20. Age-standardised population-attributable fraction of DALYs of stroke due to high daily ambient temperature, for both sexes, 2019

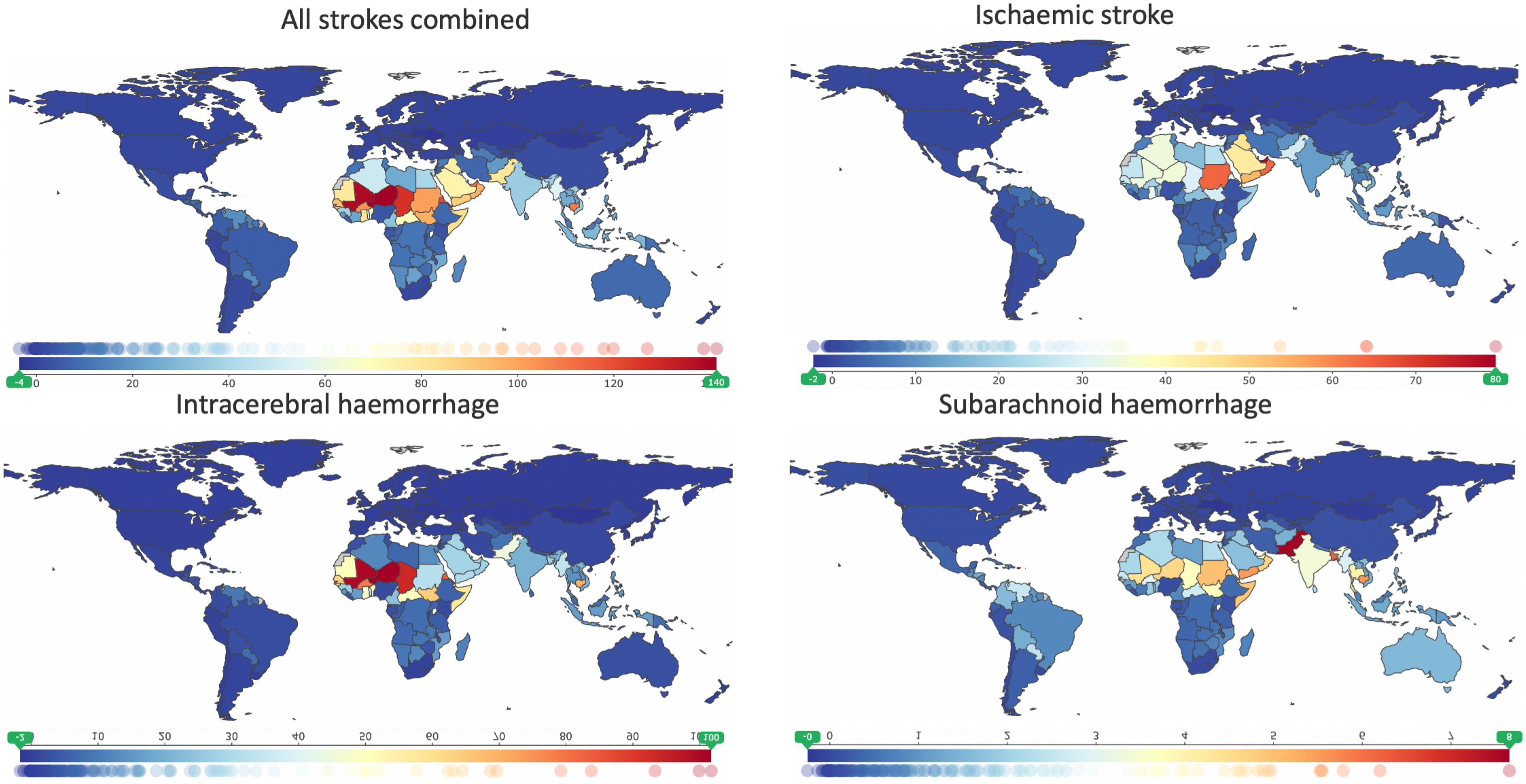

6.21 Supplement Figure 21. Stroke DALYs attributable to all risk factors combined, all ages, both sexes, 1990-2019

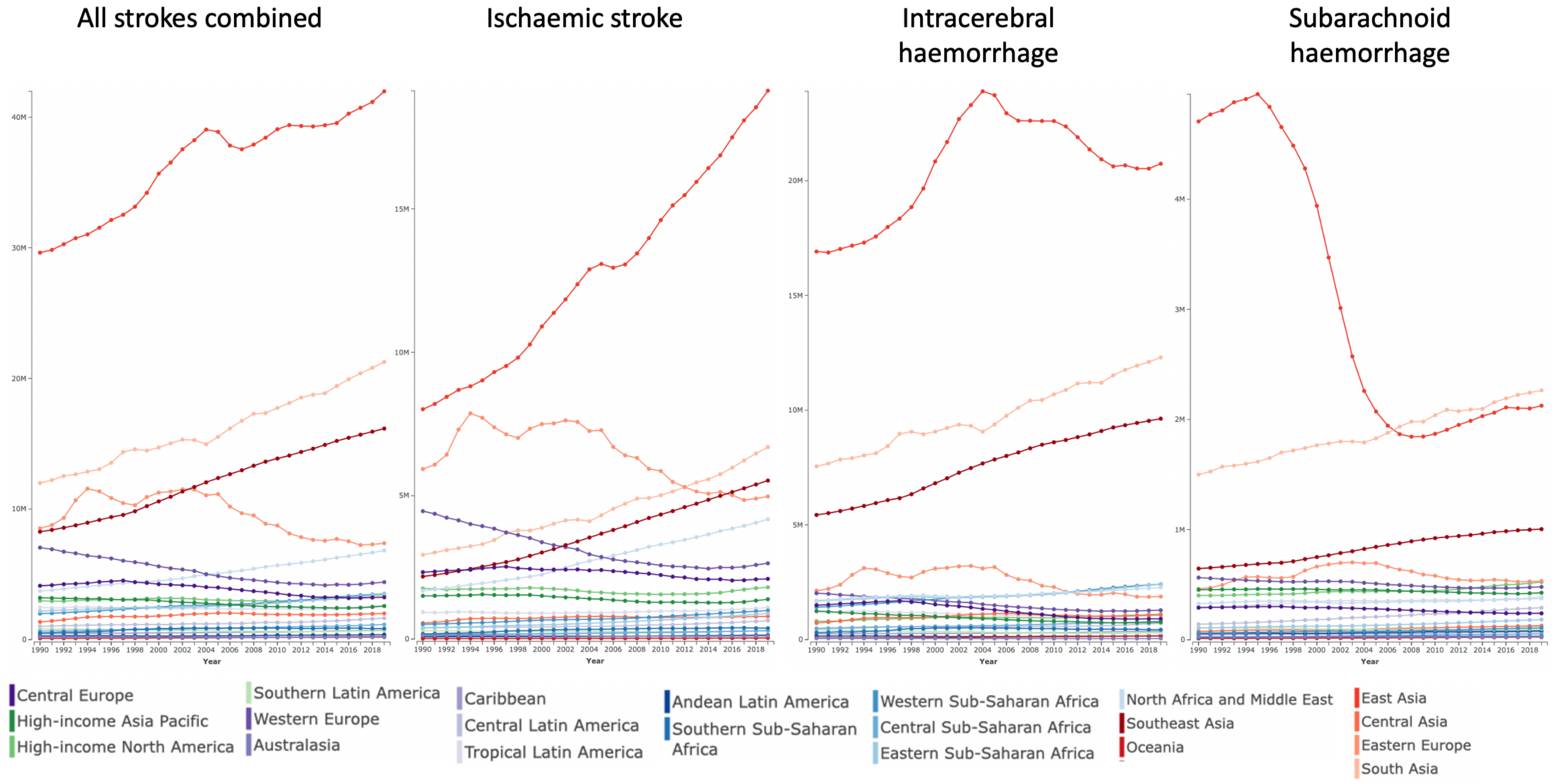

6.22 Supplement Figure 22. Age-standardised percentage of stroke DALYs (with 95% uncertainty intervals) attributable to all risk factors in males and females, 2019

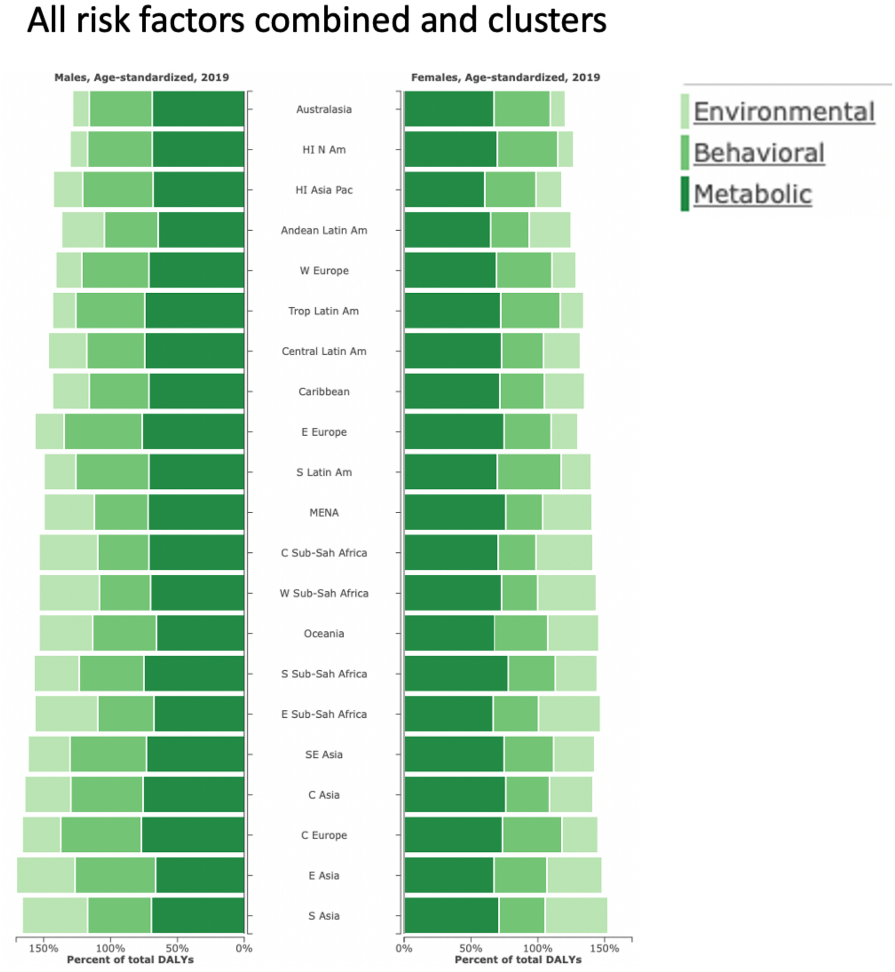

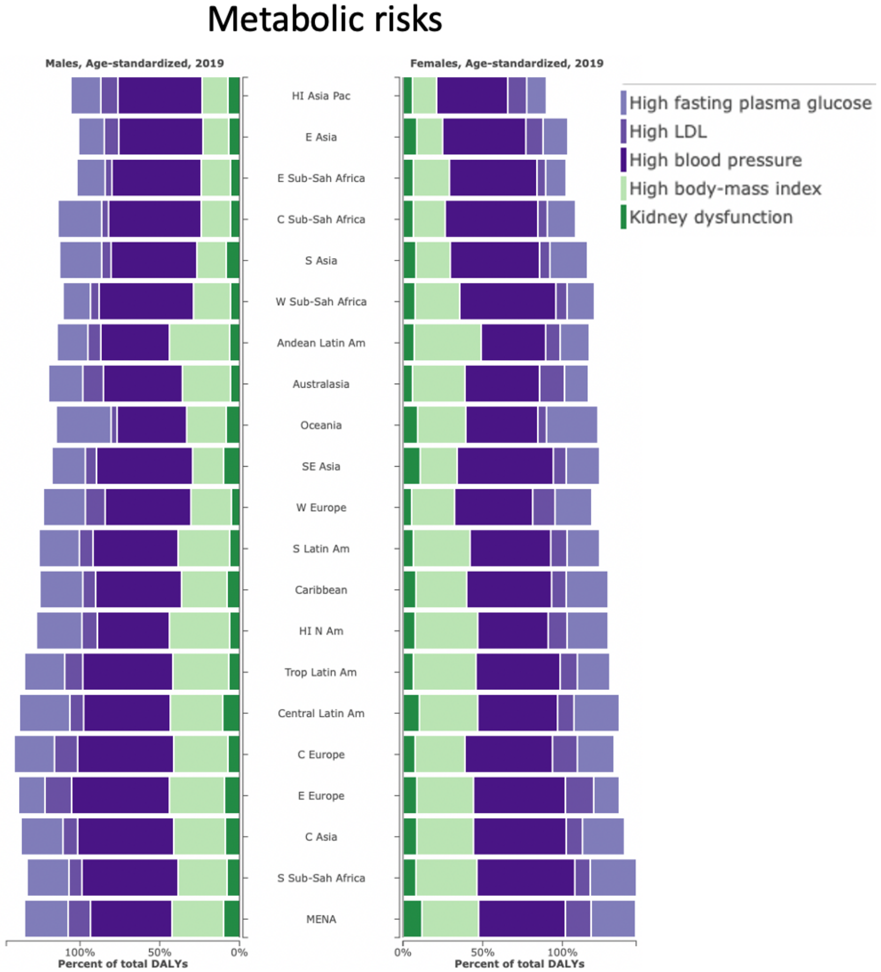

Behavioural risks

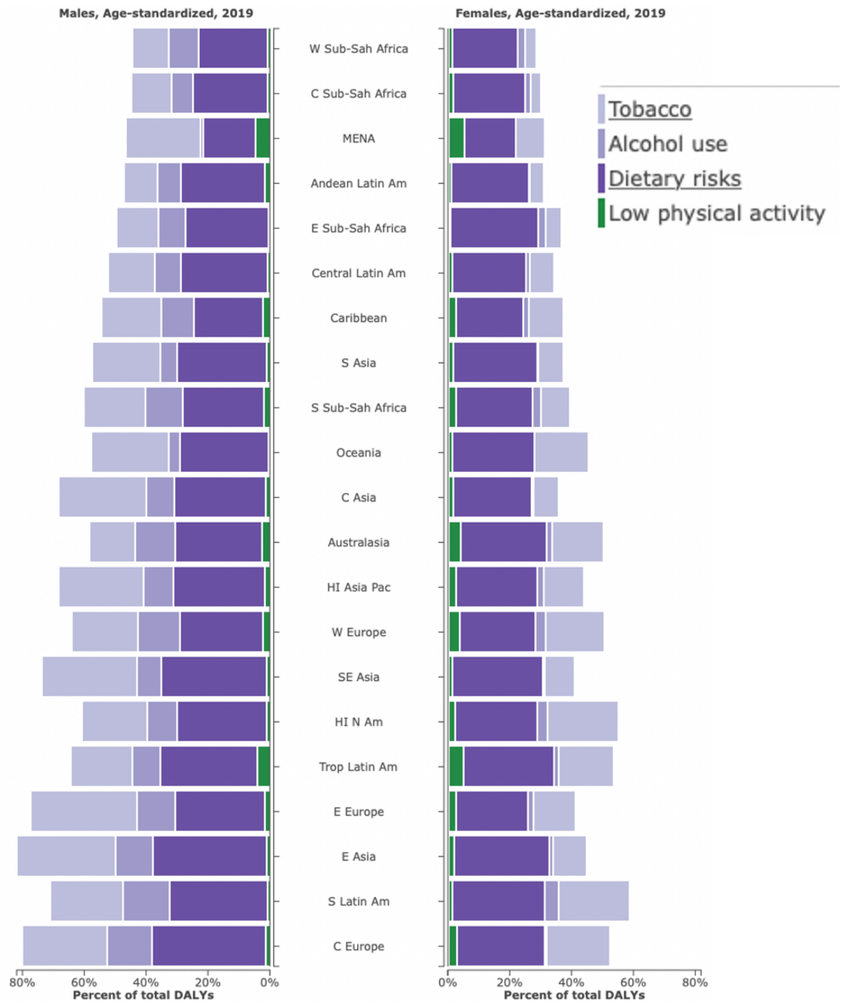

Environmental/occupational

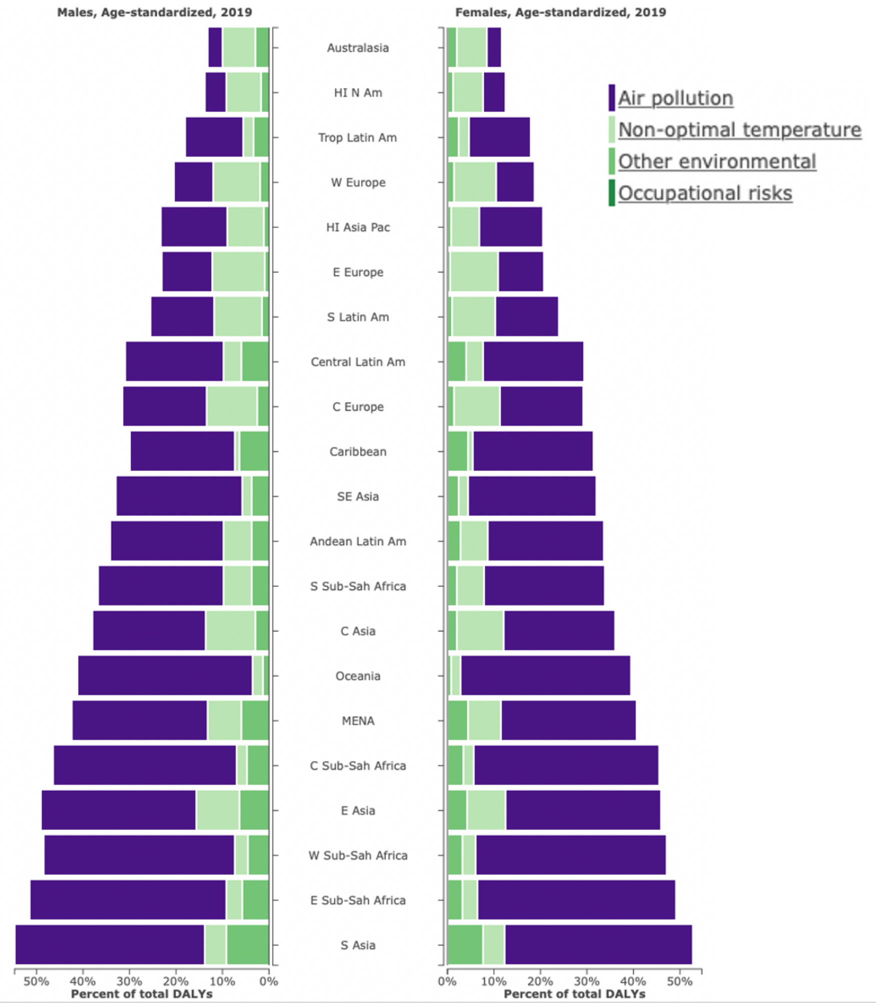

6.23 Supplement Figure 23. Age-standardised percentage of ischaemic stroke DALYs (with 95% uncertainty intervals) attributable to all risk factors in males and females, 2019

All risk factors combined and clusters

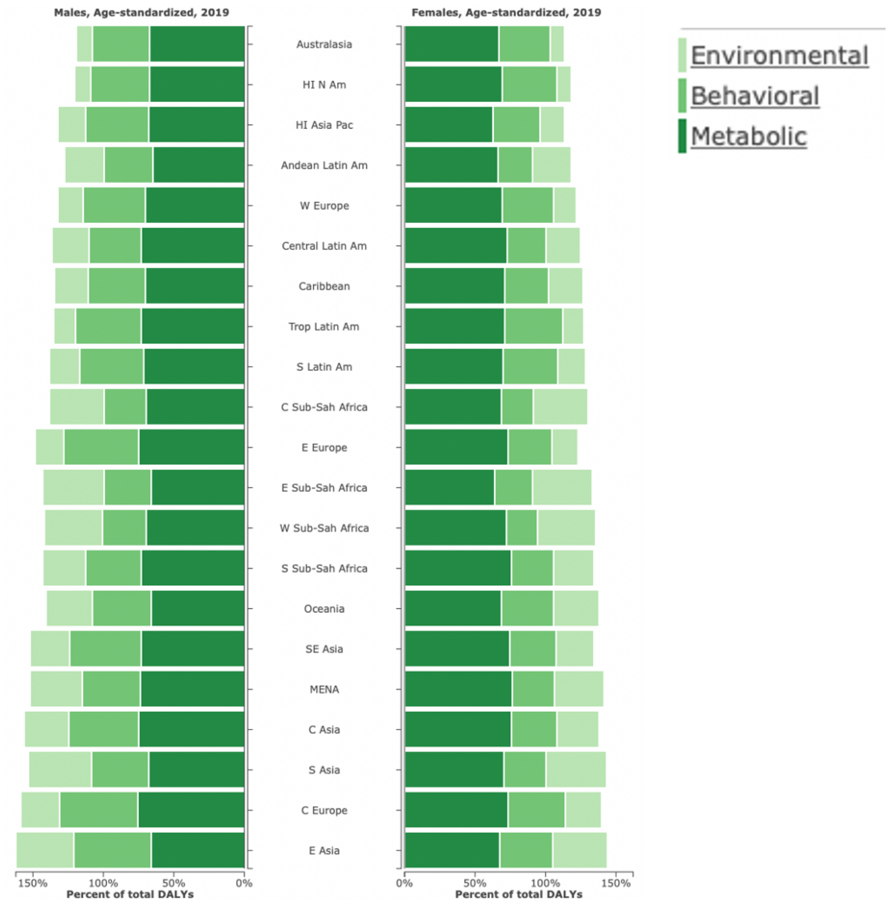

Metabolic risks

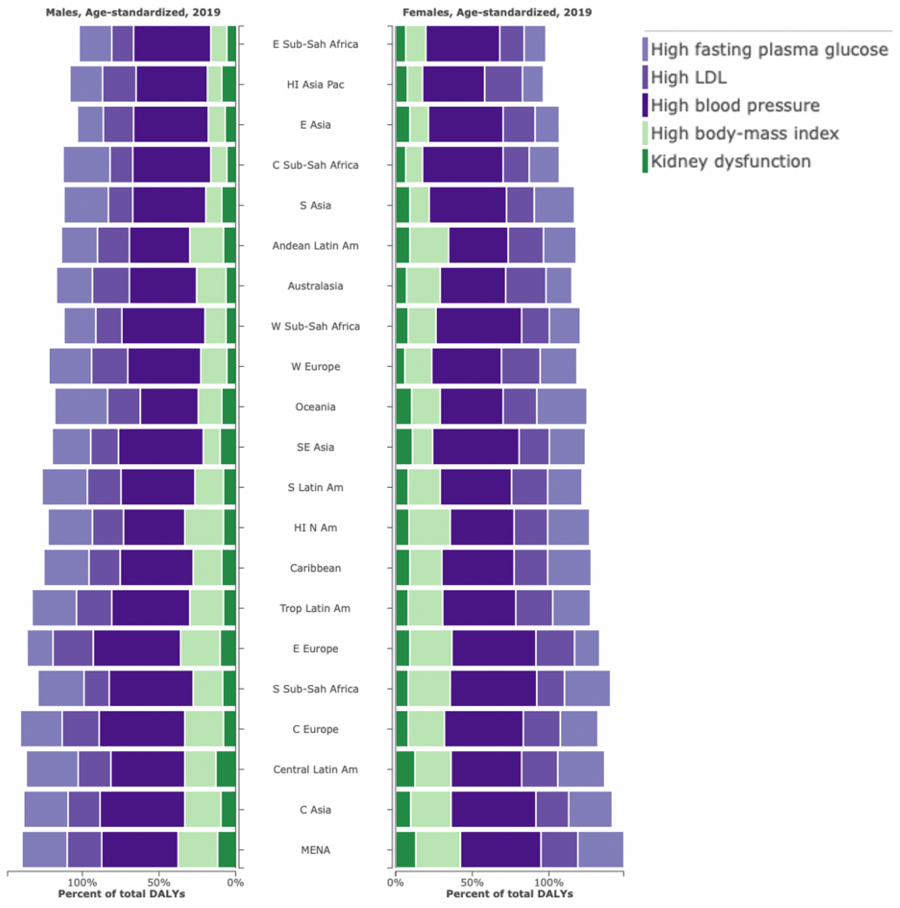

Behavioural risks

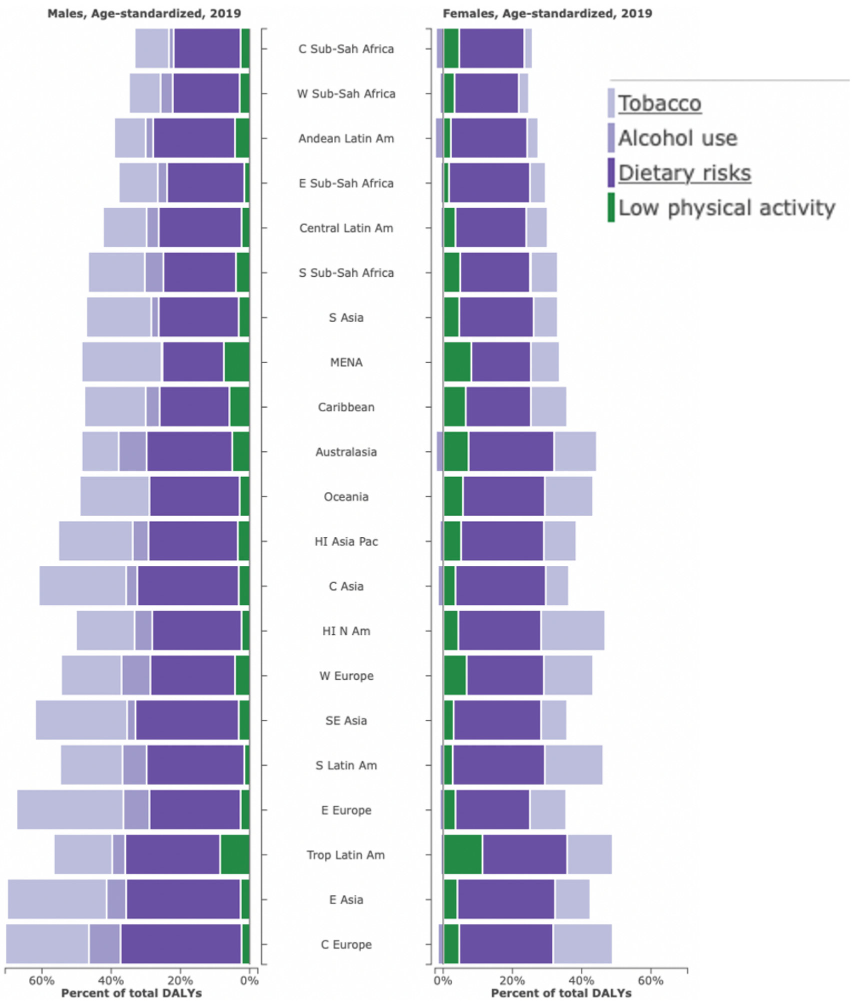

Environmental/occupational

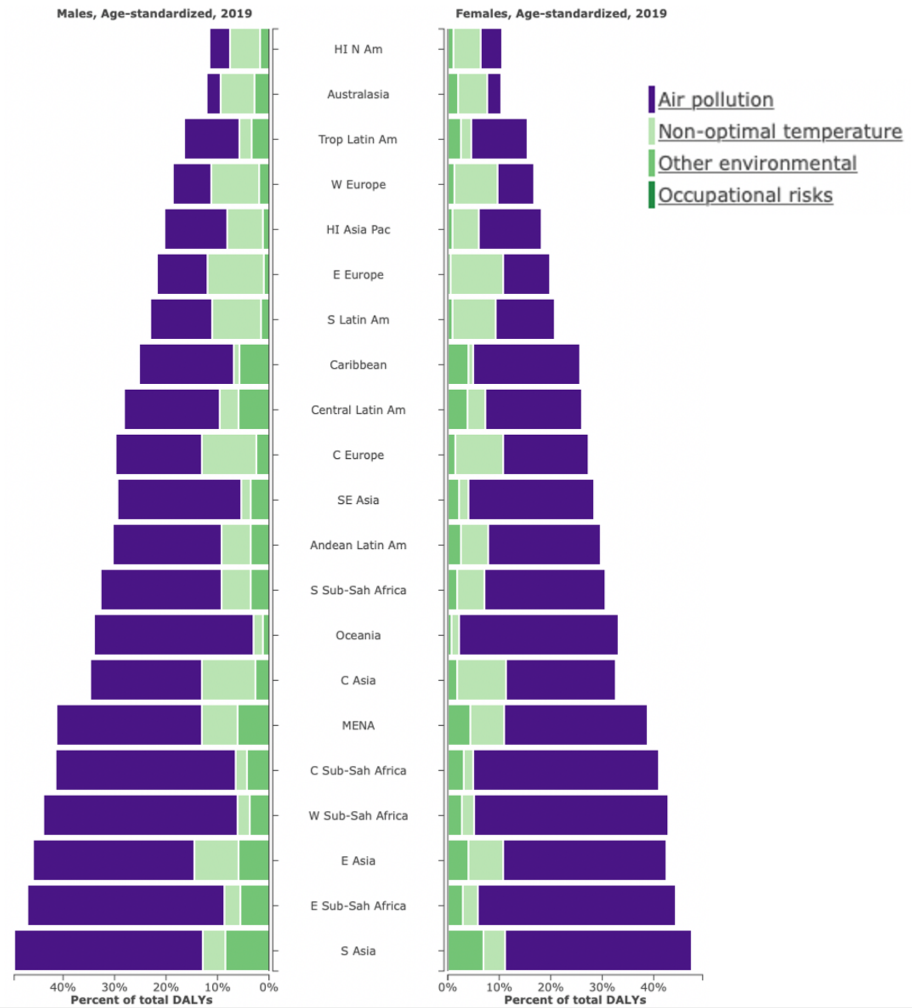

6.24 Supplement Figure 24. Age-standardised percentage of intracerebral haemorrhage DALYs (with 95% uncertainty intervals) attributable to all risk factors in males and females, 2019

All risk factors combined and clusters

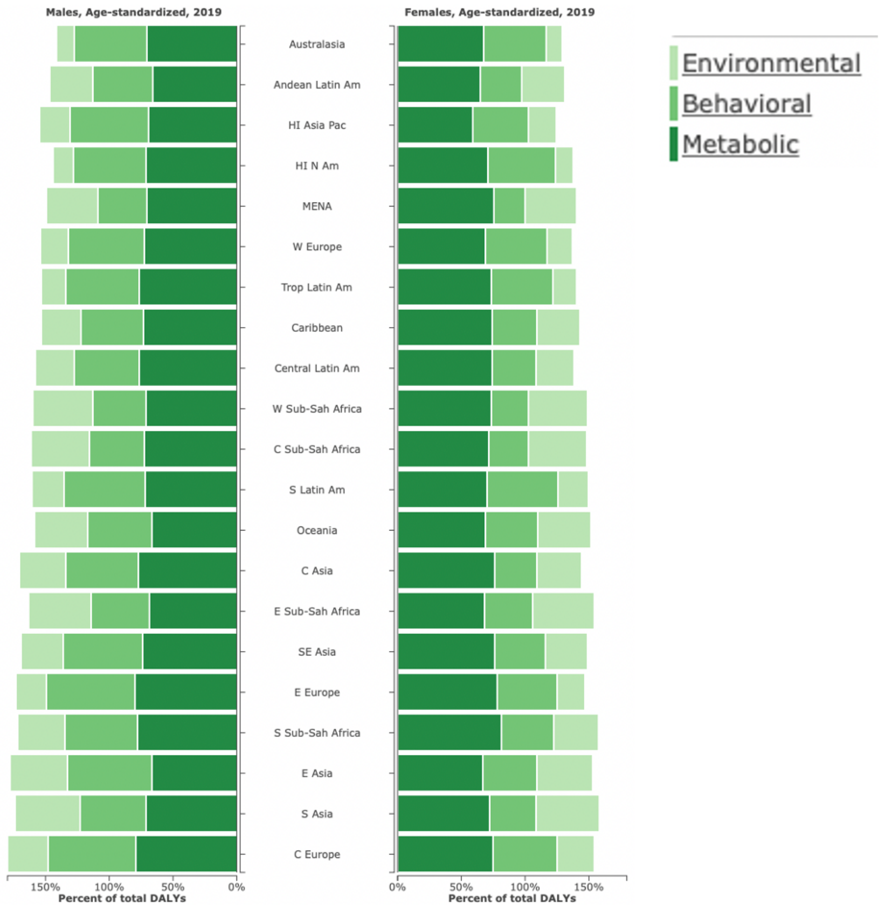

Metabolic risks

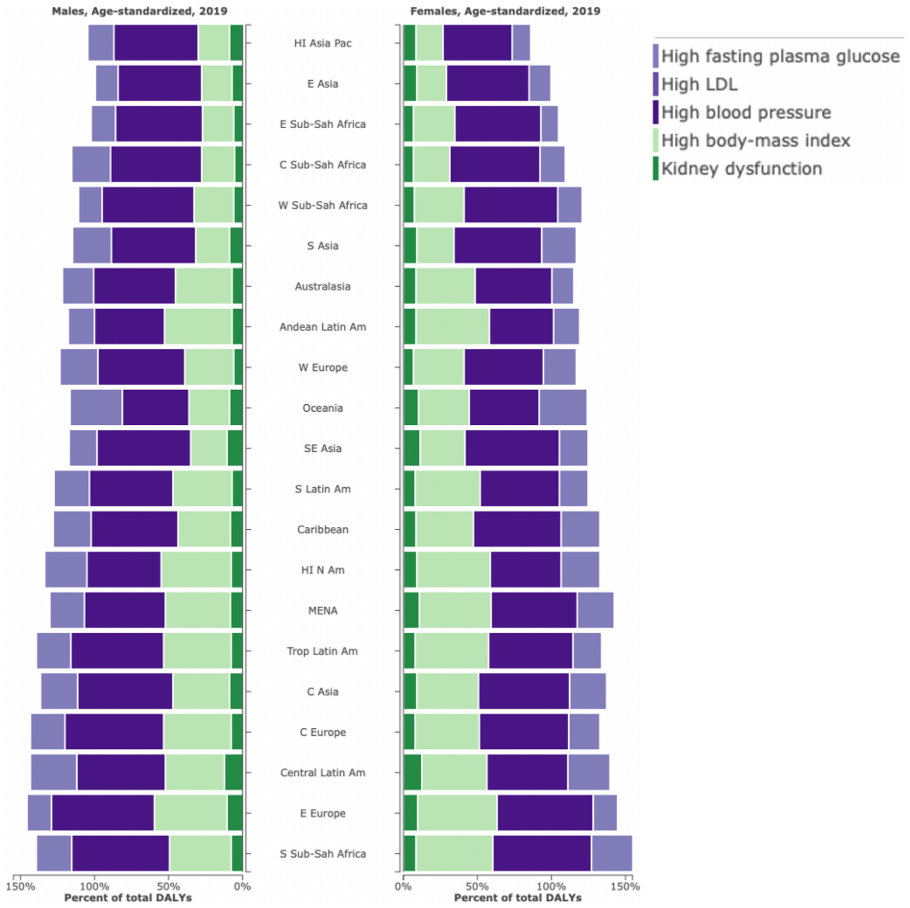

Behavioural risks

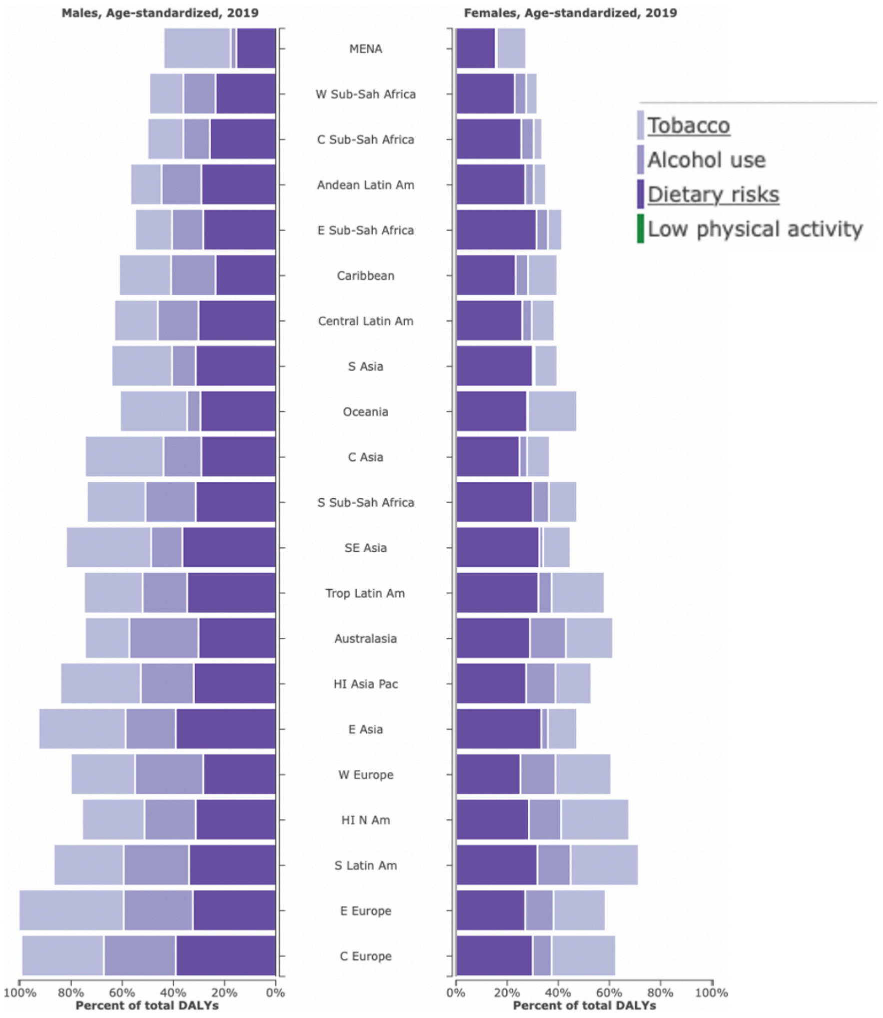

Environmental/occupational

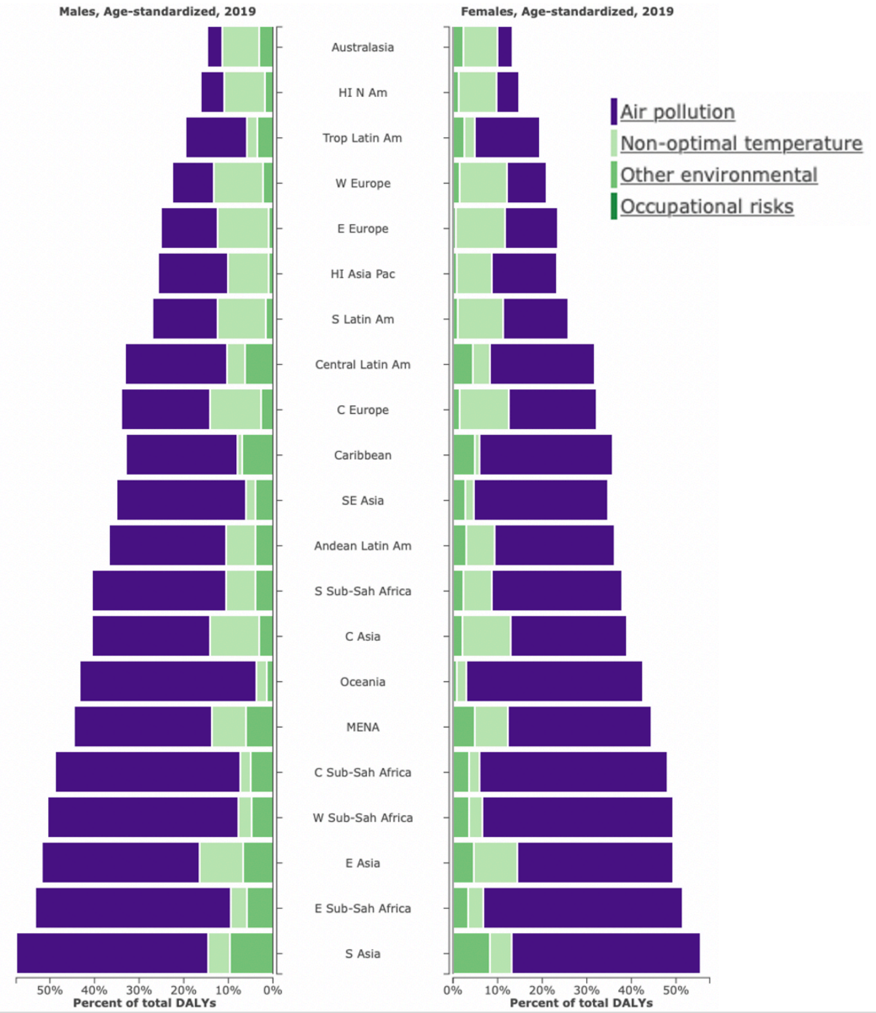

6.25 Supplement Figure 25. Age-standardised percentage of subarachnoid haemorrhage DALYs (with 95% uncertainty intervals) attributable to all risk factors in males and females, 2019

All risk factors combined and clusters

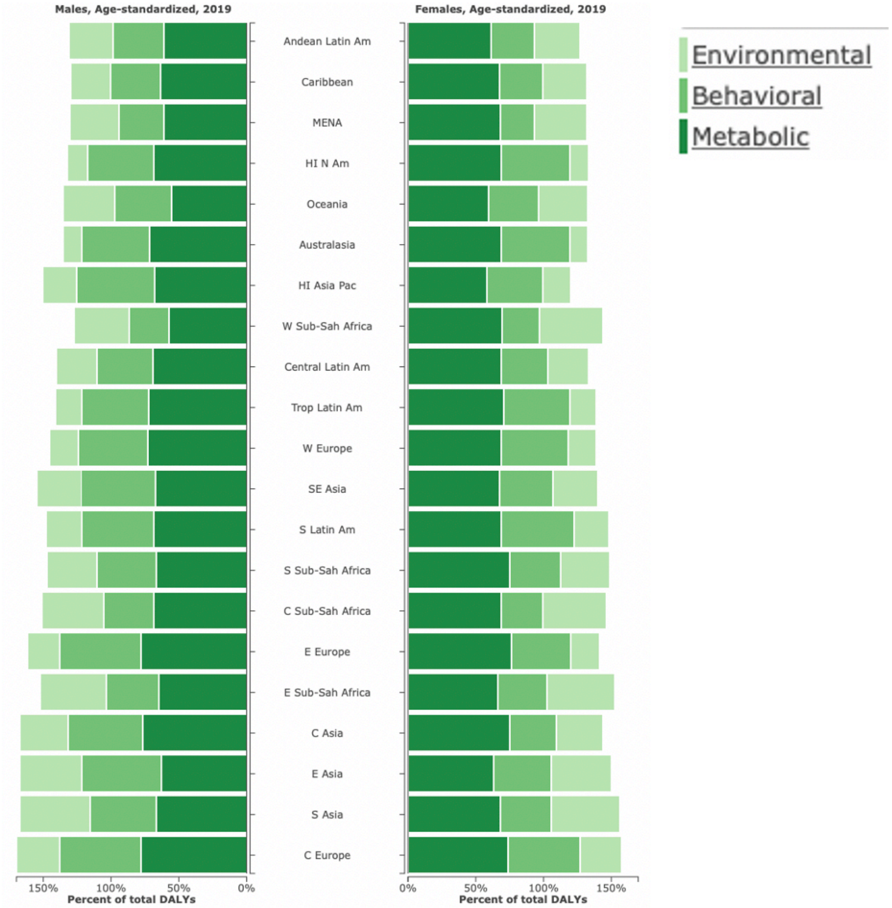

Metabolic risks

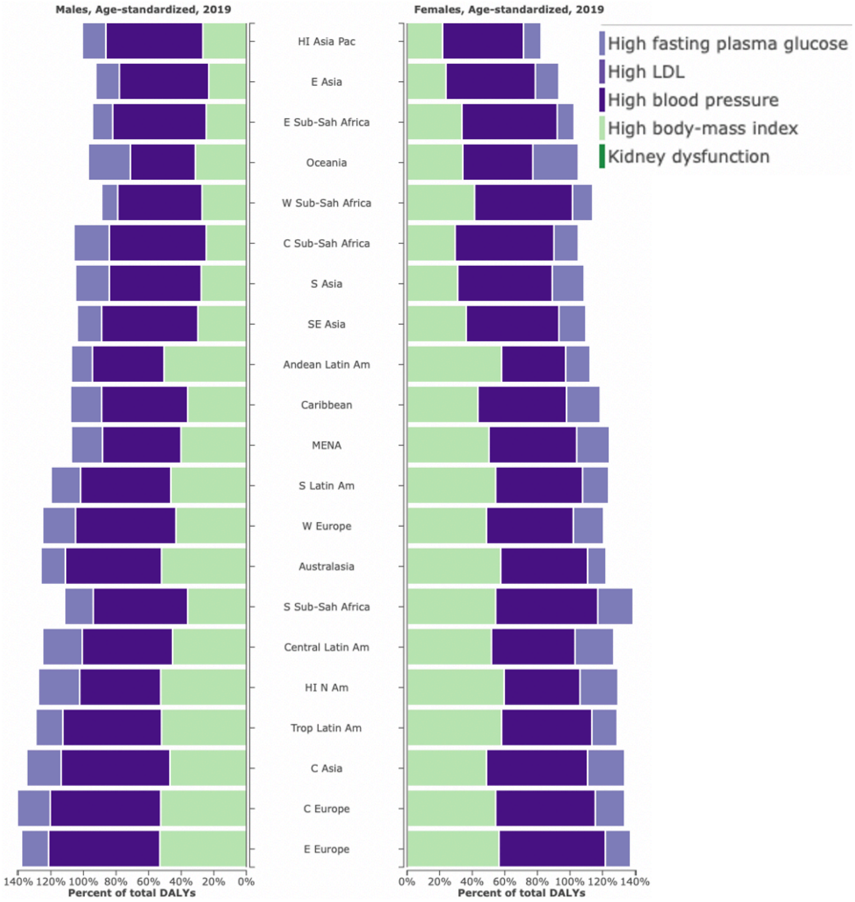

Behavioural risks

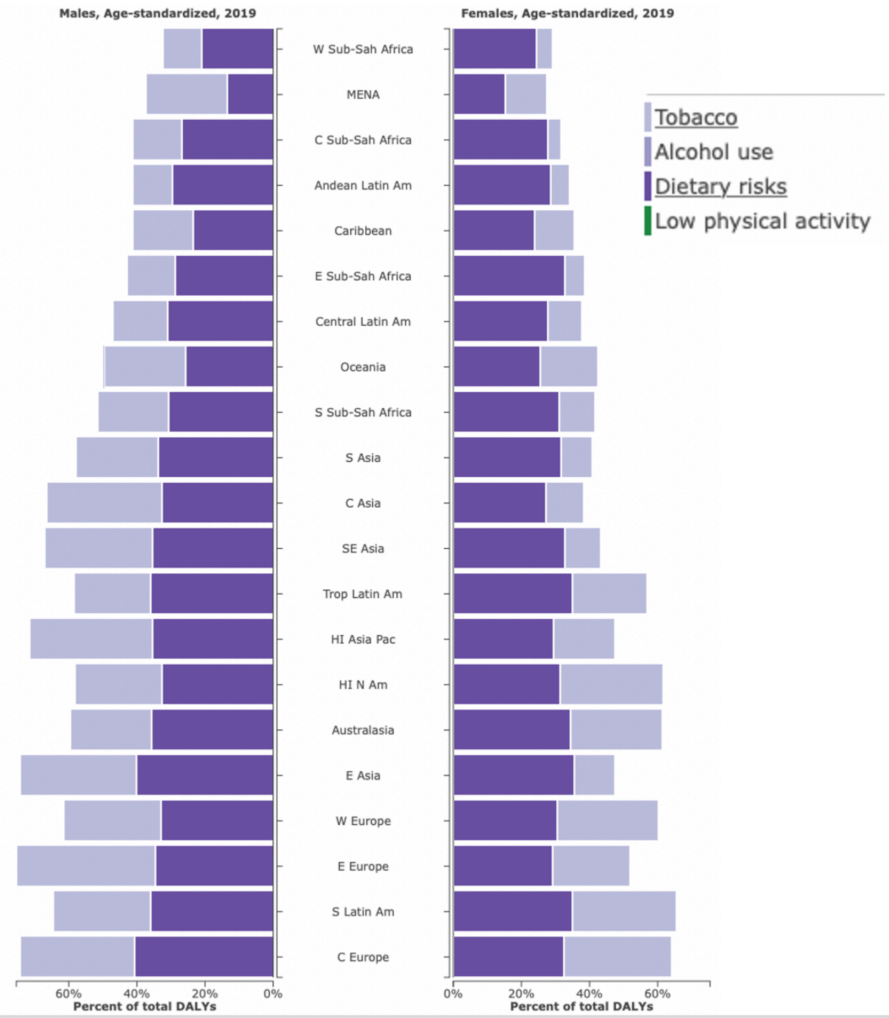

Environmental/occupational

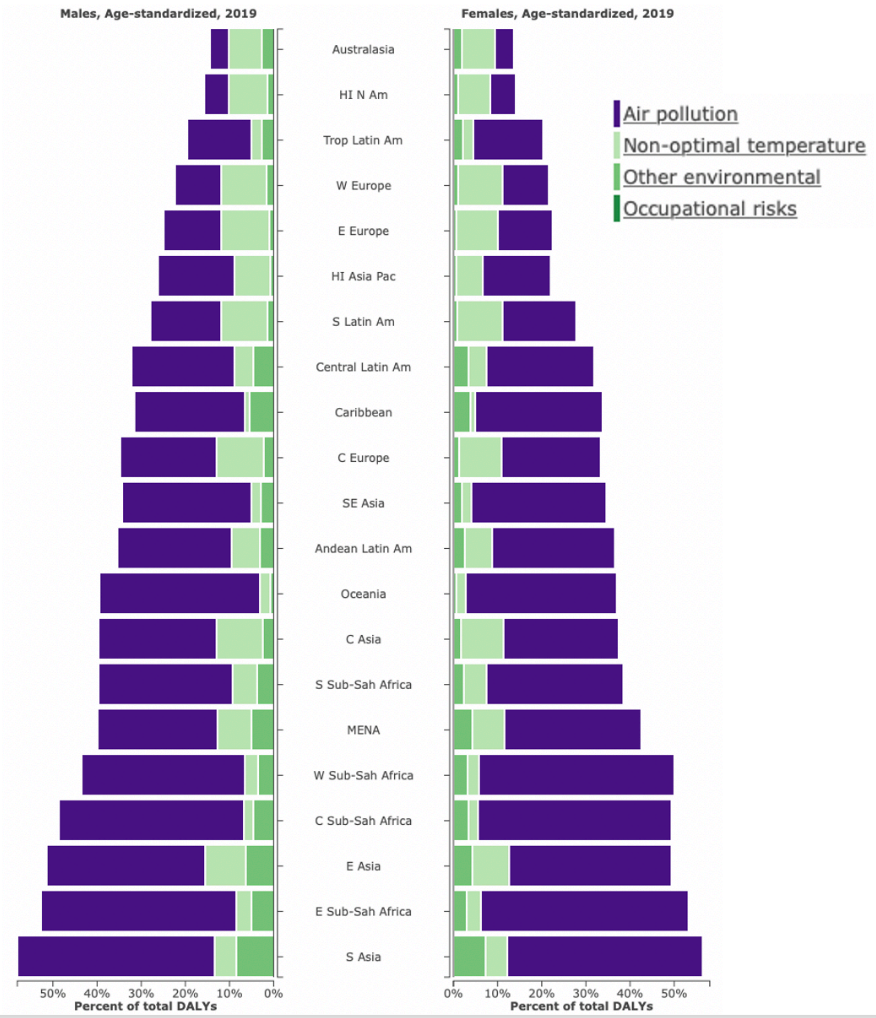

6.26 Supplement Figure 26. Years of healthy life lost (DALYs) in millions, 2019

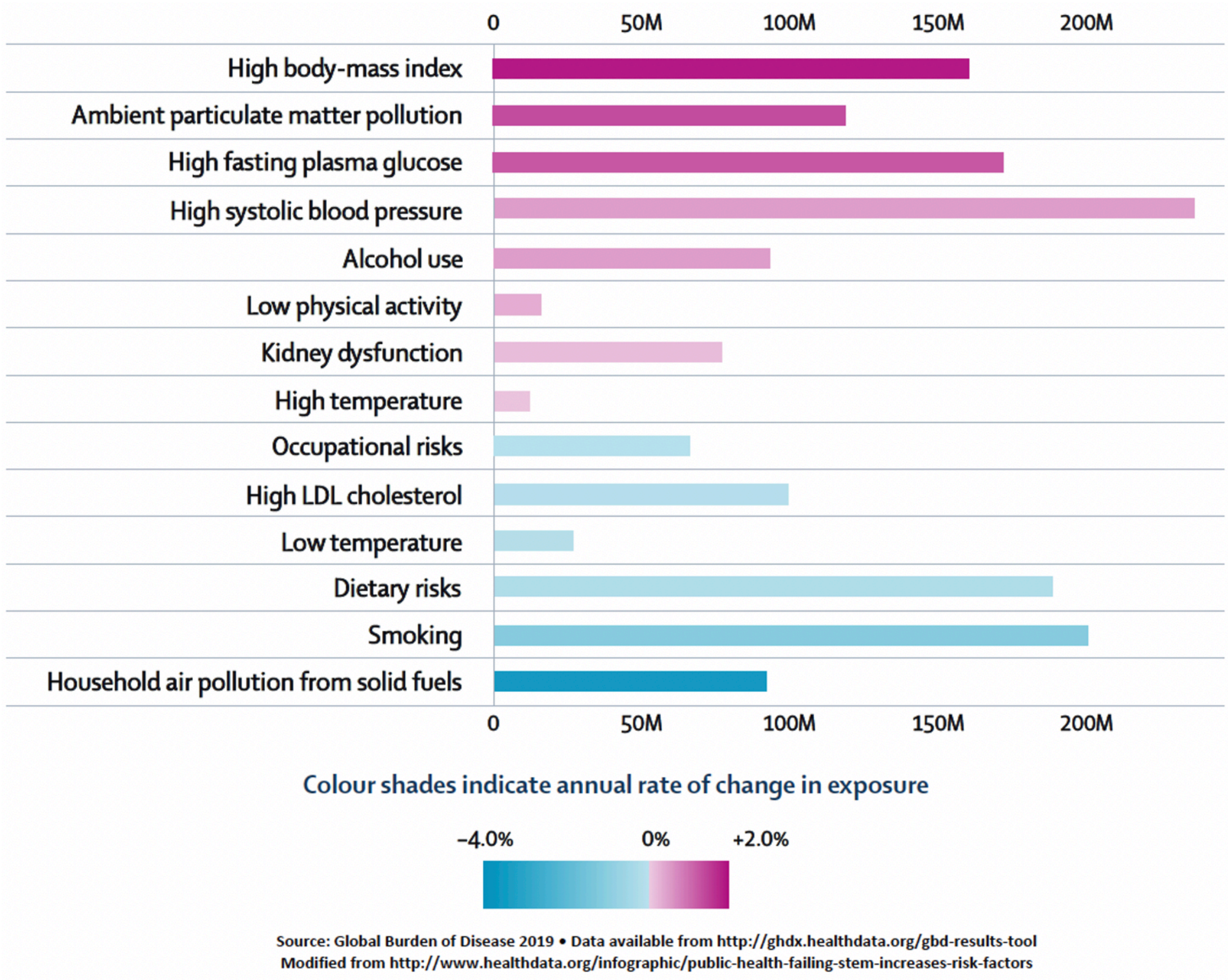

REFERENCES

1. Vos T, Lim SS, Abbafati C, Abbas KM, Abbasi M, Abbasifard M, et al. Global burden of 369 diseases and injuries in 204 countries and territories, 1990–2019: A systematic analysis for the global burden of disease study 2019. *The Lancet*. 2020;396:1204-1222
2. Murray CJL, Aravkin AY, Zheng P, Abbafati C, Abbas KM, Abbasi-Kangevari M, et al. Global burden of 87 risk factors in 204 countries and territories, 1990-2019: A systematic analysis for the global burden of disease study 2019. *The Lancet*. 2020;396:1223-1249
3. Johnson CO, Nguyen M, Roth GA, Nichols E, Alam T, Abate D, et al. Global, regional, and national burden of stroke, 1990-2016: A systematic analysis for the global burden of disease study 2016. *The Lancet Neurology*. 2019;18:439-458
4. Roth GA, Abate D, Abate KH, Abay SM, Abbafati C, Abbasi N, et al. Global, regional, and national age-sex-specific mortality for 282 causes of death in 195 countries and territories, 1980–2017: A systematic analysis for the global burden of disease study 2017. *The Lancet*. 2018;392:1736-1788
5. James SL, Abate D, Abate KH, Abay SM, Abbafati C, Abbasi N, et al. Global, regional, and national incidence, prevalence, and years lived with disability for 354 diseases and injuries for 195 countries and territories, 1990-2017: A systematic analysis for the global burden of disease study 2017. *The Lancet*. 2018;392:1789-1858
6. Kyu HH, Abate D, Abate KH, Abay SM, Abbafati C, Abbasi N, et al. Global, regional, and national disability-adjusted life-years (dalys) for 359 diseases and injuries and healthy life expectancy (hale) for 195 countries and territories, 1990-2017: A systematic analysis for the global burden of disease study 2017. *The Lancet*. 2018;392:1859-1922
7. Stanaway JD, Afshin A, Gakidou E, Lim SS, Abate D, Abate KH, et al. Global, regional, and national comparative risk assessment of 84 behavioural, environmental and occupational, and metabolic risks or clusters of risks for 195 countries and territories, 1990-2017: A systematic analysis for the global burden of disease study 2017. *The Lancet*. 2018;392:1923-1994
8. Lozano R, Freeman MK, James SL, Campbell B, Lopez AD, Flaxman AD, et al. Performance of interva for assigning causes of death to verbal autopsies: Multisite validation study using clinical diagnostic gold standards. *Popul Health Metr*. 2011;9:50
9. Aho K, Harmsen P, Hatano S, Marquardsen J, Smirnov VE, Strasser T. Cerebrovascular disease in the community: Results of a who collaborative study. *Bulletin of the World Health Organization.* 1980;58:113-130
10. Bamford J, Sandercock P, Dennis M, Burn J, Warlow C. A prospective study of acute cerebrovascular disease in the community: The oxfordshire community stroke project--1981-86. 2. Incidence, case fatality rates and overall outcome at one year of cerebral infarction, primary intracerebral and subarachnoid haemorrhage. *Journal of Neurology, Neurosurgery & Psychiatry*. 1990;53:16-22
11. Anderson CS, Jamrozik KD, Broadhurst RJ, Stewart-Wynne EG. Predicting survival for 1 year among different subtypes of stroke. Results from the perth community stroke study. *Stroke*. 1994;25:1935-1944
12. Nilsson OG, Lindgren A, Brandt L, Saveland H. Prediction of death in patients with primary intracerebral hemorrhage: A prospective study of a defined population. *J Neurosurg.* 2002;97:531-536
13. Foreman K, Lozano R, Lopez A, Murray C. Modeling causes of death: An integrated approach using codem. *Population health metrics*. 2012;10:1
14. Feigin VL, Roth GA, Naghavi M, Parmar P, Krishnamurthi R, Chugh S, et al. Global burden of stroke and risk factors in 188 countries, during 1990-2013: A systematic analysis for the global burden of disease study 2013. *The Lancet Neurology*. 2016;15:913-924
15. Murray CJ, Lopez AD. Global mortality, disability, and the contribution of risk factors: Global burden of disease study [see comments]. *Lancet*. 1997;349:1436-1442
16. Murray CJL, Lopez AD. On the comparable quantification of health risks: Lessons from the global burden of disease study. *Epidemiology*. 1999;10:594-605

17. Forouzanfar MH, Afshin A, Alexander LT, Anderson HR, Bhutta ZA, Biryukov S, et al. Global, regional, and national comparative risk assessment of 79 behavioural, environmental and occupational, and metabolic risks or clusters of risks, 1990-2015: A systematic analysis for the global burden of disease study 2015. *The Lancet*. 2016;388:1659-1724
18. Stevens GA, Alkema L, Black RE, Boerma JT, Collins GS, Ezzati M, et al. Guidelines for accurate and transparent health estimates reporting: The gather statement. *The Lancet*. 2016;388:e19-e23
19. Food, nutrition, physical activity and the prevention of cancer: A global perspective. Washington, d.C: World cancer research fund & american institute for cancer research, 2007. Https://discovery.Ucl.Ac.Uk/id/eprint/4841/1/4841.Pdf accesssed 6 november 2020.
20. Aravkin A, Davis D. Trimmed statistical estimation via variance reduction. *Mathematics of Operations Research*. 2019;45:292-322
21. Pavarin RM. Cocaine consumption and death risk: A follow-up study on 347 cocaine addicts in the metropolitan area of bologna. *Ann Ist Super Sanita*. 2008;44:91-98
22. Lim SS, Carnahan E, Nelson EC, Gillespie CW, Mokdad AH, Murray CJL, et al. Validation of a new predictive risk model: Measuring the impact of the major modifiable risks of death for patients and populations. *Population Health Metrics*. 2015;13:27
23. Danaei G, Singh GM, Paciorek CJ, Lin JK, Cowan MJ, Finucane MM, et al. The global cardiovascular risk transition: Associations of four metabolic risk factors with national income, urbanization, and western diet in 1980 and 2008. *Circulation*. 2013;127:1493-1502, 1502e1491-1498
24. Das gupta p. Standardization and decomposition of rates: A user's manual. Washington d.C.: U.S. Bureau of the census, 1993.
